# Analysis of Upstream Regulators, Networks, and Pathways Associated With the Expression Patterns of Polycystic Ovary Syndrome Candidate Genes During Fetal Ovary Development

**DOI:** 10.3389/fgene.2021.762177

**Published:** 2022-02-07

**Authors:** Rafiatu Azumah, Katja Hummitzsch, Monica D. Hartanti, Justin C. St. John, Richard A. Anderson, Raymond J. Rodgers

**Affiliations:** ^1^ Discipline of Obstetrics and Gynaecology, School of Medicine, Robinson Research Institute, The University of Adelaide, Adelaide, SA, Australia; ^2^ Faculty of Medicine, Universitas Trisakti, Jakarta, Indonesia; ^3^ MRC Centre for Reproductive Health, Queen’s Medical Research Institute, University of Edinburgh, Edinburgh, United Kingdom

**Keywords:** polycystic ovary syndrome, mitochondrial dysfunction, stromal expansion, steroidogenesis, upstream regulators, fetal ovary

## Abstract

Polycystic Ovary Syndrome (PCOS) is a multifactorial syndrome with reproductive, endocrine, and metabolic symptoms, affecting about 10% women of reproductive age. Pathogenesis of the syndrome is poorly understood with genetic and fetal origins being the focus of the conundrum. Genetic predisposition of PCOS has been confirmed by candidate gene studies and Genome-Wide Association Studies (GWAS). Recently, the expression of PCOS candidate genes across gestation has been studied in human and bovine fetal ovaries. The current study sought to identify potential upstream regulators and mechanisms associated with PCOS candidate genes. Using RNA sequencing data of bovine fetal ovaries (62–276 days, *n* = 19), expression of PCOS candidate genes across gestation was analysed using Partek Flow. A supervised heatmap of the expression data of all 24,889 genes across gestation was generated. Most of the PCOS genes fell into one of four clusters according to their expression patterns. Some genes correlated negatively (early genes; *C8H9orf3*, *TOX3*, *FBN3*, *GATA4*, *HMGA2*, and *DENND1A*) and others positively (late genes; *FDFT1*, *LHCGR*, *AMH*, *FSHR*, *ZBTB16*, and *PLGRKT*) with gestational age. Pathways associated with PCOS candidate genes and genes co-expressed with them were determined using Ingenuity pathway analysis (IPA) software as well as DAVID Bioinformatics Resources for KEGG pathway analysis and Gene Ontology databases. Genes expressed in the early cluster were mainly involved in mitochondrial function and oxidative phosphorylation and their upstream regulators included *PTEN*, *ESRRG/A* and *MYC*. Genes in the late cluster were involved in stromal expansion, cholesterol biosynthesis and steroidogenesis and their upstream regulators included *TGFB1/2/3*, *TNF, ERBB2/3*, *VEGF*, *INSIG1*, *POR*, and *IL25*. These findings provide insight into ovarian development of relevance to the origins of PCOS, and suggest that multiple aetiological pathways might exist for the development of PCOS.

## Introduction

Polycystic Ovary syndrome (PCOS) is a debilitating syndrome with reproductive, endocrine and metabolic symptoms, affecting up to 10% women of reproductive age, with about 72% suffering infertility due to anovulation (Joham et al., 2015). Hyperandrogenism, oligo-/anovulation and the presence of polycystic ovary morphology are the cardinal features of the syndrome. However, hyperinsulinemia, type 2 diabetes mellitus, and obesity are also associated with PCOS [reviewed in detail by Teede et al. (2010)]. Despite the high prevalence of the syndrome, a comprehensive mechanism elucidating its pathophysiology is still lacking. Research efforts to define the pathophysiology of PCOS have increased the scientific conundrum surrounding its developmental origin(s) due to the diverging nature of research outcomes.

The multifactorial aetiology of PCOS has been evident by numerous genomic studies ranging from candidate gene approaches to Genome-Wide Association Studies (GWAS) – confirming its genetic origin. Candidate gene studies have identified androgen receptor (*AR*) (Schüring et al., 2012), fibrillin 3 (*FBN3*) (Hatzirodos et al., 2011), and anti-Mullerian hormone (*AMH*) (Tata et al., 2018) among others to be related to PCOS. GWAS have also identified 19 PCOS susceptibility loci among the Han Chinese, European and Korean ancestry [reviewed in detail by McAllister et al. (2015); Jones and Goodarzi (2016); Hiam et al. (2019)]. More so, the fetal origin of PCOS has been studied in human (Davies et al., 2012; Echiburú et al., 2020; Mills et al., 2020) and animal models including mice (Sullivan and Moenter 2004), rats (Wu et al., 2010), sheep (Birch et al., 2003), and monkeys (Dumesic et al., 1997). The possible fetal predisposition of the syndrome has also been studied further in bovine and human fetal ovaries using qRT-PCR technique (Hartanti et al., 2020; Liu et al., 2020).

Hartanti, et al. (2020), Liu, et al. (2020) showed that all PCOS candidate genes in/near loci identified by GWAS, except small ubiquitin like modifier 1 pseudogene 1 (*SUMO1P1*), and three additional candidate genes *AR*, *AMH* and transforming growth factor beta 1 induced transcript 1 (*TGFB1I1*) were expressed in human and bovine fetal ovaries. Three distinct expression patterns were observed; some genes were highly expressed during early stages, others during late stages whilst others were expressed throughout gestation (Table 1). Notably, the mRNA levels of genes within the early and late groups significantly correlated with each other as well as mean age (Hartanti, et al., 2020; Liu, et al., 2020). The expression of these PCOS genes during fetal development has provided further insight to the fetal origin of the syndrome; necessitating further studies to delineate its pathophysiology. Thus, the molecular mechanisms and upstream regulators associated with PCOS candidate genes during fetal development that could probably lead to the expression of various phenotypes (such as lean, obese, insulin resistant) during adulthood still remain elusive.

**TABLE 1:** The three distinct expression patterns of PCOS candidate genes observed during fetal ovary development according to Hartanti, et al. (2020), Liu, et al. (2020); early, late and throughout gestation.

Early	Late	Throughout
Fibrillin 3 (*FBN3*)	Insulin receptor (*INSR*)	Thyroid adenoma associated (*THADA*)
GATA binding protein 4 (*GATA4*)	Follicle stimulating hormone receptor (*FSHR*)	Erb-B2 receptor tyrosine kinase 4 (*ERBB4*)
High mobility group AT-hook 2 (*HMGA2*)	Plasminogen receptor with a C-terminal lysine *(PLGRKT)*	DNA repair protein (*RAD50*)
TOX high mobility group box family member 3 (*TOX3)*	Zinc finger and BTB domain containing 16 (*ZBTB16*)	Chromosome 9 open reading frame 3 (*C8H9orf3*)
DENN domain-containing 1A (*DENND1A)*	Interferon regulatory factor 1 (*IRF1*)	Yes associated protein 1 (*YAP1*)
Luteinising hormone/chorionic gonadotrophin receptor *(LHCGR*)	Transforming growth factor beta 1 induced transcript 1 (*TGFB1I1)*	Ras-related protein (*RAB5B*)
Follicle stimulating hormone beta subunit (*FSHB*)	Luteinizing hormone/choriogonadotropin receptor (*LHCGR*)	Sulphite oxidase (*SUOX*)
Erb-B2 receptor tyrosine kinase 3 (*ERBB3*)	Anti-mullerian hormone (*AMH)*	Ca^2+^/calmodulin-dependent protein kinase (*KRR1*)
—	Androgen receptor (*AR*)	ADP ribosylation factor like GTPase 14 effector protein (*ARL14EP*)
—	—	Farnesyl-diphosphate farnesyltransferase 1 (*FDFT1*)
—	—	Nei like DNA glycosylase 2 (*NEIL2*)
—	—	Microtubule associated protein RP/EB family member 1 (*MAPRE1*)

Therefore, there is a need to identify the upstream regulators and pathways that operate when PCOS candidate genes are expressed during normal ovary development. This will provide more insight on the possible perturbations during fetal ovary development that could lead to PCOS in adulthood and also help clarify some of the conundra surrounding the genetic and fetal origins of the disorder. Although studies to delineate the roles of PCOS candidate genes during fetal development are on-going, acquisition of human fetal ovary samples across gestation, especially from the third trimester, is a limitation. Considering the strong similarities between human and bovine ovaries in morphology and physiology, gestational length and the propensity for singleton pregnancies as well as the similarity in expression of PCOS candidate genes between human and bovine during early stages of fetal ovary development (Hartanti, et al., 2020; Liu, et al., 2020), this study seeks to define the upstream regulators and pathways that operate when PCOS candidate genes are expressed (Hartanti, et al., 2020; Liu, et al., 2020) using bovine fetal ovaries.

## Materials and Methods

### Bovine Fetal Ovary Collection

Fetal ovarian pairs across gestation (62–276 days, *n* = 19) were collected from pregnant *Bos taurus* cows at the abattoir of Midfield Meat International, Warrnambool, Victoria, Australia and were immediately frozen on dry ice on site and later stored in the laboratory at −80°C. These ovaries were scavenged from animals that were being processed for human consumption and were not owned by the authors or their institutions. As such the University of Adelaide’s Animal Ethics Committee only requires notification of this. To estimate the gestational age of fetal samples, the crown-rump length (CRL) was measured (Russe 1983).

### Sex Determination of Bovine Fetuses

Genomic DNA was extracted from the tail of fetuses with a CRL <10 cm using the Wizard SV Genomic DNA Purification System (Promega Australia, Alexandria, NSW, Australia) according to the manufacturer’s instructions. Two pairs of primers specific for a region in the Sex determining region Y (SRY) sequence (sense primer: 5′-TCA​CTC​CTG​CAA​AAG​GAG​CA-3′, antisense primer: 5′-TTA​TTG​TGG​CCC​AGG​CTT​G-3′), and for the 18S ribosomal RNA (18S) gene sequence were used for amplifying the genomic DNA in individual reactions as previously described (Hummitzsch et al., 2013).

### RNA Extraction and RNA Sequencing

Whole fetal bovine ovaries were homogenised in 1 ml Trizol^®^ (Thermo Fisher Scientific, Waltham, MA, United States) using the Mo Bio Powerlyser 24 (Mo Bio Laboratories Inc., Carlsbad, CA, United States) and RNA extracted according to manufacturer’s instructions. All samples were treated with DNase I (Promega/Thermo Fisher Scientific Australia Pty Ltd., Tullmarine, Vic, Australia). The RNA concentration and quality (RQI, Table 2) was then determined using the Experion™ RNA StdSens Analysis kit and the Experion™ Automated Electrophoresis System (Bio-Rad Laboratories Pty., Ltd., Gladesville, NSW, Australia). 500 ng/50 µl per well (96-well plate) of total RNA of each sample was used for RNA-seq.

**TABLE 2:** Characteristics of bovine fetal ovaries and RNA quality index (RQI).

Sample ID	Identical samples	Crown-rump length [cm]	Gestational age [days]	Gestational period	RQI
15/R12t	Y	7.7	62	Early I	9.0
15/R85t	Y	9	66	Early I	8.5
15/R86t	N	12	76	Early I	8.9
15/R74t	Y	14	82	Early I	9.1
15/R41t	N	17	91	Early II	9.3
15/R57t	N	19	98	Early II	9.2
15/R42t	Y	24	113	Early II	9.6
15/R51t	Y	28	124	Early II	9.6
15/R43t	Y	32	135	Early II	9.5
15/R2t	Y	39	154	Late	9.8
15/R44t	Y	45	170	Late	9.3
15/R1t	Y	58	201	Late	9.5
15/R45t	Y	74	234	Late	9.6
15/R33t	Y	80	245	Late	9.6
15/R47t	N	86	255	Late	9.4
15/R35t	Y	88	258	Late	9.5
15/R38t	Y	91	263	Late	9.3
15/R49t	N	93	266	Late	9.7
15/R50t	Y	100	276	Late	9.5

Identical samples refer to samples also analysed in Hartanti, et al. (2020), Liu, et al. (2020).

RNA-seq based transcriptome profiling was performed at the SAHMRI Genomics Facility (SAHMRI, Adelaide, SA, Australia). Briefly, Single-end Poly A-selection mRNA libraries (∼35 M reads per sample) were created using the Nugen Universal Plus mRNA-Seq library kit from Tecan (Mannedorf, Switzerland) and sequenced with an Illumina Nextseq 500 using single read 75 bp (v2.0) sequencing chemistry (Illumina Inc., San Diego, CA, United States). Two sequencing runs, with 10 samples per run, were performed and sample 15/R43t was used as internal control in both runs.

### RNA-Seq Data Analysis Using Partek Flow^®^

The raw data containing FASTQ files were uploaded to Partek Flow^®^ Software, version 8.0 (Partek Incorporated, St. Louis, Missouri, United States). All samples underwent a pre-alignment quality assessment and showed Phred Quality Scores larger than 30. The reads were aligned and annotated to the bovine genome ARS-UCD1.2 (bosTau9; https://www.ncbi.nlm.nih.gov/assembly/GCF_002263795.1/) using STAR 2.7.3a aligner (>97% alignment rate for all samples) and Partek E/M, respectively. Transcript abundances were determined and expression levels presented as normalised counts per million (CPM). Initial comparison of gene expression profiles in the samples was then carried out using principal components analysis (PCA) (Hotelling 1933).

### Analysis of PCOS Candidate Genes

Normalised RNA-seq data of bovine fetal ovaries across gestation were analysed to study the expression of PCOS candidate genes as well as their associated upstream regulators and mechanisms. The expression of 27 PCOS candidate genes, most of which are located in/near the loci associated with PCOS from previous GWAS, and three additional candidate genes *AR*, *AMH*, and *TGFB1I1* were analysed using to Partek Flow^®^ Software (version 8.0). Scatter plots showing the expression patterns for each candidate gene across gestation were also generated using GraphPad Prism version 8 (GraphPad Software Inc., La Jolla, Ca, United States). Pearson’s correlation of the genes with each other as well as with gestational age were further analysed. Fold change and statistical significance of PCOS genes across gestation (comparing late versus early) were determined using the default parameters of Gene Specific Analysis (GSA); a multi-model approach based on Akaike Information Criterion (AIC) in Partek Flow^®^.

A supervised hierarchical clustering (heatmap) of all 24,889 genes identified in the RNA-seq data for all samples across gestation was then carried out; the location of PCOS genes on the heatmap was determined and clusters of PCOS genes as well as genes co-expressed with them were identified. Four clusters associated with the PCOS candidate genes were further studied using the core analysis component of Ingenuity Pathway Analysis (IPA^®^, QIAGEN Redwood City, CA, United States). The canonical pathways, upstream regulators and networks associated with each cluster were analysed based on the statistical significance (*p*-value) and fold change (log_2_ ratio) of its gene list, determined using the default parameters of GSA in Partek Flow^®^. *C9orf3,* also known as aminopeptidase *(AOPEP)* replaced its bovine variant, *C8H9orf3,* in IPA analysis. For a more in-depth understanding of pathways associated with genes in each of the four clusters, the Database for Annotation, Visualization, and Integrated Discovery (DAVID) Bioinformatics Resources 6.8 (https://david.ncifcrf.gov/home.jsp, Frederick, MD 21702, United States) (Huang et al., 2009; Sherman and Lempicki 2009), was further used to analyse the pathway enrichment of genes for each cluster from Gene Ontology (GO) and Kyoto Encyclopedia of Genes and Genomes (KEGG) databases for bovine organisms.

### Statistical Analyses

Expression of PCOS genes across gestation was plotted using GraphPad Prism version 8 (GraphPad Software Inc., La Jolla, CA, United States). PCA and hierarchical clustering were performed using the Euclidian algorithm for dissimilarity with average linkage. Pearson’s correlation table and correlation coefficient (R) generated for scatterplots were determined in Partek Genomic Suite 7.0 (Partek Incorporated, St. Louis, Missouri, United States). The *p*-value of overlap between a set of significant molecules in each cluster and a given process/pathway/upstream regulator is determined using the Right-Tailed Fisher’s Exact Test in IPA software. The z-score, which considers the directional effect of one molecule on another molecule/process and the direction of change of molecules in the dataset, represents activation when ≥2 and inhibition when ≤ −2. R program, version 4.0.4 was used to plot GO graphs using data from DAVID bioinformatic resources.

## Results

### Expression of PCOS Candidate Genes

**FIGURE 1:**
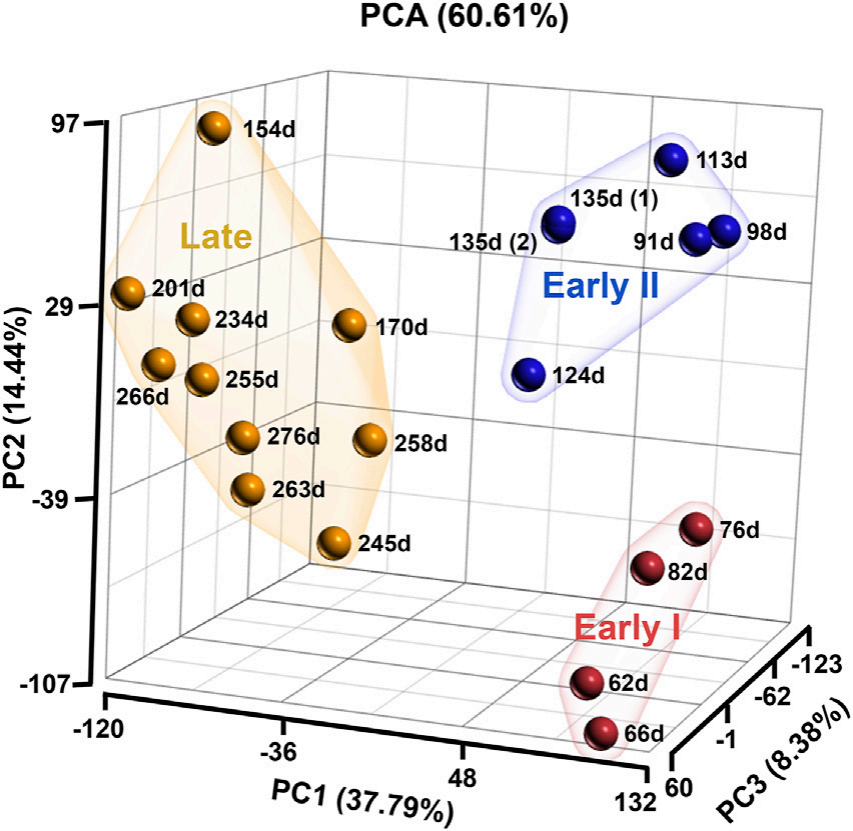
Principal Component analysis of bovine ovarian samples analysed in the RNA sequencing. The samples are grouping into three stages; early I (red; 62–82 days, *n* = 4), early II (blue; 91–135 days, *n* = 5) and late (orange; 154–276 days, *n* = 10).

The normalised RNA-seq data of bovine fetal ovaries identified 24,889 genes including uncharacterised and non-coding RNA. Principal Component Analysis (PCA) of all the genes in the data clustered the samples into three groups/stages; early I (62–82 days, *n* = 4), early II (91–135 days, *n* = 5), and late (154–276 days, *n* = 10) (Figure 1). For the purposes of this study, the two early groups were combined as “early” (62–135 days, *n* = 9) and all comparisons across gestation involved late versus early samples.

The expression of the 27 PCOS candidate genes identified in the RNA-seq data was studied across gestation. Using Pearson’s correlation, the mRNA expression of these candidate genes across gestation showed three patterns; early, late, and throughout gestation. Thus, some PCOS candidate genes correlate significantly with gestational age negatively whilst others positively (Table 3). Genes that correlate negatively with gestational age, and therefore named “early” genes, correlate positively with each other, whilst those that correlate positively with gestational age, named “late” genes, correlate positively with each other but negatively with the “early” genes. Thus, according to this study, the “early” genes consist of *C8H9orf3, ARL14EP, MAPRE1, TOX3, FBN3, GATA4, HMGA2,* and *DENND1A*; the “late” genes include *YAP1, INSR, THADA, TGFB1I1, ZBTB16, IRF1, LHCGR, FSHR, AMH,* and *PLGRKT*; whilst genes expressed throughout gestation are *NEIL2, RAB5B, KRR1, SUOX, FDFT1, ERBB3, AR, ERBB4,* and *RAD50* (Table 3). Also, Gene Specific Analysis (GSA) comparing the expression of these candidate genes across gestation showed significant fold change across gestation; 14 of the genes were up-regulated and 13 down-regulated with significance at *p* ≤ 0.05 for most candidate genes as shown in Supplementary Table S1. In addition to the “early” and “late” genes identified using Pearson’s correlation, other genes that correlate significantly with gestational age according to GSA are; *ERBB3* (negatively) as well as *AR* and *ERBB4* (positively); see Supplementary Table S1, Supplementary Figure S1.

**TABLE 3:** Pearson’s correlation coefficients (R) between PCOS candidate genes mRNA expression levels and gestational age (*n* = 19) in bovine fetal ovaries.

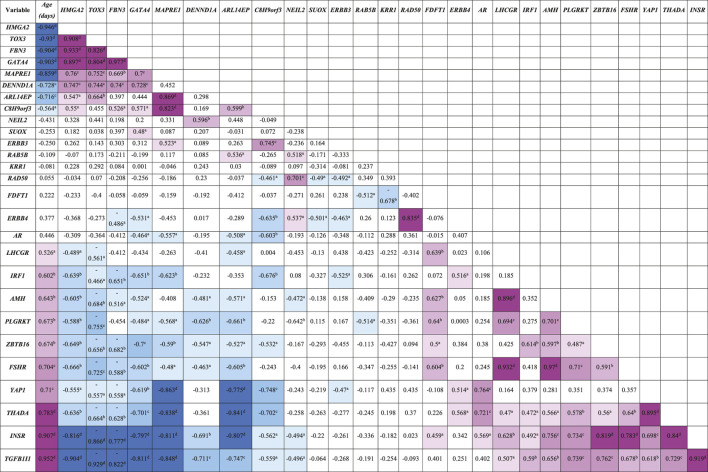

Positive and negative correlations are marked in pink and blue, respectively. The colour intensity corresponds with the strength of the correlation. *p*-values: a <0.05; b < 0.01; c < 0.001; d < 0.0001.

**FIGURE 2:**
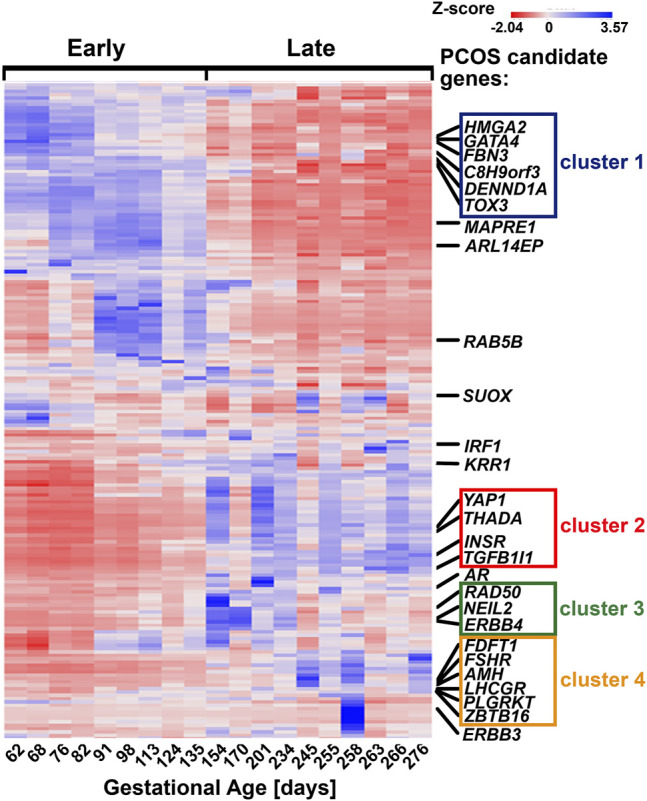
Location of PCOS candidate genes and their respective clusters on the supervised heatmap of 24,889 genes identified in the fetal ovaries (62–276 days, *n* = 19) using RNA sequencing. Each cluster is made up of PCOS candidate genes as well as genes co-expressed with them; detailed lists of each cluster can be found in Supplementary Tables S2–S5.

A supervised hierarchical clustering of all 24,889 genes showed that most PCOS candidate genes formed close clusters with each other according to the patterns earlier observed. Thus, most of the PCOS candidate genes that correlate significantly with each other in Table 3 formed clusters closely with each other on the heatmap (Figure 2). Two strong clusters and two weak clusters were formed. The strong clusters contain “early” genes (cluster 1); *C8H9orf3, TOX3, FBN3, GATA4, HMGA2,* and *DENND1A* and “late” genes (cluster 4); *FDFT1*, *LHCGR*, *AMH*, *FSHR, ZBTB16* and *PLGRKT* (Figure 2). The two weak clusters comprised of “late” genes (cluster 2), *YAP1, INSR, THADA,* and *TGFB1I1* and genes expressed throughout gestation (cluster 3), *RAD50*, *NEIL2,* and *ERBB4*. It is worth noting that although *FDFT1* does not correlate significantly with gestational age, it correlates significantly with most of the genes in cluster 4 according to the Pearson’s correlation (Table 3). Fold change and statistical significance were determined for the gene list of each of the clusters which were composed of PCOS candidate genes as well as the genes co-expressed with them (Supplementary Tables S2–S5). They were mapped to the IPA knowledge base and DAVID bioinformatics database to identify the canonical pathways, upstream regulators and networks associated with each cluster.

**FIGURE 3:**
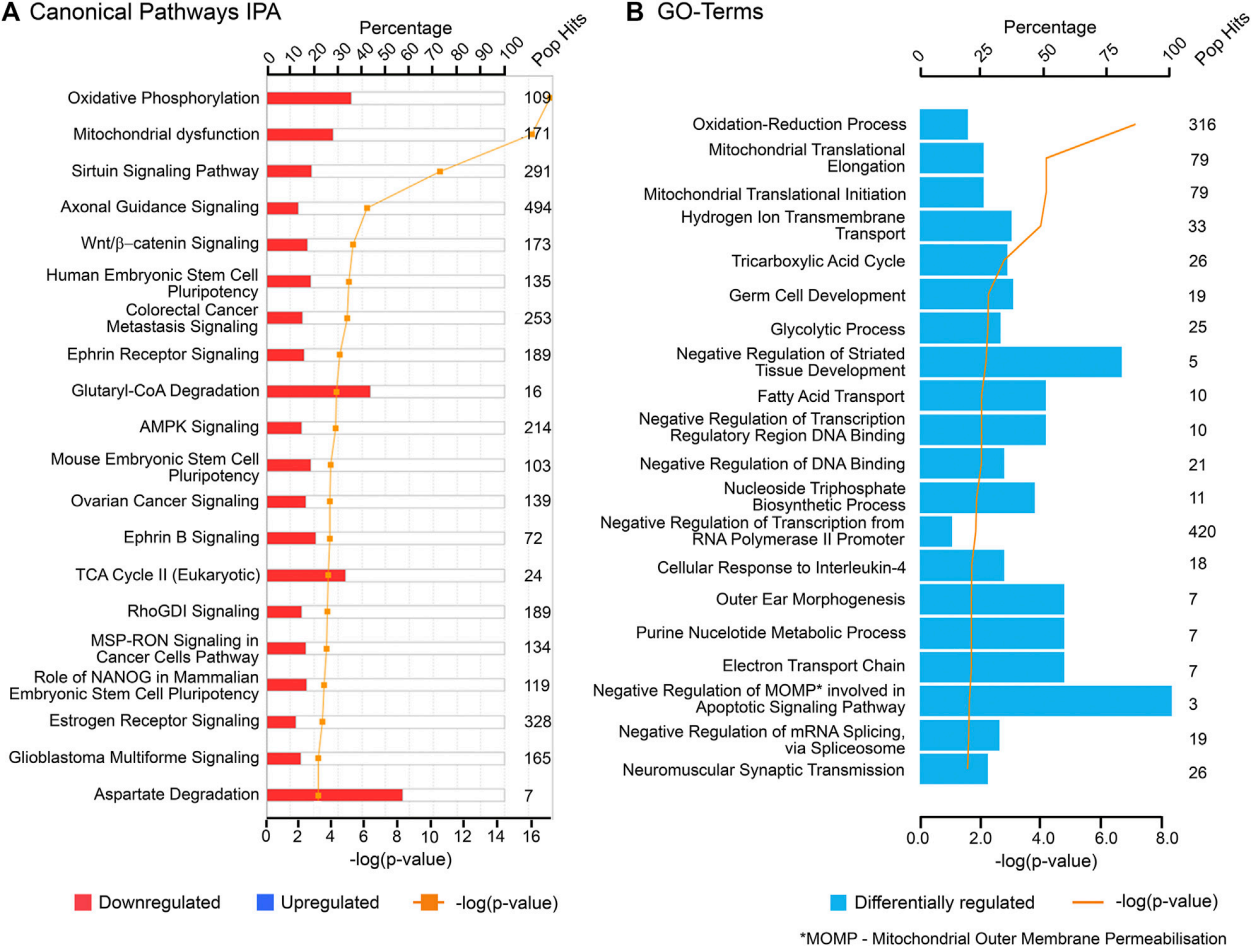
Top canonical pathways associated with cluster 1 (“early” genes) using **(A)** Ingenuity Pathway Analysis (IPA) and **(B)** Gene Ontology (GO) biological processes from DAVID database. “Pop Hits” refers to the total number of genes associated with each of the pathway in the database. The bar graphs represent the percentage of genes from the data set that map to each canonical pathway whilst the orange line shows the *p*-value of overlap between genes in each cluster and a given pathway.

**FIGURE 4:**
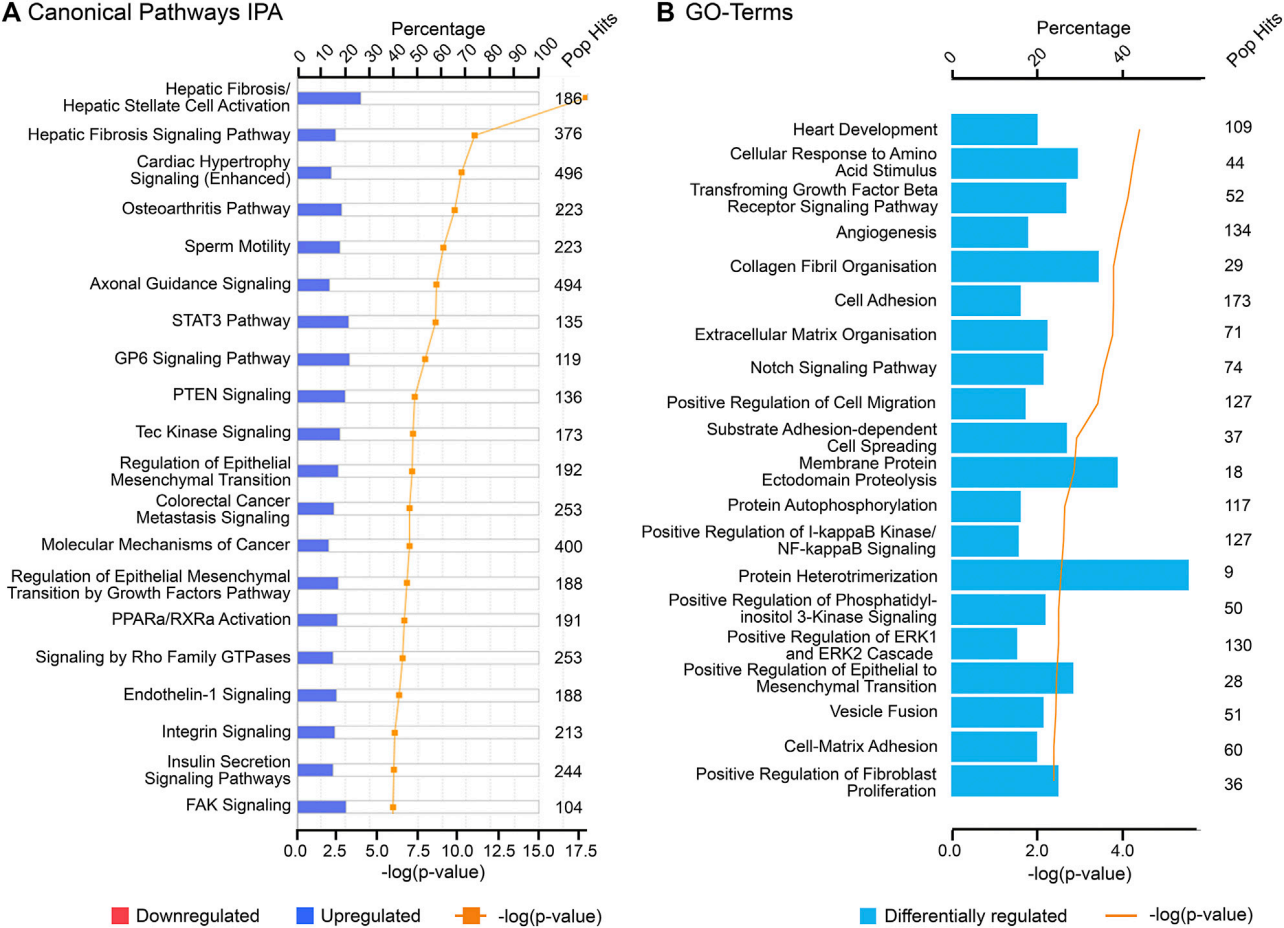
Top canonical pathways associated with cluster 2 (“late” genes) using **(A)** Ingenuity Pathway Analysis (IPA) and **(B)** Gene Ontology (GO), biological processes from DAVID database. “Pop Hits” refers to the total number of genes associated with each of the pathway in the database. The bar graphs represent the percentage of genes from the data set that map to each canonical pathway whilst the orange line shows the *p*-value of overlap between genes in each cluster and a given pathway.

**FIGURE 5:**
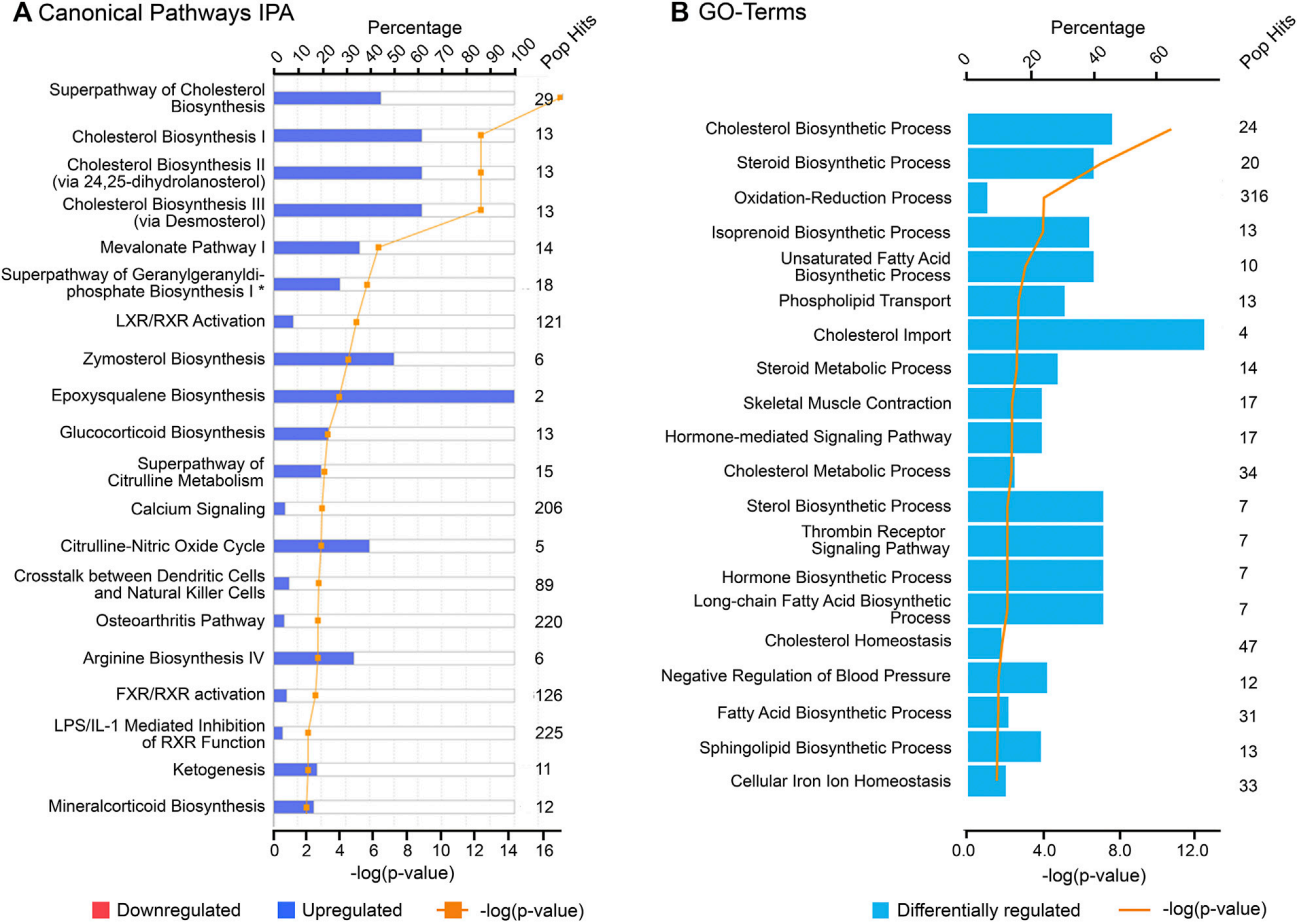
Top canonical pathways associated with cluster 4 (“late” genes) using **(A)** Ingenuity Pathway Analysis (IPA) and **(B)** Gene Ontology (GO), biological processes from DAVID database. “Pop Hits” refers to the total number of genes associated with each of the pathway in the database. The bar graphs represent the percentage of genes from the data set that map to each canonical pathway whilst the orange line shows the *p*-value of overlap between genes in each cluster and a given pathway.

**FIGURE 6:**
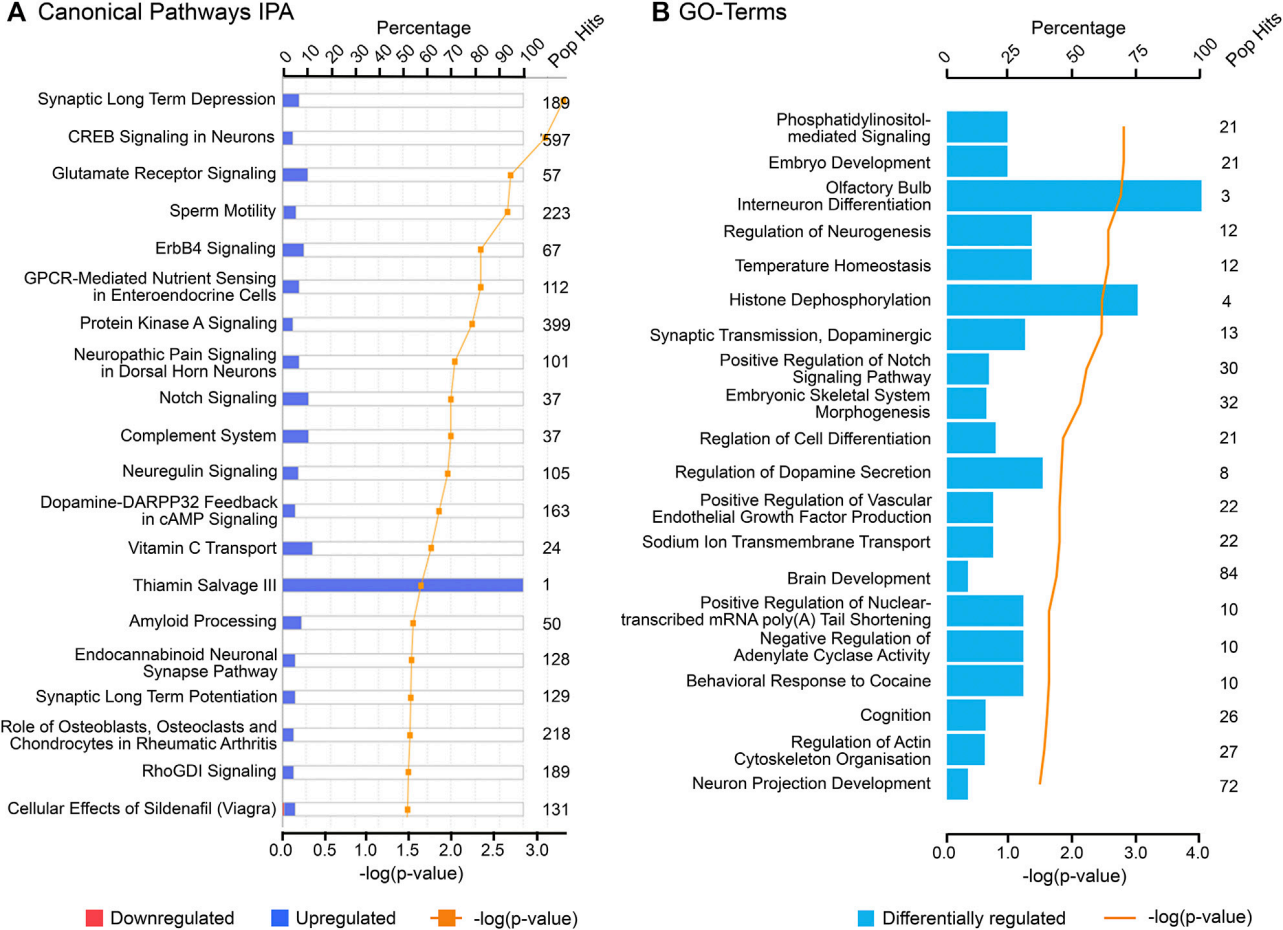
Top canonical pathways associated with cluster 3 (“throughout” genes) using **(A)** Ingenuity Pathway Analysis (IPA) and **(B)** Gene Ontology (GO), biological processes from DAVID database. “Pop Hits” refers to the total number of genes associated with each of the pathway in the database. The bar graphs represent the percentage of genes from the data set that map to each canonical pathway whilst the orange line shows the *p*-value of overlap between genes in each cluster and a given pathway.

The findings of this study were then aligned with the developmental stages of the ovary. Both IPA and GO (biological processes) analyses of each cluster revealed relevant and similar canonical pathways to be associated with genes co-expressed with PCOS candidate genes across gestation. The top canonical pathways associated with the “early” genes (cluster 1) are mainly involved in mitochondrial function (Figure 3) whilst “late” genes were involved in stromal development and expansion (cluster 2) and lipid biosynthesis/steroidogenesis (cluster 4) (Figures 4, 5). Cluster 3, which consists of genes expressed throughout gestation, seems to be associated with a range of different pathways including central nervous system regulation and signalling among others (Figure 6). These pathways were also observed to some extent in the GO and KEGG pathway analysis for each cluster; with the strongest similarities to IPA observed in clusters 1 and 4 for mitochondrial function and steroidogenesis, respectively (Supplementary Tables S6–S9). Also, the number of genes extracted from each of these clusters as well as those that map to the IPA knowledge base and DAVID bioinformatic database for each cluster are shown in Supplementary Table S10.

Top biological upstream regulators associated with the strong clusters 1 and 4 and the weak clusters 2 and 3 were identified using IPA and are shown in Tables 4, 5, respectively. Upstream regulators such as *TGFβ, TNF*, angiotensin, *ESR1*, among others were common to the “late” clusters 2 and 4. Upstream regulators such as *PTEN, HNF4A, ESRRA/G, PSEN1, MYC,* mitochondrial *LONP1,* and *TP53* among others were associated with cluster 1 whereas *HNF1A, GATA2, PSENEN, IL25, REST, OCT4-NANOG, APHIA,* and *AGRN* were associated with cluster 3. Chemical upstream regulators (including endogenous molecules) associated with each cluster was also studied. The list of the top chemical (including endogenous molecules) upstream regulators associated with each cluster are detailed in Supplementary Tables S11, S12. Estradiol is among the top chemical upstream regulators associated with certain genes in clusters 1, 2, and 4 while dexamethasone regulates some genes in cluster 2 and 4 among others.

**TABLE 4:** Top biological upstream regulators and their respective activation z-score as well as *p*-value of association for the strong clusters, cluster 1 and 4.

	Upstream regulator	Name	z-score	*p*-value
Cluster 1	*CLPP*	Caseinolytic mitochondrial matrix peptidase proteolytic subunit	4.899	1.11E-11
	*PTEN*	Phosphatase and tensin homolog	−0.143	1.46E-10
	*HNF4A*	Hepatocyte nuclear factor 4 alpha	−3.972	1.90E-10
	*TLE3*	TLE family member 3, Transcriptional corepressor	—	2.69E-10
	*DAP3*	Death associated protein 3	−3.162	1.05E-09
	*ARNT*	Aryl hydrocarbon receptor nuclear translocator	−3.689	2.98E-09
	*LONP1*	Lon peptidase 1, Mitochondrial	−1.457	3.71E-09
	*ESRRG*	Estrogen related receptor gamma	−2.006	6.71E-09
	*Firre*	Firre intergenic repeating RNA element	−4.796	1.41E-08
	*APP*	Amyloid beta precursor protein	−2.107	3.01E-08
	*MYC*	MYC proto-oncogene, BHLH transcription factor	−7.632	3.11E-08
	*STK11*	Serine/Threonine kinase 11	−4.041	3.31E-08
	*ALKBH1*	AlkB homolog 1, Histone H2A dioxygenase	−2.646	8.62E-08
	*NSUN3*	NOP2/Sun RNA methyltransferase 3	-2.646	8.62E-08
	*PSEN1*	Presenilin 1	−0.649	1.64E-07
	*KDM5A*	Lysine demethylase 5A	4.914	2.45E-07
	*MAPT*	Microtubule associated protein tau	—	3.58E-07
	*RICTOR*	RPTOR independent companion of MTOR complex 2	4.854	5.14E-07
	*DDX5*	DEAD-box helicase 5	−3.638	2.46E-06
	*TP53*	Tumor protein P53	−1.511	2.90E-06
	*Esrra*	Estrogen related receptor alpha	−1.849	3.18E-06
Cluster 4	*SREBF2*	Sterol regulatory element binding transcription factor 2	4.586	1.41E-22
	*INSIG1*	Insulin induced gene 1	−4.447	5.74E-21
	*MAP2K5*	Mitogen-activated protein kinase 5	4.088	3.78E-18
	*SCAP*	SREBF chaperone	4.269	9.03E-18
	*POR*	Cytochrome P450 oxidoreductase	−3.776	4.93E-16
	*SREBF1*	Sterol regulatory element binding transcription factor 1	4.508	3.27E-14
	*MFSD2A*	Major facilitator superfamily domain containing 2A	−3.138	8.14E-13
	*ATP7B*	ATPase copper transporting beta	3.464	1.46E-12
	*MAPK7*	Mitogen-activated protein kinase 7	3.873	2.30E-12
	*NPPB*	Natriuretic peptide B	−3.283	3.41E-12
	*SH3TC2*	SH3 domain and tetratricopeptide repeats 2	3.162	2.27E-11
	*PROM1*	Prominin 1	−2.828	3.28E-11
	*PPARA*	Peroxisome proliferator activated receptor alpha	1.112	6.45E-11
	*NR5A1*	Nuclear receptor subfamily 5 group A member 1	3.446	7.22E-11
	*C4BP*	Complement component 4 binding protein	2.449	5.69E-10
	*RPTOR*	Regulatory associated protein of MTOR complex 1	3.2	1.43E-09
	*KIF3A*	Kinesin family member 3A	−2.596	1.47E-09
	*INSIG2*	Insulin induced gene 2	−2.586	1.97E-09
	*TGFB1*	Transforming growth factor beta 1	2.172	3.90E-09
	*SIRT2*	Sirtuin 2	1.913	6.77E-09

**TABLE 5:** Top biological upstream regulators and their respective activation z-score as well as *p*-value of association for the weak clusters, cluster 2 and cluster 3.

	Upstream regulator	Name	z-score	*p*-value
Cluster 2	*TGFB1*	Transforming growth factor beta 1	7.017	1.77E-21
	*HRAS*	HRas proto-oncogene, GTPase	−0.699	1.60E-14
	*ERBB2*	Erb-B2 receptor tyrosine kinase 2	−0.667	7.95E-13
	*F2*	Coagulation factor II, Thrombin	5.485	9.38E-13
	Alpha catenin	Alpha catenin group	−5.256	1.76E-12
	*TGFB2*	Transforming growth factor beta 2	3.062	3.71E-12
	*TNF*	Tumor necrosis factor	3.597	1.00E-11
	*Mek*	Mitogen-activated protein kinase 1	3.218	1.23E-11
	*COLQ*	Collagen like tail subunit of asymmetric acetylcholinesterase	1.511	1.29E-11
	*MRTFB*	Myocardin related transcription factor B	5.14	1.60E-11
	*VEGF*	Vascular endothelial growth factor	5.581	9.26E-11
	*TP53*	Tumor protein P53	4.493	9.90E-11
	*Tgf beta*	Transforming growth factor beta	4.093	2.28E-10
	*AGT*	Angiotensinogen	3.331	3.01E-10
	*FGF2*	Fibroblast growth factor 2	4.223	4.14E-10
	*ITGB1*	Integrin subunit beta 1	−1.223	5.19E-10
	*SP1*	Sp1 transcription factor	5.601	1.01E-09
	*ERBB3*	Erb-B2 receptor tyrosine kinase 3	0.325	1.71E-09
	*TP63*	Tumor protein P63	1.448	1.80E-09
	*TGFBR2*	Transforming growth factor beta receptor 2	3.079	3.79E-09
	*TGFB3*	Transforming growth factor beta 3	3.759	4.62E-09
Cluster 3	*ASCL1*	Achaete-scute family BHLH transcription factor 1	2.543	7.44E-05
	*FOXQ1*	Forkhead box Q1	—	5.54E-04
	*Dst*	Dystonin	—	5.54E-04
	*NOBOX*	NOBOX oogenesis homeobox	—	9.83E-04
	*HNF1A*	HNF1 homeobox A	2.88	1.51E-03
	*RSPO2*	R-spondin 2	—	3.22E-03
	*TBPL1*	TATA-box binding protein like 1	—	3.22E-03
	*ATOH1*	Atonal BHLH transcription factor 1	—	3.48E-03
	*GATA2*	GATA binding protein 2	−0.128	4.78E-03
	*SNCA*	Synuclein alpha	−1.673	4.83E-03
	*GRIN3A*	Glutamate ionotropic receptor NMDA type subunit 3A	−2.828	5.61E-03
	*SAMD4A*	Sterile alpha motif domain containing 4A	—	7.81E-03
	*PSENEN*	Presenilin enhancer, gamma-secretase subunit	—	1.08E-02
	*PTPN11*	Protein tyrosine phosphatase non-receptor type 11	—	1.09E-02
	*REST*	RE1 silencing transcription factor	−2.19	1.14E-02
	*OCT4-NANOG*	POU class 5 homeobox 1-nanog homeobox	—	1.41E-02
	*IL25*	Interleukin 25	0.277	1.63E-02
	*APH1A*	Aph-1 homolog A, gamma-secretase subunit	—	1.79E-02
	*AGRN*	Agrin	—	1.79E-02

**FIGURE 7:**
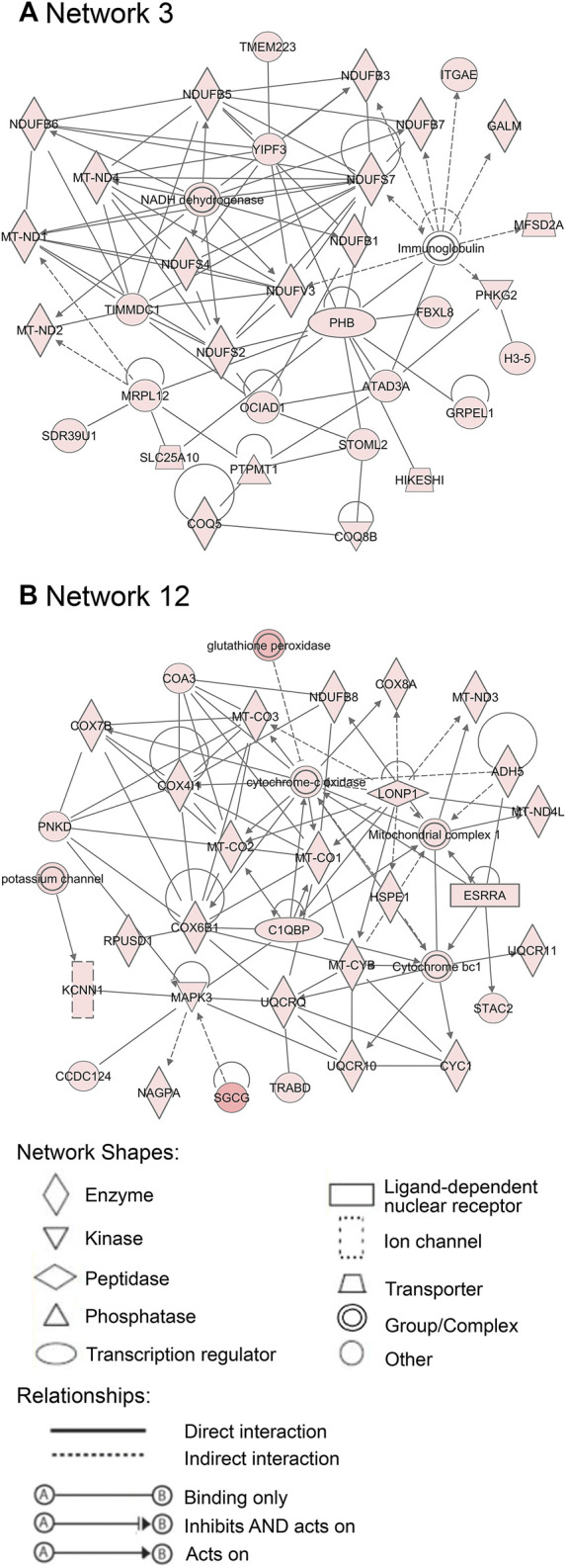
Important networks connected with cluster 1. Network 3 **(A)** and network 12 **(B)** are associated with mitochondrial functions. Red color of molecules represents downregulation in the second half of gestation and the intensity of each color shows the strength of regulation.

**FIGURE 8:**
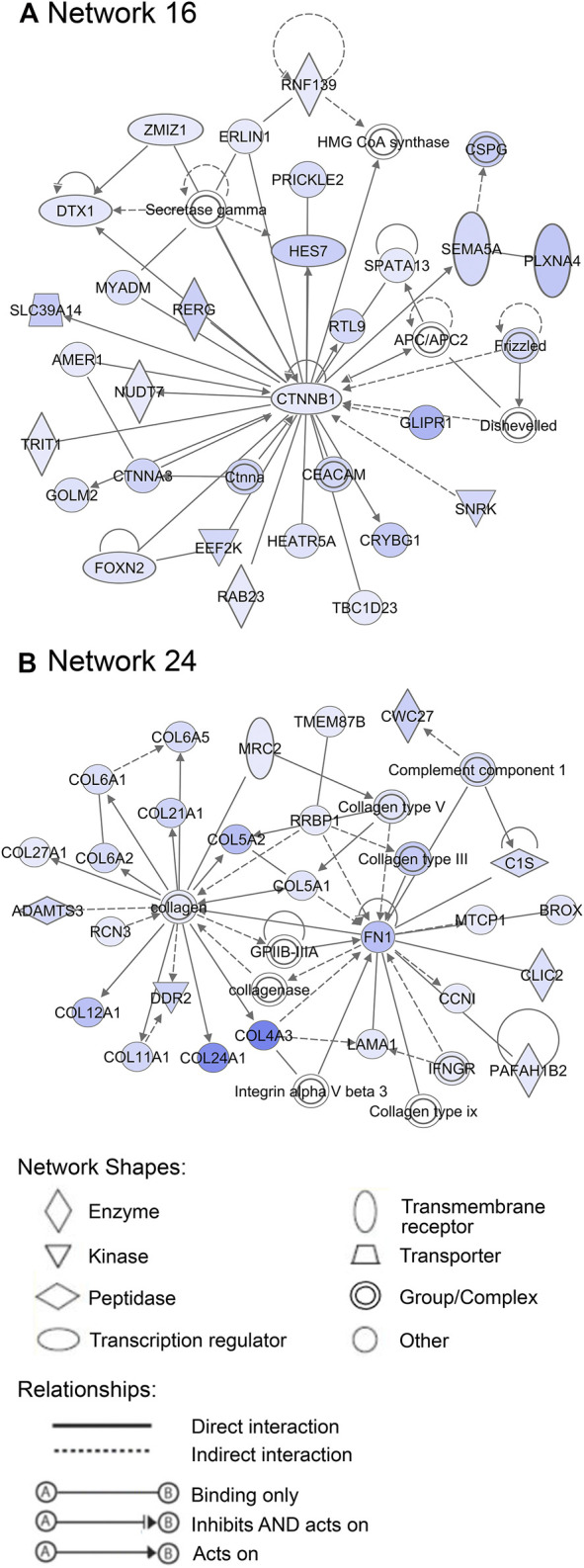
Important networks associated with cluster 2. **(A)** Network 16 contains β-catenin as central player, whereas **(B)** network 24 is associated with the components of extracellular matrix, such as collagens and fibronectin. Blue color of molecules represents upregulation in the second half of gestation and the intensity of each color shows the strength of regulation.

**FIGURE 9:**
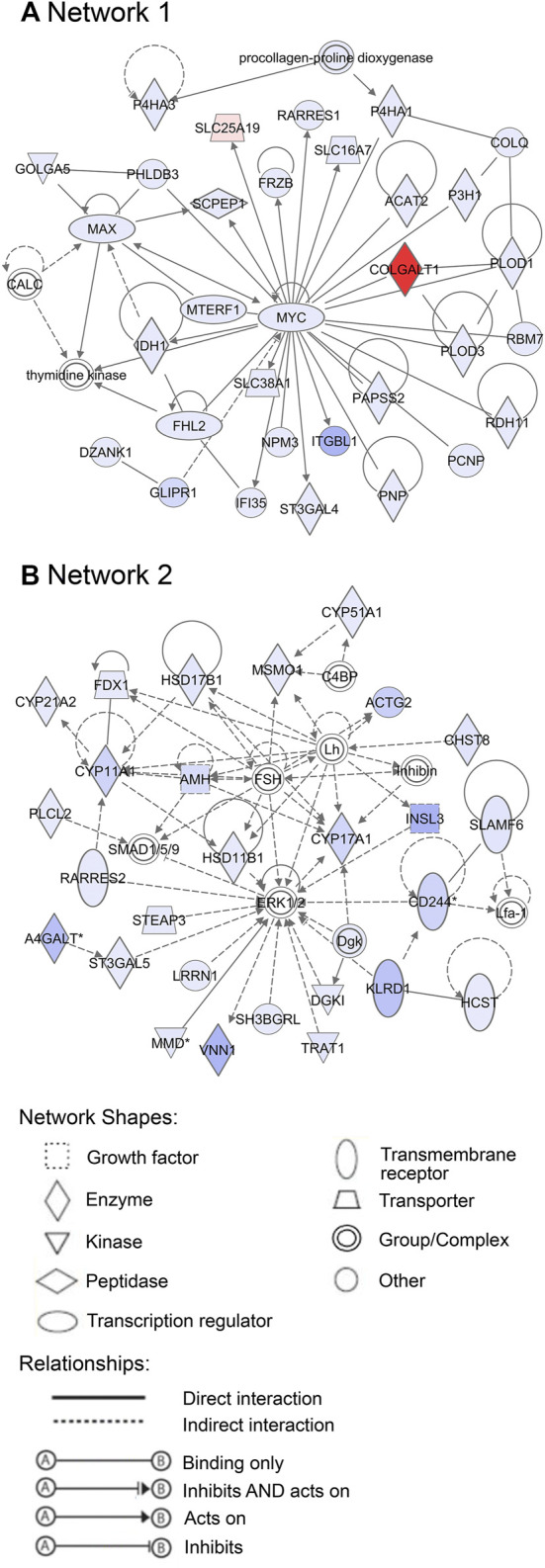
Important networks of genes associated with cluster 4. **(A)** Network 1 is associated with the effects of the transcription factor MYC. **(B)** Network 2 is connected with steroidogenesis. Red represents downregulation and blue upregulation in the second half of gestation. The strength of regulation is shown by the intensity of each color.

Additionally, 25 networks were generated by the IPA database for each cluster showing their functions and diseases. However, only those rated as important for fetal development and the role of PCOS candidate genes are shown in Figures 7–9. Specifically, mitochondrial function networks (1 and 3) of cluster 1 consisting of nuclear-encoded and mitochondrial DNA-encoded genes were downregulated during the second half of gestation (Figure 7). Networks associated with cluster 2, which were upregulated during the second half of gestation, consist of β-catenin as the central player (network 16) interacting with other subunits of its kind, namely frizzled molecules and cadherins, which are cell adhesion molecules (CAM) (Figure 8A). The second network of cluster 2 (network 24) consists of extracellular matrix components including the different types of collagen with two central molecules, collagen, and fibronectin (Figure 8B). The top networks for cluster 4, which were also upregulated during the second half of gestation, are associated with the effects of the transcription factor *MYC* in metabolism (network 1) and steroidogenesis (network 2) (Figure 9). All other relevant networks are summarized in Supplementary Figures S2–S5.

### Nuclear-Encoded Mitochondrial Genes and Mitochondrial-DNA Encoded Genes

Oxidative phosphorylation and mitochondrial dysfunction pathways are the top canonical pathways co-expressed with the PCOS candidate genes expressed during early gestation (cluster 1). mRNA expression of nuclear-encoded mitochondrial genes associated with these top pathways as well as genes encoded by mitochondrial DNA from the RNA-seq are shown as scatterplots to outline their expression patterns across gestation (Supplementary Figures S6, S7, respectively). Similar patterns of expression were observed for both; thus, these genes are highly expressed during the early stages of gestation with expression decreasing gradually until 200 days of gestation, then the expression is maintained or increased slightly at that level in the third trimester.

## Discussion

Using RNA-seq data from normal fetal ovaries collected across gestation, we examined the expression patterns of PCOS candidate genes and found that most belonged to one of four gene clusters. The expression patterns of most of the PCOS candidate genes in the RNA-seq data were consistent with those previously identified by both Hartanti, et al. (2020) and Liu, et al. (2020). In addition to the PCOS candidate genes identified as expressed “early” or “late” in gestation in these previous studies, we found that *MAPRE1*, *ARL14EP,* and *C8H9orf3* were also “early” genes, whilst *YAP1* and *THADA* were “late” genes. The study then identified canonical pathways, biological and chemical upstream regulators and networks of genes that are co-expressed in each of the clusters. This knowledge could be vital for understanding the mechanisms and identifying upstream regulators for delineating the fetal origin of PCOS.

During the early stages of fetal ovary development, primordial germ cells settle in the developing ovary as oogonia and start to proliferate between the proliferating Gonadal Ridge Epithelial-Like (GREL) cells. Then stroma of the mesonephros, containing fibroblasts, fibres, and capillaries, penetrates into the gonadal ridge. This results in the formation of the ovigerous cords containing the GREL cells and proliferating oogonia. The ovigerous cords are separated from the penetrating stroma by a basal lamina and are open at the surface of the ovary during early gestation (Hummitzsch, et al., 2013; Hummitzsch et al., 2019). Interestingly, we found in cluster 1 that mitochondrial function is a key canonical pathway at this early stage of fetal ovary development. Expression patterns of nuclear-encoded mitochondrial genes and mitochondrial DNA-encoded genes across gestation showed that mitochondrial function and oxidative phosphorylation are at peak during stromal proliferation and penetration, specifically from the time of ovigerous cord formation until the cords begin to breakdown and primordial follicle formation occurs during fetal ovary development.

The relationship between mitochondrial dysfunction and the pathogenesis of PCOS remains unclear, although studies focussed on this topic began over the last decade. Mitochondrial DNA copy number and abnormal reactive oxidative species have been associated with numerous phenotypes of PCOS (Hyderali and Mala 2015; Shukla et al., 2020). Thus, insulin resistance, obesity, hyperandrogenism among others have been linked directly/indirectly to mitochondrial dysfunction [see reviews (Ilie 2018; Zhang et al., 2019; Zeng et al., 2020)]. A meta-analysis involving lean (BMI ≤ 23) and obese (BMI ≥ 23) PCOS patients using a systematic and comparative study by Idicula-Thomas et al. (2020) showed that nuclear-encoded mitochondrial genes were downregulated mainly in the cumulus cells of obese PCOS patients, but upregulated mainly in subcutaneous adipose tissues of lean PCOS patients. Furthermore, previous studies have shown that offspring of androgenised lean PCOS mice had impaired ovarian mitochondrial ultrastructure and function when compared to their controls (Chappell et al., 2020). Thus, mitochondrial ultrastructure in oocytes of androgenised offspring had disorganised cristae and swollen vacuoles without any electron dense content; a lower inner mitochondrial membrane potential of oocytes was also observed as compromised mitochondria function in the PCOS lean mice (Chappell, et al., 2020). This implies that *in utero* exposures to high androgen levels can significantly affect the development of the ovaries, most probably at the early stages of gestation, when germ cells undergo mitosis, ovigerous cords form, and stroma proliferates and migrates leading to the fetal programming of PCOS. More so, aberrant mitochondrial function was observed in oocytes at germinal vesicles stage of PCOS patients when compared to healthy controls in a study that compared the various stages of oocyte development in the two groups using single cell RNA sequencing (Qi et al., 2020). Additionally, studies focussed on the early stages of fetal ovary development in pig have also shown upregulation of mitochondrial activity and oxidative phosphorylation genes, inferred by higher expression of mitochondrial DNA-encoded genes at early stages of development (E31). During this stage, extensive proliferation of primordial germ cells and the global waves of methylation and demethylation occur in the porcine ovary; requiring significant levels of energy to sustain the changes (Zhu et al., 2021). The high energy produced results in by-products (e.g., alpha-ketoglutarate) that are then used to establish various epigenetic marks and modulate demethylation by oxidising of 5-methylcytosine (5mC) to 5-hydroxymethylcytosine (5hmC) (Tahiliani et al., 2009; Wu and Zhang 2017). The findings together infer that mitochondrial function could play a significant role in the predisposition of PCOS; requiring further studies.

Furthermore, the identification of upstream regulators such as *DAP3,* which plays significant roles in mitochondrial respiration and apoptosis (Tang et al., 2009), and *MYC,* which is a regulator of mitochondrial biogenesis (Li et al., 2005), as well as the downregulation of nuclear and mitochondrial DNA-encoded genes during the second half of gestation, in this study, implies that mitochondrial function is vital to the early stages of ovary development.

During mid-gestation, compartmentalisation of the ovary into the cortex and medulla becomes apparent, where there are alternating ovigerous cords and stromal areas in the cortex and stroma from the mesonephros containing extracellular matrix, fibroblasts and vasculature in the medulla. The stroma starts to migrate laterally after reaching just below the ovarian surface resulting in the closing of the ovigerous cords on the surface and the establishment of an ovarian surface epithelium underlain by a basal lamina (Hummitzsch, et al., 2019; Hummitzsch, et al., 2013). This is, to some extent, consistent with our observations of genes connected with cluster 2, which includes genes involved in stromal expansion. Notably, the presence of elevated levels of fibrous tissues and collagen in the ovarian capsule or tunica albuginea resulting in denser ovarian stroma are common features of polycystic ovaries. Increased stromal collagen and ovarian cortex expansion in PCOS patients are associated with dysfunction of fibrillin 3 (*FBN3*) which is highly expressed in early stages of fetal development and not expressed late in gestation or adult ovaries (Hatzirodos, et al., 2011). *FBN3* regulates transforming growth factor-β (TGF-β) pathways, which stimulate fibroblast proliferation and collagen formation. TGF-β may also play significant roles in the cardiovascular and metabolic symptoms of PCOS as discussed in review by Raja-Khan et al. (2014). Upstream regulators involved in TGF-β signalling pathways such as *TGFB1, TGFB2, TGFB3,* and *TGFBR2* and fibroblast proliferation regulators such as *FGF2*, coagulation factor II (*F2*) were consistent with findings of this study. In addition, networks associated with the components of extracellular matrix, such as collagen and fibronectin which are relevant to this stage of ovary development were associated with this cluster of genes.

Furthermore, *YAP1*, a PCOS candidate gene in cluster 2, is a critical regulator of granulosa cell proliferation, differentiation and survival by interaction with epidermal growth factor receptor, gonadotrophin, and TGF-β signalling pathways (Lv et al., 2019). More so, *ERBB3,* which is an early gene according to our GSA analysis and Liu et al. (2020) and a member of the epidermal growth factor family, was identified as an upstream regulator for genes in cluster 2. Additionally, β-catenin, which is a central molecule for one of the networks associated with this cluster, plays an essential role in Wnt signal transduction and in intercellular adhesion by interacting with cadherin (Liu et al., 2009; Bush et al., 2019). Undoubtedly, the regulation of the PCOS candidate genes during this stage plays a substantial role in the cascade of events that occur later in fetal development.

During the late stages of ovary development, follicles consisting of oocytes and granulosa cells are formed from the differentiation of ovigerous cords (Hummitzsch, et al., 2013; Heeren et al., 2015). The first primordial follicles appear in the inner cortex-medulla region, surrounded by a basal lamina. The surface epithelium, mostly single-layered, then surrounds the ovary. Stroma beneath the surface epithelial basal lamina then develops into the tunica albuginea during the final developmental stages of the human and bovine ovary (Hummitzsch, et al., 2013; Heeren, et al., 2015; Hummitzsch, et al., 2019). Activation of some primordial follicles occurs leading to their development into primary and preantral follicles. Remarkably, the genes in cluster 4 (including the PCOS candidate genes), top canonical pathways, upstream regulators and networks were involved in folliculogenesis and ovarian steroidogenesis which is consistent with occurrence during this stage of development. *FDFT1,* which is an essential enzyme in the synthesis of sterols further leading to the synthesis of cholesterol, correlates significantly with genes involved in folliculogenesis such as *AMH, FSHR, LHCGR*. More so, upstream regulators involved in steroid synthesis such as *SREBF2, INSIG1, TGFB1, RPTOR*, and networks such as network 2 which has a central molecule, Mitogen-Activated Protein Kinases, *ERK1/2*, interacting with other molecules of steroidogenesis such as FSH, LH, AMH, *CYP11A1* were also identified for this cluster.

Despite the increasing number of genetics studies to define the abnormalities associated with PCOS, the pathogenesis of the syndrome still remains a challenge. Numerous studies to delineate the transgenerational susceptibility of PCOS in animals and human are on-going (Tata, et al., 2018; Risal et al., 2019; Mimouni et al., 2021) and this study provides upstream regulators as well as canonical pathways that could be further studied to delineate the pathogenesis of the syndrome. Notably, genes co-expressed with most PCOS candidate genes during both the early and late developmental stages of fetal development are involved in pathways that have previously been associated with PCOS, although mechanisms remain elusive. It would be very logical to infer that perturbations associated with mitochondrial function in the early stages of fetal ovary development lead to a cascade of events across gestation, some of which impact key canonical pathways such as stromal expansion and steroidogenesis, possibly, leading to PCOS in adulthood. The outcome of this study supports the need to further study the fetal origin of PCOS with the hope to, not only define the syndrome, but also towards the diagnosis, treatment and prevention of syndrome.

In summary, although this study is an *in-silico* analysis of PCOS candidate genes and their co-expressed genes from RNA-seq data, it has shown significant consistency with the literature on ovarian development and PCOS. Additional studies to delineate the pathogenesis of the syndrome will be required based on these findings. The limitations of IPA software are also acknowledged as IPA is a knowledge based curated software.

## Conclusion

These findings highlight the involvement of PCOS-associated genes in mitochondrial function, stromal expansion and steroidogenesis during ovarian development. It could be speculated that perturbations during fetal ovary development resulting from dysregulation of these pathways could result in the different phenotypes of PCOS observed during adulthood. These perturbations could be genetic and/or environmental depending on maternal environment and fetal exposures associated with it. Further studies to delineate the role(s) of these pathways in the pathogenesis of PCOS are necessary. Notably, these findings infer a relationship between these major pathways, usually studied separately to define the syndrome and the need for studies investigating their interactions.

## Data Availability

The datasets presented in this study can be found in online repositories. The names of the repository/repositories and accession number(s) can be found below: https://www.ncbi.nlm.nih.gov/, GSE178450.
