# Supplementary material for: Analysis of Upstream Regulators, Networks, and Pathways Associated With the Expression Patterns of Polycystic Ovary Syndrome Candidate Genes During Fetal Ovary Development
Source: Front Genet. 2022 Feb 7;12:762177. doi: 10.3389/fgene.2021.762177 (PMC8860493; doi:10.3389/fgene.2021.762177)
Supplement: Supplementary file 1 [file DataSheet2.PDF]

S1 Table. Gene Specific Analysis (late versus early gestation) of PCOS candidate genes. Shown are the fold change and its significance (p-value), log 2 ratio and False Discovery Rate (FDR) across gestation; least square (LS) mean (late and early gestation); total counts per million.

Gene symbol	Total counts	P-value	FDR step up	Ratio	Log2(Ratio)	Fold change	LSMean(Late)	LSMean(Early)
gene-INSR	1942.25	9.05E-10	2.44E-08	3.58	1.84	3.58	155.25	43.31
gene-AR	115.50	1.48E-07	2.00E-06	20.22	4.34	20.22	11.06	0.55
gene-MAPRE1	1480.03	4.16E-07	3.01E-06	0.61	-0.72	-1.65	59.56	98.27
gene-TGFB1I1	1593.22	4.46E-07	3.01E-06	2.09	1.06	2.09	111.35	53.30
gene-THADA	469.07	9.42E-07	4.33E-06	1.58	0.66	1.58	29.87	18.93
gene-GATA4	7348.90	1.07E-06	4.33E-06	0.54	-0.89	-1.86	275.16	510.81
gene-FBN3	6416.64	1.12E-06	4.33E-06	0.24	-2.03	-4.10	136.86	560.89
gene-YAP1	2543.69	5.58E-06	1.88E-05	1.64	0.71	1.64	164.20	100.19
gene-FSHR	117.47	3.24E-05	8.81E-05	5.23	2.39	5.23	10.02	1.92
gene-ARL14EP	732.89	3.26E-05	8.81E-05	0.63	-0.68	-1.60	30.06	48.03
gene-HMGA2	68.48	4.12E-05	1.01E-04	0.29	-1.79	-3.46	1.66	5.76
gene-ZBTB16	728.91	4.83E-05	1.09E-04	2.63	1.40	2.63	54.32	20.63
gene-TOX3	231.36	6.51E-05	1.35E-04	0.28	-1.84	-3.58	5.47	19.62
gene-C8H9orf3	1205.21	1.20E-03	2.30E-03	0.71	-0.50	-1.41	53.04	74.98
gene-AMH	233.84	1.37E-03	2.47E-03	5.42	2.44	5.42	20.05	3.70
gene-IRF1	662.25	2.54E-03	4.28E-03	1.52	0.60	1.52	41.59	27.38
gene-ERBB3	346.91	1.60E-02	2.53E-02	0.68	-0.56	-1.47	14.91	21.98
gene-DENND1A	1915.27	2.10E-02	3.08E-02	0.82	-0.29	-1.22	91.26	111.40
gene-ERBB4	24.70	2.17E-02	3.08E-02	3.93	1.97	3.93	2.01	0.51
gene-PLGRKT	146.53	2.70E-02	3.65E-02	1.26	0.34	1.26	8.56	6.77
gene-LHCGR	131.88	9.50E-02	1.22E-01	6.39	2.68	6.39	11.56	1.81
gene-RAD50	2093.91	1.72E-01	2.12E-01	1.35	0.43	1.35	125.48	93.23
gene-NEIL2	579.54	2.33E-01	2.73E-01	0.91	-0.14	-1.10	29.11	32.05
gene-SUOX	695.27	2.97E-01	3.35E-01	0.93	-0.10	-1.08	35.33	37.99
gene-FDFT1	1618.89	3.11E-01	3.35E-01	1.15	0.20	1.15	90.68	79.12
gene-RAB5B	4927.55	5.85E-01	6.07E-01	0.96	-0.06	-1.04	254.55	264.68
gene-KRR1	509.82	8.98E-01	8.98E-01	1.00	-0.01	-1.00	26.78	26.89

S2 Table . Gene Specific Analysis (late versus early gestation) of genes in cluster 1. Shown are the fold change and its significance (p-value), log 2 ratio and False Discovery Rate (FDR) across gestation; least square (LS) mean (late and early gestation); total counts per million.

Gene symbol	Total counts	P-value	FDR step up	Ratio	Log2(Ratio)	Fold change	LSMean(Late)	LSMean(Early)
ABCG4	1.63E+01	5.69E-05	1.05E-04	4.14E-01	-1.27E+00	-2.42E+00	5.15E-01	1.24E+00
ABHD12	1.64E+03	4.79E-06	1.29E-05	7.83E-01	-3.53E-01	-1.28E+00	7.67E+01	9.79E+01
ABHD17A	1.70E+03	1.41E-06	4.74E-06	7.48E-01	-4.19E-01	-1.34E+00	7.74E+01	1.04E+02
ABHD6	3.11E+02	2.87E-06	8.43E-06	5.16E-01	-9.56E-01	-1.94E+00	1.15E+01	2.24E+01
ABHD8	6.50E+02	1.03E-08	1.19E-07	6.97E-01	-5.22E-01	-1.44E+00	2.85E+01	4.10E+01
ABR	4.68E+03	2.30E-07	1.22E-06	6.30E-01	-6.66E-01	-1.59E+00	1.93E+02	3.07E+02
ABRACL	7.02E+01	1.60E-02	1.68E-02	7.01E-01	-5.12E-01	-1.43E+00	3.11E+00	4.44E+00
ACAA2	6.54E+02	7.30E-04	9.67E-04	7.73E-01	-3.71E-01	-1.29E+00	3.01E+01	3.90E+01
ACIN1	9.59E+03	2.86E-04	4.22E-04	8.36E-01	-2.59E-01	-1.20E+00	4.65E+02	5.56E+02
ACOT2-2	1.08E+01	5.31E-03	5.94E-03	1.50E-01	-2.74E+00	-6.69E+00	1.53E-01	1.02E+00
ACTG1	4.62E+04	1.22E-05	2.80E-05	7.68E-01	-3.81E-01	-1.30E+00	2.14E+03	2.79E+03
ACY1	2.40E+02	4.81E-03	5.42E-03	7.82E-01	-3.55E-01	-1.28E+00	1.13E+01	1.45E+01
ADAM11	1.54E+02	1.30E-06	4.47E-06	3.64E-01	-1.46E+00	-2.75E+00	4.47E+00	1.23E+01
ADAM8	5.58E+02	1.69E-06	5.46E-06	4.38E-01	-1.19E+00	-2.28E+00	1.85E+01	4.22E+01
ADAMTS13	2.17E+02	2.85E-05	5.72E-05	3.25E-01	-1.62E+00	-3.08E+00	5.83E+00	1.80E+01
ADCK1	2.16E+02	2.53E-05	5.17E-05	5.96E-01	-7.48E-01	-1.68E+00	8.71E+00	1.46E+01
ADD2	1.97E+02	3.90E-06	1.09E-05	4.40E-01	-1.18E+00	-2.27E+00	6.66E+00	1.51E+01
ADGRA1	9.67E+01	7.31E-03	7.99E-03	5.99E-01	-7.39E-01	-1.67E+00	3.89E+00	6.50E+00
ADGRB2	1.23E+03	7.72E-05	1.37E-04	6.46E-01	-6.31E-01	-1.55E+00	5.18E+01	8.02E+01
ADGRG1	2.40E+03	8.79E-07	3.31E-06	4.02E-01	-1.31E+00	-2.49E+00	7.56E+01	1.88E+02
ADH5	1.75E+03	2.00E-07	1.10E-06	6.66E-01	-5.87E-01	-1.50E+00	7.50E+01	1.13E+02
ADH6-2	2.28E+01	6.29E-07	2.58E-06	1.92E-01	-2.38E+00	-5.22E+00	4.16E-01	2.17E+00
ADK	3.78E+02	2.53E-03	3.02E-03	6.92E-01	-5.32E-01	-1.45E+00	1.64E+01	2.37E+01
ADRA2B	9.25E+01	4.11E-06	1.14E-05	5.23E-01	-9.34E-01	-1.91E+00	3.49E+00	6.66E+00
ADSSL1	3.78E+02	2.08E-08	2.04E-07	5.33E-01	-9.09E-01	-1.88E+00	1.42E+01	2.67E+01
AGA	7.43E+02	2.01E-03	2.43E-03	8.41E-01	-2.49E-01	-1.19E+00	3.58E+01	4.26E+01
AGAP2	1.48E+03	1.28E-06	4.45E-06	4.03E-01	-1.31E+00	-2.48E+00	4.63E+01	1.15E+02
AGAP3	1.85E+03	2.09E-07	1.14E-06	7.63E-01	-3.91E-01	-1.31E+00	8.52E+01	1.12E+02
AGER	2.82E+02	1.34E-03	1.68E-03	7.31E-01	-4.52E-01	-1.37E+00	1.27E+01	1.74E+01

AGPAT1	2.74E+03	1.09E-07	7.23E-07	7.35E-01	-4.45E-01	-1.36E+00	1.24E+02	1.68E+02
AGPAT4	2.72E+02	2.29E-07	1.22E-06	4.44E-01	-1.17E+00	-2.25E+00	9.26E+00	2.08E+01
AHCY	4.86E+03	8.15E-07	3.12E-06	5.98E-01	-7.42E-01	-1.67E+00	1.96E+02	3.27E+02
AHDC1	3.32E+03	4.32E-06	1.18E-05	5.39E-01	-8.92E-01	-1.86E+00	1.26E+02	2.33E+02
AIF1L	4.11E+02	7.71E-03	8.39E-03	5.90E-01	-7.62E-01	-1.70E+00	1.68E+01	2.85E+01
AIFM3	3.45E+03	5.58E-05	1.03E-04	3.36E-01	-1.57E+00	-2.98E+00	9.45E+01	2.81E+02
AIMP2	4.88E+02	2.41E-07	1.26E-06	6.28E-01	-6.72E-01	-1.59E+00	2.02E+01	3.22E+01
AJAP1	7.91E+01	1.24E-11	1.68E-09	3.55E-02	-4.82E+00	-2.82E+01	3.16E-01	8.92E+00
AK1	4.19E+02	1.12E-02	1.20E-02	7.40E-01	-4.33E-01	-1.35E+00	1.90E+01	2.57E+01
AK2	1.69E+03	1.06E-05	2.48E-05	7.56E-01	-4.04E-01	-1.32E+00	7.75E+01	1.03E+02
AKR1B1	2.74E+03	4.15E-05	7.99E-05	5.82E-01	-7.81E-01	-1.72E+00	1.10E+02	1.90E+02
AKT1	4.68E+03	2.30E-07	1.22E-06	7.36E-01	-4.42E-01	-1.36E+00	2.13E+02	2.89E+02
ALAS1	8.56E+02	8.34E-09	1.02E-07	6.28E-01	-6.70E-01	-1.59E+00	3.55E+01	5.64E+01
ALAS2	9.37E+01	4.86E-03	5.47E-03	3.92E-01	-1.35E+00	-2.55E+00	2.86E+00	7.31E+00
ALDH16A1	1.03E+03	1.76E-04	2.79E-04	7.93E-01	-3.35E-01	-1.26E+00	4.81E+01	6.07E+01
ALDH1A2	2.88E+03	3.10E-04	4.53E-04	4.71E-01	-1.09E+00	-2.12E+00	1.00E+02	2.13E+02
ALDH1L1	1.06E+02	1.75E-04	2.79E-04	4.61E-01	-1.12E+00	-2.17E+00	3.67E+00	7.96E+00
ALDH2	3.87E+03	9.53E-09	1.13E-07	6.48E-01	-6.27E-01	-1.54E+00	1.64E+02	2.54E+02
ALDH4A1	1.22E+03	5.87E-09	8.26E-08	5.85E-01	-7.74E-01	-1.71E+00	4.85E+01	8.30E+01
ALDOA	7.91E+03	2.22E-08	2.11E-07	5.03E-01	-9.90E-01	-1.99E+00	2.90E+02	5.76E+02
ALDOC	8.95E+02	1.79E-06	5.72E-06	6.30E-01	-6.68E-01	-1.59E+00	3.72E+01	5.90E+01
ALG14	4.30E+02	2.29E-03	2.74E-03	7.77E-01	-3.64E-01	-1.29E+00	2.00E+01	2.58E+01
ALG3	3.30E+02	3.58E-06	1.01E-05	6.69E-01	-5.80E-01	-1.49E+00	1.42E+01	2.12E+01
ALG5	2.33E+02	7.94E-07	3.08E-06	6.62E-01	-5.96E-01	-1.51E+00	9.93E+00	1.50E+01
ALKBH6	9.58E+01	8.92E-04	1.16E-03	7.54E-01	-4.07E-01	-1.33E+00	4.36E+00	5.78E+00
ALPL	2.90E+03	3.41E-08	2.97E-07	3.97E-01	-1.33E+00	-2.52E+00	9.02E+01	2.27E+02
ALX4	3.98E+01	2.04E-11	2.20E-09	2.53E-02	-5.30E+00	-3.95E+01	1.12E-01	4.44E+00
AMPD2	1.60E+03	1.68E-07	9.65E-07	6.71E-01	-5.75E-01	-1.49E+00	6.89E+01	1.03E+02
ANKRD2	4.18E+01	3.56E-06	1.01E-05	4.29E-01	-1.22E+00	-2.33E+00	1.37E+00	3.20E+00
ANKRD33	8.55E+01	9.31E-04	1.20E-03	6.34E-01	-6.58E-01	-1.58E+00	3.57E+00	5.64E+00
ANKRD55	2.62E+01	7.43E-06	1.86E-05	2.13E-01	-2.23E+00	-4.70E+00	5.26E-01	2.47E+00
ANP32A	2.62E+03	2.78E-10	1.22E-08	5.91E-01	-7.59E-01	-1.69E+00	1.05E+02	1.78E+02
ANP32B	6.01E+03	2.50E-06	7.48E-06	7.33E-01	-4.48E-01	-1.36E+00	2.71E+02	3.70E+02

ANXA6	6.51E+03	1.91E-05	4.07E-05	6.88E-01	-5.40E-01	-1.45E+00	2.85E+02	4.15E+02
AP1G2	8.53E+02	4.89E-05	9.23E-05	7.63E-01	-3.91E-01	-1.31E+00	3.94E+01	5.17E+01
AP1S3	7.08E+01	2.30E-04	3.50E-04	5.48E-01	-8.67E-01	-1.82E+00	2.65E+00	4.83E+00
AP2M1	5.54E+03	3.04E-04	4.46E-04	8.60E-01	-2.17E-01	-1.16E+00	2.72E+02	3.16E+02
AP4M1	6.97E+02	1.89E-07	1.06E-06	6.84E-01	-5.48E-01	-1.46E+00	3.04E+01	4.44E+01
APBB1	1.83E+03	7.32E-09	9.45E-08	6.44E-01	-6.36E-01	-1.55E+00	7.73E+01	1.20E+02
APCDD1	5.25E+03	1.67E-08	1.71E-07	3.30E-01	-1.60E+00	-3.03E+00	1.44E+02	4.37E+02
APEX1	2.49E+03	7.45E-09	9.50E-08	5.74E-01	-8.00E-01	-1.74E+00	9.85E+01	1.71E+02
APEX2	4.23E+02	1.34E-04	2.19E-04	7.54E-01	-4.08E-01	-1.33E+00	1.94E+01	2.58E+01
APH1A	1.51E+03	1.03E-06	3.74E-06	7.32E-01	-4.51E-01	-1.37E+00	6.80E+01	9.29E+01
APOA2	1.62E+00	1.65E-02	1.74E-02	3.62E-01	-1.46E+00	-2.76E+00	4.85E-02	1.34E-01
APOBEC3H	2.84E+02	9.90E-07	3.62E-06	5.60E-01	-8.37E-01	-1.79E+00	1.10E+01	1.97E+01
APOBEC3Z2	1.49E+02	7.27E-08	5.26E-07	4.53E-01	-1.14E+00	-2.21E+00	5.07E+00	1.12E+01
APOE	5.92E+03	6.69E-05	1.21E-04	4.92E-01	-1.02E+00	-2.03E+00	2.14E+02	4.36E+02
APOM	1.10E+02	4.87E-07	2.15E-06	3.39E-01	-1.56E+00	-2.95E+00	3.06E+00	9.02E+00
APOOL	2.35E+02	1.76E-04	2.79E-04	7.39E-01	-4.36E-01	-1.35E+00	1.07E+01	1.45E+01
AQP1	1.53E+03	1.55E-07	9.22E-07	5.11E-01	-9.67E-01	-1.96E+00	5.67E+01	1.11E+02
AQP5	1.45E+01	1.63E-03	2.00E-03	3.81E-01	-1.39E+00	-2.62E+00	4.21E-01	1.10E+00
ARC	1.83E+02	1.15E-09	3.00E-08	1.15E-01	-3.12E+00	-8.72E+00	2.15E+00	1.87E+01
ARF1	6.52E+03	1.91E-05	4.07E-05	8.38E-01	-2.55E-01	-1.19E+00	3.15E+02	3.76E+02
ARFGAP1	1.12E+03	9.29E-06	2.22E-05	8.42E-01	-2.48E-01	-1.19E+00	5.41E+01	6.43E+01
ARG2	1.27E+02	6.47E-11	5.11E-09	1.92E-01	-2.38E+00	-5.22E+00	2.28E+00	1.19E+01
ARHGAP45	8.65E+02	5.17E-09	7.66E-08	3.61E-01	-1.47E+00	-2.77E+00	2.51E+01	6.97E+01
ARHGAP9	6.02E+01	6.76E-04	9.06E-04	5.71E-01	-8.07E-01	-1.75E+00	2.34E+00	4.10E+00
ARHGDIA	5.13E+03	6.69E-05	1.21E-04	8.37E-01	-2.57E-01	-1.19E+00	2.48E+02	2.97E+02
ARHGEF18	1.71E+03	4.44E-04	6.24E-04	8.08E-01	-3.08E-01	-1.24E+00	8.08E+01	1.00E+02
ARL2	7.60E+02	3.98E-08	3.31E-07	6.09E-01	-7.17E-01	-1.64E+00	3.10E+01	5.09E+01
ARL2BP	8.86E+02	1.07E-03	1.37E-03	8.49E-01	-2.37E-01	-1.18E+00	4.30E+01	5.06E+01
ARL3	9.15E+02	7.75E-09	9.69E-08	6.00E-01	-7.38E-01	-1.67E+00	3.68E+01	6.14E+01
ARL6IP4	6.69E+02	6.23E-07	2.56E-06	7.39E-01	-4.37E-01	-1.35E+00	3.04E+01	4.12E+01
ARL8A	5.99E+02	7.40E-07	2.93E-06	7.79E-01	-3.60E-01	-1.28E+00	2.79E+01	3.58E+01
ARL9	8.72E+01	2.35E-07	1.23E-06	3.87E-01	-1.37E+00	-2.59E+00	2.64E+00	6.84E+00
ARMH4	2.98E+03	4.46E-04	6.26E-04	6.63E-01	-5.94E-01	-1.51E+00	1.27E+02	1.92E+02

ARPC3	1.27E+03	1.78E-04	2.82E-04	7.51E-01	-4.13E-01	-1.33E+00	5.81E+01	7.73E+01
ARPC4	1.27E+03	1.54E-06	5.10E-06	7.30E-01	-4.54E-01	-1.37E+00	5.70E+01	7.81E+01
ARPC5L	7.05E+02	3.85E-08	3.25E-07	5.33E-01	-9.08E-01	-1.88E+00	2.65E+01	4.97E+01
AS3MT	2.96E+02	4.18E-03	4.76E-03	6.98E-01	-5.20E-01	-1.43E+00	1.29E+01	1.84E+01
ASAP3	5.54E+02	1.16E-04	1.94E-04	6.12E-01	-7.08E-01	-1.63E+00	2.26E+01	3.70E+01
ASB16	2.35E+02	4.09E-09	6.70E-08	3.89E-01	-1.36E+00	-2.57E+00	7.18E+00	1.85E+01
ASCC1	4.20E+02	5.84E-06	1.53E-05	5.10E-01	-9.70E-01	-1.96E+00	1.53E+01	3.01E+01
ASCL4	1.20E+02	7.19E-05	1.28E-04	6.78E-01	-5.60E-01	-1.47E+00	5.19E+00	7.66E+00
ASIC4	3.04E+02	2.35E-06	7.09E-06	3.56E-01	-1.49E+00	-2.81E+00	8.72E+00	2.45E+01
ASMTL	1.25E+03	6.77E-06	1.72E-05	6.69E-01	-5.79E-01	-1.49E+00	5.37E+01	8.02E+01
ASNA1	1.22E+03	8.84E-07	3.31E-06	7.27E-01	-4.60E-01	-1.38E+00	5.49E+01	7.55E+01
ATAD3A	7.51E+02	1.10E-05	2.56E-05	5.80E-01	-7.85E-01	-1.72E+00	2.97E+01	5.12E+01
ATG10	1.16E+02	1.13E-04	1.89E-04	6.23E-01	-6.83E-01	-1.61E+00	4.78E+00	7.68E+00
ATOX1	3.91E+02	1.25E-03	1.58E-03	6.90E-01	-5.35E-01	-1.45E+00	1.71E+01	2.48E+01
ATP13A5	1.65E+00	4.99E-07	2.18E-06	4.73E-02	-4.40E+00	-2.11E+01	8.61E-03	1.82E-01
ATP1A3	3.78E+01	1.11E-04	1.86E-04	3.06E-01	-1.71E+00	-3.27E+00	9.67E-01	3.16E+00
ATP2A3	8.25E+02	7.80E-13	3.74E-10	2.06E-01	-2.28E+00	-4.84E+00	1.60E+01	7.73E+01
ATP5F1A	6.93E+03	2.73E-07	1.37E-06	6.76E-01	-5.65E-01	-1.48E+00	2.99E+02	4.42E+02
ATP5F1B	6.67E+03	9.25E-10	2.81E-08	5.79E-01	-7.89E-01	-1.73E+00	2.63E+02	4.55E+02
ATP5MC1	6.75E+02	4.57E-06	1.24E-05	6.91E-01	-5.34E-01	-1.45E+00	2.95E+01	4.28E+01
ATP5MC2	5.19E+03	6.14E-06	1.57E-05	6.98E-01	-5.19E-01	-1.43E+00	2.28E+02	3.27E+02
ATP5MC3	1.80E+03	9.88E-10	2.85E-08	5.04E-01	-9.88E-01	-1.98E+00	6.55E+01	1.30E+02
ATP5MD	2.69E+02	1.03E-04	1.75E-04	6.04E-01	-7.27E-01	-1.66E+00	1.09E+01	1.80E+01
ATP5ME	9.74E+02	9.02E-03	9.76E-03	7.80E-01	-3.59E-01	-1.28E+00	4.55E+01	5.83E+01
ATP5MPL	1.04E+03	8.60E-06	2.09E-05	6.27E-01	-6.74E-01	-1.60E+00	4.28E+01	6.82E+01
ATP5PD	1.37E+03	2.53E-05	5.17E-05	6.76E-01	-5.65E-01	-1.48E+00	5.88E+01	8.70E+01
ATP6	6.25E+04	9.00E-04	1.17E-03	7.11E-01	-4.93E-01	-1.41E+00	2.76E+03	3.88E+03
ATP6V1B1	1.65E+02	4.36E-04	6.15E-04	3.13E-01	-1.67E+00	-3.19E+00	4.22E+00	1.35E+01
ATP6V1C2	7.97E+01	1.28E-03	1.61E-03	3.99E-01	-1.32E+00	-2.51E+00	2.54E+00	6.37E+00
ATP8	2.26E+04	1.95E-03	2.37E-03	7.34E-01	-4.46E-01	-1.36E+00	1.01E+03	1.38E+03
ATP9B	1.14E+03	6.62E-03	7.29E-03	8.55E-01	-2.26E-01	-1.17E+00	5.57E+01	6.51E+01
ATPAF1	8.53E+02	2.53E-06	7.52E-06	7.44E-01	-4.26E-01	-1.34E+00	3.89E+01	5.22E+01
ATXN7L2	3.40E+02	3.43E-03	3.97E-03	8.17E-01	-2.92E-01	-1.22E+00	1.62E+01	1.99E+01

ATXN7L3	2.09E+03	1.18E-11	1.68E-09	5.71E-01	-8.08E-01	-1.75E+00	8.20E+01	1.44E+02
AURKAIP1	4.46E+02	8.21E-05	1.45E-04	7.58E-01	-4.00E-01	-1.32E+00	2.05E+01	2.71E+01
AURKB	4.90E+02	4.00E-08	3.31E-07	3.67E-01	-1.44E+00	-2.72E+00	1.46E+01	3.97E+01
AVIL	8.50E+01	4.41E-12	8.67E-10	1.44E-01	-2.80E+00	-6.94E+00	1.24E+00	8.63E+00
B3GALNT2	8.31E+02	2.54E-04	3.82E-04	7.71E-01	-3.75E-01	-1.30E+00	3.87E+01	5.01E+01
B3GALT4	3.47E+02	4.88E-06	1.31E-05	7.50E-01	-4.14E-01	-1.33E+00	1.59E+01	2.12E+01
B4GALNT3	2.05E+02	8.17E-10	2.62E-08	1.01E-01	-3.31E+00	-9.95E+00	2.16E+00	2.15E+01
B4GALT2	1.87E+03	3.53E-06	1.00E-05	7.57E-01	-4.03E-01	-1.32E+00	8.54E+01	1.13E+02
B4GALT3	7.54E+02	9.64E-05	1.65E-04	7.98E-01	-3.26E-01	-1.25E+00	3.56E+01	4.46E+01
B4GALT7	4.21E+02	2.26E-06	6.87E-06	7.49E-01	-4.17E-01	-1.33E+00	1.92E+01	2.56E+01
B9D1	2.88E+02	4.85E-06	1.30E-05	5.48E-01	-8.69E-01	-1.83E+00	1.10E+01	2.01E+01
B9D2	2.41E+02	6.37E-05	1.16E-04	6.73E-01	-5.71E-01	-1.49E+00	1.03E+01	1.54E+01
BAP1	2.10E+03	2.64E-09	5.11E-08	7.01E-01	-5.12E-01	-1.43E+00	9.25E+01	1.32E+02
BASP1	2.50E+03	2.62E-05	5.32E-05	5.31E-01	-9.13E-01	-1.88E+00	9.43E+01	1.78E+02
BAX	2.69E+02	5.73E-06	1.50E-05	6.46E-01	-6.31E-01	-1.55E+00	1.14E+01	1.76E+01
BCAR1	1.64E+03	4.66E-03	5.26E-03	8.76E-01	-1.90E-01	-1.14E+00	8.07E+01	9.21E+01
BCCIP	9.19E+02	7.66E-04	1.01E-03	7.57E-01	-4.01E-01	-1.32E+00	4.21E+01	5.56E+01
BCKDHB	2.75E+02	5.30E-05	9.87E-05	6.93E-01	-5.29E-01	-1.44E+00	1.21E+01	1.75E+01
BCKDK	8.81E+02	6.30E-07	2.58E-06	7.88E-01	-3.44E-01	-1.27E+00	4.12E+01	5.23E+01
BCL2L12	4.96E+02	4.18E-06	1.16E-05	7.33E-01	-4.48E-01	-1.36E+00	2.24E+01	3.06E+01
BCL3	4.01E+02	4.16E-06	1.15E-05	6.63E-01	-5.93E-01	-1.51E+00	1.72E+01	2.59E+01
BCO1	8.13E+00	2.40E-02	2.49E-02	2.24E-01	-2.16E+00	-4.47E+00	1.69E-01	7.57E-01
BDH1	7.15E+01	7.00E-04	9.33E-04	4.77E-01	-1.07E+00	-2.10E+00	2.52E+00	5.29E+00
BEAN1	1.40E+02	2.91E-05	5.81E-05	5.65E-01	-8.25E-01	-1.77E+00	5.43E+00	9.61E+00
BEND3	3.89E+02	4.85E-05	9.19E-05	6.96E-01	-5.22E-01	-1.44E+00	1.71E+01	2.46E+01
BEND5	3.69E+02	3.84E-03	4.42E-03	8.43E-01	-2.47E-01	-1.19E+00	1.78E+01	2.12E+01
BEX2-2	8.40E+02	2.47E-06	7.38E-06	5.68E-01	-8.17E-01	-1.76E+00	3.28E+01	5.77E+01
BEX5	9.11E+02	2.11E-05	4.42E-05	5.89E-01	-7.64E-01	-1.70E+00	3.64E+01	6.19E+01
BFAR	1.12E+03	5.44E-03	6.07E-03	8.50E-01	-2.34E-01	-1.18E+00	5.43E+01	6.39E+01
BFSP1	2.73E+02	6.06E-06	1.56E-05	4.53E-01	-1.14E+00	-2.21E+00	9.42E+00	2.08E+01
BIN1	1.32E+03	1.41E-07	8.58E-07	6.36E-01	-6.53E-01	-1.57E+00	5.53E+01	8.69E+01
BLVRA	1.23E+03	1.22E-08	1.34E-07	5.99E-01	-7.40E-01	-1.67E+00	5.00E+01	8.35E+01
BMP4	3.62E+02	3.76E-03	4.32E-03	6.74E-01	-5.68E-01	-1.48E+00	1.58E+01	2.34E+01

BORA	2.70E+02	2.76E-09	5.23E-08	4.76E-01	-1.07E+00	-2.10E+00	9.41E+00	1.98E+01
BORCS6	2.66E+02	4.80E-04	6.69E-04	7.43E-01	-4.29E-01	-1.35E+00	1.22E+01	1.64E+01
BORCS8	3.32E+02	1.08E-02	1.16E-02	8.15E-01	-2.95E-01	-1.23E+00	1.58E+01	1.94E+01
BPHL	4.62E+02	5.54E-03	6.16E-03	8.17E-01	-2.92E-01	-1.22E+00	2.22E+01	2.71E+01
BREH1	7.66E+01	2.01E-06	6.28E-06	2.50E-01	-2.00E+00	-3.99E+00	1.71E+00	6.82E+00
BRF2	3.37E+02	7.45E-05	1.32E-04	7.67E-01	-3.82E-01	-1.30E+00	1.56E+01	2.03E+01
BRICD5	1.77E+01	4.59E-03	5.19E-03	5.42E-01	-8.83E-01	-1.84E+00	6.65E-01	1.23E+00
BRINP1	1.81E+02	1.82E-03	2.22E-03	5.66E-01	-8.20E-01	-1.77E+00	7.10E+00	1.25E+01
BRMS1	7.16E+02	9.19E-09	1.11E-07	5.73E-01	-8.04E-01	-1.75E+00	2.82E+01	4.92E+01
BSCL2	6.23E+02	9.55E-03	1.03E-02	8.24E-01	-2.80E-01	-1.21E+00	2.96E+01	3.59E+01
BSG	6.90E+03	5.06E-04	7.01E-04	8.42E-01	-2.49E-01	-1.19E+00	3.35E+02	3.98E+02
BTD	2.41E+02	2.48E-03	2.95E-03	8.03E-01	-3.17E-01	-1.25E+00	1.13E+01	1.41E+01
C10H14orf119	6.12E+02	3.02E-05	6.01E-05	7.54E-01	-4.08E-01	-1.33E+00	2.79E+01	3.71E+01
C10H15orf59	8.32E+02	3.94E-07	1.82E-06	3.29E-01	-1.60E+00	-3.04E+00	2.29E+01	6.95E+01
C13H20orf27	9.93E+02	4.07E-06	1.14E-05	7.26E-01	-4.63E-01	-1.38E+00	4.44E+01	6.12E+01
C14H8orf82	3.49E+02	5.90E-04	8.01E-04	8.12E-01	-3.01E-01	-1.23E+00	1.67E+01	2.06E+01
C15H11orf49	5.24E+02	6.00E-06	1.56E-05	6.77E-01	-5.63E-01	-1.48E+00	2.26E+01	3.34E+01
C18H16orf74	1.64E+02	2.00E-08	1.98E-07	2.50E-01	-2.00E+00	-4.00E+00	3.69E+00	1.48E+01
C18H16orf78	1.23E+01	9.45E-09	1.13E-07	1.15E-02	-6.45E+00	-8.71E+01	1.66E-02	1.44E+00
C18H19orf33	5.51E+01	5.04E-08	3.94E-07	1.60E-01	-2.64E+00	-6.25E+00	8.89E-01	5.55E+00
C18H19orf48	7.86E+02	1.33E-04	2.18E-04	7.32E-01	-4.50E-01	-1.37E+00	3.55E+01	4.85E+01
C19H17orf49	9.45E+02	1.89E-04	2.96E-04	7.28E-01	-4.57E-01	-1.37E+00	4.25E+01	5.83E+01
C19H17orf67	4.33E+01	1.77E-09	3.98E-08	7.90E-02	-3.66E+00	-1.27E+01	3.74E-01	4.73E+00
C1QBP	1.23E+03	1.28E-04	2.11E-04	7.33E-01	-4.48E-01	-1.36E+00	5.60E+01	7.64E+01
C22H3orf62	1.92E+02	5.43E-03	6.06E-03	7.40E-01	-4.34E-01	-1.35E+00	8.68E+00	1.17E+01
C25H16orf45	2.10E+02	6.72E-07	2.70E-06	3.39E-01	-1.56E+00	-2.95E+00	5.84E+00	1.72E+01
C25H16orf91	4.57E+02	2.77E-05	5.58E-05	7.89E-01	-3.43E-01	-1.27E+00	2.14E+01	2.72E+01
C3H1orf210	4.06E+01	4.83E-04	6.73E-04	2.75E-01	-1.86E+00	-3.63E+00	1.01E+00	3.66E+00
C3H1orf226	3.20E+02	1.30E-05	2.94E-05	4.47E-01	-1.16E+00	-2.24E+00	1.09E+01	2.44E+01
C3H1orf94	2.25E+00	5.06E-04	7.01E-04	1.09E-01	-3.20E+00	-9.19E+00	2.51E-02	2.31E-01
C5H12orf73	3.49E+02	1.54E-05	3.37E-05	7.40E-01	-4.34E-01	-1.35E+00	1.58E+01	2.14E+01
C7H19orf25	3.92E+02	2.58E-04	3.86E-04	7.44E-01	-4.27E-01	-1.34E+00	1.78E+01	2.40E+01
C7H19orf53	1.54E+03	1.08E-04	1.81E-04	7.81E-01	-3.57E-01	-1.28E+00	7.21E+01	9.24E+01

C7H19orf70	3.23E+02	2.08E-03	2.51E-03	7.08E-01	-4.99E-01	-1.41E+00	1.43E+01	2.02E+01
C8H9orf3	1.21E+03	3.19E-04	4.65E-04	6.98E-01	-5.19E-01	-1.43E+00	5.30E+01	7.60E+01
C8H9orf40	2.55E+02	1.50E-04	2.43E-04	7.51E-01	-4.13E-01	-1.33E+00	1.16E+01	1.54E+01
C9H6orf203	2.95E+02	1.13E-04	1.88E-04	7.66E-01	-3.85E-01	-1.31E+00	1.36E+01	1.78E+01
CA11	4.39E+02	6.95E-04	9.30E-04	7.30E-01	-4.53E-01	-1.37E+00	1.97E+01	2.70E+01
CA14	3.84E+02	3.60E-04	5.18E-04	6.31E-01	-6.65E-01	-1.59E+00	1.60E+01	2.54E+01
CA8	2.96E+02	1.59E-04	2.56E-04	4.92E-01	-1.02E+00	-2.03E+00	1.07E+01	2.17E+01
CACFD1	8.07E+02	7.27E-08	5.26E-07	6.49E-01	-6.24E-01	-1.54E+00	3.42E+01	5.27E+01
CACNA1H	4.38E+03	4.13E-03	4.72E-03	6.88E-01	-5.39E-01	-1.45E+00	1.92E+02	2.79E+02
CACNG4	2.69E+02	1.91E-07	1.06E-06	3.69E-01	-1.44E+00	-2.71E+00	7.98E+00	2.16E+01
CACTIN	1.15E+03	1.34E-06	4.55E-06	7.79E-01	-3.59E-01	-1.28E+00	5.35E+01	6.86E+01
CADM4	7.15E+02	5.27E-09	7.66E-08	3.30E-01	-1.60E+00	-3.03E+00	1.96E+01	5.93E+01
CALB2	2.55E+02	1.80E-08	1.80E-07	1.40E-01	-2.84E+00	-7.14E+00	3.49E+00	2.50E+01
CAMKK1	1.49E+02	4.46E-08	3.62E-07	4.47E-01	-1.16E+00	-2.24E+00	5.02E+00	1.12E+01
CAMKV	2.92E+02	1.13E-10	6.64E-09	6.77E-02	-3.88E+00	-1.48E+01	2.16E+00	3.19E+01
CAPN10	7.36E+02	1.66E-06	5.40E-06	7.06E-01	-5.03E-01	-1.42E+00	3.25E+01	4.61E+01
CAPN12	7.63E+00	8.94E-02	9.01E-02	6.73E-01	-5.72E-01	-1.49E+00	3.31E-01	4.92E-01
CAPN14	2.44E+01	1.15E-06	4.10E-06	1.04E-01	-3.26E+00	-9.59E+00	2.71E-01	2.59E+00
CARHSP1	1.29E+03	4.86E-04	6.76E-04	7.90E-01	-3.41E-01	-1.27E+00	6.06E+01	7.68E+01
CASKIN2	2.41E+03	1.89E-07	1.06E-06	7.20E-01	-4.74E-01	-1.39E+00	1.08E+02	1.50E+02
CASP3	5.19E+02	7.71E-07	3.01E-06	6.89E-01	-5.37E-01	-1.45E+00	2.28E+01	3.30E+01
CBARP	2.38E+02	8.26E-08	5.82E-07	3.82E-01	-1.39E+00	-2.61E+00	7.19E+00	1.88E+01
CBLN4	1.43E+02	1.14E-03	1.45E-03	3.41E-01	-1.55E+00	-2.93E+00	4.07E+00	1.19E+01
CBS	8.57E+02	7.99E-07	3.10E-06	4.91E-01	-1.03E+00	-2.04E+00	3.07E+01	6.25E+01
CBX4	9.87E+02	6.25E-06	1.60E-05	7.33E-01	-4.49E-01	-1.36E+00	4.44E+01	6.06E+01
CBY1	9.88E+02	3.89E-05	7.54E-05	7.84E-01	-3.50E-01	-1.27E+00	4.63E+01	5.90E+01
CCDC124	6.66E+02	5.80E-03	6.43E-03	8.56E-01	-2.25E-01	-1.17E+00	3.26E+01	3.80E+01
CCDC148	7.84E+00	2.91E-02	3.01E-02	6.15E-01	-7.02E-01	-1.63E+00	3.15E-01	5.12E-01
CCDC173	5.11E+01	2.78E-03	3.28E-03	5.84E-01	-7.76E-01	-1.71E+00	1.99E+00	3.40E+00
CCDC189	4.67E+02	6.48E-04	8.70E-04	7.17E-01	-4.80E-01	-1.39E+00	2.09E+01	2.91E+01
CCDC28B	1.29E+03	2.43E-04	3.67E-04	7.19E-01	-4.77E-01	-1.39E+00	5.76E+01	8.02E+01
CCDC68	4.68E+01	3.98E-08	3.31E-07	1.98E-01	-2.34E+00	-5.05E+00	8.79E-01	4.44E+00
CCDC70	4.51E+00	2.50E-07	1.29E-06	1.21E-02	-6.37E+00	-8.25E+01	6.37E-03	5.26E-01

CCDC86	4.23E+02	4.25E-03	4.83E-03	7.89E-01	-3.42E-01	-1.27E+00	1.98E+01	2.51E+01
CCKBR	4.76E+02	1.23E-06	4.32E-06	2.05E-01	-2.29E+00	-4.88E+00	8.95E+00	4.37E+01
CCL25	1.25E+03	6.17E-08	4.68E-07	4.03E-01	-1.31E+00	-2.48E+00	3.93E+01	9.76E+01
CCNA2	2.99E+02	1.60E-06	5.24E-06	5.56E-01	-8.48E-01	-1.80E+00	1.16E+01	2.08E+01
CCNL2	1.45E+03	1.81E-06	5.76E-06	8.09E-01	-3.06E-01	-1.24E+00	6.93E+01	8.57E+01
CCRL2	2.12E+01	2.14E-02	2.23E-02	7.01E-01	-5.12E-01	-1.43E+00	9.32E-01	1.33E+00
CD19	9.22E+01	4.31E-04	6.08E-04	5.22E-01	-9.39E-01	-1.92E+00	3.39E+00	6.49E+00
CD24	3.09E+02	5.65E-11	4.67E-09	7.11E-02	-3.81E+00	-1.41E+01	2.45E+00	3.45E+01
CD2BP2	1.51E+03	7.28E-05	1.30E-04	8.11E-01	-3.03E-01	-1.23E+00	7.19E+01	8.87E+01
CD320	5.28E+02	7.51E-07	2.95E-06	5.93E-01	-7.55E-01	-1.69E+00	2.12E+01	3.57E+01
CD3G	1.53E+02	1.26E-08	1.38E-07	2.12E-01	-2.24E+00	-4.71E+00	2.94E+00	1.38E+01
CD79B	3.06E+01	1.28E-05	2.91E-05	1.48E-01	-2.75E+00	-6.75E+00	4.62E-01	3.12E+00
CD8B	8.62E+01	8.60E-08	6.00E-07	2.12E-01	-2.24E+00	-4.73E+00	1.71E+00	8.07E+00
CD9	1.92E+03	7.31E-08	5.26E-07	2.48E-01	-2.01E+00	-4.03E+00	4.31E+01	1.74E+02
CDC25B	4.74E+02	2.79E-04	4.13E-04	7.48E-01	-4.20E-01	-1.34E+00	2.16E+01	2.89E+01
CDC42EP3	4.67E+02	1.47E-05	3.25E-05	5.58E-01	-8.43E-01	-1.79E+00	1.84E+01	3.29E+01
CDCA3	3.63E+02	1.66E-07	9.57E-07	4.20E-01	-1.25E+00	-2.38E+00	1.17E+01	2.80E+01
CDCP1	2.80E+02	1.45E-02	1.53E-02	6.67E-01	-5.85E-01	-1.50E+00	1.18E+01	1.77E+01
CDH24	7.91E+02	5.69E-09	8.11E-08	4.55E-01	-1.14E+00	-2.20E+00	2.71E+01	5.96E+01
CDH4	4.84E+02	2.38E-03	2.84E-03	5.41E-01	-8.86E-01	-1.85E+00	1.85E+01	3.42E+01
CDH9	5.37E+01	4.44E-04	6.24E-04	2.82E-01	-1.82E+00	-3.54E+00	1.27E+00	4.49E+00
CDHR1	2.40E+01	5.67E-03	6.29E-03	1.29E-01	-2.96E+00	-7.76E+00	3.21E-01	2.49E+00
CDHR4	1.71E+01	5.46E-02	5.54E-02	4.33E-01	-1.21E+00	-2.31E+00	5.48E-01	1.26E+00
CDIP1	1.45E+03	4.78E-09	7.16E-08	6.73E-01	-5.72E-01	-1.49E+00	6.25E+01	9.30E+01
CDIPT	8.30E+02	3.73E-10	1.43E-08	6.35E-01	-6.54E-01	-1.57E+00	3.48E+01	5.48E+01
CDK18	3.83E+02	3.87E-04	5.51E-04	5.74E-01	-8.00E-01	-1.74E+00	1.52E+01	2.64E+01
CDK2	9.38E+02	1.60E-09	3.68E-08	5.95E-01	-7.49E-01	-1.68E+00	3.76E+01	6.31E+01
CDK20	9.54E+02	6.94E-09	9.01E-08	6.44E-01	-6.36E-01	-1.55E+00	3.99E+01	6.21E+01
CDK4	1.82E+03	3.63E-08	3.12E-07	6.47E-01	-6.28E-01	-1.55E+00	7.70E+01	1.19E+02
CDKN1C	1.34E+03	1.03E-07	6.93E-07	5.40E-01	-8.89E-01	-1.85E+00	5.09E+01	9.42E+01
CDKN2AIPNL	5.80E+02	3.24E-03	3.77E-03	8.32E-01	-2.65E-01	-1.20E+00	2.80E+01	3.37E+01
CDX1	2.78E+02	5.88E-09	8.26E-08	6.05E-02	-4.05E+00	-1.65E+01	1.87E+00	3.08E+01
CEP78	2.53E+02	6.62E-07	2.68E-06	7.05E-01	-5.04E-01	-1.42E+00	1.12E+01	1.58E+01

CERK	1.05E+03	1.96E-06	6.14E-06	7.16E-01	-4.81E-01	-1.40E+00	4.68E+01	6.54E+01
CERS4	1.07E+03	1.20E-03	1.52E-03	6.45E-01	-6.33E-01	-1.55E+00	4.51E+01	6.99E+01
CETN2	1.56E+03	2.39E-05	4.93E-05	6.89E-01	-5.38E-01	-1.45E+00	6.73E+01	9.78E+01
CETN4	4.45E+02	9.25E-06	2.22E-05	3.90E-01	-1.36E+00	-2.57E+00	1.32E+01	3.40E+01
CFAP161	1.29E+02	9.05E-06	2.19E-05	3.43E-01	-1.55E+00	-2.92E+00	3.68E+00	1.07E+01
CFL1	1.16E+04	6.63E-06	1.69E-05	7.28E-01	-4.59E-01	-1.37E+00	5.23E+02	7.19E+02
CHCHD1	5.32E+02	3.74E-06	1.05E-05	6.65E-01	-5.88E-01	-1.50E+00	2.26E+01	3.40E+01
CHCHD5	2.18E+02	6.46E-03	7.12E-03	7.77E-01	-3.65E-01	-1.29E+00	1.01E+01	1.30E+01
CHCHD8	1.98E+02	6.36E-05	1.16E-04	7.66E-01	-3.84E-01	-1.31E+00	9.13E+00	1.19E+01
CHD1L	4.99E+02	2.92E-03	3.43E-03	8.52E-01	-2.31E-01	-1.17E+00	2.44E+01	2.87E+01
chrM:1453..302	4.85E+03	5.36E-04	7.36E-04	5.07E-01	-9.79E-01	-1.97E+00	1.76E+02	3.47E+02
rM:15726..157	3.36E+00	7.34E-01	7.34E-01	4.30E-01	-1.22E+00	-2.33E+00	1.11E-01	2.58E-01
chrM:431..1385	1.93E+03	1.13E-02	1.21E-02	7.13E-01	-4.88E-01	-1.40E+00	8.59E+01	1.21E+02
CHRNA2	9.08E+01	6.11E-06	1.57E-05	1.50E-01	-2.74E+00	-6.68E+00	1.36E+00	9.11E+00
CHRNA5	6.92E+01	1.79E-05	3.86E-05	5.47E-01	-8.69E-01	-1.83E+00	2.64E+00	4.83E+00
CHRNB2	1.35E+01	2.73E-03	3.23E-03	4.68E-01	-1.09E+00	-2.14E+00	4.71E-01	1.01E+00
CHRNB4	1.95E+02	4.00E-06	1.12E-05	3.58E-01	-1.48E+00	-2.79E+00	5.57E+00	1.56E+01
CKM	1.29E+01	1.15E-07	7.49E-07	1.25E-01	-3.00E+00	-7.98E+00	1.62E-01	1.30E+00
CLBA1	8.44E+01	1.92E-04	3.00E-04	5.66E-01	-8.21E-01	-1.77E+00	3.24E+00	5.73E+00
CLDN6	9.63E+01	4.74E-12	8.67E-10	6.73E-03	-7.21E+00	-1.49E+02	7.82E-02	1.16E+01
CLIP2	2.92E+03	2.57E-07	1.32E-06	6.88E-01	-5.40E-01	-1.45E+00	1.28E+02	1.85E+02
CLN6	1.11E+03	5.76E-05	1.06E-04	6.88E-01	-5.40E-01	-1.45E+00	4.84E+01	7.04E+01
CLPP	6.01E+02	3.08E-03	3.60E-03	8.61E-01	-2.16E-01	-1.16E+00	2.96E+01	3.44E+01
CLSTN3	2.59E+03	7.59E-06	1.89E-05	4.04E-01	-1.31E+00	-2.47E+00	8.11E+01	2.01E+02
CLUH	1.66E+03	9.00E-05	1.56E-04	6.78E-01	-5.62E-01	-1.48E+00	7.23E+01	1.07E+02
CMIP	2.50E+03	2.54E-05	5.18E-05	6.91E-01	-5.33E-01	-1.45E+00	1.09E+02	1.58E+02
CMTM5	2.37E+01	1.94E-05	4.11E-05	4.36E-01	-1.20E+00	-2.29E+00	7.91E-01	1.81E+00
CNIH2	2.27E+02	1.33E-08	1.43E-07	3.51E-01	-1.51E+00	-2.85E+00	6.57E+00	1.87E+01
CNKSR1	6.41E+01	4.39E-05	8.42E-05	4.54E-01	-1.14E+00	-2.20E+00	2.18E+00	4.79E+00
CNNM1	4.62E+01	5.47E-05	1.01E-04	1.46E-02	-6.10E+00	-6.84E+01	8.03E-02	5.49E+00
CNPY3	9.70E+02	4.29E-06	1.17E-05	8.07E-01	-3.10E-01	-1.24E+00	4.60E+01	5.70E+01
CNTF	1.05E+01	1.22E-01	1.22E-01	7.02E-01	-5.10E-01	-1.42E+00	4.53E-01	6.45E-01
COA3	7.09E+02	1.27E-05	2.89E-05	6.86E-01	-5.43E-01	-1.46E+00	3.09E+01	4.50E+01

COASY	5.29E+02	5.33E-06	1.40E-05	7.50E-01	-4.15E-01	-1.33E+00	2.41E+01	3.22E+01
COL18A1	1.35E+04	1.62E-03	2.00E-03	7.02E-01	-5.11E-01	-1.43E+00	5.94E+02	8.46E+02
COL23A1	1.28E+02	5.04E-06	1.34E-05	3.40E-01	-1.56E+00	-2.94E+00	3.61E+00	1.06E+01
COL26A1	1.91E+03	2.30E-07	1.22E-06	1.97E-01	-2.34E+00	-5.08E+00	3.50E+01	1.78E+02
COMMD1	8.32E+02	1.28E-03	1.61E-03	7.93E-01	-3.34E-01	-1.26E+00	3.91E+01	4.93E+01
COMMD3	7.92E+02	6.14E-06	1.57E-05	7.37E-01	-4.40E-01	-1.36E+00	3.58E+01	4.86E+01
COMMD5	5.31E+02	6.57E-05	1.19E-04	8.16E-01	-2.94E-01	-1.23E+00	2.53E+01	3.10E+01
COMMD9	6.90E+02	8.00E-06	1.97E-05	7.40E-01	-4.35E-01	-1.35E+00	3.12E+01	4.22E+01
COPE	2.12E+03	6.39E-06	1.64E-05	7.39E-01	-4.36E-01	-1.35E+00	9.59E+01	1.30E+02
COPS4	1.02E+03	1.32E-03	1.65E-03	8.72E-01	-1.97E-01	-1.15E+00	5.05E+01	5.78E+01
COPS5	6.91E+02	7.84E-06	1.94E-05	7.52E-01	-4.10E-01	-1.33E+00	3.17E+01	4.22E+01
COQ5	2.35E+02	1.02E-02	1.09E-02	8.61E-01	-2.16E-01	-1.16E+00	1.15E+01	1.33E+01
COQ8B	2.73E+02	2.88E-05	5.76E-05	7.70E-01	-3.76E-01	-1.30E+00	1.26E+01	1.64E+01
COTL1	2.79E+03	4.96E-07	2.17E-06	6.31E-01	-6.64E-01	-1.58E+00	1.16E+02	1.84E+02
COX1	2.30E+05	2.23E-03	2.68E-03	6.55E-01	-6.10E-01	-1.53E+00	9.70E+03	1.48E+04
COX19	1.28E+03	1.13E-05	2.62E-05	7.88E-01	-3.43E-01	-1.27E+00	6.01E+01	7.62E+01
COX2	2.40E+04	2.12E-04	3.27E-04	6.06E-01	-7.23E-01	-1.65E+00	9.74E+02	1.61E+03
COX3	9.42E+04	4.98E-04	6.90E-04	6.37E-01	-6.50E-01	-1.57E+00	3.91E+03	6.13E+03
COX4I1	4.35E+03	6.13E-04	8.28E-04	7.92E-01	-3.36E-01	-1.26E+00	2.05E+02	2.58E+02
COX6B1	1.60E+03	3.71E-06	1.05E-05	6.46E-01	-6.30E-01	-1.55E+00	6.72E+01	1.04E+02
COX7B	6.41E+02	6.35E-05	1.16E-04	6.93E-01	-5.29E-01	-1.44E+00	2.80E+01	4.04E+01
COX8A	1.47E+03	1.35E-07	8.42E-07	6.70E-01	-5.77E-01	-1.49E+00	6.34E+01	9.46E+01
CPAMD8	1.12E+03	8.57E-07	3.26E-06	2.87E-01	-1.80E+00	-3.48E+00	2.75E+01	9.58E+01
CPLX3	4.13E+01	4.87E-03	5.47E-03	1.42E-01	-2.81E+00	-7.02E+00	5.90E-01	4.14E+00
CRABP2	5.33E+02	3.86E-04	5.51E-04	4.59E-01	-1.12E+00	-2.18E+00	1.85E+01	4.03E+01
CREB3	5.97E+02	7.03E-04	9.36E-04	8.28E-01	-2.73E-01	-1.21E+00	2.87E+01	3.46E+01
CRLF1	2.78E+02	2.00E-07	1.10E-06	5.14E-02	-4.28E+00	-1.95E+01	1.61E+00	3.13E+01
CRMP1	8.17E+02	8.97E-08	6.22E-07	3.92E-01	-1.35E+00	-2.55E+00	2.54E+01	6.49E+01
CRTAC1	9.47E+01	2.35E-06	7.09E-06	1.53E-01	-2.71E+00	-6.54E+00	1.39E+00	9.10E+00
CS	1.52E+03	1.07E-04	1.80E-04	7.90E-01	-3.41E-01	-1.27E+00	7.12E+01	9.02E+01
CSKMT	2.50E+02	1.24E-03	1.57E-03	4.74E-01	-1.08E+00	-2.11E+00	8.86E+00	1.87E+01
CSNK1G2	2.66E+03	7.89E-04	1.04E-03	7.40E-01	-4.34E-01	-1.35E+00	1.21E+02	1.63E+02
CSRNP1	3.27E+02	1.34E-06	4.55E-06	5.14E-01	-9.62E-01	-1.95E+00	1.21E+01	2.35E+01

CSTF2T	1.38E+03	4.10E-09	6.70E-08	6.27E-01	-6.74E-01	-1.60E+00	5.68E+01	9.07E+01
CT55	3.86E+01	5.55E-07	2.39E-06	1.19E-02	-6.39E+00	-8.40E+01	5.29E-02	4.44E+00
CTBP1	3.46E+03	3.82E-07	1.78E-06	7.61E-01	-3.94E-01	-1.31E+00	1.59E+02	2.09E+02
CTRC	1.82E+00	6.59E-02	6.65E-02	1.74E-01	-2.52E+00	-5.75E+00	2.98E-02	1.71E-01
CTSD	8.87E+03	3.90E-07	1.81E-06	6.14E-01	-7.04E-01	-1.63E+00	3.64E+02	5.92E+02
CTSV	2.32E+03	1.66E-07	9.57E-07	6.14E-01	-7.04E-01	-1.63E+00	9.54E+01	1.55E+02
CUTA	8.99E+02	1.20E-06	4.23E-06	6.68E-01	-5.83E-01	-1.50E+00	3.84E+01	5.76E+01
CXXC1	1.90E+03	2.88E-04	4.25E-04	8.59E-01	-2.19E-01	-1.16E+00	9.30E+01	1.08E+02
CYB561	1.55E+02	4.37E-02	4.46E-02	6.19E-01	-6.93E-01	-1.62E+00	6.19E+00	1.00E+01
CYB561A3	7.20E+02	1.72E-05	3.72E-05	6.41E-01	-6.41E-01	-1.56E+00	3.00E+01	4.69E+01
CYC1	1.26E+03	1.36E-07	8.43E-07	5.89E-01	-7.65E-01	-1.70E+00	5.02E+01	8.53E+01
CYP2S1	1.37E+02	5.62E-06	1.47E-05	1.39E-01	-2.85E+00	-7.20E+00	1.96E+00	1.41E+01
CYP4F2	4.02E+02	8.16E-06	2.00E-05	3.14E-01	-1.67E+00	-3.18E+00	1.05E+01	3.36E+01
CYTB	5.81E+04	7.14E-04	9.49E-04	6.58E-01	-6.03E-01	-1.52E+00	2.47E+03	3.75E+03
D2HGDH	7.05E+02	1.25E-06	4.37E-06	6.84E-01	-5.48E-01	-1.46E+00	3.06E+01	4.47E+01
DAD1	1.51E+03	5.23E-06	1.38E-05	7.26E-01	-4.62E-01	-1.38E+00	6.75E+01	9.30E+01
DBN1	4.18E+03	7.46E-10	2.43E-08	5.56E-01	-8.46E-01	-1.80E+00	1.62E+02	2.90E+02
DBNL	2.99E+03	2.99E-06	8.66E-06	7.89E-01	-3.42E-01	-1.27E+00	1.40E+02	1.78E+02
DCAF12L2	1.71E+02	1.56E-11	1.78E-09	4.05E-02	-4.62E+00	-2.47E+01	7.69E-01	1.90E+01
DDAH2	1.98E+03	7.03E-04	9.36E-04	7.62E-01	-3.93E-01	-1.31E+00	9.15E+01	1.20E+02
DDIT4	2.31E+03	8.52E-08	5.96E-07	3.96E-01	-1.34E+00	-2.53E+00	7.26E+01	1.83E+02
DDOST	4.43E+03	1.03E-06	3.75E-06	7.38E-01	-4.38E-01	-1.35E+00	2.00E+02	2.72E+02
DDT	3.84E+02	5.16E-05	9.67E-05	5.29E-01	-9.18E-01	-1.89E+00	1.46E+01	2.76E+01
DDX11	9.77E+02	2.34E-06	7.08E-06	5.02E-01	-9.95E-01	-1.99E+00	3.53E+01	7.03E+01
DDX19B	6.46E+02	7.20E-03	7.88E-03	8.46E-01	-2.42E-01	-1.18E+00	3.13E+01	3.70E+01
DDX51	8.18E+02	2.78E-07	1.39E-06	5.77E-01	-7.93E-01	-1.73E+00	3.23E+01	5.60E+01
DDX54	1.91E+03	1.47E-06	4.92E-06	6.09E-01	-7.15E-01	-1.64E+00	7.80E+01	1.28E+02
DECR2	4.24E+02	1.16E-07	7.49E-07	6.88E-01	-5.40E-01	-1.45E+00	1.84E+01	2.68E+01
DEF6	2.51E+02	3.51E-06	9.99E-06	5.08E-01	-9.76E-01	-1.97E+00	9.15E+00	1.80E+01
DEGS1	1.31E+03	1.63E-04	2.61E-04	8.13E-01	-2.99E-01	-1.23E+00	6.20E+01	7.63E+01
DEGS2	3.14E+01	7.81E-04	1.03E-03	4.37E-01	-1.19E+00	-2.29E+00	1.06E+00	2.41E+00
DENND1A	1.92E+03	1.10E-02	1.18E-02	8.11E-01	-3.02E-01	-1.23E+00	9.13E+01	1.12E+02
DENND3	3.73E+02	2.84E-02	2.93E-02	7.82E-01	-3.54E-01	-1.28E+00	1.73E+01	2.21E+01

DEUP1	2.83E+01	9.40E-04	1.21E-03	3.89E-01	-1.36E+00	-2.57E+00	8.43E-01	2.17E+00
DEXI	4.76E+02	8.40E-04	1.09E-03	8.02E-01	-3.18E-01	-1.25E+00	2.26E+01	2.81E+01
DGAT1	1.35E+03	1.70E-07	9.77E-07	6.08E-01	-7.18E-01	-1.65E+00	5.49E+01	9.03E+01
DGCR6L	1.11E+03	2.21E-05	4.59E-05	7.25E-01	-4.63E-01	-1.38E+00	4.97E+01	6.85E+01
DGKA	9.50E+02	6.48E-03	7.13E-03	8.37E-01	-2.57E-01	-1.20E+00	4.59E+01	5.49E+01
DGKQ	4.39E+02	1.16E-06	4.12E-06	5.70E-01	-8.11E-01	-1.75E+00	1.72E+01	3.01E+01
DGUOK	4.90E+02	2.17E-05	4.53E-05	7.73E-01	-3.71E-01	-1.29E+00	2.27E+01	2.94E+01
DHDH	1.76E+02	2.80E-06	8.26E-06	5.72E-01	-8.05E-01	-1.75E+00	6.92E+00	1.21E+01
DHRS11	7.60E+02	2.08E-09	4.39E-08	5.97E-01	-7.44E-01	-1.67E+00	3.08E+01	5.16E+01
DISP3	8.49E+01	5.33E-06	1.40E-05	1.29E-01	-2.95E+00	-7.74E+00	1.14E+00	8.84E+00
DKKL1	4.44E+02	4.12E-09	6.70E-08	4.06E-01	-1.30E+00	-2.46E+00	1.40E+01	3.44E+01
DLGAP4	3.68E+03	1.30E-05	2.93E-05	7.04E-01	-5.06E-01	-1.42E+00	1.62E+02	2.30E+02
DMTN	2.59E+02	9.98E-08	6.75E-07	3.82E-01	-1.39E+00	-2.62E+00	7.81E+00	2.05E+01
DNAI2	8.71E+02	2.33E-04	3.53E-04	5.15E-01	-9.57E-01	-1.94E+00	3.17E+01	6.15E+01
DNAJC19	1.37E+02	5.70E-07	2.42E-06	5.68E-01	-8.16E-01	-1.76E+00	5.37E+00	9.45E+00
DNAL4	4.04E+02	3.25E-04	4.72E-04	7.08E-01	-4.97E-01	-1.41E+00	1.80E+01	2.54E+01
DOC2A	5.07E+01	4.24E-09	6.76E-08	2.29E-01	-2.13E+00	-4.37E+00	1.06E+00	4.65E+00
DOCK6	1.69E+03	3.84E-07	1.79E-06	4.80E-01	-1.06E+00	-2.08E+00	6.04E+01	1.26E+02
DOHH	2.54E+02	3.77E-04	5.39E-04	7.85E-01	-3.48E-01	-1.27E+00	1.19E+01	1.51E+01
DOK4	9.38E+02	2.07E-06	6.41E-06	3.99E-01	-1.32E+00	-2.50E+00	2.97E+01	7.43E+01
DOLK	4.16E+02	1.00E-07	6.75E-07	6.71E-01	-5.75E-01	-1.49E+00	1.78E+01	2.66E+01
DPEP1	2.56E+02	2.43E-08	2.23E-07	1.78E-01	-2.49E+00	-5.61E+00	4.38E+00	2.45E+01
DPH7	4.71E+02	1.68E-04	2.68E-04	7.96E-01	-3.28E-01	-1.26E+00	2.22E+01	2.79E+01
DPP3	4.95E+02	4.77E-03	5.38E-03	7.92E-01	-3.37E-01	-1.26E+00	2.32E+01	2.94E+01
DPY30	6.05E+02	1.83E-05	3.92E-05	7.28E-01	-4.57E-01	-1.37E+00	2.72E+01	3.73E+01
DPYSL5	3.74E+02	7.24E-11	5.30E-09	8.60E-02	-3.54E+00	-1.16E+01	3.41E+00	3.96E+01
DSCAM	1.48E+01	4.10E-07	1.88E-06	9.59E-02	-3.38E+00	-1.04E+01	1.49E-01	1.55E+00
DSG2	4.95E+02	3.50E-04	5.05E-04	4.22E-01	-1.24E+00	-2.37E+00	1.61E+01	3.81E+01
DTD1	1.27E+03	1.71E-04	2.72E-04	8.14E-01	-2.97E-01	-1.23E+00	6.06E+01	7.45E+01
DTL	3.55E+02	1.84E-04	2.90E-04	6.72E-01	-5.74E-01	-1.49E+00	1.53E+01	2.28E+01
DUS2	1.60E+02	3.47E-05	6.82E-05	6.62E-01	-5.95E-01	-1.51E+00	6.73E+00	1.02E+01
DUS3L	5.86E+02	3.58E-06	1.01E-05	7.00E-01	-5.15E-01	-1.43E+00	2.58E+01	3.68E+01
DUSP15	3.85E+02	1.49E-04	2.40E-04	7.02E-01	-5.11E-01	-1.42E+00	1.69E+01	2.41E+01

DUSP26	2.93E+02	4.94E-04	6.86E-04	5.74E-01	-8.01E-01	-1.74E+00	1.16E+01	2.03E+01
DUSP4	7.71E+01	7.39E-04	9.77E-04	4.77E-01	-1.07E+00	-2.10E+00	2.74E+00	5.73E+00
DUT	9.42E+02	6.73E-06	1.71E-05	5.47E-01	-8.70E-01	-1.83E+00	3.61E+01	6.60E+01
DYRK1B	1.21E+03	6.64E-07	2.68E-06	7.01E-01	-5.13E-01	-1.43E+00	5.34E+01	7.62E+01
E2F8	1.18E+02	2.98E-05	5.94E-05	4.81E-01	-1.06E+00	-2.08E+00	4.17E+00	8.68E+00
EBP	2.51E+02	1.65E-02	1.74E-02	7.81E-01	-3.56E-01	-1.28E+00	1.18E+01	1.50E+01
ECI1	7.06E+02	4.45E-04	6.25E-04	7.15E-01	-4.84E-01	-1.40E+00	3.15E+01	4.40E+01
EDAR	5.68E+01	2.24E-05	4.66E-05	4.66E-01	-1.10E+00	-2.15E+00	1.97E+00	4.22E+00
EDF1	2.19E+03	9.22E-05	1.59E-04	7.43E-01	-4.29E-01	-1.35E+00	9.93E+01	1.34E+02
EEF1A2	2.93E+02	2.27E-08	2.15E-07	7.20E-02	-3.80E+00	-1.39E+01	2.23E+00	3.09E+01
EFCAB11	1.45E+02	1.49E-04	2.41E-04	6.02E-01	-7.33E-01	-1.66E+00	5.77E+00	9.59E+00
EFNA4	3.39E+02	3.98E-10	1.48E-08	3.75E-01	-1.42E+00	-2.67E+00	1.02E+01	2.72E+01
EFNB3	1.03E+03	5.19E-04	7.15E-04	6.88E-01	-5.39E-01	-1.45E+00	4.50E+01	6.54E+01
EFS	1.97E+03	4.94E-04	6.86E-04	6.85E-01	-5.45E-01	-1.46E+00	8.60E+01	1.25E+02
EGLN2	7.69E+02	1.22E-06	4.30E-06	6.58E-01	-6.03E-01	-1.52E+00	3.26E+01	4.95E+01
EHMT2	5.34E+03	1.82E-05	3.92E-05	7.04E-01	-5.05E-01	-1.42E+00	2.35E+02	3.34E+02
EID2	1.49E+02	9.06E-05	1.57E-04	7.31E-01	-4.52E-01	-1.37E+00	6.71E+00	9.18E+00
EIF1	3.64E+03	1.16E-03	1.47E-03	8.11E-01	-3.02E-01	-1.23E+00	1.74E+02	2.14E+02
EIF3D	5.53E+03	2.04E-03	2.47E-03	8.60E-01	-2.18E-01	-1.16E+00	2.71E+02	3.15E+02
EIF3I	2.10E+03	1.78E-05	3.84E-05	7.14E-01	-4.86E-01	-1.40E+00	9.32E+01	1.31E+02
EIF3M	6.22E+03	2.18E-04	3.34E-04	7.77E-01	-3.63E-01	-1.29E+00	2.90E+02	3.73E+02
EIF4A1	6.41E+03	1.54E-05	3.38E-05	6.40E-01	-6.44E-01	-1.56E+00	2.70E+02	4.22E+02
ELOB	1.32E+03	5.24E-09	7.66E-08	5.42E-01	-8.84E-01	-1.85E+00	5.01E+01	9.24E+01
EMC4	7.34E+02	8.80E-07	3.31E-06	7.39E-01	-4.36E-01	-1.35E+00	3.32E+01	4.48E+01
EMC6	3.66E+02	4.93E-07	2.17E-06	6.22E-01	-6.85E-01	-1.61E+00	1.51E+01	2.43E+01
EMG1	4.39E+02	6.05E-07	2.52E-06	5.79E-01	-7.89E-01	-1.73E+00	1.73E+01	2.99E+01
EMILIN3	6.27E+02	2.23E-04	3.41E-04	4.85E-01	-1.04E+00	-2.06E+00	2.23E+01	4.59E+01
ENDOU	1.14E+01	6.10E-03	6.73E-03	3.96E-01	-1.34E+00	-2.52E+00	3.62E-01	9.13E-01
ENO1	9.88E+03	3.05E-04	4.46E-04	6.65E-01	-5.89E-01	-1.50E+00	4.28E+02	6.43E+02
ENOX1	3.25E+02	3.92E-04	5.58E-04	6.87E-01	-5.42E-01	-1.46E+00	1.42E+01	2.07E+01
EPHA1	1.57E+02	5.94E-06	1.55E-05	4.62E-01	-1.11E+00	-2.16E+00	5.44E+00	1.18E+01
EPHA2	1.12E+03	1.06E-05	2.47E-05	7.08E-01	-4.98E-01	-1.41E+00	4.97E+01	7.02E+01
EPHB4	2.31E+03	5.27E-11	4.67E-09	6.21E-01	-6.86E-01	-1.61E+00	9.55E+01	1.54E+02

EPHX2	1.27E+03	8.88E-04	1.15E-03	6.88E-01	-5.41E-01	-1.45E+00	5.56E+01	8.08E+01
EPO	1.08E+01	2.07E-05	4.36E-05	2.80E-01	-1.84E+00	-3.57E+00	2.62E-01	9.38E-01
EPOR	1.70E+02	5.68E-07	2.42E-06	4.29E-01	-1.22E+00	-2.33E+00	5.54E+00	1.29E+01
ERF	1.42E+03	1.47E-10	7.92E-09	5.37E-01	-8.97E-01	-1.86E+00	5.40E+01	1.01E+02
ERI3	9.01E+02	1.57E-05	3.44E-05	8.35E-01	-2.60E-01	-1.20E+00	4.36E+01	5.22E+01
ERICH2	4.59E+01	2.37E-03	2.83E-03	3.96E-01	-1.34E+00	-2.53E+00	1.38E+00	3.48E+00
ESPN	4.31E+02	3.91E-09	6.58E-08	2.40E-01	-2.06E+00	-4.17E+00	9.28E+00	3.86E+01
ESRRA	3.80E+02	1.19E-09	3.02E-08	4.85E-01	-1.04E+00	-2.06E+00	1.35E+01	2.79E+01
ESRRB	3.15E+01	8.93E-06	2.16E-05	1.31E-02	-6.26E+00	-7.66E+01	4.84E-02	3.71E+00
ETFA	1.00E+03	1.11E-09	2.99E-08	5.35E-01	-9.02E-01	-1.87E+00	3.80E+01	7.10E+01
ETFB	1.87E+03	2.52E-07	1.30E-06	6.33E-01	-6.60E-01	-1.58E+00	7.79E+01	1.23E+02
ETNK2	7.92E+03	4.61E-08	3.72E-07	4.73E-01	-1.08E+00	-2.11E+00	2.76E+02	5.84E+02
ETV4	5.84E+02	7.11E-11	5.30E-09	8.11E-02	-3.62E+00	-1.23E+01	5.13E+00	6.32E+01
EVA1A	5.59E+01	2.90E-03	3.41E-03	7.00E-01	-5.15E-01	-1.43E+00	2.46E+00	3.51E+00
EVL	6.12E+03	1.00E-09	2.86E-08	6.39E-01	-6.45E-01	-1.56E+00	2.57E+02	4.02E+02
EVX1	6.73E+01	6.61E-04	8.87E-04	4.08E-02	-4.62E+00	-2.45E+01	3.06E-01	7.51E+00
EXO5	1.55E+02	2.23E-03	2.68E-03	8.03E-01	-3.17E-01	-1.25E+00	7.37E+00	9.19E+00
EXOC3L1	8.13E+02	4.58E-07	2.05E-06	4.56E-01	-1.13E+00	-2.19E+00	2.78E+01	6.10E+01
EXOC7	1.77E+03	3.53E-04	5.08E-04	8.42E-01	-2.47E-01	-1.19E+00	8.55E+01	1.02E+02
EXOSC4	4.79E+02	5.29E-06	1.39E-05	7.13E-01	-4.88E-01	-1.40E+00	2.13E+01	2.98E+01
EXOSC5	2.11E+02	2.76E-05	5.57E-05	5.97E-01	-7.44E-01	-1.67E+00	8.48E+00	1.42E+01
EXOSC6	3.70E+02	7.34E-04	9.71E-04	7.52E-01	-4.10E-01	-1.33E+00	1.70E+01	2.27E+01
EXTL1	1.99E+02	4.50E-07	2.02E-06	2.72E-01	-1.88E+00	-3.68E+00	4.72E+00	1.74E+01
F11	1.65E+01	5.81E-04	7.90E-04	3.93E-01	-1.35E+00	-2.55E+00	5.17E-01	1.31E+00
F11R	1.96E+03	3.55E-10	1.40E-08	4.71E-01	-1.09E+00	-2.12E+00	6.86E+01	1.46E+02
FAAP100	5.99E+02	5.87E-10	2.01E-08	6.03E-01	-7.31E-01	-1.66E+00	2.42E+01	4.02E+01
FAAP20	4.51E+02	6.61E-08	4.88E-07	6.59E-01	-6.02E-01	-1.52E+00	1.92E+01	2.91E+01
FABP7	2.59E+02	1.26E-07	7.96E-07	3.11E-01	-1.69E+00	-3.22E+00	6.94E+00	2.23E+01
FADD	4.13E+02	1.87E-02	1.96E-02	8.40E-01	-2.51E-01	-1.19E+00	2.00E+01	2.38E+01
FAIM	1.98E+02	2.30E-05	4.76E-05	7.28E-01	-4.59E-01	-1.37E+00	8.85E+00	1.22E+01
FAM107B	5.42E+02	5.76E-04	7.84E-04	7.09E-01	-4.96E-01	-1.41E+00	2.41E+01	3.40E+01
FAM110A	1.29E+02	5.04E-03	5.65E-03	7.64E-01	-3.88E-01	-1.31E+00	6.01E+00	7.86E+00
FAM131B	2.78E+01	2.72E-06	8.04E-06	3.98E-01	-1.33E+00	-2.51E+00	8.71E-01	2.19E+00

FAM19A5	2.96E+01	2.48E-08	2.27E-07	1.63E-01	-2.62E+00	-6.13E+00	4.66E-01	2.85E+00
FAM212A	3.18E+02	2.40E-03	2.87E-03	7.52E-01	-4.11E-01	-1.33E+00	1.46E+01	1.94E+01
FAM214B	5.65E+02	5.93E-06	1.55E-05	6.53E-01	-6.15E-01	-1.53E+00	2.39E+01	3.66E+01
FAM217A	4.53E+01	1.34E-05	3.00E-05	2.08E-01	-2.27E+00	-4.82E+00	8.72E-01	4.20E+00
FAM227A	2.53E+01	6.55E-04	8.80E-04	3.56E-01	-1.49E+00	-2.81E+00	7.22E-01	2.03E+00
FAM241B	3.72E+02	4.43E-10	1.62E-08	6.26E-01	-6.76E-01	-1.60E+00	1.54E+01	2.46E+01
FAM47E	2.00E+02	9.51E-03	1.03E-02	7.30E-01	-4.54E-01	-1.37E+00	9.04E+00	1.24E+01
FAM53A	1.39E+01	1.64E-06	5.33E-06	2.41E-01	-2.05E+00	-4.15E+00	3.02E-01	1.25E+00
FAM53B	1.91E+03	2.42E-05	4.98E-05	6.64E-01	-5.91E-01	-1.51E+00	8.24E+01	1.24E+02
FAM57A	6.16E+02	3.08E-04	4.51E-04	7.72E-01	-3.73E-01	-1.29E+00	2.86E+01	3.70E+01
FAM71F1	6.28E+00	1.66E-05	3.61E-05	2.02E-01	-2.31E+00	-4.95E+00	1.21E-01	5.96E-01
FAM89B	7.43E+02	2.46E-04	3.71E-04	8.16E-01	-2.93E-01	-1.23E+00	3.54E+01	4.34E+01
FAR2	1.56E+02	2.00E-05	4.22E-05	5.48E-01	-8.69E-01	-1.83E+00	5.98E+00	1.09E+01
FARS2	4.78E+02	4.51E-06	1.23E-05	7.44E-01	-4.27E-01	-1.34E+00	2.18E+01	2.93E+01
FASLG	2.75E+02	1.77E-07	1.01E-06	2.03E-01	-2.30E+00	-4.93E+00	5.23E+00	2.58E+01
FASN	7.56E+03	8.05E-05	1.42E-04	6.12E-01	-7.08E-01	-1.63E+00	3.09E+02	5.05E+02
FASTK	1.10E+03	2.31E-08	2.17E-07	7.19E-01	-4.76E-01	-1.39E+00	4.91E+01	6.83E+01
FBLN2	4.92E+03	1.11E-08	1.25E-07	2.31E-01	-2.12E+00	-4.34E+00	1.04E+02	4.50E+02
FBN3	6.42E+03	3.11E-07	1.52E-06	2.36E-01	-2.09E+00	-4.25E+00	1.37E+02	5.81E+02
FBXL15	2.45E+02	1.24E-05	2.84E-05	7.39E-01	-4.36E-01	-1.35E+00	1.11E+01	1.50E+01
FBXL19	1.16E+03	1.44E-06	4.80E-06	5.99E-01	-7.39E-01	-1.67E+00	4.69E+01	7.83E+01
FBXL21	9.91E+01	3.89E-04	5.55E-04	6.42E-01	-6.40E-01	-1.56E+00	4.16E+00	6.48E+00
FBXL8	2.41E+02	1.21E-02	1.28E-02	8.18E-01	-2.91E-01	-1.22E+00	1.15E+01	1.40E+01
FCRL3	3.28E+00	5.67E-03	6.29E-03	1.61E-01	-2.64E+00	-6.21E+00	5.06E-02	3.14E-01
FDXR	1.33E+03	8.14E-03	8.85E-03	6.90E-01	-5.35E-01	-1.45E+00	5.85E+01	8.48E+01
FEM1A	2.20E+03	1.35E-08	1.44E-07	7.23E-01	-4.68E-01	-1.38E+00	9.85E+01	1.36E+02
FETUB	4.35E+00	1.94E-04	3.03E-04	2.75E-01	-1.86E+00	-3.64E+00	1.04E-01	3.76E-01
FEZ1	5.51E+02	2.91E-04	4.28E-04	6.38E-01	-6.48E-01	-1.57E+00	2.32E+01	3.63E+01
FGD1	1.56E+03	1.27E-07	8.01E-07	6.52E-01	-6.17E-01	-1.53E+00	6.60E+01	1.01E+02
FGF11	1.89E+03	4.23E-04	5.99E-04	5.52E-01	-8.58E-01	-1.81E+00	7.21E+01	1.31E+02
FGF19	1.07E+02	7.90E-11	5.43E-09	1.07E-02	-6.55E+00	-9.36E+01	1.33E-01	1.25E+01
FGFR3	4.79E+02	2.72E-11	2.79E-09	9.90E-02	-3.34E+00	-1.01E+01	4.97E+00	5.02E+01
FGL1	1.24E+02	2.21E-05	4.61E-05	4.77E-01	-1.07E+00	-2.10E+00	4.32E+00	9.06E+00

FH	1.06E+03	6.37E-09	8.58E-08	7.09E-01	-4.95E-01	-1.41E+00	4.71E+01	6.64E+01
FITM1	8.87E+01	1.11E-04	1.86E-04	6.66E-01	-5.86E-01	-1.50E+00	3.78E+00	5.68E+00
FMNL1	3.45E+02	5.92E-03	6.55E-03	8.07E-01	-3.10E-01	-1.24E+00	1.64E+01	2.03E+01
FOLR1	1.36E+01	2.62E-04	3.93E-04	6.26E-02	-4.00E+00	-1.60E+01	9.35E-02	1.50E+00
FOXH1	4.74E+01	2.20E-08	2.10E-07	4.28E-02	-4.55E+00	-2.34E+01	2.30E-01	5.37E+00
FOXO6	4.32E+02	6.88E-08	5.04E-07	3.33E-01	-1.59E+00	-3.01E+00	1.20E+01	3.60E+01
FPGS	7.17E+02	4.55E-09	7.02E-08	6.23E-01	-6.83E-01	-1.61E+00	2.97E+01	4.77E+01
FRAT2	2.62E+02	1.62E-08	1.67E-07	4.51E-01	-1.15E+00	-2.22E+00	8.96E+00	1.99E+01
FRMD8	6.51E+02	1.34E-03	1.67E-03	8.19E-01	-2.89E-01	-1.22E+00	3.12E+01	3.81E+01
FSCN1	7.51E+03	1.32E-05	2.97E-05	7.14E-01	-4.86E-01	-1.40E+00	3.34E+02	4.68E+02
FTH1	2.39E+04	1.14E-06	4.05E-06	7.41E-01	-4.32E-01	-1.35E+00	1.09E+03	1.47E+03
FUS	9.65E+03	3.06E-08	2.71E-07	7.01E-01	-5.12E-01	-1.43E+00	4.26E+02	6.07E+02
FUT2	1.46E+01	7.84E-04	1.03E-03	6.92E-02	-3.85E+00	-1.44E+01	1.07E-01	1.54E+00
FUZ	3.68E+02	5.59E-07	2.39E-06	5.76E-01	-7.95E-01	-1.73E+00	1.45E+01	2.51E+01
FXN	6.22E+02	4.76E-08	3.79E-07	6.67E-01	-5.84E-01	-1.50E+00	2.67E+01	4.01E+01
FZD10	2.50E+02	1.03E-05	2.42E-05	3.48E-01	-1.52E+00	-2.88E+00	7.16E+00	2.06E+01
FZD2	1.08E+03	1.45E-03	1.80E-03	5.69E-01	-8.14E-01	-1.76E+00	4.23E+01	7.44E+01
G6PC3	1.02E+03	9.15E-09	1.11E-07	6.87E-01	-5.41E-01	-1.46E+00	4.46E+01	6.49E+01
G6PD	4.05E+03	6.44E-08	4.83E-07	4.97E-01	-1.01E+00	-2.01E+00	1.45E+02	2.92E+02
GADL1	1.01E+01	2.65E-05	5.37E-05	2.77E-01	-1.85E+00	-3.60E+00	2.34E-01	8.42E-01
GAL3ST2	1.13E+01	9.05E-06	2.19E-05	2.24E-01	-2.16E+00	-4.46E+00	2.30E-01	1.03E+00
GALE	2.74E+02	1.32E-08	1.42E-07	5.68E-01	-8.15E-01	-1.76E+00	1.08E+01	1.89E+01
GALM	5.04E+02	4.40E-04	6.20E-04	8.02E-01	-3.18E-01	-1.25E+00	2.39E+01	2.98E+01
GALNT16	5.70E+02	1.22E-04	2.02E-04	5.99E-01	-7.39E-01	-1.67E+00	2.31E+01	3.86E+01
GALT	5.40E+02	1.03E-08	1.19E-07	5.94E-01	-7.51E-01	-1.68E+00	2.16E+01	3.64E+01
GAP43	1.08E+02	3.29E-03	3.82E-03	3.41E-01	-1.55E+00	-2.93E+00	3.10E+00	9.10E+00
GAPDH	1.55E+04	2.46E-06	7.38E-06	6.56E-01	-6.09E-01	-1.53E+00	6.62E+02	1.01E+03
GAREM1	4.11E+02	2.26E-05	4.70E-05	6.71E-01	-5.76E-01	-1.49E+00	1.77E+01	2.64E+01
GARS	1.20E+03	2.87E-09	5.34E-08	4.93E-01	-1.02E+00	-2.03E+00	4.33E+01	8.80E+01
GAS2L1	9.08E+02	7.98E-04	1.05E-03	7.68E-01	-3.81E-01	-1.30E+00	4.20E+01	5.47E+01
GATA3	1.19E+02	5.04E-10	1.78E-08	1.78E-01	-2.49E+00	-5.63E+00	2.03E+00	1.14E+01
GATA4	7.35E+03	2.85E-09	5.34E-08	5.28E-01	-9.23E-01	-1.90E+00	2.75E+02	5.22E+02
GATD1	7.07E+02	1.23E-03	1.56E-03	7.84E-01	-3.51E-01	-1.28E+00	3.33E+01	4.24E+01

GATD3A	8.98E+02	1.11E-05	2.56E-05	7.29E-01	-4.56E-01	-1.37E+00	4.04E+01	5.54E+01
GCAT	3.39E+02	2.76E-05	5.56E-05	6.30E-01	-6.66E-01	-1.59E+00	1.41E+01	2.24E+01
GCDH	9.78E+02	1.24E-06	4.33E-06	7.29E-01	-4.55E-01	-1.37E+00	4.41E+01	6.04E+01
GCHFR	1.29E+02	1.81E-02	1.90E-02	7.38E-01	-4.38E-01	-1.36E+00	5.86E+00	7.95E+00
GDAP1L1	1.70E+02	6.45E-08	4.83E-07	3.22E-01	-1.63E+00	-3.10E+00	4.58E+00	1.42E+01
GDPD2	8.52E+01	9.90E-06	2.35E-05	2.73E-01	-1.87E+00	-3.66E+00	2.05E+00	7.49E+00
GDPD5	3.95E+02	1.22E-05	2.80E-05	3.91E-01	-1.36E+00	-2.56E+00	1.22E+01	3.13E+01
GDPGP1	1.71E+02	1.72E-04	2.74E-04	7.30E-01	-4.53E-01	-1.37E+00	7.76E+00	1.06E+01
GEMIN7	1.80E+02	1.36E-08	1.45E-07	5.05E-01	-9.85E-01	-1.98E+00	6.58E+00	1.30E+01
GFER	5.20E+02	1.34E-06	4.55E-06	6.31E-01	-6.65E-01	-1.59E+00	2.16E+01	3.43E+01
GFRA1	2.83E+02	2.26E-07	1.21E-06	3.82E-01	-1.39E+00	-2.62E+00	8.65E+00	2.26E+01
GINS3	2.39E+02	1.72E-04	2.74E-04	7.65E-01	-3.87E-01	-1.31E+00	1.11E+01	1.45E+01
GJB1	2.85E+01	3.38E-03	3.91E-03	4.36E-01	-1.20E+00	-2.30E+00	9.27E-01	2.13E+00
GLDC	8.52E+02	2.93E-05	5.83E-05	4.61E-01	-1.12E+00	-2.17E+00	2.92E+01	6.34E+01
GLOD4	9.54E+02	5.53E-06	1.45E-05	8.02E-01	-3.19E-01	-1.25E+00	4.52E+01	5.64E+01
GLRX5	8.93E+02	1.62E-03	1.99E-03	7.76E-01	-3.65E-01	-1.29E+00	4.17E+01	5.37E+01
GLYATL3	4.25E+01	5.79E-07	2.45E-06	1.67E-01	-2.58E+00	-5.97E+00	7.00E-01	4.18E+00
GMDS	7.25E+02	1.38E-05	3.08E-05	7.40E-01	-4.33E-01	-1.35E+00	3.28E+01	4.43E+01
GNAI2	8.78E+03	5.49E-05	1.02E-04	8.21E-01	-2.84E-01	-1.22E+00	4.21E+02	5.13E+02
GNAT1	2.70E+02	3.40E-07	1.64E-06	4.06E-01	-1.30E+00	-2.46E+00	8.58E+00	2.11E+01
GNB1L	2.01E+03	1.84E-03	2.23E-03	7.87E-01	-3.45E-01	-1.27E+00	9.42E+01	1.20E+02
GNB2	4.44E+03	5.89E-05	1.08E-04	7.79E-01	-3.60E-01	-1.28E+00	2.08E+02	2.67E+02
GNG13	1.68E+01	7.16E-06	1.81E-05	1.95E-01	-2.36E+00	-5.12E+00	3.04E-01	1.56E+00
GNG3	5.73E+01	1.59E-07	9.36E-07	3.48E-01	-1.52E+00	-2.87E+00	1.63E+00	4.69E+00
GNG5	1.08E+03	2.36E-05	4.88E-05	7.26E-01	-4.62E-01	-1.38E+00	4.83E+01	6.65E+01
GNMT	1.35E+02	4.31E-07	1.96E-06	5.59E-01	-8.40E-01	-1.79E+00	5.23E+00	9.36E+00
GOT1	7.61E+02	1.10E-03	1.41E-03	6.73E-01	-5.71E-01	-1.49E+00	3.28E+01	4.88E+01
GOT2	2.51E+03	1.42E-03	1.78E-03	8.33E-01	-2.64E-01	-1.20E+00	1.21E+02	1.45E+02
GPANK1	1.42E+03	4.63E-05	8.83E-05	7.90E-01	-3.41E-01	-1.27E+00	6.67E+01	8.45E+01
GPC2	1.43E+02	6.09E-08	4.64E-07	2.36E-01	-2.08E+00	-4.23E+00	3.04E+00	1.29E+01
GPC3	2.90E+03	6.55E-07	2.66E-06	4.70E-01	-1.09E+00	-2.13E+00	1.01E+02	2.16E+02
GPR1	7.80E+01	3.38E-06	9.66E-06	1.71E-01	-2.55E+00	-5.85E+00	1.33E+00	7.81E+00
GPR108	8.46E+02	1.54E-04	2.48E-04	8.16E-01	-2.94E-01	-1.23E+00	4.01E+01	4.92E+01

GPR153	1.66E+03	2.14E-06	6.58E-06	6.40E-01	-6.44E-01	-1.56E+00	6.92E+01	1.08E+02
GPR162	2.52E+02	2.10E-06	6.49E-06	5.85E-01	-7.72E-01	-1.71E+00	9.99E+00	1.71E+01
GPR3	1.21E+01	2.20E-04	3.37E-04	4.33E-01	-1.21E+00	-2.31E+00	3.98E-01	9.20E-01
GPR45	8.30E+01	4.13E-04	5.86E-04	6.84E-01	-5.49E-01	-1.46E+00	3.61E+00	5.28E+00
GPR61	6.66E+00	1.32E-03	1.66E-03	3.74E-01	-1.42E+00	-2.68E+00	1.92E-01	5.14E-01
GPR85	1.16E+01	6.13E-02	6.19E-02	5.85E-01	-7.73E-01	-1.71E+00	4.71E-01	8.05E-01
GPSM1	6.86E+03	8.32E-06	2.03E-05	6.82E-01	-5.52E-01	-1.47E+00	2.98E+02	4.38E+02
GPT	3.58E+02	4.27E-04	6.04E-04	6.49E-01	-6.24E-01	-1.54E+00	1.51E+01	2.33E+01
GRAMD1A	1.95E+03	5.64E-07	2.41E-06	7.37E-01	-4.40E-01	-1.36E+00	8.89E+01	1.21E+02
GRAMD4	9.03E+02	2.53E-04	3.80E-04	7.57E-01	-4.03E-01	-1.32E+00	4.16E+01	5.50E+01
GRIK5	1.06E+03	4.77E-04	6.65E-04	7.90E-01	-3.40E-01	-1.27E+00	4.99E+01	6.31E+01
GRIN1	2.85E+01	3.22E-04	4.69E-04	4.89E-01	-1.03E+00	-2.04E+00	9.94E-01	2.03E+00
GRK2	1.44E+03	8.54E-05	1.49E-04	8.42E-01	-2.48E-01	-1.19E+00	6.98E+01	8.29E+01
GRN	2.55E+03	2.03E-06	6.33E-06	7.89E-01	-3.42E-01	-1.27E+00	1.20E+02	1.52E+02
GRPEL1	9.06E+02	1.41E-05	3.13E-05	6.94E-01	-5.28E-01	-1.44E+00	3.97E+01	5.73E+01
GSS	3.82E+02	5.28E-05	9.83E-05	6.98E-01	-5.18E-01	-1.43E+00	1.69E+01	2.42E+01
GSTA4	1.35E+03	1.82E-04	2.87E-04	6.85E-01	-5.45E-01	-1.46E+00	5.90E+01	8.60E+01
GSTP1	1.33E+01	6.55E-08	4.88E-07	1.96E-01	-2.35E+00	-5.10E+00	2.50E-01	1.28E+00
GSTP1-2	2.82E+03	1.04E-07	6.94E-07	5.93E-01	-7.54E-01	-1.69E+00	1.14E+02	1.92E+02
GSTT1	1.20E+03	3.76E-10	1.43E-08	5.65E-01	-8.23E-01	-1.77E+00	4.69E+01	8.29E+01
GSTZ1	4.30E+02	1.69E-08	1.72E-07	6.29E-01	-6.68E-01	-1.59E+00	1.78E+01	2.83E+01
GTF2F1	1.73E+03	2.96E-06	8.59E-06	7.52E-01	-4.10E-01	-1.33E+00	7.92E+01	1.05E+02
GTF2H4	3.20E+02	1.76E-07	1.01E-06	6.85E-01	-5.46E-01	-1.46E+00	1.39E+01	2.03E+01
GTF2H5	3.62E+02	3.49E-03	4.03E-03	8.13E-01	-2.99E-01	-1.23E+00	1.73E+01	2.13E+01
GTF2IRD1	4.00E+02	3.20E-07	1.55E-06	5.82E-01	-7.82E-01	-1.72E+00	1.58E+01	2.72E+01
GTF3C2	1.37E+03	1.42E-04	2.31E-04	8.40E-01	-2.51E-01	-1.19E+00	6.64E+01	7.90E+01
GTPBP3	8.99E+02	8.88E-07	3.32E-06	7.67E-01	-3.83E-01	-1.30E+00	4.15E+01	5.42E+01
GUCA2A	1.70E+01	4.44E-07	2.00E-06	5.88E-02	-4.09E+00	-1.70E+01	1.11E-01	1.89E+00
GUCD1	1.07E+03	6.67E-07	2.69E-06	6.63E-01	-5.94E-01	-1.51E+00	4.57E+01	6.90E+01
GUK1	1.84E+03	3.71E-06	1.05E-05	7.24E-01	-4.65E-01	-1.38E+00	8.25E+01	1.14E+02
GYS1	9.24E+02	2.92E-06	8.53E-06	7.30E-01	-4.53E-01	-1.37E+00	4.16E+01	5.69E+01
H1FX	6.39E+03	2.58E-05	5.25E-05	7.78E-01	-3.63E-01	-1.29E+00	2.98E+02	3.84E+02
H2AFX	6.96E+02	4.51E-03	5.10E-03	7.45E-01	-4.26E-01	-1.34E+00	3.19E+01	4.29E+01

H2AFY	3.21E+03	7.33E-06	1.84E-05	7.70E-01	-3.77E-01	-1.30E+00	1.49E+02	1.93E+02
H2AFY2	1.34E+03	2.04E-07	1.12E-06	4.70E-01	-1.09E+00	-2.13E+00	4.69E+01	9.97E+01
H2AFZ	8.70E+02	2.59E-04	3.89E-04	6.16E-01	-6.98E-01	-1.62E+00	3.57E+01	5.79E+01
H3F3A	8.42E+03	7.90E-09	9.76E-08	5.17E-01	-9.53E-01	-1.94E+00	3.11E+02	6.02E+02
H3F3C	1.57E+02	9.25E-06	2.22E-05	6.84E-01	-5.47E-01	-1.46E+00	6.80E+00	9.94E+00
H4	2.44E+01	9.33E-06	2.23E-05	4.55E-01	-1.14E+00	-2.20E+00	8.34E-01	1.83E+00
HADH	1.84E+03	1.88E-05	4.02E-05	8.08E-01	-3.07E-01	-1.24E+00	8.75E+01	1.08E+02
HAGHL	3.50E+02	8.03E-07	3.10E-06	6.97E-01	-5.20E-01	-1.43E+00	1.54E+01	2.21E+01
HARS2	6.20E+02	8.25E-04	1.08E-03	7.92E-01	-3.37E-01	-1.26E+00	2.89E+01	3.65E+01
HBA	4.92E+03	3.15E-03	3.68E-03	3.62E-01	-1.47E+00	-2.76E+00	1.43E+02	3.95E+02
HBA1	4.72E+02	3.37E-03	3.90E-03	3.67E-01	-1.45E+00	-2.73E+00	1.38E+01	3.77E+01
HBG	2.49E+03	3.12E-03	3.64E-03	3.37E-01	-1.57E+00	-2.97E+00	6.88E+01	2.05E+02
HBM	5.44E+00	5.95E-03	6.58E-03	1.79E-01	-2.48E+00	-5.57E+00	9.48E-02	5.28E-01
HCRT	2.45E+00	1.08E-03	1.38E-03	2.12E-01	-2.24E+00	-4.72E+00	4.92E-02	2.32E-01
HDAC3	1.10E+03	1.45E-07	8.76E-07	7.51E-01	-4.14E-01	-1.33E+00	5.01E+01	6.68E+01
HDAC5	3.45E+03	2.57E-07	1.32E-06	7.68E-01	-3.81E-01	-1.30E+00	1.60E+02	2.08E+02
HDDC3	2.80E+02	1.33E-02	1.41E-02	8.20E-01	-2.86E-01	-1.22E+00	1.34E+01	1.64E+01
HDGF	4.08E+03	2.55E-09	5.03E-08	6.65E-01	-5.88E-01	-1.50E+00	1.75E+02	2.63E+02
HESX1	9.52E+01	2.94E-04	4.32E-04	6.87E-01	-5.42E-01	-1.46E+00	4.10E+00	5.96E+00
HEXDC	1.23E+03	3.12E-05	6.18E-05	7.06E-01	-5.03E-01	-1.42E+00	5.44E+01	7.71E+01
HEXIM1	1.87E+03	1.13E-02	1.21E-02	8.55E-01	-2.25E-01	-1.17E+00	9.15E+01	1.07E+02
HGFAC	2.86E+02	1.95E-04	3.04E-04	6.59E-01	-6.01E-01	-1.52E+00	1.22E+01	1.86E+01
HGH1	7.60E+02	3.30E-04	4.78E-04	7.74E-01	-3.69E-01	-1.29E+00	3.53E+01	4.56E+01
HHATL	8.19E+00	3.21E-03	3.73E-03	2.35E-01	-2.09E+00	-4.26E+00	1.69E-01	7.20E-01
HIBADH	1.20E+03	3.39E-08	2.96E-07	6.90E-01	-5.36E-01	-1.45E+00	5.23E+01	7.59E+01
HIGD2A	7.82E+02	5.64E-04	7.72E-04	7.59E-01	-3.98E-01	-1.32E+00	3.60E+01	4.74E+01
HIKESHI	4.10E+02	9.15E-06	2.21E-05	6.27E-01	-6.73E-01	-1.59E+00	1.70E+01	2.70E+01
HINT1	6.68E+02	2.90E-04	4.28E-04	7.40E-01	-4.35E-01	-1.35E+00	3.03E+01	4.10E+01
HJURP	6.17E+02	6.48E-05	1.17E-04	6.51E-01	-6.19E-01	-1.54E+00	2.60E+01	3.99E+01
HK1	5.65E+03	5.53E-06	1.45E-05	6.47E-01	-6.28E-01	-1.55E+00	2.38E+02	3.68E+02
HK2	4.70E+02	1.58E-03	1.95E-03	5.36E-01	-9.00E-01	-1.87E+00	1.80E+01	3.36E+01
HM13	1.32E+03	5.09E-03	5.70E-03	8.70E-01	-2.01E-01	-1.15E+00	6.51E+01	7.49E+01
HMGA2	6.85E+01	1.39E-05	3.10E-05	2.81E-01	-1.83E+00	-3.56E+00	1.66E+00	5.91E+00

HMGN1	4.04E+03	4.36E-06	1.19E-05	7.21E-01	-4.72E-01	-1.39E+00	1.81E+02	2.52E+02
HMGN3	1.73E+03	5.24E-08	4.04E-07	5.59E-01	-8.40E-01	-1.79E+00	6.71E+01	1.20E+02
HMOX1	1.74E+03	3.03E-03	3.55E-03	6.41E-01	-6.41E-01	-1.56E+00	7.42E+01	1.16E+02
HNF1A	4.43E+00	2.34E-06	7.08E-06	1.72E-01	-2.54E+00	-5.81E+00	7.20E-02	4.18E-01
HNF4A	1.31E+02	2.03E-06	6.33E-06	3.72E-03	-8.07E+00	-2.69E+02	5.86E-02	1.57E+01
HNRNPA0	4.69E+03	1.82E-12	5.81E-10	5.61E-01	-8.33E-01	-1.78E+00	1.82E+02	3.25E+02
HNRNPA1	1.70E+04	1.13E-05	2.61E-05	7.49E-01	-4.17E-01	-1.34E+00	7.79E+02	1.04E+03
HNRNPAB	4.13E+03	1.77E-10	8.85E-09	4.79E-01	-1.06E+00	-2.09E+00	1.45E+02	3.03E+02
HNRNPD	7.22E+03	2.49E-07	1.29E-06	7.43E-01	-4.29E-01	-1.35E+00	3.29E+02	4.42E+02
HOMER3	5.86E+02	1.56E-05	3.42E-05	7.20E-01	-4.74E-01	-1.39E+00	2.63E+01	3.65E+01
HOXA1	5.88E+01	2.72E-04	4.06E-04	5.31E-02	-4.23E+00	-1.88E+01	3.40E-01	6.41E+00
HOXC5	8.39E+01	3.66E-04	5.25E-04	5.00E-01	-1.00E+00	-2.00E+00	3.01E+00	6.02E+00
HPD	5.47E+00	1.27E-05	2.89E-05	3.31E-02	-4.92E+00	-3.02E+01	2.10E-02	6.36E-01
HPRT1	5.85E+02	2.09E-02	2.18E-02	7.97E-01	-3.28E-01	-1.25E+00	2.74E+01	3.44E+01
HPS6	2.06E+02	1.43E-07	8.66E-07	5.99E-01	-7.40E-01	-1.67E+00	8.29E+00	1.38E+01
HSD17B10	1.24E+03	1.41E-07	8.58E-07	5.73E-01	-8.02E-01	-1.74E+00	4.85E+01	8.46E+01
HSD17B14	6.93E+01	8.13E-06	2.00E-05	5.57E-01	-8.43E-01	-1.79E+00	2.68E+00	4.81E+00
HSD17B8	5.36E+02	1.31E-03	1.64E-03	7.87E-01	-3.46E-01	-1.27E+00	2.51E+01	3.19E+01
HSPBP1	6.40E+02	1.29E-04	2.13E-04	7.58E-01	-3.99E-01	-1.32E+00	2.95E+01	3.88E+01
HSPE1	2.47E+03	2.16E-06	6.64E-06	5.35E-01	-9.03E-01	-1.87E+00	9.29E+01	1.74E+02
HTRA2	6.68E+02	7.43E-06	1.86E-05	8.24E-01	-2.80E-01	-1.21E+00	3.19E+01	3.88E+01
HYAL2	1.26E+03	1.92E-08	1.92E-07	6.48E-01	-6.27E-01	-1.54E+00	5.30E+01	8.18E+01
IAH1	5.73E+02	1.01E-04	1.72E-04	7.31E-01	-4.52E-01	-1.37E+00	2.57E+01	3.51E+01
ICA1	5.19E+02	3.63E-05	7.09E-05	7.63E-01	-3.91E-01	-1.31E+00	2.40E+01	3.14E+01
ICAM5	1.89E+02	4.89E-06	1.31E-05	4.32E-01	-1.21E+00	-2.32E+00	6.25E+00	1.45E+01
IDH2	2.13E+03	8.76E-10	2.77E-08	5.94E-01	-7.51E-01	-1.68E+00	8.60E+01	1.45E+02
IDH3B	1.02E+03	1.90E-06	5.96E-06	7.33E-01	-4.48E-01	-1.36E+00	4.59E+01	6.27E+01
IFT122	1.25E+03	8.71E-05	1.52E-04	7.42E-01	-4.31E-01	-1.35E+00	5.62E+01	7.57E+01
IFT27	5.62E+02	8.10E-06	1.99E-05	7.32E-01	-4.51E-01	-1.37E+00	2.53E+01	3.46E+01
IFT46	6.44E+02	2.39E-02	2.48E-02	8.56E-01	-2.24E-01	-1.17E+00	3.15E+01	3.68E+01
IGBP1	1.01E+03	5.51E-04	7.56E-04	8.02E-01	-3.19E-01	-1.25E+00	4.78E+01	5.97E+01
IGF2	5.84E+04	2.18E-03	2.63E-03	7.95E-01	-3.32E-01	-1.26E+00	2.74E+03	3.45E+03
IGSF21	2.85E+01	1.67E-03	2.05E-03	9.17E-02	-3.45E+00	-1.09E+01	2.75E-01	2.99E+00

IGSF8	1.73E+03	1.10E-08	1.25E-07	5.70E-01	-8.11E-01	-1.75E+00	6.83E+01	1.20E+02
IGSF9	5.63E+02	2.69E-07	1.36E-06	3.14E-01	-1.67E+00	-3.19E+00	1.49E+01	4.76E+01
IL11RA	2.09E+03	2.08E-04	3.21E-04	5.79E-01	-7.88E-01	-1.73E+00	8.26E+01	1.43E+02
IL17RA	8.66E+02	1.81E-04	2.86E-04	7.32E-01	-4.51E-01	-1.37E+00	3.90E+01	5.34E+01
IL18BP	1.43E+01	6.28E-02	6.34E-02	2.49E-01	-2.01E+00	-4.02E+00	3.25E-01	1.31E+00
IL23A	1.35E+02	1.59E-07	9.36E-07	5.99E-01	-7.39E-01	-1.67E+00	5.41E+00	9.03E+00
IL27RA	1.22E+03	1.33E-04	2.17E-04	6.50E-01	-6.22E-01	-1.54E+00	5.17E+01	7.95E+01
ILVBL	1.10E+03	2.92E-08	2.61E-07	5.53E-01	-8.55E-01	-1.81E+00	4.26E+01	7.71E+01
IMP4	6.68E+02	3.56E-07	1.69E-06	6.67E-01	-5.84E-01	-1.50E+00	2.88E+01	4.31E+01
IMPDH2	2.14E+03	5.61E-05	1.03E-04	7.64E-01	-3.89E-01	-1.31E+00	9.88E+01	1.29E+02
INAVA	4.59E+01	6.32E-09	8.58E-08	1.14E-01	-3.13E+00	-8.78E+00	5.36E-01	4.70E+00
ING1	7.11E+02	1.36E-05	3.05E-05	7.26E-01	-4.62E-01	-1.38E+00	3.19E+01	4.39E+01
INO80B	8.10E+02	2.19E-07	1.19E-06	6.93E-01	-5.29E-01	-1.44E+00	3.54E+01	5.11E+01
INSRR	1.89E+02	1.59E-06	5.23E-06	4.80E-01	-1.06E+00	-2.08E+00	6.68E+00	1.39E+01
IP6K2	1.51E+03	3.37E-09	5.95E-08	6.68E-01	-5.82E-01	-1.50E+00	6.45E+01	9.65E+01
IQCG	1.11E+02	5.91E-04	8.02E-04	4.09E-01	-1.29E+00	-2.45E+00	3.39E+00	8.29E+00
IQGAP3	5.39E+02	3.75E-03	4.31E-03	7.03E-01	-5.08E-01	-1.42E+00	2.39E+01	3.40E+01
IQSEC2	1.36E+03	2.44E-03	2.92E-03	7.02E-01	-5.10E-01	-1.42E+00	5.97E+01	8.50E+01
IRAK1	1.38E+03	2.68E-03	3.17E-03	8.34E-01	-2.62E-01	-1.20E+00	6.67E+01	8.00E+01
IRF2BP1	1.47E+03	3.28E-02	3.38E-02	8.78E-01	-1.87E-01	-1.14E+00	7.31E+01	8.33E+01
IRX1	1.16E+02	9.23E-06	2.22E-05	7.91E-03	-6.98E+00	-1.26E+02	1.08E-01	1.37E+01
IRX3	3.20E+02	1.46E-07	8.76E-07	3.46E-01	-1.53E+00	-2.89E+00	8.98E+00	2.60E+01
ISG20L2	7.27E+02	1.68E-06	5.44E-06	7.57E-01	-4.02E-01	-1.32E+00	3.34E+01	4.41E+01
ISOC2	2.34E+02	1.07E-04	1.80E-04	6.46E-01	-6.30E-01	-1.55E+00	9.81E+00	1.52E+01
ISX	6.60E-01	8.68E-03	9.41E-03	1.21E-03	-9.69E+00	-8.24E+02	1.00E-04	8.24E-02
ISY1	5.92E+02	2.04E-07	1.12E-06	7.57E-01	-4.02E-01	-1.32E+00	2.72E+01	3.59E+01
ITGAE	5.27E+02	5.69E-04	7.77E-04	8.39E-01	-2.54E-01	-1.19E+00	2.55E+01	3.05E+01
ITGB1BP1	8.28E+02	4.07E-03	4.66E-03	8.62E-01	-2.14E-01	-1.16E+00	4.05E+01	4.70E+01
ITM2C	7.84E+03	2.08E-04	3.22E-04	7.98E-01	-3.26E-01	-1.25E+00	3.70E+02	4.64E+02
ITPA	4.59E+02	4.85E-05	9.19E-05	7.49E-01	-4.17E-01	-1.34E+00	2.09E+01	2.80E+01
ITPK1	1.01E+03	4.13E-03	4.72E-03	7.46E-01	-4.22E-01	-1.34E+00	4.67E+01	6.26E+01
JKAMP	8.05E+02	1.81E-04	2.86E-04	7.20E-01	-4.73E-01	-1.39E+00	3.62E+01	5.02E+01
JPT1	6.20E+02	4.18E-07	1.90E-06	5.37E-01	-8.96E-01	-1.86E+00	2.34E+01	4.35E+01

JTB	6.47E+02	4.32E-04	6.09E-04	8.52E-01	-2.31E-01	-1.17E+00	3.15E+01	3.70E+01
KANK4	1.46E+03	8.51E-05	1.49E-04	3.37E-01	-1.57E+00	-2.97E+00	4.02E+01	1.19E+02
KAT8	1.38E+03	1.95E-03	2.37E-03	9.04E-01	-1.46E-01	-1.11E+00	6.91E+01	7.64E+01
KCNA2	1.33E+01	2.66E-02	2.75E-02	5.65E-01	-8.23E-01	-1.77E+00	5.22E-01	9.24E-01
KCNAB3	4.29E+01	3.38E-04	4.89E-04	4.92E-01	-1.02E+00	-2.03E+00	1.50E+00	3.05E+00
KCND1	6.05E+02	8.10E-05	1.43E-04	7.65E-01	-3.86E-01	-1.31E+00	2.80E+01	3.66E+01
KCNG1	4.96E+01	1.06E-04	1.79E-04	4.53E-01	-1.14E+00	-2.21E+00	1.69E+00	3.74E+00
KCNH2	4.29E+03	2.18E-06	6.66E-06	5.52E-01	-8.57E-01	-1.81E+00	1.65E+02	2.99E+02
KCNJ5	4.02E+01	1.89E-06	5.95E-06	7.96E-02	-3.65E+00	-1.26E+01	3.48E-01	4.37E+00
KCNK1	1.95E+02	4.42E-11	4.32E-09	1.44E-01	-2.79E+00	-6.92E+00	2.79E+00	1.93E+01
KCNK15	1.44E+01	6.90E-04	9.23E-04	9.74E-02	-3.36E+00	-1.03E+01	1.45E-01	1.49E+00
KCNK5	1.23E+02	1.65E-04	2.65E-04	4.62E-01	-1.11E+00	-2.17E+00	4.20E+00	9.10E+00
KCNN1	1.49E+02	4.94E-03	5.55E-03	5.99E-01	-7.38E-01	-1.67E+00	6.03E+00	1.01E+01
KCNN4	8.00E+01	6.20E-04	8.37E-04	3.95E-01	-1.34E+00	-2.53E+00	2.48E+00	6.29E+00
KCTD1	8.07E+02	3.82E-08	3.24E-07	3.61E-01	-1.47E+00	-2.77E+00	2.36E+01	6.53E+01
KCTD17	9.10E+02	2.40E-09	4.88E-08	5.10E-01	-9.73E-01	-1.96E+00	3.35E+01	6.58E+01
KHNYN	1.67E+03	2.93E-07	1.45E-06	7.80E-01	-3.59E-01	-1.28E+00	7.81E+01	1.00E+02
KIAA0895L	1.26E+02	5.99E-04	8.11E-04	6.15E-01	-7.01E-01	-1.63E+00	5.13E+00	8.34E+00
KIAA1522	2.50E+03	5.87E-07	2.47E-06	5.13E-01	-9.63E-01	-1.95E+00	9.13E+01	1.78E+02
KIF17	2.61E+02	1.49E-03	1.85E-03	6.54E-01	-6.12E-01	-1.53E+00	1.10E+01	1.68E+01
KIF21B	3.07E+03	8.62E-04	1.12E-03	4.92E-01	-1.02E+00	-2.03E+00	1.11E+02	2.26E+02
KISS1	4.35E+01	1.37E-06	4.61E-06	8.66E-02	-3.53E+00	-1.16E+01	3.99E-01	4.61E+00
KLC2	2.69E+03	3.07E-10	1.28E-08	6.20E-01	-6.89E-01	-1.61E+00	1.11E+02	1.79E+02
KLF16	8.75E+02	7.17E-04	9.52E-04	7.75E-01	-3.69E-01	-1.29E+00	4.07E+01	5.26E+01
KLF17	3.68E+01	8.01E-06	1.97E-05	1.11E-02	-6.49E+00	-9.00E+01	4.80E-02	4.32E+00
KLHDC8B	5.77E+02	7.33E-03	8.00E-03	7.91E-01	-3.38E-01	-1.26E+00	2.72E+01	3.44E+01
KLHL26	2.26E+02	8.44E-08	5.93E-07	6.68E-01	-5.83E-01	-1.50E+00	9.65E+00	1.44E+01
KLHL29	8.27E+01	3.75E-05	7.31E-05	4.31E-01	-1.21E+00	-2.32E+00	2.72E+00	6.31E+00
KLK1	4.83E+02	3.22E-10	1.30E-08	2.30E-01	-2.12E+00	-4.35E+00	9.94E+00	4.32E+01
KLK12	2.14E+02	3.19E-07	1.55E-06	1.66E-01	-2.59E+00	-6.03E+00	3.52E+00	2.12E+01
KLK6	1.94E+02	1.11E-06	3.98E-06	3.83E-03	-8.03E+00	-2.61E+02	8.91E-02	2.33E+01
KLK7	4.77E+01	1.44E-04	2.34E-04	2.74E-02	-5.19E+00	-3.64E+01	1.50E-01	5.48E+00
KLK8	2.20E+01	1.03E-05	2.42E-05	1.17E-01	-3.09E+00	-8.54E+00	2.69E-01	2.30E+00

KMT5C	1.90E+03	4.37E-08	3.57E-07	4.12E-01	-1.28E+00	-2.43E+00	6.00E+01	1.45E+02
KREMEN1	1.45E+03	3.29E-04	4.78E-04	5.78E-01	-7.90E-01	-1.73E+00	5.62E+01	9.72E+01
KREMEN2	5.09E+01	9.67E-08	6.61E-07	6.01E-02	-4.06E+00	-1.66E+01	3.35E-01	5.58E+00
KRT7	4.99E+02	4.17E-03	4.76E-03	2.35E-01	-2.09E+00	-4.25E+00	1.09E+01	4.65E+01
KRTCAP2	6.80E+02	2.67E-05	5.41E-05	7.66E-01	-3.84E-01	-1.30E+00	3.14E+01	4.09E+01
KY	2.15E+01	3.51E-04	5.06E-04	3.13E-01	-1.67E+00	-3.19E+00	5.62E-01	1.79E+00
KYAT3	2.69E+02	5.42E-03	6.05E-03	5.42E-01	-8.83E-01	-1.84E+00	1.02E+01	1.89E+01
L1CAM	7.30E+02	3.05E-09	5.59E-08	1.54E-01	-2.70E+00	-6.50E+00	1.11E+01	7.25E+01
L3HYPDH	1.92E+02	4.87E-08	3.87E-07	4.43E-01	-1.17E+00	-2.26E+00	6.50E+00	1.47E+01
LAMTOR4	8.66E+02	9.50E-04	1.22E-03	8.29E-01	-2.71E-01	-1.21E+00	4.17E+01	5.04E+01
LBHD2	7.22E+01	7.61E-06	1.89E-05	1.44E-01	-2.80E+00	-6.94E+00	1.05E+00	7.29E+00
LCK	1.30E+02	1.80E-04	2.85E-04	5.59E-01	-8.40E-01	-1.79E+00	5.02E+00	8.99E+00
LCNL1	2.56E+01	2.20E-04	3.37E-04	4.79E-01	-1.06E+00	-2.09E+00	9.03E-01	1.88E+00
LDB1	6.22E+03	2.16E-08	2.08E-07	6.26E-01	-6.77E-01	-1.60E+00	2.57E+02	4.11E+02
LDHA	1.42E+03	1.89E-04	2.96E-04	5.76E-01	-7.95E-01	-1.74E+00	5.65E+01	9.81E+01
LDLRAP1	1.13E+03	9.89E-04	1.27E-03	8.75E-01	-1.92E-01	-1.14E+00	5.57E+01	6.36E+01
LEF1	1.66E+03	1.51E-05	3.33E-05	3.78E-01	-1.41E+00	-2.65E+00	4.95E+01	1.31E+02
LFNG	2.25E+02	7.05E-04	9.37E-04	6.09E-01	-7.14E-01	-1.64E+00	9.24E+00	1.52E+01
LGALS3BP	2.19E+03	1.61E-03	1.98E-03	7.66E-01	-3.84E-01	-1.30E+00	1.01E+02	1.32E+02
LGI2	8.11E+02	3.03E-07	1.49E-06	2.42E-01	-2.05E+00	-4.14E+00	1.78E+01	7.34E+01
LHFPL1	5.46E+01	8.41E-07	3.20E-06	2.54E-01	-1.98E+00	-3.94E+00	1.23E+00	4.84E+00
LHPP	9.35E+02	5.43E-07	2.36E-06	6.35E-01	-6.56E-01	-1.58E+00	3.92E+01	6.18E+01
LHX2	1.74E+02	4.86E-05	9.19E-05	2.75E-01	-1.86E+00	-3.64E+00	4.17E+00	1.52E+01
LIMD2	4.31E+02	7.38E-07	2.93E-06	6.14E-01	-7.03E-01	-1.63E+00	1.76E+01	2.86E+01
LIMK1	9.50E+02	7.41E-08	5.31E-07	6.61E-01	-5.97E-01	-1.51E+00	4.05E+01	6.13E+01
LIN28A	3.24E+01	1.31E-05	2.96E-05	1.39E-02	-6.17E+00	-7.22E+01	5.17E-02	3.73E+00
LIPH	1.82E+01	1.88E-04	2.95E-04	5.19E-02	-4.27E+00	-1.93E+01	1.05E-01	2.03E+00
LIPT1	1.13E+02	2.11E-03	2.55E-03	7.65E-01	-3.87E-01	-1.31E+00	5.21E+00	6.82E+00
LLGL2	7.34E+02	4.94E-03	5.55E-03	7.48E-01	-4.19E-01	-1.34E+00	3.36E+01	4.49E+01
LMAN1L	3.86E+01	1.94E-04	3.02E-04	5.27E-02	-4.25E+00	-1.90E+01	2.31E-01	4.38E+00
LMNB1	1.31E+03	2.52E-06	7.52E-06	6.38E-01	-6.49E-01	-1.57E+00	5.49E+01	8.61E+01
LMNB2	2.50E+03	1.04E-06	3.78E-06	6.32E-01	-6.61E-01	-1.58E+00	1.04E+02	1.64E+02
LMNTD2	2.95E+01	3.08E-06	8.90E-06	1.63E-01	-2.61E+00	-6.12E+00	4.70E-01	2.88E+00

LOC100139325	2.15E+02	1.40E-09	3.35E-08	2.78E-01	-1.85E+00	-3.60E+00	5.23E+00	1.89E+01
LOC100140550	5.01E+01	1.15E-05	2.66E-05	1.05E-02	-6.57E+00	-9.49E+01	6.28E-02	5.96E+00
LOC100141025	1.71E+02	1.30E-05	2.94E-05	5.29E-01	-9.19E-01	-1.89E+00	6.43E+00	1.22E+01
LOC100141117	1.72E+01	1.13E-02	1.21E-02	6.86E-01	-5.44E-01	-1.46E+00	7.39E-01	1.08E+00
LOC100295842	8.87E+00	3.22E-10	1.30E-08	3.88E-04	-1.13E+01	-2.57E+03	4.21E-04	1.08E+00
LOC100297725	4.05E+03	2.88E-12	7.32E-10	4.57E-01	-1.13E+00	-2.19E+00	1.38E+02	3.03E+02
LOC100298871	8.10E+01	9.76E-03	1.05E-02	8.09E-01	-3.05E-01	-1.24E+00	3.86E+00	4.77E+00
LOC100300760	4.64E+03	6.13E-07	2.54E-06	2.36E-01	-2.09E+00	-4.25E+00	9.84E+01	4.18E+02
LOC100336682	9.94E-01	1.68E-03	2.06E-03	2.22E-02	-5.49E+00	-4.50E+01	2.66E-03	1.20E-01
LOC100336980	1.39E+00	6.41E-01	6.41E-01	1.60E-01	-2.64E+00	-6.25E+00	2.21E-02	1.38E-01
LOC100847700	2.81E+02	9.67E-04	1.24E-03	6.39E-01	-6.46E-01	-1.57E+00	1.18E+01	1.85E+01
LOC100848253	2.22E+01	9.87E-05	1.69E-04	4.35E-01	-1.20E+00	-2.30E+00	7.35E-01	1.69E+00
LOC100848325	7.31E+01	1.43E-03	1.78E-03	4.39E-01	-1.19E+00	-2.28E+00	2.40E+00	5.47E+00
LOC100848353	1.39E+03	1.49E-02	1.57E-02	8.58E-01	-2.22E-01	-1.17E+00	6.84E+01	7.97E+01
LOC100849068	9.25E+00	3.69E-04	5.28E-04	7.19E-02	-3.80E+00	-1.39E+01	7.29E-02	1.01E+00
LOC100850808	1.33E+01	1.10E-03	1.40E-03	9.37E-02	-3.42E+00	-1.07E+01	1.37E-01	1.46E+00
LOC101902029	1.18E+01	1.06E-02	1.13E-02	5.33E-01	-9.08E-01	-1.88E+00	4.38E-01	8.23E-01
LOC101902937	1.01E+03	6.95E-05	1.25E-04	6.75E-01	-5.66E-01	-1.48E+00	4.32E+01	6.40E+01
LOC101903140	1.35E+01	2.36E-03	2.82E-03	5.97E-01	-7.44E-01	-1.67E+00	5.45E-01	9.12E-01
LOC101903991	1.61E+01	4.45E-03	5.05E-03	4.15E-01	-1.27E+00	-2.41E+00	5.06E-01	1.22E+00
LOC101904187	1.39E+02	3.91E-07	1.81E-06	3.13E-01	-1.68E+00	-3.20E+00	3.69E+00	1.18E+01
LOC101904275	2.76E+02	1.31E-03	1.65E-03	8.43E-01	-2.46E-01	-1.19E+00	1.33E+01	1.58E+01
LOC101904314	5.60E+01	1.33E-02	1.42E-02	7.37E-01	-4.40E-01	-1.36E+00	2.50E+00	3.39E+00
LOC101904810	6.76E+01	1.98E-05	4.18E-05	4.41E-01	-1.18E+00	-2.27E+00	2.25E+00	5.11E+00
LOC101905156	5.12E+02	2.33E-07	1.23E-06	6.78E-01	-5.60E-01	-1.47E+00	2.22E+01	3.28E+01
LOC101905222	8.68E+01	2.34E-09	4.81E-08	1.24E-01	-3.02E+00	-8.09E+00	1.10E+00	8.89E+00
LOC101905514	8.08E+02	5.51E-05	1.02E-04	6.86E-01	-5.43E-01	-1.46E+00	3.54E+01	5.16E+01
LOC101906010	1.57E+02	1.11E-06	3.99E-06	5.57E-01	-8.44E-01	-1.80E+00	6.00E+00	1.08E+01
LOC101906273	7.09E+01	4.76E-06	1.28E-05	5.67E-01	-8.18E-01	-1.76E+00	2.77E+00	4.89E+00
LOC101906280	1.34E+02	1.44E-04	2.34E-04	6.49E-01	-6.23E-01	-1.54E+00	5.66E+00	8.72E+00
LOC101906855	3.81E+02	4.93E-06	1.31E-05	6.84E-01	-5.48E-01	-1.46E+00	1.65E+01	2.41E+01
LOC101906916	2.14E+03	1.85E-06	5.86E-06	6.73E-01	-5.72E-01	-1.49E+00	9.21E+01	1.37E+02
LOC101906939	8.25E+01	1.02E-05	2.41E-05	6.00E-01	-7.38E-01	-1.67E+00	3.33E+00	5.55E+00

LOC101907000	8.88E+01	5.89E-05	1.08E-04	5.82E-01	-7.81E-01	-1.72E+00	3.52E+00	6.05E+00
LOC101907022	2.46E+01	9.47E-03	1.02E-02	5.83E-01	-7.78E-01	-1.71E+00	9.95E-01	1.71E+00
LOC101907127	6.49E+00	1.27E-03	1.60E-03	1.17E-01	-3.09E+00	-8.51E+00	7.86E-02	6.69E-01
LOC101907157	4.53E+01	3.70E-05	7.22E-05	3.35E-01	-1.58E+00	-2.99E+00	1.22E+00	3.66E+00
LOC101907294	5.01E+01	8.08E-04	1.06E-03	6.71E-01	-5.76E-01	-1.49E+00	2.16E+00	3.22E+00
LOC101907557	6.94E+01	4.59E-05	8.78E-05	5.89E-01	-7.64E-01	-1.70E+00	2.76E+00	4.68E+00
LOC101907853	9.49E+00	1.95E-04	3.03E-04	4.00E-01	-1.32E+00	-2.50E+00	2.99E-01	7.48E-01
LOC101907940	1.99E+01	5.26E-06	1.39E-05	4.62E-01	-1.11E+00	-2.17E+00	6.85E-01	1.48E+00
LOC104968964	1.76E+02	2.01E-04	3.11E-04	7.23E-01	-4.68E-01	-1.38E+00	7.87E+00	1.09E+01
LOC104969026	5.08E+00	1.14E-03	1.46E-03	8.58E-02	-3.54E+00	-1.17E+01	4.67E-02	5.45E-01
LOC104969408	3.57E+01	5.15E-04	7.10E-04	5.26E-01	-9.28E-01	-1.90E+00	1.34E+00	2.54E+00
LOC104970173	7.07E+02	2.21E-06	6.73E-06	7.36E-01	-4.42E-01	-1.36E+00	3.19E+01	4.34E+01
LOC104970930	6.36E+01	2.04E-04	3.16E-04	2.55E-01	-1.97E+00	-3.92E+00	1.45E+00	5.69E+00
LOC104970966	1.28E+01	1.27E-02	1.35E-02	6.49E-01	-6.23E-01	-1.54E+00	5.43E-01	8.35E-01
LOC104971292	6.68E+01	2.81E-06	8.28E-06	5.26E-01	-9.26E-01	-1.90E+00	2.48E+00	4.71E+00
LOC104971855	6.59E+00	1.39E-02	1.48E-02	6.00E-01	-7.37E-01	-1.67E+00	2.60E-01	4.33E-01
LOC104973427	4.26E+01	4.49E-03	5.09E-03	7.31E-01	-4.51E-01	-1.37E+00	1.91E+00	2.61E+00
LOC104973517	3.13E+02	1.73E-04	2.76E-04	6.77E-01	-5.63E-01	-1.48E+00	1.36E+01	2.01E+01
LOC104974770	4.16E+01	6.31E-04	8.50E-04	4.35E-01	-1.20E+00	-2.30E+00	1.37E+00	3.15E+00
LOC104975196	1.60E+03	3.20E-06	9.21E-06	7.03E-01	-5.09E-01	-1.42E+00	7.05E+01	1.00E+02
LOC107131293	8.66E+01	1.57E-06	5.18E-06	5.36E-01	-8.99E-01	-1.86E+00	3.26E+00	6.07E+00
LOC107131324	1.09E+02	1.66E-03	2.04E-03	7.64E-01	-3.89E-01	-1.31E+00	5.04E+00	6.60E+00
LOC107131465	8.45E+00	6.93E-03	7.61E-03	1.54E-01	-2.70E+00	-6.49E+00	1.32E-01	8.59E-01
LOC107131695	3.51E+01	1.91E-05	4.07E-05	5.46E-01	-8.74E-01	-1.83E+00	1.32E+00	2.42E+00
LOC107131728	1.22E+03	8.45E-06	2.06E-05	6.50E-01	-6.23E-01	-1.54E+00	5.14E+01	7.91E+01
LOC107132067	9.53E+01	1.55E-06	5.11E-06	5.84E-01	-7.77E-01	-1.71E+00	3.79E+00	6.49E+00
LOC107132075	2.39E+01	5.11E-05	9.58E-05	3.97E-02	-4.65E+00	-2.52E+01	1.07E-01	2.68E+00
LOC107132205	1.14E+00	6.01E-02	6.08E-02	9.58E-02	-3.38E+00	-1.04E+01	1.19E-02	1.24E-01
LOC107132228	2.59E+02	6.15E-05	1.12E-04	7.18E-01	-4.79E-01	-1.39E+00	1.16E+01	1.61E+01
LOC107132243	4.10E+02	7.87E-08	5.60E-07	6.76E-01	-5.64E-01	-1.48E+00	1.77E+01	2.62E+01
LOC107132247	4.16E+02	4.48E-07	2.02E-06	6.76E-01	-5.64E-01	-1.48E+00	1.80E+01	2.66E+01
LOC107132450	2.04E+02	4.29E-04	6.06E-04	7.11E-01	-4.92E-01	-1.41E+00	9.02E+00	1.27E+01
LOC107132556	8.51E+01	6.87E-05	1.23E-04	5.76E-01	-7.95E-01	-1.74E+00	3.33E+00	5.79E+00

LOC107132734	4.57E+01	1.42E-03	1.77E-03	6.43E-01	-6.37E-01	-1.56E+00	1.91E+00	2.97E+00
LOC107132856	1.16E+02	2.26E-02	2.35E-02	8.10E-01	-3.03E-01	-1.23E+00	5.50E+00	6.78E+00
LOC112441531	1.47E+00	1.79E-05	3.86E-05	8.99E-02	-3.48E+00	-1.11E+01	1.40E-02	1.56E-01
LOC112441539	9.10E+00	1.48E-03	1.83E-03	9.33E-02	-3.42E+00	-1.07E+01	9.17E-02	9.83E-01
LOC112441980	1.01E+02	1.29E-05	2.92E-05	3.03E-01	-1.72E+00	-3.30E+00	2.55E+00	8.41E+00
LOC112442207	1.34E+02	5.21E-09	7.66E-08	8.89E-03	-6.81E+00	-1.13E+02	1.42E-01	1.60E+01
LOC112442246	2.02E+02	3.34E-03	3.88E-03	7.49E-01	-4.17E-01	-1.34E+00	9.29E+00	1.24E+01
LOC112442288	8.93E+01	8.37E-07	3.19E-06	5.99E-01	-7.39E-01	-1.67E+00	3.63E+00	6.05E+00
LOC112442368	2.50E+00	3.44E-06	9.82E-06	8.18E-02	-3.61E+00	-1.22E+01	2.15E-02	2.63E-01
LOC112442373	4.71E+01	3.08E-07	1.51E-06	1.16E-01	-3.10E+00	-8.59E+00	5.64E-01	4.85E+00
LOC112442982	3.25E+01	4.22E-06	1.16E-05	5.30E-01	-9.16E-01	-1.89E+00	1.21E+00	2.28E+00
LOC112443216	8.09E+02	1.16E-07	7.49E-07	5.66E-01	-8.22E-01	-1.77E+00	3.16E+01	5.58E+01
LOC112444532	2.82E+00	2.21E-02	2.30E-02	3.76E-01	-1.41E+00	-2.66E+00	8.22E-02	2.18E-01
LOC112444733	5.15E+00	4.24E-02	4.33E-02	3.44E-01	-1.54E+00	-2.91E+00	1.50E-01	4.35E-01
LOC112445078	9.87E+02	1.10E-05	2.56E-05	5.95E-01	-7.48E-01	-1.68E+00	3.96E+01	6.65E+01
LOC112445127	7.59E+01	1.30E-04	2.14E-04	2.07E-02	-5.60E+00	-4.84E+01	1.85E-01	8.97E+00
LOC112445996	2.58E+03	2.63E-04	3.93E-04	7.98E-01	-3.26E-01	-1.25E+00	1.22E+02	1.53E+02
LOC112446393	1.23E+02	8.29E-06	2.03E-05	1.46E-02	-6.09E+00	-6.83E+01	2.11E-01	1.44E+01
LOC112446406	1.63E+03	1.30E-07	8.15E-07	5.99E-01	-7.40E-01	-1.67E+00	6.58E+01	1.10E+02
LOC112446645	6.39E+02	3.79E-07	1.77E-06	6.27E-01	-6.73E-01	-1.59E+00	2.65E+01	4.23E+01
LOC112446738	1.15E+01	5.94E-02	6.02E-02	7.30E-01	-4.54E-01	-1.37E+00	5.20E-01	7.13E-01
LOC112447018	3.28E+00	3.23E-01	3.23E-01	2.09E-01	-2.26E+00	-4.79E+00	6.45E-02	3.09E-01
LOC112447610	2.46E+01	1.64E-03	2.01E-03	6.79E-02	-3.88E+00	-1.47E+01	1.80E-01	2.65E+00
LOC112449049	2.77E+00	4.86E-02	4.95E-02	4.21E-01	-1.25E+00	-2.38E+00	8.66E-02	2.06E-01
LOC407163	2.88E+01	1.84E-03	2.23E-03	1.12E-01	-3.16E+00	-8.94E+00	3.37E-01	3.02E+00
LOC504861	1.77E+01	1.66E-05	3.61E-05	1.25E-02	-6.33E+00	-8.03E+01	2.57E-02	2.06E+00
LOC505183	4.94E+00	1.18E-03	1.50E-03	3.14E-01	-1.67E+00	-3.18E+00	1.30E-01	4.14E-01
LOC505972	8.55E+00	1.16E-04	1.93E-04	4.05E-02	-4.63E+00	-2.47E+01	3.84E-02	9.49E-01
LOC508098	5.23E+02	7.01E-05	1.26E-04	2.77E-01	-1.85E+00	-3.61E+00	1.30E+01	4.69E+01
LOC508455	3.08E+01	3.05E-04	4.46E-04	5.38E-01	-8.94E-01	-1.86E+00	1.15E+00	2.13E+00
LOC509911	7.71E+00	3.66E-04	5.25E-04	7.74E-02	-3.69E+00	-1.29E+01	6.44E-02	8.32E-01
LOC511130	1.05E+02	1.09E-03	1.39E-03	7.02E-01	-5.11E-01	-1.42E+00	4.67E+00	6.66E+00
LOC511386	4.08E+02	3.24E-05	6.42E-05	7.26E-01	-4.62E-01	-1.38E+00	1.83E+01	2.53E+01

LOC512175	5.56E+01	6.68E-03	7.35E-03	7.51E-01	-4.13E-01	-1.33E+00	2.52E+00	3.35E+00
LOC512953	1.63E+03	1.29E-06	4.45E-06	6.70E-01	-5.77E-01	-1.49E+00	7.01E+01	1.05E+02
LOC513322	1.13E+01	3.68E-05	7.18E-05	4.33E-01	-1.21E+00	-2.31E+00	3.78E-01	8.73E-01
LOC513690	7.84E+01	5.62E-03	6.25E-03	1.72E-01	-2.54E+00	-5.82E+00	1.26E+00	7.31E+00
LOC515042	2.83E+03	4.22E-05	8.13E-05	7.60E-01	-3.96E-01	-1.32E+00	1.31E+02	1.72E+02
LOC516108	4.79E+02	2.33E-03	2.79E-03	7.35E-01	-4.44E-01	-1.36E+00	2.16E+01	2.94E+01
LOC516378	2.99E+00	7.09E-05	1.27E-04	5.15E-02	-4.28E+00	-1.94E+01	1.71E-02	3.33E-01
LOC518080	1.27E+01	3.24E-02	3.34E-02	4.96E-01	-1.01E+00	-2.02E+00	4.43E-01	8.95E-01
LOC518961	1.31E+01	2.39E-03	2.85E-03	5.45E-01	-8.76E-01	-1.83E+00	4.98E-01	9.14E-01
LOC521224	1.96E+03	4.40E-07	1.99E-06	6.18E-01	-6.93E-01	-1.62E+00	8.04E+01	1.30E+02
LOC526230	5.27E+00	1.15E-03	1.46E-03	8.85E-02	-3.50E+00	-1.13E+01	4.86E-02	5.49E-01
LOC526769	6.88E+01	2.63E-05	5.35E-05	4.98E-01	-1.01E+00	-2.01E+00	2.46E+00	4.94E+00
LOC527388	1.91E+01	1.76E-03	2.15E-03	5.23E-01	-9.36E-01	-1.91E+00	7.12E-01	1.36E+00
LOC529657	1.67E+01	8.71E-03	9.43E-03	3.33E-01	-1.59E+00	-3.01E+00	4.32E-01	1.30E+00
LOC529823	1.37E+01	2.75E-04	4.09E-04	3.60E-01	-1.48E+00	-2.78E+00	3.92E-01	1.09E+00
LOC533307	5.34E+03	2.47E-07	1.29E-06	6.61E-01	-5.97E-01	-1.51E+00	2.29E+02	3.46E+02
LOC534391	2.11E+03	5.91E-03	6.54E-03	8.58E-01	-2.20E-01	-1.16E+00	1.04E+02	1.21E+02
LOC534967	1.33E+02	3.34E-06	9.59E-06	2.00E-01	-2.32E+00	-4.99E+00	2.55E+00	1.27E+01
LOC539705	6.90E+01	1.74E-10	8.85E-09	2.60E-02	-5.27E+00	-3.85E+01	2.06E-01	7.92E+00
LOC613345	5.94E+00	2.13E-03	2.57E-03	4.68E-01	-1.09E+00	-2.14E+00	2.05E-01	4.38E-01
LOC613364	2.88E+00	6.17E-02	6.23E-02	5.31E-01	-9.14E-01	-1.88E+00	1.09E-01	2.06E-01
LOC613401	8.30E+01	6.04E-06	1.56E-05	5.56E-01	-8.48E-01	-1.80E+00	3.20E+00	5.76E+00
LOC613660	1.63E+02	5.12E-03	5.73E-03	7.97E-01	-3.27E-01	-1.25E+00	7.72E+00	9.69E+00
LOC614423	1.87E+02	3.55E-02	3.65E-02	8.59E-01	-2.20E-01	-1.16E+00	9.19E+00	1.07E+01
LOC614748	1.72E+02	7.27E-06	1.83E-05	5.31E-01	-9.14E-01	-1.88E+00	6.56E+00	1.24E+01
LOC614882	2.51E+01	6.44E-07	2.63E-06	3.95E-01	-1.34E+00	-2.53E+00	7.78E-01	1.97E+00
LOC614922	4.60E+03	8.25E-05	1.45E-04	8.01E-01	-3.21E-01	-1.25E+00	2.18E+02	2.73E+02
LOC615002	2.60E+02	1.12E-05	2.59E-05	6.65E-01	-5.88E-01	-1.50E+00	1.12E+01	1.69E+01
LOC616199	1.05E+03	1.14E-04	1.91E-04	5.90E-01	-7.62E-01	-1.70E+00	4.26E+01	7.23E+01
LOC616200	3.68E+03	1.36E-05	3.04E-05	6.67E-01	-5.85E-01	-1.50E+00	1.59E+02	2.38E+02
LOC616281	2.20E+03	2.76E-06	8.13E-06	7.45E-01	-4.24E-01	-1.34E+00	1.00E+02	1.34E+02
LOC616860	9.92E+01	9.31E-10	2.81E-08	1.08E-03	-9.85E+00	-9.23E+02	1.29E-02	1.19E+01
LOC616995	3.19E+00	2.33E-04	3.53E-04	2.58E-01	-1.95E+00	-3.87E+00	7.29E-02	2.83E-01

LOC617396	3.76E+01	5.83E-05	1.07E-04	5.68E-01	-8.16E-01	-1.76E+00	1.47E+00	2.58E+00
LOC618377	4.96E+02	1.63E-04	2.61E-04	5.63E-01	-8.29E-01	-1.78E+00	1.94E+01	3.44E+01
LOC780968	7.04E+02	6.40E-04	8.61E-04	8.12E-01	-3.00E-01	-1.23E+00	3.35E+01	4.13E+01
LOC781100	2.78E+02	8.85E-06	2.15E-05	6.44E-01	-6.34E-01	-1.55E+00	1.17E+01	1.82E+01
LOC781381	3.24E+03	8.80E-04	1.14E-03	8.13E-01	-2.98E-01	-1.23E+00	1.54E+02	1.90E+02
LOC782061	3.41E+01	1.94E-05	4.11E-05	1.20E-01	-3.06E+00	-8.35E+00	4.29E-01	3.58E+00
LOC782264	8.83E+02	8.11E-07	3.11E-06	5.64E-01	-8.26E-01	-1.77E+00	3.46E+01	6.14E+01
LOC782305	1.85E+01	2.25E-04	3.44E-04	5.96E-01	-7.46E-01	-1.68E+00	7.41E-01	1.24E+00
LOC782392	1.71E+01	9.54E-03	1.03E-02	6.94E-01	-5.27E-01	-1.44E+00	7.41E-01	1.07E+00
LOC782966	6.76E+02	4.65E-05	8.87E-05	6.79E-01	-5.58E-01	-1.47E+00	2.94E+01	4.32E+01
LOC783202	3.83E+02	8.99E-07	3.36E-06	6.62E-01	-5.95E-01	-1.51E+00	1.64E+01	2.48E+01
LOC783434	1.24E+01	1.69E-02	1.78E-02	6.85E-01	-5.45E-01	-1.46E+00	5.41E-01	7.89E-01
LOC783477	3.96E+00	6.08E-02	6.15E-02	3.72E-01	-1.43E+00	-2.69E+00	1.18E-01	3.17E-01
LOC783497	2.23E+01	2.09E-04	3.23E-04	3.40E-01	-1.55E+00	-2.94E+00	6.19E-01	1.82E+00
LOC783612	7.47E+02	5.36E-07	2.33E-06	6.20E-01	-6.90E-01	-1.61E+00	3.10E+01	5.00E+01
LOC783657	1.95E+03	4.78E-06	1.28E-05	7.26E-01	-4.62E-01	-1.38E+00	8.78E+01	1.21E+02
LOC783776	6.29E+01	4.28E-04	6.05E-04	7.17E-01	-4.79E-01	-1.39E+00	2.80E+00	3.91E+00
LOC783835	7.85E+01	6.98E-04	9.32E-04	6.78E-01	-5.61E-01	-1.48E+00	3.40E+00	5.01E+00
LOC784266	8.96E+01	4.80E-04	6.69E-04	6.93E-01	-5.29E-01	-1.44E+00	3.93E+00	5.67E+00
LOC784357	2.54E+00	3.33E-02	3.43E-02	3.95E-01	-1.34E+00	-2.53E+00	7.78E-02	1.97E-01
LOC784697	3.76E+01	1.60E-05	3.48E-05	5.23E-01	-9.34E-01	-1.91E+00	1.40E+00	2.67E+00
LOC784713	1.53E+02	2.21E-06	6.75E-06	6.59E-01	-6.02E-01	-1.52E+00	6.47E+00	9.82E+00
LOC785479	5.84E+01	3.26E-05	6.45E-05	6.35E-01	-6.56E-01	-1.58E+00	2.44E+00	3.84E+00
LOC785503	1.60E+03	4.17E-06	1.15E-05	8.33E-01	-2.64E-01	-1.20E+00	7.72E+01	9.27E+01
LOC785569	1.87E+02	2.50E-05	5.13E-05	6.87E-01	-5.41E-01	-1.45E+00	8.16E+00	1.19E+01
LOC785735	8.19E+00	2.88E-03	3.39E-03	3.79E-01	-1.40E+00	-2.64E+00	2.51E-01	6.63E-01
LOC785761	2.75E+03	2.77E-04	4.11E-04	7.93E-01	-3.34E-01	-1.26E+00	1.30E+02	1.64E+02
LOC785843	1.68E+02	1.36E-04	2.23E-04	7.54E-01	-4.07E-01	-1.33E+00	7.66E+00	1.01E+01
LOC786733	1.43E+02	2.12E-04	3.27E-04	6.55E-01	-6.11E-01	-1.53E+00	6.11E+00	9.33E+00
LOC786906	6.51E+01	8.56E-05	1.50E-04	5.94E-01	-7.51E-01	-1.68E+00	2.62E+00	4.41E+00
LOC789364	3.50E+00	2.20E-02	2.30E-02	2.94E-01	-1.77E+00	-3.40E+00	9.02E-02	3.07E-01
LOC790266	2.21E+03	1.11E-04	1.85E-04	8.18E-01	-2.90E-01	-1.22E+00	1.06E+02	1.30E+02
LONP1	1.85E+03	3.46E-07	1.66E-06	6.12E-01	-7.07E-01	-1.63E+00	7.57E+01	1.24E+02

LONP2	7.81E+02	1.38E-02	1.46E-02	8.62E-01	-2.14E-01	-1.16E+00	3.81E+01	4.42E+01
LPAR2	2.60E+02	1.14E-07	7.48E-07	5.17E-01	-9.51E-01	-1.93E+00	9.55E+00	1.85E+01
LRCH4	1.44E+03	1.32E-07	8.26E-07	6.83E-01	-5.50E-01	-1.46E+00	6.21E+01	9.09E+01
LRFN1	3.79E+02	1.97E-07	1.09E-06	5.64E-01	-8.27E-01	-1.77E+00	1.47E+01	2.61E+01
LRFN2	1.12E+02	6.04E-06	1.56E-05	3.04E-01	-1.72E+00	-3.29E+00	2.88E+00	9.48E+00
LRFN4	3.42E+02	2.38E-08	2.21E-07	5.54E-01	-8.53E-01	-1.81E+00	1.31E+01	2.37E+01
LRG1	3.08E+01	2.75E-06	8.12E-06	2.18E-01	-2.20E+00	-4.59E+00	6.27E-01	2.88E+00
LRP5	3.83E+03	2.79E-04	4.13E-04	7.39E-01	-4.37E-01	-1.35E+00	1.74E+02	2.35E+02
LRRC26	2.95E+00	2.16E-02	2.25E-02	3.49E-01	-1.52E+00	-2.87E+00	8.15E-02	2.33E-01
LRRC4	1.72E+02	1.93E-05	4.09E-05	4.46E-01	-1.16E+00	-2.24E+00	5.78E+00	1.30E+01
LRRC4B	9.60E+02	1.33E-06	4.54E-06	2.58E-01	-1.96E+00	-3.88E+00	2.15E+01	8.35E+01
LRRC4C	9.62E+01	5.48E-05	1.02E-04	4.13E-01	-1.27E+00	-2.42E+00	3.05E+00	7.38E+00
LRRC77	3.24E+00	3.32E-02	3.42E-02	3.56E-01	-1.49E+00	-2.81E+00	9.76E-02	2.75E-01
LRRN2	9.23E+02	1.09E-04	1.82E-04	7.18E-01	-4.79E-01	-1.39E+00	4.12E+01	5.74E+01
LSM6	4.81E+02	1.85E-04	2.91E-04	7.54E-01	-4.07E-01	-1.33E+00	2.21E+01	2.93E+01
LSM8	4.05E+02	1.50E-06	4.97E-06	6.77E-01	-5.63E-01	-1.48E+00	1.74E+01	2.58E+01
LTF	2.49E+02	8.84E-02	8.91E-02	6.95E-01	-5.25E-01	-1.44E+00	1.11E+01	1.60E+01
LUC7L	3.93E+03	2.31E-04	3.51E-04	8.68E-01	-2.04E-01	-1.15E+00	1.94E+02	2.23E+02
LURAP1L	1.77E+02	6.07E-05	1.11E-04	4.03E-01	-1.31E+00	-2.48E+00	5.73E+00	1.42E+01
LXN	5.04E+02	6.41E-06	1.64E-05	6.61E-01	-5.97E-01	-1.51E+00	2.16E+01	3.27E+01
LY6E	1.34E+03	3.68E-05	7.18E-05	4.26E-01	-1.23E+00	-2.34E+00	4.27E+01	1.00E+02
LYPD2	1.01E+01	1.02E-04	1.74E-04	7.47E-02	-3.74E+00	-1.34E+01	8.10E-02	1.08E+00
LYPLA1	4.77E+02	4.01E-03	4.60E-03	8.30E-01	-2.69E-01	-1.20E+00	2.29E+01	2.75E+01
LYPLA2	1.03E+03	3.87E-09	6.56E-08	5.64E-01	-8.25E-01	-1.77E+00	4.02E+01	7.13E+01
LYRM2	3.09E+02	5.53E-09	7.99E-08	5.32E-01	-9.09E-01	-1.88E+00	1.15E+01	2.17E+01
LYRM4	4.98E+02	9.82E-04	1.26E-03	7.76E-01	-3.66E-01	-1.29E+00	2.31E+01	2.98E+01
LZTFL1	6.17E+02	1.05E-05	2.46E-05	5.52E-01	-8.58E-01	-1.81E+00	2.33E+01	4.23E+01
LZTS1	2.88E+03	1.75E-06	5.61E-06	5.96E-01	-7.47E-01	-1.68E+00	1.16E+02	1.94E+02
LZTS3	1.79E+03	2.74E-07	1.38E-06	6.81E-01	-5.54E-01	-1.47E+00	7.80E+01	1.15E+02
MACROD1	5.17E+02	4.66E-07	2.07E-06	6.60E-01	-6.00E-01	-1.52E+00	2.20E+01	3.33E+01
MAD2L2	3.23E+02	6.29E-04	8.48E-04	7.96E-01	-3.30E-01	-1.26E+00	1.52E+01	1.91E+01
MAF1	2.21E+03	1.22E-06	4.29E-06	7.57E-01	-4.02E-01	-1.32E+00	1.01E+02	1.34E+02
MAFK	3.47E+02	2.41E-02	2.50E-02	7.53E-01	-4.09E-01	-1.33E+00	1.58E+01	2.10E+01

MAGED1	8.61E+03	1.87E-02	1.96E-02	8.87E-01	-1.73E-01	-1.13E+00	4.27E+02	4.81E+02
MAGED2	5.48E+03	4.88E-06	1.31E-05	7.12E-01	-4.90E-01	-1.40E+00	2.43E+02	3.42E+02
MAGED4B	4.15E+03	5.31E-04	7.30E-04	8.59E-01	-2.19E-01	-1.16E+00	2.03E+02	2.36E+02
MAGOHB	3.53E+02	7.23E-06	1.82E-05	6.98E-01	-5.18E-01	-1.43E+00	1.56E+01	2.23E+01
MAMDC4	1.99E+02	1.47E-02	1.55E-02	7.94E-01	-3.34E-01	-1.26E+00	9.35E+00	1.18E+01
MAN1B1	2.16E+03	1.81E-03	2.21E-03	7.80E-01	-3.58E-01	-1.28E+00	1.01E+02	1.29E+02
MANBAL	8.66E+02	6.97E-04	9.31E-04	8.73E-01	-1.96E-01	-1.15E+00	4.28E+01	4.90E+01
MAP3K11	1.27E+03	1.64E-07	9.55E-07	7.56E-01	-4.04E-01	-1.32E+00	5.83E+01	7.71E+01
MAP4K2	8.84E+02	1.71E-04	2.73E-04	6.24E-01	-6.80E-01	-1.60E+00	3.65E+01	5.85E+01
MAP6D1	3.10E+02	1.48E-03	1.84E-03	7.02E-01	-5.10E-01	-1.42E+00	1.36E+01	1.94E+01
MAPK10	8.44E+01	5.56E-02	5.64E-02	7.41E-01	-4.32E-01	-1.35E+00	3.77E+00	5.09E+00
MAPK13	4.09E+02	5.28E-05	9.83E-05	3.89E-01	-1.36E+00	-2.57E+00	1.26E+01	3.24E+01
MAPK3	2.05E+03	1.09E-05	2.54E-05	7.98E-01	-3.25E-01	-1.25E+00	9.65E+01	1.21E+02
MAPK4	2.47E+02	4.00E-05	7.74E-05	5.06E-01	-9.83E-01	-1.98E+00	9.10E+00	1.80E+01
MAPK8IP2	2.17E+02	2.28E-09	4.73E-08	8.15E-02	-3.62E+00	-1.23E+01	1.88E+00	2.30E+01
MARCKSL1	7.28E+03	7.84E-09	9.75E-08	5.59E-01	-8.38E-01	-1.79E+00	2.83E+02	5.06E+02
MARK4-2	5.11E+02	4.52E-05	8.65E-05	7.40E-01	-4.35E-01	-1.35E+00	2.32E+01	3.14E+01
MATN1	6.76E+01	9.69E-07	3.56E-06	1.92E-01	-2.38E+00	-5.20E+00	1.23E+00	6.40E+00
MB	5.10E+01	1.03E-02	1.10E-02	7.29E-01	-4.57E-01	-1.37E+00	2.28E+00	3.14E+00
MBD3	2.08E+03	6.64E-08	4.88E-07	6.98E-01	-5.18E-01	-1.43E+00	9.13E+01	1.31E+02
MBD4	4.35E+02	2.27E-04	3.46E-04	7.19E-01	-4.77E-01	-1.39E+00	1.93E+01	2.69E+01
MBNL3	7.38E+02	2.44E-05	5.01E-05	5.11E-01	-9.69E-01	-1.96E+00	2.72E+01	5.32E+01
MBOAT7	1.10E+03	2.29E-05	4.75E-05	6.80E-01	-5.57E-01	-1.47E+00	4.77E+01	7.02E+01
MCHR2	4.62E+01	2.14E-03	2.58E-03	7.00E-01	-5.15E-01	-1.43E+00	2.04E+00	2.91E+00
MCOLN3	1.10E+02	5.12E-06	1.36E-05	1.86E-01	-2.43E+00	-5.39E+00	1.99E+00	1.07E+01
MCRIP2	2.69E+02	1.55E-03	1.91E-03	6.65E-01	-5.88E-01	-1.50E+00	1.16E+01	1.74E+01
MDFI	1.22E+03	3.56E-04	5.12E-04	6.91E-01	-5.33E-01	-1.45E+00	5.31E+01	7.69E+01
MDH1	1.53E+03	1.10E-09	2.99E-08	4.80E-01	-1.06E+00	-2.08E+00	5.36E+01	1.12E+02
MDH2	3.03E+03	1.68E-06	5.45E-06	6.09E-01	-7.16E-01	-1.64E+00	1.23E+02	2.03E+02
MDK	9.90E+03	8.90E-05	1.55E-04	5.50E-01	-8.62E-01	-1.82E+00	3.81E+02	6.92E+02
MEA1	1.09E+03	1.25E-05	2.86E-05	6.29E-01	-6.69E-01	-1.59E+00	4.51E+01	7.16E+01
MECR	2.33E+02	2.06E-05	4.34E-05	6.83E-01	-5.50E-01	-1.46E+00	1.02E+01	1.49E+01
MED16	1.55E+03	4.32E-09	6.82E-08	6.56E-01	-6.09E-01	-1.52E+00	6.60E+01	1.01E+02

MED21	1.16E+03	3.75E-09	6.41E-08	5.40E-01	-8.88E-01	-1.85E+00	4.38E+01	8.11E+01
MED6	3.33E+02	8.07E-07	3.11E-06	6.75E-01	-5.67E-01	-1.48E+00	1.43E+01	2.12E+01
MEPCE	1.94E+03	8.72E-07	3.30E-06	7.49E-01	-4.17E-01	-1.34E+00	8.84E+01	1.18E+02
METRN	3.77E+02	1.21E-07	7.76E-07	5.38E-01	-8.95E-01	-1.86E+00	1.42E+01	2.64E+01
METTL23	3.03E+02	4.32E-03	4.91E-03	7.22E-01	-4.70E-01	-1.38E+00	1.35E+01	1.87E+01
METTL26	4.16E+02	1.09E-03	1.40E-03	7.51E-01	-4.13E-01	-1.33E+00	1.90E+01	2.53E+01
METTL27	9.52E+01	9.43E-05	1.62E-04	5.26E-01	-9.26E-01	-1.90E+00	3.53E+00	6.70E+00
METTL2A	4.91E+02	1.32E-03	1.66E-03	8.87E-01	-1.72E-01	-1.13E+00	2.45E+01	2.76E+01
MEX3D	1.55E+03	7.46E-07	2.94E-06	7.05E-01	-5.03E-01	-1.42E+00	6.90E+01	9.78E+01
MFAP3L	7.71E+01	1.20E-07	7.67E-07	2.84E-01	-1.82E+00	-3.52E+00	1.91E+00	6.71E+00
MFSD10	9.42E+02	2.88E-03	3.39E-03	8.59E-01	-2.19E-01	-1.16E+00	4.62E+01	5.38E+01
MFSD12	3.78E+02	2.42E-06	7.27E-06	6.45E-01	-6.32E-01	-1.55E+00	1.60E+01	2.48E+01
MFSD2A	1.94E+02	4.16E-02	4.25E-02	6.29E-01	-6.68E-01	-1.59E+00	8.25E+00	1.31E+01
MGAM	1.95E+02	6.96E-12	1.10E-09	6.27E-03	-7.32E+00	-1.59E+02	1.46E-01	2.33E+01
MGAT3	8.42E+02	6.62E-09	8.75E-08	3.28E-01	-1.61E+00	-3.05E+00	2.30E+01	7.01E+01
MIA	1.12E+02	5.20E-06	1.38E-05	5.36E-01	-9.00E-01	-1.87E+00	4.23E+00	7.89E+00
MIA2	5.08E+02	8.25E-03	8.95E-03	8.48E-01	-2.38E-01	-1.18E+00	2.46E+01	2.90E+01
MID1IP1	1.68E+03	1.86E-06	5.87E-06	7.36E-01	-4.43E-01	-1.36E+00	7.60E+01	1.03E+02
MIDN	2.23E+03	4.28E-08	3.51E-07	6.35E-01	-6.55E-01	-1.57E+00	9.32E+01	1.47E+02
MIF	1.93E+03	3.55E-05	6.96E-05	6.14E-01	-7.03E-01	-1.63E+00	7.93E+01	1.29E+02
MIF4GD	4.91E+02	3.69E-02	3.78E-02	8.97E-01	-1.57E-01	-1.12E+00	2.47E+01	2.75E+01
MLST8	7.48E+02	9.46E-05	1.62E-04	7.34E-01	-4.46E-01	-1.36E+00	3.38E+01	4.60E+01
MMADHC	1.09E+03	1.51E-05	3.32E-05	7.58E-01	-3.99E-01	-1.32E+00	5.00E+01	6.59E+01
MMP11	4.45E+02	2.66E-07	1.35E-06	4.94E-01	-1.02E+00	-2.02E+00	1.61E+01	3.27E+01
MMP17	6.76E+01	7.32E-05	1.30E-04	5.99E-01	-7.40E-01	-1.67E+00	2.74E+00	4.58E+00
MMP25	2.90E+02	8.11E-07	3.11E-06	2.15E-01	-2.22E+00	-4.65E+00	5.57E+00	2.59E+01
MNX1	8.17E+01	2.21E-07	1.20E-06	1.24E-01	-3.01E+00	-8.08E+00	1.03E+00	8.30E+00
MOGAT1	5.15E+01	2.06E-05	4.34E-05	4.16E-01	-1.26E+00	-2.40E+00	1.66E+00	3.98E+00
MOK	2.29E+02	1.69E-03	2.07E-03	6.76E-01	-5.64E-01	-1.48E+00	9.82E+00	1.45E+01
MORN4	3.50E+02	4.40E-03	4.99E-03	7.69E-01	-3.78E-01	-1.30E+00	1.60E+01	2.08E+01
MOSPD3	6.80E+02	9.69E-03	1.04E-02	8.42E-01	-2.48E-01	-1.19E+00	3.30E+01	3.92E+01
MOV10	2.17E+03	8.69E-13	3.74E-10	3.59E-01	-1.48E+00	-2.79E+00	6.38E+01	1.78E+02
MPC2	2.75E+02	2.84E-03	3.35E-03	7.89E-01	-3.42E-01	-1.27E+00	1.29E+01	1.63E+01

MPG	5.34E+02	3.16E-04	4.62E-04	7.35E-01	-4.43E-01	-1.36E+00	2.41E+01	3.27E+01
MPND	4.01E+02	1.66E-04	2.65E-04	8.10E-01	-3.03E-01	-1.23E+00	1.91E+01	2.36E+01
MPP1	1.18E+03	7.71E-05	1.37E-04	8.08E-01	-3.08E-01	-1.24E+00	5.62E+01	6.96E+01
MPV17	6.93E+02	1.66E-04	2.66E-04	8.02E-01	-3.19E-01	-1.25E+00	3.29E+01	4.11E+01
MPV17L2	1.48E+02	1.63E-05	3.56E-05	4.61E-01	-1.12E+00	-2.17E+00	5.07E+00	1.10E+01
MRAP	6.16E+00	1.39E-02	1.48E-02	5.61E-01	-8.34E-01	-1.78E+00	2.41E-01	4.30E-01
MRAS	1.31E+03	7.52E-06	1.88E-05	7.94E-01	-3.33E-01	-1.26E+00	6.17E+01	7.77E+01
MRI1	4.56E+02	4.28E-05	8.23E-05	7.69E-01	-3.79E-01	-1.30E+00	2.12E+01	2.76E+01
MRPL11	1.18E+03	2.26E-04	3.45E-04	7.07E-01	-5.00E-01	-1.41E+00	5.20E+01	7.35E+01
MRPL12	4.87E+02	9.63E-07	3.55E-06	5.55E-01	-8.50E-01	-1.80E+00	1.88E+01	3.39E+01
MRPL24	6.60E+02	1.78E-07	1.01E-06	6.02E-01	-7.31E-01	-1.66E+00	2.67E+01	4.42E+01
MRPL28	8.14E+02	1.35E-07	8.42E-07	7.08E-01	-4.98E-01	-1.41E+00	3.61E+01	5.10E+01
MRPL34	3.59E+02	1.80E-05	3.87E-05	7.04E-01	-5.06E-01	-1.42E+00	1.60E+01	2.27E+01
MRPL36	5.57E+02	3.72E-03	4.29E-03	8.07E-01	-3.09E-01	-1.24E+00	2.65E+01	3.28E+01
MRPL4	1.12E+03	1.09E-02	1.16E-02	8.70E-01	-2.01E-01	-1.15E+00	5.54E+01	6.37E+01
MRPL45	9.30E+02	8.02E-04	1.05E-03	8.05E-01	-3.13E-01	-1.24E+00	4.41E+01	5.47E+01
MRPL52	7.66E+02	6.90E-03	7.58E-03	8.58E-01	-2.21E-01	-1.17E+00	3.75E+01	4.37E+01
MRPL54	7.03E+02	9.95E-05	1.70E-04	7.87E-01	-3.45E-01	-1.27E+00	3.29E+01	4.18E+01
MRPL55	5.73E+02	4.01E-06	1.12E-05	6.28E-01	-6.72E-01	-1.59E+00	2.37E+01	3.78E+01
MRPL9	1.62E+03	1.35E-05	3.03E-05	7.21E-01	-4.73E-01	-1.39E+00	7.23E+01	1.00E+02
MRPS12	2.46E+02	5.39E-04	7.40E-04	7.24E-01	-4.65E-01	-1.38E+00	1.11E+01	1.53E+01
MRPS15	4.86E+02	4.09E-06	1.14E-05	5.91E-01	-7.59E-01	-1.69E+00	1.94E+01	3.28E+01
MRPS18A	7.13E+02	1.71E-02	1.80E-02	8.90E-01	-1.68E-01	-1.12E+00	3.57E+01	4.01E+01
MRPS24	2.62E+02	1.85E-06	5.86E-06	6.50E-01	-6.22E-01	-1.54E+00	1.10E+01	1.70E+01
MRPS27	9.54E+02	4.15E-05	7.99E-05	7.76E-01	-3.65E-01	-1.29E+00	4.43E+01	5.71E+01
MRPS28	1.72E+02	1.28E-06	4.45E-06	6.90E-01	-5.35E-01	-1.45E+00	7.49E+00	1.09E+01
MRPS33	6.99E+02	1.13E-03	1.44E-03	8.16E-01	-2.94E-01	-1.23E+00	3.33E+01	4.08E+01
MRPS35	5.64E+02	4.47E-05	8.57E-05	7.43E-01	-4.29E-01	-1.35E+00	2.54E+01	3.43E+01
MRPS9	8.81E+02	2.38E-08	2.21E-07	6.64E-01	-5.91E-01	-1.51E+00	3.78E+01	5.69E+01
MRTO4	1.41E+03	3.30E-05	6.52E-05	8.00E-01	-3.22E-01	-1.25E+00	6.65E+01	8.30E+01
MSH2	1.12E+03	3.68E-04	5.27E-04	7.73E-01	-3.71E-01	-1.29E+00	5.21E+01	6.74E+01
MSI1	7.89E+02	1.22E-07	7.76E-07	3.00E-01	-1.74E+00	-3.33E+00	1.99E+01	6.64E+01
MSLN	1.13E+03	2.35E-03	2.81E-03	3.81E-01	-1.39E+00	-2.62E+00	3.38E+01	8.86E+01

MSRA	3.06E+02	8.58E-05	1.50E-04	6.90E-01	-5.36E-01	-1.45E+00	1.34E+01	1.94E+01
MSX2	9.23E+01	8.84E-05	1.54E-04	2.06E-01	-2.28E+00	-4.86E+00	1.79E+00	8.72E+00
MTA1	8.34E+02	2.09E-05	4.40E-05	7.37E-01	-4.40E-01	-1.36E+00	3.77E+01	5.11E+01
MTA2	3.52E+03	1.12E-09	2.99E-08	6.48E-01	-6.26E-01	-1.54E+00	1.48E+02	2.29E+02
MTFR2	1.61E+02	4.49E-03	5.09E-03	5.92E-01	-7.56E-01	-1.69E+00	6.35E+00	1.07E+01
MTHFD1L	8.09E+02	1.02E-04	1.74E-04	5.75E-01	-7.98E-01	-1.74E+00	3.20E+01	5.57E+01
MTHFS	7.67E+02	2.15E-10	1.05E-08	5.39E-01	-8.93E-01	-1.86E+00	2.90E+01	5.39E+01
MTSS1L	5.88E+03	7.51E-07	2.95E-06	5.41E-01	-8.85E-01	-1.85E+00	2.23E+02	4.11E+02
MXD3	1.77E+02	5.10E-05	9.58E-05	5.47E-01	-8.72E-01	-1.83E+00	6.79E+00	1.24E+01
MYCBPAP	1.85E+02	1.01E-04	1.72E-04	5.19E-01	-9.45E-01	-1.93E+00	6.76E+00	1.30E+01
MYCN	5.57E+02	1.11E-06	3.98E-06	3.44E-01	-1.54E+00	-2.90E+00	1.57E+01	4.55E+01
MYH6	1.81E+02	1.04E-06	3.78E-06	3.11E-01	-1.68E+00	-3.21E+00	4.80E+00	1.54E+01
MYH8	6.30E+01	5.01E-05	9.43E-05	3.10E-01	-1.69E+00	-3.22E+00	1.62E+00	5.23E+00
MYL10	1.93E+00	1.57E-04	2.53E-04	1.29E-01	-2.95E+00	-7.74E+00	2.56E-02	1.99E-01
MYL6B	4.33E+02	2.94E-07	1.45E-06	5.21E-01	-9.40E-01	-1.92E+00	1.60E+01	3.07E+01
MYLK2	7.17E+00	1.89E-02	1.98E-02	5.42E-01	-8.82E-01	-1.84E+00	2.73E-01	5.03E-01
MYO19	3.39E+02	1.94E-04	3.03E-04	6.41E-01	-6.41E-01	-1.56E+00	1.43E+01	2.24E+01
MYO3B	1.86E+01	1.65E-06	5.38E-06	1.63E-01	-2.61E+00	-6.12E+00	2.98E-01	1.82E+00
MYOM3	3.04E+02	7.73E-04	1.02E-03	5.73E-01	-8.03E-01	-1.74E+00	1.20E+01	2.09E+01
MZB1	4.72E+01	4.49E-02	4.58E-02	7.22E-01	-4.69E-01	-1.38E+00	2.14E+00	2.96E+00
MZF1	3.02E+03	2.19E-05	4.56E-05	7.25E-01	-4.63E-01	-1.38E+00	1.35E+02	1.87E+02
N4BP3	2.43E+02	9.83E-06	2.33E-05	5.78E-01	-7.92E-01	-1.73E+00	9.61E+00	1.66E+01
NABP2	8.33E+02	1.16E-06	4.11E-06	7.39E-01	-4.36E-01	-1.35E+00	3.78E+01	5.11E+01
NACA	5.54E+03	1.50E-03	1.85E-03	8.61E-01	-2.16E-01	-1.16E+00	2.71E+02	3.15E+02
NAGPA	3.15E+02	1.93E-04	3.02E-04	8.09E-01	-3.06E-01	-1.24E+00	1.50E+01	1.86E+01
NAIF1	1.60E+02	5.47E-04	7.51E-04	7.23E-01	-4.68E-01	-1.38E+00	7.12E+00	9.85E+00
NANOG	2.63E+02	7.55E-09	9.50E-08	9.18E-04	-1.01E+01	-1.09E+03	2.91E-02	3.17E+01
NANP	1.61E+02	8.98E-02	9.05E-02	8.96E-01	-1.59E-01	-1.12E+00	8.00E+00	8.94E+00
NANS	4.54E+02	1.44E-03	1.79E-03	7.41E-01	-4.32E-01	-1.35E+00	2.07E+01	2.79E+01
NAP1L5	1.47E+02	9.63E-04	1.24E-03	6.67E-01	-5.85E-01	-1.50E+00	6.28E+00	9.41E+00
NAPA	1.50E+03	9.00E-05	1.56E-04	8.23E-01	-2.81E-01	-1.21E+00	7.19E+01	8.74E+01
NAT14	4.67E+02	5.75E-04	7.83E-04	8.04E-01	-3.15E-01	-1.24E+00	2.21E+01	2.75E+01
NAXD	9.98E+02	4.82E-05	9.14E-05	7.47E-01	-4.22E-01	-1.34E+00	4.54E+01	6.08E+01

NCKAP5L	2.79E+03	2.16E-04	3.33E-04	7.72E-01	-3.73E-01	-1.30E+00	1.30E+02	1.68E+02
NCR3LG1	1.35E+02	2.33E-06	7.06E-06	6.32E-01	-6.63E-01	-1.58E+00	5.60E+00	8.86E+00
NCS1	4.73E+02	7.95E-05	1.41E-04	6.46E-01	-6.31E-01	-1.55E+00	1.99E+01	3.08E+01
ND1	3.40E+04	3.61E-04	5.19E-04	5.32E-01	-9.10E-01	-1.88E+00	1.27E+03	2.39E+03
ND2	3.12E+04	1.88E-04	2.95E-04	6.02E-01	-7.33E-01	-1.66E+00	1.25E+03	2.08E+03
ND3	1.82E+04	2.96E-02	3.05E-02	7.30E-01	-4.54E-01	-1.37E+00	8.16E+02	1.12E+03
ND4	4.98E+04	4.58E-04	6.41E-04	6.20E-01	-6.89E-01	-1.61E+00	2.03E+03	3.28E+03
ND4L	5.60E+03	4.94E-04	6.86E-04	6.16E-01	-6.98E-01	-1.62E+00	2.28E+02	3.69E+02
NDC80	1.92E+02	1.17E-04	1.94E-04	6.26E-01	-6.75E-01	-1.60E+00	7.93E+00	1.27E+01
NDN	1.07E+03	4.20E-06	1.16E-05	7.07E-01	-5.01E-01	-1.42E+00	4.75E+01	6.73E+01
NDUFAF5	8.15E+01	5.80E-03	6.43E-03	7.43E-01	-4.28E-01	-1.35E+00	3.72E+00	5.00E+00
NDUFB1	1.25E+02	2.55E-05	5.20E-05	5.23E-01	-9.36E-01	-1.91E+00	4.64E+00	8.87E+00
NDUFB3	4.51E+02	1.19E-04	1.98E-04	6.82E-01	-5.52E-01	-1.47E+00	1.96E+01	2.88E+01
NDUFB5	5.65E+02	1.37E-06	4.61E-06	5.87E-01	-7.68E-01	-1.70E+00	2.24E+01	3.82E+01
NDUFB6	3.54E+02	5.40E-03	6.03E-03	8.06E-01	-3.11E-01	-1.24E+00	1.68E+01	2.09E+01
NDUFB7	5.94E+02	3.03E-07	1.49E-06	6.51E-01	-6.20E-01	-1.54E+00	2.51E+01	3.86E+01
NDUFB8	1.01E+03	9.72E-06	2.31E-05	7.20E-01	-4.75E-01	-1.39E+00	4.52E+01	6.28E+01
NDUFC2	1.04E+03	5.68E-05	1.05E-04	7.54E-01	-4.08E-01	-1.33E+00	4.76E+01	6.32E+01
NDUFS2	2.70E+03	8.59E-05	1.50E-04	7.82E-01	-3.54E-01	-1.28E+00	1.26E+02	1.61E+02
NDUFS4	6.37E+02	3.46E-03	3.99E-03	7.28E-01	-4.58E-01	-1.37E+00	2.85E+01	3.92E+01
NDUFS7	6.42E+02	7.59E-07	2.97E-06	6.91E-01	-5.33E-01	-1.45E+00	2.81E+01	4.07E+01
NDUFV3	1.32E+03	2.33E-05	4.83E-05	6.90E-01	-5.34E-01	-1.45E+00	5.78E+01	8.37E+01
NECAB2	4.74E+02	3.28E-05	6.48E-05	4.39E-01	-1.19E+00	-2.28E+00	1.60E+01	3.63E+01
NECAP2	1.25E+03	1.19E-05	2.74E-05	7.73E-01	-3.72E-01	-1.29E+00	5.76E+01	7.45E+01
NECTIN1	5.95E+02	3.45E-08	2.98E-07	3.21E-01	-1.64E+00	-3.12E+00	1.59E+01	4.96E+01
NECTIN2	3.92E+03	7.27E-08	5.26E-07	5.83E-01	-7.79E-01	-1.72E+00	1.56E+02	2.67E+02
NEK6	1.59E+03	7.24E-04	9.61E-04	8.23E-01	-2.82E-01	-1.22E+00	7.65E+01	9.30E+01
NFATC4	2.11E+03	6.11E-04	8.26E-04	7.80E-01	-3.58E-01	-1.28E+00	9.81E+01	1.26E+02
NFE2L3	3.42E+02	1.36E-03	1.70E-03	6.15E-01	-7.02E-01	-1.63E+00	1.38E+01	2.25E+01
NHLH1	7.24E+01	1.64E-03	2.01E-03	6.68E-01	-5.83E-01	-1.50E+00	3.09E+00	4.63E+00
NHP2	4.21E+02	2.51E-02	2.60E-02	8.32E-01	-2.65E-01	-1.20E+00	2.03E+01	2.44E+01
NIFK	8.31E+02	9.98E-07	3.64E-06	6.90E-01	-5.36E-01	-1.45E+00	3.63E+01	5.26E+01
NINJ1	3.69E+02	2.44E-04	3.69E-04	6.87E-01	-5.42E-01	-1.46E+00	1.61E+01	2.34E+01

NIPSNAP2	1.12E+03	4.72E-02	4.81E-02	9.24E-01	-1.14E-01	-1.08E+00	5.68E+01	6.15E+01
NIT1	3.14E+02	2.87E-08	2.59E-07	5.98E-01	-7.41E-01	-1.67E+00	1.26E+01	2.11E+01
NKD1	1.88E+03	1.54E-02	1.62E-02	6.17E-01	-6.97E-01	-1.62E+00	7.73E+01	1.25E+02
NKIRAS2	1.80E+03	2.22E-04	3.40E-04	8.29E-01	-2.70E-01	-1.21E+00	8.65E+01	1.04E+02
NLGN4X	3.66E+02	1.33E-04	2.17E-04	4.02E-01	-1.32E+00	-2.49E+00	1.17E+01	2.91E+01
NLRX1	4.32E+02	1.31E-11	1.68E-09	4.66E-01	-1.10E+00	-2.15E+00	1.50E+01	3.22E+01
NME1	4.19E+02	2.63E-07	1.34E-06	5.56E-01	-8.47E-01	-1.80E+00	1.61E+01	2.90E+01
NME3	8.29E+02	6.64E-03	7.30E-03	8.57E-01	-2.22E-01	-1.17E+00	4.06E+01	4.74E+01
NME4	2.13E+03	8.21E-11	5.43E-09	5.70E-01	-8.12E-01	-1.76E+00	8.37E+01	1.47E+02
NMUR2	4.53E+00	3.78E-07	1.77E-06	1.17E-01	-3.09E+00	-8.54E+00	5.45E-02	4.65E-01
NNAT	1.28E+03	7.75E-06	1.92E-05	5.87E-01	-7.68E-01	-1.70E+00	5.12E+01	8.72E+01
NOB1	8.96E+02	1.33E-05	2.99E-05	7.24E-01	-4.66E-01	-1.38E+00	4.02E+01	5.55E+01
NOC4L	5.89E+02	4.44E-08	3.61E-07	5.64E-01	-8.25E-01	-1.77E+00	2.29E+01	4.06E+01
NOG	2.67E+01	1.77E-01	1.78E-01	5.65E-01	-8.23E-01	-1.77E+00	1.07E+00	1.90E+00
NOL4L	2.33E+03	5.35E-04	7.35E-04	6.94E-01	-5.26E-01	-1.44E+00	1.02E+02	1.46E+02
NOP56	1.81E+03	4.38E-04	6.16E-04	7.89E-01	-3.43E-01	-1.27E+00	8.47E+01	1.07E+02
NOS1AP	6.70E+01	6.85E-05	1.23E-04	4.04E-01	-1.31E+00	-2.47E+00	2.11E+00	5.22E+00
NOSIP	1.26E+03	3.17E-03	3.69E-03	8.78E-01	-1.87E-01	-1.14E+00	6.26E+01	7.13E+01
NPFF	7.02E+00	2.40E-06	7.22E-06	1.01E-01	-3.30E+00	-9.87E+00	7.54E-02	7.45E-01
NPM1	7.53E+03	5.59E-07	2.39E-06	7.20E-01	-4.74E-01	-1.39E+00	3.37E+02	4.68E+02
NPSR1	2.11E+01	3.32E-03	3.85E-03	3.00E-01	-1.74E+00	-3.34E+00	5.54E-01	1.85E+00
NQO1	2.35E+02	1.03E-04	1.74E-04	4.98E-01	-1.00E+00	-2.01E+00	8.66E+00	1.74E+01
NR_001576.1	1.75E+01	1.29E-04	2.12E-04	4.53E-01	-1.14E+00	-2.21E+00	6.02E-01	1.33E+00
NR_024614.1	2.32E+02	1.32E-06	4.49E-06	5.46E-01	-8.73E-01	-1.83E+00	8.86E+00	1.62E+01
NR_028029.1	6.63E+02	3.95E-04	5.62E-04	7.59E-01	-3.98E-01	-1.32E+00	3.03E+01	3.99E+01
NR_031133.1	2.10E+00	1.95E-02	2.04E-02	2.65E-01	-1.92E+00	-3.78E+00	4.91E-02	1.85E-01
NR_036663.1	2.44E+02	7.34E-04	9.71E-04	6.11E-01	-7.11E-01	-1.64E+00	1.00E+01	1.64E+01
NR_046270.1	1.84E+02	1.40E-03	1.74E-03	7.93E-01	-3.35E-01	-1.26E+00	8.66E+00	1.09E+01
NR_104052.1	4.98E+00	9.70E-03	1.04E-02	3.58E-01	-1.48E+00	-2.80E+00	1.48E-01	4.13E-01
NR_110389.1	1.06E+02	2.25E-04	3.43E-04	5.21E-01	-9.42E-01	-1.92E+00	3.88E+00	7.45E+00
NR_120605.1	3.09E+02	2.54E-06	7.55E-06	5.25E-01	-9.30E-01	-1.91E+00	1.16E+01	2.21E+01
NR_131901.1	3.38E+03	8.34E-05	1.46E-04	6.46E-01	-6.29E-01	-1.55E+00	1.43E+02	2.22E+02
NR_131934.1	1.77E+02	6.38E-05	1.16E-04	3.00E-01	-1.73E+00	-3.33E+00	4.57E+00	1.52E+01

NR_132275.1	6.96E+01	9.82E-04	1.26E-03	6.50E-01	-6.22E-01	-1.54E+00	2.93E+00	4.51E+00
NR_137293.1	3.69E+02	8.49E-06	2.07E-05	4.23E-01	-1.24E+00	-2.36E+00	1.20E+01	2.83E+01
NR0B1	1.67E+02	2.07E-08	2.03E-07	3.98E-01	-1.33E+00	-2.51E+00	5.27E+00	1.33E+01
NR2C2AP	2.52E+02	9.52E-04	1.23E-03	7.65E-01	-3.86E-01	-1.31E+00	1.16E+01	1.52E+01
NR5A1	4.33E+03	4.96E-08	3.91E-07	5.26E-01	-9.27E-01	-1.90E+00	1.62E+02	3.08E+02
NRG1	3.13E+02	6.39E-05	1.16E-04	4.97E-01	-1.01E+00	-2.01E+00	1.14E+01	2.29E+01
NRGN	6.65E+01	1.66E-02	1.74E-02	6.68E-01	-5.83E-01	-1.50E+00	2.88E+00	4.31E+00
NRM	5.17E+02	1.43E-06	4.80E-06	6.11E-01	-7.10E-01	-1.64E+00	2.11E+01	3.45E+01
NRSN2	1.89E+02	1.37E-06	4.61E-06	3.68E-01	-1.44E+00	-2.71E+00	5.58E+00	1.52E+01
NSMF	2.60E+03	2.11E-05	4.42E-05	7.22E-01	-4.69E-01	-1.38E+00	1.17E+02	1.61E+02
NT5C	5.05E+02	1.63E-03	2.01E-03	6.76E-01	-5.66E-01	-1.48E+00	2.21E+01	3.27E+01
NTF4	1.13E+02	3.25E-04	4.72E-04	5.31E-01	-9.12E-01	-1.88E+00	4.21E+00	7.93E+00
NTRK1	1.69E+02	1.06E-04	1.79E-04	3.31E-01	-1.60E+00	-3.02E+00	4.72E+00	1.43E+01
NUDCD3	1.63E+03	5.63E-09	8.07E-08	7.23E-01	-4.69E-01	-1.38E+00	7.33E+01	1.01E+02
NUDT10	1.55E+02	3.35E-06	9.61E-06	4.98E-01	-1.01E+00	-2.01E+00	5.60E+00	1.13E+01
NUDT14	2.97E+02	8.01E-07	3.10E-06	5.65E-01	-8.24E-01	-1.77E+00	1.16E+01	2.05E+01
NUDT16	5.01E+02	3.16E-03	3.69E-03	7.46E-01	-4.22E-01	-1.34E+00	2.29E+01	3.08E+01
NUDT5	3.66E+02	3.71E-02	3.80E-02	8.90E-01	-1.68E-01	-1.12E+00	1.83E+01	2.05E+01
NUP35	3.39E+02	1.89E-03	2.30E-03	8.03E-01	-3.16E-01	-1.24E+00	1.60E+01	1.99E+01
NUTM1	1.03E+02	6.73E-10	2.23E-08	3.28E-02	-4.93E+00	-3.05E+01	3.76E-01	1.15E+01
NXPH3	2.16E+02	1.28E-02	1.36E-02	6.66E-01	-5.85E-01	-1.50E+00	9.37E+00	1.41E+01
NYNRIN	7.89E+03	1.23E-06	4.32E-06	5.37E-01	-8.98E-01	-1.86E+00	2.99E+02	5.58E+02
OAZ1	5.71E+03	1.31E-06	4.49E-06	6.83E-01	-5.50E-01	-1.46E+00	2.48E+02	3.62E+02
OAZ2	2.74E+03	6.23E-07	2.56E-06	7.68E-01	-3.82E-01	-1.30E+00	1.27E+02	1.65E+02
OBP	7.97E+00	1.95E-02	2.04E-02	2.60E-01	-1.94E+00	-3.85E+00	1.88E-01	7.24E-01
OCA2	3.28E+01	4.79E-05	9.11E-05	1.70E-01	-2.55E+00	-5.87E+00	5.49E-01	3.22E+00
OCIAD1	2.38E+03	7.46E-07	2.94E-06	7.19E-01	-4.76E-01	-1.39E+00	1.06E+02	1.48E+02
OLFM1	1.70E+01	1.75E-07	1.00E-06	1.14E-01	-3.13E+00	-8.78E+00	1.99E-01	1.74E+00
OLIG3	1.66E+01	1.06E-10	6.39E-09	1.52E-03	-9.36E+00	-6.59E+02	2.99E-03	1.97E+00
ORAI1	3.85E+02	8.96E-04	1.16E-03	8.40E-01	-2.51E-01	-1.19E+00	1.86E+01	2.22E+01
OSGEP	5.27E+02	2.16E-05	4.51E-05	7.41E-01	-4.33E-01	-1.35E+00	2.39E+01	3.23E+01
OSR1	3.29E+02	2.00E-09	4.27E-08	1.60E-01	-2.64E+00	-6.25E+00	5.24E+00	3.27E+01
OTUB2	1.61E+02	2.26E-04	3.45E-04	7.15E-01	-4.83E-01	-1.40E+00	7.17E+00	1.00E+01

OXA1L	1.99E+03	9.56E-07	3.54E-06	7.12E-01	-4.90E-01	-1.40E+00	8.85E+01	1.24E+02
OXLD1	1.35E+02	3.92E-08	3.29E-07	5.83E-01	-7.78E-01	-1.71E+00	5.35E+00	9.18E+00
P2RX3	7.81E+01	2.15E-05	4.50E-05	4.37E-01	-1.19E+00	-2.29E+00	2.61E+00	5.96E+00
P3H4	1.12E+03	6.18E-03	6.82E-03	8.43E-01	-2.47E-01	-1.19E+00	5.42E+01	6.43E+01
PABPC1L	1.51E+02	6.59E-05	1.19E-04	5.99E-01	-7.40E-01	-1.67E+00	6.10E+00	1.02E+01
PABPN1	4.37E+03	6.13E-07	2.54E-06	7.21E-01	-4.71E-01	-1.39E+00	1.96E+02	2.72E+02
PACS2	2.50E+03	2.93E-06	8.55E-06	7.61E-01	-3.95E-01	-1.31E+00	1.15E+02	1.51E+02
PAGR1	1.11E+03	5.22E-07	2.27E-06	7.50E-01	-4.14E-01	-1.33E+00	5.08E+01	6.76E+01
PAK4	1.52E+03	1.17E-07	7.52E-07	5.71E-01	-8.09E-01	-1.75E+00	5.98E+01	1.05E+02
PALD1	7.47E+02	1.81E-06	5.76E-06	6.66E-01	-5.87E-01	-1.50E+00	3.20E+01	4.81E+01
PAMR1	1.22E+02	2.35E-04	3.56E-04	2.78E-01	-1.85E+00	-3.59E+00	3.02E+00	1.08E+01
PANX3	7.66E+00	5.30E-03	5.93E-03	2.18E-01	-2.19E+00	-4.58E+00	1.54E-01	7.06E-01
PAPSS1	2.06E+03	4.54E-04	6.35E-04	8.15E-01	-2.96E-01	-1.23E+00	9.84E+01	1.21E+02
PARVB	3.55E+02	6.78E-05	1.22E-04	7.07E-01	-4.99E-01	-1.41E+00	1.57E+01	2.23E+01
PATZ1	2.05E+03	3.52E-09	6.12E-08	6.24E-01	-6.80E-01	-1.60E+00	8.45E+01	1.35E+02
PAX5	1.05E+01	1.45E-02	1.54E-02	2.73E-01	-1.87E+00	-3.66E+00	2.45E-01	8.97E-01
PAXX	4.63E+02	9.34E-05	1.61E-04	6.92E-01	-5.30E-01	-1.44E+00	2.03E+01	2.93E+01
PBX2	4.88E+03	6.18E-09	8.56E-08	5.25E-01	-9.30E-01	-1.91E+00	1.82E+02	3.47E+02
PCBP3	1.15E+02	4.74E-07	2.10E-06	3.03E-01	-1.72E+00	-3.30E+00	2.94E+00	9.69E+00
PCBP4	2.17E+03	1.93E-09	4.17E-08	5.18E-01	-9.49E-01	-1.93E+00	8.00E+01	1.54E+02
PCED1B	1.46E+03	7.51E-06	1.88E-05	5.89E-01	-7.63E-01	-1.70E+00	5.88E+01	9.98E+01
PCGF2	2.33E+03	2.04E-05	4.31E-05	7.63E-01	-3.89E-01	-1.31E+00	1.08E+02	1.41E+02
PCP4L1	6.81E+02	6.10E-06	1.57E-05	4.47E-01	-1.16E+00	-2.24E+00	2.28E+01	5.11E+01
PCSK4	8.13E+02	2.11E-06	6.49E-06	5.45E-01	-8.75E-01	-1.83E+00	3.07E+01	5.64E+01
PCYOX1L	5.04E+02	1.43E-05	3.17E-05	5.69E-01	-8.14E-01	-1.76E+00	1.97E+01	3.46E+01
PCYT2	9.29E+02	1.29E-04	2.12E-04	6.40E-01	-6.44E-01	-1.56E+00	3.89E+01	6.08E+01
PDCL3	5.85E+02	2.00E-04	3.11E-04	7.87E-01	-3.45E-01	-1.27E+00	2.75E+01	3.49E+01
PDE6G	2.91E+01	8.11E-05	1.43E-04	3.18E-01	-1.65E+00	-3.14E+00	7.59E-01	2.38E+00
PDE9A	4.47E+02	2.74E-04	4.07E-04	7.80E-01	-3.59E-01	-1.28E+00	2.09E+01	2.68E+01
PDF	1.10E+02	1.43E-03	1.78E-03	7.60E-01	-3.97E-01	-1.32E+00	5.09E+00	6.70E+00
PDGFA	1.13E+03	1.74E-08	1.77E-07	3.63E-01	-1.46E+00	-2.76E+00	3.28E+01	9.05E+01
PDIA4	6.00E+03	1.31E-06	4.49E-06	7.15E-01	-4.85E-01	-1.40E+00	2.67E+02	3.73E+02
PDIA5	1.06E+03	7.67E-03	8.35E-03	7.42E-01	-4.30E-01	-1.35E+00	4.81E+01	6.47E+01

PDZD4	1.88E+02	6.11E-09	8.53E-08	2.91E-01	-1.78E+00	-3.44E+00	4.74E+00	1.63E+01
PEBP4	1.41E+01	1.06E-02	1.13E-02	3.70E-01	-1.44E+00	-2.70E+00	4.31E-01	1.17E+00
PEPD	9.44E+02	3.50E-05	6.86E-05	7.64E-01	-3.89E-01	-1.31E+00	4.34E+01	5.68E+01
PEX10	1.39E+03	3.00E-05	5.97E-05	7.87E-01	-3.45E-01	-1.27E+00	6.52E+01	8.29E+01
PEX11G	5.39E+02	7.36E-03	8.03E-03	8.30E-01	-2.69E-01	-1.21E+00	2.60E+01	3.13E+01
PEX14	1.07E+03	3.85E-06	1.08E-05	7.31E-01	-4.52E-01	-1.37E+00	4.84E+01	6.62E+01
PEX2	3.16E+02	3.43E-04	4.95E-04	7.98E-01	-3.26E-01	-1.25E+00	1.49E+01	1.87E+01
PEX5	1.29E+03	4.60E-09	7.03E-08	6.13E-01	-7.06E-01	-1.63E+00	5.27E+01	8.59E+01
PFDN1	9.06E+02	6.30E-04	8.48E-04	7.51E-01	-4.14E-01	-1.33E+00	4.15E+01	5.52E+01
PFKFB3	1.16E+03	8.76E-07	3.31E-06	4.69E-01	-1.09E+00	-2.13E+00	4.06E+01	8.65E+01
PFKL	1.71E+03	1.85E-06	5.86E-06	5.90E-01	-7.62E-01	-1.70E+00	6.91E+01	1.17E+02
PFN1	5.32E+03	2.61E-05	5.32E-05	7.50E-01	-4.16E-01	-1.33E+00	2.44E+02	3.26E+02
PGLS	1.06E+03	4.51E-06	1.23E-05	6.93E-01	-5.29E-01	-1.44E+00	4.65E+01	6.71E+01
PHB	1.72E+03	7.02E-07	2.81E-06	6.86E-01	-5.44E-01	-1.46E+00	7.51E+01	1.09E+02
PHB2	2.29E+03	4.51E-04	6.32E-04	7.98E-01	-3.26E-01	-1.25E+00	1.08E+02	1.36E+02
PHC2	3.03E+03	6.06E-06	1.56E-05	8.22E-01	-2.83E-01	-1.22E+00	1.46E+02	1.77E+02
PHETA1	3.92E+02	3.25E-07	1.57E-06	7.38E-01	-4.39E-01	-1.36E+00	1.78E+01	2.41E+01
PHF21B	8.63E+01	3.51E-07	1.67E-06	2.12E-01	-2.24E+00	-4.72E+00	1.71E+00	8.09E+00
PHGDH	2.09E+03	3.34E-08	2.93E-07	3.88E-01	-1.37E+00	-2.58E+00	6.43E+01	1.66E+02
PHKG2	1.21E+03	4.98E-08	3.91E-07	6.46E-01	-6.31E-01	-1.55E+00	5.09E+01	7.88E+01
PHLDA1	8.27E+02	1.10E-05	2.56E-05	4.51E-01	-1.15E+00	-2.22E+00	2.81E+01	6.22E+01
PHLDB1	7.13E+03	3.70E-04	5.30E-04	7.21E-01	-4.71E-01	-1.39E+00	3.20E+02	4.44E+02
PHOSPHO1	9.29E+01	3.01E-14	6.17E-11	4.34E-02	-4.53E+00	-2.31E+01	4.56E-01	1.05E+01
PI3	3.03E+01	1.46E-04	2.38E-04	1.94E-01	-2.36E+00	-5.15E+00	5.63E-01	2.90E+00
PID1	1.98E+02	1.28E-02	1.37E-02	6.27E-01	-6.72E-01	-1.59E+00	8.37E+00	1.33E+01
PIDD1	9.90E+02	6.90E-04	9.23E-04	5.98E-01	-7.41E-01	-1.67E+00	3.99E+01	6.68E+01
PIGB	2.11E+02	1.24E-04	2.05E-04	7.01E-01	-5.12E-01	-1.43E+00	9.28E+00	1.32E+01
PIGC	1.27E+03	3.73E-04	5.34E-04	8.39E-01	-2.53E-01	-1.19E+00	6.17E+01	7.36E+01
PIGQ	1.19E+03	1.95E-05	4.14E-05	7.34E-01	-4.46E-01	-1.36E+00	5.40E+01	7.35E+01
PIH1D1	5.22E+02	4.47E-04	6.26E-04	7.71E-01	-3.75E-01	-1.30E+00	2.42E+01	3.14E+01
PIK3CD	4.95E+02	9.12E-13	3.74E-10	2.70E-01	-1.89E+00	-3.70E+00	1.17E+01	4.33E+01
PIK3R2	4.35E+03	2.25E-07	1.21E-06	6.36E-01	-6.52E-01	-1.57E+00	1.81E+02	2.85E+02
PIMREG	1.93E+02	8.13E-05	1.43E-04	5.48E-01	-8.67E-01	-1.82E+00	7.43E+00	1.36E+01

PIN1	2.26E+03	1.55E-05	3.39E-05	7.03E-01	-5.08E-01	-1.42E+00	9.97E+01	1.42E+02
PITPNM1	2.73E+02	4.63E-08	3.72E-07	5.18E-01	-9.50E-01	-1.93E+00	1.01E+01	1.95E+01
PKHD1	6.57E+00	3.18E-03	3.71E-03	3.04E-01	-1.72E+00	-3.28E+00	1.66E-01	5.46E-01
PKM	1.27E+04	6.45E-09	8.60E-08	5.30E-01	-9.17E-01	-1.89E+00	4.81E+02	9.08E+02
PLA2G5	9.44E+00	5.07E-04	7.01E-04	3.38E-01	-1.56E+00	-2.96E+00	2.66E-01	7.86E-01
PLCXD1	7.79E+01	4.53E-09	7.02E-08	1.51E-01	-2.72E+00	-6.61E+00	1.19E+00	7.86E+00
PLD3	8.21E+03	1.03E-05	2.43E-05	6.30E-01	-6.67E-01	-1.59E+00	3.41E+02	5.42E+02
PLEC	9.29E+03	2.58E-03	3.07E-03	7.69E-01	-3.79E-01	-1.30E+00	4.29E+02	5.58E+02
PLEK2	1.30E+02	2.70E-07	1.37E-06	3.69E-01	-1.44E+00	-2.71E+00	3.97E+00	1.08E+01
PLEKHG5	1.85E+03	2.72E-07	1.37E-06	6.44E-01	-6.34E-01	-1.55E+00	7.78E+01	1.21E+02
PLEKHM2	1.85E+03	1.42E-05	3.15E-05	8.36E-01	-2.59E-01	-1.20E+00	8.96E+01	1.07E+02
PLIN3	1.17E+03	9.61E-08	6.59E-07	5.77E-01	-7.94E-01	-1.73E+00	4.67E+01	8.10E+01
PLK3	2.19E+02	9.62E-03	1.04E-02	8.12E-01	-3.01E-01	-1.23E+00	1.04E+01	1.29E+01
PLPP7	4.36E+02	9.75E-05	1.67E-04	7.58E-01	-4.01E-01	-1.32E+00	2.00E+01	2.64E+01
PLVAP	2.21E+02	1.82E-03	2.21E-03	5.02E-01	-9.94E-01	-1.99E+00	8.07E+00	1.61E+01
PLXNA1	2.64E+03	2.87E-05	5.75E-05	6.39E-01	-6.45E-01	-1.56E+00	1.11E+02	1.73E+02
PLXNB1	1.20E+03	1.30E-06	4.47E-06	6.67E-01	-5.84E-01	-1.50E+00	5.14E+01	7.71E+01
PMM2	6.23E+02	1.67E-03	2.04E-03	8.39E-01	-2.54E-01	-1.19E+00	3.01E+01	3.60E+01
PNKD	7.67E+02	1.93E-07	1.07E-06	6.42E-01	-6.40E-01	-1.56E+00	3.21E+01	5.00E+01
PNKP	7.26E+02	4.29E-06	1.17E-05	7.11E-01	-4.91E-01	-1.41E+00	3.22E+01	4.53E+01
PNMT	3.56E+01	7.65E-06	1.90E-05	2.37E-01	-2.07E+00	-4.21E+00	7.79E-01	3.28E+00
POLR2D	3.68E+02	7.39E-07	2.93E-06	6.68E-01	-5.82E-01	-1.50E+00	1.57E+01	2.36E+01
POLR2E	1.93E+03	2.12E-08	2.06E-07	6.41E-01	-6.41E-01	-1.56E+00	8.08E+01	1.26E+02
POLR2L	4.70E+02	1.70E-04	2.71E-04	6.87E-01	-5.41E-01	-1.45E+00	2.05E+01	2.98E+01
POLR3E	6.82E+02	2.64E-04	3.94E-04	8.28E-01	-2.72E-01	-1.21E+00	3.29E+01	3.97E+01
POMGNT1	1.71E+03	1.27E-05	2.89E-05	7.73E-01	-3.72E-01	-1.29E+00	7.93E+01	1.03E+02
POP1	2.84E+02	8.38E-06	2.04E-05	6.47E-01	-6.28E-01	-1.55E+00	1.20E+01	1.86E+01
POP7	1.21E+03	2.64E-03	3.13E-03	8.03E-01	-3.16E-01	-1.24E+00	5.74E+01	7.15E+01
POU5F1	9.28E+02	2.90E-07	1.44E-06	6.58E-02	-3.93E+00	-1.52E+01	6.75E+00	1.03E+02
PPA2	2.95E+02	7.60E-03	8.28E-03	7.47E-01	-4.21E-01	-1.34E+00	1.34E+01	1.80E+01
PPCS	4.97E+02	8.57E-04	1.11E-03	7.90E-01	-3.39E-01	-1.27E+00	2.34E+01	2.96E+01
PPIA	5.14E+03	3.20E-06	9.21E-06	5.84E-01	-7.76E-01	-1.71E+00	2.04E+02	3.49E+02
PPIL1	6.12E+02	9.67E-07	3.56E-06	6.74E-01	-5.69E-01	-1.48E+00	2.64E+01	3.92E+01

PPM1F	1.45E+03	2.25E-03	2.70E-03	8.14E-01	-2.96E-01	-1.23E+00	6.96E+01	8.55E+01
PPP1CA	1.88E+03	3.76E-08	3.20E-07	5.96E-01	-7.47E-01	-1.68E+00	7.57E+01	1.27E+02
PPP1R12C	1.33E+03	4.09E-03	4.68E-03	8.74E-01	-1.95E-01	-1.14E+00	6.57E+01	7.52E+01
PPP1R14B	7.59E+02	9.19E-05	1.59E-04	6.82E-01	-5.51E-01	-1.47E+00	3.31E+01	4.85E+01
PPP1R14C	1.09E+02	1.09E-05	2.55E-05	2.90E-01	-1.78E+00	-3.44E+00	2.72E+00	9.36E+00
PPP1R16A	9.97E+02	1.80E-07	1.01E-06	5.47E-01	-8.72E-01	-1.83E+00	3.80E+01	6.96E+01
PPP1R1B	4.42E+02	1.26E-06	4.40E-06	3.13E-01	-1.68E+00	-3.20E+00	1.16E+01	3.72E+01
PPP2R1A	6.95E+03	6.23E-09	8.57E-08	6.54E-01	-6.14E-01	-1.53E+00	2.95E+02	4.52E+02
PPP2R2A	1.19E+03	3.34E-04	4.84E-04	8.76E-01	-1.91E-01	-1.14E+00	5.89E+01	6.73E+01
PPP4R1	1.94E+03	1.32E-08	1.42E-07	7.59E-01	-3.98E-01	-1.32E+00	8.91E+01	1.17E+02
PPP6R2	1.91E+03	7.11E-05	1.27E-04	7.20E-01	-4.74E-01	-1.39E+00	8.53E+01	1.18E+02
PPT2	6.07E+02	1.37E-08	1.45E-07	6.86E-01	-5.44E-01	-1.46E+00	2.64E+01	3.85E+01
PQBP1	9.82E+02	9.09E-05	1.57E-04	8.33E-01	-2.64E-01	-1.20E+00	4.76E+01	5.71E+01
PQLC3	9.76E+01	6.18E-05	1.13E-04	3.96E-01	-1.33E+00	-2.52E+00	3.06E+00	7.72E+00
PRDM1	6.41E+02	3.28E-08	2.90E-07	5.73E-01	-8.04E-01	-1.75E+00	2.54E+01	4.44E+01
PRDM14	1.60E+02	1.14E-06	4.05E-06	8.06E-03	-6.95E+00	-1.24E+02	1.47E-01	1.83E+01
PRDX1	4.47E+03	1.45E-11	1.74E-09	4.26E-01	-1.23E+00	-2.35E+00	1.46E+02	3.44E+02
PRDX2	4.56E+03	3.58E-05	7.01E-05	6.98E-01	-5.19E-01	-1.43E+00	2.01E+02	2.89E+02
PRDX5	1.35E+03	1.14E-06	4.05E-06	6.42E-01	-6.40E-01	-1.56E+00	5.67E+01	8.83E+01
PREB	8.06E+02	1.63E-06	5.33E-06	7.73E-01	-3.72E-01	-1.29E+00	3.74E+01	4.83E+01
PRELID1	1.38E+03	1.59E-04	2.56E-04	6.87E-01	-5.41E-01	-1.45E+00	6.03E+01	8.77E+01
PRELID3B	6.35E+02	2.33E-02	2.42E-02	8.51E-01	-2.32E-01	-1.17E+00	3.09E+01	3.63E+01
PRKAR1B	5.12E+02	1.65E-05	3.59E-05	7.07E-01	-5.00E-01	-1.41E+00	2.26E+01	3.20E+01
PRKRA	3.64E+02	1.46E-03	1.81E-03	8.31E-01	-2.68E-01	-1.20E+00	1.76E+01	2.11E+01
PRMT6	7.69E+02	1.84E-05	3.93E-05	6.74E-01	-5.69E-01	-1.48E+00	3.33E+01	4.94E+01
PRODH	2.67E+02	3.76E-13	3.74E-10	9.95E-02	-3.33E+00	-1.00E+01	2.79E+00	2.81E+01
PROSER3	2.97E+02	9.59E-07	3.54E-06	5.59E-01	-8.40E-01	-1.79E+00	1.15E+01	2.06E+01
PRPF19	2.27E+03	2.16E-08	2.08E-07	6.82E-01	-5.53E-01	-1.47E+00	9.87E+01	1.45E+02
PRR12	4.07E+03	2.50E-04	3.76E-04	6.94E-01	-5.26E-01	-1.44E+00	1.78E+02	2.56E+02
PRR13	4.41E+02	6.16E-04	8.31E-04	8.04E-01	-3.15E-01	-1.24E+00	2.07E+01	2.58E+01
PRR14	8.84E+02	2.13E-03	2.57E-03	8.44E-01	-2.44E-01	-1.18E+00	4.31E+01	5.10E+01
PRR3	9.17E+02	3.83E-05	7.44E-05	7.89E-01	-3.42E-01	-1.27E+00	4.30E+01	5.45E+01
PRR5	6.67E+02	1.27E-03	1.60E-03	6.91E-01	-5.34E-01	-1.45E+00	2.92E+01	4.23E+01

PRSS16	6.65E+00	3.61E-07	1.70E-06	1.18E-02	-6.40E+00	-8.44E+01	8.92E-03	7.53E-01
PRSS33	1.29E+01	7.91E-07	3.08E-06	2.90E-01	-1.79E+00	-3.45E+00	3.22E-01	1.11E+00
PRX	1.74E+02	1.25E-08	1.37E-07	3.93E-01	-1.35E+00	-2.55E+00	5.34E+00	1.36E+01
PSAT1	8.11E+02	3.09E-05	6.13E-05	4.64E-01	-1.11E+00	-2.15E+00	2.82E+01	6.07E+01
PSMA4	8.15E+02	3.78E-07	1.77E-06	5.33E-01	-9.09E-01	-1.88E+00	3.05E+01	5.73E+01
PSMB3	1.18E+03	2.03E-06	6.33E-06	6.35E-01	-6.55E-01	-1.57E+00	4.90E+01	7.71E+01
PSMB4	1.97E+03	3.50E-07	1.67E-06	6.14E-01	-7.04E-01	-1.63E+00	8.05E+01	1.31E+02
PSMB5	1.48E+03	1.27E-05	2.89E-05	6.58E-01	-6.03E-01	-1.52E+00	6.33E+01	9.61E+01
PSMC4	1.32E+03	1.41E-07	8.58E-07	6.73E-01	-5.72E-01	-1.49E+00	5.68E+01	8.45E+01
PSMD4	1.88E+03	5.86E-06	1.53E-05	7.12E-01	-4.91E-01	-1.40E+00	8.34E+01	1.17E+02
PSMD7	8.98E+02	2.50E-03	2.98E-03	8.47E-01	-2.39E-01	-1.18E+00	4.36E+01	5.15E+01
PSMD8	1.67E+03	2.20E-04	3.38E-04	7.68E-01	-3.81E-01	-1.30E+00	7.74E+01	1.01E+02
PSMD9	9.12E+02	3.30E-09	5.88E-08	4.24E-01	-1.24E+00	-2.36E+00	2.98E+01	7.02E+01
PSME3	1.90E+03	8.98E-08	6.22E-07	7.32E-01	-4.49E-01	-1.37E+00	8.55E+01	1.17E+02
PSMG3	3.07E+02	1.01E-04	1.72E-04	7.24E-01	-4.66E-01	-1.38E+00	1.37E+01	1.89E+01
PTBP1	8.21E+03	6.40E-09	8.58E-08	6.99E-01	-5.16E-01	-1.43E+00	3.63E+02	5.19E+02
PTDSS1	1.04E+03	2.63E-04	3.93E-04	7.47E-01	-4.21E-01	-1.34E+00	4.70E+01	6.30E+01
PTMA	3.61E+03	3.38E-05	6.66E-05	7.70E-01	-3.76E-01	-1.30E+00	1.67E+02	2.17E+02
PTMA-2	2.00E+04	2.01E-04	3.12E-04	7.94E-01	-3.32E-01	-1.26E+00	9.42E+02	1.19E+03
PTOV1	5.06E+03	3.95E-09	6.58E-08	6.58E-01	-6.03E-01	-1.52E+00	2.16E+02	3.28E+02
PTP4A1	1.42E+03	9.62E-04	1.24E-03	8.54E-01	-2.27E-01	-1.17E+00	6.91E+01	8.09E+01
PTPMT1	2.86E+02	4.50E-04	6.30E-04	6.94E-01	-5.26E-01	-1.44E+00	1.25E+01	1.80E+01
PTPRC	4.30E+02	5.53E-05	1.02E-04	5.47E-01	-8.70E-01	-1.83E+00	1.66E+01	3.04E+01
PTPRN2	5.54E+02	1.93E-08	1.92E-07	3.66E-01	-1.45E+00	-2.73E+00	1.62E+01	4.42E+01
PTRH2	1.64E+02	5.21E-05	9.75E-05	7.18E-01	-4.77E-01	-1.39E+00	7.32E+00	1.02E+01
PXDN	5.65E+03	9.06E-03	9.80E-03	8.00E-01	-3.22E-01	-1.25E+00	2.69E+02	3.36E+02
PXN	2.58E+03	5.81E-05	1.07E-04	6.46E-01	-6.31E-01	-1.55E+00	1.09E+02	1.68E+02
PYCR1	2.49E+03	1.86E-05	3.98E-05	6.43E-01	-6.38E-01	-1.56E+00	1.05E+02	1.63E+02
PYCR3	1.55E+03	4.01E-07	1.85E-06	6.67E-01	-5.84E-01	-1.50E+00	6.64E+01	9.96E+01
PYGO2	2.11E+03	1.56E-07	9.28E-07	6.50E-01	-6.22E-01	-1.54E+00	8.90E+01	1.37E+02
PYM1	3.56E+02	7.96E-03	8.65E-03	8.40E-01	-2.51E-01	-1.19E+00	1.73E+01	2.06E+01
QTRT1	7.45E+02	2.72E-05	5.50E-05	8.19E-01	-2.88E-01	-1.22E+00	3.58E+01	4.37E+01
R3HCC1	6.77E+02	6.85E-04	9.18E-04	8.13E-01	-2.99E-01	-1.23E+00	3.21E+01	3.95E+01

RAB11B	3.35E+03	6.11E-05	1.12E-04	8.12E-01	-3.00E-01	-1.23E+00	1.59E+02	1.96E+02
RAB15	6.68E+02	8.67E-05	1.51E-04	5.85E-01	-7.74E-01	-1.71E+00	2.66E+01	4.55E+01
RAB17	5.10E+01	1.36E-05	3.05E-05	1.72E-01	-2.54E+00	-5.82E+00	8.72E-01	5.07E+00
RAB19	1.70E+01	4.65E-03	5.26E-03	4.98E-01	-1.00E+00	-2.01E+00	5.96E-01	1.20E+00
RAB1B	3.46E+03	9.69E-10	2.84E-08	6.53E-01	-6.15E-01	-1.53E+00	1.47E+02	2.25E+02
RAB20	4.05E+01	4.23E-04	5.99E-04	5.23E-01	-9.34E-01	-1.91E+00	1.52E+00	2.90E+00
RAB34	2.26E+03	1.66E-07	9.57E-07	7.22E-01	-4.69E-01	-1.38E+00	1.01E+02	1.40E+02
RAB35	1.51E+03	1.27E-03	1.60E-03	8.79E-01	-1.87E-01	-1.14E+00	7.49E+01	8.52E+01
RAB38	1.89E+02	4.07E-03	4.66E-03	4.81E-01	-1.06E+00	-2.08E+00	6.81E+00	1.42E+01
RAB43	1.54E+03	6.56E-10	2.21E-08	5.74E-01	-8.02E-01	-1.74E+00	6.12E+01	1.07E+02
RAB7A	3.88E+03	1.61E-06	5.27E-06	8.03E-01	-3.16E-01	-1.24E+00	1.84E+02	2.29E+02
RABEP2	7.25E+02	4.37E-04	6.16E-04	8.10E-01	-3.05E-01	-1.24E+00	3.46E+01	4.28E+01
RABGGTA	3.90E+02	2.46E-04	3.70E-04	7.97E-01	-3.27E-01	-1.25E+00	1.84E+01	2.31E+01
RABGGTB	4.33E+02	1.09E-04	1.84E-04	7.48E-01	-4.19E-01	-1.34E+00	1.98E+01	2.64E+01
RAC3	5.73E+02	1.34E-06	4.55E-06	5.79E-01	-7.88E-01	-1.73E+00	2.27E+01	3.92E+01
RAD54L	5.64E+02	1.78E-05	3.84E-05	6.22E-01	-6.84E-01	-1.61E+00	2.33E+01	3.74E+01
RAD9A	5.44E+02	4.50E-09	7.02E-08	5.92E-01	-7.57E-01	-1.69E+00	2.19E+01	3.69E+01
RALY	5.21E+03	6.08E-07	2.52E-06	7.51E-01	-4.12E-01	-1.33E+00	2.38E+02	3.17E+02
RAMP1	3.03E+02	2.90E-06	8.51E-06	6.22E-01	-6.86E-01	-1.61E+00	1.24E+01	2.00E+01
RAMP3	4.01E+01	3.06E-03	3.58E-03	4.49E-01	-1.16E+00	-2.23E+00	1.37E+00	3.06E+00
RANBP1	2.14E+03	5.83E-07	2.46E-06	5.97E-01	-7.45E-01	-1.68E+00	8.62E+01	1.44E+02
RANBP3L	3.30E+01	3.23E-04	4.70E-04	3.88E-01	-1.36E+00	-2.58E+00	1.04E+00	2.67E+00
RANGAP1	1.01E+03	1.25E-07	7.89E-07	5.29E-01	-9.17E-01	-1.89E+00	3.80E+01	7.18E+01
RAP1GAP	6.83E+02	1.09E-06	3.93E-06	2.90E-01	-1.78E+00	-3.44E+00	1.69E+01	5.81E+01
RARA	2.87E+03	3.16E-03	3.69E-03	8.62E-01	-2.15E-01	-1.16E+00	1.41E+02	1.64E+02
RARS	1.24E+03	1.19E-05	2.75E-05	7.63E-01	-3.91E-01	-1.31E+00	5.75E+01	7.53E+01
RBFOX3	2.19E+02	2.57E-02	2.66E-02	6.96E-01	-5.24E-01	-1.44E+00	9.58E+00	1.38E+01
RBM10	6.01E+03	7.10E-07	2.83E-06	8.02E-01	-3.19E-01	-1.25E+00	2.84E+02	3.54E+02
RBM15B	1.61E+03	2.61E-09	5.09E-08	7.24E-01	-4.66E-01	-1.38E+00	7.22E+01	9.97E+01
RBM3	1.59E+03	1.07E-04	1.80E-04	6.85E-01	-5.47E-01	-1.46E+00	6.95E+01	1.02E+02
RBM4	3.32E+03	5.84E-07	2.46E-06	7.73E-01	-3.72E-01	-1.29E+00	1.54E+02	1.99E+02
RBP1	3.43E+03	1.45E-07	8.76E-07	5.77E-01	-7.92E-01	-1.73E+00	1.36E+02	2.35E+02
RBX1	1.50E+03	2.88E-04	4.25E-04	8.37E-01	-2.57E-01	-1.19E+00	7.25E+01	8.67E+01

RCC1L	7.91E+02	1.20E-08	1.33E-07	6.65E-01	-5.89E-01	-1.50E+00	3.38E+01	5.09E+01
RDH12	1.86E+01	1.96E-04	3.04E-04	4.39E-01	-1.19E+00	-2.28E+00	6.20E-01	1.41E+00
RELN	7.53E+02	4.42E-05	8.46E-05	4.75E-01	-1.07E+00	-2.11E+00	2.64E+01	5.55E+01
REM2	1.46E+01	1.47E-05	3.25E-05	4.37E-01	-1.20E+00	-2.29E+00	4.84E-01	1.11E+00
RENBP	1.20E+02	3.03E-03	3.55E-03	6.80E-01	-5.55E-01	-1.47E+00	5.22E+00	7.68E+00
REPIN1	2.33E+03	7.60E-06	1.89E-05	7.46E-01	-4.22E-01	-1.34E+00	1.06E+02	1.42E+02
RESP18	9.90E-01	5.73E-03	6.35E-03	1.65E-01	-2.60E+00	-6.05E+00	1.61E-02	9.72E-02
RFX4	7.92E+00	6.73E-04	9.03E-04	1.08E-01	-3.21E+00	-9.28E+00	8.44E-02	7.83E-01
RFXANK	1.12E+03	1.20E-08	1.33E-07	6.66E-01	-5.87E-01	-1.50E+00	4.80E+01	7.21E+01
RGL2	3.25E+03	2.34E-07	1.23E-06	7.76E-01	-3.66E-01	-1.29E+00	1.51E+02	1.95E+02
RGP1	2.21E+03	2.71E-03	3.21E-03	8.46E-01	-2.41E-01	-1.18E+00	1.08E+02	1.27E+02
RGS12	1.23E+03	4.00E-05	7.73E-05	7.37E-01	-4.40E-01	-1.36E+00	5.55E+01	7.54E+01
RGS20	3.27E+01	7.16E-05	1.28E-04	4.25E-01	-1.23E+00	-2.35E+00	1.08E+00	2.53E+00
RHBDD2	1.40E+03	6.63E-07	2.68E-06	6.92E-01	-5.30E-01	-1.44E+00	6.10E+01	8.81E+01
RHBG	2.40E+02	1.27E-03	1.60E-03	3.81E-01	-1.39E+00	-2.62E+00	7.43E+00	1.95E+01
RHEBL1	3.31E+01	1.60E-08	1.67E-07	1.49E-01	-2.75E+00	-6.73E+00	4.92E-01	3.31E+00
RHNO1	7.18E+02	9.22E-06	2.22E-05	7.22E-01	-4.70E-01	-1.38E+00	3.22E+01	4.45E+01
RHOD	1.40E+02	4.30E-02	4.39E-02	7.71E-01	-3.75E-01	-1.30E+00	6.54E+00	8.48E+00
RHPN1	8.35E+01	1.38E-03	1.73E-03	6.96E-01	-5.23E-01	-1.44E+00	3.62E+00	5.20E+00
RILPL2	7.39E+02	9.76E-07	3.58E-06	6.94E-01	-5.28E-01	-1.44E+00	3.25E+01	4.68E+01
RIN3	1.18E+03	2.24E-09	4.68E-08	4.85E-01	-1.04E+00	-2.06E+00	4.21E+01	8.67E+01
RING1	2.27E+03	9.74E-08	6.63E-07	7.81E-01	-3.56E-01	-1.28E+00	1.06E+02	1.36E+02
RNASEH2B	3.56E+02	5.23E-04	7.19E-04	8.28E-01	-2.72E-01	-1.21E+00	1.71E+01	2.07E+01
RND1	4.89E+02	4.81E-07	2.13E-06	4.62E-01	-1.11E+00	-2.17E+00	1.69E+01	3.66E+01
RNF10	2.12E+03	9.18E-08	6.34E-07	7.20E-01	-4.73E-01	-1.39E+00	9.47E+01	1.32E+02
RNF122	4.62E+02	1.38E-07	8.47E-07	5.37E-01	-8.98E-01	-1.86E+00	1.76E+01	3.27E+01
RNF126	7.66E+02	2.22E-07	1.20E-06	6.65E-01	-5.89E-01	-1.50E+00	3.27E+01	4.92E+01
RNF181	3.31E+02	3.83E-05	7.44E-05	7.22E-01	-4.70E-01	-1.38E+00	1.47E+01	2.03E+01
RNF215	7.07E+02	1.31E-03	1.64E-03	8.56E-01	-2.24E-01	-1.17E+00	3.46E+01	4.05E+01
RNF44	2.06E+03	9.46E-10	2.81E-08	4.93E-01	-1.02E+00	-2.03E+00	7.37E+01	1.50E+02
RNH1	1.59E+03	9.90E-07	3.62E-06	6.84E-01	-5.49E-01	-1.46E+00	6.94E+01	1.01E+02
RNPEP	1.00E+03	2.69E-04	4.01E-04	6.61E-01	-5.98E-01	-1.51E+00	4.22E+01	6.39E+01
RNPEPL1	1.20E+03	3.42E-03	3.96E-03	7.92E-01	-3.36E-01	-1.26E+00	5.64E+01	7.11E+01

ROGDI	1.24E+03	2.98E-07	1.47E-06	5.87E-01	-7.68E-01	-1.70E+00	4.96E+01	8.45E+01
RPL22L1	1.63E+03	2.05E-06	6.38E-06	6.58E-01	-6.03E-01	-1.52E+00	7.00E+01	1.06E+02
RPP25	9.06E+01	6.05E-07	2.52E-06	2.71E-01	-1.89E+00	-3.70E+00	2.12E+00	7.83E+00
RPP25L	4.82E+02	4.16E-07	1.90E-06	7.16E-01	-4.81E-01	-1.40E+00	2.14E+01	2.99E+01
RPP38	2.72E+02	3.72E-02	3.81E-02	9.03E-01	-1.47E-01	-1.11E+00	1.37E+01	1.51E+01
RPS6KA2	1.72E+03	7.31E-03	7.99E-03	8.11E-01	-3.02E-01	-1.23E+00	8.22E+01	1.01E+02
RPUSD1	3.80E+02	1.20E-06	4.23E-06	7.13E-01	-4.89E-01	-1.40E+00	1.69E+01	2.37E+01
RRM2	6.05E+02	5.16E-05	9.66E-05	6.32E-01	-6.63E-01	-1.58E+00	2.51E+01	3.97E+01
RRS1	3.71E+02	6.04E-02	6.11E-02	9.02E-01	-1.49E-01	-1.11E+00	1.86E+01	2.07E+01
RSPO1	7.73E+02	1.84E-04	2.90E-04	2.61E-01	-1.94E+00	-3.84E+00	1.76E+01	6.76E+01
RTL4	1.82E+01	2.23E-04	3.41E-04	3.31E-01	-1.59E+00	-3.02E+00	5.01E-01	1.51E+00
RTL6	1.70E+03	3.73E-06	1.05E-05	7.10E-01	-4.95E-01	-1.41E+00	7.53E+01	1.06E+02
RTN1	1.51E+02	3.28E-05	6.48E-05	2.43E-01	-2.04E+00	-4.11E+00	3.16E+00	1.30E+01
RTN4R	1.02E+02	1.25E-04	2.06E-04	4.33E-01	-1.21E+00	-2.31E+00	3.39E+00	7.82E+00
RTRAF	3.66E+03	6.99E-04	9.32E-04	7.84E-01	-3.51E-01	-1.28E+00	1.71E+02	2.18E+02
SALL1	1.77E+03	4.59E-04	6.41E-04	4.25E-01	-1.23E+00	-2.35E+00	5.75E+01	1.35E+02
SAMM50	1.52E+03	4.05E-04	5.75E-04	7.46E-01	-4.23E-01	-1.34E+00	6.89E+01	9.24E+01
SAP30BP	1.64E+03	2.82E-04	4.17E-04	8.28E-01	-2.72E-01	-1.21E+00	7.90E+01	9.54E+01
SAPCD1	4.41E+02	2.82E-04	4.17E-04	5.79E-01	-7.87E-01	-1.73E+00	1.71E+01	2.96E+01
SARAF	2.77E+03	2.33E-03	2.79E-03	7.68E-01	-3.81E-01	-1.30E+00	1.27E+02	1.66E+02
SARS2	6.16E+02	3.06E-06	8.85E-06	7.95E-01	-3.31E-01	-1.26E+00	2.90E+01	3.65E+01
SBK1	7.81E+02	1.05E-09	2.95E-08	3.71E-01	-1.43E+00	-2.69E+00	2.33E+01	6.26E+01
SBK2	5.14E+02	6.87E-06	1.74E-05	2.37E-01	-2.08E+00	-4.23E+00	1.12E+01	4.72E+01
SCAND1	2.06E+03	2.15E-06	6.61E-06	5.92E-01	-7.57E-01	-1.69E+00	8.30E+01	1.40E+02
SCCPDH	5.03E+02	2.66E-03	3.15E-03	8.02E-01	-3.19E-01	-1.25E+00	2.35E+01	2.93E+01
SCD5	1.44E+03	3.80E-02	3.89E-02	8.42E-01	-2.48E-01	-1.19E+00	7.00E+01	8.31E+01
SCG5	1.42E+03	4.65E-06	1.25E-05	6.29E-01	-6.69E-01	-1.59E+00	5.89E+01	9.36E+01
SCML4	9.89E+00	1.03E-04	1.74E-04	2.81E-01	-1.83E+00	-3.56E+00	2.44E-01	8.67E-01
SCNN1A	1.02E+03	3.71E-08	3.17E-07	1.28E-01	-2.97E+00	-7.84E+00	1.33E+01	1.04E+02
SCNN1G	2.18E+01	1.81E-03	2.21E-03	6.29E-02	-3.99E+00	-1.59E+01	1.53E-01	2.43E+00
SCRN2	6.39E+02	2.86E-03	3.37E-03	8.23E-01	-2.81E-01	-1.22E+00	3.07E+01	3.73E+01
SCUBE1	4.44E+02	5.06E-08	3.94E-07	1.84E-01	-2.44E+00	-5.43E+00	7.76E+00	4.22E+01
SDC1	5.31E+02	3.58E-07	1.69E-06	4.47E-01	-1.16E+00	-2.24E+00	1.80E+01	4.02E+01

SDE2	9.54E+02	4.79E-07	2.12E-06	4.46E-01	-1.16E+00	-2.24E+00	3.22E+01	7.22E+01
SDF2	5.84E+02	9.24E-06	2.22E-05	7.38E-01	-4.39E-01	-1.36E+00	2.64E+01	3.58E+01
SDHAF1	2.23E+02	9.53E-06	2.27E-05	6.65E-01	-5.89E-01	-1.50E+00	9.55E+00	1.44E+01
SDHAF2	6.56E+02	5.20E-08	4.03E-07	6.29E-01	-6.69E-01	-1.59E+00	2.71E+01	4.31E+01
SDHD	1.11E+03	8.77E-07	3.31E-06	7.47E-01	-4.21E-01	-1.34E+00	5.04E+01	6.75E+01
SDR39U1	3.71E+02	4.84E-03	5.45E-03	8.73E-01	-1.95E-01	-1.14E+00	1.83E+01	2.10E+01
SDR42E1	1.23E+02	1.00E-04	1.71E-04	5.12E-01	-9.67E-01	-1.95E+00	4.43E+00	8.66E+00
SEC14L1	2.43E+03	1.13E-07	7.43E-07	6.93E-01	-5.29E-01	-1.44E+00	1.07E+02	1.54E+02
SEC14L2	4.63E+02	3.95E-08	3.31E-07	2.96E-01	-1.76E+00	-3.38E+00	1.17E+01	3.96E+01
SEC14L4	6.49E+01	4.94E-04	6.86E-04	4.20E-01	-1.25E+00	-2.38E+00	2.12E+00	5.06E+00
SEC14L5	6.12E+01	1.19E-04	1.97E-04	2.40E-01	-2.06E+00	-4.16E+00	1.37E+00	5.69E+00
SEC14L6	6.49E+00	5.29E-03	5.91E-03	4.09E-02	-4.61E+00	-2.44E+01	3.09E-02	7.57E-01
SEC61G	7.97E+02	7.46E-06	1.87E-05	5.86E-01	-7.70E-01	-1.71E+00	3.16E+01	5.39E+01
SELENOF	1.44E+03	1.49E-06	4.96E-06	7.22E-01	-4.70E-01	-1.38E+00	6.45E+01	8.93E+01
SELENOO	1.09E+03	1.79E-06	5.71E-06	7.11E-01	-4.91E-01	-1.41E+00	4.84E+01	6.80E+01
SEMA3F	1.12E+03	1.65E-09	3.77E-08	4.77E-01	-1.07E+00	-2.10E+00	3.93E+01	8.25E+01
SEMA4B	1.31E+03	6.04E-06	1.56E-05	5.16E-01	-9.55E-01	-1.94E+00	4.80E+01	9.31E+01
SEMA4C	2.47E+03	5.56E-04	7.61E-04	8.34E-01	-2.62E-01	-1.20E+00	1.19E+02	1.43E+02
SEMA5B	6.27E+02	2.89E-05	5.78E-05	3.81E-01	-1.39E+00	-2.62E+00	1.91E+01	5.02E+01
SEPT9	7.84E+03	1.92E-09	4.17E-08	5.85E-01	-7.73E-01	-1.71E+00	3.13E+02	5.34E+02
SERINC2	3.70E+02	3.24E-09	5.83E-08	2.48E-01	-2.01E+00	-4.03E+00	8.21E+00	3.31E+01
SERPINA12	4.01E+00	3.11E-06	8.98E-06	2.67E-02	-5.23E+00	-3.75E+01	1.22E-02	4.58E-01
SERPINB6	3.60E+03	3.73E-07	1.75E-06	6.79E-01	-5.58E-01	-1.47E+00	1.56E+02	2.30E+02
SERPINC1	1.55E+01	1.36E-02	1.44E-02	6.32E-01	-6.63E-01	-1.58E+00	6.37E-01	1.01E+00
SERTM1	4.57E+01	1.73E-03	2.12E-03	4.47E-01	-1.16E+00	-2.24E+00	1.57E+00	3.51E+00
SESN2	9.69E+02	1.46E-09	3.39E-08	3.93E-01	-1.35E+00	-2.54E+00	2.98E+01	7.57E+01
SETD9	5.30E+01	4.39E-02	4.48E-02	7.79E-01	-3.61E-01	-1.28E+00	2.46E+00	3.15E+00
SF3B6	9.39E+02	1.09E-04	1.82E-04	7.70E-01	-3.76E-01	-1.30E+00	4.35E+01	5.65E+01
SGCG	9.73E+01	1.64E-07	9.55E-07	1.47E-01	-2.77E+00	-6.81E+00	1.38E+00	9.43E+00
SH3BP1	7.08E+02	2.06E-06	6.40E-06	5.87E-01	-7.68E-01	-1.70E+00	2.84E+01	4.83E+01
SH3GL1	4.29E+03	7.46E-07	2.94E-06	7.77E-01	-3.63E-01	-1.29E+00	2.00E+02	2.58E+02
SH3TC1	3.34E+02	1.28E-09	3.17E-08	4.09E-01	-1.29E+00	-2.44E+00	1.05E+01	2.57E+01
SHC4	3.59E+01	9.13E-04	1.18E-03	5.61E-01	-8.33E-01	-1.78E+00	1.40E+00	2.49E+00

SHF	8.85E+02	6.38E-04	8.59E-04	6.05E-01	-7.26E-01	-1.65E+00	3.64E+01	6.03E+01
SHISA3	7.27E+01	1.79E-09	3.99E-08	1.95E-01	-2.36E+00	-5.14E+00	1.34E+00	6.89E+00
SHISAL2A	7.29E+01	4.58E-06	1.24E-05	4.12E-01	-1.28E+00	-2.43E+00	2.34E+00	5.68E+00
SHROOM2	1.18E+03	6.64E-05	1.20E-04	6.12E-01	-7.07E-01	-1.63E+00	4.80E+01	7.84E+01
SIDT1	9.12E+01	1.01E-10	6.29E-09	5.44E-02	-4.20E+00	-1.84E+01	5.54E-01	1.02E+01
SIK1	8.33E+02	1.13E-02	1.21E-02	7.93E-01	-3.35E-01	-1.26E+00	3.95E+01	4.98E+01
SIRT5	8.17E+02	4.97E-03	5.58E-03	7.77E-01	-3.65E-01	-1.29E+00	3.79E+01	4.88E+01
SIRT6	2.90E+02	1.55E-06	5.11E-06	7.43E-01	-4.28E-01	-1.35E+00	1.32E+01	1.77E+01
SIT1	3.16E+01	3.95E-03	4.53E-03	5.89E-01	-7.64E-01	-1.70E+00	1.27E+00	2.16E+00
SIX5	2.81E+03	1.21E-05	2.77E-05	7.25E-01	-4.64E-01	-1.38E+00	1.26E+02	1.74E+02
SKA2	8.78E+02	1.62E-07	9.51E-07	6.98E-01	-5.18E-01	-1.43E+00	3.85E+01	5.51E+01
SLC17A9	6.33E+01	1.02E-04	1.73E-04	4.41E-01	-1.18E+00	-2.27E+00	2.12E+00	4.82E+00
SLC1A5	8.39E+02	7.02E-03	7.69E-03	7.71E-01	-3.74E-01	-1.30E+00	3.90E+01	5.05E+01
SLC20A1	1.49E+03	2.18E-08	2.09E-07	6.64E-01	-5.91E-01	-1.51E+00	6.38E+01	9.61E+01
SLC22A1	6.38E+01	6.28E-07	2.58E-06	6.81E-02	-3.88E+00	-1.47E+01	4.81E-01	7.06E+00
SLC22A17	4.77E+03	3.37E-06	9.66E-06	6.89E-01	-5.37E-01	-1.45E+00	2.09E+02	3.04E+02
SLC25A10	3.38E+03	1.00E-05	2.37E-05	4.49E-01	-1.15E+00	-2.23E+00	1.14E+02	2.54E+02
SLC25A11	1.08E+03	3.43E-05	6.75E-05	8.02E-01	-3.18E-01	-1.25E+00	5.10E+01	6.36E+01
SLC25A29	1.15E+03	2.74E-05	5.54E-05	6.90E-01	-5.35E-01	-1.45E+00	5.09E+01	7.37E+01
SLC25A3	2.08E+03	5.39E-05	1.00E-04	7.00E-01	-5.14E-01	-1.43E+00	9.21E+01	1.32E+02
SLC25A34	2.30E+02	5.19E-06	1.38E-05	6.40E-01	-6.44E-01	-1.56E+00	9.68E+00	1.51E+01
SLC25A35	1.45E+02	1.01E-10	6.29E-09	2.96E-01	-1.76E+00	-3.38E+00	3.63E+00	1.23E+01
SLC25A39	2.23E+03	2.07E-06	6.41E-06	6.75E-01	-5.66E-01	-1.48E+00	9.63E+01	1.43E+02
SLC25A5	1.90E+03	1.77E-10	8.85E-09	1.46E-01	-2.78E+00	-6.86E+00	2.79E+01	1.91E+02
SLC26A6	4.31E+02	8.22E-03	8.93E-03	8.42E-01	-2.48E-01	-1.19E+00	2.09E+01	2.48E+01
SLC27A1	2.98E+03	4.16E-03	4.75E-03	8.09E-01	-3.05E-01	-1.24E+00	1.42E+02	1.76E+02
SLC27A6	1.06E+02	7.09E-06	1.79E-05	1.02E-01	-3.30E+00	-9.83E+00	1.13E+00	1.12E+01
SLC28A1	2.60E+02	4.69E-08	3.76E-07	1.53E-01	-2.70E+00	-6.52E+00	3.98E+00	2.59E+01
SLC28A3	5.58E+00	2.82E-03	3.32E-03	4.05E-01	-1.30E+00	-2.47E+00	1.77E-01	4.38E-01
SLC29A4	9.10E+02	4.65E-06	1.25E-05	3.90E-01	-1.36E+00	-2.57E+00	2.81E+01	7.20E+01
SLC2A1	9.13E+02	1.86E-02	1.95E-02	8.07E-01	-3.09E-01	-1.24E+00	4.38E+01	5.43E+01
SLC2A4	6.94E+01	6.34E-09	8.58E-08	3.61E-01	-1.47E+00	-2.77E+00	2.02E+00	5.61E+00
SLC2A4RG	9.45E+02	6.02E-07	2.51E-06	6.78E-01	-5.62E-01	-1.48E+00	4.10E+01	6.04E+01

SLC2A8	3.16E+02	1.59E-05	3.47E-05	6.46E-01	-6.30E-01	-1.55E+00	1.33E+01	2.06E+01
SLC35F4	6.70E+01	4.15E-07	1.90E-06	4.66E-02	-4.42E+00	-2.14E+01	3.47E-01	7.44E+00
SLC35F6	6.33E+02	2.82E-05	5.67E-05	7.07E-01	-5.01E-01	-1.42E+00	2.82E+01	3.99E+01
SLC37A4	5.26E+02	3.43E-03	3.97E-03	8.36E-01	-2.58E-01	-1.20E+00	2.53E+01	3.03E+01
SLC39A4	5.78E+02	4.92E-11	4.59E-09	3.70E-02	-4.76E+00	-2.70E+01	2.49E+00	6.73E+01
SLC39A8	1.47E+02	4.79E-03	5.40E-03	4.95E-01	-1.01E+00	-2.02E+00	5.38E+00	1.09E+01
SLC3A2	1.99E+03	1.06E-09	2.95E-08	5.58E-01	-8.42E-01	-1.79E+00	7.74E+01	1.39E+02
SLC41A3	1.25E+03	5.24E-05	9.80E-05	7.66E-01	-3.85E-01	-1.31E+00	5.76E+01	7.52E+01
SLC43A3	2.63E+02	7.31E-08	5.26E-07	3.89E-01	-1.36E+00	-2.57E+00	8.21E+00	2.11E+01
SLC44A4	1.47E+02	7.70E-06	1.91E-05	1.75E-01	-2.52E+00	-5.73E+00	2.53E+00	1.45E+01
SLC45A4	7.35E+02	1.78E-07	1.01E-06	4.02E-01	-1.31E+00	-2.48E+00	2.32E+01	5.76E+01
SLC6A7	2.14E+01	9.32E-07	3.46E-06	1.86E-01	-2.42E+00	-5.37E+00	3.80E-01	2.04E+00
SLC6A9	1.58E+02	8.43E-04	1.10E-03	6.76E-01	-5.65E-01	-1.48E+00	6.87E+00	1.02E+01
SLC7A1	1.61E+03	3.58E-08	3.08E-07	5.28E-01	-9.20E-01	-1.89E+00	6.02E+01	1.14E+02
SLC7A7	5.63E+02	1.10E-04	1.84E-04	7.32E-01	-4.50E-01	-1.37E+00	2.54E+01	3.47E+01
SLC7A8	1.21E+03	2.52E-09	5.03E-08	2.14E-01	-2.23E+00	-4.68E+00	2.40E+01	1.12E+02
SLC8A3	3.38E+01	2.54E-03	3.02E-03	4.81E-01	-1.06E+00	-2.08E+00	1.20E+00	2.51E+00
SLC9A3R1	2.47E+03	4.73E-05	9.02E-05	4.80E-01	-1.06E+00	-2.08E+00	8.79E+01	1.83E+02
SLC9A5	1.01E+03	4.23E-06	1.16E-05	5.13E-01	-9.63E-01	-1.95E+00	3.72E+01	7.26E+01
SLIT1	4.52E+02	6.34E-08	4.80E-07	5.05E-02	-4.31E+00	-1.98E+01	2.57E+00	5.09E+01
SLITRK5	8.12E+01	3.95E-04	5.62E-04	5.80E-01	-7.86E-01	-1.72E+00	3.22E+00	5.55E+00
SLURP1	6.22E+00	1.32E-03	1.66E-03	1.13E-01	-3.15E+00	-8.88E+00	7.49E-02	6.66E-01
SMAD6	5.77E+02	2.64E-06	7.82E-06	6.38E-01	-6.48E-01	-1.57E+00	2.42E+01	3.80E+01
SMAD7	3.38E+02	4.21E-06	1.16E-05	6.23E-01	-6.84E-01	-1.61E+00	1.40E+01	2.25E+01
SMAGP	1.88E+02	6.65E-09	8.75E-08	4.14E-01	-1.27E+00	-2.41E+00	6.05E+00	1.46E+01
SMIM12	4.92E+02	2.27E-04	3.46E-04	7.69E-01	-3.78E-01	-1.30E+00	2.28E+01	2.96E+01
SMIM3	3.47E+02	8.38E-03	9.10E-03	7.50E-01	-4.15E-01	-1.33E+00	1.60E+01	2.13E+01
SMIM30	8.52E+01	3.96E-02	4.05E-02	8.13E-01	-3.00E-01	-1.23E+00	4.06E+00	5.00E+00
SMIM32	1.68E+02	1.25E-10	7.10E-09	4.86E-02	-4.36E+00	-2.06E+01	9.25E-01	1.90E+01
SMO	5.40E+03	2.40E-04	3.62E-04	7.98E-01	-3.26E-01	-1.25E+00	2.54E+02	3.19E+02
SMOC1	3.59E+03	2.39E-05	4.93E-05	3.82E-01	-1.39E+00	-2.62E+00	1.08E+02	2.82E+02
SMYD5	4.76E+02	1.30E-05	2.94E-05	7.47E-01	-4.20E-01	-1.34E+00	2.17E+01	2.90E+01
SNAPC4	3.19E+03	1.70E-05	3.70E-05	6.45E-01	-6.34E-01	-1.55E+00	1.34E+02	2.07E+02

SNN	2.14E+02	4.91E-06	1.31E-05	6.41E-01	-6.42E-01	-1.56E+00	8.95E+00	1.40E+01
SNRPB	1.98E+03	4.87E-10	1.75E-08	4.82E-01	-1.05E+00	-2.07E+00	6.98E+01	1.45E+02
SNX27	1.14E+01	7.97E-04	1.05E-03	2.98E-02	-5.07E+00	-3.35E+01	4.05E-02	1.36E+00
SNX7	2.59E+02	7.63E-04	1.01E-03	7.71E-01	-3.75E-01	-1.30E+00	1.20E+01	1.56E+01
SOCS1	1.24E+02	1.37E-05	3.05E-05	5.33E-01	-9.07E-01	-1.88E+00	4.69E+00	8.80E+00
SORD	1.70E+02	2.97E-07	1.47E-06	4.20E-01	-1.25E+00	-2.38E+00	5.51E+00	1.31E+01
SOSTDC1	3.89E+01	2.00E-02	2.09E-02	6.29E-01	-6.68E-01	-1.59E+00	1.64E+00	2.60E+00
SOX11	6.83E+02	1.52E-05	3.34E-05	5.74E-01	-8.02E-01	-1.74E+00	2.69E+01	4.69E+01
SOX12	4.88E+03	9.50E-05	1.63E-04	7.12E-01	-4.91E-01	-1.40E+00	2.16E+02	3.04E+02
SOX15	3.98E+02	8.00E-11	5.43E-09	2.01E-01	-2.32E+00	-4.99E+00	7.66E+00	3.82E+01
SOX17	3.16E+02	4.37E-07	1.99E-06	3.62E-01	-1.47E+00	-2.76E+00	9.40E+00	2.60E+01
SOX8	9.26E+01	8.81E-09	1.08E-07	2.28E-01	-2.13E+00	-4.38E+00	1.95E+00	8.56E+00
SPAG7	1.20E+03	1.48E-03	1.83E-03	8.23E-01	-2.82E-01	-1.22E+00	5.79E+01	7.04E+01
SPAG8	1.41E+02	2.98E-02	3.07E-02	7.64E-01	-3.89E-01	-1.31E+00	6.42E+00	8.41E+00
SPATA2L	1.75E+02	9.26E-06	2.22E-05	5.91E-01	-7.58E-01	-1.69E+00	7.02E+00	1.19E+01
SPC24	3.29E+02	2.87E-07	1.43E-06	3.64E-01	-1.46E+00	-2.75E+00	9.65E+00	2.65E+01
SPC25	3.80E+02	3.08E-06	8.90E-06	5.94E-01	-7.51E-01	-1.68E+00	1.53E+01	2.57E+01
SPIB	3.95E+01	2.86E-04	4.23E-04	4.09E-01	-1.29E+00	-2.44E+00	1.25E+00	3.06E+00
SPNS1	1.46E+03	2.36E-08	2.21E-07	7.47E-01	-4.20E-01	-1.34E+00	6.64E+01	8.89E+01
SPOCK1	7.01E+02	1.48E-04	2.40E-04	3.03E-01	-1.72E+00	-3.31E+00	1.82E+01	6.00E+01
SPOCK2	1.38E+03	1.23E-03	1.56E-03	4.42E-01	-1.18E+00	-2.26E+00	4.73E+01	1.07E+02
SPOP	1.17E+03	3.57E-09	6.15E-08	6.55E-01	-6.11E-01	-1.53E+00	4.98E+01	7.60E+01
SPRN	4.41E+02	3.37E-02	3.47E-02	6.50E-01	-6.21E-01	-1.54E+00	1.90E+01	2.92E+01
SPRY4	4.71E+02	1.92E-05	4.09E-05	5.28E-01	-9.20E-01	-1.89E+00	1.78E+01	3.38E+01
SPSB2	3.06E+02	8.41E-05	1.47E-04	6.72E-01	-5.74E-01	-1.49E+00	1.31E+01	1.95E+01
SPSB3	1.78E+03	1.43E-05	3.16E-05	7.90E-01	-3.40E-01	-1.27E+00	8.35E+01	1.06E+02
SPSB4	1.48E+02	2.80E-10	1.22E-08	1.75E-01	-2.51E+00	-5.70E+00	2.51E+00	1.43E+01
SRC	1.92E+03	7.49E-04	9.89E-04	7.92E-01	-3.36E-01	-1.26E+00	9.01E+01	1.14E+02
SRD5A1	2.24E+02	1.12E-04	1.88E-04	6.43E-01	-6.38E-01	-1.56E+00	9.32E+00	1.45E+01
SRI	7.20E+02	2.84E-03	3.35E-03	8.65E-01	-2.09E-01	-1.16E+00	3.54E+01	4.09E+01
SRSF1	3.62E+03	4.86E-03	5.47E-03	8.57E-01	-2.22E-01	-1.17E+00	1.77E+02	2.07E+02
SRSF3	1.81E+03	5.52E-05	1.02E-04	7.73E-01	-3.72E-01	-1.29E+00	8.40E+01	1.09E+02
SRSF4	4.37E+03	6.75E-05	1.22E-04	8.66E-01	-2.08E-01	-1.15E+00	2.15E+02	2.48E+02

SRSF7	2.38E+03	2.73E-09	5.23E-08	6.67E-01	-5.84E-01	-1.50E+00	1.02E+02	1.53E+02
SSBP3	1.54E+03	9.51E-04	1.23E-03	7.09E-01	-4.96E-01	-1.41E+00	6.82E+01	9.61E+01
SSLP1	2.77E+01	5.64E-03	6.27E-03	5.97E-01	-7.43E-01	-1.67E+00	1.09E+00	1.82E+00
SSTR2	3.61E+01	3.79E-04	5.40E-04	5.35E-01	-9.03E-01	-1.87E+00	1.37E+00	2.57E+00
SSTR3	2.93E+02	6.95E-07	2.79E-06	1.75E-01	-2.51E+00	-5.71E+00	4.86E+00	2.78E+01
ST14	1.26E+02	2.88E-05	5.77E-05	3.11E-01	-1.69E+00	-3.22E+00	3.31E+00	1.06E+01
STAC2	5.77E+01	2.10E-04	3.24E-04	4.70E-01	-1.09E+00	-2.13E+00	2.02E+00	4.29E+00
STARD3	1.03E+03	7.13E-05	1.27E-04	7.97E-01	-3.28E-01	-1.26E+00	4.85E+01	6.08E+01
STK32C	7.23E+01	1.42E-05	3.15E-05	4.95E-01	-1.02E+00	-2.02E+00	2.59E+00	5.24E+00
STK38	1.04E+03	8.22E-05	1.45E-04	8.26E-01	-2.76E-01	-1.21E+00	5.02E+01	6.07E+01
STK40	1.25E+03	2.94E-06	8.57E-06	7.71E-01	-3.75E-01	-1.30E+00	5.82E+01	7.55E+01
STMN2	7.52E+01	1.28E-06	4.45E-06	1.20E-01	-3.06E+00	-8.37E+00	9.29E-01	7.78E+00
STMN3	3.02E+01	1.10E-05	2.55E-05	2.27E-01	-2.14E+00	-4.41E+00	5.95E-01	2.62E+00
STOML2	1.50E+03	5.94E-07	2.49E-06	7.02E-01	-5.10E-01	-1.42E+00	6.61E+01	9.42E+01
STX3	1.20E+03	9.03E-07	3.37E-06	5.74E-01	-8.02E-01	-1.74E+00	4.73E+01	8.25E+01
STYXL1	5.76E+02	8.23E-08	5.82E-07	1.97E-01	-2.35E+00	-5.08E+00	1.06E+01	5.40E+01
SUCLA2	6.33E+02	6.39E-05	1.16E-04	7.90E-01	-3.39E-01	-1.27E+00	2.97E+01	3.75E+01
SUCLG1	1.71E+03	5.06E-06	1.34E-05	7.47E-01	-4.20E-01	-1.34E+00	7.76E+01	1.04E+02
SULT4A1	1.36E+02	5.48E-07	2.37E-06	2.10E-01	-2.25E+00	-4.75E+00	2.60E+00	1.23E+01
SUMO1	1.13E+03	5.11E-07	2.23E-06	6.90E-01	-5.35E-01	-1.45E+00	4.95E+01	7.18E+01
SUN2	5.83E+03	1.41E-05	3.13E-05	7.13E-01	-4.88E-01	-1.40E+00	2.61E+02	3.66E+02
SURF4	3.36E+03	6.49E-07	2.64E-06	7.97E-01	-3.28E-01	-1.25E+00	1.58E+02	1.98E+02
SYNDIG1L	9.86E+00	3.59E-05	7.03E-05	3.62E-01	-1.47E+00	-2.76E+00	2.91E-01	8.04E-01
SYNGR2	6.00E+02	5.07E-04	7.01E-04	6.75E-01	-5.68E-01	-1.48E+00	2.56E+01	3.80E+01
SZRD1	2.65E+03	1.19E-03	1.51E-03	8.24E-01	-2.79E-01	-1.21E+00	1.27E+02	1.55E+02
TADA3	2.74E+03	7.16E-04	9.51E-04	8.71E-01	-1.99E-01	-1.15E+00	1.35E+02	1.55E+02
TAF10	8.86E+02	1.93E-04	3.01E-04	7.78E-01	-3.62E-01	-1.28E+00	4.13E+01	5.30E+01
TAF6	2.42E+03	1.09E-08	1.25E-07	6.39E-01	-6.45E-01	-1.56E+00	1.01E+02	1.58E+02
TAF6L	4.97E+02	4.12E-08	3.40E-07	6.36E-01	-6.53E-01	-1.57E+00	2.07E+01	3.25E+01
TAF9	1.09E+03	6.39E-08	4.82E-07	6.36E-01	-6.53E-01	-1.57E+00	4.53E+01	7.13E+01
TAGLN2	2.80E+03	4.49E-02	4.58E-02	7.95E-01	-3.31E-01	-1.26E+00	1.33E+02	1.68E+02
TARBP2	6.93E+02	1.67E-06	5.40E-06	6.81E-01	-5.55E-01	-1.47E+00	3.00E+01	4.41E+01
TBC1D2	2.51E+02	1.22E-04	2.01E-04	7.15E-01	-4.83E-01	-1.40E+00	1.11E+01	1.55E+01

TBXAS1	2.55E+01	7.28E-04	9.66E-04	6.11E-01	-7.10E-01	-1.64E+00	1.05E+00	1.71E+00
TBXT	4.96E+01	5.07E-12	8.67E-10	5.80E-04	-1.08E+01	-1.72E+03	3.39E-03	5.84E+00
TCEA3	5.65E+02	4.16E-06	1.15E-05	5.82E-01	-7.82E-01	-1.72E+00	2.25E+01	3.86E+01
TCEAL4	1.80E+03	1.77E-06	5.66E-06	7.23E-01	-4.67E-01	-1.38E+00	8.07E+01	1.12E+02
TCF3	2.37E+03	1.19E-08	1.33E-07	6.04E-01	-7.27E-01	-1.65E+00	9.58E+01	1.59E+02
TCF7	8.61E+02	2.54E-09	5.03E-08	3.39E-01	-1.56E+00	-2.95E+00	2.38E+01	7.03E+01
TCL1A	7.13E+01	3.47E-03	4.01E-03	4.51E-01	-1.15E+00	-2.22E+00	2.45E+00	5.43E+00
TCL1B	4.63E+01	1.10E-02	1.17E-02	5.70E-01	-8.11E-01	-1.75E+00	1.82E+00	3.19E+00
TCTN1	7.53E+02	7.41E-05	1.32E-04	7.33E-01	-4.48E-01	-1.36E+00	3.40E+01	4.64E+01
TDH	2.55E+01	2.75E-04	4.09E-04	4.28E-01	-1.22E+00	-2.34E+00	8.25E-01	1.93E+00
TDRP	5.29E+02	1.23E-04	2.02E-04	6.14E-01	-7.03E-01	-1.63E+00	2.11E+01	3.44E+01
TEAD2	2.19E+03	4.65E-09	7.04E-08	5.38E-01	-8.95E-01	-1.86E+00	8.32E+01	1.55E+02
TEAD3	1.76E+03	4.02E-05	7.76E-05	8.02E-01	-3.18E-01	-1.25E+00	8.33E+01	1.04E+02
TEAD4	4.09E+02	3.31E-04	4.80E-04	6.69E-01	-5.80E-01	-1.50E+00	1.76E+01	2.63E+01
TECR	2.44E+03	1.12E-07	7.41E-07	6.00E-01	-7.37E-01	-1.67E+00	9.89E+01	1.65E+02
TELO2	7.05E+02	2.17E-06	6.64E-06	6.88E-01	-5.39E-01	-1.45E+00	3.07E+01	4.47E+01
TEN1	3.75E+02	4.17E-03	4.76E-03	8.35E-01	-2.60E-01	-1.20E+00	1.82E+01	2.18E+01
TEPSIN	8.00E+02	2.49E-08	2.27E-07	6.73E-01	-5.71E-01	-1.49E+00	3.44E+01	5.12E+01
TFAP2C	3.62E+02	5.13E-10	1.78E-08	2.05E-02	-5.61E+00	-4.88E+01	8.62E-01	4.21E+01
TFAP4	4.96E+02	5.18E-04	7.15E-04	7.58E-01	-3.99E-01	-1.32E+00	2.29E+01	3.02E+01
TFCP2L1	3.27E+02	1.66E-08	1.71E-07	3.34E-02	-4.91E+00	-3.00E+01	1.23E+00	3.67E+01
TFG	2.36E+03	3.00E-09	5.54E-08	6.17E-01	-6.97E-01	-1.62E+00	9.64E+01	1.56E+02
TFPT	4.26E+02	1.57E-07	9.28E-07	6.80E-01	-5.56E-01	-1.47E+00	1.84E+01	2.71E+01
TFR2	9.48E+01	5.51E-07	2.38E-06	4.01E-01	-1.32E+00	-2.50E+00	2.98E+00	7.45E+00
TGFBI	1.88E+03	7.17E-07	2.85E-06	4.49E-01	-1.15E+00	-2.23E+00	6.43E+01	1.43E+02
TGFBR3L	7.00E+01	1.91E-04	2.99E-04	5.82E-01	-7.82E-01	-1.72E+00	2.77E+00	4.77E+00
TGM3	3.08E+01	3.85E-06	1.08E-05	1.75E-01	-2.52E+00	-5.72E+00	5.25E-01	3.00E+00
THAP7	5.09E+02	1.47E-05	3.25E-05	7.85E-01	-3.49E-01	-1.27E+00	2.38E+01	3.03E+01
THBS3	2.07E+03	1.21E-06	4.26E-06	3.99E-01	-1.33E+00	-2.51E+00	6.40E+01	1.60E+02
THEM6	1.26E+02	1.49E-06	4.97E-06	3.23E-01	-1.63E+00	-3.10E+00	3.43E+00	1.06E+01
TIGD5	1.59E+03	4.86E-05	9.19E-05	7.18E-01	-4.78E-01	-1.39E+00	7.12E+01	9.92E+01
TIMM17A	4.67E+02	1.57E-04	2.53E-04	6.50E-01	-6.22E-01	-1.54E+00	1.98E+01	3.04E+01
TIMM44	6.81E+02	2.93E-06	8.55E-06	7.84E-01	-3.51E-01	-1.28E+00	3.18E+01	4.06E+01

TIMM8B	4.00E+02	2.67E-04	3.98E-04	7.43E-01	-4.28E-01	-1.35E+00	1.82E+01	2.46E+01
TIMM9	2.80E+02	2.80E-02	2.89E-02	8.71E-01	-2.00E-01	-1.15E+00	1.38E+01	1.59E+01
TIMMDC1	1.02E+02	1.43E-04	2.32E-04	6.47E-01	-6.28E-01	-1.55E+00	4.23E+00	6.53E+00
TK1	5.58E+02	1.31E-09	3.20E-08	3.58E-01	-1.48E+00	-2.79E+00	1.62E+01	4.51E+01
TLE2	1.92E+03	9.52E-09	1.13E-07	4.81E-01	-1.05E+00	-2.08E+00	6.79E+01	1.41E+02
TMA7	9.37E+02	1.30E-06	4.47E-06	6.18E-01	-6.95E-01	-1.62E+00	3.85E+01	6.23E+01
TMBIM7	1.11E+01	1.40E-02	1.48E-02	5.32E-01	-9.11E-01	-1.88E+00	4.11E-01	7.73E-01
TMCC2	8.37E+02	1.06E-08	1.21E-07	4.51E-01	-1.15E+00	-2.22E+00	2.82E+01	6.26E+01
TMEM102	6.96E+01	1.29E-05	2.92E-05	4.73E-01	-1.08E+00	-2.12E+00	2.42E+00	5.12E+00
TMEM11	7.04E+02	6.51E-05	1.18E-04	7.45E-01	-4.25E-01	-1.34E+00	3.21E+01	4.30E+01
TMEM115	1.21E+03	1.20E-05	2.77E-05	7.79E-01	-3.60E-01	-1.28E+00	5.63E+01	7.22E+01
TMEM120A	8.66E+02	8.02E-04	1.05E-03	6.70E-01	-5.79E-01	-1.49E+00	3.73E+01	5.58E+01
TMEM121B	6.35E+01	1.58E-07	9.34E-07	3.83E-01	-1.39E+00	-2.61E+00	1.92E+00	5.01E+00
TMEM126A	2.85E+02	9.61E-08	6.59E-07	4.73E-01	-1.08E+00	-2.11E+00	9.91E+00	2.09E+01
TMEM132A	1.46E+03	1.45E-08	1.53E-07	4.80E-01	-1.06E+00	-2.08E+00	5.15E+01	1.07E+02
TMEM139	2.45E+01	1.87E-04	2.94E-04	2.94E-01	-1.77E+00	-3.40E+00	6.28E-01	2.13E+00
TMEM141	1.20E+02	2.94E-04	4.32E-04	6.34E-01	-6.57E-01	-1.58E+00	5.01E+00	7.90E+00
TMEM147	1.74E+03	7.39E-03	8.05E-03	8.65E-01	-2.10E-01	-1.16E+00	8.52E+01	9.86E+01
TMEM150A	5.26E+02	4.98E-05	9.37E-05	6.67E-01	-5.85E-01	-1.50E+00	2.27E+01	3.40E+01
TMEM169	1.63E+02	2.16E-02	2.25E-02	7.61E-01	-3.94E-01	-1.31E+00	7.51E+00	9.86E+00
TMEM175	1.52E+03	7.56E-07	2.96E-06	7.78E-01	-3.62E-01	-1.28E+00	7.09E+01	9.11E+01
TMEM176A	6.19E+02	1.75E-05	3.78E-05	5.14E-01	-9.61E-01	-1.95E+00	2.28E+01	4.44E+01
TMEM179	1.23E+01	4.99E-03	5.60E-03	4.49E-01	-1.15E+00	-2.23E+00	4.14E-01	9.22E-01
TMEM182	3.19E+01	5.67E-03	6.29E-03	5.69E-01	-8.13E-01	-1.76E+00	1.23E+00	2.16E+00
TMEM183A	9.54E+02	8.75E-05	1.52E-04	8.01E-01	-3.21E-01	-1.25E+00	4.51E+01	5.63E+01
TMEM185B	3.06E+02	2.47E-05	5.07E-05	7.85E-01	-3.50E-01	-1.27E+00	1.44E+01	1.83E+01
TMEM190	1.43E+03	6.64E-08	4.88E-07	1.09E-01	-3.20E+00	-9.17E+00	1.60E+01	1.47E+02
TMEM200C	2.95E+01	2.78E-03	3.28E-03	4.11E-01	-1.28E+00	-2.43E+00	9.43E-01	2.29E+00
TMEM208	3.57E+02	1.17E-04	1.94E-04	7.52E-01	-4.12E-01	-1.33E+00	1.63E+01	2.16E+01
TMEM221	5.76E+01	8.51E-05	1.49E-04	5.08E-01	-9.78E-01	-1.97E+00	2.11E+00	4.16E+00
TMEM222	7.52E+02	3.05E-08	2.71E-07	7.21E-01	-4.72E-01	-1.39E+00	3.36E+01	4.66E+01
TMEM223	3.82E+02	4.22E-06	1.16E-05	6.90E-01	-5.36E-01	-1.45E+00	1.67E+01	2.42E+01
TMEM229B	8.97E+02	3.89E-03	4.47E-03	6.60E-01	-5.99E-01	-1.51E+00	3.81E+01	5.77E+01

TMEM231	5.58E+02	8.32E-06	2.03E-05	5.97E-01	-7.44E-01	-1.68E+00	2.22E+01	3.71E+01
TMEM238	7.97E+01	6.51E-03	7.16E-03	7.43E-01	-4.29E-01	-1.35E+00	3.60E+00	4.85E+00
TMEM25	8.68E+02	2.31E-04	3.51E-04	7.50E-01	-4.15E-01	-1.33E+00	3.97E+01	5.29E+01
TMEM265	1.46E+02	3.94E-05	7.64E-05	6.80E-01	-5.56E-01	-1.47E+00	6.34E+00	9.32E+00
TMEM30C	4.77E+00	1.30E-02	1.38E-02	5.18E-01	-9.50E-01	-1.93E+00	1.73E-01	3.34E-01
TMEM37	1.53E+02	4.88E-07	2.15E-06	4.72E-01	-1.08E+00	-2.12E+00	5.38E+00	1.14E+01
TMEM39B	5.28E+02	6.09E-06	1.57E-05	7.63E-01	-3.90E-01	-1.31E+00	2.44E+01	3.20E+01
TMEM53	2.37E+02	2.12E-06	6.54E-06	6.41E-01	-6.43E-01	-1.56E+00	9.91E+00	1.55E+01
TMEM59L	2.10E+03	1.75E-08	1.77E-07	9.72E-02	-3.36E+00	-1.03E+01	2.11E+01	2.18E+02
TMEM63C	3.00E+01	7.25E-06	1.83E-05	2.50E-01	-2.00E+00	-4.00E+00	6.58E-01	2.63E+00
TMEM88	3.58E+02	6.42E-06	1.64E-05	6.40E-01	-6.43E-01	-1.56E+00	1.51E+01	2.35E+01
TMEM8A	1.30E+03	2.91E-10	1.24E-08	2.85E-01	-1.81E+00	-3.51E+00	3.19E+01	1.12E+02
TMEM9	2.33E+03	1.62E-08	1.67E-07	5.79E-01	-7.87E-01	-1.73E+00	9.21E+01	1.59E+02
TMEM94	5.60E+03	4.17E-07	1.90E-06	5.70E-01	-8.10E-01	-1.75E+00	2.18E+02	3.83E+02
TMEM97	1.51E+03	3.11E-03	3.64E-03	7.61E-01	-3.93E-01	-1.31E+00	6.96E+01	9.15E+01
TMEM98	1.26E+03	4.71E-03	5.31E-03	8.43E-01	-2.47E-01	-1.19E+00	6.12E+01	7.27E+01
TMPRSS2	8.34E+01	1.40E-09	3.35E-08	8.08E-02	-3.63E+00	-1.24E+01	7.22E-01	8.94E+00
TMSB10	1.70E+04	4.64E-06	1.25E-05	6.76E-01	-5.66E-01	-1.48E+00	7.31E+02	1.08E+03
TMUB1	5.08E+02	2.78E-05	5.59E-05	7.26E-01	-4.61E-01	-1.38E+00	2.28E+01	3.14E+01
TNKS1BP1	8.09E+03	4.82E-05	9.14E-05	7.08E-01	-4.99E-01	-1.41E+00	3.60E+02	5.09E+02
TOE1	5.77E+02	3.70E-04	5.30E-04	8.51E-01	-2.33E-01	-1.17E+00	2.81E+01	3.30E+01
TOM1	1.03E+03	2.39E-07	1.25E-06	7.69E-01	-3.80E-01	-1.30E+00	4.78E+01	6.22E+01
TOMM40	1.08E+03	6.63E-08	4.88E-07	5.52E-01	-8.57E-01	-1.81E+00	4.15E+01	7.51E+01
TOR4A	1.18E+02	2.43E-06	7.28E-06	4.72E-01	-1.08E+00	-2.12E+00	4.18E+00	8.86E+00
TP53	2.11E+03	5.71E-07	2.42E-06	7.02E-01	-5.11E-01	-1.43E+00	9.37E+01	1.33E+02
TP53I11	2.22E+03	9.19E-06	2.21E-05	6.48E-01	-6.25E-01	-1.54E+00	9.37E+01	1.44E+02
TPC3	4.92E+01	2.85E-06	8.39E-06	2.95E-01	-1.76E+00	-3.39E+00	1.26E+00	4.27E+00
TPI1	4.12E+03	1.19E-04	1.97E-04	7.23E-01	-4.67E-01	-1.38E+00	1.86E+02	2.58E+02
TPM3	5.71E+03	1.01E-05	2.39E-05	7.48E-01	-4.19E-01	-1.34E+00	2.61E+02	3.49E+02
TPPP	3.97E+02	9.14E-07	3.40E-06	2.48E-01	-2.01E+00	-4.03E+00	8.93E+00	3.60E+01
TRABD	1.89E+03	4.61E-05	8.81E-05	8.27E-01	-2.74E-01	-1.21E+00	9.08E+01	1.10E+02
TRAF3IP2	4.53E+02	1.00E-07	6.75E-07	6.04E-01	-7.27E-01	-1.65E+00	1.85E+01	3.07E+01
TRAF4	9.36E+02	2.89E-08	2.60E-07	4.79E-01	-1.06E+00	-2.09E+00	3.31E+01	6.90E+01

TRAPPC12	1.12E+03	9.88E-09	1.16E-07	5.56E-01	-8.48E-01	-1.80E+00	4.31E+01	7.76E+01
TRAPPC6A	1.17E+02	2.19E-03	2.64E-03	6.82E-01	-5.52E-01	-1.47E+00	5.07E+00	7.43E+00
TRIAP1	3.97E+02	2.82E-03	3.32E-03	8.19E-01	-2.89E-01	-1.22E+00	1.91E+01	2.33E+01
TRIM13	5.50E+02	7.54E-08	5.39E-07	6.15E-01	-7.01E-01	-1.63E+00	2.25E+01	3.66E+01
TRIM32	6.98E+02	2.80E-03	3.30E-03	7.56E-01	-4.03E-01	-1.32E+00	3.19E+01	4.22E+01
TRIM46	2.35E+02	1.53E-07	9.17E-07	2.60E-01	-1.94E+00	-3.84E+00	5.37E+00	2.06E+01
TRIM8	1.27E+03	3.52E-07	1.67E-06	6.77E-01	-5.62E-01	-1.48E+00	5.54E+01	8.18E+01
TRIML2	3.34E+01	9.04E-06	2.19E-05	1.66E-02	-5.91E+00	-6.03E+01	6.24E-02	3.76E+00
TRIP6	1.34E+03	3.48E-04	5.03E-04	8.08E-01	-3.07E-01	-1.24E+00	6.39E+01	7.91E+01
TRIR	1.99E+03	1.83E-06	5.81E-06	7.29E-01	-4.55E-01	-1.37E+00	8.97E+01	1.23E+02
TRMT61A	5.48E+02	6.31E-02	6.37E-02	9.06E-01	-1.42E-01	-1.10E+00	2.76E+01	3.05E+01
TRMU	7.77E+02	2.10E-04	3.24E-04	7.67E-01	-3.83E-01	-1.30E+00	3.60E+01	4.69E+01
TROAP	6.89E+02	3.43E-05	6.75E-05	5.72E-01	-8.05E-01	-1.75E+00	2.71E+01	4.74E+01
TSC22D4	2.05E+03	8.43E-03	9.14E-03	9.06E-01	-1.43E-01	-1.10E+00	1.03E+02	1.14E+02
TSEN54	6.78E+02	2.34E-05	4.83E-05	7.43E-01	-4.29E-01	-1.35E+00	3.08E+01	4.14E+01
TSNARE1	5.88E+02	5.90E-07	2.48E-06	6.12E-01	-7.08E-01	-1.63E+00	2.40E+01	3.92E+01
TSPAN11	6.62E+02	6.05E-06	1.56E-05	6.35E-01	-6.55E-01	-1.57E+00	2.76E+01	4.34E+01
TSPAN14	1.69E+03	2.13E-04	3.27E-04	7.70E-01	-3.77E-01	-1.30E+00	7.84E+01	1.02E+02
TSPAN15	3.62E+02	2.63E-03	3.12E-03	7.17E-01	-4.81E-01	-1.40E+00	1.62E+01	2.26E+01
TSPAN31	1.24E+03	2.74E-05	5.54E-05	7.97E-01	-3.26E-01	-1.25E+00	5.88E+01	7.37E+01
TSPAN6	1.82E+03	9.85E-09	1.16E-07	5.00E-01	-9.99E-01	-2.00E+00	6.56E+01	1.31E+02
TSPAN9	2.19E+03	1.27E-03	1.60E-03	8.45E-01	-2.43E-01	-1.18E+00	1.07E+02	1.26E+02
TSR3	3.56E+02	7.56E-04	9.97E-04	7.90E-01	-3.39E-01	-1.27E+00	1.67E+01	2.12E+01
TSSC4	5.03E+02	2.33E-04	3.53E-04	7.62E-01	-3.92E-01	-1.31E+00	2.31E+01	3.04E+01
TSTD1	1.60E+02	2.29E-05	4.75E-05	5.95E-01	-7.49E-01	-1.68E+00	6.41E+00	1.08E+01
TTC30B	5.99E+01	2.63E-03	3.12E-03	6.44E-01	-6.35E-01	-1.55E+00	2.47E+00	3.83E+00
TTC36	7.45E+01	1.04E-06	3.78E-06	3.76E-01	-1.41E+00	-2.66E+00	2.21E+00	5.87E+00
TTC6	1.81E+01	3.33E-03	3.87E-03	3.62E-01	-1.47E+00	-2.76E+00	5.06E-01	1.40E+00
TTC9C	7.79E+02	2.25E-04	3.44E-04	6.61E-01	-5.98E-01	-1.51E+00	3.34E+01	5.05E+01
TTYH2	4.64E+02	6.18E-05	1.13E-04	4.59E-01	-1.12E+00	-2.18E+00	1.62E+01	3.52E+01
TTYH3	8.72E+03	2.13E-05	4.45E-05	6.38E-01	-6.47E-01	-1.57E+00	3.64E+02	5.71E+02
TUBA1B	2.17E+04	1.00E-07	6.75E-07	6.09E-01	-7.14E-01	-1.64E+00	8.84E+02	1.45E+03
TUBB	2.51E+04	2.13E-07	1.16E-06	6.67E-01	-5.85E-01	-1.50E+00	1.08E+03	1.62E+03

TUFT1	4.81E+02	1.45E-03	1.80E-03	7.23E-01	-4.68E-01	-1.38E+00	2.17E+01	3.00E+01
TXN	1.18E+03	9.14E-05	1.58E-04	6.42E-01	-6.39E-01	-1.56E+00	4.94E+01	7.69E+01
TXN2	1.22E+03	2.41E-08	2.23E-07	6.66E-01	-5.87E-01	-1.50E+00	5.26E+01	7.90E+01
TXNDC17	1.94E+02	6.90E-09	9.01E-08	4.19E-01	-1.25E+00	-2.39E+00	6.27E+00	1.49E+01
TXNRD2	4.88E+02	2.85E-05	5.72E-05	8.00E-01	-3.22E-01	-1.25E+00	2.31E+01	2.89E+01
TYRO3	3.02E+03	2.42E-06	7.27E-06	6.04E-01	-7.27E-01	-1.66E+00	1.22E+02	2.02E+02
UAP1L1	1.03E+03	2.06E-05	4.34E-05	6.64E-01	-5.90E-01	-1.51E+00	4.44E+01	6.68E+01
UBALD2	6.79E+02	6.10E-04	8.26E-04	7.51E-01	-4.13E-01	-1.33E+00	3.13E+01	4.16E+01
UBE2C	7.19E+02	8.36E-07	3.19E-06	4.25E-01	-1.24E+00	-2.36E+00	2.35E+01	5.54E+01
UBE2E2	5.98E+02	4.58E-07	2.05E-06	6.77E-01	-5.63E-01	-1.48E+00	2.60E+01	3.84E+01
UBIAD1	3.98E+02	1.94E-04	3.03E-04	7.66E-01	-3.85E-01	-1.31E+00	1.83E+01	2.39E+01
UBL5	1.01E+03	1.43E-06	4.80E-06	6.89E-01	-5.37E-01	-1.45E+00	4.39E+01	6.36E+01
UBL7	9.87E+02	1.18E-05	2.73E-05	8.00E-01	-3.22E-01	-1.25E+00	4.66E+01	5.82E+01
UCKL1	1.22E+03	2.55E-04	3.83E-04	8.65E-01	-2.09E-01	-1.16E+00	6.03E+01	6.97E+01
UGT1A1	1.22E+01	2.81E-04	4.16E-04	1.82E-01	-2.46E+00	-5.49E+00	2.22E-01	1.22E+00
UGT1A6	3.07E+00	5.27E-05	9.83E-05	2.66E-04	-1.19E+01	-3.76E+03	1.00E-04	3.76E-01
UHRF1	9.68E+02	1.14E-06	4.07E-06	4.84E-01	-1.05E+00	-2.06E+00	3.41E+01	7.05E+01
ULK1	2.85E+03	7.52E-09	9.50E-08	6.28E-01	-6.71E-01	-1.59E+00	1.18E+02	1.88E+02
UMPS	4.83E+02	5.09E-05	9.58E-05	7.99E-01	-3.25E-01	-1.25E+00	2.27E+01	2.85E+01
UNC119	2.56E+03	2.58E-07	1.32E-06	5.44E-01	-8.79E-01	-1.84E+00	9.78E+01	1.80E+02
UNC13D	4.01E+02	5.71E-04	7.79E-04	6.09E-01	-7.16E-01	-1.64E+00	1.65E+01	2.71E+01
UNC93B1	9.15E+02	1.83E-05	3.93E-05	7.67E-01	-3.83E-01	-1.30E+00	4.22E+01	5.51E+01
UPK1A	1.88E+01	4.87E-05	9.19E-05	4.66E-01	-1.10E+00	-2.14E+00	6.53E-01	1.40E+00
UQCR10	1.02E+03	5.55E-08	4.26E-07	6.25E-01	-6.79E-01	-1.60E+00	4.19E+01	6.71E+01
UQCR11	4.06E+02	2.67E-03	3.16E-03	7.72E-01	-3.72E-01	-1.29E+00	1.88E+01	2.44E+01
UQCRFS1	9.21E+02	2.85E-08	2.59E-07	4.93E-01	-1.02E+00	-2.03E+00	3.31E+01	6.72E+01
UQCRQ	4.48E+02	1.69E-02	1.78E-02	8.40E-01	-2.51E-01	-1.19E+00	2.18E+01	2.59E+01
USE1	7.55E+02	7.34E-04	9.71E-04	8.11E-01	-3.02E-01	-1.23E+00	3.59E+01	4.43E+01
USF1	1.25E+03	8.12E-07	3.11E-06	7.78E-01	-3.62E-01	-1.28E+00	5.84E+01	7.51E+01
USP43	1.15E+03	4.66E-07	2.07E-06	2.55E-01	-1.97E+00	-3.92E+00	2.57E+01	1.01E+02
UXT	2.59E+02	1.04E-02	1.12E-02	8.10E-01	-3.04E-01	-1.23E+00	1.23E+01	1.52E+01
VANGL2	1.12E+03	7.51E-05	1.33E-04	6.27E-01	-6.74E-01	-1.60E+00	4.63E+01	7.39E+01
VAPB	1.48E+03	1.41E-07	8.58E-07	7.55E-01	-4.05E-01	-1.32E+00	6.80E+01	9.00E+01

VASH1	2.71E+03	1.53E-04	2.47E-04	7.46E-01	-4.22E-01	-1.34E+00	1.23E+02	1.65E+02
VASP	2.17E+03	5.70E-11	4.67E-09	5.46E-01	-8.73E-01	-1.83E+00	8.31E+01	1.52E+02
VDAC2	1.97E+03	5.94E-04	8.06E-04	7.81E-01	-3.57E-01	-1.28E+00	9.18E+01	1.18E+02
VEGFB	1.19E+03	6.82E-06	1.73E-05	8.11E-01	-3.03E-01	-1.23E+00	5.67E+01	6.99E+01
VEGFC	4.93E+02	1.98E-06	6.19E-06	4.35E-01	-1.20E+00	-2.30E+00	1.63E+01	3.73E+01
VIL1	3.85E+01	1.06E-04	1.79E-04	2.25E-01	-2.15E+00	-4.44E+00	8.21E-01	3.64E+00
VPS18	5.88E+02	1.86E-03	2.26E-03	8.30E-01	-2.68E-01	-1.20E+00	2.82E+01	3.40E+01
VPS25	2.19E+03	3.43E-07	1.65E-06	6.86E-01	-5.43E-01	-1.46E+00	9.55E+01	1.39E+02
VPS72	8.68E+02	9.12E-03	9.86E-03	8.72E-01	-1.97E-01	-1.15E+00	4.30E+01	4.93E+01
VPS9D1	5.13E+02	2.91E-04	4.28E-04	7.65E-01	-3.87E-01	-1.31E+00	2.36E+01	3.08E+01
VSX2	2.66E+01	3.67E-06	1.04E-05	1.05E-02	-6.58E+00	-9.56E+01	3.16E-02	3.03E+00
VWCE	2.45E+02	1.21E-09	3.02E-08	3.14E-01	-1.67E+00	-3.18E+00	6.53E+00	2.08E+01
WBP1	1.67E+03	1.84E-09	4.05E-08	5.37E-01	-8.96E-01	-1.86E+00	6.35E+01	1.18E+02
WBP2	2.44E+03	8.33E-06	2.03E-05	7.81E-01	-3.56E-01	-1.28E+00	1.14E+02	1.46E+02
WC1.3	3.77E+00	4.96E-02	5.04E-02	3.07E-01	-1.70E+00	-3.25E+00	1.00E-01	3.26E-01
WDR13	2.41E+03	1.55E-06	5.11E-06	7.75E-01	-3.67E-01	-1.29E+00	1.12E+02	1.45E+02
WDR6	7.60E+03	4.25E-09	6.76E-08	5.35E-01	-9.02E-01	-1.87E+00	2.87E+02	5.36E+02
WDR83	3.68E+02	1.28E-05	2.90E-05	7.26E-01	-4.63E-01	-1.38E+00	1.65E+01	2.27E+01
WFDC3	9.28E+01	4.37E-05	8.39E-05	4.67E-01	-1.10E+00	-2.14E+00	3.11E+00	6.67E+00
WFIKKN1	4.15E+01	1.24E-07	7.87E-07	3.10E-01	-1.69E+00	-3.23E+00	1.07E+00	3.46E+00
WIZ	2.88E+03	4.89E-08	3.88E-07	6.82E-01	-5.52E-01	-1.47E+00	1.25E+02	1.83E+02
WNK4	1.31E+03	1.35E-05	3.02E-05	4.65E-01	-1.11E+00	-2.15E+00	4.49E+01	9.66E+01
WNT10A	2.82E+02	9.60E-06	2.29E-05	1.66E-01	-2.59E+00	-6.04E+00	4.53E+00	2.74E+01
WNT5B	1.44E+02	8.95E-10	2.78E-08	2.29E-01	-2.13E+00	-4.37E+00	3.00E+00	1.31E+01
WNT6	1.80E+03	5.75E-08	4.40E-07	2.34E-01	-2.10E+00	-4.27E+00	3.77E+01	1.61E+02
WNT9B	4.96E+01	2.07E-08	2.03E-07	1.38E-01	-2.86E+00	-7.25E+00	6.90E-01	5.00E+00
WRAP53	5.53E+02	2.59E-07	1.32E-06	7.31E-01	-4.52E-01	-1.37E+00	2.50E+01	3.42E+01
WT1	5.42E+03	3.01E-02	3.11E-02	7.68E-01	-3.80E-01	-1.30E+00	2.51E+02	3.26E+02
XKR4	2.89E+01	7.20E-05	1.28E-04	2.11E-01	-2.25E+00	-4.74E+00	5.82E-01	2.76E+00
XKR7	4.81E+00	7.03E-03	7.70E-03	1.66E-01	-2.59E+00	-6.01E+00	7.82E-02	4.70E-01
XPNPEP1	1.06E+03	1.45E-09	3.39E-08	6.36E-01	-6.52E-01	-1.57E+00	4.42E+01	6.94E+01
XPNPEP2	2.55E+02	1.02E-08	1.19E-07	2.07E-01	-2.27E+00	-4.83E+00	5.01E+00	2.42E+01
XR_001494160.2	5.63E+01	1.59E-03	1.96E-03	6.80E-01	-5.56E-01	-1.47E+00	2.43E+00	3.58E+00

KR_001494187.2	1.48E+02	1.31E-04	2.15E-04	5.96E-01	-7.47E-01	-1.68E+00	5.95E+00	9.98E+00
KR_001494238.2	3.30E+00	4.26E-01	4.27E-01	2.89E-01	-1.79E+00	-3.45E+00	8.51E-02	2.94E-01
KR_001494285.2	8.67E+00	1.88E-01	1.89E-01	2.29E-01	-2.12E+00	-4.36E+00	1.90E-01	8.29E-01
KR_001494523.2	4.25E+00	3.76E-02	3.86E-02	3.59E-01	-1.48E+00	-2.78E+00	1.22E-01	3.40E-01
KR_001495283.2	2.43E+00	3.80E-02	3.89E-02	3.88E-01	-1.37E+00	-2.58E+00	7.55E-02	1.94E-01
KR_001495345.2	4.22E+02	1.01E-01	1.02E-01	8.72E-01	-1.97E-01	-1.15E+00	2.09E+01	2.40E+01
KR_001495359.2	6.51E+01	6.31E-03	6.96E-03	8.21E-01	-2.85E-01	-1.22E+00	3.12E+00	3.80E+00
KR_001500534.2	2.35E+03	1.91E-06	6.01E-06	6.51E-01	-6.19E-01	-1.54E+00	9.97E+01	1.53E+02
KR_001500577.2	3.02E+01	2.59E-03	3.08E-03	5.72E-01	-8.07E-01	-1.75E+00	1.19E+00	2.09E+00
KR_001501856.2	1.78E+01	4.92E-05	9.27E-05	3.17E-01	-1.66E+00	-3.16E+00	4.67E-01	1.47E+00
KR_003029376.1	1.63E+00	2.05E-03	2.48E-03	2.71E-01	-1.88E+00	-3.68E+00	3.98E-02	1.47E-01
KR_003029471.1	2.36E+01	9.42E-03	1.02E-02	4.40E-01	-1.19E+00	-2.28E+00	7.85E-01	1.79E+00
KR_003029479.1	3.48E+01	9.39E-04	1.21E-03	6.51E-01	-6.19E-01	-1.54E+00	1.46E+00	2.25E+00
KR_003029507.1	4.19E+00	1.07E-01	1.07E-01	4.72E-01	-1.08E+00	-2.12E+00	1.50E-01	3.17E-01
KR_003029761.1	1.72E+02	4.35E-02	4.44E-02	7.93E-01	-3.34E-01	-1.26E+00	8.06E+00	1.02E+01
KR_003029836.1	6.10E+00	1.86E-07	1.05E-06	1.11E-02	-6.49E+00	-9.00E+01	8.11E-03	7.30E-01
KR_003029934.1	5.97E+02	5.52E-07	2.38E-06	5.84E-01	-7.75E-01	-1.71E+00	2.37E+01	4.06E+01
KR_003030031.1	5.24E+01	5.73E-04	7.80E-04	5.19E-01	-9.46E-01	-1.93E+00	1.90E+00	3.65E+00
KR_003030051.1	1.02E+01	1.81E-03	2.21E-03	1.18E-01	-3.08E+00	-8.46E+00	1.25E-01	1.06E+00
KR_003030120.1	1.34E+01	2.73E-04	4.07E-04	2.82E-01	-1.83E+00	-3.55E+00	3.18E-01	1.13E+00
KR_003030162.1	1.32E+02	2.37E-04	3.59E-04	7.54E-01	-4.07E-01	-1.33E+00	6.07E+00	8.05E+00
KR_003030168.1	1.02E+02	3.46E-06	9.86E-06	4.40E-01	-1.18E+00	-2.27E+00	3.43E+00	7.79E+00
KR_003030238.1	2.59E+02	1.38E-03	1.72E-03	6.77E-01	-5.62E-01	-1.48E+00	1.12E+01	1.65E+01
KR_003030302.1	2.27E+01	1.77E-07	1.01E-06	8.75E-02	-3.51E+00	-1.14E+01	2.13E-01	2.43E+00
KR_003030324.1	2.42E+02	3.44E-09	6.02E-08	1.97E-01	-2.35E+00	-5.08E+00	4.62E+00	2.35E+01
KR_003030341.1	1.47E+02	1.02E-04	1.74E-04	3.73E-01	-1.42E+00	-2.68E+00	4.39E+00	1.18E+01
KR_003030429.1	5.29E+01	1.80E-04	2.85E-04	4.67E-01	-1.10E+00	-2.14E+00	1.83E+00	3.92E+00
KR_003030478.1	6.40E+00	1.61E-03	1.98E-03	3.33E-01	-1.59E+00	-3.01E+00	1.73E-01	5.20E-01
KR_003030498.1	2.63E+01	7.14E-03	7.82E-03	7.19E-01	-4.76E-01	-1.39E+00	1.18E+00	1.64E+00
KR_003030651.1	6.82E+01	1.76E-03	2.15E-03	2.86E-01	-1.81E+00	-3.50E+00	1.72E+00	6.03E+00
KR_003030652.1	1.16E+02	2.50E-03	2.98E-03	2.57E-01	-1.96E+00	-3.89E+00	2.74E+00	1.07E+01
KR_003030653.1	2.40E+02	7.59E-04	1.00E-03	1.94E-01	-2.37E+00	-5.16E+00	4.52E+00	2.33E+01
KR_003030744.1	2.61E+01	8.28E-04	1.08E-03	3.67E-01	-1.45E+00	-2.73E+00	7.70E-01	2.10E+00

XR_003030817.1	3.78E+01	1.37E-04	2.23E-04	5.59E-01	-8.39E-01	-1.79E+00	1.46E+00	2.62E+00
XR_003030820.1	4.17E+01	1.15E-07	7.49E-07	3.42E-01	-1.55E+00	-2.93E+00	1.17E+00	3.43E+00
XR_003030867.1	9.34E+02	7.17E-05	1.28E-04	7.36E-01	-4.42E-01	-1.36E+00	4.23E+01	5.75E+01
XR_003030868.1	1.60E+02	1.77E-05	3.82E-05	5.90E-01	-7.61E-01	-1.69E+00	6.37E+00	1.08E+01
XR_003030928.1	6.40E+01	5.89E-04	8.01E-04	6.44E-01	-6.34E-01	-1.55E+00	2.68E+00	4.15E+00
XR_003030959.1	1.41E+02	3.20E-03	3.72E-03	7.64E-01	-3.89E-01	-1.31E+00	6.48E+00	8.48E+00
XR_003030982.1	2.16E+01	5.08E-02	5.16E-02	6.63E-01	-5.94E-01	-1.51E+00	9.00E-01	1.36E+00
XR_003031000.1	2.35E+01	7.24E-03	7.92E-03	2.55E-01	-1.97E+00	-3.93E+00	5.04E-01	1.98E+00
XR_003031003.1	2.91E+00	3.52E-02	3.62E-02	3.79E-01	-1.40E+00	-2.64E+00	8.32E-02	2.19E-01
XR_003031018.1	3.67E+00	4.18E-03	4.76E-03	1.73E-01	-2.54E+00	-5.80E+00	6.12E-02	3.55E-01
XR_003031179.1	7.08E+01	8.53E-04	1.11E-03	6.38E-01	-6.48E-01	-1.57E+00	2.98E+00	4.67E+00
XR_003031266.1	5.01E+01	1.06E-04	1.79E-04	4.99E-01	-1.00E+00	-2.00E+00	1.78E+00	3.57E+00
XR_003031361.1	3.67E+01	5.54E-03	6.16E-03	2.31E-01	-2.12E+00	-4.33E+00	7.78E-01	3.37E+00
XR_003031495.1	3.29E+00	8.09E-06	1.99E-05	4.29E-02	-4.54E+00	-2.33E+01	1.53E-02	3.56E-01
XR_003031543.1	7.89E+00	5.53E-04	7.57E-04	2.89E-01	-1.79E+00	-3.47E+00	1.96E-01	6.81E-01
XR_003031596.1	1.34E+01	3.18E-04	4.63E-04	3.39E-01	-1.56E+00	-2.95E+00	3.66E-01	1.08E+00
XR_003031713.1	1.34E+02	9.61E-06	2.29E-05	2.70E-02	-5.21E+00	-3.71E+01	4.07E-01	1.51E+01
XR_003031756.1	2.96E+01	7.11E-05	1.27E-04	4.98E-01	-1.01E+00	-2.01E+00	1.09E+00	2.18E+00
XR_003031802.1	3.25E+01	9.84E-04	1.26E-03	3.16E-01	-1.66E+00	-3.17E+00	8.54E-01	2.70E+00
XR_003031863.1	1.83E+01	7.09E-07	2.83E-06	7.97E-03	-6.97E+00	-1.25E+02	1.68E-02	2.11E+00
XR_003031970.1	5.53E+00	1.41E-02	1.50E-02	4.81E-01	-1.06E+00	-2.08E+00	1.94E-01	4.04E-01
XR_003032146.1	1.64E+01	2.52E-05	5.16E-05	3.87E-01	-1.37E+00	-2.58E+00	5.00E-01	1.29E+00
XR_003032451.1	1.42E+01	1.30E-03	1.63E-03	1.70E-01	-2.56E+00	-5.89E+00	2.35E-01	1.38E+00
XR_003032531.1	6.82E+01	1.10E-03	1.40E-03	5.28E-01	-9.21E-01	-1.89E+00	2.53E+00	4.79E+00
XR_003032593.1	1.39E+01	2.53E-04	3.80E-04	3.87E-01	-1.37E+00	-2.59E+00	4.19E-01	1.08E+00
XR_003032648.1	3.03E+01	5.66E-03	6.29E-03	6.74E-01	-5.68E-01	-1.48E+00	1.31E+00	1.94E+00
XR_003032672.1	6.16E+02	1.55E-03	1.92E-03	7.70E-01	-3.77E-01	-1.30E+00	2.85E+01	3.71E+01
XR_003032689.1	1.80E+02	1.31E-06	4.49E-06	5.76E-01	-7.96E-01	-1.74E+00	7.08E+00	1.23E+01
XR_003032753.1	7.19E+01	3.50E-04	5.04E-04	4.84E-01	-1.05E+00	-2.07E+00	2.54E+00	5.26E+00
XR_003032844.1	8.85E+00	9.07E-05	1.57E-04	4.86E-02	-4.36E+00	-2.06E+01	4.80E-02	9.88E-01
XR_003032957.1	4.00E+00	1.24E-02	1.32E-02	1.71E-01	-2.54E+00	-5.83E+00	6.85E-02	4.00E-01
XR_003032984.1	2.22E+01	5.34E-03	5.97E-03	1.00E-01	-3.32E+00	-9.97E+00	2.39E-01	2.38E+00
XR_003033023.1	1.34E+01	3.18E-04	4.63E-04	3.39E-01	-1.56E+00	-2.95E+00	3.66E-01	1.08E+00

XR_003033053.1	9.71E+01	5.72E-04	7.80E-04	6.29E-01	-6.68E-01	-1.59E+00	3.98E+00	6.33E+00
XR_003033069.1	8.54E+00	8.33E-04	1.08E-03	1.71E-01	-2.55E+00	-5.86E+00	1.40E-01	8.19E-01
XR_003033139.1	8.18E+01	4.04E-03	4.63E-03	8.21E-01	-2.85E-01	-1.22E+00	3.92E+00	4.77E+00
XR_003033185.1	9.71E+02	3.88E-05	7.53E-05	6.21E-01	-6.88E-01	-1.61E+00	4.02E+01	6.48E+01
XR_003033257.1	1.85E+02	9.27E-04	1.20E-03	7.08E-01	-4.98E-01	-1.41E+00	8.24E+00	1.16E+01
XR_003033315.1	4.77E+00	3.55E-03	4.09E-03	1.75E-01	-2.51E+00	-5.70E+00	8.46E-02	4.82E-01
XR_003033363.1	8.49E+00	5.40E-02	5.48E-02	7.34E-01	-4.45E-01	-1.36E+00	3.86E-01	5.26E-01
XR_003033364.1	8.49E+00	5.40E-02	5.48E-02	7.34E-01	-4.45E-01	-1.36E+00	3.86E-01	5.26E-01
XR_003033405.1	5.10E+02	1.13E-07	7.45E-07	3.83E-01	-1.39E+00	-2.61E+00	1.54E+01	4.02E+01
XR_003033429.1	2.35E+01	8.64E-07	3.28E-06	1.83E-01	-2.45E+00	-5.48E+00	4.11E-01	2.25E+00
XR_003033454.1	1.88E+00	2.31E-10	1.08E-08	4.45E-04	-1.11E+01	-2.25E+03	1.00E-04	2.25E-01
XR_003033463.1	1.58E+01	1.43E-04	2.32E-04	5.01E-01	-9.97E-01	-2.00E+00	5.63E-01	1.12E+00
XR_003033464.1	2.76E+00	2.26E-03	2.71E-03	1.41E-01	-2.82E+00	-7.08E+00	3.96E-02	2.80E-01
XR_003033525.1	4.65E+00	1.12E-01	1.13E-01	5.12E-01	-9.67E-01	-1.95E+00	1.71E-01	3.35E-01
XR_003033542.1	1.42E+01	1.75E-04	2.79E-04	4.34E-02	-4.53E+00	-2.31E+01	7.00E-02	1.61E+00
XR_003033843.1	1.85E+03	5.21E-08	4.03E-07	5.19E-01	-9.46E-01	-1.93E+00	6.82E+01	1.31E+02
XR_003034286.1	6.43E+00	3.18E-03	3.71E-03	4.47E-01	-1.16E+00	-2.24E+00	2.12E-01	4.74E-01
XR_003034564.1	5.61E+02	4.58E-07	2.05E-06	6.12E-01	-7.08E-01	-1.63E+00	2.28E+01	3.73E+01
XR_003034596.1	1.57E+01	3.35E-04	4.86E-04	2.52E-01	-1.99E+00	-3.96E+00	3.50E-01	1.39E+00
XR_003034612.1	5.14E+01	1.46E-03	1.82E-03	5.30E-01	-9.17E-01	-1.89E+00	1.89E+00	3.56E+00
XR_003034749.1	2.04E+01	1.52E-07	9.10E-07	2.55E-01	-1.97E+00	-3.92E+00	4.60E-01	1.81E+00
XR_003034902.1	2.99E+01	1.04E-05	2.44E-05	9.64E-03	-6.70E+00	-1.04E+02	3.41E-02	3.53E+00
XR_003035004.1	2.12E+02	8.94E-03	9.68E-03	8.12E-01	-3.01E-01	-1.23E+00	1.01E+01	1.24E+01
XR_003035342.1	1.98E+02	1.37E-04	2.24E-04	6.70E-01	-5.78E-01	-1.49E+00	8.58E+00	1.28E+01
XR_003035343.1	1.53E+02	1.41E-04	2.29E-04	6.71E-01	-5.75E-01	-1.49E+00	6.58E+00	9.81E+00
XR_003035344.1	1.77E+03	1.19E-07	7.63E-07	6.03E-01	-7.30E-01	-1.66E+00	7.18E+01	1.19E+02
XR_003035393.1	1.02E+02	1.05E-06	3.81E-06	3.53E-01	-1.50E+00	-2.83E+00	2.90E+00	8.22E+00
XR_003035427.1	2.00E+02	7.63E-06	1.89E-05	6.40E-01	-6.44E-01	-1.56E+00	8.37E+00	1.31E+01
XR_003035429.1	4.84E+01	2.38E-04	3.60E-04	6.59E-01	-6.01E-01	-1.52E+00	2.06E+00	3.13E+00
XR_003035460.1	2.69E+01	1.36E-10	7.55E-09	4.01E-02	-4.64E+00	-2.49E+01	1.22E-01	3.05E+00
XR_003035476.1	6.83E+01	5.95E-02	6.03E-02	5.34E-01	-9.04E-01	-1.87E+00	2.61E+00	4.88E+00
XR_003035604.1	2.45E+01	2.29E-02	2.39E-02	6.77E-01	-5.62E-01	-1.48E+00	1.05E+00	1.55E+00
XR_003035797.1	8.49E+00	5.40E-02	5.48E-02	7.34E-01	-4.45E-01	-1.36E+00	3.86E-01	5.26E-01

XR_003036083.1	1.49E+01	9.76E-03	1.05E-02	1.83E-01	-2.45E+00	-5.47E+00	2.63E-01	1.44E+00
XR_003036085.1	2.69E+01	1.90E-03	2.30E-03	3.88E-01	-1.37E+00	-2.58E+00	8.10E-01	2.09E+00
XR_003036141.1	6.83E+01	2.94E-06	8.57E-06	3.42E-01	-1.55E+00	-2.92E+00	1.93E+00	5.62E+00
XR_003036235.1	5.23E+00	3.05E-03	3.57E-03	2.22E-01	-2.17E+00	-4.50E+00	1.02E-01	4.59E-01
XR_003036500.1	4.14E+01	1.60E-04	2.58E-04	5.93E-01	-7.54E-01	-1.69E+00	1.63E+00	2.75E+00
XR_003036857.1	5.63E+01	3.52E-07	1.67E-06	4.10E-01	-1.29E+00	-2.44E+00	1.79E+00	4.37E+00
XR_003037052.1	5.30E+02	6.76E-03	7.43E-03	7.54E-01	-4.07E-01	-1.33E+00	2.42E+01	3.21E+01
XR_003037101.1	1.00E+01	4.01E-04	5.70E-04	3.89E-01	-1.36E+00	-2.57E+00	3.08E-01	7.91E-01
XR_003037273.1	1.45E+01	2.37E-02	2.46E-02	6.75E-01	-5.67E-01	-1.48E+00	6.19E-01	9.18E-01
XR_003037281.1	2.68E+01	5.26E-05	9.82E-05	5.32E-01	-9.09E-01	-1.88E+00	1.01E+00	1.89E+00
XR_003037285.1	1.45E+01	1.71E-02	1.80E-02	6.45E-01	-6.32E-01	-1.55E+00	6.15E-01	9.53E-01
XR_003037308.1	5.55E+00	7.99E-04	1.05E-03	3.65E-01	-1.45E+00	-2.74E+00	1.60E-01	4.39E-01
XR_003037317.1	3.07E+02	1.68E-02	1.77E-02	8.42E-01	-2.48E-01	-1.19E+00	1.49E+01	1.77E+01
XR_003037474.1	3.54E+02	3.20E-07	1.55E-06	2.67E-01	-1.91E+00	-3.75E+00	8.40E+00	3.15E+01
XR_003037594.1	4.11E+01	5.08E-04	7.01E-04	4.17E-01	-1.26E+00	-2.40E+00	1.32E+00	3.17E+00
XR_003037691.1	4.38E+02	4.79E-05	9.11E-05	7.12E-01	-4.90E-01	-1.40E+00	1.95E+01	2.74E+01
XR_003037718.1	1.38E+01	7.54E-09	9.50E-08	3.65E-03	-8.10E+00	-2.74E+02	5.91E-03	1.62E+00
XR_003037729.1	9.73E+01	1.73E-06	5.55E-06	4.33E-02	-4.53E+00	-2.31E+01	4.69E-01	1.08E+01
XR_003037801.1	1.09E+01	1.50E-04	2.42E-04	3.62E-02	-4.79E+00	-2.76E+01	4.56E-02	1.26E+00
XR_003037806.1	7.47E+01	4.72E-03	5.33E-03	7.46E-01	-4.23E-01	-1.34E+00	3.39E+00	4.54E+00
XR_003037808.1	3.00E+01	3.76E-05	7.31E-05	2.26E-02	-5.47E+00	-4.43E+01	8.01E-02	3.55E+00
XR_003037816.1	5.49E+00	5.94E-04	8.05E-04	1.03E-01	-3.28E+00	-9.69E+00	5.95E-02	5.77E-01
XR_003037828.1	1.81E+00	2.77E-04	4.11E-04	2.50E-01	-2.00E+00	-4.00E+00	3.97E-02	1.59E-01
XR_003037846.1	1.36E+01	5.31E-02	5.40E-02	4.06E-01	-1.30E+00	-2.46E+00	4.45E-01	1.10E+00
XR_003037929.1	1.93E+00	3.12E-01	3.13E-01	3.34E-01	-1.58E+00	-2.99E+00	5.45E-02	1.63E-01
XR_003038009.1	3.60E+00	1.18E-01	1.19E-01	6.75E-01	-5.67E-01	-1.48E+00	1.55E-01	2.29E-01
XR_003038056.1	2.52E+00	1.53E-06	5.07E-06	2.58E-02	-5.28E+00	-3.87E+01	7.32E-03	2.84E-01
XR_003038178.1	8.02E+00	1.48E-04	2.39E-04	6.37E-02	-3.97E+00	-1.57E+01	5.65E-02	8.87E-01
XR_003038179.1	3.98E+01	1.20E-09	3.02E-08	1.22E-01	-3.03E+00	-8.18E+00	4.91E-01	4.02E+00
XR_003038180.1	3.02E+01	8.14E-08	5.78E-07	1.33E-01	-2.91E+00	-7.53E+00	4.10E-01	3.09E+00
XR_083674.4	1.27E+03	2.63E-06	7.81E-06	6.65E-01	-5.88E-01	-1.50E+00	5.48E+01	8.23E+01
XR_084136.4	3.94E+03	1.01E-05	2.39E-05	7.56E-01	-4.03E-01	-1.32E+00	1.81E+02	2.39E+02
XR_139212.4	8.38E+01	6.02E-03	6.64E-03	7.35E-01	-4.45E-01	-1.36E+00	3.80E+00	5.17E+00

XR_139625.4	5.24E+00	5.08E-04	7.01E-04	3.47E-01	-1.53E+00	-2.88E+00	1.49E-01	4.29E-01
XR_233379.4	6.55E+00	7.61E-03	8.29E-03	4.91E-01	-1.03E+00	-2.04E+00	2.34E-01	4.76E-01
XR_234350.2	6.88E+01	3.86E-02	3.96E-02	7.93E-01	-3.34E-01	-1.26E+00	3.25E+00	4.09E+00
XR_234970.4	3.47E+01	7.82E-04	1.03E-03	5.36E-01	-9.01E-01	-1.87E+00	1.30E+00	2.42E+00
XR_234993.3	1.15E+02	2.62E-03	3.11E-03	5.00E-01	-9.99E-01	-2.00E+00	4.02E+00	8.03E+00
XR_235101.4	5.01E+00	6.98E-04	9.32E-04	3.36E-01	-1.57E+00	-2.97E+00	1.42E-01	4.21E-01
XR_235867.4	1.68E+02	6.05E-04	8.19E-04	6.99E-01	-5.17E-01	-1.43E+00	7.43E+00	1.06E+01
XR_235954.4	2.40E+00	1.75E-04	2.79E-04	5.53E-02	-4.18E+00	-1.81E+01	1.48E-02	2.68E-01
XR_236319.2	1.51E+02	2.34E-02	2.43E-02	8.02E-01	-3.18E-01	-1.25E+00	7.15E+00	8.91E+00
XR_236839.4	4.30E+00	5.66E-04	7.73E-04	3.57E-01	-1.49E+00	-2.80E+00	1.24E-01	3.48E-01
XR_237014.4	1.46E+01	8.06E-04	1.05E-03	5.46E-01	-8.73E-01	-1.83E+00	5.60E-01	1.03E+00
XR_237209.4	7.74E+01	3.74E-03	4.30E-03	5.31E-01	-9.12E-01	-1.88E+00	2.86E+00	5.38E+00
XR_237845.3	3.60E+02	4.61E-03	5.22E-03	7.42E-01	-4.31E-01	-1.35E+00	1.64E+01	2.22E+01
XR_238808.3	3.06E+02	1.26E-03	1.59E-03	6.62E-01	-5.94E-01	-1.51E+00	1.30E+01	1.97E+01
XR_238819.3	1.38E+02	1.82E-03	2.22E-03	7.40E-01	-4.34E-01	-1.35E+00	6.27E+00	8.47E+00
XR_238838.4	2.95E+01	1.69E-06	5.46E-06	2.62E-01	-1.93E+00	-3.81E+00	6.78E-01	2.59E+00
XR_239260.4	8.85E+00	3.47E-04	5.02E-04	4.35E-01	-1.20E+00	-2.30E+00	2.93E-01	6.73E-01
XR_239333.4	2.51E+01	3.24E-03	3.77E-03	6.62E-01	-5.95E-01	-1.51E+00	1.07E+00	1.62E+00
XR_239539.4	2.43E+02	1.69E-05	3.67E-05	5.97E-01	-7.44E-01	-1.67E+00	9.74E+00	1.63E+01
XR_239770.4	1.56E+01	1.83E-02	1.91E-02	5.11E-01	-9.68E-01	-1.96E+00	5.67E-01	1.11E+00
XR_239936.3	2.31E+02	1.13E-03	1.44E-03	7.75E-01	-3.68E-01	-1.29E+00	1.07E+01	1.38E+01
XR_239937.4	8.47E+02	9.15E-04	1.18E-03	8.40E-01	-2.51E-01	-1.19E+00	4.10E+01	4.88E+01
XR_240083.4	1.39E+02	3.34E-04	4.83E-04	6.72E-01	-5.74E-01	-1.49E+00	6.01E+00	8.95E+00
XR_240196.4	1.07E+02	1.98E-12	5.81E-10	3.51E-02	-4.83E+00	-2.85E+01	4.23E-01	1.20E+01
XR_240634.4	3.63E+01	1.59E-06	5.21E-06	3.75E-01	-1.41E+00	-2.67E+00	1.09E+00	2.90E+00
XR_240655.4	8.27E+00	2.10E-03	2.54E-03	5.16E-01	-9.55E-01	-1.94E+00	3.06E-01	5.93E-01
XR_240730.3	2.61E+01	7.96E-05	1.41E-04	3.16E-01	-1.66E+00	-3.16E+00	6.91E-01	2.18E+00
XR_805221.3	4.68E+01	1.04E-07	6.94E-07	4.49E-03	-7.80E+00	-2.23E+02	2.50E-02	5.58E+00
XR_805447.3	8.55E+02	4.15E-09	6.71E-08	4.08E-01	-1.29E+00	-2.45E+00	2.74E+01	6.72E+01
XR_806416.2	8.53E+01	8.23E-04	1.07E-03	5.86E-01	-7.72E-01	-1.71E+00	3.39E+00	5.79E+00
XR_806651.3	7.91E+01	3.10E-09	5.63E-08	2.37E-01	-2.08E+00	-4.21E+00	1.74E+00	7.32E+00
XR_806820.3	1.71E+00	1.02E-02	1.10E-02	1.19E-01	-3.07E+00	-8.42E+00	2.19E-02	1.84E-01
XR_806995.3	2.10E+01	1.12E-01	1.12E-01	5.24E-01	-9.33E-01	-1.91E+00	7.78E-01	1.48E+00

XR_807571.3	4.82E+02	4.19E-03	4.77E-03	7.69E-01	-3.79E-01	-1.30E+00	2.24E+01	2.91E+01
XR_807592.3	4.23E+02	9.48E-07	3.51E-06	3.02E-01	-1.73E+00	-3.31E+00	1.08E+01	3.58E+01
XR_807685.3	1.06E+02	2.35E-03	2.81E-03	7.18E-01	-4.77E-01	-1.39E+00	4.72E+00	6.57E+00
XR_807866.3	3.35E+02	2.11E-07	1.15E-06	7.51E-02	-3.74E+00	-1.33E+01	2.75E+00	3.67E+01
XR_809071.1	1.64E+03	2.54E-07	1.31E-06	5.96E-01	-7.46E-01	-1.68E+00	6.67E+01	1.12E+02
XR_809153.2	2.19E+03	1.76E-06	5.65E-06	6.01E-01	-7.33E-01	-1.66E+00	8.84E+01	1.47E+02
XR_809928.3	1.70E+00	2.45E-02	2.54E-02	1.38E-01	-2.85E+00	-7.23E+00	2.45E-02	1.77E-01
XR_810193.3	1.54E+01	1.18E-03	1.50E-03	2.73E-01	-1.88E+00	-3.67E+00	3.64E-01	1.34E+00
XR_810407.3	2.62E+01	3.52E-06	1.00E-05	1.87E-01	-2.42E+00	-5.36E+00	4.63E-01	2.48E+00
XR_810408.3	3.00E+01	1.72E-06	5.53E-06	1.89E-01	-2.40E+00	-5.29E+00	5.38E-01	2.85E+00
XR_810409.3	2.69E+01	1.46E-03	1.81E-03	3.29E-01	-1.60E+00	-3.04E+00	7.21E-01	2.19E+00
XR_810468.3	1.02E+01	4.62E-04	6.45E-04	3.42E-01	-1.55E+00	-2.92E+00	2.90E-01	8.49E-01
XR_810885.3	1.16E+02	4.35E-03	4.94E-03	6.15E-01	-7.02E-01	-1.63E+00	4.73E+00	7.69E+00
XR_811112.3	4.77E+01	1.37E-07	8.47E-07	3.01E-01	-1.73E+00	-3.33E+00	1.21E+00	4.02E+00
XR_811114.3	7.66E+00	6.30E-07	2.58E-06	2.01E-01	-2.31E+00	-4.98E+00	1.45E-01	7.22E-01
XR_811148.3	2.26E+02	1.46E-02	1.54E-02	7.73E-01	-3.72E-01	-1.29E+00	1.05E+01	1.36E+01
XR_811391.3	2.23E+01	3.45E-05	6.78E-05	3.90E-01	-1.36E+00	-2.57E+00	6.77E-01	1.74E+00
XR_811484.3	1.82E+01	3.59E-02	3.69E-02	7.00E-01	-5.15E-01	-1.43E+00	8.08E-01	1.15E+00
XR_811713.3	3.07E+01	2.66E-02	2.75E-02	7.26E-01	-4.62E-01	-1.38E+00	1.38E+00	1.90E+00
XR_811837.3	2.50E+00	1.09E-02	1.17E-02	1.02E-01	-3.29E+00	-9.79E+00	2.68E-02	2.62E-01
XR_812569.3	1.43E+00	1.47E-02	1.56E-02	1.35E-01	-2.89E+00	-7.41E+00	1.99E-02	1.47E-01
XR_812916.3	1.18E+02	3.61E-03	4.16E-03	6.39E-01	-6.45E-01	-1.56E+00	4.90E+00	7.67E+00
XR_813062.2	3.72E+01	2.74E-03	3.24E-03	6.81E-01	-5.53E-01	-1.47E+00	1.59E+00	2.33E+00
XR_813149.3	1.62E+01	7.44E-04	9.83E-04	1.35E-01	-2.88E+00	-7.38E+00	2.28E-01	1.68E+00
XR_813476.3	1.42E+01	3.46E-03	3.99E-03	5.56E-01	-8.46E-01	-1.80E+00	5.44E-01	9.78E-01
XR_813537.3	4.33E+01	1.98E-05	4.18E-05	1.44E-01	-2.80E+00	-6.95E+00	6.43E-01	4.47E+00
XR_814326.1	5.52E+00	7.28E-03	7.95E-03	5.31E-01	-9.13E-01	-1.88E+00	2.09E-01	3.93E-01
XR_814368.3	2.56E+02	5.38E-05	1.00E-04	6.75E-01	-5.66E-01	-1.48E+00	1.11E+01	1.64E+01
XR_814548.3	2.40E+02	4.21E-03	4.79E-03	6.96E-01	-5.23E-01	-1.44E+00	1.04E+01	1.50E+01
XR_814962.3	4.53E+02	5.15E-04	7.10E-04	7.32E-01	-4.50E-01	-1.37E+00	2.06E+01	2.81E+01
XR_814975.3	9.87E+00	3.83E-01	3.83E-01	4.70E-01	-1.09E+00	-2.13E+00	3.40E-01	7.24E-01
XR_815135.3	6.24E+00	2.71E-01	2.72E-01	5.09E-01	-9.76E-01	-1.97E+00	2.36E-01	4.64E-01
XR_815259.2	1.31E+01	9.20E-05	1.59E-04	7.20E-02	-3.80E+00	-1.39E+01	1.01E-01	1.40E+00

XR_815718.3	2.91E+01	5.99E-03	6.62E-03	4.65E-01	-1.10E+00	-2.15E+00	1.01E+00	2.17E+00
XR_815719.3	1.94E+01	5.62E-04	7.69E-04	4.61E-01	-1.12E+00	-2.17E+00	6.66E-01	1.44E+00
XR_816495.3	7.56E+00	4.12E-03	4.71E-03	4.58E-01	-1.13E+00	-2.18E+00	2.64E-01	5.77E-01
XR_816506.3	1.21E+02	5.53E-03	6.16E-03	7.58E-01	-4.01E-01	-1.32E+00	5.58E+00	7.36E+00
XR_816816.3	4.18E+01	1.49E-02	1.57E-02	6.48E-01	-6.26E-01	-1.54E+00	1.78E+00	2.75E+00
YIF1B	4.90E+02	2.22E-07	1.20E-06	5.88E-01	-7.65E-01	-1.70E+00	1.95E+01	3.32E+01
YIPF3	2.84E+03	2.68E-05	5.43E-05	8.11E-01	-3.02E-01	-1.23E+00	1.35E+02	1.67E+02
YJEFN3	1.30E+01	1.97E-02	2.06E-02	6.56E-01	-6.09E-01	-1.52E+00	5.43E-01	8.28E-01
YPEL1	1.02E+03	7.57E-06	1.89E-05	6.51E-01	-6.20E-01	-1.54E+00	4.30E+01	6.61E+01
YPEL4	1.38E+02	3.86E-03	4.44E-03	6.86E-01	-5.43E-01	-1.46E+00	6.08E+00	8.85E+00
ZBTB12	8.53E+02	2.99E-04	4.38E-04	7.03E-01	-5.09E-01	-1.42E+00	3.77E+01	5.36E+01
ZBTB8OS	5.08E+02	2.50E-05	5.12E-05	7.20E-01	-4.74E-01	-1.39E+00	2.24E+01	3.11E+01
ZC3H7B	3.67E+03	1.92E-04	3.00E-04	7.67E-01	-3.83E-01	-1.30E+00	1.70E+02	2.21E+02
ZCCHC3	2.40E+03	4.56E-06	1.24E-05	7.29E-01	-4.57E-01	-1.37E+00	1.08E+02	1.48E+02
ZDHHC24	3.66E+02	6.84E-03	7.51E-03	8.34E-01	-2.61E-01	-1.20E+00	1.75E+01	2.10E+01
ZDHHC5	1.95E+03	2.65E-10	1.21E-08	6.02E-01	-7.33E-01	-1.66E+00	7.87E+01	1.31E+02
ZDHHC7	1.48E+03	6.00E-07	2.51E-06	7.05E-01	-5.03E-01	-1.42E+00	6.58E+01	9.32E+01
ZFP42	1.11E+01	4.67E-09	7.04E-08	4.98E-03	-7.65E+00	-2.01E+02	6.68E-03	1.34E+00
ZFPM1	9.37E+02	4.22E-06	1.16E-05	6.10E-01	-7.14E-01	-1.64E+00	3.82E+01	6.27E+01
ZIM3	6.64E+01	4.51E-06	1.23E-05	1.39E-02	-6.17E+00	-7.21E+01	1.09E-01	7.87E+00
ZNF114	1.21E+01	9.11E-05	1.57E-04	2.99E-02	-5.06E+00	-3.34E+01	4.13E-02	1.38E+00
ZNF219	7.33E+02	1.43E-03	1.78E-03	7.51E-01	-4.13E-01	-1.33E+00	3.31E+01	4.41E+01
ZNF282	1.59E+03	4.08E-04	5.79E-04	8.43E-01	-2.46E-01	-1.19E+00	7.70E+01	9.13E+01
ZNF296	8.84E+01	3.21E-12	7.32E-10	6.17E-02	-4.02E+00	-1.62E+01	5.98E-01	9.70E+00
ZNF362	2.65E+03	2.30E-06	6.98E-06	6.42E-01	-6.39E-01	-1.56E+00	1.11E+02	1.73E+02
ZNF382	2.13E+02	1.02E-05	2.41E-05	7.40E-01	-4.34E-01	-1.35E+00	9.69E+00	1.31E+01
ZNF423	1.13E+03	2.89E-04	4.26E-04	6.56E-01	-6.08E-01	-1.52E+00	4.80E+01	7.32E+01
ZNF428	1.40E+03	2.80E-07	1.40E-06	6.86E-01	-5.44E-01	-1.46E+00	6.15E+01	8.96E+01
ZNF444	1.29E+03	3.33E-08	2.93E-07	6.06E-01	-7.22E-01	-1.65E+00	5.26E+01	8.67E+01
ZNF446	4.42E+02	2.34E-07	1.23E-06	4.69E-01	-1.09E+00	-2.13E+00	1.53E+01	3.26E+01
ZNF488	2.34E+01	1.06E-04	1.79E-04	4.66E-01	-1.10E+00	-2.14E+00	8.06E-01	1.73E+00
ZNF579	9.72E+02	6.49E-06	1.65E-05	6.40E-01	-6.45E-01	-1.56E+00	4.09E+01	6.40E+01
ZNF582	8.80E+01	5.25E-03	5.87E-03	7.62E-01	-3.93E-01	-1.31E+00	4.03E+00	5.30E+00

ZNF703	8.28E+02	1.15E-09	3.00E-08	3.83E-01	-1.39E+00	-2.61E+00	2.53E+01	6.61E+01
ZNF710	9.76E+02	1.98E-03	2.40E-03	7.92E-01	-3.37E-01	-1.26E+00	4.59E+01	5.79E+01
ZNF740	1.79E+03	9.59E-05	1.64E-04	7.36E-01	-4.41E-01	-1.36E+00	8.07E+01	1.10E+02
ZNF768	1.87E+03	4.32E-05	8.31E-05	7.78E-01	-3.62E-01	-1.29E+00	8.71E+01	1.12E+02
ZNF789	6.74E+01	1.76E-04	2.79E-04	6.34E-01	-6.57E-01	-1.58E+00	2.78E+00	4.39E+00
ZNF865	6.21E+02	8.22E-05	1.45E-04	7.40E-01	-4.35E-01	-1.35E+00	2.82E+01	3.81E+01
ZNHIT1	4.70E+02	1.16E-04	1.93E-04	6.76E-01	-5.64E-01	-1.48E+00	2.04E+01	3.01E+01
ZSCAN10	1.16E+02	2.28E-10	1.08E-08	1.00E-02	-6.64E+00	-9.95E+01	1.42E-01	1.41E+01
ZSWIM1	1.85E+02	1.29E-06	4.45E-06	5.78E-01	-7.90E-01	-1.73E+00	7.30E+00	1.26E+01
C9orf3	1.21E+03	3.19E-04	4.65E-04	6.98E-01	-5.19E-01	-1.43E+00	5.30E+01	7.60E+01

S3 Table. Gene Specific Analysis (late versus early gestation) for genes in cluster 2. Shown are the fold change and its significance (p-value), log 2 ratio and False Discovery Rate (FDR) across gestation; least square (LS) mean (late and early gestation); total counts per million.

Gene symbol	Total counts	P-value	FDR step up	Ratio	Log2(Ratio)	Fold change	LSMean(Late)	LSMean(Early)
FLT1	7.30E+02	2.30E-16	4.10E-13	3.99E+00	2.00E+00	3.99E+00	5.94E+01	1.49E+01
EBF1	1.64E+02	1.07E-14	9.57E-12	8.53E+00	3.09E+00	8.53E+00	1.48E+01	1.73E+00
CNNM2	5.57E+02	6.44E-14	3.82E-11	3.30E+00	1.72E+00	3.30E+00	4.34E+01	1.32E+01
JAZF1	2.23E+02	9.10E-14	4.05E-11	6.68E+00	2.74E+00	6.68E+00	1.95E+01	2.92E+00
JAG1	1.48E+03	1.53E-13	4.57E-11	5.18E+00	2.37E+00	5.18E+00	1.25E+02	2.41E+01
DSE	1.18E+03	1.54E-13	4.57E-11	3.54E+00	1.83E+00	3.54E+00	9.38E+01	2.65E+01
EDIL3	3.68E+02	2.83E-13	7.20E-11	1.08E+01	3.44E+00	1.08E+01	3.39E+01	3.13E+00
FBXO32-2	2.09E+02	6.18E-13	1.38E-10	2.41E+01	4.59E+00	2.41E+01	2.01E+01	8.34E-01
CRIM1	9.40E+02	8.83E-13	1.60E-10	4.09E+00	2.03E+00	4.09E+00	7.66E+01	1.88E+01
SLC40A1	1.08E+03	9.00E-13	1.60E-10	2.53E+00	1.34E+00	2.53E+00	7.89E+01	3.12E+01
TMTC2	6.60E+02	1.35E-12	2.07E-10	4.78E+00	2.26E+00	4.78E+00	5.54E+01	1.16E+01
MRVI1	1.41E+03	1.40E-12	2.07E-10	1.24E+01	3.63E+00	1.24E+01	1.31E+02	1.06E+01
ARHGEF6	5.91E+02	2.22E-12	3.05E-10	3.44E+00	1.78E+00	3.44E+00	4.65E+01	1.35E+01
COL5A2	5.58E+03	2.79E-12	3.55E-10	4.78E+00	2.26E+00	4.78E+00	4.68E+02	9.80E+01
SORBS1	1.16E+03	3.48E-12	3.69E-10	5.08E+00	2.35E+00	5.08E+00	9.83E+01	1.93E+01
EPAS1	6.79E+02	3.86E-12	3.69E-10	7.33E+00	2.87E+00	7.33E+00	6.01E+01	8.20E+00
MDFIC	4.39E+02	3.86E-12	3.69E-10	3.94E+00	1.98E+00	3.94E+00	3.56E+01	9.03E+00
KLF6	2.32E+03	4.06E-12	3.69E-10	3.38E+00	1.76E+00	3.38E+00	1.82E+02	5.39E+01
ITPR1	1.73E+03	4.10E-12	3.69E-10	4.52E+00	2.18E+00	4.52E+00	1.44E+02	3.18E+01
ELF1	3.63E+02	4.61E-12	3.69E-10	2.94E+00	1.55E+00	2.94E+00	2.75E+01	9.36E+00
ECM2	2.18E+03	4.69E-12	3.69E-10	4.81E+00	2.27E+00	4.81E+00	1.83E+02	3.79E+01
FOXP1	1.41E+03	4.70E-12	3.69E-10	2.28E+00	1.19E+00	2.28E+00	1.01E+02	4.43E+01
PTGS2	5.32E+01	4.77E-12	3.69E-10	7.13E+00	2.83E+00	7.13E+00	4.70E+00	6.59E-01
RAB30	3.96E+02	5.77E-12	4.28E-10	5.32E+00	2.41E+00	5.32E+00	3.38E+01	6.35E+00
CRYBG1	4.00E+02	6.66E-12	4.74E-10	3.38E+00	1.76E+00	3.38E+00	3.13E+01	9.25E+00
TGFB2	3.83E+02	7.68E-12	4.74E-10	4.91E+00	2.30E+00	4.91E+00	3.23E+01	6.58E+00
DKK3	9.80E+02	7.68E-12	4.74E-10	3.64E+00	1.87E+00	3.64E+00	7.78E+01	2.13E+01
TGFBR2	2.27E+03	7.73E-12	4.74E-10	2.64E+00	1.40E+00	2.64E+00	1.67E+02	6.34E+01
MYO1D	1.07E+03	7.89E-12	4.74E-10	3.40E+00	1.76E+00	3.40E+00	8.44E+01	2.48E+01

IQGAP1	2.52E+03	8.14E-12	4.74E-10	3.15E+00	1.65E+00	3.15E+00	1.96E+02	6.22E+01
GGTA1	3.29E+02	8.39E-12	4.74E-10	4.84E+00	2.28E+00	4.84E+00	2.75E+01	5.69E+00
SYTL2	3.94E+02	8.52E-12	4.74E-10	6.23E+00	2.64E+00	6.23E+00	3.43E+01	5.51E+00
MTURN	1.56E+03	1.05E-11	5.53E-10	2.88E+00	1.53E+00	2.88E+00	1.18E+02	4.11E+01
JPH1	4.74E+02	1.06E-11	5.53E-10	7.40E+00	2.89E+00	7.40E+00	4.19E+01	5.67E+00
DDAH1	1.04E+03	1.10E-11	5.59E-10	6.20E+00	2.63E+00	6.20E+00	9.00E+01	1.45E+01
ADAMTSL1	2.80E+02	1.17E-11	5.77E-10	6.03E+00	2.59E+00	6.03E+00	2.41E+01	4.00E+00
RAB33B	2.67E+02	1.21E-11	5.84E-10	3.01E+00	1.59E+00	3.01E+00	2.04E+01	6.80E+00
CARD6	3.17E+02	1.28E-11	6.00E-10	2.53E+00	1.34E+00	2.53E+00	2.32E+01	9.18E+00
ANTXR1	7.63E+02	1.47E-11	6.73E-10	8.16E+00	3.03E+00	8.16E+00	6.86E+01	8.41E+00
RNF19A	1.45E+03	1.54E-11	6.76E-10	2.77E+00	1.47E+00	2.77E+00	1.09E+02	3.94E+01
PARD3B	7.11E+02	1.57E-11	6.76E-10	3.49E+00	1.80E+00	3.49E+00	5.62E+01	1.61E+01
SYNM	1.20E+03	1.59E-11	6.76E-10	5.43E+00	2.44E+00	5.43E+00	1.02E+02	1.88E+01
PLCL1	1.21E+02	1.69E-11	6.77E-10	7.00E+00	2.81E+00	7.00E+00	1.07E+01	1.52E+00
IL6ST	1.49E+03	1.72E-11	6.77E-10	3.21E+00	1.68E+00	3.21E+00	1.16E+02	3.60E+01
FRY	3.82E+02	1.72E-11	6.77E-10	3.20E+00	1.68E+00	3.20E+00	2.96E+01	9.27E+00
MYLK	8.43E+03	1.75E-11	6.77E-10	7.80E+00	2.96E+00	7.80E+00	7.52E+02	9.65E+01
GUCY1B1	8.43E+02	1.81E-11	6.85E-10	2.97E+00	1.57E+00	2.97E+00	6.42E+01	2.16E+01
MFHAS1	1.24E+03	1.95E-11	7.21E-10	3.03E+00	1.60E+00	3.03E+00	9.53E+01	3.14E+01
NFIA	1.95E+03	1.98E-11	7.21E-10	2.76E+00	1.46E+00	2.76E+00	1.47E+02	5.32E+01
CAMK2D	7.89E+02	2.05E-11	7.32E-10	2.48E+00	1.31E+00	2.48E+00	5.76E+01	2.33E+01
SNRK	1.05E+03	2.16E-11	7.54E-10	2.54E+00	1.35E+00	2.54E+00	7.75E+01	3.05E+01
FBN1	2.45E+03	2.24E-11	7.68E-10	3.81E+00	1.93E+00	3.81E+00	1.98E+02	5.19E+01
GUCY1A1	9.40E+02	2.33E-11	7.83E-10	3.68E+00	1.88E+00	3.68E+00	7.52E+01	2.04E+01
ETV6	4.49E+02	2.47E-11	8.16E-10	6.48E+00	2.70E+00	6.48E+00	3.92E+01	6.05E+00
TIMP3	2.02E+03	2.52E-11	8.16E-10	3.82E+00	1.93E+00	3.82E+00	1.63E+02	4.27E+01
CYBRD1	9.26E+02	2.58E-11	8.20E-10	3.48E+00	1.80E+00	3.48E+00	7.31E+01	2.10E+01
PDP2	3.54E+02	2.98E-11	9.30E-10	2.56E+00	1.36E+00	2.56E+00	2.61E+01	1.02E+01
GDAP1	1.20E+02	3.07E-11	9.44E-10	4.39E+00	2.13E+00	4.39E+00	9.88E+00	2.25E+00
TENM2	1.70E+03	3.18E-11	9.61E-10	8.94E+00	3.16E+00	8.94E+00	1.53E+02	1.71E+01
SMAD3	8.38E+02	3.25E-11	9.64E-10	5.35E+00	2.42E+00	5.35E+00	7.12E+01	1.33E+01
PDGFD	2.02E+02	3.34E-11	9.75E-10	2.11E+01	4.40E+00	2.11E+01	1.93E+01	9.17E-01
FUT11	7.25E+02	3.96E-11	1.13E-09	2.75E+00	1.46E+00	2.75E+00	5.44E+01	1.98E+01

MAN1A1	8.18E+02	4.21E-11	1.13E-09	3.10E+00	1.63E+00	3.10E+00	6.31E+01	2.04E+01
ROR1	4.77E+02	4.24E-11	1.13E-09	5.03E+00	2.33E+00	5.03E+00	4.03E+01	8.01E+00
COL24A1	1.50E+02	4.24E-11	1.13E-09	1.54E+01	3.95E+00	1.54E+01	1.42E+01	9.17E-01
MYH11	1.36E+04	4.25E-11	1.13E-09	1.07E+01	3.42E+00	1.07E+01	1.25E+03	1.16E+02
TWSG1	1.38E+03	4.26E-11	1.13E-09	3.07E+00	1.62E+00	3.07E+00	1.06E+02	3.46E+01
SGMS1	6.67E+02	4.42E-11	1.16E-09	3.27E+00	1.71E+00	3.27E+00	5.20E+01	1.59E+01
CALCRL	4.72E+02	4.84E-11	1.25E-09	5.77E+00	2.53E+00	5.77E+00	4.07E+01	7.05E+00
ANO6	7.64E+02	5.04E-11	1.27E-09	3.70E+00	1.89E+00	3.70E+00	6.13E+01	1.66E+01
ARCN1	1.92E+03	5.06E-11	1.27E-09	2.28E+00	1.19E+00	2.28E+00	1.36E+02	5.96E+01
SPON1	1.53E+04	5.17E-11	1.28E-09	6.49E+00	2.70E+00	6.49E+00	1.34E+03	2.06E+02
CRYBG3	4.88E+02	5.38E-11	1.31E-09	2.33E+00	1.22E+00	2.33E+00	3.49E+01	1.50E+01
CAVIN2	1.04E+03	5.68E-11	1.37E-09	4.01E+00	2.01E+00	4.01E+00	8.44E+01	2.10E+01
PRKAR2A	1.80E+03	5.80E-11	1.38E-09	1.89E+00	9.18E-01	1.89E+00	1.22E+02	6.44E+01
AFAP1L2	2.00E+03	6.19E-11	1.45E-09	5.04E+00	2.33E+00	5.04E+00	1.68E+02	3.34E+01
MYOCD	1.31E+03	6.39E-11	1.48E-09	5.72E+00	2.52E+00	5.72E+00	1.13E+02	1.97E+01
SMAD9	6.98E+02	7.15E-11	1.63E-09	3.35E+00	1.75E+00	3.35E+00	5.46E+01	1.63E+01
SYNPO2	2.29E+03	7.21E-11	1.63E-09	8.69E+00	3.12E+00	8.69E+00	2.06E+02	2.38E+01
LAMA4	1.56E+03	7.55E-11	1.68E-09	5.48E+00	2.45E+00	5.48E+00	1.34E+02	2.44E+01
CALD1	7.36E+03	7.84E-11	1.72E-09	4.39E+00	2.13E+00	4.39E+00	6.10E+02	1.39E+02
FRMD6	5.85E+02	8.18E-11	1.78E-09	5.36E+00	2.42E+00	5.36E+00	4.98E+01	9.29E+00
MTMR11	3.36E+02	8.53E-11	1.82E-09	2.53E+00	1.34E+00	2.53E+00	2.46E+01	9.73E+00
SERINC5	2.30E+03	8.61E-11	1.82E-09	3.86E+00	1.95E+00	3.86E+00	1.85E+02	4.80E+01
FNBP1	2.14E+03	8.75E-11	1.82E-09	6.75E+00	2.75E+00	6.75E+00	1.87E+02	2.78E+01
DGKH	4.99E+02	8.79E-11	1.82E-09	3.05E+00	1.61E+00	3.05E+00	3.83E+01	1.26E+01
TMEM170B	4.22E+02	9.29E-11	1.90E-09	2.12E+00	1.09E+00	2.12E+00	2.94E+01	1.39E+01
ACTA2	1.10E+04	9.43E-11	1.90E-09	8.34E+00	3.06E+00	8.34E+00	9.87E+02	1.18E+02
FAM198B	1.25E+02	9.50E-11	1.90E-09	1.74E+01	4.12E+00	1.74E+01	1.19E+01	6.82E-01
USP46	9.33E+02	9.83E-11	1.95E-09	2.05E+00	1.04E+00	2.05E+00	6.45E+01	3.15E+01
EIF2AK3	5.30E+02	1.02E-10	2.00E-09	2.30E+00	1.20E+00	2.30E+00	3.80E+01	1.65E+01
ACSL5	2.74E+02	1.07E-10	2.07E-09	4.07E+00	2.03E+00	4.07E+00	2.22E+01	5.45E+00
SSPN	1.20E+03	1.11E-10	2.11E-09	2.54E+00	1.34E+00	2.54E+00	8.89E+01	3.50E+01
DPYD	6.33E+02	1.11E-10	2.11E-09	4.41E+00	2.14E+00	4.41E+00	5.25E+01	1.19E+01
ITGA4	8.65E+01	1.13E-10	2.12E-09	9.21E+00	3.20E+00	9.21E+00	7.84E+00	8.52E-01

FYCO1	1.93E+03	1.14E-10	2.12E-09	2.95E+00	1.56E+00	2.95E+00	1.47E+02	4.98E+01
FOSL2	3.99E+03	1.17E-10	2.16E-09	4.65E+00	2.22E+00	4.65E+00	3.33E+02	7.16E+01
CHN2	2.20E+02	1.19E-10	2.17E-09	4.00E+00	2.00E+00	4.00E+00	1.79E+01	4.48E+00
MBNL2	8.99E+02	1.23E-10	2.22E-09	3.15E+00	1.66E+00	3.15E+00	6.98E+01	2.21E+01
RIMS1	3.75E+02	1.30E-10	2.31E-09	1.51E+01	3.92E+00	1.51E+01	3.53E+01	2.34E+00
GPM6B	1.05E+03	1.36E-10	2.37E-09	2.61E+00	1.38E+00	2.61E+00	7.82E+01	3.00E+01
FAM172A	6.23E+02	1.37E-10	2.37E-09	2.24E+00	1.16E+00	2.24E+00	4.43E+01	1.98E+01
SUDS3	3.56E+02	1.38E-10	2.37E-09	3.32E+00	1.73E+00	3.32E+00	2.78E+01	8.37E+00
TOR1AIP1	1.22E+02	1.39E-10	2.37E-09	4.54E+00	2.18E+00	4.54E+00	1.01E+01	2.22E+00
PDE1A	3.57E+02	1.39E-10	2.37E-09	5.16E+00	2.37E+00	5.16E+00	3.02E+01	5.85E+00
CXHXorf36	1.08E+03	1.42E-10	2.38E-09	2.90E+00	1.53E+00	2.90E+00	8.22E+01	2.84E+01
GNA14	3.88E+02	1.47E-10	2.44E-09	8.30E+00	3.05E+00	8.30E+00	3.48E+01	4.19E+00
EHD4	3.07E+02	1.48E-10	2.44E-09	3.36E+00	1.75E+00	3.36E+00	2.39E+01	7.10E+00
PCMTD2	8.33E+02	1.49E-10	2.44E-09	1.88E+00	9.11E-01	1.88E+00	5.60E+01	2.98E+01
UNC13C	9.14E+01	1.62E-10	2.62E-09	7.83E+00	2.97E+00	7.83E+00	8.15E+00	1.04E+00
PCMTD1	1.88E+03	1.67E-10	2.69E-09	1.99E+00	9.94E-01	1.99E+00	1.29E+02	6.47E+01
FBXO30	4.82E+02	1.74E-10	2.77E-09	2.26E+00	1.17E+00	2.26E+00	3.42E+01	1.51E+01
IGF1	6.89E+02	1.91E-10	2.98E-09	3.65E+00	1.87E+00	3.65E+00	5.51E+01	1.51E+01
CAST	9.89E+02	1.93E-10	2.98E-09	3.20E+00	1.68E+00	3.20E+00	7.70E+01	2.40E+01
GPR107	2.22E+02	1.94E-10	2.98E-09	4.40E+00	2.14E+00	4.40E+00	1.82E+01	4.13E+00
TBC1D15	3.46E+02	1.94E-10	2.98E-09	2.00E+00	9.97E-01	2.00E+00	2.37E+01	1.19E+01
PRKG1	2.35E+02	1.98E-10	3.00E-09	3.44E+00	1.78E+00	3.44E+00	1.86E+01	5.40E+00
FAM120A	2.24E+03	1.99E-10	3.00E-09	2.10E+00	1.07E+00	2.10E+00	1.56E+02	7.45E+01
COL14A1	9.93E+03	2.02E-10	3.03E-09	5.70E+00	2.51E+00	5.70E+00	8.53E+02	1.50E+02
ADAM33	1.43E+03	2.05E-10	3.05E-09	5.98E+00	2.58E+00	5.98E+00	1.24E+02	2.07E+01
ATP8B4	8.71E+01	2.10E-10	3.08E-09	7.21E+00	2.85E+00	7.21E+00	7.68E+00	1.06E+00
ADAMTS12	7.24E+02	2.12E-10	3.09E-09	6.45E+00	2.69E+00	6.45E+00	6.32E+01	9.81E+00
PRDM8	2.31E+02	2.17E-10	3.11E-09	4.33E+00	2.11E+00	4.33E+00	1.89E+01	4.37E+00
SLC25A37-2	1.85E+03	2.20E-10	3.11E-09	2.12E+00	1.09E+00	2.12E+00	1.30E+02	6.11E+01
MBNL1	1.06E+03	2.21E-10	3.11E-09	2.81E+00	1.49E+00	2.81E+00	8.03E+01	2.86E+01
GBF1	8.12E+01	2.22E-10	3.11E-09	4.16E+00	2.06E+00	4.16E+00	6.57E+00	1.58E+00
TMEM47	1.87E+02	2.23E-10	3.11E-09	7.43E+00	2.89E+00	7.43E+00	1.67E+01	2.24E+00
XR_234157.4	3.02E+02	2.24E-10	3.11E-09	2.68E+00	1.42E+00	2.68E+00	2.25E+01	8.38E+00

DST	5.50E+03	2.33E-10	3.22E-09	3.47E+00	1.80E+00	3.47E+00	4.36E+02	1.26E+02
COLEC12	2.65E+03	2.35E-10	3.22E-09	3.18E+00	1.67E+00	3.18E+00	2.05E+02	6.45E+01
ADAMTSL4	7.35E+02	2.37E-10	3.22E-09	3.91E+00	1.97E+00	3.91E+00	5.94E+01	1.52E+01
SH3D19	2.55E+03	2.52E-10	3.40E-09	2.13E+00	1.09E+00	2.13E+00	1.79E+02	8.40E+01
COL4A3	5.61E+02	2.61E-10	3.47E-09	2.03E+01	4.35E+00	2.03E+01	5.37E+01	2.64E+00
FIGN	1.63E+02	2.61E-10	3.47E-09	2.96E+00	1.57E+00	2.96E+00	1.25E+01	4.22E+00
ADAMTS2	8.40E+02	2.63E-10	3.47E-09	5.42E+00	2.44E+00	5.42E+00	7.18E+01	1.33E+01
SAMD4A	4.67E+02	2.66E-10	3.48E-09	3.49E+00	1.80E+00	3.49E+00	3.70E+01	1.06E+01
SYT11	5.56E+02	2.81E-10	3.65E-09	3.51E+00	1.81E+00	3.51E+00	4.40E+01	1.25E+01
SNX31	9.32E+01	2.93E-10	3.75E-09	8.87E+00	3.15E+00	8.87E+00	8.41E+00	9.48E-01
TBL2	4.25E+02	2.93E-10	3.75E-09	2.09E+00	1.06E+00	2.09E+00	2.94E+01	1.41E+01
VSTM4	5.86E+02	2.99E-10	3.75E-09	5.20E+00	2.38E+00	5.20E+00	4.96E+01	9.52E+00
PELI2	5.42E+02	2.99E-10	3.75E-09	3.32E+00	1.73E+00	3.32E+00	4.25E+01	1.28E+01
XR_003030698.1	7.60E+01	3.00E-10	3.75E-09	2.23E+01	4.48E+00	2.23E+01	7.30E+00	3.27E-01
SPARCL1	1.24E+04	3.01E-10	3.75E-09	7.46E+00	2.90E+00	7.46E+00	1.10E+03	1.47E+02
TRIM2	8.80E+02	3.05E-10	3.77E-09	2.89E+00	1.53E+00	2.89E+00	6.71E+01	2.33E+01
ANO4	5.69E+02	3.07E-10	3.77E-09	3.86E+00	1.95E+00	3.86E+00	4.58E+01	1.19E+01
ABLIM1	5.72E+03	3.10E-10	3.78E-09	2.79E+00	1.48E+00	2.79E+00	4.31E+02	1.54E+02
XR_003034559.1	2.28E+02	3.19E-10	3.87E-09	4.99E+00	2.32E+00	4.99E+00	1.92E+01	3.84E+00
LOC101904642	1.83E+02	3.39E-10	4.08E-09	4.08E+00	2.03E+00	4.08E+00	1.48E+01	3.64E+00
LRRK1	6.47E+02	3.42E-10	4.08E-09	2.61E+00	1.39E+00	2.61E+00	4.75E+01	1.82E+01
SULF2	4.30E+03	3.43E-10	4.08E-09	5.36E+00	2.42E+00	5.36E+00	3.66E+02	6.83E+01
XR_003036136.1	3.40E+02	3.52E-10	4.14E-09	3.11E+00	1.64E+00	3.11E+00	2.59E+01	8.32E+00
LRRK2	2.46E+02	3.54E-10	4.14E-09	4.17E+00	2.06E+00	4.17E+00	2.02E+01	4.83E+00
ADGRF5	8.77E+02	3.69E-10	4.25E-09	3.59E+00	1.85E+00	3.59E+00	6.99E+01	1.95E+01
RFTN2	4.68E+02	3.72E-10	4.25E-09	2.98E+00	1.58E+00	2.98E+00	3.58E+01	1.20E+01
SLC10A7	1.77E+02	3.73E-10	4.25E-09	2.73E+00	1.45E+00	2.73E+00	1.32E+01	4.84E+00
FOS	2.84E+03	3.73E-10	4.25E-09	8.82E+00	3.14E+00	8.82E+00	2.59E+02	2.93E+01
INSR	1.94E+03	3.75E-10	4.25E-09	3.63E+00	1.86E+00	3.63E+00	1.55E+02	4.27E+01
NUAK1	1.13E+02	3.79E-10	4.27E-09	1.01E+01	3.33E+00	1.01E+01	1.03E+01	1.02E+00
NTRK2	3.03E+03	3.83E-10	4.29E-09	1.34E+01	3.75E+00	1.34E+01	2.82E+02	2.10E+01
STUM	4.18E+02	3.87E-10	4.31E-09	3.22E+00	1.69E+00	3.22E+00	3.23E+01	1.01E+01
ENPP1	4.55E+02	3.92E-10	4.33E-09	3.78E+00	1.92E+00	3.78E+00	3.67E+01	9.71E+00

FGF2	2.99E+02	4.03E-10	4.39E-09	2.72E+00	1.44E+00	2.72E+00	2.23E+01	8.20E+00
FN1	2.12E+04	4.04E-10	4.39E-09	4.46E+00	2.16E+00	4.46E+00	1.75E+03	3.93E+02
PRKG2	4.98E+01	4.05E-10	4.39E-09	5.45E+00	2.45E+00	5.45E+00	4.24E+00	7.78E-01
CHSY1	8.81E+02	4.07E-10	4.39E-09	2.84E+00	1.50E+00	2.84E+00	6.66E+01	2.35E+01
PEAK1	1.17E+03	4.11E-10	4.41E-09	3.38E+00	1.76E+00	3.38E+00	9.22E+01	2.73E+01
CD302	4.37E+02	4.32E-10	4.60E-09	3.12E+00	1.64E+00	3.12E+00	3.38E+01	1.08E+01
IL13RA1	1.93E+03	4.39E-10	4.65E-09	2.54E+00	1.34E+00	2.54E+00	1.41E+02	5.56E+01
THBS2	9.10E+02	4.44E-10	4.68E-09	1.58E+01	3.98E+00	1.58E+01	8.60E+01	5.44E+00
LRP12	3.85E+02	4.50E-10	4.71E-09	2.46E+00	1.30E+00	2.46E+00	2.80E+01	1.14E+01
EDNRB	3.78E+02	4.55E-10	4.74E-09	9.05E+00	3.18E+00	9.05E+00	3.42E+01	3.78E+00
XR_003036597.1	3.24E+02	4.59E-10	4.75E-09	2.78E+01	4.80E+00	2.78E+01	3.14E+01	1.13E+00
CCDC142	1.73E+02	4.63E-10	4.76E-09	2.40E+00	1.26E+00	2.40E+00	1.24E+01	5.19E+00
APBB2	1.05E+03	4.86E-10	4.97E-09	2.08E+00	1.06E+00	2.08E+00	7.32E+01	3.51E+01
TIRAP	3.58E+02	4.90E-10	4.99E-09	2.89E+00	1.53E+00	2.89E+00	2.71E+01	9.35E+00
ITGB8	1.27E+02	4.99E-10	5.05E-09	4.21E+00	2.07E+00	4.21E+00	1.04E+01	2.48E+00
AOC3	2.05E+03	5.03E-10	5.05E-09	3.87E+00	1.95E+00	3.87E+00	1.65E+02	4.26E+01
SLC7A2	5.61E+02	5.05E-10	5.05E-09	2.89E+00	1.53E+00	2.89E+00	4.24E+01	1.46E+01
RIOK3	6.62E+02	5.11E-10	5.06E-09	1.70E+00	7.65E-01	1.70E+00	4.30E+01	2.53E+01
GDA	8.45E+01	5.13E-10	5.06E-09	7.19E+00	2.85E+00	7.19E+00	7.46E+00	1.04E+00
RUNX1T1	8.02E+02	5.18E-10	5.06E-09	2.91E+00	1.54E+00	2.91E+00	6.09E+01	2.09E+01
SCUBE2	1.18E+03	5.18E-10	5.06E-09	5.76E+00	2.53E+00	5.76E+00	1.01E+02	1.75E+01
C28H1orf198	6.72E+02	5.20E-10	5.06E-09	3.11E+00	1.63E+00	3.11E+00	5.16E+01	1.66E+01
ZFYVE26	7.85E+02	5.34E-10	5.16E-09	2.52E+00	1.33E+00	2.52E+00	5.75E+01	2.28E+01
IPO5	9.55E+02	5.36E-10	5.16E-09	4.77E+00	2.25E+00	4.77E+00	7.95E+01	1.67E+01
XYLT1	1.14E+03	5.51E-10	5.27E-09	4.79E+00	2.26E+00	4.79E+00	9.54E+01	1.99E+01
ZEB1	1.34E+03	5.69E-10	5.41E-09	4.25E+00	2.09E+00	4.25E+00	1.10E+02	2.58E+01
ZFP36	9.75E+02	5.71E-10	5.41E-09	3.24E+00	1.70E+00	3.24E+00	7.60E+01	2.34E+01
RAPGEF4	1.07E+02	5.81E-10	5.48E-09	4.37E+00	2.13E+00	4.37E+00	8.85E+00	2.03E+00
RNASEL	6.14E+01	5.86E-10	5.49E-09	9.21E+00	3.20E+00	9.21E+00	5.55E+00	6.03E-01
COL12A1	8.73E+03	5.90E-10	5.50E-09	4.41E+00	2.14E+00	4.41E+00	7.17E+02	1.62E+02
ANOS1	1.06E+02	6.01E-10	5.57E-09	3.47E+00	1.80E+00	3.47E+00	8.40E+00	2.42E+00
SASH1	1.04E+03	6.08E-10	5.61E-09	3.45E+00	1.79E+00	3.45E+00	8.25E+01	2.39E+01
SAP30L	2.83E+02	6.19E-10	5.65E-09	7.32E+00	2.87E+00	7.32E+00	2.50E+01	3.42E+00

LOC784451	1.18E+02	6.19E-10	5.65E-09	4.20E+00	2.07E+00	4.20E+00	9.67E+00	2.30E+00
HSPA12A	4.41E+02	6.26E-10	5.69E-09	9.62E+00	3.27E+00	9.62E+00	4.02E+01	4.18E+00
PHLDB2	1.22E+03	6.38E-10	5.74E-09	3.50E+00	1.81E+00	3.50E+00	9.72E+01	2.78E+01
THBS1	1.94E+03	6.40E-10	5.74E-09	1.04E+01	3.38E+00	1.04E+01	1.78E+02	1.70E+01
IFNAR1	3.95E+02	6.43E-10	5.74E-09	3.26E+00	1.71E+00	3.26E+00	3.07E+01	9.40E+00
KIAA0040	1.88E+02	6.48E-10	5.74E-09	4.53E+00	2.18E+00	4.53E+00	1.56E+01	3.44E+00
EGFR	1.03E+03	6.48E-10	5.74E-09	3.30E+00	1.72E+00	3.30E+00	8.08E+01	2.44E+01
GGCX	8.53E+02	6.57E-10	5.79E-09	2.57E+00	1.36E+00	2.57E+00	6.25E+01	2.43E+01
TMOD2	1.37E+02	6.63E-10	5.82E-09	3.09E+00	1.63E+00	3.09E+00	1.05E+01	3.40E+00
LOC784007	5.59E+03	6.66E-10	5.82E-09	3.23E+00	1.69E+00	3.23E+00	4.35E+02	1.35E+02
CD200	1.17E+02	6.76E-10	5.88E-09	2.45E+00	1.29E+00	2.45E+00	8.51E+00	3.47E+00
DCAF7	4.53E+02	6.81E-10	5.89E-09	2.65E+00	1.41E+00	2.65E+00	3.35E+01	1.26E+01
COL3A1	8.71E+04	7.15E-10	6.15E-09	4.09E+00	2.03E+00	4.09E+00	7.10E+03	1.74E+03
CCBE1	7.28E+01	7.29E-10	6.24E-09	5.41E+00	2.44E+00	5.41E+00	6.24E+00	1.15E+00
FLRT2	1.01E+03	7.45E-10	6.35E-09	2.22E+00	1.15E+00	2.22E+00	7.19E+01	3.24E+01
PARP8	2.83E+02	7.52E-10	6.36E-09	3.30E+00	1.72E+00	3.30E+00	2.21E+01	6.70E+00
ABCA6	3.41E+02	7.55E-10	6.36E-09	4.79E+00	2.26E+00	4.79E+00	2.85E+01	5.94E+00
CLIC4	1.36E+03	7.58E-10	6.36E-09	4.54E+00	2.18E+00	4.54E+00	1.13E+02	2.48E+01
MGAT5	8.41E+02	7.90E-10	6.60E-09	1.85E+00	8.85E-01	1.85E+00	5.67E+01	3.07E+01
DHRS12	1.66E+02	8.17E-10	6.80E-09	2.10E+00	1.07E+00	2.10E+00	1.16E+01	5.51E+00
COL1A1	1.63E+05	8.57E-10	7.06E-09	3.86E+00	1.95E+00	3.86E+00	1.31E+04	3.41E+03
MAML2	4.09E+02	8.60E-10	7.06E-09	4.30E+00	2.10E+00	4.30E+00	3.37E+01	7.83E+00
PGM5	1.59E+03	8.60E-10	7.06E-09	3.21E+00	1.68E+00	3.21E+00	1.23E+02	3.84E+01
TRIO	3.13E+03	8.69E-10	7.09E-09	2.42E+00	1.28E+00	2.42E+00	2.27E+02	9.36E+01
INPP4B	9.90E+01	8.72E-10	7.09E-09	3.10E+00	1.63E+00	3.10E+00	7.66E+00	2.47E+00
PLEKHG1	5.29E+02	8.79E-10	7.09E-09	2.94E+00	1.56E+00	2.94E+00	4.04E+01	1.37E+01
PRRG3	1.07E+03	8.80E-10	7.09E-09	2.37E+00	1.25E+00	2.37E+00	7.69E+01	3.24E+01
PDE3A	2.11E+02	9.21E-10	7.39E-09	4.24E+00	2.08E+00	4.24E+00	1.74E+01	4.11E+00
SGCD	2.33E+02	9.30E-10	7.40E-09	4.22E+00	2.08E+00	4.22E+00	1.91E+01	4.54E+00
RECK	6.24E+02	9.31E-10	7.40E-09	2.80E+00	1.48E+00	2.80E+00	4.70E+01	1.68E+01
PLEKHA2	1.57E+03	9.52E-10	7.46E-09	2.38E+00	1.25E+00	2.38E+00	1.14E+02	4.77E+01
ADGRL4	2.86E+02	9.53E-10	7.46E-09	4.19E+00	2.07E+00	4.19E+00	2.34E+01	5.59E+00
USP6NL	4.90E+02	9.54E-10	7.46E-09	1.76E+00	8.18E-01	1.76E+00	3.25E+01	1.84E+01

PARVA	2.89E+03	9.57E-10	7.46E-09	1.87E+00	9.05E-01	1.87E+00	1.94E+02	1.04E+02
ARRDC4	6.19E+02	9.59E-10	7.46E-09	4.00E+00	2.00E+00	4.00E+00	5.01E+01	1.25E+01
TM4SF1	6.84E+02	9.75E-10	7.55E-09	3.60E+00	1.85E+00	3.60E+00	5.44E+01	1.51E+01
COLGALT2	6.73E+01	9.80E-10	7.56E-09	5.35E+00	2.42E+00	5.35E+00	5.71E+00	1.07E+00
LOC789192	8.46E+02	9.97E-10	7.64E-09	1.77E+00	8.23E-01	1.77E+00	5.57E+01	3.15E+01
TGFBR3	5.26E+03	9.99E-10	7.64E-09	5.24E+00	2.39E+00	5.24E+00	4.48E+02	8.54E+01
EDEM3	5.50E+02	1.02E-09	7.77E-09	2.09E+00	1.06E+00	2.09E+00	3.83E+01	1.83E+01
FRMD4A	1.11E+03	1.04E-09	7.90E-09	2.04E+00	1.03E+00	2.04E+00	7.63E+01	3.74E+01
DKK2	5.77E+01	1.05E-09	7.90E-09	6.36E+00	2.67E+00	6.36E+00	5.03E+00	7.91E-01
MTMR10	3.84E+02	1.06E-09	7.93E-09	3.00E+00	1.58E+00	3.00E+00	2.94E+01	9.80E+00
WIPF1	4.82E+02	1.08E-09	8.07E-09	3.04E+00	1.60E+00	3.04E+00	3.69E+01	1.21E+01
LPP	2.76E+03	1.12E-09	8.32E-09	3.03E+00	1.60E+00	3.03E+00	2.13E+02	7.02E+01
ACTN1	3.78E+03	1.14E-09	8.32E-09	2.05E+00	1.04E+00	2.05E+00	2.63E+02	1.28E+02
NAPEPLD	2.65E+02	1.14E-09	8.32E-09	6.63E+00	2.73E+00	6.63E+00	2.32E+01	3.50E+00
ITGB1	8.82E+03	1.14E-09	8.32E-09	1.98E+00	9.83E-01	1.98E+00	6.05E+02	3.06E+02
GREB1	4.66E+03	1.14E-09	8.32E-09	3.35E+00	1.74E+00	3.35E+00	3.65E+02	1.09E+02
XR_805198.3	1.55E+02	1.14E-09	8.32E-09	4.13E+00	2.04E+00	4.13E+00	1.27E+01	3.08E+00
MID2	2.34E+02	1.14E-09	8.32E-09	2.74E+00	1.45E+00	2.74E+00	1.75E+01	6.39E+00
XR_003035396.1	7.99E+02	1.15E-09	8.35E-09	1.70E+01	4.08E+00	1.70E+01	7.56E+01	4.46E+00
DMD	5.46E+02	1.16E-09	8.35E-09	2.62E+00	1.39E+00	2.62E+00	4.06E+01	1.55E+01
MSN	3.93E+03	1.17E-09	8.40E-09	1.88E+00	9.14E-01	1.88E+00	2.64E+02	1.40E+02
ARMCX3	1.49E+03	1.18E-09	8.42E-09	2.26E+00	1.18E+00	2.26E+00	1.06E+02	4.70E+01
STARD9	1.39E+03	1.18E-09	8.42E-09	3.33E+00	1.74E+00	3.33E+00	1.09E+02	3.26E+01
TBC1D12	3.85E+02	1.19E-09	8.42E-09	1.88E+00	9.09E-01	1.88E+00	2.58E+01	1.37E+01
FBXL7	1.52E+03	1.21E-09	8.50E-09	2.34E+00	1.23E+00	2.34E+00	1.09E+02	4.65E+01
DBT	3.07E+02	1.21E-09	8.50E-09	2.34E+00	1.23E+00	2.34E+00	2.22E+01	9.47E+00
COL21A1	2.81E+03	1.23E-09	8.66E-09	2.86E+00	1.52E+00	2.86E+00	2.13E+02	7.42E+01
ATG4A	7.74E+01	1.25E-09	8.76E-09	2.99E+00	1.58E+00	2.99E+00	5.89E+00	1.97E+00
MECOM	2.46E+02	1.30E-09	9.03E-09	3.85E+00	1.94E+00	3.85E+00	1.99E+01	5.16E+00
CFAP97	3.55E+02	1.30E-09	9.03E-09	2.49E+00	1.31E+00	2.49E+00	2.60E+01	1.05E+01
NID1	9.52E+03	1.31E-09	9.05E-09	3.50E+00	1.81E+00	3.50E+00	7.51E+02	2.15E+02
NEURL1B	2.13E+03	1.37E-09	9.42E-09	3.35E+00	1.74E+00	3.35E+00	1.66E+02	4.97E+01
LAMB1	8.78E+03	1.42E-09	9.73E-09	2.94E+00	1.56E+00	2.94E+00	6.71E+02	2.28E+02

LAMB2	3.39E+03	1.43E-09	9.73E-09	1.75E+00	8.04E-01	1.75E+00	2.23E+02	1.28E+02
GCNT2	2.59E+01	1.46E-09	9.94E-09	5.62E+00	2.49E+00	5.62E+00	2.22E+00	3.96E-01
PTPRB	4.65E+02	1.47E-09	9.99E-09	4.28E+00	2.10E+00	4.28E+00	3.83E+01	8.95E+00
TMOD3	5.51E+02	1.48E-09	1.00E-08	2.86E+00	1.52E+00	2.86E+00	4.18E+01	1.46E+01
ADAM19	1.27E+03	1.53E-09	1.02E-08	1.84E+00	8.78E-01	1.84E+00	8.51E+01	4.63E+01
BACE1	1.25E+03	1.53E-09	1.02E-08	2.35E+00	1.23E+00	2.35E+00	9.02E+01	3.83E+01
EML1	5.18E+02	1.57E-09	1.04E-08	2.07E+00	1.05E+00	2.07E+00	3.60E+01	1.74E+01
MLLT3	6.41E+02	1.58E-09	1.05E-08	1.97E+00	9.80E-01	1.97E+00	4.38E+01	2.22E+01
XR_003029755.1	3.38E+02	1.70E-09	1.12E-08	4.38E+00	2.13E+00	4.38E+00	2.79E+01	6.37E+00
PDE8B	9.85E+02	1.71E-09	1.13E-08	3.11E+00	1.64E+00	3.11E+00	7.57E+01	2.44E+01
PARD3	1.97E+03	1.72E-09	1.13E-08	2.07E+00	1.05E+00	2.07E+00	1.37E+02	6.61E+01
CALHM5	4.77E+01	1.73E-09	1.13E-08	1.17E+01	3.54E+00	1.17E+01	4.40E+00	3.78E-01
SYT1	9.25E+02	1.74E-09	1.14E-08	5.46E+00	2.45E+00	5.46E+00	7.91E+01	1.45E+01
XR_003035418.1	1.68E+02	1.76E-09	1.14E-08	3.83E+00	1.94E+00	3.83E+00	1.35E+01	3.53E+00
CD99	6.16E+03	1.78E-09	1.16E-08	1.95E+00	9.64E-01	1.95E+00	4.18E+02	2.14E+02
XR_003030575.1	3.83E+02	1.84E-09	1.19E-08	3.89E+00	1.96E+00	3.89E+00	3.09E+01	7.93E+00
ARHGEF3	5.70E+02	1.87E-09	1.20E-08	2.43E+00	1.28E+00	2.43E+00	4.15E+01	1.71E+01
PTPN21	1.40E+03	1.87E-09	1.20E-08	2.34E+00	1.23E+00	2.34E+00	1.01E+02	4.29E+01
ABCB1	8.21E+02	1.89E-09	1.21E-08	2.68E+00	1.42E+00	2.68E+00	6.15E+01	2.29E+01
XR_003032007.1	8.46E+00	1.91E-09	1.21E-08	1.35E+01	3.76E+00	1.35E+01	7.89E-01	5.84E-02
CDON	1.45E+03	1.92E-09	1.21E-08	2.24E+00	1.17E+00	2.24E+00	1.03E+02	4.58E+01
SCN2A	8.16E+01	1.93E-09	1.21E-08	2.54E+00	1.35E+00	2.54E+00	6.03E+00	2.37E+00
SCUBE3	7.72E+02	1.93E-09	1.21E-08	8.14E+00	3.03E+00	8.14E+00	6.94E+01	8.53E+00
ITGA9	6.02E+02	1.94E-09	1.21E-08	5.54E+00	2.47E+00	5.54E+00	5.15E+01	9.30E+00
SP140L	4.41E+01	1.94E-09	1.21E-08	6.55E+00	2.71E+00	6.55E+00	3.85E+00	5.87E-01
NNT	6.78E+02	1.98E-09	1.23E-08	2.25E+00	1.17E+00	2.25E+00	4.82E+01	2.15E+01
MICU3	1.67E+02	1.98E-09	1.23E-08	2.67E+00	1.42E+00	2.67E+00	1.23E+01	4.61E+00
HSPB6	2.63E+03	1.98E-09	1.23E-08	2.80E+00	1.48E+00	2.80E+00	1.98E+02	7.06E+01
FAM124B	1.23E+02	2.01E-09	1.24E-08	3.07E+00	1.62E+00	3.07E+00	9.52E+00	3.10E+00
COL1A2	7.73E+04	2.04E-09	1.25E-08	3.44E+00	1.78E+00	3.44E+00	6.10E+03	1.78E+03
GRK3	3.62E+02	2.04E-09	1.25E-08	2.40E+00	1.26E+00	2.40E+00	2.61E+01	1.09E+01
FZD4	2.59E+02	2.06E-09	1.26E-08	4.90E+00	2.29E+00	4.90E+00	2.19E+01	4.46E+00
SNX18	7.78E+02	2.14E-09	1.30E-08	2.47E+00	1.31E+00	2.47E+00	5.70E+01	2.31E+01

CCDC141	1.26E+02	2.15E-09	1.30E-08	3.66E+00	1.87E+00	3.66E+00	1.00E+01	2.74E+00
IKBIP	9.61E+02	2.16E-09	1.30E-08	1.83E+00	8.75E-01	1.83E+00	6.43E+01	3.51E+01
ABCA9	5.41E+02	2.21E-09	1.33E-08	5.13E+00	2.36E+00	5.13E+00	4.56E+01	8.89E+00
TCF4	1.15E+03	2.26E-09	1.35E-08	2.35E+00	1.23E+00	2.35E+00	8.28E+01	3.53E+01
EEF2K	1.76E+03	2.26E-09	1.35E-08	2.52E+00	1.34E+00	2.52E+00	1.29E+02	5.12E+01
NLRP1	4.93E+01	2.27E-09	1.35E-08	5.24E+00	2.39E+00	5.24E+00	4.19E+00	8.01E-01
MTPN	8.01E+02	2.28E-09	1.35E-08	3.83E+00	1.94E+00	3.83E+00	6.41E+01	1.67E+01
FBXL3	8.57E+02	2.33E-09	1.37E-08	2.13E+00	1.09E+00	2.13E+00	5.99E+01	2.81E+01
ZNF366	5.90E+01	2.33E-09	1.37E-08	9.75E+00	3.29E+00	9.75E+00	5.36E+00	5.50E-01
CC2D2A	4.63E+02	2.35E-09	1.38E-08	2.40E+00	1.27E+00	2.40E+00	3.37E+01	1.40E+01
MTMR12	4.14E+02	2.39E-09	1.40E-08	2.19E+00	1.13E+00	2.19E+00	2.92E+01	1.34E+01
VPS13B	1.09E+03	2.41E-09	1.40E-08	2.14E+00	1.09E+00	2.14E+00	7.65E+01	3.58E+01
IGSF3	2.84E+02	2.42E-09	1.41E-08	6.10E+00	2.61E+00	6.10E+00	2.46E+01	4.03E+00
PER3	4.16E+02	2.45E-09	1.42E-08	2.53E+00	1.34E+00	2.53E+00	3.05E+01	1.21E+01
GPC5	1.51E+03	2.48E-09	1.44E-08	2.80E+00	1.48E+00	2.80E+00	1.13E+02	4.05E+01
NKAIN3	7.96E+01	2.52E-09	1.45E-08	2.77E+00	1.47E+00	2.77E+00	5.98E+00	2.16E+00
ACER2	3.06E+02	2.58E-09	1.48E-08	2.27E+00	1.18E+00	2.27E+00	2.18E+01	9.61E+00
ZNF391	4.06E+01	2.62E-09	1.50E-08	2.85E+00	1.51E+00	2.85E+00	3.06E+00	1.07E+00
B3GNT3	2.23E+02	2.63E-09	1.50E-08	2.27E+00	1.18E+00	2.27E+00	1.60E+01	7.05E+00
ANKRD27	9.05E+02	2.69E-09	1.53E-08	1.72E+00	7.83E-01	1.72E+00	5.92E+01	3.44E+01
TMEM140	1.34E+02	2.72E-09	1.54E-08	3.06E+00	1.61E+00	3.06E+00	1.03E+01	3.36E+00
CLCC1	1.40E+03	2.72E-09	1.54E-08	1.88E+00	9.11E-01	1.88E+00	9.43E+01	5.01E+01
ZNF318	6.70E+02	2.74E-09	1.54E-08	2.11E+00	1.08E+00	2.11E+00	4.69E+01	2.23E+01
RNF152	1.59E+02	2.75E-09	1.54E-08	3.36E+00	1.75E+00	3.36E+00	1.26E+01	3.74E+00
AFAP1L1	4.08E+02	2.79E-09	1.56E-08	2.57E+00	1.36E+00	2.57E+00	3.00E+01	1.17E+01
MYLIP	9.94E+02	2.79E-09	1.56E-08	2.40E+00	1.26E+00	2.40E+00	7.16E+01	2.98E+01
OSBPL6	4.63E+02	2.81E-09	1.56E-08	1.72E+00	7.86E-01	1.72E+00	3.04E+01	1.76E+01
VCL	4.43E+03	2.82E-09	1.56E-08	2.46E+00	1.30E+00	2.46E+00	3.23E+02	1.32E+02
XR_003038221.1	2.83E+00	2.82E-09	1.56E-08	6.78E+00	2.76E+00	6.78E+00	2.47E-01	3.64E-02
CP	1.55E+02	2.82E-09	1.56E-08	2.58E+00	1.37E+00	2.58E+00	1.14E+01	4.40E+00
MOB3B	6.78E+02	2.83E-09	1.56E-08	2.63E+00	1.40E+00	2.63E+00	5.03E+01	1.91E+01
ZNF697	6.50E+02	2.85E-09	1.56E-08	2.38E+00	1.25E+00	2.38E+00	4.70E+01	1.97E+01
SERINC3	1.19E+03	2.86E-09	1.56E-08	1.83E+00	8.74E-01	1.83E+00	7.90E+01	4.31E+01

HRH1	1.59E+02	2.90E-09	1.58E-08	3.08E+00	1.62E+00	3.08E+00	1.22E+01	3.96E+00
PDZRN3	1.24E+03	2.93E-09	1.59E-08	2.53E+00	1.34E+00	2.53E+00	9.13E+01	3.61E+01
FXYD1	6.05E+02	2.98E-09	1.61E-08	2.72E+00	1.45E+00	2.72E+00	4.51E+01	1.66E+01
XR_001495344.2	5.19E+02	2.99E-09	1.62E-08	2.52E+00	1.33E+00	2.52E+00	3.78E+01	1.50E+01
JAM2	9.07E+02	3.05E-09	1.64E-08	3.10E+00	1.63E+00	3.10E+00	7.00E+01	2.26E+01
SLC39A14	1.10E+03	3.06E-09	1.64E-08	3.43E+00	1.78E+00	3.43E+00	8.68E+01	2.53E+01
XR_003029634.1	1.51E+02	3.24E-09	1.73E-08	1.25E+01	3.64E+00	1.25E+01	1.40E+01	1.12E+00
MCL1	2.68E+03	3.28E-09	1.75E-08	1.97E+00	9.76E-01	1.97E+00	1.84E+02	9.36E+01
PCYT1A	7.72E+02	3.30E-09	1.76E-08	1.99E+00	9.89E-01	1.99E+00	5.28E+01	2.66E+01
MEF2C	8.16E+02	3.32E-09	1.76E-08	2.06E+00	1.04E+00	2.06E+00	5.66E+01	2.76E+01
ATP7A	2.43E+02	3.33E-09	1.76E-08	2.50E+00	1.32E+00	2.50E+00	1.78E+01	7.12E+00
LOC112441542	5.78E+01	3.40E-09	1.79E-08	5.98E+00	2.58E+00	5.98E+00	5.00E+00	8.36E-01
MITF	3.97E+02	3.43E-09	1.80E-08	5.25E+00	2.39E+00	5.25E+00	3.38E+01	6.44E+00
LOC101902366	4.53E+02	3.46E-09	1.81E-08	1.77E+00	8.27E-01	1.77E+00	2.98E+01	1.68E+01
PCOLCE2	2.45E+02	3.50E-09	1.83E-08	2.87E+00	1.52E+00	2.87E+00	1.85E+01	6.46E+00
CCDC80	3.03E+03	3.55E-09	1.85E-08	4.24E+00	2.08E+00	4.24E+00	2.49E+02	5.87E+01
PECAM1	1.39E+03	3.83E-09	1.99E-08	2.38E+00	1.25E+00	2.38E+00	1.00E+02	4.21E+01
UTRN	1.56E+03	3.86E-09	2.00E-08	2.26E+00	1.18E+00	2.26E+00	1.12E+02	4.95E+01
NR1H4	1.84E+02	4.01E-09	2.07E-08	4.42E+00	2.14E+00	4.42E+00	1.51E+01	3.42E+00
SREK1IP1	4.58E+02	4.04E-09	2.08E-08	1.90E+00	9.25E-01	1.90E+00	3.10E+01	1.63E+01
CDK14	9.05E+02	4.21E-09	2.16E-08	2.33E+00	1.22E+00	2.33E+00	6.52E+01	2.80E+01
ITGA1	7.83E+02	4.26E-09	2.18E-08	7.14E+00	2.84E+00	7.14E+00	6.93E+01	9.72E+00
XR_233332.4	5.96E+01	4.33E-09	2.21E-08	9.54E+00	3.25E+00	9.54E+00	5.42E+00	5.68E-01
MTCL1	6.92E+02	4.34E-09	2.21E-08	2.88E+00	1.53E+00	2.88E+00	5.24E+01	1.82E+01
CEACAM1	3.60E+02	4.40E-09	2.23E-08	2.59E+00	1.38E+00	2.59E+00	2.65E+01	1.02E+01
RNF169	7.10E+02	4.46E-09	2.25E-08	1.75E+00	8.08E-01	1.75E+00	4.68E+01	2.67E+01
LPGAT1	8.14E+02	4.47E-09	2.25E-08	2.30E+00	1.20E+00	2.30E+00	5.81E+01	2.53E+01
CASC4	1.44E+03	4.50E-09	2.26E-08	2.02E+00	1.02E+00	2.02E+00	9.89E+01	4.89E+01
Sep-08	1.99E+03	4.54E-09	2.27E-08	1.94E+00	9.56E-01	1.94E+00	1.35E+02	6.95E+01
ZNF704	4.29E+02	4.55E-09	2.27E-08	2.41E+00	1.27E+00	2.41E+00	3.11E+01	1.29E+01
XR_235579.4	7.55E+01	4.56E-09	2.27E-08	3.07E+00	1.62E+00	3.07E+00	5.78E+00	1.88E+00
GHR	1.53E+02	4.69E-09	2.33E-08	8.06E+00	3.01E+00	8.06E+00	1.38E+01	1.71E+00
ECHDC3	3.39E+03	4.76E-09	2.36E-08	1.92E+00	9.45E-01	1.92E+00	2.30E+02	1.20E+02

LOC507055	8.63E+01	4.80E-09	2.37E-08	4.23E+00	2.08E+00	4.23E+00	7.06E+00	1.67E+00
ITSN1	1.41E+03	4.81E-09	2.37E-08	2.05E+00	1.04E+00	2.05E+00	9.75E+01	4.75E+01
FILIP1	4.81E+02	4.92E-09	2.42E-08	3.25E+00	1.70E+00	3.25E+00	3.77E+01	1.16E+01
XR_003029603.1	6.87E+01	4.98E-09	2.44E-08	6.09E+01	5.93E+00	6.09E+01	6.76E+00	1.11E-01
CAMSAP2	1.45E+03	5.02E-09	2.45E-08	2.68E+00	1.42E+00	2.68E+00	1.08E+02	4.04E+01
MB21D2	8.52E+01	5.03E-09	2.45E-08	3.31E+00	1.73E+00	3.31E+00	6.66E+00	2.01E+00
RHOBTB1	8.01E+02	5.04E-09	2.45E-08	2.69E+00	1.43E+00	2.69E+00	5.96E+01	2.22E+01
ZNF226	1.28E+03	5.07E-09	2.46E-08	1.89E+00	9.15E-01	1.89E+00	8.63E+01	4.58E+01
XR_813933.3	4.36E+01	5.15E-09	2.49E-08	7.28E+00	2.86E+00	7.28E+00	3.87E+00	5.32E-01
ERLIN2	3.10E+03	5.16E-09	2.49E-08	1.93E+00	9.48E-01	1.93E+00	2.09E+02	1.09E+02
GLI2	1.41E+03	5.40E-09	2.60E-08	2.57E+00	1.36E+00	2.57E+00	1.04E+02	4.05E+01
BROX	5.70E+02	5.52E-09	2.65E-08	1.88E+00	9.11E-01	1.88E+00	3.83E+01	2.04E+01
CPNE8	6.08E+01	5.72E-09	2.74E-08	6.18E+00	2.63E+00	6.18E+00	5.27E+00	8.53E-01
NEK7	7.36E+02	5.79E-09	2.76E-08	1.75E+00	8.07E-01	1.75E+00	4.83E+01	2.76E+01
SVEP1	3.50E+02	5.80E-09	2.76E-08	4.77E+00	2.25E+00	4.77E+00	2.93E+01	6.15E+00
PIK3CA	5.33E+02	5.83E-09	2.77E-08	1.71E+00	7.70E-01	1.71E+00	3.48E+01	2.04E+01
SMIM14	5.54E+02	5.85E-09	2.77E-08	2.05E+00	1.03E+00	2.05E+00	3.84E+01	1.87E+01
ADAMTS18	4.60E+01	5.93E-09	2.80E-08	3.99E+00	2.00E+00	3.99E+00	3.73E+00	9.35E-01
SELENOP	5.31E+03	5.97E-09	2.81E-08	2.53E+00	1.34E+00	2.53E+00	3.89E+02	1.54E+02
PRNP	1.07E+03	5.97E-09	2.81E-08	2.03E+00	1.02E+00	2.03E+00	7.37E+01	3.63E+01
XR_813947.3	3.15E+02	6.10E-09	2.86E-08	5.17E+00	2.37E+00	5.17E+00	2.68E+01	5.18E+00
B3GNT5	1.06E+02	6.14E-09	2.87E-08	3.52E+00	1.81E+00	3.52E+00	8.49E+00	2.41E+00
OPHN1	5.48E+02	6.33E-09	2.95E-08	2.55E+00	1.35E+00	2.55E+00	4.04E+01	1.58E+01
SDC2	4.12E+03	6.34E-09	2.95E-08	3.40E+00	1.77E+00	3.40E+00	3.25E+02	9.55E+01
TMEM100	1.54E+03	6.52E-09	3.02E-08	4.12E+00	2.04E+00	4.12E+00	1.24E+02	3.02E+01
TTC37	7.99E+02	6.52E-09	3.02E-08	1.97E+00	9.77E-01	1.97E+00	5.46E+01	2.77E+01
SH3PXD2A	7.90E+03	6.54E-09	3.02E-08	3.21E+00	1.68E+00	3.21E+00	6.13E+02	1.91E+02
PCOLCE	2.04E+03	6.58E-09	3.03E-08	2.63E+00	1.39E+00	2.63E+00	1.51E+02	5.76E+01
SETMAR	3.90E+02	6.77E-09	3.11E-08	2.22E+00	1.15E+00	2.22E+00	2.75E+01	1.24E+01
ANTXR2	1.53E+03	6.85E-09	3.14E-08	3.10E+00	1.63E+00	3.10E+00	1.18E+02	3.81E+01
PLCB1	2.68E+02	6.90E-09	3.15E-08	3.50E+00	1.81E+00	3.50E+00	2.13E+01	6.08E+00
TTL	2.28E+02	7.06E-09	3.21E-08	2.57E+00	1.36E+00	2.57E+00	1.67E+01	6.51E+00
SLC30A5	5.32E+02	7.07E-09	3.21E-08	2.06E+00	1.04E+00	2.06E+00	3.70E+01	1.80E+01

POC1B	3.05E+02	7.09E-09	3.21E-08	1.85E+00	8.90E-01	1.85E+00	2.04E+01	1.10E+01
HIPK3	7.80E+02	7.14E-09	3.23E-08	2.05E+00	1.04E+00	2.05E+00	5.41E+01	2.63E+01
FAM185A	1.53E+02	7.25E-09	3.27E-08	3.36E+00	1.75E+00	3.36E+00	1.20E+01	3.58E+00
HIPK1	1.57E+03	7.32E-09	3.29E-08	2.57E+00	1.36E+00	2.57E+00	1.16E+02	4.53E+01
CYP4V2	9.36E+02	7.38E-09	3.31E-08	2.18E+00	1.12E+00	2.18E+00	6.60E+01	3.03E+01
CFH	2.18E+03	7.39E-09	3.31E-08	1.27E+01	3.67E+00	1.27E+01	2.03E+02	1.60E+01
MAP3K21	2.46E+02	7.63E-09	3.41E-08	3.57E+00	1.84E+00	3.57E+00	1.95E+01	5.46E+00
PTPN14	1.12E+03	7.80E-09	3.47E-08	2.74E+00	1.46E+00	2.74E+00	8.42E+01	3.07E+01
CRTC3	1.76E+03	7.85E-09	3.49E-08	1.85E+00	8.86E-01	1.85E+00	1.18E+02	6.37E+01
PSD3	1.23E+03	8.22E-09	3.64E-08	2.12E+00	1.09E+00	2.12E+00	8.57E+01	4.04E+01
PRKACB	8.30E+02	8.24E-09	3.64E-08	1.90E+00	9.29E-01	1.90E+00	5.63E+01	2.95E+01
DDR2	9.66E+02	8.26E-09	3.64E-08	2.91E+00	1.54E+00	2.91E+00	7.38E+01	2.53E+01
LRRC8B	2.74E+02	8.29E-09	3.65E-08	1.98E+00	9.88E-01	1.98E+00	1.88E+01	9.46E+00
SYTL4	3.58E+02	8.50E-09	3.73E-08	1.69E+00	7.59E-01	1.69E+00	2.32E+01	1.37E+01
STON1	3.96E+02	8.54E-09	3.74E-08	2.74E+00	1.45E+00	2.74E+00	2.98E+01	1.09E+01
LOC101906235	3.17E+01	8.57E-09	3.74E-08	3.87E+00	1.95E+00	3.87E+00	2.56E+00	6.62E-01
LOC527414	9.96E+00	8.61E-09	3.75E-08	1.04E+02	6.70E+00	1.04E+02	9.87E-01	9.52E-03
MYCT1	2.18E+02	8.63E-09	3.75E-08	3.02E+00	1.59E+00	3.02E+00	1.67E+01	5.53E+00
SPARC	6.06E+04	8.89E-09	3.85E-08	2.56E+00	1.36E+00	2.56E+00	4.47E+03	1.74E+03
RAB8B	5.76E+02	8.90E-09	3.85E-08	2.23E+00	1.16E+00	2.23E+00	4.08E+01	1.83E+01
ZEB2	7.02E+01	8.98E-09	3.87E-08	3.02E+01	4.92E+00	3.02E+01	6.81E+00	2.26E-01
DIP2A	1.03E+03	9.15E-09	3.94E-08	1.92E+00	9.43E-01	1.92E+00	7.03E+01	3.65E+01
XR_003030590.1	2.54E+02	9.21E-09	3.95E-08	2.05E+00	1.04E+00	2.05E+00	1.76E+01	8.59E+00
EPS8	3.23E+02	9.25E-09	3.96E-08	2.15E+00	1.11E+00	2.15E+00	2.26E+01	1.05E+01
ADAMTS1	3.69E+03	9.27E-09	3.96E-08	6.64E+00	2.73E+00	6.64E+00	3.24E+02	4.88E+01
PRRG1	2.64E+02	9.33E-09	3.98E-08	2.21E+00	1.14E+00	2.21E+00	1.87E+01	8.45E+00
DENND6A	5.13E+02	9.40E-09	3.99E-08	1.98E+00	9.83E-01	1.98E+00	3.51E+01	1.78E+01
SRP72	8.30E+02	9.47E-09	4.01E-08	1.87E+00	9.01E-01	1.87E+00	5.57E+01	2.98E+01
LOC100848991	1.75E+02	9.48E-09	4.01E-08	2.32E+00	1.21E+00	2.32E+00	1.25E+01	5.39E+00
XR_003029636.1	1.85E+02	9.62E-09	4.06E-08	4.09E+00	2.03E+00	4.09E+00	1.51E+01	3.68E+00
GFOD1	2.21E+02	9.65E-09	4.06E-08	2.34E+00	1.23E+00	2.34E+00	1.59E+01	6.79E+00
LMCD1	1.51E+03	9.82E-09	4.13E-08	3.81E+00	1.93E+00	3.81E+00	1.22E+02	3.20E+01
MFN1	7.41E+02	9.95E-09	4.17E-08	2.01E+00	1.01E+00	2.01E+00	5.09E+01	2.53E+01

XR_003037702.1	2.33E+02	1.01E-08	4.23E-08	5.10E+00	2.35E+00	5.10E+00	1.97E+01	3.85E+00
XR_003032005.1	1.07E+01	1.02E-08	4.26E-08	8.98E+00	3.17E+00	8.98E+00	9.69E-01	1.08E-01
L3MBTL3	6.07E+02	1.03E-08	4.29E-08	1.86E+00	8.93E-01	1.86E+00	4.06E+01	2.19E+01
CCDC102B	2.53E+01	1.06E-08	4.41E-08	7.02E+00	2.81E+00	7.02E+00	2.22E+00	3.17E-01
NOTCH2	7.22E+03	1.09E-08	4.51E-08	3.13E+00	1.64E+00	3.13E+00	5.60E+02	1.79E+02
SSFA2	7.45E+02	1.10E-08	4.55E-08	2.50E+00	1.32E+00	2.50E+00	5.44E+01	2.18E+01
ZSCAN30	6.27E+01	1.10E-08	4.55E-08	3.46E+00	1.79E+00	3.46E+00	4.93E+00	1.43E+00
LRRC8C	7.25E+01	1.13E-08	4.65E-08	3.33E+00	1.74E+00	3.33E+00	5.68E+00	1.70E+00
SSR3	2.22E+03	1.14E-08	4.66E-08	1.58E+00	6.62E-01	1.58E+00	1.41E+02	8.95E+01
SLAIN2	7.42E+02	1.15E-08	4.69E-08	1.92E+00	9.42E-01	1.92E+00	5.03E+01	2.62E+01
ERAP1	3.07E+02	1.17E-08	4.77E-08	3.47E+00	1.80E+00	3.47E+00	2.42E+01	6.96E+00
OLFML1	1.31E+03	1.18E-08	4.80E-08	2.06E+00	1.04E+00	2.06E+00	9.05E+01	4.40E+01
C19H17orf75	5.32E+02	1.19E-08	4.84E-08	1.71E+00	7.75E-01	1.71E+00	3.47E+01	2.03E+01
XR_235055.4	1.77E+02	1.20E-08	4.86E-08	3.54E+00	1.82E+00	3.54E+00	1.40E+01	3.95E+00
GSN	9.17E+03	1.21E-08	4.89E-08	3.07E+00	1.62E+00	3.07E+00	7.06E+02	2.30E+02
ZFP36L2	3.28E+03	1.21E-08	4.89E-08	1.97E+00	9.78E-01	1.97E+00	2.24E+02	1.14E+02
ATL3	1.19E+03	1.22E-08	4.91E-08	1.73E+00	7.93E-01	1.73E+00	7.82E+01	4.51E+01
XR_003036004.1	1.87E+02	1.22E-08	4.92E-08	5.08E+00	2.34E+00	5.08E+00	1.58E+01	3.11E+00
CD46	2.23E+03	1.25E-08	5.02E-08	1.80E+00	8.51E-01	1.80E+00	1.48E+02	8.22E+01
SPART	1.44E+03	1.26E-08	5.04E-08	1.81E+00	8.55E-01	1.81E+00	9.55E+01	5.28E+01
LOC100298868	6.48E+01	1.26E-08	5.04E-08	3.08E+00	1.62E+00	3.08E+00	4.97E+00	1.62E+00
C23H6orf89	2.27E+03	1.27E-08	5.05E-08	1.61E+00	6.90E-01	1.61E+00	1.45E+02	8.98E+01
POFUT1	1.31E+03	1.33E-08	5.27E-08	1.70E+00	7.66E-01	1.70E+00	8.49E+01	4.99E+01
SLC44A1	9.37E+02	1.36E-08	5.36E-08	2.05E+00	1.04E+00	2.05E+00	6.52E+01	3.18E+01
LOC521981	1.27E+02	1.36E-08	5.36E-08	5.35E+00	2.42E+00	5.35E+00	1.09E+01	2.03E+00
CNTN1	1.99E+02	1.36E-08	5.36E-08	5.58E+00	2.48E+00	5.58E+00	1.70E+01	3.05E+00
DOCK9	1.09E+03	1.37E-08	5.42E-08	2.19E+00	1.13E+00	2.19E+00	7.68E+01	3.51E+01
CCDC47	6.49E+02	1.39E-08	5.48E-08	1.72E+00	7.81E-01	1.72E+00	4.24E+01	2.47E+01
NRIP2	3.05E+02	1.40E-08	5.50E-08	4.23E+00	2.08E+00	4.23E+00	2.49E+01	5.90E+00
ABI1	1.19E+03	1.41E-08	5.53E-08	1.46E+00	5.42E-01	1.46E+00	7.31E+01	5.02E+01
C7	2.03E+03	1.43E-08	5.60E-08	4.00E+00	2.00E+00	4.00E+00	1.64E+02	4.11E+01
SP140	5.99E+02	1.48E-08	5.76E-08	1.68E+00	7.52E-01	1.68E+00	3.87E+01	2.30E+01
CHRM3	3.40E+01	1.49E-08	5.78E-08	4.78E+00	2.26E+00	4.78E+00	2.85E+00	5.96E-01

KR_003032918.1	1.36E+02	1.50E-08	5.83E-08	3.00E+00	1.58E+00	3.00E+00	1.04E+01	3.46E+00
KR_003029971.1	1.23E+02	1.54E-08	5.95E-08	2.59E+00	1.37E+00	2.59E+00	9.10E+00	3.51E+00
MYOF	2.20E+03	1.54E-08	5.96E-08	2.17E+00	1.12E+00	2.17E+00	1.56E+02	7.18E+01
FRYL	5.48E+02	1.55E-08	5.97E-08	1.84E+00	8.77E-01	1.84E+00	3.68E+01	2.00E+01
DLC1	1.47E+03	1.58E-08	6.07E-08	3.01E+00	1.59E+00	3.01E+00	1.13E+02	3.76E+01
HACD2	2.28E+02	1.59E-08	6.11E-08	2.04E+00	1.03E+00	2.04E+00	1.57E+01	7.70E+00
WWC3	1.17E+03	1.60E-08	6.13E-08	1.77E+00	8.24E-01	1.77E+00	7.71E+01	4.35E+01
C2CD5	7.03E+02	1.61E-08	6.14E-08	1.90E+00	9.27E-01	1.90E+00	4.77E+01	2.51E+01
ARHGAP28	1.44E+03	1.63E-08	6.23E-08	2.60E+00	1.38E+00	2.60E+00	1.07E+02	4.11E+01
MAP3K20	8.49E+02	1.65E-08	6.26E-08	2.71E+00	1.44E+00	2.71E+00	6.36E+01	2.35E+01
JDP2	3.66E+02	1.65E-08	6.28E-08	2.62E+00	1.39E+00	2.62E+00	2.71E+01	1.04E+01
VSIG10	5.30E+02	1.69E-08	6.42E-08	1.73E+00	7.93E-01	1.73E+00	3.48E+01	2.01E+01
GANC	9.69E+01	1.71E-08	6.46E-08	2.52E+00	1.33E+00	2.52E+00	7.05E+00	2.80E+00
LOC100139670	1.16E+02	1.74E-08	6.57E-08	2.97E+00	1.57E+00	2.97E+00	8.73E+00	2.94E+00
C1QTNF9	2.50E+01	1.75E-08	6.57E-08	1.04E+01	3.37E+00	1.04E+01	2.29E+00	2.21E-01
MEOX2	5.97E+01	1.77E-08	6.66E-08	8.36E+00	3.06E+00	8.36E+00	5.39E+00	6.46E-01
TEAD1	1.71E+03	1.79E-08	6.70E-08	2.30E+00	1.20E+00	2.30E+00	1.22E+02	5.31E+01
SEMA3C	2.25E+02	1.81E-08	6.76E-08	3.64E+00	1.86E+00	3.64E+00	1.80E+01	4.94E+00
LOC100138922	2.96E+01	1.81E-08	6.76E-08	5.80E+00	2.54E+00	5.80E+00	2.54E+00	4.38E-01
CALU	4.18E+03	1.83E-08	6.81E-08	1.53E+00	6.11E-01	1.53E+00	2.62E+02	1.72E+02
KR_003036623.1	6.32E+02	1.87E-08	6.96E-08	1.97E+00	9.81E-01	1.97E+00	4.34E+01	2.20E+01
KR_003037048.1	4.50E+01	1.92E-08	7.11E-08	1.35E+01	3.76E+00	1.35E+01	4.21E+00	3.11E-01
MYO18A	1.36E+03	1.94E-08	7.20E-08	2.16E+00	1.11E+00	2.16E+00	9.53E+01	4.41E+01
TCP11L2	3.65E+02	1.98E-08	7.32E-08	1.98E+00	9.86E-01	1.98E+00	2.50E+01	1.26E+01
PARP4	4.46E+02	1.99E-08	7.32E-08	1.83E+00	8.73E-01	1.83E+00	2.97E+01	1.62E+01
ITGA2	3.37E+02	2.01E-08	7.38E-08	3.38E+00	1.76E+00	3.38E+00	2.66E+01	7.86E+00
CRK	1.42E+03	2.01E-08	7.39E-08	1.63E+00	7.00E-01	1.63E+00	9.09E+01	5.60E+01
IFNAR2	4.12E+02	2.04E-08	7.49E-08	1.68E+00	7.46E-01	1.68E+00	2.67E+01	1.59E+01
FOXN3	1.77E+03	2.05E-08	7.49E-08	1.80E+00	8.46E-01	1.80E+00	1.18E+02	6.56E+01
IL1RAP	1.16E+02	2.06E-08	7.52E-08	2.69E+00	1.43E+00	2.69E+00	8.75E+00	3.25E+00
PMEPA1	3.45E+03	2.08E-08	7.57E-08	2.79E+00	1.48E+00	2.79E+00	2.59E+02	9.30E+01
KDELC2	8.34E+02	2.15E-08	7.82E-08	1.84E+00	8.82E-01	1.84E+00	5.60E+01	3.04E+01
CADM1	8.80E+02	2.16E-08	7.82E-08	2.73E+00	1.45E+00	2.73E+00	6.63E+01	2.43E+01

DIAPH2-2	3.53E+02	2.24E-08	8.09E-08	1.78E+00	8.29E-01	1.78E+00	2.34E+01	1.32E+01
FBXO33	7.49E+01	2.30E-08	8.31E-08	2.98E+00	1.58E+00	2.98E+00	5.69E+00	1.91E+00
RABGAP1L-2	5.13E+02	2.33E-08	8.40E-08	1.62E+00	6.99E-01	1.62E+00	3.30E+01	2.03E+01
RECQL	3.01E+02	2.34E-08	8.41E-08	1.81E+00	8.56E-01	1.81E+00	1.99E+01	1.10E+01
LOC107131134	2.12E+02	2.35E-08	8.43E-08	3.67E+00	1.88E+00	3.67E+00	1.69E+01	4.60E+00
GBE1	6.27E+02	2.40E-08	8.59E-08	1.96E+00	9.70E-01	1.96E+00	4.30E+01	2.20E+01
ETS1	2.25E+03	2.43E-08	8.67E-08	1.98E+00	9.88E-01	1.98E+00	1.54E+02	7.79E+01
SGPL1	9.82E+02	2.45E-08	8.74E-08	1.60E+00	6.79E-01	1.60E+00	6.24E+01	3.90E+01
PDE7B	6.46E+02	2.45E-08	8.74E-08	2.35E+00	1.23E+00	2.35E+00	4.67E+01	1.99E+01
TGM2	5.57E+03	2.49E-08	8.86E-08	2.68E+00	1.42E+00	2.68E+00	4.13E+02	1.54E+02
FLI1	5.62E+02	2.50E-08	8.86E-08	2.09E+00	1.07E+00	2.09E+00	3.91E+01	1.87E+01
ANK2	6.55E+02	2.50E-08	8.86E-08	2.61E+00	1.38E+00	2.61E+00	4.85E+01	1.86E+01
ADAMTS5	4.37E+01	2.52E-08	8.91E-08	9.40E+00	3.23E+00	9.40E+00	3.98E+00	4.23E-01
PAQR8	3.29E+02	2.54E-08	8.95E-08	1.75E+00	8.08E-01	1.75E+00	2.17E+01	1.24E+01
LOC101907642	1.12E+02	2.56E-08	9.02E-08	5.25E+00	2.39E+00	5.25E+00	9.52E+00	1.81E+00
SAMD5	1.99E+01	2.59E-08	9.11E-08	7.45E+00	2.90E+00	7.45E+00	1.78E+00	2.38E-01
VPS4B	7.46E+02	2.62E-08	9.17E-08	1.49E+00	5.72E-01	1.49E+00	4.62E+01	3.11E+01
SMTN	2.62E+03	2.63E-08	9.19E-08	1.68E+00	7.49E-01	1.68E+00	1.70E+02	1.01E+02
TEK	6.93E+02	2.67E-08	9.33E-08	1.94E+00	9.59E-01	1.94E+00	4.74E+01	2.44E+01
CDH11	4.22E+03	2.74E-08	9.54E-08	2.80E+00	1.48E+00	2.80E+00	3.18E+02	1.14E+02
RAB27B	3.03E+01	2.77E-08	9.62E-08	3.44E+00	1.78E+00	3.44E+00	2.40E+00	6.98E-01
CAV2	1.30E+02	2.77E-08	9.62E-08	2.60E+00	1.38E+00	2.60E+00	9.64E+00	3.71E+00
FHDC1	2.25E+02	2.80E-08	9.69E-08	2.96E+00	1.56E+00	2.96E+00	1.71E+01	5.79E+00
MIER1	7.91E+02	2.82E-08	9.75E-08	1.90E+00	9.22E-01	1.90E+00	5.34E+01	2.82E+01
RPS6KA5	5.96E+02	2.92E-08	1.01E-07	1.73E+00	7.93E-01	1.73E+00	3.90E+01	2.25E+01
XR_810543.3	3.60E+01	2.93E-08	1.01E-07	4.09E+00	2.03E+00	4.09E+00	2.93E+00	7.18E-01
XR_003030695.1	3.06E+01	2.94E-08	1.01E-07	5.01E+00	2.33E+00	5.01E+00	2.59E+00	5.17E-01
ERCC5	1.18E+03	2.98E-08	1.02E-07	1.58E+00	6.56E-01	1.58E+00	7.46E+01	4.74E+01
MAP3K7CL	7.62E+01	3.05E-08	1.05E-07	7.60E+00	2.93E+00	7.60E+00	6.77E+00	8.92E-01
WDFY4	2.72E+02	3.07E-08	1.05E-07	3.95E+00	1.98E+00	3.95E+00	2.20E+01	5.56E+00
AFF2	8.21E+02	3.15E-08	1.07E-07	3.34E+00	1.74E+00	3.34E+00	6.44E+01	1.93E+01
DSEL	1.02E+03	3.16E-08	1.07E-07	2.07E+00	1.05E+00	2.07E+00	7.07E+01	3.41E+01
RAB23	5.78E+02	3.19E-08	1.08E-07	1.63E+00	7.06E-01	1.63E+00	3.72E+01	2.28E+01

RERG	2.41E+02	3.21E-08	1.09E-07	3.01E+00	1.59E+00	3.01E+00	1.85E+01	6.13E+00
MAP1B	1.65E+03	3.24E-08	1.10E-07	1.86E+00	8.96E-01	1.86E+00	1.11E+02	5.97E+01
CSGALNACT1	1.12E+03	3.25E-08	1.10E-07	2.44E+00	1.29E+00	2.44E+00	8.17E+01	3.35E+01
XR_003029640.1	3.29E+02	3.25E-08	1.10E-07	2.38E+00	1.25E+00	2.38E+00	2.38E+01	1.00E+01
PDGFRA	7.16E+03	3.26E-08	1.10E-07	3.19E+00	1.67E+00	3.19E+00	5.57E+02	1.75E+02
GOLM1	3.46E+03	3.29E-08	1.10E-07	1.61E+00	6.88E-01	1.61E+00	2.20E+02	1.37E+02
XR_003038029.1	5.38E+02	3.30E-08	1.11E-07	2.36E+00	1.24E+00	2.36E+00	3.88E+01	1.65E+01
GPR17	3.49E+01	3.32E-08	1.11E-07	4.00E+00	2.00E+00	4.00E+00	2.82E+00	7.04E-01
C5AR2	2.41E+01	3.37E-08	1.12E-07	9.70E+00	3.28E+00	9.70E+00	2.19E+00	2.26E-01
MFAP3	5.37E+02	3.51E-08	1.17E-07	1.83E+00	8.71E-01	1.83E+00	3.60E+01	1.97E+01
LOC101902991	4.13E+01	3.51E-08	1.17E-07	3.10E+00	1.63E+00	3.10E+00	3.19E+00	1.03E+00
COPA	5.27E+03	3.58E-08	1.19E-07	1.56E+00	6.42E-01	1.56E+00	3.32E+02	2.13E+02
ERBIN	1.34E+03	3.60E-08	1.19E-07	2.02E+00	1.01E+00	2.02E+00	9.25E+01	4.59E+01
ADAP2	5.40E+01	3.64E-08	1.21E-07	5.53E+00	2.47E+00	5.53E+00	4.63E+00	8.37E-01
MFAP4	3.95E+03	3.77E-08	1.25E-07	2.85E+00	1.51E+00	2.85E+00	2.98E+02	1.05E+02
TYK2	1.70E+03	3.80E-08	1.25E-07	1.80E+00	8.46E-01	1.80E+00	1.13E+02	6.29E+01
ZNF185	2.28E+02	3.83E-08	1.26E-07	3.88E+00	1.96E+00	3.88E+00	1.84E+01	4.74E+00
CAPN2	3.07E+03	3.85E-08	1.26E-07	1.66E+00	7.32E-01	1.66E+00	1.99E+02	1.20E+02
GNE	9.20E+02	3.85E-08	1.26E-07	1.82E+00	8.66E-01	1.82E+00	6.14E+01	3.37E+01
TP53BP2	1.97E+03	3.85E-08	1.26E-07	1.58E+00	6.59E-01	1.58E+00	1.26E+02	7.96E+01
XR_003032575.1	1.05E+02	3.90E-08	1.27E-07	3.83E+00	1.94E+00	3.83E+00	8.45E+00	2.21E+00
ITGA8	9.90E+02	3.94E-08	1.28E-07	3.54E+00	1.82E+00	3.54E+00	7.90E+01	2.23E+01
ANK3	5.06E+02	3.97E-08	1.29E-07	2.62E+00	1.39E+00	2.62E+00	3.77E+01	1.44E+01
NGF	2.69E+02	3.97E-08	1.29E-07	3.10E+00	1.63E+00	3.10E+00	2.06E+01	6.64E+00
TANC2	1.32E+03	4.04E-08	1.31E-07	2.68E+00	1.42E+00	2.68E+00	9.81E+01	3.66E+01
IGF2R	8.61E+03	4.21E-08	1.36E-07	1.72E+00	7.86E-01	1.72E+00	5.63E+02	3.26E+02
FRMD3	1.22E+02	4.22E-08	1.36E-07	3.57E+00	1.84E+00	3.57E+00	9.70E+00	2.72E+00
FOCAD	4.58E+02	4.32E-08	1.39E-07	1.97E+00	9.82E-01	1.97E+00	3.13E+01	1.59E+01
XR_001495001.2	2.81E+01	4.33E-08	1.40E-07	8.85E+00	3.15E+00	8.85E+00	2.54E+00	2.87E-01
PRKAR2B	1.19E+03	4.41E-08	1.42E-07	3.05E+00	1.61E+00	3.05E+00	9.19E+01	3.01E+01
TMX3	8.09E+02	4.42E-08	1.42E-07	1.70E+00	7.65E-01	1.70E+00	5.28E+01	3.11E+01
CRISPLD2	1.46E+03	4.43E-08	1.42E-07	3.49E+00	1.80E+00	3.49E+00	1.15E+02	3.30E+01
GXYLT2	6.55E+02	4.44E-08	1.42E-07	3.79E+00	1.92E+00	3.79E+00	5.26E+01	1.39E+01

XR_815453.3	4.07E+02	4.44E-08	1.42E-07	1.78E+00	8.33E-01	1.78E+00	2.69E+01	1.51E+01
PDGFRL	7.98E+01	4.44E-08	1.42E-07	3.93E+00	1.97E+00	3.93E+00	6.47E+00	1.65E+00
CBLB	3.85E+02	4.47E-08	1.42E-07	1.71E+00	7.74E-01	1.71E+00	2.52E+01	1.47E+01
PPP1R12A	1.08E+03	4.47E-08	1.42E-07	2.02E+00	1.01E+00	2.02E+00	7.46E+01	3.70E+01
ADAMTS14	1.96E+02	4.58E-08	1.45E-07	2.76E+00	1.46E+00	2.76E+00	1.48E+01	5.36E+00
ZBTB25	8.83E+01	4.71E-08	1.49E-07	2.47E+00	1.30E+00	2.47E+00	6.41E+00	2.60E+00
ELK3	1.44E+03	4.72E-08	1.49E-07	1.89E+00	9.19E-01	1.89E+00	9.76E+01	5.16E+01
GPD2	4.47E+02	4.79E-08	1.51E-07	3.39E+00	1.76E+00	3.39E+00	3.52E+01	1.04E+01
LOC515240	1.62E+01	4.81E-08	1.51E-07	4.64E+00	2.21E+00	4.64E+00	1.35E+00	2.91E-01
DDX21	6.32E+02	4.84E-08	1.52E-07	2.43E+00	1.28E+00	2.43E+00	4.60E+01	1.89E+01
FSTL1	8.07E+03	4.86E-08	1.52E-07	1.93E+00	9.48E-01	1.93E+00	5.48E+02	2.84E+02
XR_003031732.1	4.63E+02	4.86E-08	1.52E-07	2.32E+00	1.21E+00	2.32E+00	3.33E+01	1.43E+01
PGM2	1.30E+02	4.92E-08	1.54E-07	2.47E+01	4.63E+00	2.47E+01	1.25E+01	5.06E-01
PER2	2.81E+02	4.96E-08	1.55E-07	2.66E+00	1.41E+00	2.66E+00	2.09E+01	7.84E+00
AMER1	3.90E+02	4.99E-08	1.55E-07	1.65E+00	7.25E-01	1.65E+00	2.52E+01	1.52E+01
SREK1	1.66E+03	5.06E-08	1.57E-07	1.87E+00	9.05E-01	1.87E+00	1.12E+02	5.99E+01
DAB2	1.97E+03	5.08E-08	1.58E-07	1.91E+00	9.30E-01	1.91E+00	1.34E+02	7.01E+01
RHOBTB3	1.59E+03	5.15E-08	1.60E-07	2.29E+00	1.19E+00	2.29E+00	1.14E+02	5.00E+01
EIF2A	1.48E+03	5.24E-08	1.62E-07	1.44E+00	5.22E-01	1.44E+00	9.07E+01	6.32E+01
ATG16L2	7.78E+02	5.50E-08	1.70E-07	2.19E+00	1.13E+00	2.19E+00	5.46E+01	2.49E+01
HIVEP2	2.54E+02	5.51E-08	1.70E-07	2.88E+00	1.53E+00	2.88E+00	1.93E+01	6.71E+00
LRRC32	7.28E+02	5.59E-08	1.72E-07	2.52E+00	1.33E+00	2.52E+00	5.34E+01	2.12E+01
KLHL7	2.87E+02	5.60E-08	1.72E-07	1.70E+00	7.63E-01	1.70E+00	1.86E+01	1.10E+01
XR_003030731.1	1.60E+02	5.64E-08	1.73E-07	2.11E+00	1.08E+00	2.11E+00	1.10E+01	5.20E+00
CACNA2D1	3.32E+02	5.78E-08	1.77E-07	2.45E+00	1.29E+00	2.45E+00	2.43E+01	9.92E+00
GSTM2	1.50E+03	5.88E-08	1.80E-07	2.49E+00	1.32E+00	2.49E+00	1.10E+02	4.41E+01
DTNA	3.68E+02	5.89E-08	1.80E-07	2.20E+00	1.14E+00	2.20E+00	2.61E+01	1.18E+01
RCOR3	6.40E+02	5.92E-08	1.80E-07	1.78E+00	8.34E-01	1.78E+00	4.22E+01	2.37E+01
ITGA7	6.05E+02	5.97E-08	1.81E-07	2.63E+00	1.39E+00	2.63E+00	4.47E+01	1.70E+01
PI4K2B	2.35E+02	6.01E-08	1.82E-07	1.87E+00	9.02E-01	1.87E+00	1.58E+01	8.44E+00
CTDSPL	1.33E+03	6.20E-08	1.88E-07	1.60E+00	6.74E-01	1.60E+00	8.43E+01	5.28E+01
CH25H	2.59E+02	6.38E-08	1.93E-07	9.34E+00	3.22E+00	9.34E+00	2.35E+01	2.52E+00
STX6	1.03E+03	6.45E-08	1.95E-07	1.66E+00	7.30E-01	1.66E+00	6.65E+01	4.01E+01

FOXN2	1.79E+02	6.46E-08	1.95E-07	1.89E+00	9.20E-01	1.89E+00	1.20E+01	6.36E+00
ABCC9	2.67E+02	6.49E-08	1.95E-07	3.21E+00	1.68E+00	3.21E+00	2.08E+01	6.47E+00
ADAMTS3	4.31E+02	6.64E-08	1.99E-07	2.55E+00	1.35E+00	2.55E+00	3.18E+01	1.25E+01
LGI4	3.22E+02	6.68E-08	2.00E-07	2.89E+00	1.53E+00	2.89E+00	2.43E+01	8.41E+00
CCDC6	1.27E+03	6.71E-08	2.01E-07	1.50E+00	5.83E-01	1.50E+00	7.93E+01	5.30E+01
TMEM65	3.83E+02	6.81E-08	2.03E-07	1.79E+00	8.41E-01	1.79E+00	2.53E+01	1.41E+01
SEMA5A	3.18E+03	6.87E-08	2.05E-07	2.48E+00	1.31E+00	2.48E+00	2.33E+02	9.37E+01
FAM76A	1.34E+02	7.05E-08	2.10E-07	3.01E+00	1.59E+00	3.01E+00	1.02E+01	3.40E+00
RHOJ	3.96E+02	7.06E-08	2.10E-07	1.94E+00	9.56E-01	1.94E+00	2.68E+01	1.38E+01
CPE	2.54E+03	7.08E-08	2.10E-07	1.83E+00	8.75E-01	1.83E+00	1.69E+02	9.22E+01
AKAP13	3.21E+03	7.09E-08	2.10E-07	1.80E+00	8.45E-01	1.80E+00	2.13E+02	1.19E+02
PRKAA1	3.76E+02	7.12E-08	2.11E-07	1.90E+00	9.23E-01	1.90E+00	2.55E+01	1.34E+01
MN1	6.89E+02	7.24E-08	2.14E-07	3.02E+00	1.59E+00	3.02E+00	5.27E+01	1.75E+01
XR_003029468.1	2.41E+02	7.27E-08	2.14E-07	5.15E+00	2.37E+00	5.15E+00	2.04E+01	3.97E+00
CCDC93	3.67E+02	7.33E-08	2.16E-07	2.00E+00	1.00E+00	2.00E+00	2.51E+01	1.26E+01
XR_815171.3	1.99E+01	7.36E-08	2.16E-07	5.39E+00	2.43E+00	5.39E+00	1.69E+00	3.13E-01
CCNG1	4.04E+03	7.39E-08	2.17E-07	1.50E+00	5.89E-01	1.50E+00	2.52E+02	1.67E+02
USP33	6.06E+02	7.44E-08	2.18E-07	1.77E+00	8.26E-01	1.77E+00	4.01E+01	2.26E+01
LTBP1	1.34E+04	7.60E-08	2.22E-07	3.09E+00	1.63E+00	3.09E+00	1.03E+03	3.35E+02
CYYR1	6.72E+02	7.77E-08	2.27E-07	2.26E+00	1.17E+00	2.26E+00	4.80E+01	2.13E+01
IPO7	1.06E+03	7.79E-08	2.27E-07	2.00E+00	1.00E+00	2.00E+00	7.32E+01	3.65E+01
ADAM9	4.29E+02	7.80E-08	2.27E-07	2.44E+00	1.29E+00	2.44E+00	3.13E+01	1.28E+01
MAP4K5	6.94E+02	7.85E-08	2.28E-07	1.75E+00	8.04E-01	1.75E+00	4.57E+01	2.61E+01
AEBP1	4.42E+03	8.08E-08	2.34E-07	1.95E+00	9.65E-01	1.95E+00	3.01E+02	1.54E+02
CDH19	8.18E+00	8.09E-08	2.34E-07	4.74E+01	5.57E+00	4.74E+01	8.01E-01	1.69E-02
KANSL1L	4.91E+02	8.09E-08	2.34E-07	1.89E+00	9.15E-01	1.89E+00	3.32E+01	1.76E+01
FAM171B	7.23E+02	8.10E-08	2.34E-07	2.93E+00	1.55E+00	2.93E+00	5.51E+01	1.88E+01
HEPH	3.92E+02	8.19E-08	2.36E-07	2.98E+00	1.58E+00	2.98E+00	3.01E+01	1.01E+01
XR_003029589.1	3.04E+01	8.20E-08	2.36E-07	2.82E+00	1.50E+00	2.82E+00	2.29E+00	8.11E-01
CTNNB1	4.06E+03	8.32E-08	2.39E-07	1.46E+00	5.49E-01	1.46E+00	2.50E+02	1.71E+02
SLC26A10	3.32E+02	8.35E-08	2.40E-07	2.45E+00	1.29E+00	2.45E+00	2.42E+01	9.88E+00
TOGARAM1	3.95E+02	8.46E-08	2.42E-07	2.09E+00	1.06E+00	2.09E+00	2.75E+01	1.32E+01
TRAK1	1.13E+03	8.46E-08	2.42E-07	1.58E+00	6.63E-01	1.58E+00	7.18E+01	4.54E+01

NOTCH4	3.20E+02	8.49E-08	2.42E-07	2.03E+00	1.02E+00	2.03E+00	2.21E+01	1.09E+01
RGS6	5.78E+02	8.52E-08	2.43E-07	3.11E+00	1.64E+00	3.11E+00	4.46E+01	1.43E+01
ATP10D	5.38E+02	8.59E-08	2.44E-07	2.21E+00	1.14E+00	2.21E+00	3.82E+01	1.73E+01
KIAA0355	2.29E+03	8.63E-08	2.45E-07	1.68E+00	7.50E-01	1.68E+00	1.48E+02	8.79E+01
NR4A1	3.44E+03	8.66E-08	2.46E-07	1.02E+01	3.35E+00	1.02E+01	3.16E+02	3.09E+01
SLC46A2	5.58E+01	8.71E-08	2.47E-07	3.23E+00	1.69E+00	3.23E+00	4.34E+00	1.35E+00
LOC107131398	1.61E+02	8.81E-08	2.49E-07	3.26E+00	1.71E+00	3.26E+00	1.24E+01	3.81E+00
ARMC10	1.69E+03	8.90E-08	2.51E-07	1.35E+00	4.36E-01	1.35E+00	1.01E+02	7.49E+01
LOC101902475	1.38E+02	8.95E-08	2.52E-07	2.72E+00	1.44E+00	2.72E+00	1.03E+01	3.80E+00
PABPC5	8.95E+01	9.03E-08	2.54E-07	2.80E+00	1.48E+00	2.80E+00	6.76E+00	2.42E+00
XR_239392.4	5.93E+02	9.11E-08	2.56E-07	1.65E+00	7.20E-01	1.65E+00	3.83E+01	2.33E+01
CASP8	1.75E+02	9.22E-08	2.59E-07	2.31E+00	1.21E+00	2.31E+00	1.25E+01	5.42E+00
PLA2G12A	5.82E+02	9.32E-08	2.61E-07	1.41E+00	4.93E-01	1.41E+00	3.56E+01	2.53E+01
LRIG3	3.33E+02	9.40E-08	2.63E-07	2.41E+00	1.27E+00	2.41E+00	2.41E+01	9.96E+00
F8	1.71E+02	9.41E-08	2.63E-07	1.86E+00	8.94E-01	1.86E+00	1.15E+01	6.17E+00
FXYD5	3.53E+02	9.55E-08	2.66E-07	2.24E+00	1.17E+00	2.24E+00	2.50E+01	1.12E+01
MKL2	1.26E+03	9.56E-08	2.66E-07	1.96E+00	9.70E-01	1.96E+00	8.67E+01	4.43E+01
TOR1AIP2	1.03E+03	9.69E-08	2.69E-07	1.47E+00	5.51E-01	1.47E+00	6.35E+01	4.33E+01
PHKB	4.51E+02	9.82E-08	2.72E-07	1.65E+00	7.24E-01	1.65E+00	2.91E+01	1.76E+01
GLG1	8.57E+03	1.01E-07	2.80E-07	1.70E+00	7.62E-01	1.70E+00	5.57E+02	3.29E+02
JAML	3.77E+01	1.02E-07	2.81E-07	4.05E+00	2.02E+00	4.05E+00	3.06E+00	7.55E-01
GPC6	6.25E+01	1.03E-07	2.83E-07	9.94E+00	3.31E+00	9.94E+00	5.74E+00	5.77E-01
FRMD4B	5.20E+02	1.03E-07	2.84E-07	1.89E+00	9.15E-01	1.89E+00	3.51E+01	1.86E+01
DYSF	1.32E+03	1.05E-07	2.89E-07	2.50E+00	1.32E+00	2.50E+00	9.69E+01	3.87E+01
MMRN2	3.12E+02	1.06E-07	2.90E-07	3.23E+00	1.69E+00	3.23E+00	2.43E+01	7.52E+00
DRAM2	3.64E+02	1.06E-07	2.92E-07	1.77E+00	8.20E-01	1.77E+00	2.41E+01	1.36E+01
FAM13C	5.03E+02	1.07E-07	2.94E-07	1.86E+00	8.97E-01	1.86E+00	3.37E+01	1.81E+01
SGCB	1.09E+03	1.09E-07	2.99E-07	1.77E+00	8.20E-01	1.77E+00	7.18E+01	4.07E+01
PAPD5	3.15E+02	1.09E-07	2.99E-07	1.90E+00	9.29E-01	1.90E+00	2.13E+01	1.12E+01
RBPJ	1.86E+03	1.12E-07	3.06E-07	2.06E+00	1.04E+00	2.06E+00	1.29E+02	6.27E+01
ACVR1	7.58E+02	1.13E-07	3.08E-07	1.49E+00	5.77E-01	1.49E+00	4.73E+01	3.17E+01
MS4A2	4.97E+00	1.15E-07	3.13E-07	4.97E+01	5.64E+00	4.97E+01	4.89E-01	9.83E-03
SERPINB9	1.65E+02	1.20E-07	3.25E-07	2.33E+00	1.22E+00	2.33E+00	1.18E+01	5.05E+00

BNIP1	1.43E+02	1.21E-07	3.29E-07	1.73E+00	7.89E-01	1.73E+00	9.31E+00	5.39E+00
RNASE4	5.04E+02	1.24E-07	3.35E-07	3.20E+00	1.68E+00	3.20E+00	3.91E+01	1.22E+01
ACOX1	1.23E+03	1.24E-07	3.36E-07	1.63E+00	7.08E-01	1.63E+00	7.90E+01	4.83E+01
XR_003033573.1	6.98E+02	1.25E-07	3.37E-07	1.54E+00	6.23E-01	1.54E+00	4.38E+01	2.85E+01
ALPK1	2.31E+02	1.25E-07	3.38E-07	2.50E+00	1.32E+00	2.50E+00	1.69E+01	6.77E+00
RIN2	1.94E+03	1.27E-07	3.42E-07	1.99E+00	9.96E-01	1.99E+00	1.33E+02	6.69E+01
RNASE10	1.60E+02	1.27E-07	3.42E-07	2.48E+00	1.31E+00	2.48E+00	1.18E+01	4.75E+00
DAPK1	1.07E+03	1.29E-07	3.46E-07	2.33E+00	1.22E+00	2.33E+00	7.69E+01	3.30E+01
LBR	5.81E+02	1.30E-07	3.49E-07	1.59E+00	6.70E-01	1.59E+00	3.70E+01	2.33E+01
DCTN4	1.90E+03	1.32E-07	3.53E-07	1.54E+00	6.25E-01	1.54E+00	1.20E+02	7.77E+01
FAM168A	1.99E+03	1.33E-07	3.56E-07	1.66E+00	7.28E-01	1.66E+00	1.28E+02	7.75E+01
XR_003031013.1	2.61E+02	1.34E-07	3.56E-07	2.13E+00	1.09E+00	2.13E+00	1.82E+01	8.50E+00
PPFIBP1	1.18E+03	1.35E-07	3.59E-07	2.12E+00	1.08E+00	2.12E+00	8.30E+01	3.92E+01
TGFB1I1	1.59E+03	1.35E-07	3.59E-07	2.12E+00	1.08E+00	2.12E+00	1.11E+02	5.26E+01
XR_003032607.1	2.18E+01	1.36E-07	3.62E-07	4.17E+00	2.06E+00	4.17E+00	1.78E+00	4.27E-01
GAS6	2.35E+03	1.37E-07	3.63E-07	2.62E+00	1.39E+00	2.62E+00	1.75E+02	6.68E+01
NRIP1	1.13E+03	1.41E-07	3.73E-07	2.37E+00	1.25E+00	2.37E+00	8.16E+01	3.44E+01
XR_003035005.1	1.79E+01	1.44E-07	3.80E-07	1.00E+01	3.32E+00	1.00E+01	1.64E+00	1.64E-01
S100A4	5.33E+01	1.45E-07	3.83E-07	4.37E+00	2.13E+00	4.37E+00	4.41E+00	1.01E+00
ACBD3	1.19E+03	1.50E-07	3.96E-07	1.59E+00	6.65E-01	1.59E+00	7.55E+01	4.76E+01
TMC7	1.98E+01	1.52E-07	4.00E-07	3.25E+00	1.70E+00	3.25E+00	1.54E+00	4.73E-01
COL11A1	9.08E+02	1.53E-07	4.01E-07	2.63E+00	1.39E+00	2.63E+00	6.75E+01	2.57E+01
BCL6B	4.86E+02	1.54E-07	4.04E-07	1.71E+00	7.71E-01	1.71E+00	3.17E+01	1.86E+01
PDE4A	1.02E+03	1.54E-07	4.04E-07	1.90E+00	9.27E-01	1.90E+00	6.80E+01	3.58E+01
GPCPD1	1.67E+03	1.54E-07	4.04E-07	1.74E+00	7.99E-01	1.74E+00	1.10E+02	6.31E+01
ELP6	2.05E+02	1.56E-07	4.06E-07	2.34E+00	1.23E+00	2.34E+00	1.47E+01	6.30E+00
PGR	3.03E+02	1.56E-07	4.07E-07	2.84E+00	1.51E+00	2.84E+00	2.27E+01	8.00E+00
PLEKHH2	6.72E+02	1.56E-07	4.07E-07	2.44E+00	1.29E+00	2.44E+00	4.90E+01	2.01E+01
RNF168	4.97E+02	1.58E-07	4.12E-07	1.70E+00	7.63E-01	1.70E+00	3.24E+01	1.91E+01
PLOD2	1.01E+03	1.59E-07	4.14E-07	1.90E+00	9.28E-01	1.90E+00	6.85E+01	3.60E+01
LOC789091	2.02E+01	1.60E-07	4.16E-07	3.13E+00	1.65E+00	3.13E+00	1.56E+00	4.99E-01
TMPO	2.07E+03	1.61E-07	4.18E-07	1.50E+00	5.83E-01	1.50E+00	1.29E+02	8.62E+01
LMNA	4.01E+03	1.62E-07	4.19E-07	1.87E+00	9.04E-01	1.87E+00	2.70E+02	1.44E+02

DIXDC1	4.12E+02	1.63E-07	4.20E-07	1.95E+00	9.61E-01	1.95E+00	2.81E+01	1.44E+01
HSPG2	1.97E+04	1.63E-07	4.20E-07	1.83E+00	8.74E-01	1.83E+00	1.33E+03	7.23E+02
DOCK7	7.76E+02	1.64E-07	4.23E-07	1.67E+00	7.38E-01	1.67E+00	5.05E+01	3.03E+01
XR_809167.3	2.83E+02	1.66E-07	4.26E-07	2.63E+00	1.40E+00	2.63E+00	2.09E+01	7.94E+00
CCR8	4.80E+01	1.71E-07	4.38E-07	3.37E+00	1.75E+00	3.37E+00	3.75E+00	1.12E+00
EPC2	6.78E+02	1.71E-07	4.39E-07	1.74E+00	7.97E-01	1.74E+00	4.44E+01	2.56E+01
TCF21	3.02E+03	1.72E-07	4.40E-07	2.92E+00	1.55E+00	2.92E+00	2.29E+02	7.84E+01
LIG4	2.11E+02	1.73E-07	4.43E-07	2.02E+00	1.01E+00	2.02E+00	1.45E+01	7.19E+00
MED23	5.01E+02	1.75E-07	4.47E-07	1.94E+00	9.58E-01	1.94E+00	3.42E+01	1.76E+01
BMPR1A	1.04E+03	1.78E-07	4.53E-07	2.32E+00	1.21E+00	2.32E+00	7.45E+01	3.21E+01
LOC112448856	2.03E+01	1.82E-07	4.63E-07	8.01E+00	3.00E+00	8.01E+00	1.82E+00	2.27E-01
SETBP1	4.73E+02	1.84E-07	4.68E-07	3.20E+00	1.68E+00	3.20E+00	3.68E+01	1.15E+01
ZNF606	3.80E+02	1.87E-07	4.75E-07	2.46E+00	1.30E+00	2.46E+00	2.78E+01	1.13E+01
KCNMB1	4.13E+01	1.88E-07	4.76E-07	1.21E+01	3.59E+00	1.21E+01	3.84E+00	3.18E-01
TIPRL	5.31E+02	1.88E-07	4.76E-07	1.46E+00	5.49E-01	1.46E+00	3.27E+01	2.23E+01
LITAF	1.30E+03	1.92E-07	4.85E-07	1.77E+00	8.26E-01	1.77E+00	8.60E+01	4.85E+01
IPO8	7.40E+02	1.94E-07	4.89E-07	1.62E+00	6.99E-01	1.62E+00	4.76E+01	2.93E+01
EPC1	1.50E+03	1.94E-07	4.89E-07	1.44E+00	5.21E-01	1.44E+00	9.20E+01	6.41E+01
RCAN2	2.50E+02	2.01E-07	5.05E-07	3.52E+00	1.81E+00	3.52E+00	1.98E+01	5.63E+00
LIN7C	6.32E+02	2.02E-07	5.06E-07	1.75E+00	8.06E-01	1.75E+00	4.17E+01	2.38E+01
ANKRD13C	1.03E+02	2.04E-07	5.11E-07	1.99E+00	9.92E-01	1.99E+00	7.06E+00	3.55E+00
ADCY3	9.95E+02	2.04E-07	5.11E-07	2.02E+00	1.02E+00	2.02E+00	6.83E+01	3.37E+01
ZNF112	2.03E+02	2.07E-07	5.17E-07	2.12E+00	1.08E+00	2.12E+00	1.43E+01	6.73E+00
GLCE	9.28E+02	2.07E-07	5.17E-07	1.97E+00	9.77E-01	1.97E+00	6.35E+01	3.23E+01
RTL9	4.67E+01	2.09E-07	5.20E-07	2.43E+00	1.28E+00	2.43E+00	3.41E+00	1.40E+00
PDK1	1.03E+03	2.10E-07	5.24E-07	1.75E+00	8.05E-01	1.75E+00	6.78E+01	3.88E+01
XR_003032993.1	3.88E+01	2.13E-07	5.31E-07	3.01E+00	1.59E+00	3.01E+00	2.97E+00	9.84E-01
TRIM44	8.30E+02	2.14E-07	5.31E-07	1.72E+00	7.85E-01	1.72E+00	5.43E+01	3.15E+01
LOC101903572	1.07E+02	2.22E-07	5.51E-07	1.81E+01	4.18E+00	1.81E+01	1.02E+01	5.64E-01
SENP7	2.58E+02	2.25E-07	5.56E-07	2.13E+00	1.09E+00	2.13E+00	1.81E+01	8.51E+00
ANGPTL2	2.96E+03	2.25E-07	5.57E-07	2.26E+00	1.17E+00	2.26E+00	2.11E+02	9.35E+01
MMP23	1.47E+03	2.33E-07	5.76E-07	1.98E+00	9.89E-01	1.98E+00	1.01E+02	5.09E+01
ZNF383	1.08E+02	2.35E-07	5.81E-07	1.76E+00	8.20E-01	1.76E+00	7.11E+00	4.03E+00

FAM162B	2.67E+01	2.36E-07	5.82E-07	3.60E+00	1.85E+00	3.60E+00	2.11E+00	5.86E-01
CDH5	2.30E+03	2.38E-07	5.85E-07	1.65E+00	7.26E-01	1.65E+00	1.48E+02	8.95E+01
CDKN1B	9.59E+02	2.44E-07	5.99E-07	1.49E+00	5.79E-01	1.49E+00	5.95E+01	3.99E+01
PDE5A	4.76E+02	2.45E-07	6.01E-07	2.45E+00	1.30E+00	2.45E+00	3.46E+01	1.41E+01
KR_001494670.2	4.03E+01	2.46E-07	6.03E-07	2.88E+00	1.53E+00	2.88E+00	3.07E+00	1.06E+00
LRRFIP1	7.85E+02	2.46E-07	6.03E-07	1.98E+00	9.84E-01	1.98E+00	5.39E+01	2.72E+01
MTM1	1.23E+02	2.49E-07	6.08E-07	1.86E+00	8.92E-01	1.86E+00	8.17E+00	4.41E+00
TLR3	4.49E+02	2.50E-07	6.09E-07	2.11E+00	1.08E+00	2.11E+00	3.12E+01	1.48E+01
PLXNA4	1.11E+03	2.51E-07	6.11E-07	3.76E+00	1.91E+00	3.76E+00	8.96E+01	2.38E+01
CCR2	2.04E+01	2.51E-07	6.11E-07	6.01E+00	2.59E+00	6.01E+00	1.76E+00	2.92E-01
ERLIN1	9.15E+02	2.52E-07	6.12E-07	1.40E+00	4.83E-01	1.40E+00	5.54E+01	3.96E+01
MIGA1	1.65E+02	2.52E-07	6.12E-07	2.00E+00	1.00E+00	2.00E+00	1.14E+01	5.67E+00
OGA	1.74E+03	2.53E-07	6.14E-07	1.66E+00	7.28E-01	1.66E+00	1.13E+02	6.79E+01
MBLAC2	1.81E+02	2.55E-07	6.18E-07	1.70E+00	7.62E-01	1.70E+00	1.18E+01	6.96E+00
KR_003032359.1	1.10E+02	2.58E-07	6.24E-07	3.75E+00	1.91E+00	3.75E+00	8.74E+00	2.33E+00
KR_003038036.1	1.01E+02	2.58E-07	6.24E-07	3.40E+00	1.77E+00	3.40E+00	7.90E+00	2.32E+00
KR_003033144.1	1.09E+01	2.62E-07	6.32E-07	9.17E+00	3.20E+00	9.17E+00	9.86E-01	1.07E-01
LOC101902435	8.47E+01	2.64E-07	6.36E-07	2.43E+00	1.28E+00	2.43E+00	6.11E+00	2.52E+00
PALMD	1.47E+02	2.66E-07	6.40E-07	2.99E+00	1.58E+00	2.99E+00	1.13E+01	3.76E+00
TMED10	5.79E+03	2.67E-07	6.42E-07	1.52E+00	6.05E-01	1.52E+00	3.63E+02	2.39E+02
SH3BGRL2	5.93E+02	2.70E-07	6.48E-07	2.17E+00	1.12E+00	2.17E+00	4.15E+01	1.91E+01
KR_003037059.1	2.59E+01	2.71E-07	6.49E-07	4.06E+00	2.02E+00	4.06E+00	2.10E+00	5.17E-01
GULP1	9.03E+01	2.71E-07	6.49E-07	3.01E+00	1.59E+00	3.01E+00	6.93E+00	2.30E+00
MYCBP2	1.48E+03	2.72E-07	6.49E-07	2.17E+00	1.12E+00	2.17E+00	1.04E+02	4.81E+01
ATXN1	1.69E+02	2.73E-07	6.52E-07	3.01E+00	1.59E+00	3.01E+00	1.29E+01	4.30E+00
TCAF1	4.29E+03	2.76E-07	6.56E-07	2.00E+00	1.00E+00	2.00E+00	2.96E+02	1.48E+02
ZZZ3	9.18E+02	2.76E-07	6.56E-07	1.68E+00	7.45E-01	1.68E+00	5.96E+01	3.55E+01
LURAP1	3.60E+02	2.79E-07	6.63E-07	2.56E+00	1.35E+00	2.56E+00	2.65E+01	1.04E+01
CLMN	7.00E+02	2.80E-07	6.63E-07	2.23E+00	1.16E+00	2.23E+00	4.97E+01	2.23E+01
STAM	5.25E+02	2.83E-07	6.69E-07	1.56E+00	6.40E-01	1.56E+00	3.32E+01	2.13E+01
PUM1	1.87E+03	2.87E-07	6.79E-07	1.87E+00	9.05E-01	1.87E+00	1.26E+02	6.73E+01
PRICKLE2	7.88E+02	2.88E-07	6.79E-07	2.22E+00	1.15E+00	2.22E+00	5.59E+01	2.51E+01
RNF217	2.62E+02	2.89E-07	6.83E-07	1.62E+00	6.99E-01	1.62E+00	1.68E+01	1.04E+01

ZNF557	3.99E+02	2.91E-07	6.85E-07	1.69E+00	7.53E-01	1.69E+00	2.59E+01	1.54E+01
CLEC2B	5.11E+01	2.97E-07	6.98E-07	5.01E+00	2.33E+00	5.01E+00	4.30E+00	8.57E-01
AIDA	2.65E+02	2.97E-07	6.98E-07	1.78E+00	8.32E-01	1.78E+00	1.75E+01	9.83E+00
USP28	1.13E+03	2.98E-07	6.98E-07	1.46E+00	5.43E-01	1.46E+00	6.98E+01	4.79E+01
WISP2	2.12E+01	2.98E-07	6.99E-07	1.47E+01	3.88E+00	1.47E+01	1.99E+00	1.35E-01
CDC40	3.61E+02	2.99E-07	6.99E-07	1.57E+00	6.55E-01	1.57E+00	2.29E+01	1.46E+01
SHPK	6.18E+01	3.00E-07	7.00E-07	2.19E+00	1.13E+00	2.19E+00	4.34E+00	1.98E+00
PBX1	3.56E+03	3.02E-07	7.04E-07	2.30E+00	1.20E+00	2.30E+00	2.55E+02	1.11E+02
CAMKK2	1.46E+03	3.06E-07	7.12E-07	1.75E+00	8.05E-01	1.75E+00	9.54E+01	5.46E+01
RPS6KC1	6.20E+02	3.14E-07	7.32E-07	1.60E+00	6.75E-01	1.60E+00	3.97E+01	2.49E+01
DAAM2	1.46E+03	3.17E-07	7.37E-07	1.87E+00	9.01E-01	1.87E+00	9.85E+01	5.27E+01
OXR1	6.89E+02	3.18E-07	7.39E-07	1.89E+00	9.17E-01	1.89E+00	4.62E+01	2.45E+01
BICC1	8.29E+02	3.19E-07	7.39E-07	2.62E+00	1.39E+00	2.62E+00	6.15E+01	2.35E+01
PWWP2A	7.33E+02	3.19E-07	7.39E-07	1.48E+00	5.67E-01	1.48E+00	4.54E+01	3.07E+01
CACNB2	3.08E+02	3.28E-07	7.60E-07	2.03E+00	1.02E+00	2.03E+00	2.12E+01	1.04E+01
LIMA1	1.06E+03	3.31E-07	7.65E-07	1.83E+00	8.68E-01	1.83E+00	7.09E+01	3.88E+01
ADAM10	9.14E+02	3.33E-07	7.69E-07	1.89E+00	9.19E-01	1.89E+00	6.20E+01	3.28E+01
KR_003031941.1	1.44E+01	3.34E-07	7.69E-07	4.76E+00	2.25E+00	4.76E+00	1.20E+00	2.53E-01
DHX36	5.28E+02	3.34E-07	7.69E-07	1.87E+00	9.05E-01	1.87E+00	3.56E+01	1.90E+01
THSD4	9.20E+01	3.36E-07	7.72E-07	3.36E+01	5.07E+00	3.36E+01	8.93E+00	2.66E-01
ZFP3	9.91E+01	3.38E-07	7.76E-07	2.83E+00	1.50E+00	2.83E+00	7.47E+00	2.63E+00
RCN1	7.07E+03	3.40E-07	7.79E-07	1.60E+00	6.80E-01	1.60E+00	4.52E+02	2.82E+02
KR_003030279.1	1.03E+01	3.41E-07	7.80E-07	1.84E+01	4.20E+00	1.84E+01	9.81E-01	5.34E-02
DBX1	4.47E+00	3.41E-07	7.80E-07	5.68E+00	2.51E+00	5.68E+00	3.84E-01	6.75E-02
MOG	2.17E+02	3.42E-07	7.81E-07	3.30E+00	1.72E+00	3.30E+00	1.70E+01	5.14E+00
KR_003031257.1	6.03E+01	3.43E-07	7.83E-07	9.91E+00	3.31E+00	9.91E+00	5.53E+00	5.58E-01
LOC781318	3.71E+01	3.46E-07	7.88E-07	2.40E+00	1.26E+00	2.40E+00	2.67E+00	1.11E+00
ZNF550	2.21E+02	3.47E-07	7.89E-07	2.44E+00	1.29E+00	2.44E+00	1.61E+01	6.60E+00
PAFAH1B2	7.93E+02	3.53E-07	8.01E-07	1.73E+00	7.92E-01	1.73E+00	5.19E+01	3.00E+01
VWF	2.17E+03	3.53E-07	8.01E-07	4.15E+00	2.05E+00	4.15E+00	1.78E+02	4.28E+01
KLHL5	7.64E+02	3.56E-07	8.06E-07	1.53E+00	6.11E-01	1.53E+00	4.79E+01	3.14E+01
TRIT1	3.53E+02	3.57E-07	8.06E-07	1.61E+00	6.85E-01	1.61E+00	2.25E+01	1.40E+01
APLP2	1.05E+04	3.57E-07	8.06E-07	1.47E+00	5.54E-01	1.47E+00	6.49E+02	4.42E+02

ENG	4.41E+03	3.59E-07	8.11E-07	1.86E+00	8.99E-01	1.86E+00	2.96E+02	1.59E+02
TSPOAP1	1.08E+03	3.63E-07	8.19E-07	1.66E+00	7.34E-01	1.66E+00	6.93E+01	4.17E+01
ZNF175	4.50E+02	3.66E-07	8.24E-07	1.82E+00	8.66E-01	1.82E+00	3.01E+01	1.65E+01
GALNT7	5.22E+02	3.67E-07	8.26E-07	1.80E+00	8.51E-01	1.80E+00	3.48E+01	1.93E+01
SLC36A4	4.93E+02	3.69E-07	8.27E-07	1.92E+00	9.41E-01	1.92E+00	3.33E+01	1.74E+01
LTBP3	6.20E+03	3.69E-07	8.27E-07	1.91E+00	9.36E-01	1.91E+00	4.17E+02	2.18E+02
LOC781280	2.05E+02	3.70E-07	8.29E-07	1.85E+00	8.88E-01	1.85E+00	1.37E+01	7.39E+00
PTGDR	4.82E+01	3.70E-07	8.29E-07	4.99E+00	2.32E+00	4.99E+00	4.05E+00	8.11E-01
IL17B	4.11E+01	3.72E-07	8.31E-07	4.84E+00	2.28E+00	4.84E+00	3.47E+00	7.18E-01
MARK1	1.36E+03	3.75E-07	8.36E-07	1.61E+00	6.86E-01	1.61E+00	8.68E+01	5.40E+01
ID4	3.34E+02	3.88E-07	8.64E-07	2.78E+00	1.48E+00	2.78E+00	2.53E+01	9.10E+00
AEBP2	5.73E+02	3.92E-07	8.72E-07	1.64E+00	7.10E-01	1.64E+00	3.67E+01	2.24E+01
HECA	6.89E+02	3.92E-07	8.72E-07	1.70E+00	7.66E-01	1.70E+00	4.47E+01	2.63E+01
HECTD4	2.47E+03	3.93E-07	8.73E-07	1.75E+00	8.07E-01	1.75E+00	1.61E+02	9.21E+01
GPATCH2L	1.41E+03	3.94E-07	8.73E-07	1.68E+00	7.48E-01	1.68E+00	9.14E+01	5.44E+01
TNFSF10	6.16E+01	4.00E-07	8.87E-07	3.01E+01	4.91E+00	3.01E+01	5.97E+00	1.98E-01
ATP2B4	2.68E+03	4.01E-07	8.88E-07	1.93E+00	9.49E-01	1.93E+00	1.83E+02	9.46E+01
VANGL1	4.93E+02	4.11E-07	9.09E-07	1.70E+00	7.68E-01	1.70E+00	3.22E+01	1.89E+01
TRPM6	3.36E+01	4.15E-07	9.16E-07	2.35E+00	1.23E+00	2.35E+00	2.41E+00	1.03E+00
XR_003029618.1	2.53E+01	4.21E-07	9.29E-07	8.72E+00	3.12E+00	8.72E+00	2.29E+00	2.63E-01
RUNDC3B	2.43E+02	4.24E-07	9.33E-07	2.80E+00	1.49E+00	2.80E+00	1.82E+01	6.49E+00
XR_003029474.1	3.77E+01	4.30E-07	9.45E-07	6.01E+00	2.59E+00	6.01E+00	3.26E+00	5.42E-01
C19H17orf100	1.04E+02	4.39E-07	9.64E-07	1.99E+00	9.96E-01	1.99E+00	7.09E+00	3.55E+00
Mar-07	7.18E+02	4.41E-07	9.66E-07	1.95E+00	9.62E-01	1.95E+00	4.92E+01	2.52E+01
DNMBP	1.17E+03	4.45E-07	9.76E-07	2.49E+00	1.31E+00	2.49E+00	8.54E+01	3.44E+01
SPRED2	1.50E+03	4.47E-07	9.79E-07	1.63E+00	7.05E-01	1.63E+00	9.65E+01	5.92E+01
LOC510362	3.56E+02	4.49E-07	9.81E-07	1.73E+00	7.89E-01	1.73E+00	2.33E+01	1.35E+01
ZC3HAV1	1.71E+03	4.49E-07	9.81E-07	1.53E+00	6.16E-01	1.53E+00	1.08E+02	7.07E+01
XR_003030148.1	1.30E+02	4.50E-07	9.82E-07	2.09E+00	1.06E+00	2.09E+00	8.97E+00	4.30E+00
ADCY4	3.19E+02	4.51E-07	9.82E-07	2.03E+00	1.02E+00	2.03E+00	2.20E+01	1.08E+01
GLIPR1	1.52E+02	4.58E-07	9.97E-07	5.89E+00	2.56E+00	5.89E+00	1.31E+01	2.23E+00
CHST1	2.13E+03	4.68E-07	1.02E-06	2.41E+00	1.27E+00	2.41E+00	1.54E+02	6.38E+01
PTPRK	6.53E+02	4.78E-07	1.04E-06	1.98E+00	9.82E-01	1.98E+00	4.49E+01	2.27E+01

XR_003033993.1	1.23E+01	4.88E-07	1.06E-06	3.36E+00	1.75E+00	3.36E+00	9.58E-01	2.85E-01
TMEM181	1.22E+03	5.00E-07	1.08E-06	1.47E+00	5.56E-01	1.47E+00	7.52E+01	5.11E+01
ZNF608	5.28E+02	5.02E-07	1.08E-06	1.96E+00	9.69E-01	1.96E+00	3.62E+01	1.85E+01
PTGFRN	4.98E+03	5.02E-07	1.08E-06	1.78E+00	8.32E-01	1.78E+00	3.31E+02	1.86E+02
GNAI1	4.98E+02	5.07E-07	1.09E-06	1.72E+00	7.86E-01	1.72E+00	3.27E+01	1.90E+01
RNF150	7.81E+02	5.11E-07	1.10E-06	1.94E+00	9.58E-01	1.94E+00	5.32E+01	2.74E+01
C1R	3.76E+03	5.16E-07	1.11E-06	2.37E+00	1.25E+00	2.37E+00	2.70E+02	1.14E+02
KCTD12	1.60E+03	5.16E-07	1.11E-06	2.71E+00	1.44E+00	2.71E+00	1.19E+02	4.40E+01
DFFB	3.97E+02	5.20E-07	1.12E-06	1.57E+00	6.51E-01	1.57E+00	2.51E+01	1.60E+01
COL6A2	2.55E+04	5.23E-07	1.12E-06	2.38E+00	1.25E+00	2.38E+00	1.84E+03	7.70E+02
INVS	6.61E+02	5.24E-07	1.12E-06	1.46E+00	5.42E-01	1.46E+00	4.07E+01	2.79E+01
LOC782545	1.50E+01	5.24E-07	1.12E-06	1.55E+01	3.95E+00	1.55E+01	1.42E+00	9.18E-02
LOC534742	5.01E+02	5.26E-07	1.12E-06	1.74E+00	7.96E-01	1.74E+00	3.30E+01	1.90E+01
PHF8	6.38E+03	5.34E-07	1.14E-06	1.50E+00	5.86E-01	1.50E+00	3.97E+02	2.64E+02
ZFP37	1.93E+02	5.36E-07	1.14E-06	1.97E+00	9.76E-01	1.97E+00	1.32E+01	6.71E+00
AAED1	5.68E+02	5.37E-07	1.14E-06	1.55E+00	6.35E-01	1.55E+00	3.59E+01	2.31E+01
PTEN	1.37E+03	5.39E-07	1.15E-06	1.46E+00	5.42E-01	1.46E+00	8.41E+01	5.78E+01
CCDC50	1.22E+03	5.41E-07	1.15E-06	1.47E+00	5.52E-01	1.47E+00	7.57E+01	5.16E+01
C21H14orf28	1.37E+02	5.65E-07	1.20E-06	1.67E+00	7.36E-01	1.67E+00	8.83E+00	5.30E+00
FAM241A	6.20E+01	5.73E-07	1.21E-06	2.27E+00	1.18E+00	2.27E+00	4.39E+00	1.93E+00
XR_003034780.1	2.20E+02	5.73E-07	1.21E-06	3.00E+00	1.59E+00	3.00E+00	1.68E+01	5.60E+00
PPM1D	4.93E+02	5.83E-07	1.23E-06	1.44E+00	5.22E-01	1.44E+00	3.02E+01	2.10E+01
HECW2	1.31E+02	5.83E-07	1.23E-06	2.15E+00	1.10E+00	2.15E+00	9.21E+00	4.28E+00
ENDOD1	2.43E+02	5.85E-07	1.23E-06	2.32E+00	1.21E+00	2.32E+00	1.74E+01	7.51E+00
LOC782470	4.98E+02	5.87E-07	1.24E-06	1.74E+00	8.01E-01	1.74E+00	3.27E+01	1.88E+01
PCDH7	7.47E+02	5.94E-07	1.25E-06	2.66E+00	1.41E+00	2.66E+00	5.60E+01	2.10E+01
CANX	7.05E+03	6.04E-07	1.27E-06	1.44E+00	5.27E-01	1.44E+00	4.32E+02	3.00E+02
KPNA1	6.70E+02	6.06E-07	1.27E-06	1.80E+00	8.46E-01	1.80E+00	4.44E+01	2.47E+01
DZIP1	2.26E+03	6.08E-07	1.27E-06	1.89E+00	9.15E-01	1.89E+00	1.53E+02	8.10E+01
P2RX7	5.38E+01	6.18E-07	1.29E-06	2.59E+00	1.37E+00	2.59E+00	3.96E+00	1.53E+00
THADA	4.69E+02	6.20E-07	1.30E-06	1.57E+00	6.55E-01	1.57E+00	2.99E+01	1.90E+01
CSNK1B	6.36E+02	6.26E-07	1.31E-06	1.71E+00	7.77E-01	1.71E+00	4.18E+01	2.44E+01
SMARCA1	1.51E+03	6.36E-07	1.33E-06	1.69E+00	7.57E-01	1.69E+00	9.84E+01	5.83E+01

KLHL4	3.36E+01	6.38E-07	1.33E-06	2.82E+00	1.50E+00	2.82E+00	2.54E+00	9.01E-01
HEATR5A	3.10E+02	6.66E-07	1.38E-06	1.95E+00	9.63E-01	1.95E+00	2.12E+01	1.09E+01
XR_003031718.1	4.41E+01	6.68E-07	1.39E-06	4.36E+00	2.13E+00	4.36E+00	3.64E+00	8.35E-01
CEMIP2	2.65E+02	6.80E-07	1.41E-06	1.87E+00	9.04E-01	1.87E+00	1.79E+01	9.56E+00
DENND4C	5.66E+02	6.84E-07	1.42E-06	1.65E+00	7.25E-01	1.65E+00	3.65E+01	2.21E+01
TRIM56	1.37E+03	7.12E-07	1.48E-06	1.58E+00	6.63E-01	1.58E+00	8.72E+01	5.51E+01
PLN	8.58E+01	7.26E-07	1.50E-06	1.33E+01	3.73E+00	1.33E+01	8.02E+00	6.03E-01
RBMS2	2.17E+03	7.27E-07	1.50E-06	1.55E+00	6.33E-01	1.55E+00	1.37E+02	8.82E+01
TMEM173	2.24E+02	7.32E-07	1.51E-06	1.99E+00	9.90E-01	1.99E+00	1.53E+01	7.69E+00
SLC35D1	5.61E+01	7.33E-07	1.51E-06	1.98E+01	4.31E+00	1.98E+01	5.36E+00	2.70E-01
ZKSCAN5	1.04E+03	7.41E-07	1.52E-06	1.47E+00	5.58E-01	1.47E+00	6.43E+01	4.37E+01
SLITRK6	1.02E+02	7.42E-07	1.53E-06	2.59E+00	1.37E+00	2.59E+00	7.51E+00	2.90E+00
FYN	1.34E+03	7.48E-07	1.54E-06	1.48E+00	5.62E-01	1.48E+00	8.32E+01	5.64E+01
XR_003031804.1	6.51E+01	7.50E-07	1.54E-06	4.87E+00	2.28E+00	4.87E+00	5.46E+00	1.12E+00
PNPLA8	6.00E+02	7.55E-07	1.55E-06	1.52E+00	6.05E-01	1.52E+00	3.76E+01	2.48E+01
PIK3R1	2.43E+03	7.61E-07	1.56E-06	1.69E+00	7.60E-01	1.69E+00	1.59E+02	9.38E+01
ZNF770	2.31E+02	7.69E-07	1.57E-06	1.53E+00	6.16E-01	1.53E+00	1.45E+01	9.45E+00
EHBP1L1	1.75E+03	7.70E-07	1.57E-06	1.66E+00	7.34E-01	1.66E+00	1.13E+02	6.78E+01
DYRK2	7.62E+02	7.74E-07	1.58E-06	1.64E+00	7.14E-01	1.64E+00	4.92E+01	3.00E+01
XR_003034585.1	6.87E+01	7.86E-07	1.60E-06	5.22E+00	2.39E+00	5.22E+00	5.84E+00	1.12E+00
N4BP2L2	5.99E+02	8.15E-07	1.66E-06	1.54E+00	6.23E-01	1.54E+00	3.78E+01	2.45E+01
NCOA4	1.26E+03	8.20E-07	1.67E-06	1.39E+00	4.76E-01	1.39E+00	7.61E+01	5.47E+01
IGFBP6	4.19E+03	8.24E-07	1.67E-06	2.11E+00	1.08E+00	2.11E+00	2.92E+02	1.38E+02
CNST	2.05E+02	8.32E-07	1.69E-06	1.91E+00	9.33E-01	1.91E+00	1.40E+01	7.31E+00
RILP	1.44E+02	8.35E-07	1.69E-06	1.60E+00	6.77E-01	1.60E+00	9.09E+00	5.68E+00
XR_003037447.1	2.84E+00	8.39E-07	1.70E-06	1.39E+01	3.79E+00	1.39E+01	2.66E-01	1.92E-02
MSS51	1.94E+02	8.66E-07	1.75E-06	1.72E+00	7.86E-01	1.72E+00	1.27E+01	7.35E+00
UBQLN2	8.45E+02	8.73E-07	1.76E-06	1.55E+00	6.29E-01	1.55E+00	5.31E+01	3.44E+01
ASCC3	3.43E+02	8.74E-07	1.76E-06	1.57E+00	6.53E-01	1.57E+00	2.17E+01	1.38E+01
PTPRJ	5.41E+02	8.76E-07	1.76E-06	2.24E+00	1.16E+00	2.24E+00	3.86E+01	1.72E+01
ANXA5	6.18E+03	8.77E-07	1.76E-06	1.67E+00	7.43E-01	1.67E+00	4.00E+02	2.39E+02
LOC515551	1.05E+03	8.83E-07	1.77E-06	1.38E+00	4.68E-01	1.38E+00	6.35E+01	4.59E+01
SERTAD4	2.56E+02	8.91E-07	1.79E-06	3.68E+00	1.88E+00	3.68E+00	2.06E+01	5.61E+00

NEDD4	3.66E+02	8.93E-07	1.79E-06	1.65E+00	7.22E-01	1.65E+00	2.37E+01	1.44E+01
XR_003031806.1	2.60E+02	8.94E-07	1.79E-06	3.15E+00	1.66E+00	3.15E+00	2.00E+01	6.35E+00
LOC514507	1.30E+02	8.96E-07	1.79E-06	2.00E+00	1.00E+00	2.00E+00	8.89E+00	4.44E+00
IBTK	8.41E+02	9.00E-07	1.80E-06	1.52E+00	6.04E-01	1.52E+00	5.26E+01	3.46E+01
POGZ	2.60E+03	9.05E-07	1.81E-06	1.55E+00	6.31E-01	1.55E+00	1.64E+02	1.06E+02
PICALM	2.71E+03	9.06E-07	1.81E-06	1.38E+00	4.60E-01	1.38E+00	1.63E+02	1.19E+02
PHACTR4	1.27E+03	9.09E-07	1.81E-06	1.61E+00	6.91E-01	1.61E+00	8.13E+01	5.04E+01
XR_003030700.1	6.16E+01	9.11E-07	1.81E-06	4.96E+00	2.31E+00	4.96E+00	5.18E+00	1.04E+00
EVC2	8.23E+02	9.15E-07	1.82E-06	1.59E+00	6.68E-01	1.59E+00	5.20E+01	3.27E+01
ANG2	7.67E+01	9.22E-07	1.83E-06	2.56E+00	1.36E+00	2.56E+00	5.63E+00	2.20E+00
PODXL	2.79E+03	9.34E-07	1.85E-06	1.97E+00	9.76E-01	1.97E+00	1.92E+02	9.76E+01
MAGT1	9.09E+02	9.47E-07	1.88E-06	1.42E+00	5.08E-01	1.42E+00	5.54E+01	3.89E+01
SIK2	1.56E+03	9.59E-07	1.90E-06	1.71E+00	7.71E-01	1.71E+00	1.02E+02	5.96E+01
ZNF25	1.09E+03	9.65E-07	1.91E-06	1.97E+00	9.76E-01	1.97E+00	7.47E+01	3.80E+01
XR_239281.4	4.57E+02	9.72E-07	1.92E-06	1.99E+00	9.91E-01	1.99E+00	3.14E+01	1.58E+01
KIF13A	1.16E+03	9.90E-07	1.95E-06	1.80E+00	8.48E-01	1.80E+00	7.73E+01	4.30E+01
SNRNP48	9.14E+02	9.93E-07	1.96E-06	1.39E+00	4.70E-01	1.39E+00	5.51E+01	3.98E+01
PSMD10	6.60E+02	9.97E-07	1.96E-06	1.43E+00	5.13E-01	1.43E+00	4.04E+01	2.83E+01
XR_233909.4	3.21E+00	1.02E-06	2.00E-06	3.60E+00	1.85E+00	3.60E+00	2.52E-01	6.99E-02
LOC784108	4.09E+00	1.03E-06	2.02E-06	1.86E+01	4.22E+00	1.86E+01	3.89E-01	2.09E-02
PLAT	2.76E+03	1.05E-06	2.06E-06	2.48E+00	1.31E+00	2.48E+00	2.03E+02	8.17E+01
NUDT7	4.90E+02	1.06E-06	2.08E-06	1.54E+00	6.23E-01	1.54E+00	3.08E+01	2.00E+01
LOC107133024	1.95E+01	1.08E-06	2.11E-06	3.14E+00	1.65E+00	3.14E+00	1.52E+00	4.83E-01
PIGN	5.40E+02	1.08E-06	2.11E-06	1.49E+00	5.74E-01	1.49E+00	3.36E+01	2.26E+01
ZADH2	9.59E+02	1.11E-06	2.16E-06	1.75E+00	8.07E-01	1.75E+00	6.35E+01	3.63E+01
PCDHA13	4.44E+02	1.14E-06	2.23E-06	1.86E+00	8.93E-01	1.86E+00	2.98E+01	1.60E+01
ACE2	9.94E+01	1.14E-06	2.23E-06	1.40E+01	3.81E+00	1.40E+01	9.32E+00	6.66E-01
MXRA7	2.34E+03	1.14E-06	2.23E-06	1.61E+00	6.90E-01	1.61E+00	1.50E+02	9.28E+01
GXYLT1	6.34E+02	1.19E-06	2.31E-06	1.90E+00	9.26E-01	1.90E+00	4.29E+01	2.26E+01
WASL	1.18E+03	1.22E-06	2.37E-06	1.70E+00	7.67E-01	1.70E+00	7.69E+01	4.52E+01
XR_003031719.1	6.88E+01	1.24E-06	2.41E-06	3.05E+00	1.61E+00	3.05E+00	5.29E+00	1.73E+00
ZNF317	4.49E+02	1.26E-06	2.44E-06	1.50E+00	5.85E-01	1.50E+00	2.80E+01	1.86E+01
FXYD6	4.45E+03	1.31E-06	2.53E-06	1.57E+00	6.55E-01	1.57E+00	2.84E+02	1.80E+02

SETD6	3.60E+02	1.31E-06	2.53E-06	1.49E+00	5.72E-01	1.49E+00	2.23E+01	1.50E+01
FAM13B	6.66E+02	1.32E-06	2.55E-06	1.70E+00	7.68E-01	1.70E+00	4.35E+01	2.55E+01
TMEM263	1.82E+03	1.32E-06	2.55E-06	1.52E+00	6.03E-01	1.52E+00	1.14E+02	7.48E+01
PLK2	9.17E+02	1.38E-06	2.65E-06	1.66E+00	7.31E-01	1.66E+00	5.93E+01	3.57E+01
JUNB	6.38E+02	1.38E-06	2.65E-06	2.50E+00	1.32E+00	2.50E+00	4.69E+01	1.87E+01
XR_003037466.1	1.69E+01	1.42E-06	2.73E-06	6.67E+00	2.74E+00	6.67E+00	1.47E+00	2.21E-01
LOC101908048	2.47E+01	1.43E-06	2.74E-06	3.27E+00	1.71E+00	3.27E+00	1.93E+00	5.91E-01
YES1	8.16E+02	1.45E-06	2.78E-06	1.90E+00	9.26E-01	1.90E+00	5.53E+01	2.91E+01
SCARA5	1.79E+03	1.49E-06	2.85E-06	2.60E+00	1.38E+00	2.60E+00	1.32E+02	5.09E+01
KCNN3	1.95E+02	1.53E-06	2.92E-06	2.25E+00	1.17E+00	2.25E+00	1.40E+01	6.22E+00
EDNRA	8.04E+02	1.54E-06	2.95E-06	2.00E+00	9.97E-01	2.00E+00	5.53E+01	2.77E+01
XR_814565.3	1.19E+03	1.55E-06	2.96E-06	1.56E+00	6.39E-01	1.56E+00	7.56E+01	4.85E+01
IRAK4	6.56E+02	1.55E-06	2.96E-06	1.43E+00	5.13E-01	1.43E+00	4.02E+01	2.82E+01
REM1	1.07E+03	1.60E-06	3.06E-06	1.86E+00	8.98E-01	1.86E+00	7.15E+01	3.84E+01
KLF2	6.52E+02	1.61E-06	3.07E-06	2.35E+00	1.23E+00	2.35E+00	4.70E+01	2.00E+01
CYFIP1	2.19E+03	1.61E-06	3.07E-06	1.46E+00	5.48E-01	1.46E+00	1.35E+02	9.23E+01
ITIH4	2.40E+02	1.62E-06	3.08E-06	3.44E+00	1.78E+00	3.44E+00	1.89E+01	5.48E+00
F5	2.46E+01	1.65E-06	3.13E-06	2.71E+00	1.44E+00	2.71E+00	1.84E+00	6.80E-01
TACC1	1.54E+03	1.65E-06	3.14E-06	1.82E+00	8.62E-01	1.82E+00	1.02E+02	5.63E+01
CCL16	5.74E+01	1.67E-06	3.17E-06	3.25E+00	1.70E+00	3.25E+00	4.47E+00	1.38E+00
MAP2K1	7.39E+02	1.70E-06	3.21E-06	1.33E+00	4.14E-01	1.33E+00	4.41E+01	3.31E+01
TLR4	8.24E+01	1.71E-06	3.23E-06	6.11E+00	2.61E+00	6.11E+00	7.15E+00	1.17E+00
XR_001501236.2	8.97E+00	1.71E-06	3.23E-06	4.10E+00	2.04E+00	4.10E+00	7.35E-01	1.79E-01
SLC25A16	1.80E+02	1.73E-06	3.27E-06	1.62E+00	6.95E-01	1.62E+00	1.15E+01	7.09E+00
XR_003032719.1	4.32E+01	1.74E-06	3.28E-06	3.70E+00	1.89E+00	3.70E+00	3.47E+00	9.39E-01
DUSP16	5.42E+02	1.74E-06	3.28E-06	1.93E+00	9.48E-01	1.93E+00	3.69E+01	1.92E+01
CSNK1G3	6.45E+02	1.76E-06	3.30E-06	1.61E+00	6.88E-01	1.61E+00	4.13E+01	2.57E+01
PLAGL1	1.73E+03	1.78E-06	3.34E-06	2.19E+00	1.13E+00	2.19E+00	1.21E+02	5.55E+01
XR_003035941.1	5.37E+01	1.80E-06	3.37E-06	2.81E+00	1.49E+00	2.81E+00	4.06E+00	1.45E+00
TMEM252	3.58E+01	1.82E-06	3.41E-06	3.33E+00	1.73E+00	3.33E+00	2.80E+00	8.41E-01
EDEM1	3.35E+01	1.82E-06	3.41E-06	2.48E+01	4.64E+00	2.48E+01	3.22E+00	1.30E-01
SYNDIG1	1.43E+02	1.82E-06	3.41E-06	6.88E+00	2.78E+00	6.88E+00	1.26E+01	1.84E+00
CD164	3.26E+03	1.87E-06	3.50E-06	1.47E+00	5.51E-01	1.47E+00	2.01E+02	1.37E+02

XR_236232.4	2.11E+02	1.89E-06	3.53E-06	2.20E+00	1.14E+00	2.20E+00	1.49E+01	6.77E+00
COL6A1	3.58E+04	1.92E-06	3.58E-06	2.16E+00	1.11E+00	2.16E+00	2.51E+03	1.16E+03
XR_003029913.1	3.00E+01	1.95E-06	3.63E-06	2.65E+00	1.41E+00	2.65E+00	2.23E+00	8.40E-01
XR_003031554.1	4.12E+01	1.96E-06	3.64E-06	3.90E+00	1.96E+00	3.90E+00	3.35E+00	8.59E-01
ZSWIM6	4.54E+02	1.98E-06	3.68E-06	1.87E+00	9.04E-01	1.87E+00	3.05E+01	1.63E+01
KCNJ8	3.33E+02	1.99E-06	3.69E-06	2.79E+00	1.48E+00	2.79E+00	2.50E+01	8.97E+00
POT1	2.51E+02	1.99E-06	3.69E-06	1.58E+00	6.61E-01	1.58E+00	1.60E+01	1.01E+01
CD300LG	8.07E+02	1.99E-06	3.69E-06	2.75E+00	1.46E+00	2.75E+00	6.05E+01	2.20E+01
BICD2	1.42E+03	2.01E-06	3.72E-06	1.45E+00	5.38E-01	1.45E+00	8.72E+01	6.01E+01
PMVK	5.80E+02	2.01E-06	3.72E-06	1.73E+00	7.94E-01	1.73E+00	3.79E+01	2.19E+01
SEL1L3	4.56E+02	2.04E-06	3.76E-06	2.41E+00	1.27E+00	2.41E+00	3.31E+01	1.37E+01
CLK4	1.10E+03	2.04E-06	3.76E-06	1.46E+00	5.48E-01	1.46E+00	6.81E+01	4.66E+01
EIF4G2	1.22E+04	2.06E-06	3.81E-06	1.48E+00	5.64E-01	1.48E+00	7.55E+02	5.11E+02
XR_003029437.1	1.80E+01	2.12E-06	3.91E-06	3.98E+00	1.99E+00	3.98E+00	1.46E+00	3.67E-01
XR_001494399.2	2.43E+01	2.14E-06	3.93E-06	3.16E+00	1.66E+00	3.16E+00	1.88E+00	5.94E-01
XR_003034233.1	4.53E+03	2.15E-06	3.95E-06	1.93E+00	9.48E-01	1.93E+00	3.08E+02	1.60E+02
XR_003032538.1	4.93E+01	2.15E-06	3.96E-06	7.95E+00	2.99E+00	7.95E+00	4.44E+00	5.58E-01
SYT4	6.80E+02	2.16E-06	3.96E-06	2.74E+00	1.45E+00	2.74E+00	5.12E+01	1.87E+01
SLC9B2	1.23E+02	2.18E-06	3.99E-06	1.98E+00	9.89E-01	1.98E+00	8.48E+00	4.27E+00
F2R	5.86E+02	2.19E-06	4.01E-06	1.86E+00	8.99E-01	1.86E+00	3.92E+01	2.10E+01
LOC505918	2.55E+02	2.21E-06	4.03E-06	1.61E+00	6.83E-01	1.61E+00	1.63E+01	1.02E+01
TMLHE	9.89E+01	2.21E-06	4.03E-06	1.54E+00	6.19E-01	1.54E+00	6.22E+00	4.05E+00
KIAA1211	7.57E+01	2.23E-06	4.06E-06	3.41E+00	1.77E+00	3.41E+00	6.00E+00	1.76E+00
CCNI	6.73E+03	2.25E-06	4.10E-06	1.39E+00	4.72E-01	1.39E+00	4.07E+02	2.93E+02
LCLAT1	3.73E+02	2.25E-06	4.10E-06	1.62E+00	6.96E-01	1.62E+00	2.40E+01	1.48E+01
QTRT2	2.22E+02	2.29E-06	4.17E-06	1.49E+00	5.79E-01	1.49E+00	1.38E+01	9.27E+00
DCUN1D1	8.19E+02	2.31E-06	4.21E-06	1.52E+00	6.07E-01	1.52E+00	5.14E+01	3.37E+01
UGDH	7.56E+02	2.32E-06	4.21E-06	1.43E+00	5.20E-01	1.43E+00	4.64E+01	3.24E+01
TMEM135	2.15E+02	2.35E-06	4.27E-06	1.73E+00	7.91E-01	1.73E+00	1.42E+01	8.20E+00
TNS1	9.08E+03	2.37E-06	4.30E-06	1.63E+00	7.07E-01	1.63E+00	5.81E+02	3.56E+02
FAM20B	1.10E+03	2.40E-06	4.33E-06	1.66E+00	7.35E-01	1.66E+00	7.09E+01	4.26E+01
XR_003033202.1	7.87E+00	2.40E-06	4.34E-06	2.48E+01	4.63E+00	2.48E+01	7.59E-01	3.06E-02
LOC112445980	3.61E+01	2.42E-06	4.37E-06	2.70E+00	1.43E+00	2.70E+00	2.68E+00	9.92E-01

LOC112445906	9.81E+00	2.45E-06	4.43E-06	2.51E+03	1.13E+01	2.51E+03	9.81E-01	3.90E-04
TBC1D23	1.16E+03	2.46E-06	4.43E-06	1.42E+00	5.02E-01	1.42E+00	7.04E+01	4.97E+01
CARNMT1	1.90E+02	2.52E-06	4.53E-06	1.53E+00	6.14E-01	1.53E+00	1.19E+01	7.78E+00
KCTD6	3.86E+02	2.54E-06	4.56E-06	1.46E+00	5.44E-01	1.46E+00	2.38E+01	1.63E+01
LOC107132952	1.71E+01	2.59E-06	4.66E-06	3.23E+00	1.69E+00	3.23E+00	1.32E+00	4.08E-01
CSRP1	3.97E+03	2.60E-06	4.66E-06	1.74E+00	7.98E-01	1.74E+00	2.61E+02	1.50E+02
ST8SIA4	5.30E+01	2.60E-06	4.66E-06	5.30E+00	2.41E+00	5.30E+00	4.52E+00	8.53E-01
RYBP	1.30E+03	2.66E-06	4.76E-06	1.72E+00	7.82E-01	1.72E+00	8.51E+01	4.95E+01
PELI1	4.56E+02	2.71E-06	4.85E-06	1.44E+00	5.29E-01	1.44E+00	2.79E+01	1.94E+01
GOLT1B	1.49E+02	2.71E-06	4.85E-06	1.71E+00	7.70E-01	1.71E+00	9.64E+00	5.65E+00
SLC12A4	2.49E+03	2.77E-06	4.95E-06	1.43E+00	5.20E-01	1.43E+00	1.52E+02	1.06E+02
COL5A1	2.72E+04	2.80E-06	5.00E-06	1.90E+00	9.29E-01	1.90E+00	1.83E+03	9.63E+02
EHD2	3.92E+03	2.81E-06	5.01E-06	1.88E+00	9.08E-01	1.88E+00	2.65E+02	1.41E+02
JADE2	1.85E+03	2.87E-06	5.12E-06	1.76E+00	8.15E-01	1.76E+00	1.22E+02	6.91E+01
XR_003029914.1	1.10E+02	2.89E-06	5.14E-06	2.37E+00	1.24E+00	2.37E+00	7.90E+00	3.34E+00
APOL3	4.38E+02	2.90E-06	5.15E-06	1.72E+00	7.85E-01	1.72E+00	2.86E+01	1.66E+01
SLC39A9	8.37E+02	2.90E-06	5.15E-06	1.59E+00	6.68E-01	1.59E+00	5.33E+01	3.36E+01
LYL1	1.77E+03	2.90E-06	5.15E-06	1.75E+00	8.09E-01	1.75E+00	1.16E+02	6.60E+01
PCNX4	4.84E+02	2.93E-06	5.19E-06	1.78E+00	8.29E-01	1.78E+00	3.21E+01	1.81E+01
XR_003036865.1	3.40E+00	2.96E-06	5.24E-06	6.32E+00	2.66E+00	6.32E+00	2.94E-01	4.65E-02
MYADM	2.57E+03	2.98E-06	5.27E-06	2.04E+00	1.03E+00	2.04E+00	1.78E+02	8.73E+01
ITPR2	3.52E+02	2.99E-06	5.28E-06	2.01E+00	1.01E+00	2.01E+00	2.44E+01	1.21E+01
XR_003030645.1	5.48E+02	3.01E-06	5.32E-06	1.88E+00	9.14E-01	1.88E+00	3.66E+01	1.94E+01
BMP5	2.37E+01	3.02E-06	5.33E-06	2.97E+00	1.57E+00	2.97E+00	1.80E+00	6.06E-01
USP8	1.49E+03	3.13E-06	5.52E-06	1.48E+00	5.70E-01	1.48E+00	9.30E+01	6.27E+01
LYSMD3	2.54E+02	3.14E-06	5.52E-06	1.72E+00	7.84E-01	1.72E+00	1.66E+01	9.66E+00
ADAMTS9	7.29E+02	3.16E-06	5.56E-06	2.11E+00	1.08E+00	2.11E+00	5.12E+01	2.42E+01
MSH3	5.89E+02	3.18E-06	5.58E-06	1.41E+00	4.96E-01	1.41E+00	3.59E+01	2.55E+01
BIVM	7.35E+02	3.21E-06	5.63E-06	1.42E+00	5.09E-01	1.42E+00	4.49E+01	3.16E+01
LOC112445030	6.06E+02	3.23E-06	5.66E-06	1.98E+00	9.85E-01	1.98E+00	4.13E+01	2.09E+01
PHACTR2	4.99E+02	3.24E-06	5.67E-06	1.68E+00	7.49E-01	1.68E+00	3.24E+01	1.93E+01
MR1	6.77E+01	3.29E-06	5.76E-06	2.24E+00	1.16E+00	2.24E+00	4.76E+00	2.12E+00
ARL4C	1.88E+02	3.30E-06	5.77E-06	1.65E+00	7.19E-01	1.65E+00	1.22E+01	7.39E+00

STXBP4	4.70E+02	3.31E-06	5.79E-06	1.68E+00	7.50E-01	1.68E+00	3.06E+01	1.82E+01
MEIS1	1.24E+03	3.38E-06	5.89E-06	2.11E+00	1.08E+00	2.11E+00	8.70E+01	4.13E+01
CTNNA3	2.79E+01	3.43E-06	5.97E-06	2.79E+00	1.48E+00	2.79E+00	2.09E+00	7.51E-01
LOC787074	3.52E+02	3.44E-06	6.00E-06	1.85E+00	8.91E-01	1.85E+00	2.37E+01	1.28E+01
FAM19A3	1.48E+02	3.45E-06	6.00E-06	2.89E+00	1.53E+00	2.89E+00	1.12E+01	3.86E+00
RGS9	2.40E+02	3.46E-06	6.02E-06	2.37E+00	1.24E+00	2.37E+00	1.72E+01	7.28E+00
ENAH	2.13E+03	3.47E-06	6.03E-06	1.92E+00	9.43E-01	1.92E+00	1.45E+02	7.53E+01
STAU2	8.75E+02	3.53E-06	6.13E-06	1.39E+00	4.76E-01	1.39E+00	5.30E+01	3.81E+01
XR_816906.3	3.55E+02	3.55E-06	6.15E-06	3.72E+00	1.89E+00	3.72E+00	2.85E+01	7.67E+00
XR_003037047.1	1.55E+02	3.56E-06	6.15E-06	4.48E+00	2.16E+00	4.48E+00	1.29E+01	2.88E+00
NOL9	1.58E+01	3.62E-06	6.26E-06	9.24E+00	3.21E+00	9.24E+00	1.43E+00	1.55E-01
XR_237771.4	9.95E+01	3.64E-06	6.28E-06	4.35E+00	2.12E+00	4.35E+00	8.21E+00	1.89E+00
FSD1L	2.23E+02	3.72E-06	6.42E-06	1.56E+00	6.40E-01	1.56E+00	1.41E+01	9.02E+00
TIMP2	9.81E+03	3.73E-06	6.43E-06	1.66E+00	7.30E-01	1.66E+00	6.34E+02	3.82E+02
TINAGL1	8.64E+02	3.75E-06	6.46E-06	2.15E+00	1.10E+00	2.15E+00	6.11E+01	2.84E+01
METTL24	2.53E+01	3.78E-06	6.51E-06	2.70E+00	1.43E+00	2.70E+00	1.88E+00	6.96E-01
XR_001501953.2	2.70E+01	3.82E-06	6.56E-06	5.06E+00	2.34E+00	5.06E+00	2.30E+00	4.54E-01
AFG1L	4.26E+01	3.82E-06	6.56E-06	2.03E+00	1.02E+00	2.03E+00	2.95E+00	1.45E+00
PAN3	1.28E+03	3.89E-06	6.67E-06	1.66E+00	7.31E-01	1.66E+00	8.26E+01	4.98E+01
XR_003035736.1	3.88E+01	3.90E-06	6.69E-06	4.71E+00	2.24E+00	4.71E+00	3.25E+00	6.89E-01
RBM18	2.44E+02	3.93E-06	6.73E-06	1.45E+00	5.39E-01	1.45E+00	1.50E+01	1.03E+01
MXRA8	5.00E+03	3.95E-06	6.77E-06	1.97E+00	9.76E-01	1.97E+00	3.42E+02	1.74E+02
ARHGAP17	1.36E+03	3.99E-06	6.82E-06	1.39E+00	4.73E-01	1.39E+00	8.27E+01	5.96E+01
MTDH	1.78E+03	4.00E-06	6.82E-06	1.40E+00	4.88E-01	1.40E+00	1.08E+02	7.72E+01
AGO4	5.52E+02	4.04E-06	6.89E-06	1.48E+00	5.63E-01	1.48E+00	3.42E+01	2.32E+01
MGC139164	1.38E+02	4.04E-06	6.89E-06	1.94E+00	9.60E-01	1.94E+00	9.41E+00	4.84E+00
ZNF8	7.06E+02	4.07E-06	6.93E-06	1.96E+00	9.73E-01	1.96E+00	4.80E+01	2.45E+01
PRPF38B	1.46E+03	4.14E-06	7.04E-06	1.30E+00	3.80E-01	1.30E+00	8.64E+01	6.64E+01
PEG3	1.29E+04	4.20E-06	7.13E-06	1.92E+00	9.44E-01	1.92E+00	8.80E+02	4.57E+02
AKAP12	4.91E+03	4.20E-06	7.13E-06	2.75E+00	1.46E+00	2.75E+00	3.69E+02	1.34E+02
ATP6V1A	6.54E+02	4.22E-06	7.15E-06	1.53E+00	6.13E-01	1.53E+00	4.11E+01	2.69E+01
OTUD6B	5.02E+02	4.22E-06	7.15E-06	1.38E+00	4.60E-01	1.38E+00	3.03E+01	2.20E+01
KANK2	5.02E+03	4.23E-06	7.17E-06	1.55E+00	6.34E-01	1.55E+00	3.16E+02	2.04E+02

XR_003037543.1	5.97E+01	4.24E-06	7.18E-06	2.49E+00	1.31E+00	2.49E+00	4.33E+00	1.74E+00
EPB41L1	8.27E+03	4.29E-06	7.24E-06	1.55E+00	6.34E-01	1.55E+00	5.23E+02	3.37E+02
DCN	2.27E+04	4.31E-06	7.27E-06	1.70E+00	7.68E-01	1.70E+00	1.48E+03	8.70E+02
LOC615521	1.06E+01	4.31E-06	7.27E-06	3.01E+00	1.59E+00	3.01E+00	8.10E-01	2.69E-01
CD93	1.80E+03	4.32E-06	7.27E-06	1.82E+00	8.65E-01	1.82E+00	1.20E+02	6.59E+01
Sep-11	2.19E+03	4.44E-06	7.48E-06	1.62E+00	6.98E-01	1.62E+00	1.41E+02	8.70E+01
LOC616063	9.56E+02	4.46E-06	7.50E-06	2.48E+00	1.31E+00	2.48E+00	6.96E+01	2.80E+01
TM9SF3	2.59E+03	4.50E-06	7.56E-06	1.35E+00	4.29E-01	1.35E+00	1.55E+02	1.15E+02
XR_001501425.2	1.02E+01	4.52E-06	7.59E-06	3.31E+00	1.73E+00	3.31E+00	7.94E-01	2.40E-01
HLF	4.81E+02	4.53E-06	7.59E-06	1.94E+00	9.56E-01	1.94E+00	3.28E+01	1.69E+01
GADD45A	6.88E+02	4.54E-06	7.61E-06	2.36E+00	1.24E+00	2.36E+00	4.97E+01	2.11E+01
XR_003030701.1	1.63E+01	4.58E-06	7.67E-06	7.58E+00	2.92E+00	7.58E+00	1.46E+00	1.92E-01
LOC511847	1.69E+02	4.70E-06	7.86E-06	1.64E+00	7.12E-01	1.64E+00	1.09E+01	6.63E+00
MAST2	4.03E+03	4.72E-06	7.88E-06	1.66E+00	7.30E-01	1.66E+00	2.61E+02	1.57E+02
TRPS1	5.40E+02	4.89E-06	8.16E-06	1.62E+00	6.94E-01	1.62E+00	3.47E+01	2.15E+01
PPARA	8.95E+02	4.97E-06	8.29E-06	1.82E+00	8.64E-01	1.82E+00	6.02E+01	3.31E+01
CNN1	5.64E+02	5.02E-06	8.36E-06	5.37E+00	2.43E+00	5.37E+00	4.82E+01	8.96E+00
XR_001500650.2	2.14E+02	5.05E-06	8.41E-06	5.21E+00	2.38E+00	5.21E+00	1.82E+01	3.49E+00
LOC104973175	4.22E+01	5.07E-06	8.43E-06	2.09E+00	1.06E+00	2.09E+00	2.93E+00	1.40E+00
XR_003037449.1	4.29E+00	5.10E-06	8.47E-06	9.84E+00	3.30E+00	9.84E+00	3.91E-01	3.98E-02
RORA	3.13E+02	5.28E-06	8.76E-06	1.68E+00	7.48E-01	1.68E+00	2.04E+01	1.22E+01
PIP4K2A	7.96E+02	5.33E-06	8.84E-06	1.39E+00	4.73E-01	1.39E+00	4.83E+01	3.48E+01
HES7	1.64E+01	5.64E-06	9.34E-06	3.09E+00	1.63E+00	3.09E+00	1.26E+00	4.08E-01
YAP1	2.54E+03	5.72E-06	9.47E-06	1.64E+00	7.11E-01	1.64E+00	1.64E+02	1.00E+02
GALNT15	9.89E+01	5.95E-06	9.84E-06	6.43E+00	2.69E+00	6.43E+00	8.63E+00	1.34E+00
XR_003032576.1	2.83E+01	5.95E-06	9.84E-06	2.57E+00	1.36E+00	2.57E+00	2.09E+00	8.11E-01
CLN5	1.56E+02	5.97E-06	9.86E-06	1.64E+00	7.18E-01	1.64E+00	1.01E+01	6.12E+00
CPT1A	9.22E+02	6.08E-06	1.00E-05	1.74E+00	7.98E-01	1.74E+00	6.07E+01	3.49E+01
XR_003032722.1	5.61E+00	6.14E-06	1.01E-05	6.26E+00	2.65E+00	6.26E+00	4.90E-01	7.83E-02
LOC511531	1.32E+02	6.14E-06	1.01E-05	2.18E+00	1.13E+00	2.18E+00	9.20E+00	4.22E+00
DEPTOR	6.22E+02	6.17E-06	1.01E-05	1.71E+00	7.71E-01	1.71E+00	4.08E+01	2.39E+01
ARHGEF38	1.91E+01	6.17E-06	1.01E-05	4.86E+00	2.28E+00	4.86E+00	1.61E+00	3.30E-01
XR_003035193.1	3.63E+01	6.28E-06	1.03E-05	3.12E+00	1.64E+00	3.12E+00	2.78E+00	8.93E-01

TMEM260	7.56E+02	6.33E-06	1.04E-05	1.54E+00	6.19E-01	1.54E+00	4.76E+01	3.10E+01
SNX33	3.13E+02	6.36E-06	1.04E-05	1.62E+00	6.99E-01	1.62E+00	2.01E+01	1.24E+01
TMEM63A	1.94E+03	6.40E-06	1.05E-05	1.54E+00	6.26E-01	1.54E+00	1.22E+02	7.92E+01
GATAD1	9.97E+01	6.41E-06	1.05E-05	2.51E+00	1.33E+00	2.51E+00	7.29E+00	2.90E+00
XR_234534.4	1.84E+00	6.45E-06	1.05E-05	1.14E+01	3.51E+00	1.14E+01	1.69E-01	1.48E-02
NCEH1	1.93E+02	6.45E-06	1.05E-05	1.85E+00	8.85E-01	1.85E+00	1.29E+01	7.00E+00
PSME4	7.73E+02	6.63E-06	1.08E-05	1.49E+00	5.75E-01	1.49E+00	4.81E+01	3.23E+01
XR_003037458.1	1.15E+01	6.63E-06	1.08E-05	4.99E+00	2.32E+00	4.99E+00	9.68E-01	1.94E-01
LIX1L	1.36E+03	6.65E-06	1.08E-05	1.59E+00	6.68E-01	1.59E+00	8.65E+01	5.44E+01
RBMS1	8.44E+02	6.74E-06	1.10E-05	1.47E+00	5.52E-01	1.47E+00	5.26E+01	3.59E+01
KDR	2.50E+03	6.79E-06	1.10E-05	1.83E+00	8.73E-01	1.83E+00	1.69E+02	9.21E+01
XR_003034845.1	3.56E+01	6.80E-06	1.10E-05	2.47E+00	1.30E+00	2.47E+00	2.58E+00	1.04E+00
TBC1D16	1.73E+03	7.01E-06	1.14E-05	1.43E+00	5.17E-01	1.43E+00	1.06E+02	7.42E+01
CHAD	1.70E+02	7.04E-06	1.14E-05	2.36E+00	1.24E+00	2.36E+00	1.22E+01	5.16E+00
CHST3	2.00E+03	7.09E-06	1.15E-05	1.91E+00	9.37E-01	1.91E+00	1.36E+02	7.09E+01
TOPORS	8.01E+02	7.16E-06	1.16E-05	1.35E+00	4.28E-01	1.35E+00	4.77E+01	3.55E+01
LOC112449111	1.56E+02	7.18E-06	1.16E-05	2.54E+00	1.34E+00	2.54E+00	1.14E+01	4.49E+00
SLIT2	3.26E+02	7.20E-06	1.16E-05	2.69E+00	1.43E+00	2.69E+00	2.45E+01	9.10E+00
ELMSAN1	1.48E+03	7.21E-06	1.16E-05	1.63E+00	7.04E-01	1.63E+00	9.49E+01	5.83E+01
HOMER1	1.94E+02	7.22E-06	1.16E-05	1.84E+00	8.78E-01	1.84E+00	1.29E+01	7.04E+00
EOGT	3.12E+02	7.23E-06	1.16E-05	1.75E+00	8.08E-01	1.75E+00	2.05E+01	1.17E+01
RASAL2	3.67E+02	7.28E-06	1.17E-05	1.70E+00	7.67E-01	1.70E+00	2.39E+01	1.40E+01
PUS10	4.34E+02	7.51E-06	1.21E-05	1.33E+00	4.11E-01	1.33E+00	2.58E+01	1.94E+01
LOC112449284	5.79E+01	7.55E-06	1.21E-05	1.85E+00	8.89E-01	1.85E+00	3.89E+00	2.10E+00
ABCE1	8.17E+02	7.60E-06	1.22E-05	1.34E+00	4.19E-01	1.34E+00	4.88E+01	3.65E+01
XR_003033508.1	1.17E+01	7.61E-06	1.22E-05	2.82E+00	1.49E+00	2.82E+00	8.80E-01	3.12E-01
DBP	5.77E+02	7.63E-06	1.22E-05	1.56E+00	6.41E-01	1.56E+00	3.66E+01	2.34E+01
RXFP1	5.26E+01	7.78E-06	1.24E-05	1.07E+02	6.73E+00	1.07E+02	5.21E+00	4.90E-02
ZNF367	2.24E+02	7.80E-06	1.25E-05	1.49E+00	5.77E-01	1.49E+00	1.38E+01	9.28E+00
EHHADH	1.84E+02	7.88E-06	1.26E-05	1.62E+00	6.94E-01	1.62E+00	1.18E+01	7.27E+00
ZBTB4	2.82E+03	7.88E-06	1.26E-05	1.60E+00	6.80E-01	1.60E+00	1.79E+02	1.12E+02
XR_003031377.1	6.68E+01	7.89E-06	1.26E-05	2.41E+00	1.27E+00	2.41E+00	4.87E+00	2.02E+00
XR_003037720.1	1.95E+01	8.24E-06	1.31E-05	1.80E+00	8.47E-01	1.80E+00	1.29E+00	7.18E-01

PRDM16	8.71E+01	8.74E-06	1.39E-05	2.25E+00	1.17E+00	2.25E+00	6.18E+00	2.75E+00
XR_003034366.1	8.92E+00	8.80E-06	1.40E-05	6.72E+00	2.75E+00	6.72E+00	7.80E-01	1.16E-01
ZFP62	4.51E+02	8.81E-06	1.40E-05	1.57E+00	6.54E-01	1.57E+00	2.86E+01	1.82E+01
NUCB1	9.48E+03	8.85E-06	1.40E-05	1.40E+00	4.86E-01	1.40E+00	5.75E+02	4.11E+02
NPY1R	3.66E+01	8.87E-06	1.41E-05	2.47E+00	1.31E+00	2.47E+00	2.69E+00	1.09E+00
FGR	1.63E+02	8.95E-06	1.42E-05	1.72E+00	7.86E-01	1.72E+00	1.06E+01	6.17E+00
C26H10orf82	6.14E+00	9.16E-06	1.45E-05	4.34E+00	2.12E+00	4.34E+00	5.05E-01	1.17E-01
KLHL18	7.17E+02	9.22E-06	1.46E-05	1.36E+00	4.43E-01	1.36E+00	4.31E+01	3.17E+01
PNISR	2.81E+03	9.33E-06	1.47E-05	1.58E+00	6.60E-01	1.58E+00	1.79E+02	1.13E+02
TDG	4.13E+02	9.34E-06	1.47E-05	1.56E+00	6.42E-01	1.56E+00	2.62E+01	1.68E+01
LOC509283	1.36E+03	9.44E-06	1.49E-05	1.68E+00	7.47E-01	1.68E+00	8.83E+01	5.26E+01
RALA	1.35E+03	9.45E-06	1.49E-05	1.30E+00	3.84E-01	1.30E+00	7.98E+01	6.11E+01
LOC101905725	6.05E+00	9.97E-06	1.57E-05	3.45E+00	1.79E+00	3.45E+00	4.71E-01	1.37E-01
FGF10	1.97E+02	9.98E-06	1.57E-05	3.04E+00	1.60E+00	3.04E+00	1.51E+01	4.97E+00
CLIC2	9.63E+01	1.00E-05	1.57E-05	2.05E+00	1.03E+00	2.05E+00	6.66E+00	3.25E+00
XR_003037739.1	1.61E+01	1.00E-05	1.57E-05	2.50E+00	1.32E+00	2.50E+00	1.18E+00	4.70E-01
PPP2R5E	9.16E+02	1.02E-05	1.60E-05	1.34E+00	4.22E-01	1.34E+00	5.48E+01	4.09E+01
XR_811487.3	2.41E+03	1.02E-05	1.60E-05	1.62E+00	6.99E-01	1.62E+00	1.54E+02	9.51E+01
LHFPL6	2.01E+03	1.03E-05	1.61E-05	2.06E+00	1.04E+00	2.06E+00	1.39E+02	6.76E+01
MFSD14B	8.11E+02	1.08E-05	1.69E-05	1.42E+00	5.07E-01	1.42E+00	4.96E+01	3.49E+01
XR_003030579.1	3.11E+02	1.11E-05	1.73E-05	2.53E+00	1.34E+00	2.53E+00	2.26E+01	8.96E+00
LY96	4.53E+01	1.14E-05	1.77E-05	3.22E+00	1.69E+00	3.22E+00	3.52E+00	1.09E+00
LOC112444773	1.47E+01	1.14E-05	1.78E-05	3.06E+00	1.62E+00	3.06E+00	1.13E+00	3.70E-01
ANXA4	1.12E+03	1.15E-05	1.79E-05	1.60E+00	6.82E-01	1.60E+00	7.15E+01	4.46E+01
SLC25A53	7.87E+01	1.15E-05	1.80E-05	1.80E+00	8.51E-01	1.80E+00	5.22E+00	2.90E+00
FBXL4	1.79E+02	1.16E-05	1.81E-05	1.45E+00	5.40E-01	1.45E+00	1.10E+01	7.54E+00
LOC104969678	2.58E+02	1.17E-05	1.82E-05	2.13E+00	1.09E+00	2.13E+00	1.81E+01	8.50E+00
ADRA1A	4.48E+01	1.20E-05	1.87E-05	3.39E+00	1.76E+00	3.39E+00	3.53E+00	1.04E+00
XR_003033178.1	7.41E+01	1.20E-05	1.87E-05	3.43E+00	1.78E+00	3.43E+00	5.85E+00	1.71E+00
XR_003033054.1	1.66E+02	1.22E-05	1.89E-05	2.30E+00	1.20E+00	2.30E+00	1.19E+01	5.16E+00
SCYL2	7.53E+02	1.23E-05	1.91E-05	1.92E+00	9.39E-01	1.92E+00	5.11E+01	2.66E+01
C1S	2.71E+03	1.24E-05	1.92E-05	2.28E+00	1.19E+00	2.28E+00	1.92E+02	8.41E+01
XR_816437.3	1.20E+01	1.24E-05	1.92E-05	2.41E+00	1.27E+00	2.41E+00	8.70E-01	3.61E-01

UFL1	1.94E+02	1.24E-05	1.92E-05	2.53E+00	1.34E+00	2.53E+00	1.42E+01	5.61E+00
LAMA1	9.25E+03	1.25E-05	1.92E-05	1.75E+00	8.06E-01	1.75E+00	6.08E+02	3.48E+02
DESI2	7.46E+02	1.25E-05	1.93E-05	1.50E+00	5.84E-01	1.50E+00	4.67E+01	3.11E+01
ETNK1	8.05E+02	1.25E-05	1.93E-05	1.74E+00	8.00E-01	1.74E+00	5.31E+01	3.05E+01
LOC784297	1.81E+03	1.26E-05	1.95E-05	1.85E+00	8.91E-01	1.85E+00	1.22E+02	6.57E+01
STRN	4.52E+02	1.27E-05	1.96E-05	1.50E+00	5.81E-01	1.50E+00	2.81E+01	1.88E+01
TPM4	6.62E+03	1.27E-05	1.96E-05	1.56E+00	6.41E-01	1.56E+00	4.18E+02	2.68E+02
CDKAL1	3.78E+02	1.28E-05	1.96E-05	1.36E+00	4.45E-01	1.36E+00	2.27E+01	1.67E+01
FGF7	7.68E+01	1.31E-05	2.02E-05	6.37E+00	2.67E+00	6.37E+00	6.68E+00	1.05E+00
XR_001500478.2	1.77E+00	1.32E-05	2.02E-05	4.63E+00	2.21E+00	4.63E+00	1.45E-01	3.14E-02
XR_003036761.1	3.74E+02	1.33E-05	2.04E-05	1.41E+00	5.00E-01	1.41E+00	2.28E+01	1.61E+01
XR_003033302.1	1.18E+02	1.33E-05	2.04E-05	2.10E+00	1.07E+00	2.10E+00	8.24E+00	3.92E+00
LCAT	6.47E+02	1.34E-05	2.05E-05	1.40E+00	4.85E-01	1.40E+00	3.93E+01	2.81E+01
XR_003038219.1	2.64E+01	1.34E-05	2.05E-05	5.24E+00	2.39E+00	5.24E+00	2.24E+00	4.26E-01
VIT	1.54E+01	1.35E-05	2.07E-05	1.23E+01	3.63E+00	1.23E+01	1.43E+00	1.16E-01
XR_003029448.1	2.26E+02	1.37E-05	2.09E-05	1.98E+00	9.84E-01	1.98E+00	1.55E+01	7.83E+00
XR_003031256.1	8.41E+01	1.38E-05	2.10E-05	2.74E+00	1.45E+00	2.74E+00	6.26E+00	2.29E+00
XR_001500250.2	1.85E+01	1.38E-05	2.10E-05	2.84E+00	1.51E+00	2.84E+00	1.40E+00	4.92E-01
TMEM87B	6.90E+02	1.38E-05	2.10E-05	1.37E+00	4.52E-01	1.37E+00	4.16E+01	3.04E+01
HIP1	3.05E+03	1.38E-05	2.10E-05	1.55E+00	6.30E-01	1.55E+00	1.92E+02	1.24E+02
GORAB	1.99E+02	1.39E-05	2.12E-05	1.57E+00	6.52E-01	1.57E+00	1.26E+01	8.03E+00
LOC112442256	3.16E+00	1.40E-05	2.12E-05	1.06E+02	6.72E+00	1.06E+02	3.13E-01	2.96E-03
XR_003032760.1	4.78E+01	1.41E-05	2.14E-05	2.57E+00	1.36E+00	2.57E+00	3.51E+00	1.37E+00
XR_003030697.1	4.17E+01	1.42E-05	2.15E-05	3.53E+00	1.82E+00	3.53E+00	3.30E+00	9.34E-01
SLFNL1	4.17E+01	1.43E-05	2.17E-05	2.62E+00	1.39E+00	2.62E+00	3.08E+00	1.18E+00
XR_233910.4	2.70E+00	1.44E-05	2.18E-05	4.35E+00	2.12E+00	4.35E+00	2.20E-01	5.06E-02
XR_816330.2	2.53E+02	1.44E-05	2.18E-05	2.67E+00	1.42E+00	2.67E+00	1.89E+01	7.07E+00
EPRS	2.35E+03	1.45E-05	2.19E-05	1.41E+00	4.98E-01	1.41E+00	1.44E+02	1.02E+02
LOC104975635	1.06E+03	1.47E-05	2.22E-05	1.35E+00	4.28E-01	1.35E+00	6.36E+01	4.73E+01
KHDC4	1.13E+03	1.47E-05	2.22E-05	1.32E+00	3.95E-01	1.32E+00	6.73E+01	5.12E+01
B4GALT1	8.34E+02	1.48E-05	2.23E-05	1.55E+00	6.31E-01	1.55E+00	5.24E+01	3.39E+01
GSTCD	1.40E+02	1.48E-05	2.23E-05	1.49E+00	5.79E-01	1.49E+00	8.74E+00	5.85E+00
TNS3	3.68E+03	1.50E-05	2.26E-05	1.47E+00	5.58E-01	1.47E+00	2.27E+02	1.54E+02

XR_003037456.1	2.91E+01	1.51E-05	2.27E-05	5.63E+00	2.49E+00	5.63E+00	2.48E+00	4.41E-01
STAT6	1.64E+03	1.54E-05	2.31E-05	1.43E+00	5.17E-01	1.43E+00	1.01E+02	7.02E+01
CIDEB	1.04E+02	1.54E-05	2.31E-05	2.36E+00	1.24E+00	2.36E+00	7.49E+00	3.18E+00
BRD8	1.92E+03	1.57E-05	2.35E-05	1.28E+00	3.54E-01	1.28E+00	1.12E+02	8.80E+01
RHBDF1	9.34E+02	1.57E-05	2.35E-05	1.39E+00	4.75E-01	1.39E+00	5.66E+01	4.07E+01
MSRB3	2.29E+03	1.58E-05	2.36E-05	1.71E+00	7.73E-01	1.71E+00	1.49E+02	8.74E+01
XR_003034852.1	1.38E+01	1.58E-05	2.36E-05	3.07E+00	1.62E+00	3.07E+00	1.06E+00	3.44E-01
IKBKB	6.61E+02	1.59E-05	2.38E-05	1.33E+00	4.10E-01	1.33E+00	3.92E+01	2.95E+01
PLPP3	9.05E+02	1.62E-05	2.42E-05	2.09E+00	1.06E+00	2.09E+00	6.32E+01	3.02E+01
GRIK3	3.91E+01	1.63E-05	2.43E-05	6.46E+00	2.69E+00	6.46E+00	3.42E+00	5.29E-01
WDR19	2.25E+03	1.64E-05	2.44E-05	1.44E+00	5.23E-01	1.44E+00	1.37E+02	9.57E+01
FAM171A1	1.88E+03	1.66E-05	2.47E-05	1.41E+00	4.95E-01	1.41E+00	1.14E+02	8.12E+01
GNG12	2.78E+01	1.67E-05	2.49E-05	1.44E+01	3.85E+00	1.44E+01	2.60E+00	1.81E-01
XR_003032006.1	2.99E+01	1.70E-05	2.53E-05	3.04E+00	1.60E+00	3.04E+00	2.29E+00	7.54E-01
CCDC149	3.60E+02	1.70E-05	2.53E-05	1.50E+00	5.87E-01	1.50E+00	2.24E+01	1.49E+01
XR_003037782.1	8.74E+01	1.74E-05	2.58E-05	2.70E+00	1.43E+00	2.70E+00	6.48E+00	2.40E+00
ALG10	2.15E+02	1.77E-05	2.62E-05	1.47E+00	5.53E-01	1.47E+00	1.33E+01	9.06E+00
XR_003031269.1	3.53E+02	1.81E-05	2.68E-05	2.25E+00	1.17E+00	2.25E+00	2.50E+01	1.11E+01
SLC8A2	2.02E+02	1.81E-05	2.68E-05	2.14E+00	1.10E+00	2.14E+00	1.40E+01	6.56E+00
ATF7	2.84E+03	1.87E-05	2.76E-05	1.65E+00	7.18E-01	1.65E+00	1.82E+02	1.10E+02
ASAP1	9.06E+02	1.89E-05	2.79E-05	1.66E+00	7.28E-01	1.66E+00	5.88E+01	3.55E+01
XR_003030182.1	2.51E+01	1.90E-05	2.81E-05	3.36E+00	1.75E+00	3.36E+00	1.97E+00	5.87E-01
UHRF2	8.50E+02	1.92E-05	2.84E-05	1.31E+00	3.94E-01	1.31E+00	5.04E+01	3.84E+01
CREB3L1	8.18E+02	1.95E-05	2.88E-05	1.59E+00	6.66E-01	1.59E+00	5.21E+01	3.28E+01
TMEM67	2.12E+02	1.98E-05	2.92E-05	1.81E+00	8.53E-01	1.81E+00	1.40E+01	7.77E+00
SMCO3	1.29E+01	2.01E-05	2.96E-05	1.06E+01	3.40E+00	1.06E+01	1.18E+00	1.12E-01
ELF4	1.05E+03	2.05E-05	3.02E-05	1.61E+00	6.91E-01	1.61E+00	6.65E+01	4.12E+01
CASK	1.06E+03	2.08E-05	3.06E-05	1.41E+00	4.97E-01	1.41E+00	6.48E+01	4.59E+01
NIPAL3	1.15E+03	2.12E-05	3.11E-05	1.38E+00	4.61E-01	1.38E+00	6.93E+01	5.04E+01
LOC101905866	1.83E+01	2.13E-05	3.12E-05	4.69E+00	2.23E+00	4.69E+00	1.53E+00	3.27E-01
DTX1	3.90E+02	2.21E-05	3.23E-05	1.75E+00	8.09E-01	1.75E+00	2.56E+01	1.46E+01
LOC782904	2.94E+01	2.21E-05	3.24E-05	1.97E+00	9.77E-01	1.97E+00	2.01E+00	1.02E+00
NR1I3	1.32E+01	2.21E-05	3.24E-05	2.94E+00	1.55E+00	2.94E+00	1.01E+00	3.42E-01

XR_003036089.1	1.87E+01	2.24E-05	3.28E-05	2.36E+00	1.24E+00	2.36E+00	1.34E+00	5.70E-01
CLU	1.34E+04	2.29E-05	3.35E-05	1.76E+00	8.15E-01	1.76E+00	8.86E+02	5.04E+02
SH3BGR	5.11E+02	2.35E-05	3.43E-05	2.44E+00	1.29E+00	2.44E+00	3.71E+01	1.52E+01
XR_003037537.1	1.60E+01	2.35E-05	3.43E-05	6.61E+00	2.73E+00	6.61E+00	1.39E+00	2.10E-01
SUMF1	1.65E+03	2.37E-05	3.46E-05	1.28E+00	3.59E-01	1.28E+00	9.67E+01	7.54E+01
GFRA4	1.08E+02	2.53E-05	3.68E-05	1.79E+00	8.36E-01	1.79E+00	7.19E+00	4.02E+00
LOC790009	3.06E+02	2.53E-05	3.68E-05	1.54E+00	6.28E-01	1.54E+00	1.93E+01	1.25E+01
EPM2AIP1	4.64E+03	2.53E-05	3.68E-05	1.55E+00	6.35E-01	1.55E+00	2.93E+02	1.89E+02
LOC101907802	1.32E+01	2.55E-05	3.70E-05	2.36E+00	1.24E+00	2.36E+00	9.53E-01	4.04E-01
VAMP8	3.76E+02	2.60E-05	3.78E-05	1.36E+00	4.42E-01	1.36E+00	2.26E+01	1.67E+01
TRABD2B	1.22E+02	2.61E-05	3.79E-05	2.27E+00	1.18E+00	2.27E+00	8.73E+00	3.85E+00
ZNF473	4.73E+02	2.62E-05	3.79E-05	1.46E+00	5.42E-01	1.46E+00	2.92E+01	2.00E+01
COL16A1	9.67E+03	2.66E-05	3.85E-05	1.52E+00	6.02E-01	1.52E+00	6.08E+02	4.01E+02
XR_003034240.1	2.80E+01	2.66E-05	3.85E-05	2.66E+00	1.41E+00	2.66E+00	2.08E+00	7.82E-01
N4BP2L1	7.08E+02	2.67E-05	3.86E-05	1.37E+00	4.56E-01	1.37E+00	4.26E+01	3.11E+01
ME3	1.72E+02	2.68E-05	3.87E-05	2.01E+00	1.01E+00	2.01E+00	1.18E+01	5.87E+00
XR_815660.3	3.99E+02	2.70E-05	3.90E-05	1.64E+00	7.09E-01	1.64E+00	2.57E+01	1.57E+01
GLI3	1.26E+03	2.70E-05	3.90E-05	1.90E+00	9.26E-01	1.90E+00	8.58E+01	4.52E+01
XR_003030699.1	1.04E+01	2.71E-05	3.91E-05	4.53E+00	2.18E+00	4.53E+00	8.66E-01	1.91E-01
XR_003030150.1	2.12E+01	2.73E-05	3.94E-05	1.12E+01	3.49E+00	1.12E+01	1.95E+00	1.74E-01
XR_003034633.1	1.12E+01	2.83E-05	4.07E-05	5.57E+00	2.48E+00	5.57E+00	9.54E-01	1.71E-01
XR_003031747.1	2.24E+01	2.85E-05	4.09E-05	2.13E+00	1.09E+00	2.13E+00	1.56E+00	7.35E-01
XR_808699.3	5.28E+01	2.93E-05	4.21E-05	8.50E+00	3.09E+00	8.50E+00	4.77E+00	5.60E-01
AGO1	1.46E+03	2.97E-05	4.26E-05	1.44E+00	5.26E-01	1.44E+00	9.03E+01	6.27E+01
XR_003029617.1	2.14E+02	3.02E-05	4.33E-05	2.24E+00	1.16E+00	2.24E+00	1.53E+01	6.82E+00
FGF1	3.91E+02	3.04E-05	4.35E-05	2.17E+00	1.12E+00	2.17E+00	2.76E+01	1.27E+01
TMC5	5.93E+01	3.04E-05	4.35E-05	6.09E+00	2.61E+00	6.09E+00	5.18E+00	8.51E-01
BBS7	2.14E+02	3.20E-05	4.58E-05	1.48E+00	5.70E-01	1.48E+00	1.32E+01	8.92E+00
XR_003033859.1	3.33E+02	3.21E-05	4.59E-05	1.45E+00	5.33E-01	1.45E+00	2.05E+01	1.42E+01
MAOB	4.06E+02	3.36E-05	4.79E-05	3.25E+00	1.70E+00	3.25E+00	3.18E+01	9.78E+00
EMC1	4.67E+03	3.37E-05	4.81E-05	1.31E+00	3.90E-01	1.31E+00	2.76E+02	2.10E+02
NR_131898.1	3.83E+03	3.43E-05	4.89E-05	1.33E+00	4.11E-01	1.33E+00	2.29E+02	1.72E+02
AFAP1	1.08E+03	3.44E-05	4.89E-05	1.55E+00	6.35E-01	1.55E+00	6.83E+01	4.40E+01

BTBD7	7.84E+02	3.45E-05	4.92E-05	1.59E+00	6.66E-01	1.59E+00	4.99E+01	3.14E+01
XR_003029614.1	4.53E+00	3.47E-05	4.94E-05	5.78E+00	2.53E+00	5.78E+00	3.88E-01	6.71E-02
JAKMIP3	1.62E+02	3.49E-05	4.97E-05	1.94E+00	9.59E-01	1.94E+00	1.09E+01	5.62E+00
XR_003035051.1	1.29E+01	3.50E-05	4.97E-05	2.76E+00	1.46E+00	2.76E+00	9.62E-01	3.49E-01
CLEC5A	7.04E+00	3.50E-05	4.97E-05	1.59E+01	3.99E+00	1.59E+01	6.64E-01	4.19E-02
TNFSF12	4.38E+02	3.60E-05	5.10E-05	1.46E+00	5.43E-01	1.46E+00	2.70E+01	1.85E+01
XR_003031288.1	6.48E+00	3.63E-05	5.15E-05	1.09E+01	3.45E+00	1.09E+01	5.96E-01	5.45E-02
LOC100297540	1.31E+01	3.63E-05	5.15E-05	4.06E+00	2.02E+00	4.06E+00	1.07E+00	2.64E-01
ZNF484	9.64E+02	3.64E-05	5.15E-05	1.37E+00	4.54E-01	1.37E+00	5.83E+01	4.25E+01
RCN3	3.35E+03	3.66E-05	5.17E-05	1.38E+00	4.61E-01	1.38E+00	2.02E+02	1.47E+02
XR_003030578.1	8.60E+02	3.69E-05	5.21E-05	2.01E+00	1.01E+00	2.01E+00	5.88E+01	2.92E+01
LOC101902656	1.70E+01	3.74E-05	5.28E-05	2.27E+00	1.18E+00	2.27E+00	1.21E+00	5.31E-01
XR_807235.3	2.29E+02	3.74E-05	5.28E-05	2.49E+00	1.32E+00	2.49E+00	1.69E+01	6.79E+00
ZCCHC10	1.15E+02	3.80E-05	5.35E-05	1.42E+00	5.05E-01	1.42E+00	6.99E+00	4.93E+00
OSR2	1.17E+03	3.88E-05	5.46E-05	1.68E+00	7.47E-01	1.68E+00	7.60E+01	4.53E+01
XR_003031737.1	4.48E+02	3.96E-05	5.57E-05	1.64E+00	7.09E-01	1.64E+00	2.87E+01	1.76E+01
RHOQ	7.02E+02	4.00E-05	5.62E-05	1.47E+00	5.61E-01	1.47E+00	4.37E+01	2.96E+01
CREBZF	1.51E+03	4.00E-05	5.62E-05	1.37E+00	4.49E-01	1.37E+00	9.06E+01	6.63E+01
LOC787234	7.70E+00	4.04E-05	5.67E-05	2.58E+00	1.37E+00	2.58E+00	5.65E-01	2.19E-01
GPR180	5.01E+01	4.05E-05	5.68E-05	1.89E+00	9.15E-01	1.89E+00	3.34E+00	1.77E+00
XR_001495596.2	1.84E+03	4.11E-05	5.76E-05	1.84E+00	8.78E-01	1.84E+00	1.23E+02	6.72E+01
TMEM68	2.75E+02	4.29E-05	6.01E-05	1.41E+00	4.94E-01	1.41E+00	1.67E+01	1.19E+01
XR_001498246.2	1.29E+01	4.31E-05	6.04E-05	1.62E+01	4.02E+00	1.62E+01	1.21E+00	7.49E-02
PCDH11X	2.38E+01	4.32E-05	6.04E-05	4.83E+00	2.27E+00	4.83E+00	2.00E+00	4.14E-01
EIF4EBP2	2.54E+02	4.35E-05	6.07E-05	3.65E+00	1.87E+00	3.65E+00	2.03E+01	5.55E+00
B4GALT6	3.61E+02	4.36E-05	6.08E-05	1.87E+00	9.00E-01	1.87E+00	2.42E+01	1.30E+01
ZRANB3	2.52E+02	4.37E-05	6.09E-05	1.43E+00	5.21E-01	1.43E+00	1.55E+01	1.08E+01
BLOC1S5	4.47E+02	4.39E-05	6.11E-05	1.31E+00	3.86E-01	1.31E+00	2.64E+01	2.02E+01
MYO1C	3.27E+03	4.39E-05	6.11E-05	1.34E+00	4.20E-01	1.34E+00	1.95E+02	1.46E+02
PUS7L	1.47E+02	4.42E-05	6.15E-05	1.62E+00	6.98E-01	1.62E+00	9.41E+00	5.80E+00
HIGD1B	1.84E+01	4.42E-05	6.15E-05	4.74E+00	2.25E+00	4.74E+00	1.55E+00	3.26E-01
XR_139535.5	1.03E+02	4.51E-05	6.26E-05	1.63E+00	7.02E-01	1.63E+00	6.59E+00	4.05E+00
LOC107131225	1.24E+02	4.68E-05	6.49E-05	1.92E+00	9.38E-01	1.92E+00	8.36E+00	4.36E+00

C1QTNF2	1.37E+02	4.68E-05	6.49E-05	1.88E+00	9.09E-01	1.88E+00	9.24E+00	4.92E+00
XR_003037481.1	3.93E+00	4.85E-05	6.73E-05	8.36E+00	3.06E+00	8.36E+00	3.55E-01	4.24E-02
PRPF18	4.65E+02	4.88E-05	6.76E-05	1.33E+00	4.17E-01	1.33E+00	2.77E+01	2.07E+01
RRAD	1.56E+03	4.91E-05	6.79E-05	1.90E+00	9.29E-01	1.90E+00	1.05E+02	5.54E+01
GCNT4	4.13E+01	4.99E-05	6.91E-05	2.23E+00	1.16E+00	2.23E+00	2.92E+00	1.31E+00
DSC2	4.50E+02	5.01E-05	6.93E-05	1.59E+00	6.71E-01	1.59E+00	2.87E+01	1.80E+01
ATAD2	2.78E+01	5.03E-05	6.94E-05	5.74E+00	2.52E+00	5.74E+00	2.39E+00	4.17E-01
PARM1	1.26E+02	5.04E-05	6.96E-05	2.69E+00	1.43E+00	2.69E+00	9.43E+00	3.50E+00
XR_003034894.1	1.38E+02	5.08E-05	7.00E-05	2.03E+00	1.02E+00	2.03E+00	9.44E+00	4.65E+00
XR_003034801.1	3.38E+00	5.19E-05	7.15E-05	2.64E+01	4.72E+00	2.64E+01	3.26E-01	1.23E-02
XR_003035381.1	1.80E+01	5.20E-05	7.16E-05	2.34E+00	1.23E+00	2.34E+00	1.29E+00	5.50E-01
RNF139	5.85E+02	5.41E-05	7.44E-05	1.22E+00	2.90E-01	1.22E+00	3.36E+01	2.74E+01
GNAO1	2.45E+02	5.44E-05	7.48E-05	3.11E+00	1.64E+00	3.11E+00	1.88E+01	6.05E+00
NTF3	6.27E+01	5.53E-05	7.59E-05	2.64E+00	1.40E+00	2.64E+00	4.64E+00	1.76E+00
BMX	1.40E+01	5.62E-05	7.71E-05	7.18E+00	2.84E+00	7.18E+00	1.24E+00	1.72E-01
QRFPR	4.89E+02	5.77E-05	7.91E-05	2.68E+00	1.42E+00	2.68E+00	3.65E+01	1.36E+01
LOC101903835	2.53E+00	5.87E-05	8.04E-05	4.34E+00	2.12E+00	4.34E+00	2.07E-01	4.76E-02
LTBR	1.50E+03	5.92E-05	8.10E-05	1.37E+00	4.52E-01	1.37E+00	9.07E+01	6.63E+01
IGFBP4	8.61E+03	5.96E-05	8.14E-05	1.51E+00	5.92E-01	1.51E+00	5.37E+02	3.56E+02
MAPK9	5.80E+02	5.96E-05	8.14E-05	1.42E+00	5.02E-01	1.42E+00	3.55E+01	2.50E+01
C1QTNF7	3.25E+02	6.08E-05	8.31E-05	1.86E+00	8.97E-01	1.86E+00	2.17E+01	1.16E+01
C5H12orf45	1.74E+02	6.13E-05	8.37E-05	1.52E+00	6.04E-01	1.52E+00	1.09E+01	7.14E+00
MAVS	8.85E+02	6.16E-05	8.40E-05	1.46E+00	5.50E-01	1.46E+00	5.46E+01	3.73E+01
LOC107131526	2.54E+00	6.21E-05	8.47E-05	1.00E+01	3.32E+00	1.00E+01	2.32E-01	2.32E-02
XR_003035852.1	2.44E+02	6.25E-05	8.51E-05	1.44E+00	5.21E-01	1.44E+00	1.49E+01	1.04E+01
POU6F1	9.17E+02	6.35E-05	8.64E-05	1.61E+00	6.86E-01	1.61E+00	5.82E+01	3.62E+01
LOC107131684	7.11E+01	6.38E-05	8.67E-05	1.83E+00	8.70E-01	1.83E+00	4.76E+00	2.60E+00
PRKAB2	8.88E+02	6.58E-05	8.95E-05	1.37E+00	4.53E-01	1.37E+00	5.37E+01	3.92E+01
XR_001501515.2	1.52E+02	6.62E-05	8.99E-05	1.40E+00	4.88E-01	1.40E+00	9.22E+00	6.58E+00
SLIT3	6.28E+02	6.71E-05	9.10E-05	2.72E+00	1.44E+00	2.72E+00	4.71E+01	1.73E+01
DEK	2.62E+03	6.72E-05	9.11E-05	1.31E+00	3.85E-01	1.31E+00	1.55E+02	1.19E+02
XR_003036885.1	7.44E+00	6.73E-05	9.12E-05	7.99E+00	3.00E+00	7.99E+00	6.64E-01	8.31E-02
SUPT7L	3.60E+02	6.78E-05	9.18E-05	1.27E+00	3.40E-01	1.27E+00	2.10E+01	1.66E+01

ZDHHC1	1.48E+02	6.87E-05	9.28E-05	1.56E+00	6.40E-01	1.56E+00	9.34E+00	5.99E+00
XR_003030358.1	7.55E+02	6.87E-05	9.29E-05	1.93E+00	9.47E-01	1.93E+00	5.10E+01	2.65E+01
XR_003037292.1	3.44E+00	6.89E-05	9.31E-05	7.76E+00	2.96E+00	7.76E+00	3.06E-01	3.94E-02
XR_003033479.1	1.30E+01	6.95E-05	9.37E-05	3.01E+00	1.59E+00	3.01E+00	9.99E-01	3.32E-01
TIA1	3.69E+03	6.95E-05	9.37E-05	1.49E+00	5.74E-01	1.49E+00	2.30E+02	1.54E+02
FZD8	7.77E+02	7.02E-05	9.45E-05	2.44E+00	1.29E+00	2.44E+00	5.63E+01	2.31E+01
XR_003034241.1	2.61E+01	7.13E-05	9.59E-05	2.03E+00	1.02E+00	2.03E+00	1.80E+00	8.86E-01
RGL1	1.20E+03	7.14E-05	9.60E-05	1.72E+00	7.82E-01	1.72E+00	7.78E+01	4.53E+01
NFE2L2	1.62E+03	7.20E-05	9.67E-05	1.29E+00	3.68E-01	1.29E+00	9.52E+01	7.38E+01
PRKCE	4.84E+02	7.35E-05	9.87E-05	1.61E+00	6.83E-01	1.61E+00	3.10E+01	1.93E+01
XR_003034239.1	1.91E+01	7.47E-05	1.00E-04	2.39E+00	1.26E+00	2.39E+00	1.38E+00	5.79E-01
LOC531090	1.15E+02	7.57E-05	1.01E-04	1.14E+01	3.51E+00	1.14E+01	1.07E+01	9.34E-01
EMCN	7.02E+02	7.60E-05	1.02E-04	1.43E+00	5.19E-01	1.43E+00	4.31E+01	3.01E+01
ANKMY2	5.18E+02	7.62E-05	1.02E-04	1.28E+00	3.59E-01	1.28E+00	3.04E+01	2.37E+01
TMEM51	4.60E+02	7.65E-05	1.02E-04	1.83E+00	8.71E-01	1.83E+00	3.05E+01	1.67E+01
BCL2	8.88E+01	7.73E-05	1.03E-04	3.05E+00	1.61E+00	3.05E+00	6.87E+00	2.25E+00
CLK1	3.47E+03	7.93E-05	1.06E-04	1.36E+00	4.48E-01	1.36E+00	2.10E+02	1.54E+02
BCL2L2	1.48E+03	7.99E-05	1.07E-04	1.34E+00	4.23E-01	1.34E+00	8.82E+01	6.58E+01
XR_003037689.1	1.86E+01	8.11E-05	1.08E-04	8.50E+00	3.09E+00	8.50E+00	1.67E+00	1.96E-01
XR_003034464.1	4.31E+00	8.37E-05	1.12E-04	7.67E+00	2.94E+00	7.67E+00	3.84E-01	5.01E-02
SLC16A2	7.92E+02	8.50E-05	1.13E-04	1.80E+00	8.45E-01	1.80E+00	5.24E+01	2.92E+01
TBX18	8.16E+00	8.58E-05	1.14E-04	4.03E+00	2.01E+00	4.03E+00	6.61E-01	1.64E-01
PCNX2	7.67E+01	8.71E-05	1.16E-04	2.04E+00	1.03E+00	2.04E+00	5.30E+00	2.60E+00
ZNF180	1.71E+02	8.92E-05	1.19E-04	1.36E+00	4.45E-01	1.36E+00	1.02E+01	7.53E+00
NR_129534.1	4.74E+01	8.96E-05	1.19E-04	1.66E+00	7.35E-01	1.66E+00	3.08E+00	1.85E+00
TMEM241	3.00E+02	9.01E-05	1.20E-04	1.43E+00	5.12E-01	1.43E+00	1.83E+01	1.28E+01
RBM5	4.83E+03	9.19E-05	1.22E-04	1.32E+00	3.99E-01	1.32E+00	2.86E+02	2.17E+02
MED28	3.76E+02	9.31E-05	1.23E-04	1.51E+00	5.95E-01	1.51E+00	2.34E+01	1.55E+01
XR_815545.3	5.18E+00	9.31E-05	1.23E-04	4.01E+00	2.00E+00	4.01E+00	4.19E-01	1.04E-01
XR_003031871.1	5.53E+01	9.39E-05	1.24E-04	2.33E+00	1.22E+00	2.33E+00	3.94E+00	1.69E+00
IZUMO2	6.88E+00	9.41E-05	1.24E-04	3.50E+00	1.81E+00	3.50E+00	5.43E-01	1.55E-01
ZNF146	1.53E+03	9.54E-05	1.26E-04	1.36E+00	4.40E-01	1.36E+00	9.20E+01	6.78E+01
LOC788150	5.38E+00	9.72E-05	1.28E-04	3.48E+00	1.80E+00	3.48E+00	4.26E-01	1.22E-01

ILDR2	4.27E+02	9.82E-05	1.30E-04	2.02E+00	1.01E+00	2.02E+00	2.94E+01	1.46E+01
GALNT1	1.08E+03	9.89E-05	1.30E-04	1.26E+00	3.36E-01	1.26E+00	6.30E+01	4.99E+01
SPATA13	7.11E+02	9.96E-05	1.31E-04	1.70E+00	7.65E-01	1.70E+00	4.62E+01	2.72E+01
CCDC188	3.05E+02	1.01E-04	1.32E-04	1.75E+00	8.10E-01	1.75E+00	2.00E+01	1.14E+01
ATG5	4.36E+02	1.02E-04	1.34E-04	1.23E+00	3.00E-01	1.23E+00	2.52E+01	2.04E+01
ERGIC2	5.53E+02	1.04E-04	1.37E-04	1.27E+00	3.50E-01	1.27E+00	3.24E+01	2.54E+01
XR_003030605.1	3.17E+00	1.05E-04	1.38E-04	5.46E+00	2.45E+00	5.46E+00	2.70E-01	4.95E-02
ZFP90	5.55E+02	1.06E-04	1.39E-04	1.30E+00	3.80E-01	1.30E+00	3.28E+01	2.52E+01
TFPI	1.60E+02	1.07E-04	1.41E-04	2.16E+00	1.11E+00	2.16E+00	1.12E+01	5.20E+00
ITIH5	1.95E+03	1.07E-04	1.41E-04	3.31E+00	1.73E+00	3.31E+00	1.51E+02	4.56E+01
RBFOX2	4.64E+03	1.09E-04	1.43E-04	1.34E+00	4.18E-01	1.34E+00	2.77E+02	2.08E+02
ROBO4	1.33E+03	1.10E-04	1.43E-04	1.67E+00	7.36E-01	1.67E+00	8.68E+01	5.21E+01
NBDY	7.65E+02	1.15E-04	1.50E-04	1.35E+00	4.30E-01	1.35E+00	4.60E+01	3.42E+01
C5H12orf56	7.04E+01	1.15E-04	1.50E-04	2.34E+00	1.23E+00	2.34E+00	5.10E+00	2.18E+00
XR_001500449.2	2.41E+02	1.17E-04	1.53E-04	1.57E+00	6.54E-01	1.57E+00	1.53E+01	9.70E+00
FECH	5.33E+02	1.18E-04	1.54E-04	1.61E+00	6.87E-01	1.61E+00	3.42E+01	2.13E+01
LOC112442967	2.12E+01	1.18E-04	1.54E-04	2.13E+00	1.09E+00	2.13E+00	1.49E+00	7.01E-01
XR_814213.2	1.86E+01	1.19E-04	1.54E-04	3.19E+00	1.67E+00	3.19E+00	1.43E+00	4.49E-01
XR_236306.4	3.30E+02	1.19E-04	1.55E-04	1.32E+00	4.01E-01	1.32E+00	1.95E+01	1.48E+01
C17H4orf46	2.79E+02	1.19E-04	1.55E-04	1.45E+00	5.38E-01	1.45E+00	1.72E+01	1.18E+01
LOC616948	2.21E+02	1.19E-04	1.55E-04	1.63E+00	7.09E-01	1.63E+00	1.42E+01	8.71E+00
GLUD1	2.29E+03	1.20E-04	1.56E-04	1.30E+00	3.75E-01	1.30E+00	1.35E+02	1.04E+02
XR_003034114.1	3.20E+02	1.20E-04	1.56E-04	1.97E+00	9.76E-01	1.97E+00	2.19E+01	1.11E+01
KIAA1841	2.07E+02	1.21E-04	1.57E-04	1.52E+00	6.01E-01	1.52E+00	1.29E+01	8.51E+00
PMP22	2.95E+03	1.23E-04	1.60E-04	1.61E+00	6.88E-01	1.61E+00	1.88E+02	1.17E+02
DNAJB4	4.97E+02	1.26E-04	1.64E-04	1.38E+00	4.62E-01	1.38E+00	3.01E+01	2.18E+01
XR_805374.3	2.87E+02	1.28E-04	1.66E-04	1.35E+00	4.35E-01	1.35E+00	1.72E+01	1.27E+01
XR_003031981.1	1.96E+02	1.30E-04	1.68E-04	1.70E+00	7.69E-01	1.70E+00	1.28E+01	7.51E+00
RCSD1	4.09E+02	1.31E-04	1.69E-04	1.50E+00	5.82E-01	1.50E+00	2.54E+01	1.70E+01
XR_003029449.1	4.46E+00	1.36E-04	1.76E-04	2.79E+00	1.48E+00	2.79E+00	3.34E-01	1.20E-01
LOC101902154	1.74E+01	1.38E-04	1.77E-04	2.11E+00	1.07E+00	2.11E+00	1.20E+00	5.69E-01
LOC539893	8.31E+01	1.39E-04	1.79E-04	1.50E+00	5.89E-01	1.50E+00	5.20E+00	3.46E+00
EXT2	2.05E+03	1.39E-04	1.79E-04	1.27E+00	3.47E-01	1.27E+00	1.19E+02	9.40E+01

CTSB	6.92E+03	1.40E-04	1.80E-04	1.35E+00	4.38E-01	1.35E+00	4.15E+02	3.06E+02
DISC1	2.44E+02	1.43E-04	1.84E-04	1.51E+00	5.98E-01	1.51E+00	1.52E+01	1.01E+01
LOC112448743	7.20E+01	1.44E-04	1.85E-04	2.32E+00	1.21E+00	2.32E+00	5.17E+00	2.23E+00
XR_808566.3	7.60E+00	1.44E-04	1.85E-04	3.07E+00	1.62E+00	3.07E+00	5.85E-01	1.91E-01
LOC100140039	2.58E+01	1.46E-04	1.88E-04	2.22E+00	1.15E+00	2.22E+00	1.80E+00	8.13E-01
INPP4A	1.22E+03	1.49E-04	1.92E-04	1.43E+00	5.17E-01	1.43E+00	7.47E+01	5.22E+01
MFSD8	2.21E+02	1.50E-04	1.92E-04	1.36E+00	4.43E-01	1.36E+00	1.33E+01	9.76E+00
XR_003035435.1	8.96E+00	1.50E-04	1.92E-04	2.57E+00	1.36E+00	2.57E+00	6.59E-01	2.57E-01
XR_003036889.1	4.08E+01	1.52E-04	1.95E-04	1.79E+00	8.41E-01	1.79E+00	2.72E+00	1.52E+00
IPMK	8.91E+01	1.53E-04	1.95E-04	1.52E+00	6.06E-01	1.52E+00	5.61E+00	3.69E+00
XR_003033130.1	2.17E+02	1.53E-04	1.95E-04	2.94E+00	1.55E+00	2.94E+00	1.63E+01	5.55E+00
TMEM106B	9.77E+02	1.53E-04	1.96E-04	1.36E+00	4.44E-01	1.36E+00	5.88E+01	4.32E+01
PDLIM7	4.37E+03	1.54E-04	1.96E-04	1.36E+00	4.45E-01	1.36E+00	2.63E+02	1.93E+02
NFX1	7.20E+02	1.62E-04	2.07E-04	1.40E+00	4.82E-01	1.40E+00	4.35E+01	3.12E+01
FAM69A	9.51E+01	1.65E-04	2.11E-04	1.78E+00	8.33E-01	1.78E+00	6.29E+00	3.53E+00
XR_003034390.1	3.39E+02	1.65E-04	2.11E-04	1.61E+00	6.90E-01	1.61E+00	2.16E+01	1.34E+01
XR_808734.3	1.94E+00	1.66E-04	2.11E-04	1.67E+02	7.38E+00	1.67E+02	1.93E-01	1.16E-03
XR_003031268.1	2.11E+02	1.67E-04	2.12E-04	1.80E+00	8.48E-01	1.80E+00	1.40E+01	7.76E+00
KIF27	3.31E+02	1.67E-04	2.13E-04	1.75E+00	8.08E-01	1.75E+00	2.18E+01	1.24E+01
DIO2	9.28E+00	1.68E-04	2.14E-04	4.17E+00	2.06E+00	4.17E+00	7.61E-01	1.83E-01
PGRMC2	1.23E+03	1.70E-04	2.16E-04	1.19E+00	2.57E-01	1.19E+00	6.99E+01	5.85E+01
XR_001495102.2	7.79E+01	1.71E-04	2.17E-04	1.94E+00	9.56E-01	1.94E+00	5.29E+00	2.73E+00
ZNF184	6.26E+02	1.73E-04	2.19E-04	1.41E+00	4.94E-01	1.41E+00	3.80E+01	2.70E+01
RC3H1	8.87E+02	1.74E-04	2.20E-04	1.30E+00	3.81E-01	1.30E+00	5.23E+01	4.01E+01
NUDT12	1.48E+02	1.77E-04	2.24E-04	1.57E+00	6.49E-01	1.57E+00	9.39E+00	5.99E+00
XR_003029746.1	2.47E+01	1.79E-04	2.27E-04	1.77E+00	8.27E-01	1.77E+00	1.62E+00	9.12E-01
RPP14	1.04E+03	1.81E-04	2.29E-04	1.23E+00	2.99E-01	1.23E+00	6.01E+01	4.88E+01
XR_003036487.1	2.93E+00	1.85E-04	2.33E-04	2.84E+00	1.50E+00	2.84E+00	2.17E-01	7.64E-02
XR_003036126.1	2.94E+01	1.86E-04	2.34E-04	1.60E+00	6.82E-01	1.60E+00	1.88E+00	1.17E+00
EPHA3	1.80E+01	1.86E-04	2.34E-04	2.35E+01	4.56E+00	2.35E+01	1.74E+00	7.38E-02
ZNF326	9.53E+02	1.86E-04	2.34E-04	1.29E+00	3.65E-01	1.29E+00	5.60E+01	4.35E+01
MCAM	1.44E+03	1.87E-04	2.36E-04	1.64E+00	7.16E-01	1.64E+00	9.28E+01	5.65E+01
XR_003032351.1	7.13E+01	1.89E-04	2.38E-04	2.96E+00	1.57E+00	2.96E+00	5.45E+00	1.84E+00

XR_003034394.1	8.43E+01	1.90E-04	2.38E-04	1.77E+00	8.26E-01	1.77E+00	5.56E+00	3.14E+00
PLD2	1.55E+03	1.91E-04	2.41E-04	1.41E+00	4.99E-01	1.41E+00	9.45E+01	6.69E+01
XR_003035894.1	1.66E+00	1.92E-04	2.42E-04	7.00E+00	2.81E+00	7.00E+00	1.46E-01	2.08E-02
XR_003034580.1	1.11E+02	1.93E-04	2.42E-04	2.06E+00	1.04E+00	2.06E+00	7.63E+00	3.71E+00
LOC104974678	8.57E+00	1.96E-04	2.46E-04	2.43E+00	1.28E+00	2.43E+00	6.23E-01	2.56E-01
COL6A5	2.03E+02	1.96E-04	2.46E-04	2.34E+00	1.23E+00	2.34E+00	1.46E+01	6.23E+00
FCHO2	3.27E+01	1.97E-04	2.47E-04	9.68E+00	3.27E+00	9.68E+00	2.98E+00	3.08E-01
SEMA3B	1.37E+03	1.98E-04	2.48E-04	1.49E+00	5.76E-01	1.49E+00	8.56E+01	5.74E+01
SCO1	9.68E+02	2.01E-04	2.52E-04	1.22E+00	2.87E-01	1.22E+00	5.56E+01	4.55E+01
XR_003030467.1	1.24E+01	2.04E-04	2.54E-04	1.68E+01	4.07E+00	1.68E+01	1.18E+00	7.04E-02
XR_003035030.1	5.47E+00	2.07E-04	2.59E-04	1.87E+01	4.22E+00	1.87E+01	5.22E-01	2.79E-02
PRR16	8.46E+01	2.11E-04	2.63E-04	8.45E+00	3.08E+00	8.45E+00	7.61E+00	9.01E-01
LRRTM3	9.87E+00	2.15E-04	2.68E-04	2.80E+00	1.49E+00	2.80E+00	7.38E-01	2.63E-01
SYNE2	3.55E+03	2.15E-04	2.68E-04	1.47E+00	5.52E-01	1.47E+00	2.19E+02	1.49E+02
MSI2	1.91E+03	2.16E-04	2.68E-04	1.52E+00	6.08E-01	1.52E+00	1.20E+02	7.89E+01
RBM39	5.66E+03	2.16E-04	2.69E-04	1.22E+00	2.83E-01	1.22E+00	3.26E+02	2.68E+02
XR_806610.3	1.04E+03	2.17E-04	2.70E-04	1.35E+00	4.36E-01	1.35E+00	6.27E+01	4.64E+01
XR_003034762.1	1.07E+01	2.17E-04	2.70E-04	2.93E+00	1.55E+00	2.93E+00	8.20E-01	2.80E-01
EHD3	5.36E+02	2.17E-04	2.70E-04	1.69E+00	7.54E-01	1.69E+00	3.48E+01	2.06E+01
SSBP2	1.37E+03	2.18E-04	2.70E-04	1.36E+00	4.41E-01	1.36E+00	8.20E+01	6.04E+01
XR_003036088.1	2.19E+01	2.23E-04	2.77E-04	5.42E+00	2.44E+00	5.42E+00	1.86E+00	3.43E-01
PIK3R6	1.32E+02	2.24E-04	2.77E-04	1.47E+00	5.52E-01	1.47E+00	8.11E+00	5.53E+00
PLPP6	2.80E+01	2.28E-04	2.82E-04	1.85E+00	8.86E-01	1.85E+00	1.88E+00	1.02E+00
LOC107132868	2.01E+00	2.28E-04	2.82E-04	2.23E+01	4.48E+00	2.23E+01	1.92E-01	8.61E-03
NFYA	5.59E+02	2.30E-04	2.84E-04	1.35E+00	4.38E-01	1.35E+00	3.35E+01	2.48E+01
CCL1	5.02E+00	2.31E-04	2.86E-04	9.65E+00	3.27E+00	9.65E+00	4.58E-01	4.75E-02
MTCP1	6.64E+01	2.32E-04	2.86E-04	1.49E+00	5.79E-01	1.49E+00	4.13E+00	2.76E+00
GDF10	4.47E+02	2.41E-04	2.98E-04	2.90E+00	1.54E+00	2.90E+00	3.40E+01	1.17E+01
XR_003036746.1	3.72E+00	2.43E-04	3.00E-04	9.72E+00	3.28E+00	9.72E+00	3.39E-01	3.49E-02
LOC107131940	7.22E+00	2.44E-04	3.01E-04	2.26E+00	1.18E+00	2.26E+00	5.13E-01	2.27E-01
XR_003036365.1	1.62E+01	2.51E-04	3.09E-04	2.30E+00	1.20E+00	2.30E+00	1.16E+00	5.04E-01
CRISPLD1	5.56E+02	2.51E-04	3.09E-04	1.62E+00	6.98E-01	1.62E+00	3.55E+01	2.19E+01
KYNU	5.40E+00	2.51E-04	3.09E-04	2.53E+00	1.34E+00	2.53E+00	3.94E-01	1.56E-01

XR_003032631.1	5.79E+01	2.56E-04	3.15E-04	2.08E+00	1.06E+00	2.08E+00	4.01E+00	1.93E+00
LRP1	2.29E+04	2.59E-04	3.18E-04	1.61E+00	6.89E-01	1.61E+00	1.46E+03	9.08E+02
XR_003037488.1	3.87E+00	2.63E-04	3.23E-04	5.85E+00	2.55E+00	5.85E+00	3.36E-01	5.74E-02
LOC104972581	7.27E+00	2.64E-04	3.23E-04	3.96E+00	1.98E+00	3.96E+00	5.93E-01	1.50E-01
XR_001494799.2	7.58E+01	2.64E-04	3.23E-04	1.73E+00	7.93E-01	1.73E+00	4.95E+00	2.85E+00
IFNGR1	1.19E+03	2.84E-04	3.48E-04	1.35E+00	4.36E-01	1.35E+00	7.10E+01	5.25E+01
SNED1	9.64E+02	2.84E-04	3.48E-04	1.91E+00	9.34E-01	1.91E+00	6.49E+01	3.40E+01
XR_001500471.2	7.28E+00	2.92E-04	3.57E-04	3.35E+00	1.74E+00	3.35E+00	5.69E-01	1.70E-01
XR_001494474.2	6.28E+01	2.92E-04	3.57E-04	1.47E+00	5.53E-01	1.47E+00	3.87E+00	2.64E+00
XR_003031034.1	6.03E+00	2.96E-04	3.62E-04	1.22E+01	3.60E+00	1.22E+01	5.60E-01	4.61E-02
LOC100298573	6.83E+00	2.98E-04	3.64E-04	5.06E+00	2.34E+00	5.06E+00	5.73E-01	1.13E-01
XR_003033297.1	6.20E+01	3.03E-04	3.69E-04	1.58E+00	6.61E-01	1.58E+00	3.94E+00	2.49E+00
SLC25A45	2.98E+01	3.21E-04	3.91E-04	1.95E+00	9.61E-01	1.95E+00	2.02E+00	1.04E+00
XR_003030781.1	1.33E+01	3.23E-04	3.94E-04	1.51E+01	3.92E+00	1.51E+01	1.25E+00	8.30E-02
C11H2orf42	2.91E+02	3.23E-04	3.94E-04	1.32E+00	4.01E-01	1.32E+00	1.73E+01	1.31E+01
MYOZ3	4.02E+01	3.33E-04	4.05E-04	1.89E+00	9.21E-01	1.89E+00	2.72E+00	1.44E+00
XR_003037518.1	5.36E+01	3.48E-04	4.22E-04	2.19E+00	1.13E+00	2.19E+00	3.77E+00	1.72E+00
XR_003037599.1	1.08E+02	3.48E-04	4.22E-04	2.23E+00	1.15E+00	2.23E+00	7.69E+00	3.45E+00
NFIX	3.56E+03	3.48E-04	4.22E-04	1.67E+00	7.39E-01	1.67E+00	2.28E+02	1.37E+02
SLC18B1	2.57E+02	3.51E-04	4.26E-04	1.34E+00	4.25E-01	1.34E+00	1.53E+01	1.14E+01
XR_003033394.1	4.99E+01	3.51E-04	4.26E-04	1.56E+00	6.38E-01	1.56E+00	3.14E+00	2.02E+00
LOC100140085	1.21E+01	3.70E-04	4.49E-04	2.15E+00	1.10E+00	2.15E+00	8.40E-01	3.91E-01
ZNF260	7.05E+02	3.73E-04	4.51E-04	1.29E+00	3.64E-01	1.29E+00	4.15E+01	3.23E+01
LOC112446390	4.05E+01	3.87E-04	4.68E-04	1.75E+00	8.10E-01	1.75E+00	2.65E+00	1.51E+00
SEC62	2.65E+03	3.93E-04	4.76E-04	1.24E+00	3.16E-01	1.24E+00	1.54E+02	1.23E+02
CAV3	5.47E+00	3.99E-04	4.83E-04	1.75E+01	4.13E+00	1.75E+01	5.19E-01	2.96E-02
XR_003029487.1	6.59E+01	4.00E-04	4.83E-04	1.52E+00	6.01E-01	1.52E+00	4.12E+00	2.72E+00
LRP3	4.36E+02	4.16E-04	5.02E-04	1.36E+00	4.40E-01	1.36E+00	2.61E+01	1.92E+01
ZBTB21	3.93E+02	4.17E-04	5.03E-04	1.75E+00	8.11E-01	1.75E+00	2.58E+01	1.47E+01
XR_236157.4	8.28E+00	4.27E-04	5.15E-04	2.70E+00	1.43E+00	2.70E+00	6.14E-01	2.27E-01
XR_236815.3	4.28E+01	4.28E-04	5.15E-04	1.61E+00	6.87E-01	1.61E+00	2.74E+00	1.70E+00
MARVELD1	3.35E+03	4.44E-04	5.34E-04	1.25E+00	3.18E-01	1.25E+00	1.94E+02	1.56E+02
RASL11A	1.45E+02	4.48E-04	5.38E-04	2.08E+00	1.06E+00	2.08E+00	1.02E+01	4.89E+00

GK	5.80E+01	4.56E-04	5.47E-04	1.46E+00	5.49E-01	1.46E+00	3.57E+00	2.44E+00
LOC104970812	1.14E+01	4.56E-04	5.47E-04	2.07E+00	1.05E+00	2.07E+00	7.86E-01	3.79E-01
USP47	1.83E+03	4.63E-04	5.56E-04	1.27E+00	3.48E-01	1.27E+00	1.07E+02	8.44E+01
COL27A1	9.42E+03	4.71E-04	5.65E-04	1.53E+00	6.12E-01	1.53E+00	5.92E+02	3.87E+02
XR_001500965.2	4.33E+01	4.73E-04	5.67E-04	3.93E+00	1.97E+00	3.93E+00	3.51E+00	8.93E-01
XR_003036642.1	6.98E+01	4.76E-04	5.71E-04	1.74E+00	8.02E-01	1.74E+00	4.61E+00	2.65E+00
TNFSF15	4.58E+00	4.80E-04	5.74E-04	1.31E+01	3.71E+00	1.31E+01	4.27E-01	3.26E-02
GPR137B	1.10E+02	4.88E-04	5.83E-04	1.52E+00	6.02E-01	1.52E+00	6.87E+00	4.53E+00
LOC101903205	4.52E+02	4.89E-04	5.84E-04	1.46E+00	5.49E-01	1.46E+00	2.80E+01	1.91E+01
XR_003033186.1	7.93E+00	4.95E-04	5.91E-04	1.28E+01	3.68E+00	1.28E+01	7.43E-01	5.79E-02
LOC112444193	3.92E+00	5.00E-04	5.96E-04	5.54E+00	2.47E+00	5.54E+00	3.33E-01	6.02E-02
TMEM154	2.09E+02	5.12E-04	6.11E-04	1.90E+00	9.23E-01	1.90E+00	1.41E+01	7.45E+00
XR_003030684.1	1.11E+02	5.13E-04	6.11E-04	1.49E+00	5.73E-01	1.49E+00	6.87E+00	4.61E+00
XR_239312.4	2.10E+02	5.18E-04	6.17E-04	1.48E+00	5.61E-01	1.48E+00	1.30E+01	8.79E+00
XR_003029544.1	6.21E+00	5.23E-04	6.22E-04	2.97E+00	1.57E+00	2.97E+00	4.75E-01	1.60E-01
YAF2	3.15E+02	5.24E-04	6.23E-04	1.31E+00	3.93E-01	1.31E+00	1.87E+01	1.42E+01
RINT1	4.24E+02	5.28E-04	6.28E-04	1.25E+00	3.17E-01	1.25E+00	2.47E+01	1.98E+01
PKD1	6.03E+03	5.36E-04	6.36E-04	1.42E+00	5.08E-01	1.42E+00	3.68E+02	2.59E+02
LYZ2	4.45E+00	5.50E-04	6.53E-04	4.79E+00	2.26E+00	4.79E+00	3.70E-01	7.73E-02
SORCS2	7.35E+02	5.54E-04	6.58E-04	1.82E+00	8.61E-01	1.82E+00	4.87E+01	2.68E+01
XR_805792.3	2.07E+01	5.66E-04	6.71E-04	1.36E+01	3.77E+00	1.36E+01	1.94E+00	1.42E-01
SLC25A41	1.79E+01	5.67E-04	6.72E-04	1.84E+00	8.79E-01	1.84E+00	1.19E+00	6.48E-01
AMMECR1	4.73E+01	5.69E-04	6.74E-04	1.36E+01	3.76E+00	1.36E+01	4.43E+00	3.26E-01
LOC100848799	5.36E+01	5.84E-04	6.91E-04	1.76E+00	8.18E-01	1.76E+00	3.54E+00	2.01E+00
FAM46A	9.98E+02	5.88E-04	6.96E-04	1.89E+00	9.17E-01	1.89E+00	6.77E+01	3.58E+01
LST1	8.04E+01	6.08E-04	7.18E-04	1.81E+00	8.55E-01	1.81E+00	5.32E+00	2.94E+00
XR_003037770.1	4.47E+00	6.12E-04	7.22E-04	5.19E+00	2.37E+00	5.19E+00	3.76E-01	7.26E-02
CTCF	2.26E+03	6.12E-04	7.23E-04	1.21E+00	2.76E-01	1.21E+00	1.29E+02	1.07E+02
ZYG11B	7.10E+02	6.54E-04	7.71E-04	1.25E+00	3.23E-01	1.25E+00	4.14E+01	3.31E+01
CCDC71L	4.32E+02	6.67E-04	7.86E-04	1.52E+00	6.03E-01	1.52E+00	2.70E+01	1.78E+01
XR_003030810.1	1.83E+02	6.78E-04	7.98E-04	1.46E+00	5.41E-01	1.46E+00	1.13E+01	7.79E+00
XR_001494366.2	1.16E+01	6.78E-04	7.99E-04	2.70E+00	1.43E+00	2.70E+00	8.63E-01	3.20E-01
CA4	4.53E+01	6.96E-04	8.19E-04	1.08E+01	3.44E+00	1.08E+01	4.20E+00	3.88E-01

KCTD18	2.86E+02	7.01E-04	8.24E-04	1.29E+00	3.64E-01	1.29E+00	1.68E+01	1.30E+01
TTC14	8.21E+02	7.01E-04	8.24E-04	1.27E+00	3.46E-01	1.27E+00	4.81E+01	3.78E+01
XR_815663.3	9.06E+00	7.17E-04	8.42E-04	1.16E+01	3.54E+00	1.16E+01	8.40E-01	7.22E-02
XR_003030780.1	4.31E+01	7.32E-04	8.59E-04	1.95E+00	9.65E-01	1.95E+00	2.95E+00	1.51E+00
XR_003034594.1	9.32E+01	7.43E-04	8.72E-04	1.47E+00	5.57E-01	1.47E+00	5.77E+00	3.92E+00
XR_003029776.1	1.21E+01	7.48E-04	8.77E-04	3.36E+00	1.75E+00	3.36E+00	9.49E-01	2.83E-01
XR_236050.4	1.82E+02	7.63E-04	8.94E-04	1.37E+00	4.53E-01	1.37E+00	1.09E+01	7.98E+00
GAPVD1	5.98E+02	7.65E-04	8.95E-04	1.37E+00	4.53E-01	1.37E+00	3.61E+01	2.63E+01
XR_003029526.1	4.13E+01	7.87E-04	9.20E-04	2.03E+00	1.02E+00	2.03E+00	2.83E+00	1.39E+00
XR_003029816.1	8.83E+00	7.91E-04	9.24E-04	7.01E+00	2.81E+00	7.01E+00	7.84E-01	1.12E-01
LOC112443452	2.60E+03	8.09E-04	9.45E-04	1.28E+00	3.58E-01	1.28E+00	1.53E+02	1.19E+02
MTAP	4.64E+02	8.18E-04	9.54E-04	1.21E+00	2.72E-01	1.21E+00	2.66E+01	2.20E+01
XR_003035399.1	3.56E+00	8.25E-04	9.62E-04	7.03E+00	2.81E+00	7.03E+00	3.13E-01	4.45E-02
XR_003030885.1	1.29E+01	8.34E-04	9.73E-04	2.42E+00	1.27E+00	2.42E+00	9.47E-01	3.91E-01
MEIS3	1.25E+03	8.37E-04	9.75E-04	1.43E+00	5.15E-01	1.43E+00	7.65E+01	5.35E+01
LOC101905723	6.63E+00	8.44E-04	9.82E-04	2.52E+00	1.33E+00	2.52E+00	4.85E-01	1.92E-01
XR_003034308.1	1.99E+00	8.51E-04	9.90E-04	6.24E+00	2.64E+00	6.24E+00	1.71E-01	2.75E-02
XR_003030580.1	1.29E+01	8.52E-04	9.91E-04	7.40E+00	2.89E+00	7.40E+00	1.14E+00	1.55E-01
CAPRIN1	6.20E+03	8.61E-04	1.00E-03	1.24E+00	3.14E-01	1.24E+00	3.60E+02	2.90E+02
XR_003034186.1	2.25E+00	8.67E-04	1.01E-03	7.81E+00	2.97E+00	7.81E+00	2.03E-01	2.59E-02
ACAN	1.08E+02	8.73E-04	1.01E-03	3.30E+00	1.72E+00	3.30E+00	8.39E+00	2.54E+00
XR_814919.2	1.75E+02	8.79E-04	1.02E-03	2.32E+00	1.21E+00	2.32E+00	1.25E+01	5.41E+00
ZBED5	9.94E+02	8.96E-04	1.04E-03	1.26E+00	3.38E-01	1.26E+00	5.80E+01	4.59E+01
EPHB3	9.32E+02	9.07E-04	1.05E-03	1.60E+00	6.76E-01	1.60E+00	5.98E+01	3.74E+01
METAP1	2.77E+02	9.15E-04	1.06E-03	1.63E+00	7.02E-01	1.63E+00	1.78E+01	1.09E+01
CYSLTR2	9.93E+00	9.60E-04	1.11E-03	7.72E+00	2.95E+00	7.72E+00	8.92E-01	1.16E-01
XR_003030074.1	6.17E+01	9.66E-04	1.12E-03	3.20E+00	1.68E+00	3.20E+00	4.71E+00	1.47E+00
LOC789799	1.29E+00	9.76E-04	1.13E-03	1.85E+01	4.21E+00	1.85E+01	1.23E-01	6.64E-03
HGF	1.59E+02	9.93E-04	1.15E-03	2.57E+00	1.36E+00	2.57E+00	1.17E+01	4.57E+00
GTF2IRD2	9.89E+02	1.01E-03	1.17E-03	1.20E+00	2.68E-01	1.20E+00	5.67E+01	4.71E+01
LOC100849050	2.75E+01	1.04E-03	1.19E-03	1.59E+00	6.65E-01	1.59E+00	1.75E+00	1.10E+00
XR_240630.4	3.38E+01	1.05E-03	1.21E-03	3.66E+00	1.87E+00	3.66E+00	2.72E+00	7.43E-01
CPNE6	7.76E+00	1.06E-03	1.22E-03	4.91E+00	2.30E+00	4.91E+00	6.54E-01	1.33E-01

KIAA1614	7.00E+02	1.08E-03	1.24E-03	1.35E+00	4.35E-01	1.35E+00	4.19E+01	3.10E+01
ENOSF1	2.11E+02	1.08E-03	1.24E-03	1.29E+00	3.64E-01	1.29E+00	1.23E+01	9.55E+00
XR_003036622.1	2.69E+01	1.08E-03	1.24E-03	9.59E+00	3.26E+00	9.59E+00	2.45E+00	2.55E-01
TOP2B	3.60E+03	1.09E-03	1.25E-03	1.28E+00	3.57E-01	1.28E+00	2.12E+02	1.65E+02
XR_003029837.1	3.86E+00	1.10E-03	1.26E-03	3.16E+00	1.66E+00	3.16E+00	3.01E-01	9.53E-02
UNKL	7.60E+02	1.11E-03	1.28E-03	1.44E+00	5.31E-01	1.44E+00	4.66E+01	3.22E+01
LOC112447863	1.31E+00	1.13E-03	1.30E-03	1.26E+01	3.66E+00	1.26E+01	1.21E-01	9.62E-03
XR_813886.3	1.25E+01	1.13E-03	1.30E-03	4.40E+00	2.14E+00	4.40E+00	1.03E+00	2.34E-01
RNF14	6.44E+02	1.17E-03	1.34E-03	1.22E+00	2.85E-01	1.22E+00	3.68E+01	3.02E+01
XR_003029580.1	1.09E+02	1.17E-03	1.34E-03	8.35E+00	3.06E+00	8.35E+00	9.82E+00	1.18E+00
USP54	7.50E+02	1.20E-03	1.37E-03	1.26E+00	3.32E-01	1.26E+00	4.37E+01	3.47E+01
XR_003037505.1	3.83E+01	1.22E-03	1.39E-03	2.93E+00	1.55E+00	2.93E+00	2.93E+00	9.98E-01
LOC112444775	2.90E+01	1.24E-03	1.41E-03	1.74E+00	7.95E-01	1.74E+00	1.90E+00	1.10E+00
SMUG1	1.19E+03	1.24E-03	1.42E-03	1.20E+00	2.62E-01	1.20E+00	6.81E+01	5.68E+01
NRAS	9.42E+02	1.26E-03	1.44E-03	1.22E+00	2.84E-01	1.22E+00	5.42E+01	4.45E+01
MYH9	1.66E+04	1.27E-03	1.45E-03	1.28E+00	3.52E-01	1.28E+00	9.72E+02	7.61E+02
XR_003037516.1	6.82E+01	1.28E-03	1.46E-03	1.86E+00	8.97E-01	1.86E+00	4.55E+00	2.44E+00
RBM43	5.99E+02	1.37E-03	1.56E-03	1.34E+00	4.20E-01	1.34E+00	3.57E+01	2.67E+01
GPR141	2.52E+00	1.37E-03	1.56E-03	5.31E+00	2.41E+00	5.31E+00	2.14E-01	4.03E-02
XR_003030194.1	1.10E+02	1.38E-03	1.56E-03	1.67E+00	7.41E-01	1.67E+00	7.09E+00	4.24E+00
NDRG4	5.14E+02	1.42E-03	1.61E-03	1.70E+00	7.63E-01	1.70E+00	3.36E+01	1.98E+01
XR_236092.4	1.54E+03	1.43E-03	1.62E-03	1.25E+00	3.20E-01	1.25E+00	8.91E+01	7.14E+01
BCLAF1	2.47E+03	1.43E-03	1.62E-03	1.28E+00	3.58E-01	1.28E+00	1.45E+02	1.13E+02
ZBED3	3.57E+03	1.46E-03	1.65E-03	1.65E+00	7.24E-01	1.65E+00	2.30E+02	1.39E+02
FUT10	1.44E+02	1.46E-03	1.66E-03	1.35E+00	4.31E-01	1.35E+00	8.59E+00	6.37E+00
XR_003030043.1	2.09E+01	1.47E-03	1.67E-03	2.13E+00	1.09E+00	2.13E+00	1.45E+00	6.83E-01
XR_805839.3	1.09E+01	1.48E-03	1.67E-03	2.00E+00	9.98E-01	2.00E+00	7.47E-01	3.74E-01
LOC783920	4.04E+01	1.55E-03	1.75E-03	2.90E+00	1.54E+00	2.90E+00	3.01E+00	1.04E+00
XR_808417.3	4.14E+00	1.56E-03	1.76E-03	2.24E+00	1.17E+00	2.24E+00	2.93E-01	1.30E-01
XR_001495436.2	2.48E+00	1.57E-03	1.77E-03	5.71E+00	2.51E+00	5.71E+00	2.13E-01	3.73E-02
SMIM33	2.10E+01	1.57E-03	1.78E-03	1.94E+00	9.59E-01	1.94E+00	1.44E+00	7.38E-01
XR_236236.4	4.30E+02	1.57E-03	1.78E-03	1.25E+00	3.20E-01	1.25E+00	2.50E+01	2.00E+01
GAS2	7.27E+01	1.62E-03	1.82E-03	1.56E+00	6.43E-01	1.56E+00	4.58E+00	2.93E+00

SYNJ2BP	3.76E+02	1.65E-03	1.86E-03	1.18E+00	2.44E-01	1.18E+00	2.14E+01	1.80E+01
LOC112445190	1.54E+01	1.66E-03	1.87E-03	2.36E+00	1.24E+00	2.36E+00	1.10E+00	4.67E-01
SERINC1	2.64E+03	1.72E-03	1.93E-03	1.21E+00	2.72E-01	1.21E+00	1.51E+02	1.25E+02
AMN1	6.41E+02	1.76E-03	1.98E-03	1.34E+00	4.24E-01	1.34E+00	3.83E+01	2.85E+01
FBXW7	7.93E+02	1.76E-03	1.98E-03	1.37E+00	4.52E-01	1.37E+00	4.75E+01	3.47E+01
XR_813457.3	8.85E+01	1.77E-03	1.99E-03	2.17E+00	1.12E+00	2.17E+00	6.21E+00	2.86E+00
TTLL3	5.84E+02	1.78E-03	2.00E-03	1.28E+00	3.61E-01	1.28E+00	3.42E+01	2.66E+01
XR_003032003.1	1.41E+01	1.85E-03	2.07E-03	2.39E+00	1.26E+00	2.39E+00	1.02E+00	4.27E-01
TTBK2	5.65E+02	1.85E-03	2.08E-03	1.26E+00	3.30E-01	1.26E+00	3.29E+01	2.61E+01
LOC519309	1.38E+01	1.87E-03	2.10E-03	1.79E+00	8.41E-01	1.79E+00	9.15E-01	5.11E-01
XR_233395.4	3.47E+01	1.91E-03	2.14E-03	1.92E+00	9.41E-01	1.92E+00	2.35E+00	1.23E+00
XR_240511.4	2.94E+01	1.92E-03	2.15E-03	1.97E+00	9.77E-01	1.97E+00	2.01E+00	1.02E+00
RGS18	6.69E+00	1.94E-03	2.17E-03	2.11E+00	1.08E+00	2.11E+00	4.66E-01	2.21E-01
FAM133B	4.39E+02	1.97E-03	2.20E-03	1.21E+00	2.72E-01	1.21E+00	2.51E+01	2.08E+01
SH3PXD2B	3.08E+03	1.99E-03	2.23E-03	1.35E+00	4.33E-01	1.35E+00	1.85E+02	1.37E+02
NEU3	4.25E+02	2.02E-03	2.25E-03	1.27E+00	3.46E-01	1.27E+00	2.47E+01	1.94E+01
DDX42	2.97E+03	2.09E-03	2.33E-03	1.15E+00	1.99E-01	1.15E+00	1.66E+02	1.45E+02
LOC112442704	1.04E+01	2.11E-03	2.35E-03	6.75E+00	2.76E+00	6.75E+00	9.16E-01	1.36E-01
CEBPZ	1.76E+03	2.13E-03	2.38E-03	1.16E+00	2.12E-01	1.16E+00	9.94E+01	8.59E+01
LOC101907083	5.01E+02	2.14E-03	2.39E-03	1.24E+00	3.14E-01	1.24E+00	2.91E+01	2.34E+01
PLXND1	4.80E+03	2.16E-03	2.40E-03	1.28E+00	3.51E-01	1.28E+00	2.82E+02	2.21E+02
EMP2	2.61E+03	2.18E-03	2.43E-03	1.28E+00	3.58E-01	1.28E+00	1.53E+02	1.20E+02
GPLD1	1.38E+01	2.19E-03	2.43E-03	4.43E+00	2.15E+00	4.43E+00	1.13E+00	2.56E-01
OPCML	1.28E+02	2.21E-03	2.45E-03	5.62E+00	2.49E+00	5.62E+00	1.10E+01	1.95E+00
PCDHB8	1.23E+02	2.22E-03	2.46E-03	1.55E+00	6.33E-01	1.55E+00	7.78E+00	5.01E+00
GRIP2	2.01E+02	2.22E-03	2.46E-03	2.10E+00	1.07E+00	2.10E+00	1.40E+01	6.67E+00
DUSP3	5.82E+02	2.23E-03	2.47E-03	1.22E+00	2.90E-01	1.22E+00	3.35E+01	2.74E+01
RAB2B	5.83E+02	2.23E-03	2.47E-03	1.19E+00	2.50E-01	1.19E+00	3.30E+01	2.78E+01
XR_233521.4	1.02E+02	2.26E-03	2.50E-03	1.70E+00	7.69E-01	1.70E+00	6.64E+00	3.89E+00
NFIC	4.84E+03	2.31E-03	2.55E-03	1.33E+00	4.16E-01	1.33E+00	2.88E+02	2.16E+02
XR_003030102.1	1.62E+01	2.32E-03	2.56E-03	1.83E+00	8.71E-01	1.83E+00	1.08E+00	5.89E-01
XR_003031758.1	4.87E+02	2.43E-03	2.69E-03	1.28E+00	3.55E-01	1.28E+00	2.85E+01	2.23E+01
EML3	1.59E+03	2.45E-03	2.70E-03	1.18E+00	2.34E-01	1.18E+00	8.98E+01	7.64E+01

IL1RL1	6.75E+00	2.50E-03	2.75E-03	2.42E+00	1.27E+00	2.42E+00	4.94E-01	2.04E-01
XR_003030756.1	4.12E+01	2.51E-03	2.77E-03	1.75E+00	8.05E-01	1.75E+00	2.71E+00	1.55E+00
MRC2	1.37E+04	2.53E-03	2.79E-03	1.32E+00	3.97E-01	1.32E+00	8.15E+02	6.19E+02
C19H17orf113	1.99E+02	2.56E-03	2.82E-03	1.29E+00	3.62E-01	1.29E+00	1.17E+01	9.07E+00
XR_003033860.1	1.76E+01	2.62E-03	2.89E-03	4.11E+00	2.04E+00	4.11E+00	1.43E+00	3.47E-01
XR_814092.3	6.58E+01	2.72E-03	2.99E-03	1.49E+00	5.77E-01	1.49E+00	4.08E+00	2.74E+00
GNAT2	1.38E+01	2.75E-03	3.02E-03	1.98E+00	9.86E-01	1.98E+00	9.48E-01	4.79E-01
LOC112448540	1.61E+01	2.77E-03	3.04E-03	4.51E+00	2.17E+00	4.51E+00	1.33E+00	2.96E-01
XR_234798.4	2.24E+01	2.78E-03	3.05E-03	3.94E+00	1.98E+00	3.94E+00	1.80E+00	4.58E-01
XR_003037247.1	4.12E+01	2.81E-03	3.08E-03	1.64E+00	7.13E-01	1.64E+00	2.65E+00	1.62E+00
XR_001494379.1	2.06E+02	2.87E-03	3.15E-03	1.43E+00	5.11E-01	1.43E+00	1.26E+01	8.81E+00
XR_003035349.1	2.07E+01	2.94E-03	3.22E-03	1.74E+00	7.98E-01	1.74E+00	1.36E+00	7.83E-01
XR_003031251.1	5.11E+00	2.98E-03	3.26E-03	2.12E+00	1.09E+00	2.12E+00	3.59E-01	1.69E-01
FAM170B	7.59E+00	3.01E-03	3.29E-03	4.78E+00	2.26E+00	4.78E+00	6.33E-01	1.32E-01
CCDC174	4.78E+02	3.02E-03	3.30E-03	1.20E+00	2.58E-01	1.20E+00	2.73E+01	2.28E+01
XR_003037207.1	2.46E+00	3.05E-03	3.33E-03	1.26E+01	3.65E+00	1.26E+01	2.29E-01	1.82E-02
XR_003038092.1	2.04E+01	3.10E-03	3.39E-03	1.86E+00	8.99E-01	1.86E+00	1.37E+00	7.36E-01
XR_003033129.1	1.70E+01	3.11E-03	3.39E-03	6.56E+00	2.71E+00	6.56E+00	1.48E+00	2.26E-01
HOXA6	2.47E+02	3.12E-03	3.40E-03	2.55E+00	1.35E+00	2.55E+00	1.82E+01	7.13E+00
NDRG2	2.25E+03	3.21E-03	3.50E-03	1.35E+00	4.35E-01	1.35E+00	1.35E+02	9.95E+01
XR_003029542.1	2.81E+00	3.23E-03	3.52E-03	5.95E+00	2.57E+00	5.95E+00	2.44E-01	4.10E-02
LARGE1	9.37E+02	3.30E-03	3.59E-03	1.17E+00	2.22E-01	1.17E+00	5.26E+01	4.51E+01
XR_003037763.1	5.66E+00	3.41E-03	3.71E-03	2.13E+00	1.09E+00	2.13E+00	3.98E-01	1.87E-01
IL1RAPL1	2.58E+00	3.51E-03	3.82E-03	2.79E+00	1.48E+00	2.79E+00	1.94E-01	6.96E-02
XR_239993.4	3.33E+01	3.61E-03	3.93E-03	1.66E+00	7.34E-01	1.66E+00	2.16E+00	1.30E+00
PARD6G	3.30E+02	3.62E-03	3.94E-03	1.29E+00	3.64E-01	1.29E+00	1.94E+01	1.51E+01
LOC101906315	1.16E+01	3.64E-03	3.95E-03	1.96E+00	9.71E-01	1.96E+00	7.89E-01	4.03E-01
XR_810846.3	5.34E+00	3.65E-03	3.96E-03	5.28E+00	2.40E+00	5.28E+00	4.54E-01	8.60E-02
LOC100300896	4.87E+02	3.67E-03	3.98E-03	2.21E+00	1.14E+00	2.21E+00	3.43E+01	1.55E+01
MPZL1	2.66E+03	3.68E-03	3.99E-03	1.17E+00	2.22E-01	1.17E+00	1.50E+02	1.28E+02
XR_003036097.1	4.77E+01	3.77E-03	4.08E-03	2.08E+00	1.05E+00	2.08E+00	3.32E+00	1.60E+00
LOC789035	5.98E+00	3.79E-03	4.10E-03	4.12E+00	2.04E+00	4.12E+00	4.89E-01	1.18E-01
XR_814093.3	9.30E+01	3.89E-03	4.21E-03	1.39E+00	4.73E-01	1.39E+00	5.60E+00	4.04E+00

XR_003032809.1	7.44E+00	3.91E-03	4.22E-03	3.64E+00	1.87E+00	3.64E+00	5.93E-01	1.63E-01
TEP1	7.34E+02	4.04E-03	4.37E-03	1.34E+00	4.21E-01	1.34E+00	4.39E+01	3.28E+01
RIT1	1.05E+03	4.07E-03	4.39E-03	1.15E+00	2.05E-01	1.15E+00	5.88E+01	5.10E+01
XR_003035990.1	7.62E+00	4.20E-03	4.53E-03	7.25E+00	2.86E+00	7.25E+00	6.76E-01	9.32E-02
MXRA5	7.06E+03	4.20E-03	4.53E-03	1.87E+00	9.06E-01	1.87E+00	4.73E+02	2.53E+02
MEIS2	8.33E+02	4.32E-03	4.66E-03	1.54E+00	6.24E-01	1.54E+00	5.25E+01	3.41E+01
XR_806997.3	1.34E+01	4.36E-03	4.69E-03	1.74E+00	8.01E-01	1.74E+00	8.71E-01	5.00E-01
XR_003036120.1	2.35E+01	4.37E-03	4.71E-03	1.42E+00	5.10E-01	1.42E+00	1.44E+00	1.01E+00
VIRMA	2.31E+03	4.42E-03	4.76E-03	1.17E+00	2.30E-01	1.17E+00	1.30E+02	1.11E+02
XR_003036064.1	5.76E+00	4.53E-03	4.87E-03	3.05E+00	1.61E+00	3.05E+00	4.39E-01	1.44E-01
XR_003033866.1	1.75E+02	4.55E-03	4.89E-03	1.42E+00	5.03E-01	1.42E+00	1.07E+01	7.55E+00
XR_003029505.1	6.53E+00	4.70E-03	5.05E-03	1.30E+01	3.70E+00	1.30E+01	6.11E-01	4.71E-02
PDGFRB	6.72E+03	4.73E-03	5.08E-03	1.33E+00	4.06E-01	1.33E+00	4.00E+02	3.02E+02
ANKRD42	2.21E+02	4.83E-03	5.18E-03	1.38E+00	4.69E-01	1.38E+00	1.33E+01	9.60E+00
MYEF2	1.33E+03	4.92E-03	5.27E-03	1.22E+00	2.84E-01	1.22E+00	7.63E+01	6.26E+01
XR_003032941.1	6.06E+00	4.99E-03	5.35E-03	4.47E+00	2.16E+00	4.47E+00	4.98E-01	1.11E-01
XR_003031472.1	2.28E+00	5.32E-03	5.69E-03	2.63E+00	1.40E+00	2.63E+00	1.67E-01	6.36E-02
ANKRD13A	1.17E+03	5.32E-03	5.69E-03	1.16E+00	2.11E-01	1.16E+00	6.57E+01	5.67E+01
XR_003035229.1	3.59E+00	5.49E-03	5.87E-03	4.56E+00	2.19E+00	4.56E+00	3.00E-01	6.58E-02
GCSAML	3.66E+00	5.58E-03	5.97E-03	1.00E+01	3.33E+00	1.00E+01	3.36E-01	3.35E-02
LOC616094	6.26E+00	5.60E-03	5.99E-03	2.67E+00	1.42E+00	2.67E+00	4.65E-01	1.74E-01
XR_808632.3	6.00E+00	5.94E-03	6.34E-03	4.22E+00	2.08E+00	4.22E+00	4.90E-01	1.16E-01
LOC112447438	5.65E+00	5.97E-03	6.37E-03	2.91E+00	1.54E+00	2.91E+00	4.24E-01	1.46E-01
XR_813935.3	3.79E+01	6.06E-03	6.46E-03	2.00E+00	1.00E+00	2.00E+00	2.64E+00	1.32E+00
C1H21orf91	6.85E+02	6.15E-03	6.55E-03	1.34E+00	4.24E-01	1.34E+00	4.12E+01	3.07E+01
MMP16	7.42E+02	6.25E-03	6.66E-03	1.99E+00	9.94E-01	1.99E+00	5.08E+01	2.55E+01
IFT52	4.93E+02	6.28E-03	6.69E-03	1.15E+00	1.99E-01	1.15E+00	2.75E+01	2.40E+01
NEURL3	3.24E+01	6.32E-03	6.73E-03	1.66E+00	7.35E-01	1.66E+00	2.08E+00	1.25E+00
LOC107131660	2.88E+01	6.38E-03	6.78E-03	3.11E+00	1.64E+00	3.11E+00	2.21E+00	7.11E-01
DACT3	6.58E+02	6.38E-03	6.78E-03	1.30E+00	3.75E-01	1.30E+00	3.89E+01	3.00E+01
XR_003029509.1	2.55E+00	6.49E-03	6.89E-03	3.76E+00	1.91E+00	3.76E+00	2.04E-01	5.44E-02
GPALPP1	6.23E+02	6.56E-03	6.97E-03	1.21E+00	2.72E-01	1.21E+00	3.56E+01	2.95E+01
OTOP1	3.49E+01	6.71E-03	7.12E-03	1.81E+00	8.55E-01	1.81E+00	2.32E+00	1.28E+00

ACAD10	7.44E+02	6.76E-03	7.17E-03	1.19E+00	2.51E-01	1.19E+00	4.23E+01	3.55E+01
TMEM138	7.30E+02	6.80E-03	7.20E-03	1.27E+00	3.42E-01	1.27E+00	4.27E+01	3.37E+01
NR_031314.1	5.83E+00	6.86E-03	7.27E-03	1.07E+01	3.42E+00	1.07E+01	5.35E-01	5.01E-02
XR_003030613.1	3.26E+00	7.05E-03	7.46E-03	2.38E+00	1.25E+00	2.38E+00	2.35E-01	9.89E-02
RAB44	4.69E+00	7.17E-03	7.59E-03	3.59E+00	1.85E+00	3.59E+00	3.78E-01	1.05E-01
XR_003029600.1	2.86E+02	7.19E-03	7.60E-03	1.63E+00	7.09E-01	1.63E+00	1.84E+01	1.12E+01
XR_001502300.2	6.62E+00	7.30E-03	7.71E-03	4.17E+00	2.06E+00	4.17E+00	5.41E-01	1.30E-01
XR_003033489.1	4.27E+00	7.42E-03	7.83E-03	4.57E+00	2.19E+00	4.57E+00	3.56E-01	7.79E-02
LOC100847546	1.46E+01	7.52E-03	7.93E-03	1.65E+00	7.21E-01	1.65E+00	9.44E-01	5.73E-01
XR_238184.4	3.31E+00	7.70E-03	8.12E-03	5.77E+00	2.53E+00	5.77E+00	2.83E-01	4.90E-02
LOC781778	9.48E+00	7.71E-03	8.12E-03	1.93E+00	9.48E-01	1.93E+00	6.42E-01	3.33E-01
NOVA2	1.13E+02	7.83E-03	8.25E-03	1.29E+00	3.69E-01	1.29E+00	6.66E+00	5.16E+00
XR_003033961.1	3.16E+00	7.84E-03	8.25E-03	2.94E+00	1.55E+00	2.94E+00	2.40E-01	8.16E-02
ATXN7L3B	2.46E+03	7.98E-03	8.40E-03	1.18E+00	2.34E-01	1.18E+00	1.39E+02	1.18E+02
DIP2B	6.60E+02	8.04E-03	8.45E-03	1.34E+00	4.20E-01	1.34E+00	3.95E+01	2.95E+01
XR_003037126.1	1.07E+01	8.35E-03	8.78E-03	3.11E+00	1.63E+00	3.11E+00	8.22E-01	2.65E-01
JCAD	1.95E+03	8.51E-03	8.94E-03	1.32E+00	4.01E-01	1.32E+00	1.16E+02	8.76E+01
PRELP	8.02E+03	8.54E-03	8.96E-03	1.72E+00	7.85E-01	1.72E+00	5.23E+02	3.03E+02
FAM196A	1.62E+02	8.70E-03	9.12E-03	1.43E+00	5.14E-01	1.43E+00	9.90E+00	6.93E+00
FBLN1	2.27E+04	8.84E-03	9.27E-03	1.64E+00	7.12E-01	1.64E+00	1.46E+03	8.89E+02
ZBED8	5.06E+02	9.15E-03	9.58E-03	1.17E+00	2.22E-01	1.17E+00	2.86E+01	2.45E+01
KRT24	1.66E+01	9.26E-03	9.69E-03	3.77E+00	1.92E+00	3.77E+00	1.33E+00	3.52E-01
DYRK1A	1.76E+03	9.39E-03	9.83E-03	1.21E+00	2.71E-01	1.21E+00	1.01E+02	8.37E+01
ZNF614	1.94E+02	9.44E-03	9.87E-03	1.26E+00	3.38E-01	1.26E+00	1.13E+01	8.95E+00
XR_003031776.1	1.17E+01	9.44E-03	9.87E-03	1.90E+00	9.27E-01	1.90E+00	7.99E-01	4.20E-01
METTL15	1.55E+02	9.71E-03	1.01E-02	1.21E+00	2.76E-01	1.21E+00	8.86E+00	7.32E+00
XR_003035129.1	3.13E+01	9.98E-03	1.04E-02	1.66E+00	7.32E-01	1.66E+00	2.02E+00	1.22E+00
ARHGEF12-2	8.83E+02	1.03E-02	1.08E-02	1.27E+00	3.47E-01	1.27E+00	5.17E+01	4.07E+01
ZNF81	8.02E+00	1.10E-02	1.14E-02	1.85E+00	8.88E-01	1.85E+00	5.34E-01	2.89E-01
MOB1B	8.04E+02	1.10E-02	1.14E-02	1.20E+00	2.59E-01	1.20E+00	4.58E+01	3.83E+01
XR_003030365.1	1.37E+01	1.21E-02	1.26E-02	3.02E+00	1.59E+00	3.02E+00	1.05E+00	3.47E-01
XR_003035726.1	1.84E+01	1.22E-02	1.26E-02	2.96E+00	1.57E+00	2.96E+00	1.41E+00	4.75E-01
XR_003037504.1	7.35E+00	1.22E-02	1.26E-02	3.74E+00	1.90E+00	3.74E+00	5.85E-01	1.56E-01

LOC101910094	4.50E+00	1.27E-02	1.32E-02	3.54E+00	1.83E+00	3.54E+00	3.58E-01	1.01E-01
OR13J1	2.49E+00	1.29E-02	1.34E-02	7.84E+00	2.97E+00	7.84E+00	2.20E-01	2.81E-02
STOML1	3.04E+02	1.32E-02	1.37E-02	1.26E+00	3.31E-01	1.26E+00	1.76E+01	1.40E+01
ACAP3	3.89E+03	1.35E-02	1.40E-02	1.22E+00	2.86E-01	1.22E+00	2.24E+02	1.84E+02
XR_003035059.1	1.85E+00	1.41E-02	1.46E-02	2.42E+00	1.28E+00	2.42E+00	1.34E-01	5.55E-02
RERE	6.28E+03	1.44E-02	1.49E-02	1.37E+00	4.53E-01	1.37E+00	3.77E+02	2.75E+02
FXYD7	9.04E+00	1.45E-02	1.50E-02	1.64E+00	7.12E-01	1.64E+00	5.81E-01	3.55E-01
XR_003037685.1	1.20E+00	1.49E-02	1.54E-02	2.31E+01	4.53E+00	2.31E+01	1.15E-01	4.96E-03
RCBTB2	1.49E+03	1.49E-02	1.54E-02	1.13E+00	1.79E-01	1.13E+00	8.27E+01	7.31E+01
SLC27A5	1.75E+02	1.52E-02	1.57E-02	1.27E+00	3.41E-01	1.27E+00	1.02E+01	8.06E+00
FTO	1.53E+03	1.54E-02	1.59E-02	1.17E+00	2.23E-01	1.17E+00	8.61E+01	7.37E+01
XR_001501102.2	2.93E+00	1.57E-02	1.62E-02	2.94E+00	1.56E+00	2.94E+00	2.22E-01	7.55E-02
SH3BP5	1.37E+03	1.58E-02	1.63E-02	1.35E+00	4.38E-01	1.35E+00	8.24E+01	6.08E+01
LOC112448773	6.51E+00	1.58E-02	1.63E-02	2.90E+00	1.54E+00	2.90E+00	4.96E-01	1.71E-01
RNF145	1.43E+03	1.61E-02	1.66E-02	1.12E+00	1.64E-01	1.12E+00	7.91E+01	7.06E+01
URGCP	5.75E+02	1.63E-02	1.68E-02	1.12E+00	1.65E-01	1.12E+00	3.18E+01	2.84E+01
PGAM2	1.15E+01	1.64E-02	1.69E-02	1.50E+00	5.81E-01	1.50E+00	7.13E-01	4.77E-01
TMEM18	1.98E+02	1.66E-02	1.71E-02	1.20E+00	2.67E-01	1.20E+00	1.13E+01	9.39E+00
XR_805838.3	3.60E+00	1.73E-02	1.78E-02	2.02E+00	1.01E+00	2.02E+00	2.47E-01	1.22E-01
SLC35E3	7.17E+02	1.78E-02	1.83E-02	1.29E+00	3.62E-01	1.29E+00	4.19E+01	3.26E+01
DACT1	1.84E+03	1.86E-02	1.91E-02	1.30E+00	3.80E-01	1.30E+00	1.08E+02	8.34E+01
XR_003029590.1	2.06E+01	1.90E-02	1.95E-02	3.47E+00	1.80E+00	3.47E+00	1.63E+00	4.68E-01
FUBP3	8.25E+02	1.90E-02	1.95E-02	1.16E+00	2.14E-01	1.16E+00	4.65E+01	4.00E+01
LOC786512	7.81E+01	1.94E-02	1.99E-02	1.39E+00	4.79E-01	1.39E+00	4.70E+00	3.37E+00
HTR2A	3.28E+00	2.02E-02	2.07E-02	2.55E+00	1.35E+00	2.55E+00	2.41E-01	9.46E-02
SP7	3.63E+01	2.18E-02	2.23E-02	1.49E+00	5.77E-01	1.49E+00	2.27E+00	1.52E+00
LOC787328	3.74E+00	2.29E-02	2.35E-02	5.27E+00	2.40E+00	5.27E+00	3.17E-01	6.03E-02
XR_813683.3	1.08E+01	2.32E-02	2.37E-02	3.06E+00	1.61E+00	3.06E+00	8.35E-01	2.73E-01
RRBP1	1.22E+04	2.33E-02	2.38E-02	1.16E+00	2.10E-01	1.16E+00	6.89E+02	5.96E+02
XR_003033176.1	1.84E+03	2.41E-02	2.47E-02	1.27E+00	3.50E-01	1.27E+00	1.07E+02	8.41E+01
XR_810077.2	3.28E+01	2.48E-02	2.53E-02	1.65E+00	7.19E-01	1.65E+00	2.11E+00	1.28E+00
SLC37A3	7.08E+02	2.50E-02	2.55E-02	1.13E+00	1.73E-01	1.13E+00	3.93E+01	3.48E+01
C21H14orf132	1.80E+00	2.50E-02	2.55E-02	6.89E+00	2.78E+00	6.89E+00	1.59E-01	2.31E-02

XR_003034598.1	1.95E+00	2.52E-02	2.57E-02	4.61E+00	2.21E+00	4.61E+00	1.62E-01	3.52E-02
ADAMTS20	4.72E+00	2.56E-02	2.61E-02	1.94E+00	9.56E-01	1.94E+00	3.18E-01	1.64E-01
LOC100140533	1.61E+02	2.76E-02	2.81E-02	1.16E+00	2.19E-01	1.16E+00	9.07E+00	7.79E+00
XR_003033835.1	3.88E+01	2.83E-02	2.88E-02	1.87E+00	9.04E-01	1.87E+00	2.58E+00	1.38E+00
NAV1	3.80E+03	2.97E-02	3.02E-02	1.25E+00	3.22E-01	1.25E+00	2.20E+02	1.76E+02
XR_003037293.1	2.00E+00	3.09E-02	3.14E-02	7.34E+00	2.88E+00	7.34E+00	1.78E-01	2.43E-02
LOC112447439	3.18E+00	3.12E-02	3.17E-02	2.47E+00	1.31E+00	2.47E+00	2.30E-01	9.31E-02
XR_003032625.1	4.62E+02	3.16E-02	3.21E-02	1.38E+00	4.65E-01	1.38E+00	2.79E+01	2.02E+01
TGFB3	2.46E+03	3.19E-02	3.24E-02	1.50E+00	5.85E-01	1.50E+00	1.53E+02	1.02E+02
XR_003029373.1	2.86E+00	3.23E-02	3.28E-02	7.33E+00	2.87E+00	7.33E+00	2.53E-01	3.46E-02
XR_003034248.1	2.38E+00	3.44E-02	3.49E-02	4.21E+00	2.07E+00	4.21E+00	1.96E-01	4.65E-02
LOC100335642	6.04E+01	3.59E-02	3.63E-02	1.50E+00	5.81E-01	1.50E+00	3.77E+00	2.52E+00
CLCA3	2.49E+00	3.81E-02	3.86E-02	3.12E+00	1.64E+00	3.12E+00	1.92E-01	6.15E-02
STX17	6.65E+02	4.28E-02	4.33E-02	1.23E+00	2.96E-01	1.23E+00	3.82E+01	3.11E+01
PTPRS	1.06E+04	4.91E-02	4.97E-02	1.17E+00	2.22E-01	1.17E+00	6.01E+02	5.15E+02
XR_003034005.1	9.24E+00	5.43E-02	5.49E-02	2.86E+00	1.52E+00	2.86E+00	6.99E-01	2.44E-01
XR_003034438.1	1.64E+00	5.56E-02	5.62E-02	1.29E+01	3.68E+00	1.29E+01	1.53E-01	1.19E-02
NAMPT	5.75E+02	6.18E-02	6.25E-02	1.11E+00	1.55E-01	1.11E+00	3.17E+01	2.85E+01
XR_812723.3	3.62E+01	6.21E-02	6.27E-02	1.37E+00	4.52E-01	1.37E+00	2.18E+00	1.59E+00
VAT1L	2.13E+02	6.27E-02	6.32E-02	1.56E+00	6.39E-01	1.56E+00	1.33E+01	8.57E+00
XR_003031808.1	3.46E+00	6.56E-02	6.62E-02	4.57E+00	2.19E+00	4.57E+00	2.89E-01	6.32E-02
LOC101905975	5.73E+00	6.59E-02	6.65E-02	1.69E+00	7.54E-01	1.69E+00	3.68E-01	2.18E-01
XR_003030727.1	3.60E+02	7.44E-02	7.49E-02	1.32E+00	3.97E-01	1.32E+00	2.13E+01	1.62E+01
C26H10orf62	8.67E+00	7.51E-02	7.56E-02	1.43E+00	5.12E-01	1.43E+00	5.30E-01	3.71E-01
ZMIZ1	3.82E+03	7.98E-02	8.03E-02	1.24E+00	3.05E-01	1.24E+00	2.21E+02	1.79E+02
XR_001501363.1	1.09E+01	9.03E-02	9.08E-02	7.16E+00	2.84E+00	7.16E+00	9.60E-01	1.34E-01
XR_813169.3	2.88E+00	9.25E-02	9.30E-02	6.97E+00	2.80E+00	6.97E+00	2.54E-01	3.64E-02
XR_003032060.1	2.46E+02	9.25E-02	9.30E-02	1.21E+00	2.73E-01	1.21E+00	1.41E+01	1.17E+01
XR_003030726.1	4.81E+02	9.29E-02	9.33E-02	1.27E+00	3.44E-01	1.27E+00	2.80E+01	2.21E+01
CAVIN4	1.51E+02	9.69E-02	9.72E-02	1.17E+00	2.31E-01	1.17E+00	8.53E+00	7.27E+00
XR_003033122.1	2.75E+02	1.09E-01	1.09E-01	1.16E+00	2.16E-01	1.16E+00	1.54E+01	1.33E+01
ZCCHC24	2.09E+03	1.67E-01	1.67E-01	1.20E+00	2.68E-01	1.20E+00	1.19E+02	9.91E+01
LOC101903326	4.74E+02	2.61E-01	2.62E-01	1.08E+00	1.17E-01	1.08E+00	2.58E+01	2.38E+01

XR_003034251.1	2.88E+00	2.66E-01	2.66E-01	8.40E+00	3.07E+00	8.40E+00	2.59E-01	3.08E-02
XR_807452.3	7.88E+00	3.19E-01	3.19E-01	3.22E+00	1.69E+00	3.22E+00	6.14E-01	1.91E-01
XR_003030423.1	3.68E+00	8.70E-01	8.70E-01	1.90E+00	9.27E-01	1.90E+00	2.48E-01	1.30E-01

S4 Table. Gene Specific Analysis (late versus early gestation) for genes in cluster 3. Shown are the fold change and its significance (p-value), log 2 ratio and False Discovery Rate (FDR) across gestation; least square (LS) mean (late and early gestation); total counts per million.

Gene symbol	Total counts	P-value	FDR step up	Ratio	Log2(Ratio)	Fold change	LSMean(Late)	LSMean(Early)
PPM1H	1.96E+01	1.78E-10	1.55E-07	1.95E+04	1.43E+01	1.95E+04	1.96E+00	1.00E-04
XR_003036541.1	3.19E+00	8.55E-08	3.72E-05	3.87E+01	5.27E+00	3.87E+01	3.11E-01	8.05E-03
XR_003035353.1	6.62E+01	3.94E-07	1.14E-04	1.14E+01	3.51E+00	1.14E+01	6.14E+00	5.38E-01
LCT	3.67E+01	8.73E-07	1.73E-04	2.16E+01	4.43E+00	2.16E+01	3.52E+00	1.63E-01
LOC104969177	3.23E+01	9.94E-07	1.73E-04	4.98E+00	2.32E+00	4.98E+00	2.74E+00	5.50E-01
XR_804252.1	1.95E+00	1.24E-06	1.80E-04	3.93E+02	8.62E+00	3.93E+02	1.95E-01	4.95E-04
LOC781927	1.73E+00	1.79E-06	1.89E-04	2.63E+01	4.72E+00	2.63E+01	1.67E-01	6.35E-03
XR_003037470.1	6.77E+00	1.88E-06	1.89E-04	2.06E+01	4.36E+00	2.06E+01	6.48E-01	3.15E-02
BMP15	1.92E+02	2.33E-06	1.89E-04	3.95E+01	5.30E+00	3.95E+01	1.88E+01	4.76E-01
GPR179	3.21E+01	2.36E-06	1.89E-04	8.92E+00	3.16E+00	8.92E+00	2.92E+00	3.27E-01
XR_003036766.1	5.31E+00	2.39E-06	1.89E-04	2.31E+01	4.53E+00	2.31E+01	5.12E-01	2.21E-02
id-IGHM	4.49E+02	2.60E-06	1.89E-04	1.11E+01	3.47E+00	1.11E+01	4.16E+01	3.75E+00
ABCC2	1.71E+02	3.17E-06	2.10E-04	1.11E+01	3.47E+00	1.11E+01	1.58E+01	1.43E+00
ZP3	1.21E+03	3.38E-06	2.10E-04	1.54E+01	3.94E+00	1.54E+01	1.15E+02	7.46E+00
KHDC3L	3.86E+02	4.75E-06	2.76E-04	7.98E+00	3.00E+00	7.98E+00	3.47E+01	4.35E+00
TREH	1.26E+02	5.11E-06	2.78E-04	1.55E+01	3.95E+00	1.55E+01	1.19E+01	7.69E-01
LOC101904481	2.40E+02	5.88E-06	3.01E-04	9.59E+00	3.26E+00	9.59E+00	2.19E+01	2.28E+00
XR_003031564.1	5.53E+00	8.63E-06	3.76E-04	2.36E+01	4.56E+00	2.36E+01	5.32E-01	2.26E-02
XR_003032366.1	4.15E+01	8.81E-06	3.76E-04	5.41E+00	2.44E+00	5.41E+00	3.56E+00	6.58E-01
KPNA7	1.25E+02	9.36E-06	3.76E-04	5.28E+00	2.40E+00	5.28E+00	1.07E+01	2.02E+00
CNTD2	1.80E+02	9.66E-06	3.76E-04	8.31E+00	3.05E+00	8.31E+00	1.62E+01	1.96E+00
ARHGDIG	3.59E+01	1.05E-05	3.76E-04	4.39E+00	2.13E+00	4.39E+00	2.98E+00	6.79E-01
XR_003035912.1	1.29E+00	1.06E-05	3.76E-04	2.60E+01	4.70E+00	2.60E+01	1.24E-01	4.78E-03
FAM170A	1.28E+00	1.06E-05	3.76E-04	1.25E+01	3.64E+00	1.25E+01	1.20E-01	9.60E-03
SLCO3A1	2.71E+02	1.08E-05	3.76E-04	2.16E+00	1.11E+00	2.16E+00	1.91E+01	8.84E+00
OLFM4	5.44E+01	1.41E-05	4.72E-04	7.17E+00	2.84E+00	7.17E+00	4.84E+00	6.75E-01
MCF2	1.87E+01	1.47E-05	4.73E-04	3.16E+00	1.66E+00	3.16E+00	1.46E+00	4.61E-01
DRD3	4.87E+00	1.53E-05	4.74E-04	2.21E+01	4.46E+00	2.21E+01	4.68E-01	2.12E-02
XR_003033896.1	7.12E+01	1.77E-05	5.28E-04	2.77E+01	4.79E+00	2.77E+01	6.90E+00	2.49E-01

LOC522174	3.55E+01	1.84E-05	5.28E-04	5.27E+00	2.40E+00	5.27E+00	3.04E+00	5.76E-01
LOC104970698	2.06E+01	1.88E-05	5.28E-04	4.65E+00	2.22E+00	4.65E+00	1.72E+00	3.71E-01
SLBP2	6.57E+01	2.08E-05	5.65E-04	5.50E+00	2.46E+00	5.50E+00	5.65E+00	1.03E+00
NPM2	3.66E+02	2.19E-05	5.78E-04	1.11E+01	3.48E+00	1.11E+01	3.39E+01	3.04E+00
XR_001501628.2	3.94E+01	2.68E-05	6.86E-04	3.34E+01	5.06E+00	3.34E+01	3.83E+00	1.15E-01
XR_003033142.1	1.43E+00	2.85E-05	7.10E-04	2.85E+01	4.83E+00	2.85E+01	1.39E-01	4.87E-03
d-LOC10190246	3.92E+02	3.06E-05	7.36E-04	5.91E+00	2.56E+00	5.91E+00	3.40E+01	5.76E+00
SELP	2.22E+02	3.18E-05	7.36E-04	2.71E+00	1.44E+00	2.71E+00	1.66E+01	6.13E+00
EGR3	1.40E+02	3.24E-05	7.36E-04	3.99E+00	2.00E+00	3.99E+00	1.14E+01	2.86E+00
MPIG6B	6.19E+02	3.29E-05	7.36E-04	9.77E+00	3.29E+00	9.77E+00	5.67E+01	5.80E+00
MOS	9.91E+01	3.63E-05	7.77E-04	1.78E+01	4.15E+00	1.78E+01	9.43E+00	5.30E-01
TBPL2	3.50E+00	3.66E-05	7.77E-04	1.18E+01	3.56E+00	1.18E+01	3.25E-01	2.75E-02
XR_003031851.1	4.23E+00	5.16E-05	1.07E-03	1.04E+01	3.38E+00	1.04E+01	3.89E-01	3.74E-02
MYO1H	3.32E+01	5.30E-05	1.07E-03	6.45E+00	2.69E+00	6.45E+00	2.91E+00	4.52E-01
XR_003029820.1	3.26E+00	7.08E-05	1.39E-03	1.17E+01	3.55E+00	1.17E+01	3.03E-01	2.58E-02
XR_003035869.1	1.90E+00	7.26E-05	1.39E-03	7.45E+01	6.22E+00	7.45E+01	1.87E-01	2.52E-03
LOC783811	3.26E+01	7.32E-05	1.39E-03	3.81E+01	5.25E+00	3.81E+01	3.18E+00	8.35E-02
XR_811475.3	4.63E+00	7.78E-05	1.44E-03	1.55E+01	3.95E+00	1.55E+01	4.38E-01	2.82E-02
XR_003036902.1	2.65E+01	8.19E-05	1.49E-03	4.37E+00	2.13E+00	4.37E+00	2.20E+00	5.03E-01
MAPRE2	2.51E+03	8.59E-05	1.53E-03	1.97E+00	9.78E-01	1.97E+00	1.72E+02	8.73E+01
XR_003036075.1	2.09E+01	9.15E-05	1.59E-03	2.45E+00	1.29E+00	2.45E+00	1.53E+00	6.26E-01
LOC618696	1.21E+01	9.82E-05	1.68E-03	8.12E+00	3.02E+00	8.12E+00	1.09E+00	1.34E-01
SLC6A5	1.71E+01	1.01E-04	1.69E-03	3.24E+00	1.70E+00	3.24E+00	1.34E+00	4.14E-01
TSPAN7	1.14E+03	1.06E-04	1.73E-03	2.62E+00	1.39E+00	2.62E+00	8.52E+01	3.25E+01
DSCAML1	1.52E+02	1.08E-04	1.73E-03	6.51E+00	2.70E+00	6.51E+00	1.33E+01	2.05E+00
SAXO1	3.10E+02	1.12E-04	1.73E-03	7.79E+00	2.96E+00	7.79E+00	2.78E+01	3.56E+00
CACNG3	1.09E+01	1.14E-04	1.73E-03	1.50E+01	3.91E+00	1.50E+01	1.03E+00	6.85E-02
XR_003030793.1	1.01E+00	1.15E-04	1.73E-03	3.19E+01	5.00E+00	3.19E+01	9.83E-02	3.08E-03
XR_003029444.1	1.69E+00	1.15E-04	1.73E-03	4.18E+01	5.39E+00	4.18E+01	1.65E-01	3.94E-03
XR_001495546.2	3.42E+00	1.29E-04	1.90E-03	1.97E+01	4.30E+00	1.97E+01	3.27E-01	1.66E-02
PLEKHG7	1.99E+02	1.43E-04	2.08E-03	5.46E+00	2.45E+00	5.46E+00	1.71E+01	3.13E+00
PANX1	6.31E+02	1.47E-04	2.10E-03	2.35E+00	1.23E+00	2.35E+00	4.56E+01	1.94E+01
KCTD16	1.03E+01	1.54E-04	2.17E-03	6.98E+00	2.80E+00	6.98E+00	9.16E-01	1.31E-01

XR_001494959.2	5.03E+00	1.61E-04	2.23E-03	5.24E+01	5.71E+00	5.24E+01	4.95E-01	9.44E-03
PIWIL3	2.80E+01	1.66E-04	2.24E-03	2.08E+01	4.38E+00	2.08E+01	2.68E+00	1.29E-01
XR_811073.3	6.32E+01	1.67E-04	2.24E-03	1.83E+01	4.20E+00	1.83E+01	6.02E+00	3.28E-01
PATL2	1.59E+02	1.85E-04	2.44E-03	6.60E+00	2.72E+00	6.60E+00	1.40E+01	2.13E+00
BCL2L10	5.28E+01	1.91E-04	2.49E-03	2.87E+00	1.52E+00	2.87E+00	4.02E+00	1.40E+00
MYO1E	9.67E+02	1.98E-04	2.54E-03	1.55E+00	6.30E-01	1.55E+00	6.11E+01	3.95E+01
MPP7	8.57E+01	2.16E-04	2.73E-03	3.32E+00	1.73E+00	3.32E+00	6.74E+00	2.03E+00
AURKA	1.08E+03	2.31E-04	2.87E-03	2.73E+00	1.45E+00	2.73E+00	8.14E+01	2.98E+01
XR_003033062.1	1.44E+00	2.37E-04	2.91E-03	7.01E+00	2.81E+00	7.01E+00	1.27E-01	1.82E-02
TRIM77	1.77E+01	2.42E-04	2.93E-03	1.50E+01	3.90E+00	1.50E+01	1.67E+00	1.11E-01
ILDR1	3.69E+01	2.53E-04	3.00E-03	4.30E+00	2.10E+00	4.30E+00	3.05E+00	7.09E-01
SLC17A1	5.65E+01	2.62E-04	3.00E-03	1.24E+01	3.63E+00	1.24E+01	5.26E+00	4.26E-01
XR_003035013.1	3.91E+00	2.62E-04	3.00E-03	1.13E+01	3.49E+00	1.13E+01	3.62E-01	3.21E-02
GLRA1	1.79E+01	2.65E-04	3.00E-03	1.04E+01	3.37E+00	1.04E+01	1.65E+00	1.59E-01
LYPD1	2.46E+02	2.65E-04	3.00E-03	1.91E+00	9.34E-01	1.91E+00	1.68E+01	8.77E+00
XR_003031039.1	9.47E+00	2.90E-04	3.21E-03	5.08E+00	2.34E+00	5.08E+00	8.04E-01	1.58E-01
SLC17A3	1.29E+01	2.91E-04	3.21E-03	1.56E+01	3.96E+00	1.56E+01	1.22E+00	7.79E-02
ARHGEF26	3.22E+02	3.02E-04	3.23E-03	1.65E+00	7.18E-01	1.65E+00	2.08E+01	1.26E+01
LOC100337390	8.73E+01	3.03E-04	3.23E-03	3.00E+00	1.59E+00	3.00E+00	6.72E+00	2.24E+00
XR_001501757.2	5.06E+00	3.04E-04	3.23E-03	1.19E+01	3.57E+00	1.19E+01	4.70E-01	3.95E-02
LOC104972713	1.64E+01	3.13E-04	3.29E-03	4.95E+00	2.31E+00	4.95E+00	1.39E+00	2.81E-01
XR_003035686.1	4.96E+00	3.32E-04	3.40E-03	9.00E+00	3.17E+00	9.00E+00	4.51E-01	5.01E-02
PAK5	1.94E+01	3.32E-04	3.40E-03	2.52E+00	1.33E+00	2.52E+00	1.43E+00	5.68E-01
CCNO	8.18E+01	3.46E-04	3.51E-03	4.85E+00	2.28E+00	4.85E+00	6.90E+00	1.42E+00
SPATA5	2.60E+02	3.63E-04	3.56E-03	1.57E+00	6.55E-01	1.57E+00	1.66E+01	1.05E+01
CACNA1E	1.10E+02	3.66E-04	3.56E-03	3.38E+00	1.76E+00	3.38E+00	8.71E+00	2.58E+00
C15H11orf52	1.35E+01	3.66E-04	3.56E-03	4.64E+00	2.21E+00	4.64E+00	1.13E+00	2.44E-01
ITGB4	7.13E+02	3.68E-04	3.56E-03	2.52E+00	1.33E+00	2.52E+00	5.26E+01	2.08E+01
XR_240539.4	5.09E+01	3.81E-04	3.63E-03	2.96E+00	1.57E+00	2.96E+00	3.90E+00	1.32E+00
CD27	3.62E+01	3.83E-04	3.63E-03	2.21E+00	1.14E+00	2.21E+00	2.58E+00	1.17E+00
MAP2	9.80E+02	3.89E-04	3.64E-03	1.52E+00	6.08E-01	1.52E+00	6.16E+01	4.04E+01
HEATR1	1.94E+01	3.93E-04	3.64E-03	4.07E+00	2.03E+00	4.07E+00	1.59E+00	3.90E-01
XR_003030871.1	1.25E+01	4.05E-04	3.71E-03	4.61E+00	2.21E+00	4.61E+00	1.04E+00	2.26E-01

CEACAM20	9.66E+00	4.12E-04	3.73E-03	4.23E+00	2.08E+00	4.23E+00	7.96E-01	1.88E-01
KRT33A	3.24E+00	4.17E-04	3.75E-03	1.58E+01	3.98E+00	1.58E+01	3.06E-01	1.94E-02
XR_003036726.1	1.16E+01	4.27E-04	3.79E-03	2.55E+00	1.35E+00	2.55E+00	8.56E-01	3.36E-01
CLPS	1.28E+00	4.44E-04	3.90E-03	9.19E+00	3.20E+00	9.19E+00	1.17E-01	1.27E-02
LOC518980	4.76E+01	4.81E-04	4.19E-03	1.94E+00	9.54E-01	1.94E+00	3.25E+00	1.68E+00
RAB39B	1.76E+01	4.95E-04	4.27E-03	4.57E+00	2.19E+00	4.57E+00	1.47E+00	3.21E-01
FAM19A2	2.92E+01	5.01E-04	4.28E-03	4.30E+00	2.10E+00	4.30E+00	2.41E+00	5.62E-01
XR_003036006.1	3.33E+00	5.19E-04	4.34E-03	1.02E+01	3.35E+00	1.02E+01	3.06E-01	3.01E-02
LY6G6C	1.98E+01	5.26E-04	4.34E-03	4.92E+00	2.30E+00	4.92E+00	1.67E+00	3.40E-01
UCP3	3.16E+01	5.27E-04	4.34E-03	1.04E+01	3.38E+00	1.04E+01	2.91E+00	2.79E-01
LRMP	2.53E+01	5.28E-04	4.34E-03	2.46E+00	1.30E+00	2.46E+00	1.85E+00	7.55E-01
XR_811466.3	9.60E+00	5.39E-04	4.37E-03	3.18E+00	1.67E+00	3.18E+00	7.49E-01	2.35E-01
XR_003034297.1	3.27E+00	5.42E-04	4.37E-03	7.34E+00	2.88E+00	7.34E+00	2.91E-01	3.96E-02
OGDHL	1.83E+02	5.59E-04	4.46E-03	4.60E+00	2.20E+00	4.60E+00	1.53E+01	3.34E+00
SPICE1	5.54E+02	5.64E-04	4.46E-03	1.32E+00	4.01E-01	1.32E+00	3.29E+01	2.49E+01
SYTL5	1.53E+01	6.04E-04	4.74E-03	1.17E+01	3.55E+00	1.17E+01	1.42E+00	1.22E-01
LOC787740	1.48E+01	6.18E-04	4.81E-03	5.95E+00	2.57E+00	5.95E+00	1.28E+00	2.15E-01
BTN3A3	2.69E+00	6.79E-04	5.17E-03	3.84E+00	1.94E+00	3.84E+00	2.18E-01	5.67E-02
DOPEY2	2.93E+02	6.81E-04	5.17E-03	2.11E+00	1.08E+00	2.11E+00	2.05E+01	9.75E+00
LOC112447758	2.16E+00	6.83E-04	5.17E-03	7.17E+00	2.84E+00	7.17E+00	1.92E-01	2.68E-02
SGMS2	2.84E+02	7.55E-04	5.67E-03	1.73E+00	7.92E-01	1.73E+00	1.87E+01	1.08E+01
XR_001494794.2	1.48E+01	7.87E-04	5.86E-03	6.64E+00	2.73E+00	6.64E+00	1.30E+00	1.96E-01
LOC100298309	6.91E+00	7.93E-04	5.86E-03	5.96E+00	2.58E+00	5.96E+00	6.00E-01	1.01E-01
DTNBP1	4.42E+02	8.04E-04	5.89E-03	1.38E+00	4.69E-01	1.38E+00	2.68E+01	1.93E+01
TDRD10	1.65E+02	8.49E-04	6.16E-03	4.51E+00	2.17E+00	4.51E+00	1.37E+01	3.04E+00
XR_003032801.1	3.02E+00	8.96E-04	6.41E-03	1.26E+01	3.65E+00	1.26E+01	2.82E-01	2.24E-02
SNX10	6.72E+01	9.02E-04	6.41E-03	1.85E+00	8.84E-01	1.85E+00	4.52E+00	2.45E+00
XR_003031821.1	1.79E+01	9.10E-04	6.41E-03	4.32E+00	2.11E+00	4.32E+00	1.48E+00	3.43E-01
ENTPD4	7.77E+02	9.13E-04	6.41E-03	1.47E+00	5.60E-01	1.47E+00	4.82E+01	3.27E+01
PRKCQ	2.47E+02	9.19E-04	6.41E-03	3.74E+00	1.90E+00	3.74E+00	1.99E+01	5.33E+00
XR_003029452.1	5.48E+00	9.34E-04	6.46E-03	1.39E+01	3.80E+00	1.39E+01	5.15E-01	3.69E-02
PLCH1	1.97E+02	1.06E-03	7.29E-03	2.83E+00	1.50E+00	2.83E+00	1.49E+01	5.28E+00
COLEC10	1.09E+01	1.07E-03	7.31E-03	3.22E+00	1.69E+00	3.22E+00	8.54E-01	2.65E-01

XR_003035363.1	1.64E+01	1.16E-03	7.77E-03	7.36E+00	2.88E+00	7.36E+00	1.46E+00	1.98E-01
LOC101901970	5.80E+00	1.16E-03	7.77E-03	5.87E+00	2.55E+00	5.87E+00	5.03E-01	8.56E-02
XR_003036137.1	9.84E+01	1.22E-03	8.13E-03	5.02E+00	2.33E+00	5.02E+00	8.35E+00	1.66E+00
PLA2G4D	4.22E+01	1.24E-03	8.17E-03	2.68E+00	1.42E+00	2.68E+00	3.16E+00	1.18E+00
TECTB	1.73E+01	1.28E-03	8.37E-03	1.02E+01	3.35E+00	1.02E+01	1.59E+00	1.56E-01
ZNF804B	1.57E+01	1.39E-03	9.04E-03	4.90E+00	2.29E+00	4.90E+00	1.33E+00	2.71E-01
LOC617633	9.01E+00	1.40E-03	9.06E-03	7.11E+00	2.83E+00	7.11E+00	8.00E-01	1.12E-01
BBS9	1.74E+02	1.43E-03	9.17E-03	1.55E+00	6.28E-01	1.55E+00	1.10E+01	7.10E+00
XR_814925.3	2.16E+01	1.47E-03	9.30E-03	3.38E+00	1.76E+00	3.38E+00	1.71E+00	5.05E-01
XR_003032350.1	4.91E+01	1.47E-03	9.30E-03	2.05E+00	1.04E+00	2.05E+00	3.41E+00	1.66E+00
XR_003030656.1	1.05E+02	1.53E-03	9.53E-03	2.28E+00	1.19E+00	2.28E+00	7.54E+00	3.31E+00
ASAH2	1.42E+01	1.54E-03	9.53E-03	3.24E+00	1.70E+00	3.24E+00	1.11E+00	3.42E-01
XR_003031027.1	2.32E+00	1.54E-03	9.53E-03	1.39E+01	3.79E+00	1.39E+01	2.18E-01	1.57E-02
XR_003034304.1	1.14E+01	1.57E-03	9.62E-03	2.95E+00	1.56E+00	2.95E+00	8.76E-01	2.97E-01
LOC100296257	3.91E+01	1.60E-03	9.71E-03	4.85E+00	2.28E+00	4.85E+00	3.30E+00	6.79E-01
XR_003037432.1	1.10E+00	1.60E-03	9.71E-03	7.11E+00	2.83E+00	7.11E+00	9.72E-02	1.37E-02
CSF1R	8.65E+02	1.71E-03	1.03E-02	1.96E+00	9.71E-01	1.96E+00	5.93E+01	3.02E+01
XR_810104.3	2.48E+01	1.72E-03	1.03E-02	2.53E+00	1.34E+00	2.53E+00	1.83E+00	7.25E-01
ARMC2	1.08E+02	1.75E-03	1.04E-02	1.84E+00	8.82E-01	1.84E+00	7.25E+00	3.93E+00
BTG2	1.47E+03	1.77E-03	1.04E-02	1.52E+00	6.03E-01	1.52E+00	9.26E+01	6.09E+01
HHAT	5.60E+01	1.83E-03	1.07E-02	2.02E+00	1.02E+00	2.02E+00	3.87E+00	1.91E+00
XR_003032686.1	3.78E+00	1.87E-03	1.08E-02	3.59E+01	5.17E+00	3.59E+01	3.68E-01	1.03E-02
CAPN13	1.68E+01	1.87E-03	1.08E-02	3.80E+00	1.93E+00	3.80E+00	1.36E+00	3.58E-01
XR_003030060.1	1.31E+01	1.96E-03	1.11E-02	5.81E+00	2.54E+00	5.81E+00	1.13E+00	1.95E-01
BSPRY	9.81E+01	1.97E-03	1.11E-02	2.52E+00	1.34E+00	2.52E+00	7.23E+00	2.86E+00
LOC782385	1.55E+02	1.97E-03	1.11E-02	2.20E+00	1.14E+00	2.20E+00	1.10E+01	5.01E+00
CRNKL1	7.49E+02	1.99E-03	1.12E-02	1.28E+00	3.59E-01	1.28E+00	4.40E+01	3.43E+01
LINGO3	6.04E+01	2.01E-03	1.12E-02	3.87E+00	1.95E+00	3.87E+00	4.90E+00	1.27E+00
LOC112446768	4.70E+01	2.02E-03	1.12E-02	2.51E+00	1.33E+00	2.51E+00	3.46E+00	1.38E+00
ZNF177	4.27E+02	2.12E-03	1.16E-02	1.39E+00	4.72E-01	1.39E+00	2.59E+01	1.87E+01
PRPF4	1.00E+03	2.12E-03	1.16E-02	1.36E+00	4.43E-01	1.36E+00	6.02E+01	4.43E+01
CADM2	4.75E+01	2.12E-03	1.16E-02	1.89E+00	9.18E-01	1.89E+00	3.22E+00	1.70E+00
XR_003030812.1	6.23E+00	2.19E-03	1.18E-02	3.52E+00	1.82E+00	3.52E+00	4.96E-01	1.41E-01

AQP3	4.07E+01	2.21E-03	1.19E-02	3.01E+00	1.59E+00	3.01E+00	3.13E+00	1.04E+00
TMCC3	2.57E+02	2.23E-03	1.19E-02	1.77E+00	8.23E-01	1.77E+00	1.70E+01	9.62E+00
TSR1	1.04E+03	2.28E-03	1.21E-02	1.27E+00	3.47E-01	1.27E+00	6.08E+01	4.78E+01
LOC787257	3.18E+02	2.31E-03	1.22E-02	1.25E+00	3.17E-01	1.25E+00	1.85E+01	1.48E+01
XR_003031395.1	4.19E+01	2.34E-03	1.23E-02	4.56E+00	2.19E+00	4.56E+00	3.50E+00	7.67E-01
POU3F2	6.02E+00	2.41E-03	1.26E-02	6.67E+00	2.74E+00	6.67E+00	5.30E-01	7.95E-02
C6H4orf17	5.81E+00	2.43E-03	1.26E-02	7.07E+00	2.82E+00	7.07E+00	5.16E-01	7.29E-02
XR_003036065.1	1.36E+01	2.44E-03	1.26E-02	2.48E+00	1.31E+00	2.48E+00	9.99E-01	4.03E-01
XR_003032804.1	2.45E+00	2.47E-03	1.26E-02	5.75E+00	2.52E+00	5.75E+00	2.12E-01	3.69E-02
XR_003030981.1	1.41E+01	2.53E-03	1.29E-02	3.21E+00	1.68E+00	3.21E+00	1.10E+00	3.42E-01
XR_808313.3	7.42E+00	2.54E-03	1.29E-02	9.45E+00	3.24E+00	9.45E+00	6.77E-01	7.17E-02
LOC112447410	2.00E+00	2.62E-03	1.32E-02	4.22E+00	2.08E+00	4.22E+00	1.65E-01	3.91E-02
LOC107131273	1.51E+01	2.70E-03	1.35E-02	3.37E+00	1.75E+00	3.37E+00	1.19E+00	3.54E-01
HES5	5.88E+01	2.74E-03	1.37E-02	7.28E+00	2.86E+00	7.28E+00	5.24E+00	7.19E-01
MLN	3.28E+00	2.78E-03	1.37E-02	6.84E+00	2.77E+00	6.84E+00	2.90E-01	4.24E-02
XR_003033380.1	1.97E+01	2.93E-03	1.44E-02	5.16E+00	2.37E+00	5.16E+00	1.67E+00	3.24E-01
NRG3	2.73E+01	2.96E-03	1.45E-02	3.25E+00	1.70E+00	3.25E+00	2.13E+00	6.57E-01
XR_813704.3	6.22E+00	3.01E-03	1.46E-02	3.02E+00	1.59E+00	3.02E+00	4.79E-01	1.59E-01
XR_003037159.1	1.13E+00	3.07E-03	1.49E-02	1.67E+01	4.06E+00	1.67E+01	1.07E-01	6.42E-03
ABCG5	1.56E+01	3.11E-03	1.50E-02	5.93E+00	2.57E+00	5.93E+00	1.35E+00	2.28E-01
KCNH1	6.15E+01	3.15E-03	1.51E-02	3.46E+00	1.79E+00	3.46E+00	4.88E+00	1.41E+00
XR_003036542.1	1.43E+00	3.19E-03	1.51E-02	1.43E+01	3.83E+00	1.43E+01	1.34E-01	9.42E-03
CHST6	7.78E+01	3.20E-03	1.51E-02	2.54E+00	1.35E+00	2.54E+00	5.75E+00	2.26E+00
XR_003036054.1	5.79E+00	3.36E-03	1.58E-02	4.95E+00	2.31E+00	4.95E+00	4.90E-01	9.89E-02
LOC107132048	2.05E+02	3.36E-03	1.58E-02	2.13E+00	1.09E+00	2.13E+00	1.44E+01	6.77E+00
FBLL1	3.36E+01	3.45E-03	1.61E-02	1.76E+00	8.15E-01	1.76E+00	2.22E+00	1.26E+00
NRXN3	6.97E+01	3.50E-03	1.62E-02	2.59E+00	1.37E+00	2.59E+00	5.18E+00	2.00E+00
LOC107131851	5.39E+00	3.62E-03	1.67E-02	4.80E+00	2.26E+00	4.80E+00	4.54E-01	9.45E-02
HGSNAT	2.35E+03	3.81E-03	1.75E-02	1.16E+00	2.10E-01	1.16E+00	1.32E+02	1.14E+02
XR_003032065.1	5.15E+01	3.83E-03	1.75E-02	2.19E+00	1.13E+00	2.19E+00	3.65E+00	1.67E+00
LOC100140216	2.49E+00	4.08E-03	1.84E-02	4.93E+00	2.30E+00	4.93E+00	2.10E-01	4.27E-02
NOTO	2.53E+01	4.09E-03	1.84E-02	2.96E+00	1.57E+00	2.96E+00	1.94E+00	6.55E-01
XR_001501286.2	2.95E+00	4.09E-03	1.84E-02	1.25E+01	3.64E+00	1.25E+01	2.75E-01	2.21E-02

XR_003036313.1	1.10E+00	4.15E-03	1.85E-02	1.30E+01	3.70E+00	1.30E+01	1.03E-01	7.97E-03
LOC525426	1.31E+02	4.16E-03	1.85E-02	1.95E+00	9.67E-01	1.95E+00	8.94E+00	4.57E+00
NSMAF	4.61E+02	4.21E-03	1.86E-02	1.21E+00	2.72E-01	1.21E+00	2.64E+01	2.19E+01
SENP8	1.93E+02	4.43E-03	1.95E-02	1.96E+00	9.73E-01	1.96E+00	1.32E+01	6.73E+00
C15H11orf91	2.74E+00	4.45E-03	1.95E-02	7.29E+00	2.87E+00	7.29E+00	2.44E-01	3.34E-02
LOC616149	1.93E+00	4.58E-03	2.00E-02	3.96E+00	1.99E+00	3.96E+00	1.57E-01	3.97E-02
XR_003032748.1	2.01E+00	4.65E-03	2.02E-02	4.09E+00	2.03E+00	4.09E+00	1.65E-01	4.04E-02
NCF2	1.35E+02	4.80E-03	2.07E-02	2.11E+00	1.08E+00	2.11E+00	9.46E+00	4.48E+00
SHTN1	3.88E+02	5.21E-03	2.24E-02	1.45E+00	5.41E-01	1.45E+00	2.40E+01	1.65E+01
LOC112444524	8.02E+00	5.26E-03	2.25E-02	2.28E+00	1.19E+00	2.28E+00	5.75E-01	2.52E-01
MED14OS	1.69E+00	5.33E-03	2.27E-02	4.94E+00	2.30E+00	4.94E+00	1.43E-01	2.89E-02
CSDE1	8.26E+03	5.36E-03	2.27E-02	1.21E+00	2.77E-01	1.21E+00	4.74E+02	3.91E+02
KLHDC9	5.81E+01	5.42E-03	2.27E-02	1.89E+00	9.20E-01	1.89E+00	3.93E+00	2.08E+00
USP49	1.88E+02	5.43E-03	2.27E-02	1.86E+00	8.98E-01	1.86E+00	1.27E+01	6.79E+00
MCTP1	4.24E+00	5.54E-03	2.30E-02	5.82E+00	2.54E+00	5.82E+00	3.68E-01	6.32E-02
XR_003032264.1	3.02E+00	5.56E-03	2.30E-02	4.81E+00	2.26E+00	4.81E+00	2.55E-01	5.30E-02
A1BG	2.00E+01	5.63E-03	2.32E-02	7.06E+00	2.82E+00	7.06E+00	1.77E+00	2.51E-01
XR_003034418.1	3.21E+00	5.67E-03	2.33E-02	4.16E+00	2.05E+00	4.16E+00	2.63E-01	6.34E-02
MED12L	1.14E+02	5.77E-03	2.36E-02	2.56E+00	1.36E+00	2.56E+00	8.47E+00	3.30E+00
XR_003030108.1	2.02E+01	5.90E-03	2.39E-02	2.44E+00	1.29E+00	2.44E+00	1.47E+00	6.05E-01
XR_003031890.1	9.77E+00	5.91E-03	2.39E-02	4.49E+00	2.17E+00	4.49E+00	8.14E-01	1.81E-01
SLC34A1	2.70E+00	6.04E-03	2.44E-02	6.74E+00	2.75E+00	6.74E+00	2.38E-01	3.53E-02
CD58	2.46E+03	6.20E-03	2.49E-02	1.22E+00	2.86E-01	1.22E+00	1.42E+02	1.16E+02
ULK4	4.22E+02	6.27E-03	2.50E-02	1.81E+00	8.60E-01	1.81E+00	2.82E+01	1.56E+01
CPB2	6.73E+00	6.44E-03	2.56E-02	5.58E+00	2.48E+00	5.58E+00	5.80E-01	1.04E-01
XR_001502178.2	7.28E+00	6.70E-03	2.65E-02	1.06E+01	3.40E+00	1.06E+01	6.71E-01	6.33E-02
TSPAN5	3.80E+02	7.31E-03	2.88E-02	1.55E+00	6.35E-01	1.55E+00	2.41E+01	1.55E+01
XR_003031860.1	1.81E+02	7.43E-03	2.91E-02	1.65E+00	7.25E-01	1.65E+00	1.17E+01	7.11E+00
MAP3K8	1.11E+02	7.45E-03	2.91E-02	2.53E+00	1.34E+00	2.53E+00	8.22E+00	3.25E+00
PPFIA2	4.05E+01	7.51E-03	2.92E-02	2.57E+00	1.36E+00	2.57E+00	3.00E+00	1.17E+00
SOX7	4.15E+02	7.61E-03	2.93E-02	1.44E+00	5.29E-01	1.44E+00	2.56E+01	1.77E+01
XR_003037742.1	1.28E+01	7.61E-03	2.93E-02	6.51E+00	2.70E+00	6.51E+00	1.13E+00	1.73E-01
XR_003029493.1	1.79E+01	7.71E-03	2.96E-02	5.71E+00	2.51E+00	5.71E+00	1.55E+00	2.71E-01

SLC6A20	2.30E+02	7.79E-03	2.96E-02	2.23E+00	1.16E+00	2.23E+00	1.64E+01	7.35E+00
KNG1	2.77E+00	7.81E-03	2.96E-02	5.45E+00	2.45E+00	5.45E+00	2.38E-01	4.37E-02
LOC101904994	5.99E+00	7.82E-03	2.96E-02	3.74E+00	1.90E+00	3.74E+00	4.83E-01	1.29E-01
NLRC5	9.07E+01	7.88E-03	2.97E-02	2.21E+00	1.14E+00	2.21E+00	6.44E+00	2.92E+00
FGD6	1.33E+02	8.43E-03	3.17E-02	1.50E+00	5.87E-01	1.50E+00	8.33E+00	5.55E+00
DERL1	1.73E+03	8.52E-03	3.19E-02	1.14E+00	1.92E-01	1.14E+00	9.67E+01	8.46E+01
LOC112449365	1.66E+00	8.58E-03	3.19E-02	2.83E+00	1.50E+00	2.83E+00	1.26E-01	4.45E-02
ACTL7B	4.68E+00	8.80E-03	3.24E-02	2.77E+00	1.47E+00	2.77E+00	3.53E-01	1.27E-01
C1H3orf38	6.99E+02	8.84E-03	3.24E-02	1.18E+00	2.34E-01	1.18E+00	3.96E+01	3.37E+01
XR_003032239.1	1.34E+00	8.85E-03	3.24E-02	3.37E+01	5.07E+00	3.37E+01	1.30E-01	3.87E-03
MPHOSPH8	1.58E+03	8.85E-03	3.24E-02	1.27E+00	3.46E-01	1.27E+00	9.25E+01	7.28E+01
ALX1	5.07E+01	8.90E-03	3.24E-02	2.04E+00	1.03E+00	2.04E+00	3.52E+00	1.72E+00
MYF5	3.37E+00	9.16E-03	3.33E-02	4.00E+00	2.00E+00	4.00E+00	2.75E-01	6.88E-02
XR_003037904.1	1.79E+01	9.32E-03	3.37E-02	4.66E+00	2.22E+00	4.66E+00	1.50E+00	3.22E-01
XR_003030623.1	3.15E+01	9.40E-03	3.38E-02	1.59E+00	6.65E-01	1.59E+00	2.01E+00	1.27E+00
TTI2	1.04E+02	9.43E-03	3.38E-02	1.51E+00	5.90E-01	1.51E+00	6.48E+00	4.30E+00
XR_003032243.1	4.63E+00	9.53E-03	3.40E-02	1.59E+01	3.99E+00	1.59E+01	4.38E-01	2.76E-02
XR_001501955.2	2.73E+00	9.59E-03	3.41E-02	4.11E+00	2.04E+00	4.11E+00	2.24E-01	5.46E-02
SORCS1	5.52E+01	9.74E-03	3.45E-02	3.37E+00	1.75E+00	3.37E+00	4.36E+00	1.29E+00
SLC47A1	6.95E+01	9.86E-03	3.48E-02	2.67E+00	1.42E+00	2.67E+00	5.20E+00	1.94E+00
SYNRG	1.09E+03	1.01E-02	3.54E-02	1.37E+00	4.55E-01	1.37E+00	6.59E+01	4.81E+01
TMEM225B	6.30E+02	1.02E-02	3.55E-02	1.25E+00	3.18E-01	1.25E+00	3.66E+01	2.94E+01
EPB42	1.62E+01	1.02E-02	3.55E-02	3.34E+00	1.74E+00	3.34E+00	1.28E+00	3.82E-01
FYB2	2.27E+02	1.02E-02	3.55E-02	3.52E+00	1.81E+00	3.52E+00	1.81E+01	5.14E+00
XR_003033982.1	7.16E+00	1.04E-02	3.60E-02	2.43E+00	1.28E+00	2.43E+00	5.23E-01	2.15E-01
GDF9	1.12E+02	1.05E-02	3.63E-02	2.37E+00	1.25E+00	2.37E+00	8.11E+00	3.42E+00
XR_003032739.1	1.68E+01	1.06E-02	3.65E-02	2.34E+00	1.23E+00	2.34E+00	1.22E+00	5.20E-01
XR_812138.3	1.19E+01	1.08E-02	3.68E-02	4.98E+00	2.32E+00	4.98E+00	1.01E+00	2.02E-01
SLC7A9	2.11E+01	1.09E-02	3.70E-02	4.05E+00	2.02E+00	4.05E+00	1.73E+00	4.26E-01
DGAT2	2.63E+02	1.14E-02	3.87E-02	1.87E+00	9.01E-01	1.87E+00	1.77E+01	9.50E+00
XR_812731.3	4.64E+01	1.18E-02	3.99E-02	1.58E+00	6.64E-01	1.58E+00	2.96E+00	1.87E+00
CPN1	5.93E+01	1.20E-02	4.05E-02	2.59E+00	1.37E+00	2.59E+00	4.40E+00	1.70E+00
ICA1L	1.81E+02	1.21E-02	4.05E-02	3.82E+00	1.93E+00	3.82E+00	1.46E+01	3.82E+00

XR_003033104.1	7.76E+00	1.22E-02	4.07E-02	4.07E+00	2.03E+00	4.07E+00	6.36E-01	1.56E-01
XR_003032983.1	3.40E+01	1.24E-02	4.12E-02	2.02E+00	1.02E+00	2.02E+00	2.36E+00	1.17E+00
LOC509267	7.31E+00	1.25E-02	4.12E-02	2.31E+00	1.21E+00	2.31E+00	5.26E-01	2.27E-01
HPSE	1.31E+02	1.25E-02	4.12E-02	2.17E+00	1.12E+00	2.17E+00	9.28E+00	4.28E+00
PLCG2	7.06E+02	1.25E-02	4.12E-02	1.59E+00	6.72E-01	1.59E+00	4.51E+01	2.83E+01
XR_003029623.1	1.56E+01	1.27E-02	4.14E-02	2.07E+00	1.05E+00	2.07E+00	1.09E+00	5.24E-01
LOC526787	6.15E+00	1.29E-02	4.21E-02	3.57E+00	1.84E+00	3.57E+00	4.91E-01	1.38E-01
XR_001496995.2	2.10E+00	1.34E-02	4.35E-02	4.58E+00	2.20E+00	4.58E+00	1.75E-01	3.83E-02
GRM5	2.80E+01	1.36E-02	4.42E-02	3.12E+00	1.64E+00	3.12E+00	2.17E+00	6.96E-01
MYLK4	4.32E+01	1.39E-02	4.49E-02	2.09E+00	1.07E+00	2.09E+00	3.02E+00	1.44E+00
ERBB4	2.47E+01	1.41E-02	4.52E-02	3.93E+00	1.97E+00	3.93E+00	2.01E+00	5.11E-01
QRICH2	1.27E+02	1.41E-02	4.52E-02	1.62E+00	6.94E-01	1.62E+00	8.19E+00	5.06E+00
KRT31	2.00E+00	1.42E-02	4.52E-02	5.43E+00	2.44E+00	5.43E+00	1.72E-01	3.16E-02
TPST2	6.36E+02	1.46E-02	4.63E-02	1.30E+00	3.74E-01	1.30E+00	3.75E+01	2.90E+01
LOC107132928	1.45E+00	1.47E-02	4.64E-02	1.02E+01	3.35E+00	1.02E+01	1.33E-01	1.30E-02
RPH3AL	3.02E+02	1.48E-02	4.66E-02	1.94E+00	9.59E-01	1.94E+00	2.06E+01	1.06E+01
XR_003033103.1	1.00E+01	1.49E-02	4.68E-02	4.12E+00	2.04E+00	4.12E+00	8.23E-01	2.00E-01
XR_003034796.1	2.44E+00	1.50E-02	4.69E-02	1.36E+01	3.77E+00	1.36E+01	2.29E-01	1.68E-02
XR_003033061.1	2.91E+00	1.55E-02	4.84E-02	2.89E+03	1.15E+01	2.89E+03	2.91E-01	1.01E-04
LOC616427	1.81E+01	1.59E-02	4.94E-02	1.78E+00	8.35E-01	1.78E+00	1.20E+00	6.74E-01
XR_808518.3	2.05E+01	1.59E-02	4.94E-02	1.95E+00	9.65E-01	1.95E+00	1.40E+00	7.17E-01
SERPINA14	6.68E+00	1.60E-02	4.95E-02	3.36E+00	1.75E+00	3.36E+00	5.27E-01	1.57E-01
XR_003035712.1	3.75E+00	1.61E-02	4.95E-02	2.11E+00	1.08E+00	2.11E+00	2.63E-01	1.25E-01
XR_001494795.2	1.51E+01	1.63E-02	4.99E-02	2.24E+00	1.16E+00	2.24E+00	1.08E+00	4.82E-01
LRRIQ4	8.50E+00	1.63E-02	5.00E-02	3.68E+00	1.88E+00	3.68E+00	6.83E-01	1.86E-01
C8B	2.72E+01	1.67E-02	5.07E-02	2.54E+00	1.35E+00	2.54E+00	2.01E+00	7.91E-01
XR_003036937.1	8.25E+00	1.67E-02	5.07E-02	1.81E+00	8.58E-01	1.81E+00	5.51E-01	3.04E-01
SYCP2L	3.28E+01	1.69E-02	5.12E-02	4.10E+00	2.04E+00	4.10E+00	2.69E+00	6.56E-01
XR_812040.3	5.54E+00	1.70E-02	5.12E-02	3.25E+00	1.70E+00	3.25E+00	4.34E-01	1.34E-01
XR_804131.3	1.74E+01	1.72E-02	5.16E-02	4.79E+00	2.26E+00	4.79E+00	1.47E+00	3.07E-01
HERC3	3.57E+02	1.73E-02	5.17E-02	1.32E+00	4.01E-01	1.32E+00	2.12E+01	1.61E+01
MFSD4B	1.34E+02	1.78E-02	5.31E-02	1.55E+00	6.35E-01	1.55E+00	8.48E+00	5.46E+00
MAP7	2.73E+02	1.81E-02	5.38E-02	1.82E+00	8.63E-01	1.82E+00	1.83E+01	1.00E+01

LOC504207	1.27E+00	1.83E-02	5.42E-02	6.83E+00	2.77E+00	6.83E+00	1.12E-01	1.64E-02
C15H11orf16	2.02E+02	1.85E-02	5.45E-02	2.27E+00	1.18E+00	2.27E+00	1.45E+01	6.37E+00
SH2D2A	4.14E+01	1.85E-02	5.45E-02	1.60E+00	6.75E-01	1.60E+00	2.65E+00	1.66E+00
TIGD7	1.06E+02	1.87E-02	5.48E-02	1.36E+00	4.43E-01	1.36E+00	6.38E+00	4.70E+00
LOC107132300	2.23E+00	1.88E-02	5.49E-02	6.22E+00	2.64E+00	6.22E+00	1.95E-01	3.13E-02
EFCAB10	4.30E+01	2.02E-02	5.88E-02	2.27E+00	1.19E+00	2.27E+00	3.08E+00	1.35E+00
XR_003031394.1	2.28E+01	2.03E-02	5.88E-02	6.39E+00	2.68E+00	6.39E+00	1.99E+00	3.12E-01
XR_003037756.1	1.91E+00	2.03E-02	5.88E-02	2.24E+00	1.16E+00	2.24E+00	1.36E-01	6.08E-02
PRDM15	3.20E+02	2.04E-02	5.89E-02	1.43E+00	5.21E-01	1.43E+00	1.96E+01	1.37E+01
LOC104974272	7.25E+00	2.07E-02	5.94E-02	1.95E+00	9.63E-01	1.95E+00	4.96E-01	2.55E-01
LOC527981	2.00E+02	2.07E-02	5.94E-02	2.32E+00	1.22E+00	2.32E+00	1.44E+01	6.20E+00
RICTOR	3.95E+02	2.08E-02	5.94E-02	1.40E+00	4.83E-01	1.40E+00	2.40E+01	1.72E+01
XR_806817.3	2.36E+00	2.09E-02	5.94E-02	4.77E+00	2.25E+00	4.77E+00	1.98E-01	4.16E-02
XR_001494570.2	1.25E+00	2.12E-02	6.01E-02	4.92E+00	2.30E+00	4.92E+00	1.05E-01	2.14E-02
GGT6	1.72E+01	2.13E-02	6.01E-02	4.43E+00	2.15E+00	4.43E+00	1.43E+00	3.23E-01
ANKRD53	2.26E+01	2.15E-02	6.05E-02	1.67E+00	7.40E-01	1.67E+00	1.47E+00	8.80E-01
XR_003030037.1	2.16E+00	2.17E-02	6.09E-02	5.74E+00	2.52E+00	5.74E+00	1.87E-01	3.25E-02
CWC25	9.33E+01	2.17E-02	6.09E-02	1.35E+00	4.37E-01	1.35E+00	5.61E+00	4.14E+00
BHLHA9	4.46E+00	2.20E-02	6.13E-02	2.79E+00	1.48E+00	2.79E+00	3.37E-01	1.21E-01
LOC101906472	1.13E+01	2.20E-02	6.13E-02	4.30E+00	2.10E+00	4.30E+00	9.34E-01	2.17E-01
XR_003037448.1	1.24E+01	2.23E-02	6.17E-02	6.01E+00	2.59E+00	6.01E+00	1.08E+00	1.79E-01
XR_003030941.1	5.99E+01	2.23E-02	6.17E-02	1.59E+00	6.69E-01	1.59E+00	3.83E+00	2.41E+00
SNAI3	4.05E+01	2.29E-02	6.31E-02	2.41E+00	1.27E+00	2.41E+00	2.95E+00	1.22E+00
LOC112447087	3.27E+02	2.30E-02	6.31E-02	1.79E+00	8.44E-01	1.79E+00	2.18E+01	1.21E+01
PUSL1	1.22E+02	2.32E-02	6.34E-02	1.32E+00	4.04E-01	1.32E+00	7.27E+00	5.49E+00
XR_003031397.1	8.17E+01	2.33E-02	6.36E-02	2.12E+00	1.08E+00	2.12E+00	5.73E+00	2.70E+00
CARMIL2	1.76E+02	2.33E-02	6.36E-02	2.07E+00	1.05E+00	2.07E+00	1.23E+01	5.93E+00
C6H4orf50	2.05E+01	2.35E-02	6.36E-02	1.99E+00	9.93E-01	1.99E+00	1.41E+00	7.08E-01
UNC13B	1.05E+03	2.45E-02	6.62E-02	1.48E+00	5.70E-01	1.48E+00	6.54E+01	4.40E+01
NR_038195.1	1.99E+01	2.51E-02	6.77E-02	2.23E+00	1.16E+00	2.23E+00	1.42E+00	6.35E-01
XR_003030045.1	3.44E+00	2.56E-02	6.88E-02	2.12E+00	1.09E+00	2.12E+00	2.42E-01	1.14E-01
XR_003030705.1	6.81E+00	2.58E-02	6.89E-02	2.41E+00	1.27E+00	2.41E+00	4.96E-01	2.06E-01
TRAPPC8	8.18E+02	2.58E-02	6.89E-02	1.22E+00	2.89E-01	1.22E+00	4.71E+01	3.86E+01

ELP1	7.76E+02	2.60E-02	6.92E-02	1.34E+00	4.26E-01	1.34E+00	4.65E+01	3.46E+01
RBP7	2.76E+00	2.61E-02	6.92E-02	3.02E+00	1.59E+00	3.02E+00	2.12E-01	7.04E-02
LOC509884	1.14E+00	2.69E-02	7.12E-02	2.09E+01	4.38E+00	2.09E+01	1.09E-01	5.22E-03
XR_001494342.2	2.78E+00	2.70E-02	7.12E-02	6.23E+00	2.64E+00	6.23E+00	2.43E-01	3.90E-02
EXOC4	1.11E+03	2.71E-02	7.14E-02	1.20E+00	2.59E-01	1.20E+00	6.31E+01	5.27E+01
LOC112442545	1.98E+01	2.72E-02	7.14E-02	1.66E+00	7.29E-01	1.66E+00	1.28E+00	7.73E-01
GJA8	6.10E+00	2.73E-02	7.15E-02	2.36E+00	1.24E+00	2.36E+00	4.42E-01	1.87E-01
NOS1	1.47E+02	2.76E-02	7.18E-02	2.74E+00	1.46E+00	2.74E+00	1.10E+01	4.03E+00
COBL	6.94E+01	2.76E-02	7.19E-02	1.73E+00	7.94E-01	1.73E+00	4.57E+00	2.63E+00
SCEL	6.40E+00	2.78E-02	7.21E-02	3.76E+00	1.91E+00	3.76E+00	5.17E-01	1.37E-01
NHSL1	3.44E+02	2.84E-02	7.33E-02	1.51E+00	5.95E-01	1.51E+00	2.15E+01	1.43E+01
XR_003035705.1	8.30E+00	2.84E-02	7.33E-02	7.59E+00	2.92E+00	7.59E+00	7.42E-01	9.78E-02
LOC107131950	1.95E+00	2.86E-02	7.35E-02	3.18E+00	1.67E+00	3.18E+00	1.52E-01	4.78E-02
NAT10	1.51E+03	2.89E-02	7.38E-02	1.31E+00	3.95E-01	1.31E+00	8.96E+01	6.82E+01
MTNR1A	4.26E+00	2.89E-02	7.38E-02	8.98E+00	3.17E+00	8.98E+00	3.87E-01	4.31E-02
TRIM64	4.08E+00	2.96E-02	7.51E-02	7.74E+00	2.95E+00	7.74E+00	3.66E-01	4.72E-02
XR_003035190.1	1.68E+01	2.97E-02	7.51E-02	2.55E+00	1.35E+00	2.55E+00	1.24E+00	4.87E-01
KLHL3	2.36E+02	2.97E-02	7.51E-02	1.52E+00	6.04E-01	1.52E+00	1.48E+01	9.74E+00
HTR1B	1.12E+01	2.98E-02	7.51E-02	2.24E+00	1.16E+00	2.24E+00	8.00E-01	3.57E-01
MRS2	2.95E+02	2.98E-02	7.51E-02	1.24E+00	3.07E-01	1.24E+00	1.71E+01	1.38E+01
GPR84	7.59E+00	3.00E-02	7.54E-02	2.25E+00	1.17E+00	2.25E+00	5.42E-01	2.41E-01
XR_003032263.1	1.04E+01	3.03E-02	7.58E-02	2.06E+00	1.04E+00	2.06E+00	7.25E-01	3.53E-01
KDM4C	2.85E+02	3.06E-02	7.64E-02	1.18E+00	2.36E-01	1.18E+00	1.61E+01	1.37E+01
XR_003037892.1	5.04E+00	3.10E-02	7.72E-02	2.75E+00	1.46E+00	2.75E+00	3.79E-01	1.38E-01
SLC23A1	9.18E+01	3.15E-02	7.81E-02	1.61E+00	6.83E-01	1.61E+00	5.88E+00	3.66E+00
XR_003033448.1	2.95E+01	3.16E-02	7.81E-02	2.94E+00	1.56E+00	2.94E+00	2.26E+00	7.66E-01
FNDC7	6.07E+00	3.21E-02	7.90E-02	2.92E+00	1.55E+00	2.92E+00	4.64E-01	1.59E-01
RNF133	5.60E+00	3.21E-02	7.90E-02	1.77E+00	8.21E-01	1.77E+00	3.71E-01	2.10E-01
XR_003037547.1	9.64E+00	3.27E-02	8.03E-02	2.56E+00	1.36E+00	2.56E+00	7.14E-01	2.78E-01
CAND1	2.43E+03	3.31E-02	8.07E-02	1.18E+00	2.38E-01	1.18E+00	1.38E+02	1.17E+02
SLC28A2	3.59E+01	3.31E-02	8.07E-02	1.77E+00	8.23E-01	1.77E+00	2.38E+00	1.34E+00
LOC529036	1.20E+01	3.35E-02	8.16E-02	6.11E+00	2.61E+00	6.11E+00	1.05E+00	1.72E-01
ZACN	1.77E+00	3.36E-02	8.16E-02	2.50E+00	1.32E+00	2.50E+00	1.30E-01	5.20E-02

LOC112445019	1.30E+00	3.37E-02	8.16E-02	2.74E+00	1.45E+00	2.74E+00	9.77E-02	3.57E-02
LOC506121	1.31E+00	3.41E-02	8.22E-02	7.58E+00	2.92E+00	7.58E+00	1.17E-01	1.55E-02
SLC1A1	1.86E+01	3.42E-02	8.22E-02	2.55E+00	1.35E+00	2.55E+00	1.37E+00	5.39E-01
RWDD2A	1.76E+02	3.49E-02	8.36E-02	1.28E+00	3.57E-01	1.28E+00	1.04E+01	8.09E+00
GLRX	3.71E+02	3.51E-02	8.40E-02	1.47E+00	5.54E-01	1.47E+00	2.30E+01	1.57E+01
XR_003030796.1	5.89E+01	3.55E-02	8.46E-02	2.02E+00	1.02E+00	2.02E+00	4.08E+00	2.02E+00
HSH2D	2.17E+01	3.56E-02	8.47E-02	1.66E+00	7.33E-01	1.66E+00	1.40E+00	8.45E-01
SUPT3H	6.59E+02	3.60E-02	8.54E-02	1.18E+00	2.37E-01	1.18E+00	3.74E+01	3.17E+01
ERMP1	6.91E+02	3.63E-02	8.59E-02	1.32E+00	4.06E-01	1.32E+00	4.11E+01	3.11E+01
USP13	4.12E+02	3.64E-02	8.59E-02	1.25E+00	3.24E-01	1.25E+00	2.39E+01	1.91E+01
XR_001495971.2	2.70E+00	3.67E-02	8.63E-02	5.44E+00	2.44E+00	5.44E+00	2.32E-01	4.26E-02
CCDC42	2.09E+02	3.68E-02	8.63E-02	2.07E+00	1.05E+00	2.07E+00	1.45E+01	7.04E+00
XR_003034336.1	5.24E+00	3.68E-02	8.63E-02	1.70E+00	7.68E-01	1.70E+00	3.43E-01	2.01E-01
DDHD1	4.55E+02	3.70E-02	8.63E-02	1.27E+00	3.39E-01	1.27E+00	2.66E+01	2.10E+01
XR_812060.3	4.21E+00	3.75E-02	8.73E-02	1.84E+00	8.79E-01	1.84E+00	2.83E-01	1.54E-01
XR_003032242.1	1.80E+00	3.77E-02	8.75E-02	6.85E+00	2.78E+00	6.85E+00	1.60E-01	2.33E-02
NF1	5.25E+00	3.78E-02	8.75E-02	2.40E+00	1.26E+00	2.40E+00	3.81E-01	1.59E-01
XR_812473.3	1.85E+00	3.85E-02	8.91E-02	2.50E+00	1.32E+00	2.50E+00	1.36E-01	5.45E-02
ANO10	6.09E+02	3.88E-02	8.93E-02	1.23E+00	3.02E-01	1.23E+00	3.52E+01	2.86E+01
SMURF1	1.35E+03	3.96E-02	9.08E-02	1.45E+00	5.41E-01	1.45E+00	8.34E+01	5.74E+01
CCDC171	1.21E+02	3.96E-02	9.08E-02	1.42E+00	5.02E-01	1.42E+00	7.43E+00	5.24E+00
XR_003030709.1	1.19E+00	4.15E-02	9.49E-02	4.51E+00	2.17E+00	4.51E+00	9.92E-02	2.20E-02
XR_806191.2	2.22E+00	4.21E-02	9.61E-02	4.37E+00	2.13E+00	4.37E+00	1.84E-01	4.21E-02
STK3	8.12E+02	4.23E-02	9.62E-02	1.20E+00	2.60E-01	1.20E+00	4.64E+01	3.87E+01
LOC523431	4.61E+00	4.25E-02	9.64E-02	2.92E+00	1.54E+00	2.92E+00	3.53E-01	1.21E-01
RAPH1	7.19E+02	4.34E-02	9.79E-02	1.35E+00	4.28E-01	1.35E+00	4.31E+01	3.20E+01
MTHFD1	1.53E+03	4.34E-02	9.79E-02	1.19E+00	2.56E-01	1.19E+00	8.71E+01	7.29E+01
GSX2	4.06E+00	4.35E-02	9.79E-02	3.25E+00	1.70E+00	3.25E+00	3.18E-01	9.77E-02
AKNAD1	1.81E+01	4.37E-02	9.81E-02	3.24E+00	1.70E+00	3.24E+00	1.42E+00	4.37E-01
GPR52	2.84E+00	4.39E-02	9.82E-02	2.57E+00	1.36E+00	2.57E+00	2.10E-01	8.18E-02
XR_001500112.2	1.04E+01	4.41E-02	9.85E-02	1.94E+00	9.58E-01	1.94E+00	7.14E-01	3.67E-01
LOC101903953	1.05E+01	4.45E-02	9.90E-02	2.63E+00	1.40E+00	2.63E+00	7.82E-01	2.97E-01
XR_001501639.2	2.27E+00	4.54E-02	1.01E-01	3.11E+00	1.64E+00	3.11E+00	1.76E-01	5.66E-02

LOC100138454	1.98E+00	4.54E-02	1.01E-01	1.85E+00	8.87E-01	1.85E+00	1.33E-01	7.22E-02
LOC101906595	2.11E+00	4.55E-02	1.01E-01	3.20E+00	1.68E+00	3.20E+00	1.65E-01	5.14E-02
IL1R2	6.55E+00	4.60E-02	1.01E-01	2.23E+00	1.16E+00	2.23E+00	4.67E-01	2.09E-01
XR_003035679.1	2.35E+00	4.61E-02	1.01E-01	3.00E+00	1.58E+00	3.00E+00	1.81E-01	6.03E-02
LOC785036	1.85E+00	4.66E-02	1.02E-01	1.64E+01	4.03E+00	1.64E+01	1.75E-01	1.07E-02
SERPINA6	6.84E+00	4.68E-02	1.02E-01	1.35E+00	4.30E-01	1.35E+00	4.10E-01	3.04E-01
XR_003035851.1	8.74E+00	4.75E-02	1.04E-01	2.73E+00	1.45E+00	2.73E+00	6.57E-01	2.41E-01
GDI2	6.87E+03	4.77E-02	1.04E-01	1.11E+00	1.50E-01	1.11E+00	3.79E+02	3.42E+02
CLIP4	9.49E+01	4.79E-02	1.04E-01	1.60E+00	6.82E-01	1.60E+00	6.08E+00	3.79E+00
LOC618124	1.15E+00	4.82E-02	1.04E-01	9.24E+00	3.21E+00	9.24E+00	1.04E-01	1.13E-02
PLA2G12B	4.07E+00	4.84E-02	1.05E-01	2.23E+00	1.15E+00	2.23E+00	2.90E-01	1.30E-01
DAPK2	2.23E+02	4.89E-02	1.06E-01	1.25E+00	3.17E-01	1.25E+00	1.30E+01	1.04E+01
ADAD1	2.52E+02	4.91E-02	1.06E-01	2.11E+00	1.08E+00	2.11E+00	1.77E+01	8.38E+00
GPR39	5.86E+01	4.92E-02	1.06E-01	1.72E+00	7.80E-01	1.72E+00	3.85E+00	2.24E+00
XR_003034709.1	3.55E+00	5.04E-02	1.08E-01	2.53E+00	1.34E+00	2.53E+00	2.62E-01	1.03E-01
FAM21A	1.51E+03	5.09E-02	1.08E-01	1.16E+00	2.13E-01	1.16E+00	8.49E+01	7.32E+01
LOC100337053	1.52E+01	5.09E-02	1.08E-01	2.40E+00	1.26E+00	2.40E+00	1.10E+00	4.59E-01
XR_003035120.1	1.55E+00	5.10E-02	1.08E-01	2.60E+01	4.70E+00	2.60E+01	1.50E-01	5.76E-03
XR_003037120.1	2.55E+00	5.11E-02	1.08E-01	4.32E+00	2.11E+00	4.32E+00	2.11E-01	4.87E-02
XR_001500662.2	1.08E+01	5.11E-02	1.08E-01	6.73E+00	2.75E+00	6.73E+00	9.56E-01	1.42E-01
XR_003038077.1	5.21E+00	5.12E-02	1.08E-01	2.07E+00	1.05E+00	2.07E+00	3.63E-01	1.76E-01
XR_003037237.1	2.17E+00	5.20E-02	1.09E-01	5.08E+00	2.35E+00	5.08E+00	1.84E-01	3.62E-02
XR_003037220.1	9.76E+00	5.23E-02	1.10E-01	4.32E+00	2.11E+00	4.32E+00	8.07E-01	1.87E-01
TTC39C	2.02E+02	5.35E-02	1.12E-01	1.35E+00	4.37E-01	1.35E+00	1.21E+01	8.95E+00
LOC107132230	3.29E+00	5.36E-02	1.12E-01	2.51E+00	1.33E+00	2.51E+00	2.42E-01	9.65E-02
XR_003036833.1	3.43E+00	5.46E-02	1.14E-01	3.55E+00	1.83E+00	3.55E+00	2.73E-01	7.71E-02
HIGD1C	6.93E+00	5.49E-02	1.14E-01	2.23E+00	1.15E+00	2.23E+00	4.94E-01	2.22E-01
LOC112445104	8.59E+00	5.71E-02	1.18E-01	1.11E+00	1.54E-01	1.11E+00	4.75E-01	4.27E-01
HRCT1	1.65E+02	5.75E-02	1.19E-01	1.31E+00	3.88E-01	1.31E+00	9.75E+00	7.45E+00
XR_237971.4	2.18E+01	5.76E-02	1.19E-01	1.52E+00	6.06E-01	1.52E+00	1.37E+00	9.02E-01
SLC17A4	2.69E+00	5.77E-02	1.19E-01	1.21E+01	3.60E+00	1.21E+01	2.50E-01	2.07E-02
MEX3C	1.87E+03	5.84E-02	1.20E-01	1.21E+00	2.76E-01	1.21E+00	1.07E+02	8.86E+01
CLDN23	1.29E+01	5.90E-02	1.21E-01	2.52E+00	1.33E+00	2.52E+00	9.51E-01	3.77E-01

XR_001500630.2	2.30E+00	5.99E-02	1.23E-01	1.65E+00	7.24E-01	1.65E+00	1.49E-01	9.02E-02
APBA1	7.30E+02	6.12E-02	1.25E-01	1.50E+00	5.83E-01	1.50E+00	4.56E+01	3.04E+01
XR_003029667.1	2.55E+00	6.14E-02	1.25E-01	1.80E+00	8.51E-01	1.80E+00	1.70E-01	9.45E-02
CPEB3	2.36E+02	6.17E-02	1.25E-01	1.49E+00	5.78E-01	1.49E+00	1.48E+01	9.88E+00
PTPRQ	1.73E+01	6.22E-02	1.26E-01	2.73E+00	1.45E+00	2.73E+00	1.30E+00	4.77E-01
XR_003030783.1	3.24E+00	6.25E-02	1.26E-01	3.16E+00	1.66E+00	3.16E+00	2.52E-01	8.00E-02
XR_001494442.2	1.97E+00	6.29E-02	1.27E-01	4.09E+00	2.03E+00	4.09E+00	1.62E-01	3.95E-02
CFAP58	1.81E+01	6.43E-02	1.29E-01	2.10E+00	1.07E+00	2.10E+00	1.27E+00	6.04E-01
SLC36A1	3.49E+02	6.53E-02	1.31E-01	1.33E+00	4.06E-01	1.33E+00	2.08E+01	1.57E+01
XR_003030235.1	2.48E+00	6.55E-02	1.31E-01	2.37E+01	4.57E+00	2.37E+01	2.39E-01	1.00E-02
XR_001498507.2	4.97E+00	6.59E-02	1.32E-01	3.09E+00	1.63E+00	3.09E+00	3.85E-01	1.25E-01
LOC100337152	1.10E+01	6.62E-02	1.32E-01	1.49E+00	5.76E-01	1.49E+00	6.88E-01	4.61E-01
XR_003034667.1	2.38E+00	6.65E-02	1.32E-01	1.82E+00	8.63E-01	1.82E+00	1.59E-01	8.74E-02
TENM1	1.54E+02	6.66E-02	1.32E-01	1.72E+00	7.82E-01	1.72E+00	1.01E+01	5.86E+00
ZNF182	1.75E+02	6.73E-02	1.33E-01	1.23E+00	2.96E-01	1.23E+00	1.01E+01	8.24E+00
LOC785617	7.60E+00	6.73E-02	1.33E-01	2.16E+00	1.11E+00	2.16E+00	5.36E-01	2.49E-01
CRH	5.46E+01	6.80E-02	1.34E-01	1.58E+00	6.60E-01	1.58E+00	3.48E+00	2.20E+00
ZNF648	1.46E+00	6.80E-02	1.34E-01	2.98E+00	1.58E+00	2.98E+00	1.12E-01	3.76E-02
B3GALT2	1.50E+02	6.84E-02	1.34E-01	1.37E+00	4.52E-01	1.37E+00	9.07E+00	6.63E+00
ASMT	2.35E+01	6.85E-02	1.34E-01	1.52E+00	6.06E-01	1.52E+00	1.48E+00	9.71E-01
C16H1orf105	9.46E+01	6.88E-02	1.34E-01	1.45E+00	5.36E-01	1.45E+00	5.83E+00	4.02E+00
C7H19orf67	4.90E+01	7.01E-02	1.37E-01	1.70E+00	7.61E-01	1.70E+00	3.20E+00	1.89E+00
PARG	7.70E+02	7.04E-02	1.37E-01	1.16E+00	2.09E-01	1.16E+00	4.33E+01	3.75E+01
TRIM45	3.70E+02	7.06E-02	1.37E-01	1.29E+00	3.72E-01	1.29E+00	2.18E+01	1.69E+01
ZNF304	3.87E+02	7.32E-02	1.42E-01	1.16E+00	2.14E-01	1.16E+00	2.18E+01	1.88E+01
LOC615183	3.03E+01	7.46E-02	1.44E-01	2.07E+00	1.05E+00	2.07E+00	2.11E+00	1.02E+00
LOC112449108	1.31E+01	7.54E-02	1.45E-01	1.88E+00	9.12E-01	1.88E+00	8.86E-01	4.71E-01
LOC788599	6.21E+01	7.62E-02	1.47E-01	1.35E+00	4.36E-01	1.35E+00	3.73E+00	2.76E+00
PHTF1	4.37E+02	7.75E-02	1.48E-01	1.19E+00	2.50E-01	1.19E+00	2.49E+01	2.09E+01
XR_233302.4	3.27E+00	7.75E-02	1.48E-01	2.84E+00	1.51E+00	2.84E+00	2.48E-01	8.73E-02
XR_003036862.1	1.04E+01	7.77E-02	1.48E-01	1.47E+00	5.60E-01	1.47E+00	6.48E-01	4.39E-01
XR_003037246.1	4.26E+00	7.77E-02	1.48E-01	2.36E+00	1.24E+00	2.36E+00	3.09E-01	1.30E-01
XR_003030782.1	7.18E+00	7.92E-02	1.50E-01	4.77E+00	2.25E+00	4.77E+00	6.04E-01	1.27E-01

NKX1-1	2.61E+00	7.92E-02	1.50E-01	3.64E+00	1.86E+00	3.64E+00	2.09E-01	5.75E-02
XR_003035096.1	3.46E-01	7.97E-02	1.51E-01	7.04E+01	6.14E+00	7.04E+01	3.42E-02	4.86E-04
ATRNL1	7.31E+02	8.01E-02	1.51E-01	1.28E+00	3.52E-01	1.28E+00	4.29E+01	3.36E+01
LOC112443761	8.02E+00	8.05E-02	1.51E-01	2.07E+00	1.05E+00	2.07E+00	5.59E-01	2.70E-01
TP63	4.35E+01	8.05E-02	1.51E-01	2.72E+00	1.44E+00	2.72E+00	3.27E+00	1.20E+00
XR_003035121.1	2.25E+00	8.09E-02	1.52E-01	4.71E+01	5.56E+00	4.71E+01	2.20E-01	4.68E-03
LOC100336282	9.69E+01	8.24E-02	1.54E-01	1.61E+00	6.89E-01	1.61E+00	6.22E+00	3.86E+00
TDRD15	4.52E+01	8.34E-02	1.56E-01	1.91E+00	9.37E-01	1.91E+00	3.08E+00	1.61E+00
CCL17	5.07E+00	8.62E-02	1.61E-01	1.68E+00	7.50E-01	1.68E+00	3.30E-01	1.96E-01
XR_003031463.1	2.05E+00	8.71E-02	1.62E-01	5.14E+00	2.36E+00	5.14E+00	1.74E-01	3.39E-02
LRRC51	1.24E+02	8.76E-02	1.63E-01	1.60E+00	6.80E-01	1.60E+00	7.97E+00	4.97E+00
CHL1	4.70E+02	8.90E-02	1.65E-01	2.10E+00	1.07E+00	2.10E+00	3.29E+01	1.56E+01
XR_001494316.2	1.59E+01	8.94E-02	1.65E-01	1.57E+00	6.53E-01	1.57E+00	1.01E+00	6.44E-01
XR_003035103.1	5.22E+00	8.94E-02	1.65E-01	2.32E+00	1.21E+00	2.32E+00	3.76E-01	1.62E-01
SLC17A8	8.57E+00	8.98E-02	1.65E-01	2.93E+00	1.55E+00	2.93E+00	6.56E-01	2.24E-01
DRD2	3.85E+00	9.04E-02	1.66E-01	2.21E+00	1.15E+00	2.21E+00	2.74E-01	1.24E-01
XR_001500537.2	1.93E+00	9.05E-02	1.66E-01	4.84E+00	2.27E+00	4.84E+00	1.63E-01	3.36E-02
LOC112444595	4.03E+01	9.18E-02	1.68E-01	7.86E-01	-3.48E-01	-1.27E+00	1.88E+00	2.39E+00
TMEM131	2.12E+03	9.25E-02	1.69E-01	1.14E+00	1.91E-01	1.14E+00	1.19E+02	1.04E+02
PCDHB1	1.28E+01	9.27E-02	1.69E-01	2.30E+00	1.20E+00	2.30E+00	9.20E-01	4.00E-01
NELL1	3.53E+01	9.33E-02	1.70E-01	2.23E+00	1.16E+00	2.23E+00	2.52E+00	1.13E+00
GRHPR	7.78E+02	9.38E-02	1.70E-01	1.26E+00	3.29E-01	1.26E+00	4.53E+01	3.61E+01
CCDC88A	9.87E+02	9.38E-02	1.70E-01	1.16E+00	2.15E-01	1.16E+00	5.56E+01	4.79E+01
XR_003030447.1	7.05E+00	9.41E-02	1.70E-01	1.86E+00	8.96E-01	1.86E+00	4.75E-01	2.55E-01
MALRD1	5.59E+01	9.56E-02	1.72E-01	2.69E+00	1.43E+00	2.69E+00	4.19E+00	1.56E+00
LRMDA	3.72E+01	9.57E-02	1.72E-01	1.36E+00	4.47E-01	1.36E+00	2.24E+00	1.64E+00
SCG3	1.54E+01	9.64E-02	1.73E-01	1.78E+00	8.30E-01	1.78E+00	1.03E+00	5.77E-01
B3GNT4	7.94E+01	9.81E-02	1.76E-01	1.78E+00	8.28E-01	1.78E+00	5.27E+00	2.97E+00
TOR2A	2.61E+02	9.87E-02	1.76E-01	1.31E+00	3.85E-01	1.31E+00	1.54E+01	1.18E+01
XR_233990.4	3.48E+00	9.89E-02	1.77E-01	1.72E+00	7.84E-01	1.72E+00	2.29E-01	1.33E-01
CD177	1.67E+01	1.00E-01	1.78E-01	2.01E+00	1.01E+00	2.01E+00	1.15E+00	5.73E-01
XR_003034451.1	5.46E+01	1.01E-01	1.79E-01	1.33E+00	4.10E-01	1.33E+00	3.26E+00	2.45E+00
XR_003036476.1	2.30E+01	1.01E-01	1.80E-01	2.12E+00	1.08E+00	2.12E+00	1.62E+00	7.64E-01

KIF28	4.65E+00	1.04E-01	1.85E-01	1.61E+00	6.87E-01	1.61E+00	2.98E-01	1.85E-01
MAATS1	6.87E+01	1.05E-01	1.85E-01	1.65E+00	7.21E-01	1.65E+00	4.44E+00	2.70E+00
RASGRP1	1.38E+02	1.05E-01	1.85E-01	1.59E+00	6.66E-01	1.59E+00	8.79E+00	5.54E+00
LOC530211	3.62E+01	1.05E-01	1.85E-01	1.32E+00	3.97E-01	1.32E+00	2.15E+00	1.63E+00
MFRP	1.16E+01	1.06E-01	1.85E-01	3.16E+00	1.66E+00	3.16E+00	9.04E-01	2.86E-01
MGC137454	1.57E+00	1.06E-01	1.85E-01	2.19E+00	1.13E+00	2.19E+00	1.11E-01	5.08E-02
MBOAT1	2.41E+02	1.07E-01	1.86E-01	1.24E+00	3.15E-01	1.24E+00	1.40E+01	1.12E+01
XR_003033848.1	3.75E+01	1.07E-01	1.86E-01	2.89E+00	1.53E+00	2.89E+00	2.86E+00	9.89E-01
XR_003037318.1	1.43E+00	1.08E-01	1.88E-01	6.63E+01	6.05E+00	6.63E+01	1.41E-01	2.13E-03
XR_003037750.1	9.87E+00	1.09E-01	1.89E-01	1.87E+00	9.04E-01	1.87E+00	6.67E-01	3.56E-01
EYA2	4.70E+01	1.10E-01	1.91E-01	2.05E+00	1.04E+00	2.05E+00	3.27E+00	1.59E+00
KALRN	9.15E+02	1.11E-01	1.92E-01	1.21E+00	2.76E-01	1.21E+00	5.25E+01	4.33E+01
LOC107132735	1.07E+01	1.13E-01	1.95E-01	1.43E+00	5.19E-01	1.43E+00	6.59E-01	4.60E-01
XR_003031396.1	6.66E+00	1.17E-01	2.01E-01	2.75E+00	1.46E+00	2.75E+00	5.02E-01	1.83E-01
KIF2A	3.58E+02	1.17E-01	2.02E-01	1.20E+00	2.67E-01	1.20E+00	2.05E+01	1.70E+01
ARHGEF33	3.99E+01	1.21E-01	2.07E-01	1.89E+00	9.15E-01	1.89E+00	2.70E+00	1.43E+00
XR_001494879.2	1.01E+01	1.22E-01	2.09E-01	2.24E+00	1.16E+00	2.24E+00	7.23E-01	3.23E-01
TTLL4	7.92E+02	1.23E-01	2.11E-01	1.26E+00	3.31E-01	1.26E+00	4.62E+01	3.67E+01
ATOH7	5.75E+00	1.24E-01	2.11E-01	2.18E+00	1.12E+00	2.18E+00	4.07E-01	1.87E-01
SETD2	2.35E+03	1.26E-01	2.15E-01	1.17E+00	2.26E-01	1.17E+00	1.33E+02	1.13E+02
XR_814952.3	5.04E-01	1.26E-01	2.15E-01	1.03E+02	6.68E+00	1.03E+02	5.00E-02	4.86E-04
XR_003033532.1	3.20E+00	1.28E-01	2.17E-01	2.84E+00	1.51E+00	2.84E+00	2.43E-01	8.56E-02
XR_003037902.1	1.30E+00	1.29E-01	2.18E-01	2.47E+00	1.30E+00	2.47E+00	9.55E-02	3.87E-02
VRTN	1.85E+02	1.32E-01	2.23E-01	9.05E-01	-1.45E-01	-1.11E+00	9.29E+00	1.03E+01
SLC12A2	2.73E+02	1.32E-01	2.23E-01	1.25E+00	3.26E-01	1.25E+00	1.59E+01	1.27E+01
GSDMC	3.25E+00	1.34E-01	2.25E-01	2.18E+00	1.13E+00	2.18E+00	2.30E-01	1.06E-01
SHISA9	5.79E+01	1.36E-01	2.29E-01	1.99E+00	9.90E-01	1.99E+00	3.98E+00	2.01E+00
XR_003031258.1	3.13E+00	1.37E-01	2.30E-01	1.71E+00	7.78E-01	1.71E+00	2.05E-01	1.20E-01
XR_001502079.2	2.83E+01	1.38E-01	2.30E-01	1.46E+00	5.46E-01	1.46E+00	1.75E+00	1.20E+00
LOC785712	8.17E-01	1.38E-01	2.31E-01	2.34E+00	1.23E+00	2.34E+00	5.90E-02	2.52E-02
CIT	1.25E+03	1.39E-01	2.31E-01	1.20E+00	2.64E-01	1.20E+00	7.15E+01	5.95E+01
CDH26	2.37E+00	1.42E-01	2.37E-01	4.49E+00	2.17E+00	4.49E+00	1.97E-01	4.39E-02
HRG	3.22E+00	1.43E-01	2.38E-01	1.85E+00	8.90E-01	1.85E+00	2.17E-01	1.17E-01

THSD7B	4.68E+01	1.44E-01	2.38E-01	1.67E+00	7.38E-01	1.67E+00	3.04E+00	1.82E+00
SLC45A1	1.34E+01	1.46E-01	2.41E-01	1.51E+00	5.93E-01	1.51E+00	8.36E-01	5.54E-01
XR_816662.3	1.65E+01	1.46E-01	2.41E-01	5.20E+00	2.38E+00	5.20E+00	1.40E+00	2.70E-01
PRKDC	8.83E+02	1.46E-01	2.41E-01	1.19E+00	2.52E-01	1.19E+00	5.03E+01	4.22E+01
RAB27A	2.39E+02	1.46E-01	2.41E-01	1.20E+00	2.58E-01	1.20E+00	1.36E+01	1.14E+01
LYPD6B	3.19E+01	1.48E-01	2.42E-01	2.04E+00	1.03E+00	2.04E+00	2.21E+00	1.09E+00
SMARCAL1	1.70E+03	1.48E-01	2.44E-01	1.19E+00	2.53E-01	1.19E+00	9.71E+01	8.15E+01
MGC138914	1.97E+00	1.49E-01	2.44E-01	8.86E-01	-1.74E-01	-1.13E+00	9.75E-02	1.10E-01
XR_003031815.1	2.29E+00	1.50E-01	2.45E-01	2.13E+00	1.09E+00	2.13E+00	1.61E-01	7.58E-02
TRIM24	1.48E+03	1.54E-01	2.51E-01	1.19E+00	2.50E-01	1.19E+00	8.40E+01	7.07E+01
GDAP2	2.24E+02	1.56E-01	2.54E-01	1.14E+00	1.93E-01	1.14E+00	1.25E+01	1.10E+01
XR_003032559.1	9.74E+00	1.57E-01	2.55E-01	1.71E+00	7.76E-01	1.71E+00	6.38E-01	3.73E-01
XR_003031850.1	1.57E+00	1.57E-01	2.55E-01	3.12E+00	1.64E+00	3.12E+00	1.22E-01	3.90E-02
XR_234317.4	1.48E+01	1.59E-01	2.56E-01	1.62E+00	6.93E-01	1.62E+00	9.52E-01	5.89E-01
SCLT1	1.49E+02	1.59E-01	2.56E-01	1.21E+00	2.74E-01	1.21E+00	8.55E+00	7.07E+00
XR_003032471.1	2.91E+00	1.59E-01	2.56E-01	1.68E+00	7.50E-01	1.68E+00	1.90E-01	1.13E-01
STXBP6	5.29E+02	1.59E-01	2.57E-01	1.26E+00	3.38E-01	1.26E+00	3.09E+01	2.45E+01
ADORA2B	7.85E+01	1.60E-01	2.57E-01	1.22E+00	2.84E-01	1.22E+00	4.51E+00	3.71E+00
XR_003038209.1	5.55E+00	1.60E-01	2.57E-01	4.26E+00	2.09E+00	4.26E+00	4.58E-01	1.07E-01
USH2A	4.30E+01	1.62E-01	2.59E-01	1.49E+00	5.79E-01	1.49E+00	2.68E+00	1.80E+00
PRSS38	1.30E+01	1.63E-01	2.61E-01	1.55E+00	6.31E-01	1.55E+00	8.23E-01	5.31E-01
XR_003030353.1	7.81E+01	1.67E-01	2.66E-01	1.33E+00	4.17E-01	1.33E+00	4.67E+00	3.50E+00
RAD50	2.09E+03	1.68E-01	2.67E-01	1.35E+00	4.29E-01	1.35E+00	1.25E+02	9.32E+01
MFN2	1.63E+03	1.68E-01	2.67E-01	1.14E+00	1.87E-01	1.14E+00	9.08E+01	7.97E+01
GPR12	9.27E+00	1.69E-01	2.67E-01	2.42E+00	1.27E+00	2.42E+00	6.75E-01	2.79E-01
XR_003038187.1	8.21E+00	1.69E-01	2.67E-01	6.26E+00	2.65E+00	6.26E+00	7.18E-01	1.15E-01
PDCD6IP	3.66E+03	1.69E-01	2.67E-01	1.17E+00	2.25E-01	1.17E+00	2.07E+02	1.77E+02
ZDHHC15	1.06E+02	1.70E-01	2.68E-01	1.19E+00	2.52E-01	1.19E+00	6.02E+00	5.05E+00
XR_814551.3	6.10E+00	1.72E-01	2.72E-01	3.41E+00	1.77E+00	3.41E+00	4.83E-01	1.42E-01
XR_235188.4	1.63E+01	1.73E-01	2.72E-01	1.63E+00	7.07E-01	1.63E+00	1.05E+00	6.44E-01
NCBP1	1.06E+03	1.75E-01	2.74E-01	1.12E+00	1.62E-01	1.12E+00	5.89E+01	5.26E+01
DYNC1I2	1.89E+03	1.77E-01	2.77E-01	1.08E+00	1.05E-01	1.08E+00	1.03E+02	9.57E+01
LOC100140356	3.13E+00	1.77E-01	2.77E-01	1.66E+00	7.30E-01	1.66E+00	2.03E-01	1.22E-01

GRAMD2A	3.06E+01	1.81E-01	2.82E-01	1.90E+00	9.25E-01	1.90E+00	2.07E+00	1.09E+00
LOC112447011	3.03E+02	1.83E-01	2.84E-01	1.49E+00	5.80E-01	1.49E+00	1.89E+01	1.27E+01
MSX1	9.12E+01	1.83E-01	2.85E-01	1.22E+00	2.84E-01	1.22E+00	5.24E+00	4.31E+00
XR_003033925.1	1.43E+00	1.86E-01	2.88E-01	9.47E-01	-7.82E-02	-1.06E+00	7.33E-02	7.74E-02
XR_003033279.1	1.10E+00	1.86E-01	2.88E-01	2.77E+00	1.47E+00	2.77E+00	8.29E-02	2.99E-02
XR_003036888.1	9.04E-01	1.88E-01	2.92E-01	5.05E+00	2.34E+00	5.05E+00	7.67E-02	1.52E-02
TBC1D9	1.51E+03	1.90E-01	2.93E-01	1.34E+00	4.21E-01	1.34E+00	9.01E+01	6.73E+01
LOC407145	1.78E+00	1.90E-01	2.93E-01	2.52E+00	1.33E+00	2.52E+00	1.31E-01	5.20E-02
C5	6.99E+00	1.91E-01	2.94E-01	1.34E+00	4.27E-01	1.34E+00	4.19E-01	3.12E-01
ATP6V0A1	1.20E+03	1.93E-01	2.97E-01	1.15E+00	1.99E-01	1.15E+00	6.73E+01	5.86E+01
PTCHD1	2.31E+00	1.95E-01	3.00E-01	8.69E-01	-2.03E-01	-1.15E+00	1.14E-01	1.31E-01
B3GALNT1	3.84E+02	1.96E-01	3.00E-01	1.14E+00	1.88E-01	1.14E+00	2.15E+01	1.88E+01
LOC616002	6.34E+00	1.96E-01	3.00E-01	3.12E+00	1.64E+00	3.12E+00	4.92E-01	1.58E-01
CD164L2	2.09E+01	1.96E-01	3.00E-01	1.41E+00	5.00E-01	1.41E+00	1.28E+00	9.05E-01
C3	2.22E+03	2.00E-01	3.05E-01	1.88E+00	9.09E-01	1.88E+00	1.50E+02	7.98E+01
TRIP12	3.81E+03	2.01E-01	3.05E-01	1.14E+00	1.92E-01	1.14E+00	2.13E+02	1.87E+02
GFI1	5.22E+01	2.01E-01	3.05E-01	1.26E+00	3.29E-01	1.26E+00	3.04E+00	2.42E+00
XR_003033233.1	1.70E+01	2.02E-01	3.05E-01	1.51E+00	5.94E-01	1.51E+00	1.06E+00	7.05E-01
CIDEA	4.47E+01	2.02E-01	3.05E-01	1.21E+00	2.72E-01	1.21E+00	2.56E+00	2.12E+00
CFHR5	2.70E+00	2.02E-01	3.05E-01	1.58E+00	6.56E-01	1.58E+00	1.72E-01	1.09E-01
TBC1D1	1.53E+03	2.03E-01	3.06E-01	1.11E+00	1.46E-01	1.11E+00	8.42E+01	7.61E+01
CENPJ	3.17E+02	2.04E-01	3.06E-01	1.21E+00	2.81E-01	1.21E+00	1.82E+01	1.50E+01
XR_003037903.1	3.93E+00	2.05E-01	3.07E-01	4.11E+00	2.04E+00	4.11E+00	3.23E-01	7.86E-02
ZFP69	4.50E+02	2.05E-01	3.08E-01	1.13E+00	1.74E-01	1.13E+00	2.51E+01	2.22E+01
LOC788414	1.14E+02	2.08E-01	3.11E-01	1.55E+00	6.28E-01	1.55E+00	7.17E+00	4.64E+00
CDH7	2.35E+02	2.08E-01	3.11E-01	2.56E+00	1.36E+00	2.56E+00	1.74E+01	6.78E+00
XR_003029478.1	1.94E+00	2.09E-01	3.12E-01	2.76E+00	1.46E+00	2.76E+00	1.46E-01	5.30E-02
CAPRIN2	2.04E+03	2.10E-01	3.13E-01	1.18E+00	2.38E-01	1.18E+00	1.15E+02	9.79E+01
LOC112449110	1.00E+01	2.12E-01	3.15E-01	2.73E+00	1.45E+00	2.73E+00	7.53E-01	2.76E-01
XR_003029901.1	1.15E+00	2.13E-01	3.17E-01	1.32E+01	3.72E+00	1.32E+01	1.08E-01	8.19E-03
FAM71F2	6.44E+00	2.15E-01	3.18E-01	1.58E+00	6.62E-01	1.58E+00	4.10E-01	2.59E-01
ABCA3	7.20E+02	2.15E-01	3.18E-01	1.31E+00	3.91E-01	1.31E+00	4.27E+01	3.25E+01
GRAP2	1.64E+01	2.19E-01	3.24E-01	1.45E+00	5.40E-01	1.45E+00	1.01E+00	6.98E-01

XR_003035449.1	2.79E+00	2.21E-01	3.25E-01	1.86E+00	8.97E-01	1.86E+00	1.88E-01	1.01E-01
LOC100336903	1.44E+01	2.21E-01	3.26E-01	2.09E+00	1.07E+00	2.09E+00	1.00E+00	4.80E-01
LOC101904580	5.62E+00	2.24E-01	3.29E-01	4.65E+00	2.22E+00	4.65E+00	4.71E-01	1.01E-01
XR_003035607.1	2.81E+01	2.25E-01	3.29E-01	1.25E+00	3.25E-01	1.25E+00	1.63E+00	1.30E+00
KIAA0556	1.17E+03	2.26E-01	3.31E-01	1.18E+00	2.38E-01	1.18E+00	6.61E+01	5.61E+01
PHYHD1	4.92E+02	2.33E-01	3.40E-01	1.11E+00	1.48E-01	1.11E+00	2.72E+01	2.45E+01
XR_816450.3	1.56E+01	2.33E-01	3.40E-01	1.25E+00	3.19E-01	1.25E+00	9.06E-01	7.26E-01
NEIL2	5.80E+02	2.35E-01	3.42E-01	9.08E-01	-1.39E-01	-1.10E+00	2.91E+01	3.21E+01
APH1B	4.64E+02	2.38E-01	3.46E-01	1.37E+00	4.51E-01	1.37E+00	2.80E+01	2.05E+01
LOC100138519	1.59E+00	2.40E-01	3.48E-01	2.80E+00	1.48E+00	2.80E+00	1.20E-01	4.30E-02
XR_807447.3	1.30E+01	2.42E-01	3.50E-01	2.27E+00	1.18E+00	2.27E+00	9.34E-01	4.11E-01
AKAIN1	7.88E-01	2.43E-01	3.52E-01	8.41E+01	6.39E+00	8.41E+01	7.80E-02	9.27E-04
CNOT1	3.08E+03	2.46E-01	3.55E-01	1.17E+00	2.24E-01	1.17E+00	1.74E+02	1.49E+02
NUP133	1.03E+03	2.47E-01	3.55E-01	1.18E+00	2.42E-01	1.18E+00	5.86E+01	4.96E+01
NUBPL	5.27E+01	2.47E-01	3.55E-01	1.17E+00	2.27E-01	1.17E+00	2.98E+00	2.54E+00
EIF4ENIF1	2.02E+03	2.47E-01	3.55E-01	1.20E+00	2.62E-01	1.20E+00	1.16E+02	9.64E+01
CHRNA3-2	3.69E+02	2.47E-01	3.55E-01	1.19E+00	2.54E-01	1.19E+00	2.10E+01	1.76E+01
ZNF181	3.69E+02	2.51E-01	3.59E-01	1.09E+00	1.24E-01	1.09E+00	2.02E+01	1.85E+01
XR_003035965.1	3.73E+00	2.51E-01	3.60E-01	8.86E-01	-1.75E-01	-1.13E+00	1.85E-01	2.09E-01
XR_003031166.1	1.65E+00	2.52E-01	3.60E-01	2.37E+00	1.25E+00	2.37E+00	1.19E-01	5.03E-02
XR_003032403.1	3.11E+00	2.52E-01	3.60E-01	1.58E+00	6.61E-01	1.58E+00	1.98E-01	1.25E-01
UBE2U	4.40E+01	2.53E-01	3.60E-01	1.55E+00	6.33E-01	1.55E+00	2.79E+00	1.80E+00
CFI	4.25E+02	2.54E-01	3.61E-01	1.42E+00	5.05E-01	1.42E+00	2.60E+01	1.83E+01
AGBL2	2.24E+02	2.56E-01	3.63E-01	1.35E+00	4.29E-01	1.35E+00	1.34E+01	9.98E+00
GJB2	7.32E+01	2.57E-01	3.64E-01	1.63E+00	7.08E-01	1.63E+00	4.72E+00	2.89E+00
FAM184A	3.23E+02	2.58E-01	3.65E-01	1.26E+00	3.35E-01	1.26E+00	1.88E+01	1.49E+01
FAM19A1	7.77E+00	2.61E-01	3.69E-01	3.51E+00	1.81E+00	3.51E+00	6.18E-01	1.76E-01
LINGO2	1.15E+02	2.62E-01	3.69E-01	1.41E+00	4.96E-01	1.41E+00	7.02E+00	4.98E+00
C10H15orf61	6.06E+01	2.63E-01	3.70E-01	1.26E+00	3.30E-01	1.26E+00	3.53E+00	2.81E+00
EYA3	1.45E+03	2.65E-01	3.72E-01	1.10E+00	1.36E-01	1.10E+00	7.97E+01	7.25E+01
XR_003030628.1	7.60E+02	2.67E-01	3.75E-01	1.11E+00	1.56E-01	1.11E+00	4.20E+01	3.77E+01
CLEC4G	8.64E+00	2.69E-01	3.77E-01	1.31E+00	3.85E-01	1.31E+00	5.12E-01	3.92E-01
RAI14	1.13E+03	2.70E-01	3.78E-01	1.22E+00	2.91E-01	1.22E+00	6.52E+01	5.33E+01

MAPT	1.31E+02	2.72E-01	3.79E-01	1.32E+00	3.97E-01	1.32E+00	7.76E+00	5.89E+00
XR_003036255.1	2.50E+00	2.76E-01	3.84E-01	7.00E+00	2.81E+00	7.00E+00	2.22E-01	3.17E-02
XR_003034271.1	6.05E+01	2.76E-01	3.84E-01	1.64E+00	7.10E-01	1.64E+00	3.90E+00	2.39E+00
LOC107132775	2.21E+01	2.78E-01	3.86E-01	2.07E+00	1.05E+00	2.07E+00	1.54E+00	7.45E-01
C23H6orf10	5.76E-01	2.79E-01	3.87E-01	1.50E+01	3.91E+00	1.50E+01	5.44E-02	3.63E-03
LOC100296379	1.16E+00	2.81E-01	3.89E-01	6.26E+00	2.65E+00	6.26E+00	1.02E-01	1.63E-02
XR_003031547.1	7.81E+00	2.82E-01	3.90E-01	1.48E+00	5.63E-01	1.48E+00	4.86E-01	3.29E-01
HIST1H1A	3.75E+00	2.83E-01	3.91E-01	1.61E+00	6.90E-01	1.61E+00	2.40E-01	1.49E-01
TAS1R3	2.91E+01	2.85E-01	3.92E-01	1.25E+00	3.21E-01	1.25E+00	1.69E+00	1.35E+00
XR_003029787.1	2.33E+00	2.86E-01	3.94E-01	2.44E+00	1.29E+00	2.44E+00	1.70E-01	6.98E-02
SPATC1L	7.81E+00	2.92E-01	4.02E-01	1.58E+00	6.62E-01	1.58E+00	4.98E-01	3.15E-01
MYRFL	9.32E+00	2.93E-01	4.02E-01	1.71E+00	7.71E-01	1.71E+00	6.10E-01	3.58E-01
CYP3A5-2	4.32E+00	2.95E-01	4.03E-01	2.38E+00	1.25E+00	2.38E+00	3.13E-01	1.32E-01
OPN4	3.65E+00	2.95E-01	4.03E-01	2.45E+00	1.29E+00	2.45E+00	2.67E-01	1.09E-01
SALL3	1.10E+02	2.99E-01	4.09E-01	1.97E+00	9.81E-01	1.97E+00	7.57E+00	3.83E+00
TMEM255B	5.26E+01	3.01E-01	4.10E-01	1.54E+00	6.23E-01	1.54E+00	3.32E+00	2.16E+00
RALGAPA2	5.16E+02	3.03E-01	4.12E-01	1.19E+00	2.48E-01	1.19E+00	2.94E+01	2.47E+01
TPK1	6.48E+02	3.04E-01	4.12E-01	1.17E+00	2.22E-01	1.17E+00	3.66E+01	3.14E+01
NRSN1	1.04E+01	3.04E-01	4.12E-01	1.65E+00	7.24E-01	1.65E+00	6.76E-01	4.09E-01
XR_003032358.1	2.68E+00	3.05E-01	4.12E-01	1.19E+01	3.57E+00	1.19E+01	2.49E-01	2.10E-02
XR_003033250.1	4.63E+00	3.05E-01	4.12E-01	1.49E+00	5.75E-01	1.49E+00	2.89E-01	1.94E-01
PPEF1	2.02E+02	3.07E-01	4.14E-01	1.25E+00	3.25E-01	1.25E+00	1.18E+01	9.38E+00
XR_003035884.1	4.06E-01	3.07E-01	4.14E-01	3.83E+02	8.58E+00	3.83E+02	4.05E-02	1.06E-04
XR_001494446.2	7.70E+00	3.07E-01	4.14E-01	1.47E+00	5.56E-01	1.47E+00	4.78E-01	3.25E-01
XR_814612.3	9.90E+00	3.09E-01	4.15E-01	1.62E+00	6.92E-01	1.62E+00	6.36E-01	3.94E-01
RASGEF1A	1.91E+02	3.09E-01	4.15E-01	1.35E+00	4.35E-01	1.35E+00	1.15E+01	8.49E+00
LOC112446361	3.87E+00	3.12E-01	4.18E-01	1.67E+00	7.44E-01	1.67E+00	2.52E-01	1.50E-01
XR_003037564.1	1.23E+00	3.16E-01	4.23E-01	1.64E+00	7.15E-01	1.64E+00	7.95E-02	4.84E-02
CDK5R1	1.30E+02	3.16E-01	4.23E-01	1.46E+00	5.50E-01	1.46E+00	8.06E+00	5.50E+00
PDE1C	1.61E+02	3.20E-01	4.27E-01	9.78E-01	-3.15E-02	-1.02E+00	8.41E+00	8.59E+00
XR_001500699.2	4.51E+00	3.20E-01	4.27E-01	1.74E+00	8.03E-01	1.74E+00	2.98E-01	1.71E-01
HBQ1	1.30E+02	3.21E-01	4.27E-01	1.10E+00	1.37E-01	1.10E+00	7.17E+00	6.52E+00
XR_003030389.1	6.91E+00	3.22E-01	4.28E-01	2.05E+00	1.03E+00	2.05E+00	4.80E-01	2.35E-01

GCKR	7.27E+00	3.26E-01	4.32E-01	1.38E+00	4.65E-01	1.38E+00	4.40E-01	3.19E-01
CCR9	1.24E+01	3.27E-01	4.33E-01	1.06E+00	8.61E-02	1.06E+00	6.71E-01	6.33E-01
XR_003037141.1	2.56E+00	3.28E-01	4.34E-01	2.24E+00	1.16E+00	2.24E+00	1.82E-01	8.15E-02
HAAO	3.44E+01	3.30E-01	4.35E-01	1.40E+00	4.80E-01	1.40E+00	2.09E+00	1.50E+00
LOC112448731	9.99E+00	3.34E-01	4.41E-01	1.31E+00	3.87E-01	1.31E+00	5.92E-01	4.53E-01
HMGXB3	1.47E+03	3.35E-01	4.41E-01	1.13E+00	1.72E-01	1.13E+00	8.17E+01	7.25E+01
ZFP2	7.41E+02	3.40E-01	4.47E-01	1.09E+00	1.29E-01	1.09E+00	4.06E+01	3.71E+01
TEX29	2.23E+01	3.43E-01	4.50E-01	1.40E+00	4.82E-01	1.40E+00	1.35E+00	9.70E-01
ALOX12	5.52E+01	3.47E-01	4.54E-01	1.33E+00	4.06E-01	1.33E+00	3.29E+00	2.48E+00
TLE4	7.18E+02	3.61E-01	4.72E-01	1.21E+00	2.73E-01	1.21E+00	4.12E+01	3.41E+01
NOX3	9.25E-01	3.61E-01	4.72E-01	2.65E+00	1.41E+00	2.65E+00	6.90E-02	2.60E-02
WDR76	6.08E+02	3.64E-01	4.75E-01	1.19E+00	2.45E-01	1.19E+00	3.45E+01	2.91E+01
SGF29	4.59E+02	3.65E-01	4.76E-01	1.20E+00	2.61E-01	1.20E+00	2.62E+01	2.19E+01
XR_003036159.1	5.47E+00	3.68E-01	4.78E-01	1.10E+00	1.32E-01	1.10E+00	3.00E-01	2.74E-01
CHRNA10	2.23E+01	3.69E-01	4.80E-01	1.43E+00	5.15E-01	1.43E+00	1.37E+00	9.59E-01
WIF1	1.27E+02	3.72E-01	4.83E-01	1.52E+00	6.08E-01	1.52E+00	7.97E+00	5.23E+00
XR_003032642.1	8.55E+00	3.78E-01	4.90E-01	1.52E+00	6.01E-01	1.52E+00	5.37E-01	3.54E-01
XR_003033863.1	4.05E+00	3.81E-01	4.93E-01	2.48E+00	1.31E+00	2.48E+00	2.97E-01	1.20E-01
DYM	1.64E+03	3.83E-01	4.94E-01	1.08E+00	1.17E-01	1.08E+00	8.97E+01	8.27E+01
FBXO10	7.68E+02	3.87E-01	4.99E-01	1.21E+00	2.81E-01	1.21E+00	4.41E+01	3.63E+01
XR_804250.3	2.45E+00	3.95E-01	5.08E-01	1.27E+00	3.50E-01	1.27E+00	1.44E-01	1.13E-01
HID1	8.00E+02	3.96E-01	5.09E-01	1.12E+00	1.61E-01	1.12E+00	4.43E+01	3.96E+01
MYO7A	1.12E+02	3.99E-01	5.11E-01	1.36E+00	4.44E-01	1.36E+00	6.72E+00	4.94E+00
LOC112441554	2.35E+00	4.01E-01	5.14E-01	1.33E+00	4.09E-01	1.33E+00	1.40E-01	1.05E-01
XR_003037900.1	2.07E+00	4.04E-01	5.16E-01	2.95E+00	1.56E+00	2.95E+00	1.58E-01	5.37E-02
LOC112442380	1.58E+01	4.05E-01	5.18E-01	1.35E+00	4.29E-01	1.35E+00	9.49E-01	7.05E-01
OS9	4.28E+03	4.07E-01	5.19E-01	1.04E+00	5.46E-02	1.04E+00	2.29E+02	2.21E+02
LOC781830	5.45E+00	4.12E-01	5.24E-01	1.45E+00	5.36E-01	1.45E+00	3.36E-01	2.32E-01
PWP1	1.41E+03	4.20E-01	5.34E-01	1.10E+00	1.44E-01	1.10E+00	7.79E+01	7.05E+01
C2H2orf66	1.69E+00	4.25E-01	5.39E-01	2.83E+00	1.50E+00	2.83E+00	1.28E-01	4.54E-02
XR_003035656.1	1.99E+01	4.25E-01	5.39E-01	1.41E+00	4.99E-01	1.41E+00	1.21E+00	8.60E-01
UNC5D	7.59E+01	4.26E-01	5.39E-01	1.62E+00	7.00E-01	1.62E+00	4.89E+00	3.01E+00
ESR2	2.60E+02	4.26E-01	5.39E-01	1.34E+00	4.21E-01	1.34E+00	1.55E+01	1.16E+01

XR_813019.3	5.58E+01	4.34E-01	5.48E-01	1.43E+00	5.21E-01	1.43E+00	3.43E+00	2.39E+00
BMP3	6.12E+01	4.35E-01	5.48E-01	1.63E+00	7.08E-01	1.63E+00	3.95E+00	2.42E+00
CEL	6.71E+00	4.36E-01	5.48E-01	1.26E+00	3.31E-01	1.26E+00	3.91E-01	3.11E-01
OR12D2	6.62E+00	4.37E-01	5.48E-01	2.64E+00	1.40E+00	2.64E+00	4.94E-01	1.87E-01
XR_003037869.1	5.06E+01	4.37E-01	5.48E-01	1.23E+00	2.96E-01	1.23E+00	2.92E+00	2.38E+00
SCGB1A1	2.33E+01	4.39E-01	5.50E-01	1.23E+00	2.96E-01	1.23E+00	1.34E+00	1.09E+00
XR_003035657.1	9.02E-01	4.42E-01	5.53E-01	2.67E+01	4.74E+00	2.67E+01	8.72E-02	3.27E-03
LOC112445942	1.20E+01	4.43E-01	5.53E-01	1.23E+00	2.94E-01	1.23E+00	6.91E-01	5.64E-01
PIF1	1.21E+02	4.44E-01	5.53E-01	1.46E+00	5.48E-01	1.46E+00	7.50E+00	5.13E+00
OMA1	1.37E+02	4.44E-01	5.53E-01	1.10E+00	1.43E-01	1.10E+00	7.53E+00	6.82E+00
PRIMPOL	2.50E+02	4.47E-01	5.56E-01	1.17E+00	2.22E-01	1.17E+00	1.41E+01	1.21E+01
XR_003032215.1	9.04E+00	4.47E-01	5.56E-01	2.50E+00	1.32E+00	2.50E+00	6.65E-01	2.66E-01
XR_003035888.1	1.84E+00	4.50E-01	5.59E-01	3.18E+00	1.67E+00	3.18E+00	1.44E-01	4.51E-02
TTC1	1.02E+03	4.53E-01	5.61E-01	9.62E-01	-5.53E-02	-1.04E+00	5.28E+01	5.49E+01
LOC112445014	4.50E+00	4.66E-01	5.76E-01	1.34E+00	4.26E-01	1.34E+00	2.70E-01	2.01E-01
GALK2	2.22E+02	4.69E-01	5.79E-01	1.11E+00	1.55E-01	1.11E+00	1.23E+01	1.10E+01
MLXIPL	1.32E+02	4.70E-01	5.80E-01	1.43E+00	5.19E-01	1.43E+00	8.14E+00	5.68E+00
C19H17orf97	6.84E+01	4.72E-01	5.82E-01	1.43E+00	5.17E-01	1.43E+00	4.20E+00	2.93E+00
DPCD	4.17E+02	4.81E-01	5.92E-01	1.16E+00	2.11E-01	1.16E+00	2.35E+01	2.03E+01
MCOLN1	6.69E+02	4.85E-01	5.96E-01	1.04E+00	5.87E-02	1.04E+00	3.59E+01	3.44E+01
XR_003034384.1	2.39E+00	4.86E-01	5.96E-01	1.60E+00	6.77E-01	1.60E+00	1.53E-01	9.57E-02
ABI2	1.43E+03	4.87E-01	5.97E-01	1.09E+00	1.29E-01	1.09E+00	7.84E+01	7.17E+01
LOC107132761	1.60E+00	4.96E-01	6.06E-01	1.28E+00	3.55E-01	1.28E+00	9.40E-02	7.35E-02
XR_003033548.1	4.05E+00	5.00E-01	6.11E-01	1.72E+00	7.87E-01	1.72E+00	2.66E-01	1.54E-01
AMHR2	6.63E+02	5.01E-01	6.11E-01	1.00E+00	3.30E-03	1.00E+00	3.49E+01	3.49E+01
TBX19	6.81E+01	5.08E-01	6.19E-01	1.12E+00	1.61E-01	1.12E+00	3.77E+00	3.38E+00
STK10	8.19E+02	5.16E-01	6.28E-01	1.06E+00	7.77E-02	1.06E+00	4.42E+01	4.19E+01
XR_003037271.1	9.43E-01	5.21E-01	6.32E-01	4.09E+00	2.03E+00	4.09E+00	7.73E-02	1.89E-02
XR_003030974.1	1.20E+00	5.21E-01	6.33E-01	1.90E+00	9.27E-01	1.90E+00	8.12E-02	4.27E-02
UNCX	1.93E+01	5.24E-01	6.34E-01	1.25E+00	3.23E-01	1.25E+00	1.12E+00	8.96E-01
XR_235065.4	5.29E+01	5.30E-01	6.41E-01	1.08E+00	1.09E-01	1.08E+00	2.88E+00	2.67E+00
PPP1R9A	6.43E+02	5.30E-01	6.41E-01	1.11E+00	1.48E-01	1.11E+00	3.55E+01	3.20E+01
ARHGAP22	4.06E+02	5.34E-01	6.44E-01	1.12E+00	1.64E-01	1.12E+00	2.25E+01	2.01E+01

XR_003032635.1	1.75E+00	5.36E-01	6.46E-01	1.52E+00	6.01E-01	1.52E+00	1.10E-01	7.23E-02
NR_003052.2	1.55E+00	5.37E-01	6.46E-01	1.62E+00	6.96E-01	1.62E+00	9.96E-02	6.15E-02
LOC785156	1.52E+00	5.41E-01	6.50E-01	2.74E+00	1.46E+00	2.74E+00	1.15E-01	4.18E-02
GABRA4	7.30E+00	5.56E-01	6.67E-01	1.50E+00	5.90E-01	1.50E+00	4.57E-01	3.04E-01
LOC783740	2.99E+00	5.57E-01	6.67E-01	1.33E+00	4.13E-01	1.33E+00	1.79E-01	1.34E-01
SCN4A	2.13E+01	5.59E-01	6.69E-01	1.61E+00	6.89E-01	1.61E+00	1.36E+00	8.46E-01
AKAP7	1.52E+02	5.64E-01	6.73E-01	1.07E+00	9.16E-02	1.07E+00	8.26E+00	7.75E+00
POU4F1	7.83E+01	5.64E-01	6.73E-01	1.19E+00	2.47E-01	1.19E+00	4.45E+00	3.75E+00
XR_003035538.1	1.46E+01	5.68E-01	6.77E-01	1.27E+00	3.50E-01	1.27E+00	8.57E-01	6.73E-01
XR_003030284.1	1.18E+01	5.72E-01	6.81E-01	1.22E+00	2.86E-01	1.22E+00	6.80E-01	5.58E-01
XR_003030938.1	1.67E+01	5.76E-01	6.84E-01	1.40E+00	4.86E-01	1.40E+00	1.02E+00	7.26E-01
XR_815375.3	6.01E+00	5.77E-01	6.85E-01	2.29E+00	1.20E+00	2.29E+00	4.32E-01	1.88E-01
PLEKHF1	4.00E+02	5.80E-01	6.87E-01	1.23E+00	2.99E-01	1.23E+00	2.31E+01	1.88E+01
GNG4	2.38E+02	5.81E-01	6.87E-01	1.35E+00	4.36E-01	1.35E+00	1.43E+01	1.06E+01
SLC24A1	1.02E+02	5.84E-01	6.90E-01	1.29E+00	3.72E-01	1.29E+00	6.02E+00	4.65E+00
TMPRSS9	1.57E+02	5.85E-01	6.90E-01	1.20E+00	2.63E-01	1.20E+00	8.99E+00	7.49E+00
SLC10A4	8.52E+01	5.86E-01	6.90E-01	1.08E+00	1.05E-01	1.08E+00	4.64E+00	4.31E+00
GPR37	2.78E+01	5.88E-01	6.92E-01	1.22E+00	2.91E-01	1.22E+00	1.60E+00	1.31E+00
XR_003035907.1	6.76E-01	5.93E-01	6.97E-01	1.67E+00	7.37E-01	1.67E+00	4.39E-02	2.63E-02
TSPAN13	4.55E+02	5.94E-01	6.97E-01	1.09E+00	1.21E-01	1.09E+00	2.49E+01	2.29E+01
PGS1	6.11E+02	5.97E-01	7.00E-01	1.11E+00	1.46E-01	1.11E+00	3.37E+01	3.04E+01
LOC100848912	1.26E+01	6.03E-01	7.04E-01	1.51E+00	5.94E-01	1.51E+00	7.86E-01	5.21E-01
CHD7	7.47E+02	6.03E-01	7.04E-01	1.18E+00	2.35E-01	1.18E+00	4.23E+01	3.60E+01
EZR	4.92E+03	6.04E-01	7.05E-01	1.01E+00	1.82E-02	1.01E+00	2.60E+02	2.57E+02
XR_003032139.1	4.29E+01	6.08E-01	7.09E-01	1.25E+00	3.26E-01	1.25E+00	2.50E+00	1.99E+00
EYA1	1.00E+02	6.14E-01	7.15E-01	1.33E+00	4.11E-01	1.33E+00	5.97E+00	4.49E+00
GSPT2	5.50E+02	6.15E-01	7.15E-01	1.04E+00	5.59E-02	1.04E+00	2.95E+01	2.84E+01
LOC112446429	1.51E+00	6.18E-01	7.17E-01	2.77E+00	1.47E+00	2.77E+00	1.14E-01	4.13E-02
LOC511713	1.47E+02	6.18E-01	7.17E-01	9.71E-01	-4.21E-02	-1.03E+00	7.65E+00	7.88E+00
SLC12A6	8.81E+02	6.20E-01	7.18E-01	1.09E+00	1.27E-01	1.09E+00	4.83E+01	4.42E+01
XR_001501641.2	1.03E+01	6.22E-01	7.19E-01	1.25E+00	3.21E-01	1.25E+00	6.00E-01	4.80E-01
S100Z	7.54E+00	6.31E-01	7.29E-01	1.51E+00	5.98E-01	1.51E+00	4.73E-01	3.12E-01
XR_003031979.1	1.49E+02	6.33E-01	7.31E-01	1.24E+00	3.16E-01	1.24E+00	8.62E+00	6.93E+00

PNOC	9.41E+00	6.34E-01	7.31E-01	1.41E+00	5.00E-01	1.41E+00	5.75E-01	4.06E-01
RPP21	1.79E+02	6.38E-01	7.34E-01	1.11E+00	1.47E-01	1.11E+00	9.86E+00	8.90E+00
LOC112449240	2.76E+01	6.38E-01	7.34E-01	1.08E+00	1.10E-01	1.08E+00	1.51E+00	1.39E+00
GLS2	1.57E+02	6.44E-01	7.39E-01	1.21E+00	2.78E-01	1.21E+00	9.01E+00	7.43E+00
SPERT	8.39E+00	6.45E-01	7.39E-01	3.08E+00	1.63E+00	3.08E+00	6.50E-01	2.11E-01
APC2	2.37E+02	6.47E-01	7.40E-01	1.11E+00	1.57E-01	1.11E+00	1.31E+01	1.18E+01
LOC522128	2.35E+01	6.49E-01	7.42E-01	1.71E+00	7.78E-01	1.71E+00	1.54E+00	8.98E-01
LOC104970608	1.28E+00	6.50E-01	7.42E-01	3.24E+00	1.69E+00	3.24E+00	1.00E-01	3.09E-02
XR_003032511.1	1.57E+01	6.51E-01	7.43E-01	2.14E+00	1.10E+00	2.14E+00	1.11E+00	5.17E-01
XR_003034346.1	6.28E-01	6.53E-01	7.43E-01	1.84E+00	8.81E-01	1.84E+00	4.22E-02	2.29E-02
XR_815227.3	3.80E+01	6.55E-01	7.44E-01	1.21E+00	2.79E-01	1.21E+00	2.18E+00	1.80E+00
DACH1	4.98E+02	6.55E-01	7.44E-01	1.14E+00	1.91E-01	1.14E+00	2.79E+01	2.44E+01
XR_003030253.1	1.03E+01	6.60E-01	7.48E-01	3.22E+00	1.69E+00	3.22E+00	8.09E-01	2.51E-01
PPP1R17	2.49E+00	6.60E-01	7.48E-01	6.75E+00	2.76E+00	6.75E+00	2.20E-01	3.25E-02
FAM192A	1.07E+03	6.66E-01	7.54E-01	9.93E-01	-1.06E-02	-1.01E+00	5.61E+01	5.65E+01
ARHGAP44	4.88E+02	6.69E-01	7.55E-01	1.19E+00	2.51E-01	1.19E+00	2.78E+01	2.34E+01
GRIA3	1.83E+02	6.70E-01	7.56E-01	1.07E+00	9.11E-02	1.07E+00	9.92E+00	9.31E+00
XR_003037218.1	3.07E+00	6.74E-01	7.59E-01	1.79E+00	8.38E-01	1.79E+00	2.04E-01	1.14E-01
KIAA1324L	1.01E+02	6.76E-01	7.60E-01	1.42E+00	5.05E-01	1.42E+00	6.19E+00	4.36E+00
HEPHL1	7.09E+00	6.77E-01	7.60E-01	1.17E+00	2.27E-01	1.17E+00	4.01E-01	3.43E-01
ANKS1B	4.44E+01	6.91E-01	7.75E-01	1.02E+00	2.58E-02	1.02E+00	2.36E+00	2.32E+00
CASZ1	5.09E+02	6.92E-01	7.75E-01	1.09E+00	1.29E-01	1.09E+00	2.79E+01	2.55E+01
MYO16	4.85E+01	6.93E-01	7.76E-01	1.52E+00	6.00E-01	1.52E+00	3.04E+00	2.01E+00
XR_003032619.1	5.67E+00	6.94E-01	7.76E-01	3.76E+00	1.91E+00	3.76E+00	4.57E-01	1.22E-01
CATSPERG	5.84E+02	7.00E-01	7.81E-01	1.07E+00	1.02E-01	1.07E+00	3.17E+01	2.96E+01
XR_003035629.1	6.10E+01	7.02E-01	7.82E-01	1.12E+00	1.62E-01	1.12E+00	3.38E+00	3.02E+00
XR_003031581.1	5.70E+00	7.02E-01	7.82E-01	1.93E+00	9.45E-01	1.93E+00	3.88E-01	2.02E-01
SLC12A5	4.04E+01	7.08E-01	7.88E-01	1.12E+00	1.67E-01	1.12E+00	2.24E+00	2.00E+00
EPPK1	1.03E+03	7.10E-01	7.89E-01	1.11E+00	1.48E-01	1.11E+00	5.70E+01	5.15E+01
LOC107132994	3.69E+00	7.11E-01	7.89E-01	1.58E+00	6.59E-01	1.58E+00	2.35E-01	1.49E-01
ZFAND5	1.52E+03	7.20E-01	7.98E-01	1.02E+00	3.14E-02	1.02E+00	8.08E+01	7.90E+01
TRHDE	5.92E+01	7.26E-01	8.04E-01	1.38E+00	4.64E-01	1.38E+00	3.59E+00	2.60E+00
LOC104969340	4.00E+02	7.29E-01	8.06E-01	1.05E+00	6.39E-02	1.05E+00	2.15E+01	2.05E+01

USP29	3.40E+00	7.38E-01	8.15E-01	1.35E+00	4.35E-01	1.35E+00	2.04E-01	1.51E-01
XR_003035572.1	1.77E+00	7.39E-01	8.15E-01	1.32E+00	4.03E-01	1.32E+00	1.05E-01	7.97E-02
DSTYK	9.97E+02	7.41E-01	8.16E-01	1.07E+00	9.22E-02	1.07E+00	5.41E+01	5.07E+01
DHX58	9.39E+02	7.45E-01	8.19E-01	1.04E+00	6.02E-02	1.04E+00	5.04E+01	4.83E+01
XR_809755.3	4.33E+00	7.47E-01	8.19E-01	1.96E+00	9.67E-01	1.96E+00	2.97E-01	1.52E-01
PSEN1	1.33E+03	7.47E-01	8.19E-01	1.03E+00	3.80E-02	1.03E+00	7.09E+01	6.91E+01
LOC516904	6.54E-01	7.48E-01	8.19E-01	6.52E+00	2.71E+00	6.52E+00	5.75E-02	8.81E-03
XR_003031875.1	2.33E+00	7.49E-01	8.19E-01	1.67E+00	7.40E-01	1.67E+00	1.51E-01	9.07E-02
XR_003032544.1	4.96E+02	7.52E-01	8.22E-01	9.87E-01	-1.87E-02	-1.01E+00	2.59E+01	2.63E+01
CHP1	1.60E+03	7.53E-01	8.22E-01	1.06E+00	8.00E-02	1.06E+00	8.64E+01	8.18E+01
XR_813547.3	4.34E+01	7.60E-01	8.28E-01	1.56E+00	6.44E-01	1.56E+00	2.75E+00	1.76E+00
XR_003031750.1	4.71E-01	7.64E-01	8.31E-01	1.43E+00	5.13E-01	1.43E+00	2.89E-02	2.02E-02
XR_001501375.2	6.23E+00	7.64E-01	8.31E-01	2.89E+00	1.53E+00	2.89E+00	4.75E-01	1.64E-01
LOC518561	2.23E+00	7.69E-01	8.34E-01	1.90E+00	9.28E-01	1.90E+00	1.51E-01	7.96E-02
XR_003033430.1	3.18E+01	7.69E-01	8.34E-01	1.14E+00	1.91E-01	1.14E+00	1.78E+00	1.56E+00
LOC619014	1.30E+01	7.76E-01	8.40E-01	2.72E+00	1.44E+00	2.72E+00	9.78E-01	3.60E-01
SV2C	3.61E+00	7.78E-01	8.41E-01	1.42E+00	5.06E-01	1.42E+00	2.21E-01	1.56E-01
LOC112442305	3.25E+00	7.78E-01	8.41E-01	1.80E+00	8.48E-01	1.80E+00	2.17E-01	1.20E-01
PSPH	8.21E+01	7.85E-01	8.46E-01	9.99E-01	-1.71E-03	-1.00E+00	4.32E+00	4.32E+00
PHEX	2.27E+02	7.85E-01	8.46E-01	1.24E+00	3.05E-01	1.24E+00	1.31E+01	1.06E+01
XR_003029443.1	4.02E+00	7.86E-01	8.46E-01	1.53E+00	6.13E-01	1.53E+00	2.53E-01	1.66E-01
WNT9A	3.22E+02	7.92E-01	8.51E-01	1.04E+00	4.99E-02	1.04E+00	1.72E+01	1.67E+01
XR_003035879.1	1.55E+00	7.96E-01	8.55E-01	1.44E+00	5.29E-01	1.44E+00	9.54E-02	6.61E-02
HSPA4L	2.29E+02	7.98E-01	8.55E-01	1.14E+00	1.92E-01	1.14E+00	1.28E+01	1.12E+01
XR_003037233.1	6.33E-01	7.98E-01	8.55E-01	3.73E+00	1.90E+00	3.73E+00	5.10E-02	1.37E-02
GPR87	1.68E+00	7.99E-01	8.55E-01	1.50E+00	5.87E-01	1.50E+00	1.05E-01	7.01E-02
MAPKAP1	1.05E+03	8.03E-01	8.58E-01	1.03E+00	3.77E-02	1.03E+00	5.61E+01	5.47E+01
XR_003036864.1	2.24E+00	8.04E-01	8.59E-01	1.31E+00	3.91E-01	1.31E+00	1.33E-01	1.01E-01
CHADL	3.25E+01	8.06E-01	8.59E-01	1.24E+00	3.08E-01	1.24E+00	1.88E+00	1.52E+00
ZNF135	2.91E+02	8.07E-01	8.59E-01	1.04E+00	6.32E-02	1.04E+00	1.56E+01	1.50E+01
TRIM50	9.47E+01	8.08E-01	8.60E-01	1.03E+00	4.37E-02	1.03E+00	5.06E+00	4.90E+00
UBASH3B	1.73E+02	8.16E-01	8.67E-01	1.21E+00	2.76E-01	1.21E+00	9.94E+00	8.21E+00
IFNT2	9.96E+00	8.22E-01	8.71E-01	1.19E+00	2.52E-01	1.19E+00	5.67E-01	4.76E-01

CYSTM1	4.79E+02	8.22E-01	8.71E-01	1.06E+00	8.68E-02	1.06E+00	2.59E+01	2.44E+01
NPAS3	9.07E+01	8.25E-01	8.73E-01	1.30E+00	3.81E-01	1.30E+00	5.36E+00	4.12E+00
GRIK1	2.25E+01	8.28E-01	8.75E-01	1.72E+00	7.83E-01	1.72E+00	1.48E+00	8.60E-01
XR_001501583.2	8.09E+00	8.29E-01	8.76E-01	1.60E+00	6.74E-01	1.60E+00	5.18E-01	3.24E-01
PLK5	4.24E+01	8.31E-01	8.76E-01	1.15E+00	2.04E-01	1.15E+00	2.38E+00	2.07E+00
SKIDA1	1.17E+02	8.33E-01	8.77E-01	1.04E+00	6.01E-02	1.04E+00	6.31E+00	6.05E+00
XR_003034017.1	1.50E+01	8.33E-01	8.77E-01	1.07E+00	9.82E-02	1.07E+00	8.13E-01	7.59E-01
XR_003031513.1	2.08E+00	8.40E-01	8.83E-01	1.69E+00	7.57E-01	1.69E+00	1.36E-01	8.03E-02
PTPN3	3.59E+02	8.42E-01	8.84E-01	1.16E+00	2.09E-01	1.16E+00	2.02E+01	1.75E+01
GPR160	7.17E+01	8.50E-01	8.91E-01	1.38E+00	4.68E-01	1.38E+00	4.34E+00	3.14E+00
PAX3	1.65E+01	8.52E-01	8.92E-01	1.18E+00	2.41E-01	1.18E+00	9.37E-01	7.93E-01
P2RY6	7.09E+01	8.61E-01	9.00E-01	1.12E+00	1.69E-01	1.12E+00	3.94E+00	3.50E+00
XR_003029730.1	1.57E+00	8.65E-01	9.03E-01	2.50E+00	1.32E+00	2.50E+00	1.15E-01	4.61E-02
GPER1	1.04E+02	8.76E-01	9.14E-01	1.35E+00	4.37E-01	1.35E+00	6.24E+00	4.61E+00
XR_003038202.1	9.26E+00	8.79E-01	9.15E-01	1.15E+00	2.07E-01	1.15E+00	5.20E-01	4.51E-01
SLC24A4	2.96E+01	8.84E-01	9.20E-01	1.34E+00	4.24E-01	1.34E+00	1.77E+00	1.32E+00
SLC38A8	1.97E+02	8.85E-01	9.20E-01	1.23E+00	2.98E-01	1.23E+00	1.14E+01	9.23E+00
SHANK2	5.51E+02	8.88E-01	9.21E-01	1.05E+00	7.35E-02	1.05E+00	2.97E+01	2.82E+01
LOC112444897	8.49E+01	8.88E-01	9.21E-01	1.17E+00	2.29E-01	1.17E+00	4.80E+00	4.10E+00
CCDC129	9.66E-01	8.94E-01	9.24E-01	1.54E+00	6.24E-01	1.54E+00	6.10E-02	3.96E-02
ANKRD6	3.63E+02	8.94E-01	9.24E-01	1.10E+00	1.32E-01	1.10E+00	1.99E+01	1.82E+01
XR_003037275.1	2.90E+00	8.94E-01	9.24E-01	1.36E+00	4.46E-01	1.36E+00	1.74E-01	1.28E-01
SLC22A20P	2.21E+00	8.95E-01	9.24E-01	1.72E+00	7.80E-01	1.72E+00	1.45E-01	8.46E-02
OLAH	3.36E+00	8.97E-01	9.24E-01	2.00E+00	1.00E+00	2.00E+00	2.32E-01	1.16E-01
GZMM	1.28E+01	9.06E-01	9.33E-01	1.09E+00	1.23E-01	1.09E+00	7.01E-01	6.44E-01
XR_234571.4	4.42E+02	9.16E-01	9.41E-01	1.05E+00	6.55E-02	1.05E+00	2.37E+01	2.27E+01
RIMBP2	6.09E+01	9.16E-01	9.41E-01	1.06E+00	8.73E-02	1.06E+00	3.30E+00	3.10E+00
KIAA1147	4.67E+02	9.32E-01	9.56E-01	1.02E+00	2.16E-02	1.02E+00	2.47E+01	2.44E+01
EPS8L3	3.89E+00	9.39E-01	9.62E-01	1.37E+00	4.58E-01	1.37E+00	2.35E-01	1.71E-01
XR_001494431.2	2.35E+00	9.40E-01	9.62E-01	1.68E+00	7.46E-01	1.68E+00	1.53E-01	9.11E-02
LOC100336843	2.86E+00	9.44E-01	9.65E-01	1.44E+00	5.27E-01	1.44E+00	1.76E-01	1.22E-01
DTX2	1.03E+03	9.47E-01	9.67E-01	1.09E+00	1.30E-01	1.09E+00	5.65E+01	5.16E+01
XR_003033412.1	1.12E+00	9.49E-01	9.67E-01	1.72E+00	7.85E-01	1.72E+00	7.36E-02	4.27E-02

LOC112442972	1.77E+01	9.50E-01	9.67E-01	1.57E+00	6.49E-01	1.57E+00	1.12E+00	7.15E-01
PTPRZ1	2.18E+02	9.51E-01	9.68E-01	1.49E+00	5.71E-01	1.49E+00	1.36E+01	9.12E+00
XR_003037094.1	1.02E+00	9.59E-01	9.74E-01	1.60E+00	6.78E-01	1.60E+00	6.50E-02	4.06E-02
XR_814259.3	4.76E+00	9.60E-01	9.74E-01	1.21E+00	2.74E-01	1.21E+00	2.73E-01	2.25E-01
LOC104973965	1.31E+01	9.62E-01	9.76E-01	1.20E+00	2.59E-01	1.20E+00	7.46E-01	6.23E-01
XR_139422.5	3.40E+00	9.67E-01	9.78E-01	2.12E+00	1.08E+00	2.12E+00	2.39E-01	1.13E-01
TPCN1	9.54E+02	9.67E-01	9.78E-01	1.04E+00	5.69E-02	1.04E+00	5.12E+01	4.92E+01
XR_003037344.1	1.53E+00	9.68E-01	9.78E-01	1.15E+00	2.08E-01	1.15E+00	8.57E-02	7.42E-02
KLB	2.43E+01	9.69E-01	9.78E-01	1.06E+00	7.93E-02	1.06E+00	1.31E+00	1.24E+00
XR_001501795.2	2.47E+01	9.75E-01	9.83E-01	1.64E+00	7.16E-01	1.64E+00	1.60E+00	9.73E-01
ME1	6.34E+02	9.76E-01	9.83E-01	1.04E+00	6.07E-02	1.04E+00	3.40E+01	3.26E+01
NLRP13	1.42E+02	9.77E-01	9.83E-01	1.28E+00	3.54E-01	1.28E+00	8.34E+00	6.53E+00
UBE2Q1	2.89E+03	9.78E-01	9.83E-01	1.07E+00	9.86E-02	1.07E+00	1.57E+02	1.46E+02
DHODH	2.68E+02	9.79E-01	9.83E-01	1.09E+00	1.20E-01	1.09E+00	1.46E+01	1.35E+01
CLSTN2	2.87E+02	9.86E-01	9.88E-01	1.23E+00	3.03E-01	1.23E+00	1.66E+01	1.34E+01
EXD1	4.46E+01	9.93E-01	9.94E-01	1.08E+00	1.14E-01	1.08E+00	2.43E+00	2.25E+00
ZNF235	5.87E+02	1.00E+00	1.00E+00	1.01E+00	1.60E-02	1.01E+00	3.11E+01	3.07E+01

S5 Table. Gene Specific Analysis (late versus early gestation) for genes in cluster 4. Shown are the fold change and its significance (p-value), log 2 ratio and False Discovery Rate (FDR) across gestation; least square (LS) mean (late and early gestation); total counts per million.

Gene symbol	Total counts	P-value	FDR step up	Ratio	Log2(Ratio)	Fold change	LSMean(Late)	LSMean(Early)
RIPOR2	1.29E+02	9.15E-12	4.72E-09	4.90E+00	2.29E+00	4.90E+00	1.08E+01	2.21E+00
ZFP57	8.72E+02	9.47E-11	2.29E-08	2.48E+00	1.31E+00	2.48E+00	6.33E+01	2.56E+01
GNRHR	1.70E+00	1.33E-10	2.29E-08	1.70E+03	1.07E+01	1.70E+03	1.70E-01	1.00E-04
HOPX	3.24E+02	2.13E-10	2.74E-08	8.41E+00	3.07E+00	8.41E+00	2.92E+01	3.48E+00
ACCSL	1.21E+02	8.58E-10	8.85E-08	1.40E+01	3.81E+00	1.40E+01	1.14E+01	8.11E-01
HEG1	1.20E+03	1.22E-09	1.05E-07	7.07E+00	2.82E+00	7.07E+00	1.06E+02	1.51E+01
NLRP8	5.22E+01	2.77E-09	2.04E-07	2.46E+01	4.62E+00	2.46E+01	5.02E+00	2.04E-01
NPR3	2.37E+02	4.56E-09	2.94E-07	8.46E+00	3.08E+00	8.46E+00	2.13E+01	2.52E+00
KRT2	1.85E+02	5.41E-09	3.10E-07	1.09E+03	1.01E+01	1.09E+03	1.85E+01	1.69E-02
MPP6	6.08E+02	6.29E-09	3.25E-07	2.01E+00	1.01E+00	2.01E+00	4.14E+01	2.06E+01
ARSB	1.08E+03	9.94E-09	4.66E-07	1.83E+00	8.70E-01	1.83E+00	7.20E+01	3.94E+01
TRAM2	1.75E+03	1.32E-08	5.66E-07	1.94E+00	9.58E-01	1.94E+00	1.19E+02	6.12E+01
ACTG2	1.89E+03	2.06E-08	8.19E-07	1.03E+01	3.36E+00	1.03E+01	1.73E+02	1.68E+01
NEDD9	1.18E+03	2.38E-08	8.78E-07	2.20E+00	1.14E+00	2.20E+00	8.30E+01	3.78E+01
EIF4B	1.55E+04	4.70E-08	1.60E-06	1.68E+00	7.45E-01	1.68E+00	1.00E+03	5.98E+02
OLFML2A	3.24E+03	5.18E-08	1.60E-06	1.91E+00	9.37E-01	1.91E+00	2.18E+02	1.14E+02
SERP1	4.76E+02	5.27E-08	1.60E-06	1.65E+00	7.25E-01	1.65E+00	3.05E+01	1.85E+01
MOCOS	4.65E+02	6.56E-08	1.88E-06	5.43E+00	2.44E+00	5.43E+00	3.95E+01	7.27E+00
LRCH1	6.17E+02	7.76E-08	2.11E-06	1.66E+00	7.32E-01	1.66E+00	3.99E+01	2.40E+01
RGMB	1.55E+03	9.50E-08	2.45E-06	1.92E+00	9.41E-01	1.92E+00	1.05E+02	5.44E+01
TAL1	2.00E+02	1.03E-07	2.52E-06	1.71E+00	7.75E-01	1.71E+00	1.30E+01	7.58E+00
ERRFI1	3.72E+03	1.12E-07	2.52E-06	3.82E+00	1.94E+00	3.82E+00	2.99E+02	7.83E+01
ATP13A3	2.82E+02	1.13E-07	2.52E-06	2.34E+00	1.23E+00	2.34E+00	2.01E+01	8.61E+00
APOD	1.16E+03	1.30E-07	2.73E-06	2.81E+01	4.81E+00	2.81E+01	1.12E+02	4.01E+00
LOC101909718	2.17E+01	1.34E-07	2.73E-06	8.99E+01	6.49E+00	8.99E+01	2.15E+00	2.39E-02
P4HA1	1.07E+03	1.38E-07	2.73E-06	1.67E+00	7.42E-01	1.67E+00	6.90E+01	4.13E+01
ANKRD33B	1.00E+02	1.45E-07	2.74E-06	4.46E+00	2.16E+00	4.46E+00	8.36E+00	1.88E+00
LOC100336869	6.24E+00	1.49E-07	2.74E-06	1.41E+02	7.14E+00	1.41E+02	6.19E-01	4.40E-03
REEP5	1.05E+03	1.58E-07	2.80E-06	2.49E+00	1.32E+00	2.49E+00	7.63E+01	3.06E+01

CHST11	5.47E+02	1.80E-07	3.03E-06	5.90E+00	2.56E+00	5.90E+00	4.74E+01	8.04E+00
TLL2	8.70E+02	1.82E-07	3.03E-06	7.46E+00	2.90E+00	7.46E+00	7.77E+01	1.04E+01
TNNI3	6.17E+02	2.99E-07	4.82E-06	6.50E+00	2.70E+00	6.50E+00	5.41E+01	8.32E+00
CDK5R2	8.08E+01	3.65E-07	5.71E-06	5.94E+00	2.57E+00	5.94E+00	6.99E+00	1.18E+00
RAI2	1.91E+02	3.94E-07	5.98E-06	4.02E+00	2.01E+00	4.02E+00	1.55E+01	3.87E+00
CPED1	1.84E+02	4.47E-07	6.58E-06	1.51E+01	3.92E+00	1.51E+01	1.73E+01	1.15E+00
XR_001502153.1	8.77E+01	4.67E-07	6.69E-06	5.64E+00	2.50E+00	5.64E+00	7.53E+00	1.33E+00
TPM2	2.65E+03	4.87E-07	6.80E-06	4.10E+00	2.04E+00	4.10E+00	2.16E+02	5.27E+01
LOC782043	2.16E+00	5.03E-07	6.82E-06	4.36E+01	5.45E+00	4.36E+01	2.11E-01	4.83E-03
LOC786303	8.15E+00	5.40E-07	7.15E-06	2.90E+00	1.53E+00	2.90E+00	6.12E-01	2.11E-01
LATS2	5.87E+02	6.88E-07	8.87E-06	1.50E+00	5.83E-01	1.50E+00	3.65E+01	2.44E+01
GCLC	1.15E+03	7.99E-07	1.00E-05	6.15E+00	2.62E+00	6.15E+00	1.00E+02	1.63E+01
LOC508153	1.54E+02	1.05E-06	1.29E-05	2.81E+00	1.49E+00	2.81E+00	1.15E+01	4.10E+00
XR_808585.3	2.75E+00	1.32E-06	1.57E-05	1.10E+01	3.46E+00	1.10E+01	2.52E-01	2.29E-02
LVRN	2.21E+02	1.34E-06	1.57E-05	4.69E+00	2.23E+00	4.69E+00	1.85E+01	3.94E+00
MGAT4C	2.58E+01	1.50E-06	1.72E-05	1.30E+01	3.70E+00	1.30E+01	2.41E+00	1.86E-01
LRRC2	1.41E+02	1.65E-06	1.85E-05	7.30E+00	2.87E+00	7.30E+00	1.25E+01	1.71E+00
GMPR	1.47E+02	1.97E-06	2.17E-05	2.59E+00	1.37E+00	2.59E+00	1.08E+01	4.18E+00
TNFAIP3	2.01E+02	2.34E-06	2.48E-05	1.89E+00	9.18E-01	1.89E+00	1.35E+01	7.14E+00
FGF14	2.89E+01	2.36E-06	2.48E-05	8.16E+00	3.03E+00	8.16E+00	2.60E+00	3.19E-01
PABPC1	2.08E+04	2.63E-06	2.72E-05	1.38E+00	4.63E-01	1.38E+00	1.25E+03	9.07E+02
TAGAP	1.76E+02	2.95E-06	2.99E-05	1.81E+00	8.57E-01	1.81E+00	1.16E+01	6.40E+00
XR_003035865.1	4.35E+02	3.08E-06	3.06E-05	3.55E+00	1.83E+00	3.55E+00	3.45E+01	9.73E+00
ZBTB16	7.29E+02	3.28E-06	3.19E-05	2.74E+00	1.46E+00	2.74E+00	5.43E+01	1.98E+01
TNIP3	2.25E+01	3.34E-06	3.20E-05	6.06E+00	2.60E+00	6.06E+00	1.97E+00	3.24E-01
LOC112449619	1.11E+01	3.56E-06	3.34E-05	3.30E+00	1.72E+00	3.30E+00	8.70E-01	2.64E-01
PLEK	7.49E+01	3.87E-06	3.56E-05	5.17E+00	2.37E+00	5.17E+00	6.33E+00	1.22E+00
KCNK6	9.35E+00	4.11E-06	3.72E-05	3.49E+00	1.80E+00	3.49E+00	7.34E-01	2.11E-01
P4HA3	1.17E+02	4.61E-06	4.10E-05	3.04E+00	1.61E+00	3.04E+00	9.01E+00	2.96E+00
XR_003035984.1	2.44E+01	4.93E-06	4.31E-05	2.50E+00	1.32E+00	2.50E+00	1.78E+00	7.13E-01
KLF15	1.84E+02	5.02E-06	4.32E-05	2.22E+00	1.15E+00	2.22E+00	1.31E+01	5.90E+00
HHIPL1	5.61E+02	5.24E-06	4.42E-05	2.54E+00	1.35E+00	2.54E+00	4.12E+01	1.62E+01
SMAD4	2.18E+03	5.31E-06	4.42E-05	1.36E+00	4.39E-01	1.36E+00	1.31E+02	9.64E+01

(R_001500496.1	2.23E+00	5.68E-06	4.65E-05	5.93E+00	2.57E+00	5.93E+00	1.97E-01	3.32E-02
ARHGAP10	1.56E+03	6.63E-06	5.35E-05	3.23E+00	1.69E+00	3.23E+00	1.21E+02	3.76E+01
MYL9	3.61E+03	7.38E-06	5.81E-05	2.78E+00	1.47E+00	2.78E+00	2.72E+02	9.79E+01
TTLL7	2.71E+01	7.43E-06	5.81E-05	3.55E+00	1.83E+00	3.55E+00	2.15E+00	6.06E-01
MMD	2.03E+03	7.73E-06	5.96E-05	1.53E+00	6.16E-01	1.53E+00	1.28E+02	8.38E+01
OMD	8.38E+01	7.93E-06	6.02E-05	1.49E+01	3.90E+00	1.49E+01	7.89E+00	5.30E-01
TPM1	9.66E+03	9.97E-06	7.46E-05	2.29E+00	1.19E+00	2.29E+00	6.95E+02	3.04E+02
LOC100335205	2.43E+01	1.03E-05	7.57E-05	7.02E+00	2.81E+00	7.02E+00	2.13E+00	3.04E-01
VNN1	1.31E+02	1.07E-05	7.78E-05	3.61E+01	5.18E+00	3.61E+01	1.28E+01	3.54E-01
CLCA2	7.45E+00	1.10E-05	7.92E-05	1.19E+01	3.57E+00	1.19E+01	6.88E-01	5.78E-02
RGS11	1.47E+02	1.17E-05	8.11E-05	2.68E+00	1.42E+00	2.68E+00	1.09E+01	4.07E+00
(R_003030154.	7.05E+00	1.17E-05	8.11E-05	5.10E+00	2.35E+00	5.10E+00	6.02E-01	1.18E-01
GOLGA5	5.74E+02	1.18E-05	8.11E-05	1.30E+00	3.76E-01	1.30E+00	3.37E+01	2.60E+01
ABCC1	1.29E+03	1.22E-05	8.28E-05	1.38E+00	4.68E-01	1.38E+00	7.78E+01	5.62E+01
EIF4E1B	1.35E+01	1.26E-05	8.45E-05	3.40E+01	5.09E+00	3.40E+01	1.31E+00	3.86E-02
HEYL	1.66E+03	1.86E-05	1.23E-04	1.70E+00	7.68E-01	1.70E+00	1.07E+02	6.31E+01
CITED4	2.39E+02	1.94E-05	1.27E-04	2.66E+00	1.41E+00	2.66E+00	1.78E+01	6.70E+00
(R_003035533.	2.32E+01	2.24E-05	1.45E-04	8.42E+01	6.40E+00	8.42E+01	2.29E+00	2.73E-02
CD244	8.94E+00	2.43E-05	1.55E-04	8.16E+00	3.03E+00	8.16E+00	8.00E-01	9.80E-02
ADAMDEC1	6.19E+01	2.65E-05	1.67E-04	1.48E+01	3.89E+00	1.48E+01	5.83E+00	3.94E-01
GALNT18	3.38E+02	2.90E-05	1.80E-04	1.98E+00	9.85E-01	1.98E+00	2.31E+01	1.17E+01
FSHR	1.17E+02	2.93E-05	1.80E-04	5.30E+00	2.41E+00	5.30E+00	1.00E+01	1.89E+00
TXK	1.91E+01	3.10E-05	1.88E-04	3.72E+00	1.90E+00	3.72E+00	1.54E+00	4.13E-01
HSPB7	1.31E+02	3.40E-05	2.03E-04	1.07E+01	3.42E+00	1.07E+01	1.21E+01	1.13E+00
FAT3	1.63E+02	3.42E-05	2.03E-04	4.69E+00	2.23E+00	4.69E+00	1.37E+01	2.92E+00
UPK1B	3.27E+02	3.60E-05	2.11E-04	1.98E+01	4.31E+00	1.98E+01	3.12E+01	1.58E+00
SPSB1	2.91E+03	3.64E-05	2.11E-04	1.43E+00	5.14E-01	1.43E+00	1.78E+02	1.24E+02
FOXS1	3.18E+02	3.76E-05	2.16E-04	2.52E+00	1.33E+00	2.52E+00	2.32E+01	9.20E+00
MYC	2.18E+03	3.90E-05	2.21E-04	2.58E+00	1.37E+00	2.58E+00	1.61E+02	6.26E+01
CHMP2B	4.19E+02	4.08E-05	2.29E-04	1.35E+00	4.35E-01	1.35E+00	2.51E+01	1.86E+01
EDN3	5.53E+02	4.22E-05	2.34E-04	1.62E+00	6.99E-01	1.62E+00	3.53E+01	2.17E+01
ARSK	1.30E+02	4.30E-05	2.36E-04	1.90E+00	9.24E-01	1.90E+00	8.76E+00	4.62E+00
(R_003035529.	2.67E+00	4.39E-05	2.39E-04	3.32E+01	5.05E+00	3.32E+01	2.60E-01	7.81E-03

(R_001500346.:	2.66E+01	4.53E-05	2.44E-04	2.20E+00	1.14E+00	2.20E+00	1.88E+00	8.55E-01
XR_235225.4	1.04E+01	4.95E-05	2.64E-04	6.16E+00	2.62E+00	6.16E+00	8.97E-01	1.46E-01
ST8SIA5	3.57E+01	5.19E-05	2.73E-04	4.06E+00	2.02E+00	4.06E+00	2.92E+00	7.18E-01
FHL2	9.92E+02	5.33E-05	2.78E-04	2.62E+00	1.39E+00	2.62E+00	7.37E+01	2.82E+01
CGREF1	7.57E+01	5.52E-05	2.85E-04	2.77E+00	1.47E+00	2.77E+00	5.71E+00	2.06E+00
LOC614781	3.03E+00	7.01E-05	3.58E-04	3.74E+01	5.23E+00	3.74E+01	2.95E-01	7.87E-03
LIPG	1.15E+02	9.56E-05	4.84E-04	2.97E+01	4.89E+00	2.97E+01	1.12E+01	3.76E-01
PCNP	1.69E+03	1.12E-04	5.62E-04	1.27E+00	3.42E-01	1.27E+00	9.88E+01	7.80E+01
d-LOC10190801	5.48E+01	1.15E-04	5.69E-04	6.62E+00	2.73E+00	6.62E+00	4.81E+00	7.26E-01
FAM20A	6.50E+01	1.24E-04	6.09E-04	6.61E+00	2.73E+00	6.61E+00	5.70E+00	8.62E-01
GPR174	1.85E+01	1.25E-04	6.09E-04	1.64E+01	4.03E+00	1.64E+01	1.74E+00	1.07E-01
CD8A	1.52E+01	1.30E-04	6.28E-04	1.04E+01	3.37E+00	1.04E+01	1.39E+00	1.34E-01
STX7	6.68E+02	1.34E-04	6.40E-04	1.26E+00	3.34E-01	1.26E+00	3.89E+01	3.09E+01
(R_003033125.:	4.86E+00	1.35E-04	6.40E-04	3.67E+00	1.88E+00	3.67E+00	3.82E-01	1.04E-01
ADAM23	7.82E+01	1.39E-04	6.47E-04	2.71E+00	1.44E+00	2.71E+00	5.80E+00	2.14E+00
ITGBL1	2.08E+02	1.39E-04	6.47E-04	3.83E+01	5.26E+00	3.83E+01	2.04E+01	5.33E-01
LPIN1	4.66E+02	1.50E-04	6.85E-04	1.69E+00	7.57E-01	1.69E+00	3.04E+01	1.80E+01
(R_003036309.:	1.72E+00	1.51E-04	6.85E-04	1.62E+01	4.02E+00	1.62E+01	1.63E-01	1.01E-02
IHH	1.17E+02	1.51E-04	6.85E-04	5.24E+01	5.71E+00	5.24E+01	1.15E+01	2.19E-01
(R_003036828.:	3.94E+01	1.57E-04	7.05E-04	4.13E+00	2.04E+00	4.13E+00	3.25E+00	7.87E-01
PTGES	3.99E+01	1.64E-04	7.28E-04	7.22E+00	2.85E+00	7.22E+00	3.56E+00	4.93E-01
KCNS3	1.14E+02	1.75E-04	7.72E-04	2.49E+00	1.32E+00	2.49E+00	8.34E+00	3.34E+00
(R_001500638.:	7.68E+01	2.16E-04	9.46E-04	3.08E+00	1.62E+00	3.08E+00	5.90E+00	1.92E+00
FAM46B	8.01E+01	2.20E-04	9.55E-04	2.06E+00	1.04E+00	2.06E+00	5.49E+00	2.66E+00
TNFSF14	1.09E+01	2.24E-04	9.62E-04	2.92E+00	1.55E+00	2.92E+00	8.18E-01	2.80E-01
SCD	2.50E+03	2.32E-04	9.88E-04	3.56E+00	1.83E+00	3.56E+00	1.99E+02	5.60E+01
CD69	3.37E+00	2.35E-04	9.96E-04	2.80E+00	1.49E+00	2.80E+00	2.49E-01	8.89E-02
RND3	4.78E+02	2.48E-04	1.04E-03	1.56E+00	6.42E-01	1.56E+00	3.04E+01	1.95E+01
TM6SF1	2.13E+02	2.54E-04	1.06E-03	1.44E+00	5.29E-01	1.44E+00	1.31E+01	9.06E+00
id-TRGC6	4.96E+00	2.61E-04	1.08E-03	1.70E+01	4.09E+00	1.70E+01	4.73E-01	2.78E-02
ZNF683	4.15E+01	2.84E-04	1.16E-03	6.37E+00	2.67E+00	6.37E+00	3.59E+00	5.64E-01
ARFGAP3	1.53E+03	3.02E-04	1.23E-03	2.39E+00	1.25E+00	2.39E+00	1.11E+02	4.65E+01
TNNT1	4.44E+01	3.12E-04	1.26E-03	2.44E+00	1.29E+00	2.44E+00	3.24E+00	1.33E+00

CD7	1.01E+01	3.20E-04	1.28E-03	6.95E+00	2.80E+00	6.95E+00	8.84E-01	1.27E-01
NR_003096.1	3.28E+01	3.29E-04	1.30E-03	7.40E+00	2.89E+00	7.40E+00	2.90E+00	3.92E-01
ERP44	8.97E+02	3.34E-04	1.32E-03	1.22E+00	2.88E-01	1.22E+00	5.16E+01	4.23E+01
PTCH1	1.11E+03	3.37E-04	1.32E-03	2.75E+00	1.46E+00	2.75E+00	8.36E+01	3.03E+01
NR_038087.1	7.03E+01	3.69E-04	1.43E-03	1.79E+00	8.39E-01	1.79E+00	4.68E+00	2.62E+00
COLQ	1.63E+02	3.70E-04	1.43E-03	1.74E+00	7.96E-01	1.74E+00	1.08E+01	6.23E+00
NR5A2	3.05E+02	3.85E-04	1.47E-03	5.78E+00	2.53E+00	5.78E+00	2.64E+01	4.56E+00
(R_001502098.:	8.78E+01	4.07E-04	1.54E-03	1.81E+00	8.57E-01	1.81E+00	5.81E+00	3.21E+00
NOS2	4.10E+02	4.43E-04	1.66E-03	2.24E+00	1.16E+00	2.24E+00	2.93E+01	1.31E+01
HIF1A	1.47E+03	4.45E-04	1.66E-03	1.97E+00	9.75E-01	1.97E+00	1.01E+02	5.12E+01
(R_003034833.	9.61E+00	4.57E-04	1.70E-03	2.33E+01	4.54E+00	2.33E+01	9.24E-01	3.96E-02
GPR88	1.87E+01	4.73E-04	1.74E-03	3.92E+00	1.97E+00	3.92E+00	1.53E+00	3.90E-01
KCNE5	4.05E+02	4.78E-04	1.74E-03	2.29E+00	1.20E+00	2.29E+00	2.90E+01	1.27E+01
OR10AD1	1.34E+00	4.80E-04	1.74E-03	4.98E+00	2.32E+00	4.98E+00	1.12E-01	2.26E-02
PAPSS2	5.55E+02	4.95E-04	1.79E-03	2.96E+00	1.57E+00	2.96E+00	4.27E+01	1.44E+01
HACD4	1.21E+02	5.47E-04	1.96E-03	2.49E+00	1.32E+00	2.49E+00	8.80E+00	3.54E+00
G0S2	2.96E+02	5.54E-04	1.97E-03	1.60E+00	6.78E-01	1.60E+00	1.90E+01	1.19E+01
HMGCS1	1.74E+03	5.59E-04	1.97E-03	2.93E+00	1.55E+00	2.93E+00	1.33E+02	4.55E+01
SLC16A14	2.02E+02	5.61E-04	1.97E-03	1.67E+00	7.42E-01	1.67E+00	1.32E+01	7.90E+00
LOC101907857	3.26E+02	5.99E-04	2.09E-03	6.10E+00	2.61E+00	6.10E+00	2.81E+01	4.61E+00
FAM167B	3.45E+01	6.07E-04	2.09E-03	2.08E+00	1.06E+00	2.08E+00	2.39E+00	1.15E+00
LOC112442215	6.61E+00	6.09E-04	2.09E-03	3.26E+00	1.71E+00	3.26E+00	5.08E-01	1.56E-01
KLRD1	9.38E+00	6.60E-04	2.25E-03	1.86E+01	4.22E+00	1.86E+01	8.93E-01	4.80E-02
XR_233092.4	3.89E+00	6.72E-04	2.28E-03	1.75E+01	4.13E+00	1.75E+01	3.71E-01	2.12E-02
FAM163A	6.01E+02	7.17E-04	2.42E-03	2.07E+00	1.05E+00	2.07E+00	4.15E+01	2.01E+01
XR_815109.3	7.51E+01	7.36E-04	2.46E-03	3.54E+00	1.83E+00	3.54E+00	5.90E+00	1.67E+00
CDH3	1.66E+03	8.67E-04	2.89E-03	1.44E+00	5.31E-01	1.44E+00	1.02E+02	7.04E+01
SEZ6L	3.86E+01	8.80E-04	2.91E-03	3.66E+00	1.87E+00	3.66E+00	3.08E+00	8.42E-01
(R_003032574.	7.00E+01	9.10E-04	2.97E-03	1.88E+00	9.13E-01	1.88E+00	4.72E+00	2.50E+00
CTPS1	1.16E+03	9.11E-04	2.97E-03	1.57E+00	6.47E-01	1.57E+00	7.40E+01	4.72E+01
LOC100848575	6.52E+00	9.16E-04	2.97E-03	1.30E+01	3.70E+00	1.30E+01	6.07E-01	4.67E-02
NAT1	1.58E+02	9.25E-04	2.98E-03	1.44E+00	5.28E-01	1.44E+00	9.72E+00	6.74E+00
LOC104972370	3.62E+00	9.78E-04	3.13E-03	8.05E+00	3.01E+00	8.05E+00	3.22E-01	4.00E-02

RPE	1.93E+02	9.89E-04	3.15E-03	1.30E+00	3.77E-01	1.30E+00	1.14E+01	8.78E+00
DZANK1	1.16E+03	1.02E-03	3.24E-03	1.41E+00	4.93E-01	1.41E+00	7.04E+01	5.00E+01
VLDLR	2.41E+02	1.04E-03	3.28E-03	1.50E+00	5.84E-01	1.50E+00	1.49E+01	9.95E+00
APOLD1	1.40E+02	1.07E-03	3.31E-03	1.82E+00	8.62E-01	1.82E+00	9.46E+00	5.20E+00
(R_003030385.	2.95E+00	1.07E-03	3.31E-03	4.82E+00	2.27E+00	4.82E+00	2.46E-01	5.10E-02
TMEM200A	4.70E+02	1.07E-03	3.31E-03	1.79E+00	8.44E-01	1.79E+00	3.14E+01	1.75E+01
LRRC8D	7.55E+02	1.13E-03	3.46E-03	1.54E+00	6.20E-01	1.54E+00	4.78E+01	3.11E+01
(R_001494808.	2.55E+01	1.15E-03	3.50E-03	3.18E+00	1.67E+00	3.18E+00	1.98E+00	6.22E-01
ST3GAL5	3.08E+02	1.17E-03	3.56E-03	1.63E+00	7.06E-01	1.63E+00	1.97E+01	1.21E+01
FAM83A	2.80E+01	1.20E-03	3.63E-03	3.11E+00	1.64E+00	3.11E+00	2.17E+00	6.98E-01
JSRP1	4.88E+01	1.24E-03	3.73E-03	9.55E+00	3.26E+00	9.55E+00	4.46E+00	4.66E-01
WIPF3	9.26E+02	1.34E-03	3.99E-03	1.35E+00	4.38E-01	1.35E+00	5.54E+01	4.09E+01
LOC100848188	2.10E+01	1.41E-03	4.19E-03	2.22E+00	1.15E+00	2.22E+00	1.49E+00	6.70E-01
LOC513767	1.23E+02	1.42E-03	4.19E-03	1.39E+00	4.72E-01	1.39E+00	7.47E+00	5.39E+00
JOSD2	2.44E+02	1.47E-03	4.32E-03	1.52E+00	6.08E-01	1.52E+00	1.53E+01	1.00E+01
DRD5	1.52E+01	1.51E-03	4.42E-03	6.47E+00	2.69E+00	6.47E+00	1.32E+00	2.04E-01
IQGAP2	8.19E+02	1.63E-03	4.71E-03	1.92E+00	9.40E-01	1.92E+00	5.57E+01	2.90E+01
LAIR1	1.92E+02	1.65E-03	4.76E-03	2.20E+00	1.13E+00	2.20E+00	1.34E+01	6.10E+00
HSD17B1	3.94E+02	1.67E-03	4.79E-03	3.34E+00	1.74E+00	3.34E+00	3.10E+01	9.29E+00
RARRES1	6.44E+02	1.73E-03	4.94E-03	2.20E+00	1.14E+00	2.20E+00	4.58E+01	2.08E+01
RANGRF	2.12E+02	1.75E-03	4.96E-03	1.29E+00	3.69E-01	1.29E+00	1.25E+01	9.64E+00
LOC511617	1.55E+01	1.82E-03	5.12E-03	4.04E+00	2.01E+00	4.04E+00	1.24E+00	3.08E-01
(R_003035035.	4.55E+01	1.86E-03	5.22E-03	3.34E+00	1.74E+00	3.34E+00	3.60E+00	1.08E+00
CITED2	2.03E+03	1.87E-03	5.23E-03	1.68E+00	7.49E-01	1.68E+00	1.33E+02	7.92E+01
CD52	1.04E+02	1.90E-03	5.27E-03	2.55E+00	1.35E+00	2.55E+00	7.58E+00	2.97E+00
GDNF	4.62E+01	1.91E-03	5.27E-03	1.98E+00	9.82E-01	1.98E+00	3.14E+00	1.59E+00
MOSPD1	1.20E+02	1.92E-03	5.27E-03	1.37E+00	4.51E-01	1.37E+00	7.23E+00	5.29E+00
AMH	2.34E+02	1.95E-03	5.32E-03	5.35E+00	2.42E+00	5.35E+00	2.01E+01	3.75E+00
SNX24	2.68E+02	2.00E-03	5.40E-03	1.25E+00	3.21E-01	1.25E+00	1.56E+01	1.25E+01
XR_813808.3	2.86E+00	2.00E-03	5.40E-03	7.74E+00	2.95E+00	7.74E+00	2.55E-01	3.30E-02
S100A7	2.16E+00	2.13E-03	5.69E-03	5.71E+00	2.51E+00	5.71E+00	1.85E-01	3.24E-02
GRB14	4.85E+02	2.13E-03	5.69E-03	2.57E+00	1.36E+00	2.57E+00	3.58E+01	1.39E+01
MCFD2	7.45E+02	2.22E-03	5.91E-03	1.20E+00	2.63E-01	1.20E+00	4.25E+01	3.54E+01

CALM1	7.00E+03	2.25E-03	5.94E-03	1.47E+00	5.55E-01	1.47E+00	4.32E+02	2.94E+02
OTOS	6.75E+01	2.26E-03	5.95E-03	3.82E+01	5.26E+00	3.82E+01	6.60E+00	1.73E-01
XR_814835.3	2.72E+01	2.35E-03	6.14E-03	5.84E+00	2.55E+00	5.84E+00	2.33E+00	3.98E-01
FRZB	4.82E+02	2.43E-03	6.34E-03	2.07E+00	1.05E+00	2.07E+00	3.38E+01	1.63E+01
LRRN1	4.38E+02	2.61E-03	6.76E-03	2.08E+00	1.06E+00	2.08E+00	3.04E+01	1.46E+01
MTERF1	2.97E+02	2.80E-03	7.22E-03	1.24E+00	3.08E-01	1.24E+00	1.72E+01	1.39E+01
FNDC4	2.06E+02	2.81E-03	7.22E-03	1.52E+00	6.03E-01	1.52E+00	1.28E+01	8.41E+00
ZBTB24	2.35E+02	2.85E-03	7.29E-03	1.21E+00	2.78E-01	1.21E+00	1.35E+01	1.11E+01
CYP21	6.48E+01	3.04E-03	7.73E-03	2.10E+00	1.07E+00	2.10E+00	4.58E+00	2.18E+00
INSL3	6.04E+03	3.11E-03	7.87E-03	4.19E+01	5.39E+00	4.19E+01	5.92E+02	1.41E+01
(R_003031551.	1.87E+00	3.20E-03	8.05E-03	4.02E+01	5.33E+00	4.02E+01	1.82E-01	4.53E-03
CBR3	1.10E+02	3.33E-03	8.33E-03	1.64E+00	7.11E-01	1.64E+00	7.09E+00	4.33E+00
KCNJ6	5.26E+00	3.41E-03	8.50E-03	9.72E+00	3.28E+00	9.72E+00	4.81E-01	4.95E-02
LOC101908581	1.11E+00	3.52E-03	8.72E-03	1.62E+01	4.02E+00	1.62E+01	1.05E-01	6.45E-03
(R_003031414.	2.48E+02	3.60E-03	8.88E-03	1.91E+00	9.32E-01	1.91E+00	1.69E+01	8.87E+00
(R_001498619.	9.35E+00	3.64E-03	8.95E-03	2.39E+01	4.58E+00	2.39E+01	9.02E-01	3.78E-02
PTPN22	8.96E+00	3.69E-03	9.02E-03	2.71E+00	1.44E+00	2.71E+00	6.67E-01	2.46E-01
SCP2	1.03E+03	3.74E-03	9.09E-03	1.21E+00	2.73E-01	1.21E+00	5.93E+01	4.91E+01
NPM3	1.56E+02	3.81E-03	9.23E-03	1.39E+00	4.70E-01	1.39E+00	9.46E+00	6.83E+00
(R_003036118.	7.20E+00	3.89E-03	9.39E-03	4.08E+00	2.03E+00	4.08E+00	5.94E-01	1.46E-01
LOC101902742	1.82E+00	3.97E-03	9.52E-03	1.44E+01	3.84E+00	1.44E+01	1.70E-01	1.19E-02
SLC5A6	6.54E+02	4.03E-03	9.64E-03	1.28E+00	3.58E-01	1.28E+00	3.81E+01	2.97E+01
CCDC38	1.37E+01	4.06E-03	9.64E-03	1.94E+00	9.58E-01	1.94E+00	9.33E-01	4.81E-01
KRT77	2.02E+00	4.09E-03	9.67E-03	8.71E+00	3.12E+00	8.71E+00	1.85E-01	2.12E-02
(R_003034730.	2.15E+02	4.10E-03	9.67E-03	1.57E+00	6.47E-01	1.57E+00	1.37E+01	8.72E+00
APOA1	1.49E+03	4.19E-03	9.83E-03	3.39E+00	1.76E+00	3.39E+00	1.19E+02	3.49E+01
GJD3	7.90E+00	4.38E-03	1.02E-02	2.03E+00	1.02E+00	2.03E+00	5.41E-01	2.66E-01
XR_808026.3	2.53E+00	4.40E-03	1.02E-02	7.76E+00	2.96E+00	7.76E+00	2.27E-01	2.93E-02
TRAT1	3.43E+00	4.40E-03	1.02E-02	3.56E+00	1.83E+00	3.56E+00	2.70E-01	7.59E-02
ELOVL5	1.38E+03	4.57E-03	1.05E-02	1.27E+00	3.42E-01	1.27E+00	8.06E+01	6.36E+01
SH3BGRL	8.36E+02	4.63E-03	1.06E-02	1.25E+00	3.22E-01	1.25E+00	4.86E+01	3.89E+01
CPXM1	2.38E+03	4.71E-03	1.08E-02	1.43E+00	5.16E-01	1.43E+00	1.47E+02	1.03E+02
HSD11B1	1.23E+01	4.84E-03	1.10E-02	2.02E+00	1.01E+00	2.02E+00	8.47E-01	4.19E-01

LOC101904824	6.28E+00	4.86E-03	1.10E-02	2.97E+00	1.57E+00	2.97E+00	4.80E-01	1.61E-01
ALOX5AP	2.13E+01	4.90E-03	1.10E-02	1.68E+00	7.52E-01	1.68E+00	1.38E+00	8.23E-01
(R_003031734.	1.45E+01	4.91E-03	1.10E-02	2.88E+00	1.52E+00	2.88E+00	1.11E+00	3.86E-01
LOC100852061	1.55E+00	5.31E-03	1.19E-02	4.08E+01	5.35E+00	4.08E+01	1.51E-01	3.72E-03
ANKS4B	2.12E+00	6.01E-03	1.33E-02	4.93E+00	2.30E+00	4.93E+00	1.80E-01	3.66E-02
LOC514686	1.60E+00	6.03E-03	1.33E-02	3.55E+00	1.83E+00	3.55E+00	1.26E-01	3.55E-02
DAG1	7.56E+03	6.05E-03	1.33E-02	1.58E+00	6.60E-01	1.58E+00	4.81E+02	3.04E+02
LOC100141014	1.78E+00	6.06E-03	1.33E-02	3.10E+00	1.63E+00	3.10E+00	1.37E-01	4.42E-02
UCK2	1.64E+02	6.92E-03	1.51E-02	1.62E+00	7.00E-01	1.62E+00	1.06E+01	6.53E+00
GALNT13	3.80E+01	6.96E-03	1.51E-02	1.78E+00	8.35E-01	1.78E+00	2.54E+00	1.42E+00
(R_001500092.	3.18E+00	7.94E-03	1.72E-02	9.97E+00	3.32E+00	9.97E+00	2.89E-01	2.90E-02
PTX3	1.85E+02	8.19E-03	1.76E-02	2.26E+00	1.18E+00	2.26E+00	1.32E+01	5.83E+00
RHBDD1	5.74E+02	8.23E-03	1.76E-02	1.22E+00	2.86E-01	1.22E+00	3.30E+01	2.71E+01
NIPAL4	2.27E+01	8.23E-03	1.76E-02	4.32E+00	2.11E+00	4.32E+00	1.86E+00	4.30E-01
SCPEP1	1.31E+03	8.57E-03	1.83E-02	1.20E+00	2.67E-01	1.20E+00	7.48E+01	6.21E+01
DBIL5	1.02E+01	1.01E-02	2.15E-02	1.67E+00	7.39E-01	1.67E+00	6.61E-01	3.96E-01
DGKI	9.03E+01	1.05E-02	2.23E-02	1.71E+00	7.73E-01	1.71E+00	5.89E+00	3.45E+00
HEATR3	3.11E+02	1.09E-02	2.31E-02	1.19E+00	2.55E-01	1.19E+00	1.77E+01	1.48E+01
LOC112442378	3.88E+00	1.11E-02	2.31E-02	4.50E+00	2.17E+00	4.50E+00	3.22E-01	7.16E-02
TXNDC5	4.30E+03	1.11E-02	2.31E-02	1.24E+00	3.15E-01	1.24E+00	2.49E+02	2.00E+02
LOC751811	5.38E+00	1.11E-02	2.31E-02	4.62E+00	2.21E+00	4.62E+00	4.48E-01	9.71E-02
(R_003037450.	5.87E+01	1.15E-02	2.38E-02	2.82E+00	1.49E+00	2.82E+00	4.45E+00	1.58E+00
EGFL7	1.11E+03	1.19E-02	2.45E-02	1.35E+00	4.34E-01	1.35E+00	6.65E+01	4.92E+01
LOC112441481	1.85E+00	1.21E-02	2.49E-02	2.74E+01	4.78E+00	2.74E+01	1.79E-01	6.52E-03
BDKRB1	3.66E+00	1.23E-02	2.53E-02	3.26E+00	1.70E+00	3.26E+00	2.86E-01	8.77E-02
KCNF1	7.07E+01	1.24E-02	2.53E-02	8.06E+00	3.01E+00	8.06E+00	6.38E+00	7.91E-01
ANXA1	8.52E+02	1.25E-02	2.55E-02	1.45E+00	5.35E-01	1.45E+00	5.27E+01	3.64E+01
CTSC	8.73E+02	1.27E-02	2.57E-02	1.70E+00	7.63E-01	1.70E+00	5.73E+01	3.37E+01
RBM7	2.75E+02	1.28E-02	2.57E-02	1.20E+00	2.68E-01	1.20E+00	1.57E+01	1.30E+01
CYP11A1	1.26E+03	1.28E-02	2.57E-02	8.03E+00	3.01E+00	8.03E+00	1.13E+02	1.41E+01
KLRF1	1.37E+00	1.28E-02	2.57E-02	1.27E+01	3.67E+00	1.27E+01	1.28E-01	1.00E-02
CDC42EP1	2.36E+03	1.33E-02	2.65E-02	1.23E+00	2.95E-01	1.23E+00	1.37E+02	1.12E+02
CCDC184	4.42E+01	1.35E-02	2.68E-02	1.57E+00	6.51E-01	1.57E+00	2.79E+00	1.78E+00

CASS4	1.26E+01	1.36E-02	2.68E-02	1.96E+00	9.68E-01	1.96E+00	8.52E-01	4.36E-01
IL18RAP	2.75E+00	1.37E-02	2.69E-02	4.27E+00	2.09E+00	4.27E+00	2.24E-01	5.26E-02
SNTG2	1.83E+01	1.38E-02	2.70E-02	2.06E+00	1.04E+00	2.06E+00	1.27E+00	6.17E-01
OAT	1.58E+03	1.38E-02	2.70E-02	1.18E+00	2.44E-01	1.18E+00	8.99E+01	7.59E+01
ST3GAL4	1.65E+03	1.40E-02	2.72E-02	1.92E+00	9.41E-01	1.92E+00	1.12E+02	5.83E+01
(R_003030242.	4.21E+00	1.45E-02	2.82E-02	4.58E+00	2.19E+00	4.58E+00	3.46E-01	7.55E-02
(R_003035554.	1.77E+01	1.46E-02	2.82E-02	2.71E+00	1.44E+00	2.71E+00	1.32E+00	4.88E-01
FAM78A	6.34E+02	1.47E-02	2.83E-02	2.84E+00	1.51E+00	2.84E+00	4.83E+01	1.70E+01
LOC100848495	2.18E+01	1.49E-02	2.86E-02	1.43E+00	5.19E-01	1.43E+00	1.33E+00	9.32E-01
ZNF831	5.44E+00	1.55E-02	2.95E-02	2.13E+00	1.09E+00	2.13E+00	3.79E-01	1.78E-01
PNP	1.08E+03	1.60E-02	3.05E-02	1.41E+00	4.91E-01	1.41E+00	6.66E+01	4.74E+01
LOC107132002	5.11E+00	1.70E-02	3.22E-02	1.98E+00	9.86E-01	1.98E+00	3.52E-01	1.78E-01
(R_003037185.	1.06E+00	1.75E-02	3.30E-02	3.53E+01	5.14E+00	3.53E+01	1.03E-01	2.93E-03
SULT1B1	1.39E+02	1.78E-02	3.35E-02	1.41E+00	4.97E-01	1.41E+00	8.51E+00	6.03E+00
GAT	2.49E+00	1.82E-02	3.41E-02	2.41E+01	4.59E+00	2.41E+01	2.39E-01	9.94E-03
ZNF565	1.17E+02	1.92E-02	3.58E-02	1.19E+00	2.46E-01	1.19E+00	6.62E+00	5.58E+00
(R_003030631.	1.70E+00	1.98E-02	3.66E-02	3.31E+00	1.73E+00	3.31E+00	1.33E-01	4.02E-02
DHCR24	1.68E+03	1.99E-02	3.66E-02	1.66E+00	7.29E-01	1.66E+00	1.08E+02	6.53E+01
(R_003036304.	2.05E+00	2.00E-02	3.66E-02	9.13E+00	3.19E+00	9.13E+00	1.87E-01	2.05E-02
SH3GL3	1.73E+02	2.00E-02	3.66E-02	1.93E+00	9.49E-01	1.93E+00	1.19E+01	6.14E+00
XR_806886.3	4.09E+00	2.00E-02	3.66E-02	2.73E+00	1.45E+00	2.73E+00	3.06E-01	1.12E-01
CYP51A1	7.52E+02	2.00E-02	3.66E-02	1.53E+00	6.09E-01	1.53E+00	4.71E+01	3.09E+01
PHLDB3	3.12E+02	2.02E-02	3.68E-02	1.68E+00	7.48E-01	1.68E+00	2.02E+01	1.21E+01
NALCN	8.06E+01	2.08E-02	3.78E-02	3.27E+00	1.71E+00	3.27E+00	6.23E+00	1.90E+00
PLGRKT	1.47E+02	2.09E-02	3.78E-02	1.26E+00	3.32E-01	1.26E+00	8.56E+00	6.80E+00
TMPRSS6	3.43E+01	2.13E-02	3.83E-02	1.51E+00	5.94E-01	1.51E+00	2.17E+00	1.44E+00
SQLE	1.17E+03	2.15E-02	3.87E-02	1.52E+00	6.01E-01	1.52E+00	7.27E+01	4.79E+01
KCND3	1.99E+02	2.16E-02	3.87E-02	2.37E+00	1.25E+00	2.37E+00	1.44E+01	6.07E+00
NR_103815.1	2.54E+02	2.20E-02	3.93E-02	1.91E+00	9.34E-01	1.91E+00	1.71E+01	8.95E+00
SPTSSA	3.72E+02	2.28E-02	4.05E-02	1.25E+00	3.22E-01	1.25E+00	2.16E+01	1.73E+01
P3H1	2.39E+03	2.32E-02	4.12E-02	1.17E+00	2.30E-01	1.17E+00	1.35E+02	1.15E+02
KIR2DL5A	1.72E+00	2.34E-02	4.12E-02	2.29E+01	4.52E+00	2.29E+01	1.65E-01	7.20E-03
OTULINL	3.47E+03	2.34E-02	4.12E-02	1.23E+00	2.99E-01	1.23E+00	2.00E+02	1.62E+02

SAMD3	7.37E+00	2.35E-02	4.13E-02	1.66E+00	7.35E-01	1.66E+00	4.77E-01	2.87E-01
(R_003036829.	1.16E+03	2.46E-02	4.30E-02	1.40E+00	4.83E-01	1.40E+00	7.09E+01	5.07E+01
MSMO1	1.46E+03	2.49E-02	4.34E-02	1.45E+00	5.35E-01	1.45E+00	8.98E+01	6.20E+01
ZNF330	3.75E+02	2.50E-02	4.34E-02	1.28E+00	3.52E-01	1.28E+00	2.20E+01	1.72E+01
(R_001500342.	3.03E+00	2.53E-02	4.37E-02	3.75E+00	1.91E+00	3.75E+00	2.41E-01	6.41E-02
ARAF	1.17E+03	2.56E-02	4.41E-02	1.15E+00	2.00E-01	1.15E+00	6.54E+01	5.69E+01
SRGN	3.38E+03	2.57E-02	4.42E-02	2.52E+00	1.33E+00	2.52E+00	2.50E+02	9.93E+01
C6H4orf3	1.59E+03	2.61E-02	4.48E-02	1.20E+00	2.63E-01	1.20E+00	9.12E+01	7.60E+01
(R_003037434.	3.25E+01	2.67E-02	4.57E-02	2.12E+00	1.08E+00	2.12E+00	2.28E+00	1.08E+00
GRAMD1B	1.01E+03	2.68E-02	4.57E-02	1.92E+00	9.44E-01	1.92E+00	6.88E+01	3.58E+01
XR_235161.2	2.10E+02	2.70E-02	4.59E-02	2.73E+00	1.45E+00	2.73E+00	1.58E+01	5.77E+00
(R_003038026.	9.90E+00	2.71E-02	4.59E-02	2.27E+00	1.18E+00	2.27E+00	7.16E-01	3.16E-01
(R_001500581.	9.29E+01	2.76E-02	4.66E-02	1.47E+00	5.58E-01	1.47E+00	5.75E+00	3.91E+00
CTSW	8.30E+01	2.81E-02	4.73E-02	3.46E+00	1.79E+00	3.46E+00	6.56E+00	1.90E+00
XR_233810.4	2.06E+01	2.82E-02	4.73E-02	1.88E+00	9.13E-01	1.88E+00	1.40E+00	7.42E-01
SCARB1	2.30E+03	2.84E-02	4.74E-02	1.78E+00	8.33E-01	1.78E+00	1.53E+02	8.60E+01
PODNL1	1.41E+02	3.01E-02	5.00E-02	1.48E+00	5.62E-01	1.48E+00	8.79E+00	5.95E+00
HEBP2	2.43E+02	3.09E-02	5.13E-02	1.68E+00	7.47E-01	1.68E+00	1.59E+01	9.46E+00
XR_804520.3	1.30E+00	3.11E-02	5.14E-02	4.70E+00	2.23E+00	4.70E+00	1.09E-01	2.31E-02
(R_003034727.	5.61E+00	3.15E-02	5.19E-02	1.04E+01	3.38E+00	1.04E+01	5.15E-01	4.93E-02
XR_236197.4	5.47E+01	3.18E-02	5.22E-02	1.60E+00	6.76E-01	1.60E+00	3.51E+00	2.20E+00
RGN	2.09E+02	3.34E-02	5.46E-02	1.40E+00	4.85E-01	1.40E+00	1.28E+01	9.12E+00
EIF4EBP1	8.28E+02	3.45E-02	5.64E-02	1.34E+00	4.26E-01	1.34E+00	4.96E+01	3.69E+01
PEAK3	6.92E+00	3.46E-02	5.64E-02	1.58E+00	6.62E-01	1.58E+00	4.36E-01	2.76E-01
(R_001501284.	1.49E+00	3.50E-02	5.66E-02	2.74E+00	1.45E+00	2.74E+00	1.11E-01	4.06E-02
WEE2	1.06E+01	3.50E-02	5.66E-02	3.24E+00	1.70E+00	3.24E+00	8.29E-01	2.56E-01
WARS2	1.75E+02	3.55E-02	5.72E-02	1.19E+00	2.50E-01	1.19E+00	9.97E+00	8.38E+00
(R_003037968.	2.71E+00	3.66E-02	5.87E-02	2.09E+00	1.06E+00	2.09E+00	1.88E-01	9.01E-02
SCG2	3.98E+02	3.66E-02	5.87E-02	8.36E+00	3.06E+00	8.36E+00	3.58E+01	4.29E+00
PNPLA2	4.64E+02	3.68E-02	5.88E-02	1.21E+00	2.72E-01	1.21E+00	2.66E+01	2.20E+01
TOR1B	4.13E+02	3.78E-02	6.02E-02	1.18E+00	2.40E-01	1.18E+00	2.34E+01	1.99E+01
TARBP1	3.56E+02	3.82E-02	6.07E-02	1.29E+00	3.66E-01	1.29E+00	2.09E+01	1.63E+01
ANGPTL5	1.76E+01	3.89E-02	6.16E-02	8.38E+00	3.07E+00	8.38E+00	1.58E+00	1.89E-01

ANXA2	3.56E+03	3.92E-02	6.19E-02	1.18E+00	2.39E-01	1.18E+00	2.02E+02	1.72E+02
HSD17B12	7.61E+02	3.97E-02	6.25E-02	1.35E+00	4.30E-01	1.35E+00	4.55E+01	3.38E+01
(R_003029390.	2.39E+01	3.99E-02	6.26E-02	1.86E+00	8.95E-01	1.86E+00	1.62E+00	8.68E-01
VAMP5	2.88E+02	4.13E-02	6.45E-02	1.35E+00	4.31E-01	1.35E+00	1.73E+01	1.29E+01
LOC112446743	1.45E+00	4.18E-02	6.52E-02	1.45E+03	1.05E+01	1.45E+03	1.45E-01	1.00E-04
LOC100847738	1.55E+00	4.25E-02	6.61E-02	1.55E+03	1.06E+01	1.55E+03	1.55E-01	1.00E-04
LOC511161	3.91E+00	4.29E-02	6.65E-02	2.36E+00	1.24E+00	2.36E+00	2.84E-01	1.20E-01
DLL1	2.98E+02	4.31E-02	6.66E-02	1.21E+00	2.71E-01	1.21E+00	1.71E+01	1.41E+01
CYP17A1	1.26E+01	4.33E-02	6.68E-02	7.32E+00	2.87E+00	7.32E+00	1.13E+00	1.54E-01
GJB5	3.65E+01	4.39E-02	6.74E-02	4.50E+00	2.17E+00	4.50E+00	3.06E+00	6.80E-01
STAR	9.10E+02	4.54E-02	6.94E-02	4.02E+00	2.01E+00	4.02E+00	7.49E+01	1.87E+01
LOC101902413	3.56E+00	4.55E-02	6.95E-02	2.28E+00	1.19E+00	2.28E+00	2.56E-01	1.12E-01
FCAR	7.94E-01	4.82E-02	7.34E-02	7.94E+02	9.63E+00	7.94E+02	7.94E-02	1.00E-04
STYK1	8.59E+00	4.96E-02	7.53E-02	6.23E+00	2.64E+00	6.23E+00	7.51E-01	1.21E-01
FKBP5	7.91E+02	5.05E-02	7.64E-02	2.62E+00	1.39E+00	2.62E+00	5.86E+01	2.24E+01
IDI1	7.39E+02	5.31E-02	8.00E-02	1.34E+00	4.24E-01	1.34E+00	4.39E+01	3.27E+01
HCST	3.05E+00	5.42E-02	8.15E-02	2.31E+00	1.21E+00	2.31E+00	2.17E-01	9.43E-02
MYL6	6.74E+03	5.62E-02	8.42E-02	1.25E+00	3.23E-01	1.25E+00	3.94E+02	3.15E+02
LDLR	1.62E+03	5.64E-02	8.44E-02	1.54E+00	6.20E-01	1.54E+00	1.02E+02	6.62E+01
LOC112442745	1.93E+01	5.66E-02	8.44E-02	1.67E+00	7.42E-01	1.67E+00	1.23E+00	7.36E-01
C10H14orf1	1.60E+02	5.83E-02	8.68E-02	1.30E+00	3.82E-01	1.30E+00	9.49E+00	7.28E+00
(R_003030234.	1.81E+00	6.00E-02	8.87E-02	6.17E+00	2.63E+00	6.17E+00	1.56E-01	2.52E-02
(R_003030005.	1.26E+00	6.02E-02	8.87E-02	1.37E+01	3.78E+00	1.37E+01	1.18E-01	8.59E-03
HMGCR	1.15E+03	6.02E-02	8.87E-02	1.79E+00	8.43E-01	1.79E+00	7.68E+01	4.28E+01
(R_003032548.	2.86E+01	6.06E-02	8.91E-02	1.43E+00	5.14E-01	1.43E+00	1.75E+00	1.23E+00
(R_003035042.	1.13E+02	6.10E-02	8.95E-02	1.44E+00	5.27E-01	1.44E+00	6.89E+00	4.78E+00
RORB	2.00E+01	6.30E-02	9.20E-02	2.79E+00	1.48E+00	2.79E+00	1.50E+00	5.39E-01
LOC112441470	2.21E+03	6.31E-02	9.20E-02	1.68E+01	4.07E+00	1.68E+01	2.10E+02	1.25E+01
LOC104973382	2.96E+00	6.52E-02	9.48E-02	2.34E+00	1.22E+00	2.34E+00	2.14E-01	9.14E-02
XR_810083.3	6.84E+01	6.62E-02	9.60E-02	1.32E+00	3.99E-01	1.32E+00	4.06E+00	3.08E+00
LOC100336984	5.92E+00	7.33E-02	1.06E-01	1.25E+01	3.64E+00	1.25E+01	5.51E-01	4.41E-02
XR_815239.3	1.60E+01	7.46E-02	1.08E-01	1.39E+00	4.77E-01	1.39E+00	9.73E-01	6.99E-01
(R_003030615.	2.08E+00	7.67E-02	1.10E-01	1.01E+01	3.34E+00	1.01E+01	1.89E-01	1.87E-02

METTL9	1.43E+03	7.87E-02	1.13E-01	1.16E+00	2.16E-01	1.16E+00	8.08E+01	6.96E+01
PANK1	2.69E+02	7.99E-02	1.14E-01	1.21E+00	2.77E-01	1.21E+00	1.55E+01	1.28E+01
ANAPC7	1.11E+03	8.06E-02	1.15E-01	1.08E+00	1.11E-01	1.08E+00	6.02E+01	5.58E+01
FDX1	3.54E+02	8.07E-02	1.15E-01	1.32E+00	3.96E-01	1.32E+00	2.10E+01	1.59E+01
XR_003032747.	7.34E+00	8.35E-02	1.18E-01	6.15E+00	2.62E+00	6.15E+00	6.46E-01	1.05E-01
XR_003034434.	5.49E-01	8.75E-02	1.24E-01	1.44E+01	3.85E+00	1.44E+01	5.14E-02	3.57E-03
LOC100138641	1.06E+02	8.95E-02	1.26E-01	2.51E+00	1.33E+00	2.51E+00	7.85E+00	3.13E+00
XR_003035956.	5.86E+00	9.21E-02	1.30E-01	1.87E+00	9.01E-01	1.87E+00	3.92E-01	2.10E-01
S100A11	2.24E+03	9.33E-02	1.31E-01	1.21E+00	2.76E-01	1.21E+00	1.29E+02	1.06E+02
ELOVL6	3.51E+02	9.63E-02	1.34E-01	1.27E+00	3.46E-01	1.27E+00	2.06E+01	1.62E+01
QPRT	3.25E+02	9.63E-02	1.34E-01	1.18E+00	2.44E-01	1.18E+00	1.85E+01	1.56E+01
XR_003034037.	1.84E+00	9.80E-02	1.36E-01	9.91E+00	3.31E+00	9.91E+00	1.71E-01	1.72E-02
XR_003031416.	2.57E+01	9.93E-02	1.38E-01	2.55E+00	1.35E+00	2.55E+00	1.90E+00	7.44E-01
KCNE4	1.93E+02	1.05E-01	1.45E-01	2.55E+00	1.35E+00	2.55E+00	1.43E+01	5.61E+00
LHCGR	1.32E+02	1.08E-01	1.49E-01	6.08E+00	2.60E+00	6.08E+00	1.16E+01	1.90E+00
INSIG1	7.50E+02	1.08E-01	1.49E-01	1.49E+00	5.74E-01	1.49E+00	4.64E+01	3.12E+01
LRP11	2.06E+02	1.09E-01	1.50E-01	1.43E+00	5.20E-01	1.43E+00	1.27E+01	8.83E+00
XR_001500563.	1.87E+00	1.11E-01	1.52E-01	1.84E+00	8.81E-01	1.84E+00	1.21E-01	6.58E-02
NDNF	6.63E+01	1.15E-01	1.57E-01	1.47E+00	5.58E-01	1.47E+00	4.13E+00	2.80E+00
LOC781813	1.39E+02	1.17E-01	1.59E-01	1.20E+00	2.62E-01	1.20E+00	7.97E+00	6.65E+00
TDGF1	6.65E+00	1.19E-01	1.62E-01	2.61E+00	1.38E+00	2.61E+00	4.94E-01	1.90E-01
XR_001501446.	3.43E+00	1.21E-01	1.63E-01	1.94E+00	9.53E-01	1.94E+00	2.33E-01	1.20E-01
XR_001494888.	4.31E-01	1.24E-01	1.68E-01	4.30E+02	8.75E+00	4.30E+02	4.30E-02	1.00E-04
FKBP11	7.11E+02	1.26E-01	1.70E-01	1.18E+00	2.39E-01	1.18E+00	4.03E+01	3.42E+01
SLC35C2	6.06E+02	1.27E-01	1.70E-01	1.10E+00	1.34E-01	1.10E+00	3.33E+01	3.04E+01
XR_812966.3	1.01E+02	1.27E-01	1.70E-01	4.04E+00	2.01E+00	4.04E+00	8.37E+00	2.07E+00
TEX45	3.86E+00	1.32E-01	1.77E-01	2.67E+00	1.42E+00	2.67E+00	2.90E-01	1.09E-01
GSTA1	2.95E+03	1.32E-01	1.77E-01	2.62E+00	1.39E+00	2.62E+00	2.21E+02	8.44E+01
NR_038147.1	2.47E+02	1.37E-01	1.82E-01	1.27E+00	3.50E-01	1.27E+00	1.45E+01	1.14E+01
PCSK6	6.50E+02	1.41E-01	1.88E-01	1.28E+00	3.60E-01	1.28E+00	3.77E+01	2.94E+01
IFI35	2.12E+02	1.46E-01	1.94E-01	1.16E+00	2.13E-01	1.16E+00	1.19E+01	1.03E+01
GRAMD2B	3.96E+02	1.48E-01	1.95E-01	1.29E+00	3.65E-01	1.29E+00	2.33E+01	1.81E+01
CC2D2B	4.27E+02	1.61E-01	2.12E-01	1.20E+00	2.64E-01	1.20E+00	2.44E+01	2.03E+01

id-TRGC3	1.39E+01	1.65E-01	2.16E-01	2.28E+00	1.19E+00	2.28E+00	1.00E+00	4.40E-01
(R_003034613.	1.62E+00	1.65E-01	2.17E-01	6.66E+00	2.73E+00	6.66E+00	1.43E-01	2.15E-02
id-LOC407201	6.16E-01	1.67E-01	2.18E-01	5.21E+00	2.38E+00	5.21E+00	5.17E-02	9.92E-03
XR_236208.4	5.07E+00	1.67E-01	2.18E-01	3.91E+00	1.97E+00	3.91E+00	4.10E-01	1.05E-01
PRF1	1.15E+01	1.76E-01	2.28E-01	2.61E+00	1.38E+00	2.61E+00	8.64E-01	3.31E-01
NR_132744.1	2.62E+02	1.76E-01	2.29E-01	1.13E+00	1.80E-01	1.13E+00	1.47E+01	1.29E+01
TSC22D3	1.49E+03	1.77E-01	2.29E-01	1.18E+00	2.36E-01	1.18E+00	8.44E+01	7.16E+01
EMID1	1.38E+03	1.80E-01	2.32E-01	1.80E+00	8.50E-01	1.80E+00	9.20E+01	5.10E+01
FADS2	9.38E+02	1.86E-01	2.39E-01	1.25E+00	3.22E-01	1.25E+00	5.46E+01	4.37E+01
BCDIN3D	1.33E+02	1.89E-01	2.43E-01	1.10E+00	1.38E-01	1.10E+00	7.33E+00	6.67E+00
d-LOC11244641	6.53E-01	1.91E-01	2.44E-01	5.55E+00	2.47E+00	5.55E+00	5.60E-02	1.01E-02
PPP1R14A	1.66E+02	1.93E-01	2.47E-01	1.50E+00	5.85E-01	1.50E+00	1.05E+01	6.97E+00
CTIF	5.48E+02	1.97E-01	2.50E-01	1.22E+00	2.86E-01	1.22E+00	3.16E+01	2.59E+01
LOC107132529	6.26E+00	1.97E-01	2.50E-01	1.47E+00	5.52E-01	1.47E+00	3.91E-01	2.66E-01
RDH11	4.51E+02	1.99E-01	2.52E-01	1.26E+00	3.33E-01	1.26E+00	2.64E+01	2.09E+01
XR_233298.4	4.03E-01	2.01E-01	2.54E-01	4.02E+02	8.65E+00	4.02E+02	4.02E-02	1.00E-04
NOP53	4.46E+03	2.02E-01	2.54E-01	1.16E+00	2.11E-01	1.16E+00	2.52E+02	2.18E+02
ABAT	8.51E+02	2.02E-01	2.55E-01	1.23E+00	3.03E-01	1.23E+00	4.93E+01	3.99E+01
ACLY	2.92E+03	2.06E-01	2.58E-01	1.15E+00	1.99E-01	1.15E+00	1.63E+02	1.42E+02
TSKU	1.51E+03	2.12E-01	2.66E-01	1.19E+00	2.54E-01	1.19E+00	8.62E+01	7.23E+01
PRORSD1	1.99E+02	2.13E-01	2.66E-01	1.31E+00	3.89E-01	1.31E+00	1.19E+01	9.09E+00
KIR3DL2	1.23E+00	2.19E-01	2.73E-01	7.91E+00	2.98E+00	7.91E+00	1.10E-01	1.39E-02
KRT1	9.97E-01	2.25E-01	2.80E-01	1.80E+00	8.51E-01	1.80E+00	6.41E-02	3.56E-02
ADGRF2	2.94E+00	2.26E-01	2.80E-01	5.43E+00	2.44E+00	5.43E+00	2.50E-01	4.61E-02
(R_001500425.:	1.39E+00	2.27E-01	2.80E-01	2.17E+01	4.44E+00	2.17E+01	1.33E-01	6.14E-03
STBD1	4.12E+02	2.37E-01	2.92E-01	1.13E+00	1.78E-01	1.13E+00	2.31E+01	2.04E+01
PLOD1	5.08E+03	2.38E-01	2.93E-01	1.08E+00	1.16E-01	1.08E+00	2.77E+02	2.55E+02
(R_001501097.:	2.81E+01	2.40E-01	2.95E-01	2.19E+00	1.13E+00	2.19E+00	2.01E+00	9.18E-01
XR_815563.3	5.50E+02	2.42E-01	2.96E-01	1.13E+00	1.72E-01	1.13E+00	3.06E+01	2.72E+01
(R_003029940.	1.13E+00	2.42E-01	2.96E-01	4.55E+00	2.19E+00	4.55E+00	9.61E-02	2.11E-02
(R_003030041.	5.15E+00	2.44E-01	2.97E-01	1.57E+00	6.49E-01	1.57E+00	3.27E-01	2.09E-01
RBBP9	3.31E+02	2.56E-01	3.11E-01	1.11E+00	1.55E-01	1.11E+00	1.83E+01	1.64E+01
PKNOX2	7.69E+02	2.61E-01	3.17E-01	1.15E+00	1.99E-01	1.15E+00	4.32E+01	3.76E+01

A4GALT	1.50E+03	2.64E-01	3.20E-01	2.20E+00	1.14E+00	2.20E+00	1.07E+02	4.84E+01
RARRES2	3.99E+03	2.64E-01	3.20E-01	1.34E+00	4.25E-01	1.34E+00	2.40E+02	1.79E+02
(R_003033396.	1.06E+00	2.81E-01	3.38E-01	5.99E+00	2.58E+00	5.99E+00	9.17E-02	1.53E-02
CAPZA2	6.04E+02	2.85E-01	3.43E-01	1.06E+00	8.69E-02	1.06E+00	3.27E+01	3.08E+01
SERBP1	4.61E+03	2.89E-01	3.47E-01	1.05E+00	6.44E-02	1.05E+00	2.48E+02	2.37E+02
MT1E-2	2.88E+01	2.92E-01	3.49E-01	1.77E+00	8.24E-01	1.77E+00	1.92E+00	1.09E+00
(R_003032455.	2.52E+01	2.93E-01	3.50E-01	1.19E+00	2.56E-01	1.19E+00	1.43E+00	1.19E+00
(R_003034854.	1.64E+00	2.97E-01	3.54E-01	3.20E+00	1.68E+00	3.20E+00	1.27E-01	3.97E-02
CD96	9.25E+00	3.07E-01	3.64E-01	1.72E+00	7.84E-01	1.72E+00	6.10E-01	3.54E-01
ASS1	9.78E+01	3.07E-01	3.64E-01	1.40E+00	4.84E-01	1.40E+00	5.98E+00	4.27E+00
XR_809763.3	2.88E+00	3.08E-01	3.65E-01	5.26E+00	2.39E+00	5.26E+00	2.44E-01	4.64E-02
(R_003034435.	3.65E+00	3.09E-01	3.65E-01	3.92E+00	1.97E+00	3.92E+00	2.96E-01	7.56E-02
RIN1	2.78E+01	3.20E-01	3.77E-01	2.40E+00	1.26E+00	2.40E+00	2.01E+00	8.39E-01
FAM3A	2.56E+02	3.21E-01	3.77E-01	1.10E+00	1.36E-01	1.10E+00	1.41E+01	1.28E+01
PTCH2	8.95E+02	3.27E-01	3.84E-01	1.73E+00	7.87E-01	1.73E+00	5.95E+01	3.45E+01
PLCL2	9.76E+02	3.38E-01	3.95E-01	1.17E+00	2.30E-01	1.17E+00	5.52E+01	4.70E+01
LOC780982	2.98E+01	3.48E-01	4.06E-01	1.19E+00	2.49E-01	1.19E+00	1.69E+00	1.42E+00
(R_001501206.:	1.54E+00	3.48E-01	4.06E-01	4.29E+00	2.10E+00	4.29E+00	1.28E-01	2.98E-02
SLC2A6	4.84E+01	3.50E-01	4.07E-01	1.45E+00	5.37E-01	1.45E+00	2.96E+00	2.04E+00
FDFT1	1.62E+03	3.54E-01	4.10E-01	1.14E+00	1.83E-01	1.14E+00	9.07E+01	7.99E+01
XR_238288.4	1.77E+00	3.54E-01	4.10E-01	4.50E+00	2.17E+00	4.50E+00	1.48E-01	3.28E-02
RGS17	2.53E+01	3.57E-01	4.12E-01	1.61E+00	6.87E-01	1.61E+00	1.64E+00	1.02E+00
TM7SF2	2.49E+02	3.62E-01	4.17E-01	1.30E+00	3.77E-01	1.30E+00	1.47E+01	1.13E+01
C16H1orf167	3.90E+01	3.86E-01	4.44E-01	9.58E-01	-6.14E-02	-1.04E+00	2.02E+00	2.10E+00
PITPNB	1.49E+03	4.08E-01	4.68E-01	1.04E+00	5.62E-02	1.04E+00	7.99E+01	7.69E+01
MBD2	7.44E+02	4.19E-01	4.79E-01	1.06E+00	8.35E-02	1.06E+00	4.04E+01	3.81E+01
BHMT2	8.96E+00	4.21E-01	4.81E-01	1.43E+00	5.11E-01	1.43E+00	5.46E-01	3.83E-01
id-LOC781951	4.96E+00	4.23E-01	4.82E-01	9.09E-01	-1.37E-01	-1.10E+00	2.45E-01	2.69E-01
HPGD	8.53E+01	4.40E-01	5.00E-01	1.57E+00	6.48E-01	1.57E+00	5.41E+00	3.45E+00
PLA2G3	9.70E+00	4.41E-01	5.01E-01	1.41E+00	4.92E-01	1.41E+00	5.96E-01	4.23E-01
LOC100139916	1.29E+01	4.48E-01	5.07E-01	2.69E+00	1.43E+00	2.69E+00	9.62E-01	3.57E-01
CENPM	3.53E+02	4.54E-01	5.12E-01	1.08E+00	1.08E-01	1.08E+00	1.92E+01	1.78E+01
PLEKHO2	7.22E+02	4.55E-01	5.12E-01	1.09E+00	1.26E-01	1.09E+00	3.96E+01	3.62E+01

TNNI1	7.05E+01	4.57E-01	5.14E-01	2.42E+00	1.27E+00	2.42E+00	5.19E+00	2.15E+00
MVK	1.59E+03	4.65E-01	5.22E-01	1.17E+00	2.26E-01	1.17E+00	8.98E+01	7.67E+01
STEAP3	2.48E+02	4.70E-01	5.27E-01	1.11E+00	1.49E-01	1.11E+00	1.37E+01	1.23E+01
MTHFD2	1.64E+02	4.75E-01	5.31E-01	1.08E+00	1.07E-01	1.08E+00	8.92E+00	8.28E+00
NR1H3	4.51E+02	4.77E-01	5.32E-01	1.07E+00	9.50E-02	1.07E+00	2.44E+01	2.28E+01
MTCH1	5.90E+03	4.94E-01	5.49E-01	1.05E+00	6.45E-02	1.05E+00	3.18E+02	3.04E+02
CRTAM	4.08E+00	4.98E-01	5.52E-01	3.65E+00	1.87E+00	3.65E+00	3.22E-01	8.81E-02
GRIA2	7.77E+00	5.09E-01	5.64E-01	1.35E+00	4.28E-01	1.35E+00	4.78E-01	3.55E-01
LOC112445109	2.17E+00	5.15E-01	5.69E-01	1.22E+00	2.92E-01	1.22E+00	1.26E-01	1.03E-01
SLC5A11	1.01E+01	5.22E-01	5.76E-01	5.73E+00	2.52E+00	5.73E+00	8.79E-01	1.53E-01
CCND3	1.50E+03	5.31E-01	5.85E-01	1.10E+00	1.40E-01	1.10E+00	8.27E+01	7.51E+01
CYB5R3	6.56E+03	5.40E-01	5.93E-01	1.10E+00	1.32E-01	1.10E+00	3.60E+02	3.29E+02
KCNK3	6.37E+01	5.44E-01	5.96E-01	1.78E+00	8.33E-01	1.78E+00	4.29E+00	2.41E+00
CLCNKA	3.46E+00	5.49E-01	6.00E-01	1.50E+00	5.88E-01	1.50E+00	2.13E-01	1.42E-01
GALNT10	1.61E+03	5.58E-01	6.09E-01	1.06E+00	7.88E-02	1.06E+00	8.67E+01	8.21E+01
XR_001494305.	5.76E+01	5.61E-01	6.10E-01	1.16E+00	2.08E-01	1.16E+00	3.22E+00	2.79E+00
CCDC69	3.61E+02	5.79E-01	6.29E-01	1.28E+00	3.56E-01	1.28E+00	2.12E+01	1.66E+01
SLC35F2	5.25E+01	5.81E-01	6.30E-01	1.20E+00	2.66E-01	1.20E+00	3.00E+00	2.50E+00
FKBP10	4.96E+03	5.83E-01	6.31E-01	1.04E+00	6.04E-02	1.04E+00	2.66E+02	2.55E+02
LOC112449109	3.12E+00	5.89E-01	6.36E-01	2.63E+00	1.39E+00	2.63E+00	2.31E-01	8.80E-02
STEAP1	9.77E+00	5.98E-01	6.44E-01	1.54E+00	6.26E-01	1.54E+00	6.20E-01	4.02E-01
FGFR4	7.49E+01	6.07E-01	6.52E-01	1.04E+00	5.76E-02	1.04E+00	4.01E+00	3.85E+00
XR_003034136.	5.72E+00	6.14E-01	6.58E-01	3.64E+00	1.86E+00	3.64E+00	4.61E-01	1.27E-01
XR_809117.3	1.34E+00	6.23E-01	6.67E-01	1.42E+00	5.05E-01	1.42E+00	8.02E-02	5.65E-02
FSTL3	2.65E+02	6.50E-01	6.94E-01	9.58E-01	-6.26E-02	-1.04E+00	1.36E+01	1.42E+01
LOC100139931	4.27E+00	6.65E-01	7.09E-01	1.88E+00	9.13E-01	1.88E+00	2.86E-01	1.52E-01
LARGE2	5.42E+02	6.69E-01	7.12E-01	1.44E+00	5.25E-01	1.44E+00	3.32E+01	2.31E+01
ACAT2	9.40E+02	6.87E-01	7.29E-01	1.10E+00	1.35E-01	1.10E+00	5.18E+01	4.71E+01
LOC107131588	2.66E+00	6.95E-01	7.37E-01	1.47E+00	5.53E-01	1.47E+00	1.63E-01	1.11E-01
PLOD3	1.88E+03	7.09E-01	7.49E-01	1.03E+00	4.24E-02	1.03E+00	1.00E+02	9.73E+01
KCNJ4	2.66E+01	7.15E-01	7.55E-01	9.33E+00	3.22E+00	9.33E+00	2.42E+00	2.60E-01
XR_003037291.	1.14E+02	7.20E-01	7.58E-01	1.00E+00	6.31E-05	1.00E+00	6.00E+00	6.00E+00
XR_003036488.	2.01E+00	7.26E-01	7.61E-01	1.64E+00	7.14E-01	1.64E+00	1.27E-01	7.73E-02

LOC614050	1.83E+00	7.26E-01	7.61E-01	2.00E+00	1.00E+00	2.00E+00	1.23E-01	6.14E-02
IDH1	1.51E+03	7.27E-01	7.61E-01	1.00E+00	3.76E-03	1.00E+00	7.98E+01	7.96E+01
LOC781773	3.99E+00	7.65E-01	7.99E-01	1.09E+00	1.23E-01	1.09E+00	2.19E-01	2.01E-01
(R_003033942.	8.39E+00	7.82E-01	8.15E-01	2.46E+00	1.30E+00	2.46E+00	6.08E-01	2.47E-01
(R_003030361.	4.01E-01	7.86E-01	8.17E-01	7.11E+00	2.83E+00	7.11E+00	3.52E-02	4.96E-03
ITGAD	3.76E+00	8.00E-01	8.30E-01	2.00E+00	1.00E+00	2.00E+00	2.56E-01	1.28E-01
XR_814656.3	3.24E+00	8.27E-01	8.57E-01	1.60E+00	6.81E-01	1.60E+00	2.06E-01	1.28E-01
COLGALT1	2.93E+03	8.38E-01	8.65E-01	9.94E-01	-8.03E-03	-1.01E+00	1.54E+02	1.54E+02
CXCL14	1.20E+02	8.38E-01	8.65E-01	3.38E+00	1.76E+00	3.38E+00	9.47E+00	2.80E+00
CHST8	1.82E+02	8.43E-01	8.69E-01	3.82E+00	1.93E+00	3.82E+00	1.48E+01	3.88E+00
FITM2	4.65E+02	8.46E-01	8.70E-01	1.00E+00	-3.75E-04	-1.00E+00	2.44E+01	2.44E+01
REG4	7.29E+00	8.61E-01	8.84E-01	6.79E+00	2.76E+00	6.79E+00	6.44E-01	9.49E-02
LRPAP1	1.81E+03	8.73E-01	8.94E-01	1.02E+00	2.70E-02	1.02E+00	9.62E+01	9.45E+01
SLC25A19	3.42E+02	8.87E-01	9.06E-01	9.99E-01	-9.38E-04	-1.00E+00	1.79E+01	1.79E+01
(R_003036663.	1.70E+00	9.02E-01	9.20E-01	1.74E+00	7.98E-01	1.74E+00	1.08E-01	6.23E-02
(R_003033094.	4.99E+01	9.11E-01	9.27E-01	1.17E+00	2.29E-01	1.17E+00	2.79E+00	2.38E+00
LSS	1.40E+03	9.17E-01	9.30E-01	1.06E+00	8.77E-02	1.06E+00	7.58E+01	7.13E+01
EBF4	9.13E+02	9.17E-01	9.30E-01	1.00E+00	4.50E-03	1.00E+00	4.82E+01	4.80E+01
DHCR7	1.17E+03	9.25E-01	9.36E-01	1.20E+00	2.66E-01	1.20E+00	6.71E+01	5.58E+01
LOC107132995	3.56E+00	9.26E-01	9.36E-01	1.12E+00	1.69E-01	1.12E+00	1.98E-01	1.76E-01
(R_003031849.	1.91E+02	9.32E-01	9.40E-01	1.02E+00	2.73E-02	1.02E+00	1.01E+01	9.96E+00
C5H12orf75	6.81E+01	9.46E-01	9.51E-01	1.31E+00	3.93E-01	1.31E+00	4.04E+00	3.08E+00
RSPO4	1.24E+01	9.83E-01	9.87E-01	1.63E+00	7.02E-01	1.63E+00	7.98E-01	4.90E-01
SLC16A7	7.43E+01	9.95E-01	9.97E-01	1.18E+00	2.38E-01	1.18E+00	4.20E+00	3.56E+00
ARTN	5.29E+00	9.98E-01	9.98E-01	1.02E+00	3.17E-02	1.02E+00	2.78E-01	2.72E-01

S6 Table. Top 20 pathways enriched for the early genes in cluster 1. Pathways were identified from Kyoto Encyclopedia of Genes and Genomes (KEGG) pathway analysis using Database for Annotation, Visualization, and Integrated Discovery (DAVID) knowledgebase.

**KEGG Term**	**Count**	**P-value**	**Pop Hits**	**Percentage**
bta01100:Metabolic pathways	185	7.35E-15	1235	14.98
bta05012:Parkinson's disease	40	4.83E-10	152	26.32
bta00190:Oxidative phosphorylation	38	5.51E-10	140	27.14
bta01130:Biosynthesis of antibiotics	48	5.86E-10	206	23.30
bta05010:Alzheimer's disease	41	1.84E-08	178	23.03
bta05016:Huntington's disease	43	5.54E-08	199	21.61
bta04932:Non-alcoholic fatty liver disease (NAFLD)	36	2.92E-07	160	22.50
bta01200:Carbon metabolism	28	5.10E-07	109	25.69
bta01230:Biosynthesis of amino acids	20	7.49E-06	71	28.17
bta04260:Cardiac muscle contraction	20	4.70E-05	80	25.00
bta04360:Axon guidance	25	1.94E-04	125	20.00
bta00051:Fructose and mannose metabolism	11	2.11E-04	31	35.48
bta05230:Central carbon metabolism in cancer	15	7.93E-04	62	24.19
bta00010:Glycolysis / Gluconeogenesis	15	9.39E-04	63	23.81
bta00052:Galactose metabolism	10	0.001035	31	32.26
bta01210:2-Oxocarboxylic acid metabolism	7	0.003367	18	38.89
bta00020:Citrate cycle (TCA cycle)	9	0.003534	30	30.00
bta00230:Purine metabolism	26	0.011116	176	14.77
bta05210:Colorectal cancer	13	0.011599	66	19.70
bta05200:Pathways in cancer	49	0.014493	398	12.31

S7 Table. Top 20 pathways enriched for the late genes in cluster 2. Pathways were identified from Kyoto Encyclopedia of Genes and Genomes (KEGG) pathway analysis using Database for Annotation, Visualization, and Integrated Discovery (DAVID) knowledgebase.

KEGG Term	Count	P-value	Pop Hits	Percentage
bta04510:Focal adhesion	54	5.83E-19	208	25.96
bta04151:PI3K-Akt signaling pathway	64	1.90E-14	347	18.44
bta04512:ECM-receptor interaction	30	4.27E-14	87	34.48
bta05200:Pathways in cancer	60	8.22E-10	398	15.08
bta04810:Regulation of actin cytoskeleton	37	6.40E-08	212	17.45
bta04611:Platelet activation	27	1.04E-07	127	21.26
bta05146:Amoebiasis	25	1.32E-07	112	22.32
bta05414:Dilated cardiomyopathy	21	3.67E-07	86	24.42
bta05205:Proteoglycans in cancer	33	1.94E-06	203	16.26
bta05410:Hypertrophic cardiomyopathy (HCM)	19	2.36E-06	80	23.75
bta04015:Rap1 signaling pathway	33	6.17E-06	214	15.42
bta05412:Arrhythmogenic right ventricular cardiomyopathy (ARVC)	16	1.18E-05	65	24.62
bta04520:Adherens junction	16	2.12E-05	68	23.53
bta04022:cGMP-PKG signaling pathway	26	2.72E-05	159	16.35
bta05218:Melanoma	16	4.33E-05	72	22.22
bta04974:Protein digestion and absorption	17	7.68E-05	84	20.24
bta04068:FoxO signaling pathway	22	1.00E-04	132	16.67
bta04921:Oxytocin signaling pathway	23	1.41E-04	145	15.86
bta04725:Cholinergic synapse	19	2.16E-04	110	17.27
bta04020:Calcium signaling pathway	26	4.60E-04	189	13.76

S8 Table. The pathways enriched for the genes in cluster 3. Pathways were identified from Kyoto Encyclopedia of Genes and Genomes (KEGG) pathway analysis using Database for Annotation, Visualization, and Integrated Discovery (DAVID) knowledgebase.

**KEGG Term**	**Count**	**P-value**	**Pop Hits**	**Percentage**
bta04724:Glutamatergic synapse	9	0.004596	113	7.96460177
bta04610:Complement and coagulation cascades	7	0.007168	74	9.45945946
bta04975:Fat digestion and absorption	5	0.01985	45	11.1111111
bta04730:Long-term depression	5	0.047364	59	8.47457627
bta04080:Neuroactive ligand-receptor interaction	12	0.076422	292	4.10958904
bta04330:Notch signaling pathway	4	0.094725	47	8.5106383

S9 Table. Top 20 pathways enriched for the late genes in cluster 4. Pathways were identified from Kyoto Encyclopedia of Genes and Genomes (KEGG) pathway analysis using Database for Annotation, Visualization, and Integrated Discovery (DAVID) knowledgebase.

KEGG Term	Count	P-value	Pop Hits	Percentage
bta01100:Metabolic pathways	56	1.75E-09	1235	4.53441296
bta00100:Steroid biosynthesis	8	7.27E-08	20	40
bta01130:Biosynthesis of antibiotics	18	7.09E-07	206	8.73786408
bta04913:Ovarian steroidogenesis	8	5.64E-05	50	16
bta01040:Biosynthesis of unsaturated fatty acids	6	9.66E-05	24	25
bta01212:Fatty acid metabolism	7	3.68E-04	48	14.5833333
bta00900:Terpenoid backbone biosynthesis	5	8.71E-04	22	22.7272727
bta04925:Aldosterone synthesis and secretion	7	0.005008	79	8.86075949
bta00140:Steroid hormone biosynthesis	6	0.005587	57	10.5263158
bta04977:Vitamin digestion and absorption	4	0.009304	22	18.1818182
bta00062:Fatty acid elongation	4	0.014816	26	15.3846154
bta04270:Vascular smooth muscle contraction	7	0.032633	119	5.88235294
bta03320:PPAR signaling pathway	5	0.05209	70	7.14285714
bta00330:Arginine and proline metabolism	4	0.079019	50	8
bta04650:Natural killer cell mediated cytotoxicity	6	0.086757	117	5.12820513
bta04340:Hedgehog signaling pathway	3	0.089385	25	12
bta00310:Lysine degradation	4	0.094478	54	7.40740741

S10 Table. The number of genes extracted from the heatmap and those from the list that mapped onto IPA and DAVID knowledge bases for each cluster.

**Cluster**	**Number of genes**			
	**Heatmap**	**Mapped to IPA**	**DAVID (*Homo sapiens*)**	**DAVID (*Bos taurus*)**
1	2052	1654	1589	1773
2	1781	1402	1290	1415
3	871	534	477	576
4	516	376	350	393

S11 Table. Chemical upstream regulators (including endogenous molecules) associated with the strong clusters (clusters 1 and 4).

**Cluster 1**	**Upstream Regulator**	**Molecule Type**	**Activation z-score**	**p-value of overlap**
	GSKJ4	chemical reagent	5.048	7.57E-18
	CD 437	chemical drug	6.173	1.69E-12
	ST1926	chemical drug	5.799	6.37E-12
	metribolone	chemical reagent	-7.648	1.87E-10
	actinonin	chemical reagent	3.317	2.70E-09
	IND S1	chemical - kinase inhibitor	0.302	1.09E-07
	5-fluorouracil	chemical drug	2.671	1.56E-08
	trichostatin A	chemical drug	-3.257	8.85E-07
	PCGEM1	other	-3.696	9.53E-07
	1,2-dithiol-3-thione	chemical reagent	-4.967	1.28E-06
	decitabine	chemical drug	-3.542	2.35E-06
	beta-estradiol	chemical - endogenous mammalian	-7.079	2.93E-06
	mono-(2-ethylhexyl)phthalate	chemical toxicant	-5.073	3.01E-06
	tazemetostat	chemical drug	1.121	3.88E-06
	SP2509	chemical reagent	1.254	4.47E-06
	torin1	chemical reagent	5.339	9.53E-06
	D-glucose	chemical - endogenous mammalian	0.006	1.18E-05
	MEL S3	chemical - kinase inhibitor	0.632	2.47E-05
	miR-127 prodrug	chemical reagent	2.234	3.96E-05
	MEL T1	chemical - kinase inhibitor	-0.378	7.27E-05
**Cluster 4**	dexamethasone	chemical drug	2.675	1.30E-19
	chloropromazine	chemical drug	2.207	3.00E-18
	(-)-norephedrine	chemical drug	3.317	3.15E-17
	lysophosphatidylcholine	chemical - other	3.873	2.52E-15
	beta-estradiol	chemical - endogenous mammalian	3.597	1.19E-13
	elaidic acid	chemical - endogenous mammalian	4.084	2.33E-13
	isoquercitrin	chemical drug	2.985	4.22E-13
	25-hydroxycholesterol	chemical reagent	-1.444	4.82E-11
	pitavastatin	chemical drug	2.937	6.13E-11
	atorvastatin	chemical drug	2.456	3.97E-10
	ezetimibe	chemical drug	2.81	1.47E-09
	rosuvastatin	chemical drug	2.345	1.84E-09
	dihydrotestosterone	chemical - endogenous mammalian	2.503	2.52E-09
	lithium chloride	chemical drug	2.557	2.86E-09
	PD98059	chemical - kinase inhibitor	-3.495	3.64E-09
	3-deoxy-2-octulosonic acid(2)-lipid A	chemical - endogenous non-mammalian	-0.378	5.79E-09
	Gm35986	other	-2.216	7.50E-09

	TO-901317	chemical reagent	1.295	3.87E-09
	sterol	chemical - endogenous mammalian	-2.587	1.31E-08
	bucladesine	chemical toxicant	4.579	1.33E-08

Activation z-scores were not generated for most of the upstream regulators in Cluster 3. These could be due to the fact that these genes are expressed throughout gestation and are not differentially expressed.

S12 Table. Chemical upstream regulators (including endogenous molecules) associated with the weak clusters (clusters 2 and 3).

**Cluster 2**	**Upstream Regulator**	**Molecule Type**	**Activation z-score**	**p-value of overlap**
	beta-estradiol	chemical - endogenous mammalian	6.994	2.34E-13
	dexamethasone	chemical drug	4.76	4.53E-13
	8-bromo-cAMP	chemical reagent	7.755	1.33E-12
	thioacetamide	chemical toxicant	4.372	1.54E-11
	medroxyprogesterone acetate	chemical drug	7.159	1.05E-10
	trans-hydroxytamoxifen	chemical drug	-2.135	5.21E-10
	halofuginone	chemical drug	-2.216	7.47E-10
	triptolide	chemical drug	-5.053	1.08E-09
	lipopolysaccharide	chemical drug	6.225	1.47E-09
	D-glucose	chemical - endogenous mammalian	5.076	7.70E-09
	trichostatin A	chemical drug	1.985	1.41E-08
	estrogen	chemical drug	2.554	1.23E-07
	2-amino-5-phosphonovaleric acid	chemical - other	-0.482	2.60E-07
	branched chain amino acids	chemical drug	-3	2.88E-07
	prednisolone	chemical drug	-1.147	7.09E-07
	thapsigargin	chemical toxicant	2.22	9.11E-07
	ramipril	chemical drug	-1.943	1.46E-06
	dihydrotestosterone	chemical - endogenous mammalian	3.025	1.60E-06
	aspirin	chemical drug	-4.891	1.78E-06
	levodopa	chemical - endogenous mammalian	0.472	1.83E-06
	MM-589	chemical reagent	-1.69	2.47E-06
	PTC-209	chemical reagent	-1.69	2.47E-06
**Cluster 3**	AMP	chemical - endogenous mammalian		3.22E-03
	sulpiride	chemical drug		7.81E-03
	Congo Red	chemical toxicant		7.81E-03
	2-(3-hydroxypropoxy)calcitriol	chemical drug		1.41E-02
	tetrodotoxin	chemical drug		1.67E-02
	6-amino-4-(4-phenoxyphenylethylamino)quinazoline	chemical reagent		1.79E-02
	ziprasidone	chemical drug		1.79E-02
	tolbutamide	chemical drug		2.20E-02

	CGP 42112	chemical reagent		2.20E-02
	formononetin	chemical - endogenous non-mammalian		2.20E-02
	5'-adenylyl (beta,gamma-methylene)diphosphonate	chemical reagent		2.36E-02
	pregnanolone	chemical - endogenous mammalian		2.36E-02
	taurolithocholate-3-sulfate	chemical - endogenous mammalian		2.36E-02
	soraphen-A1alpha	chemical - endogenous non-mammalian		2.36E-02
	A-443654	chemical reagent		2.36E-02
	tiapride	chemical drug		2.36E-02
	ATP-gamma-S	chemical reagent		2.08E-02
	CORT-108297	chemical reagent		2.36E-02
	betel quid extract	chemical reagent		2.36E-02
	RECTAS	chemical reagent		2.36E-02

Activation z-scores were not generated for most of the upstream regulators in Cluster 3. These could be due to the fact that these genes are expressed throughout gestation and are not differentially expressed.
