# Supplementary material for: Analysis of Upstream Regulators, Networks, and Pathways Associated With the Expression Patterns of Polycystic Ovary Syndrome Candidate Genes During Fetal Ovary Development
Source: Front Genet. 2022 Feb 7;12:762177. doi: 10.3389/fgene.2021.762177 (PMC8860493; doi:10.3389/fgene.2021.762177)
Supplement: Supplementary file 2 [file DataSheet1.PDF]

S1 Fig. Scatterplots showing the expression of PCOS candidate genes across gestation. The expression patterns are in three groups according to Pearson's correlation and GSA outcomes;
A. early, B. throughout, and C. late genes. The red circle shows the results of two sequencing runs of the same sample.

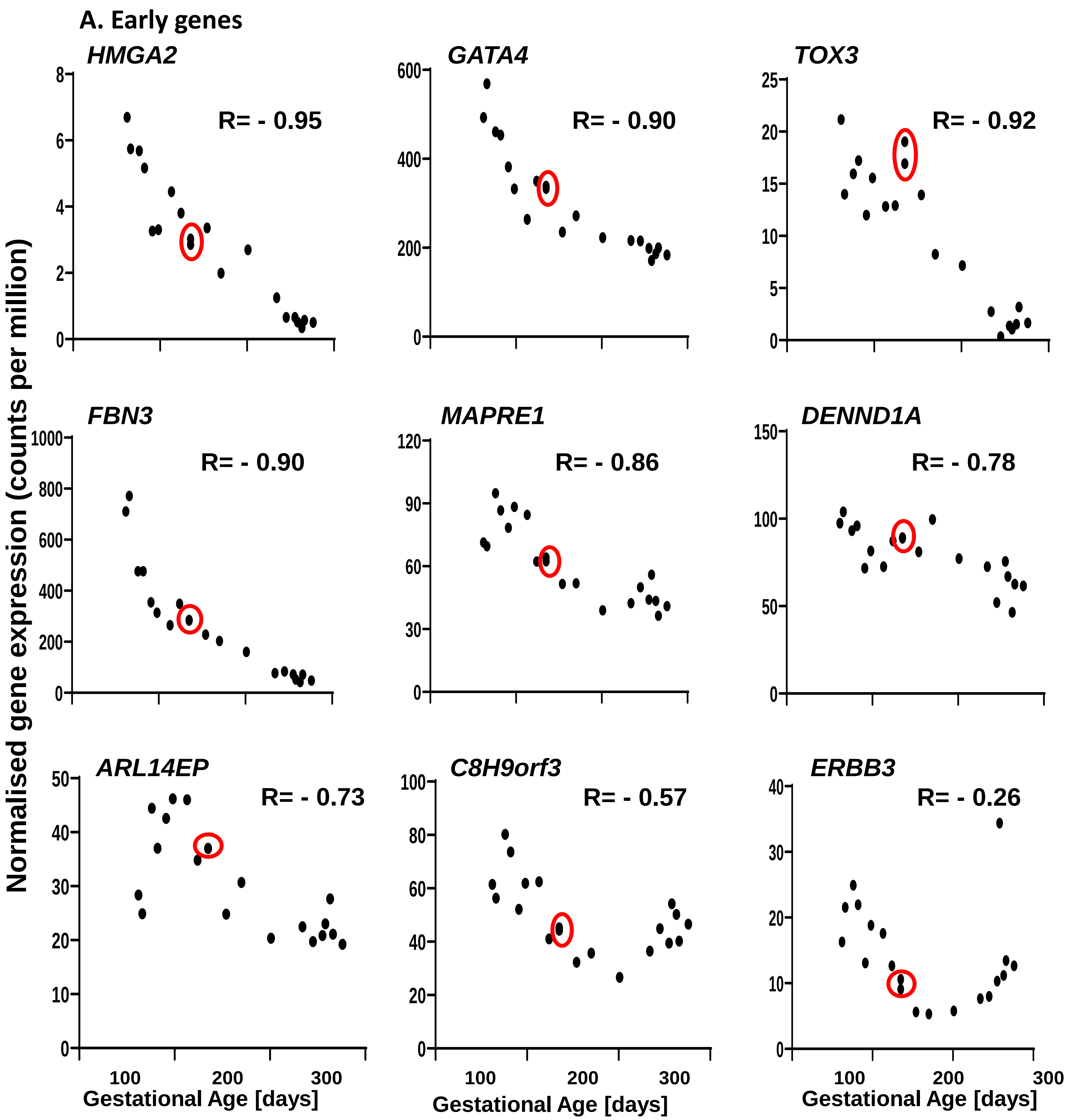

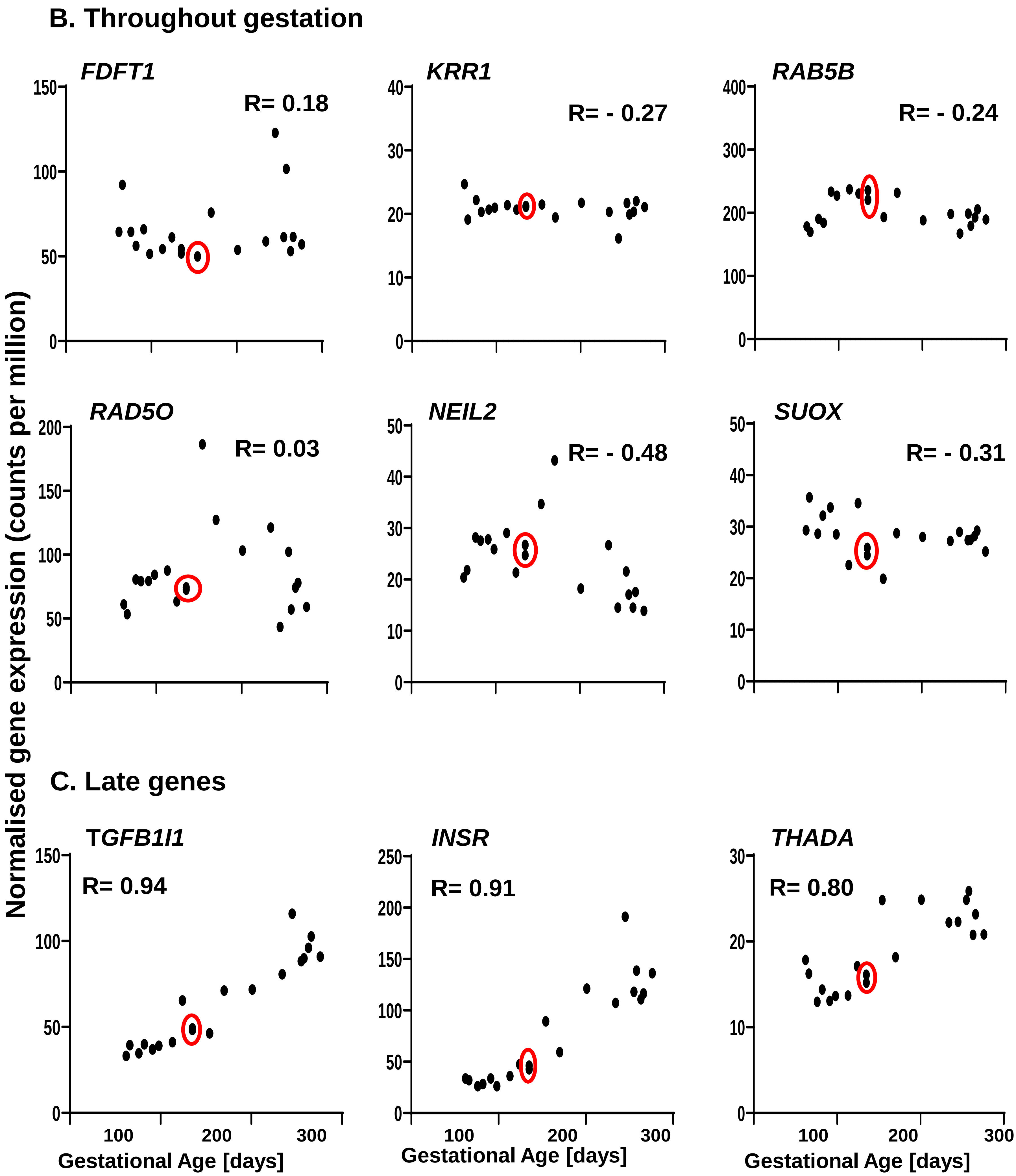
B. Throughout gestation
FDFT1
R= 0.18
KRR1
R= - 0.27
RAB5B
R= - 0.24
RAD5O
R= 0.03
NEIL2
R= - 0.48
SUOX
R= - 0.31
C. Late genes
TGFB1I1
R= 0.94
INSR
R= 0.91
THADA
R= 0.80
Normalised gene expression (counts per million)
Gestational Age [days]
Gestational Age [days]
Gestational Age [days]
100
200
300
0
50
100
150
0
10
20
30
40
0
100
200
300
400
0
50
100
150
200
250

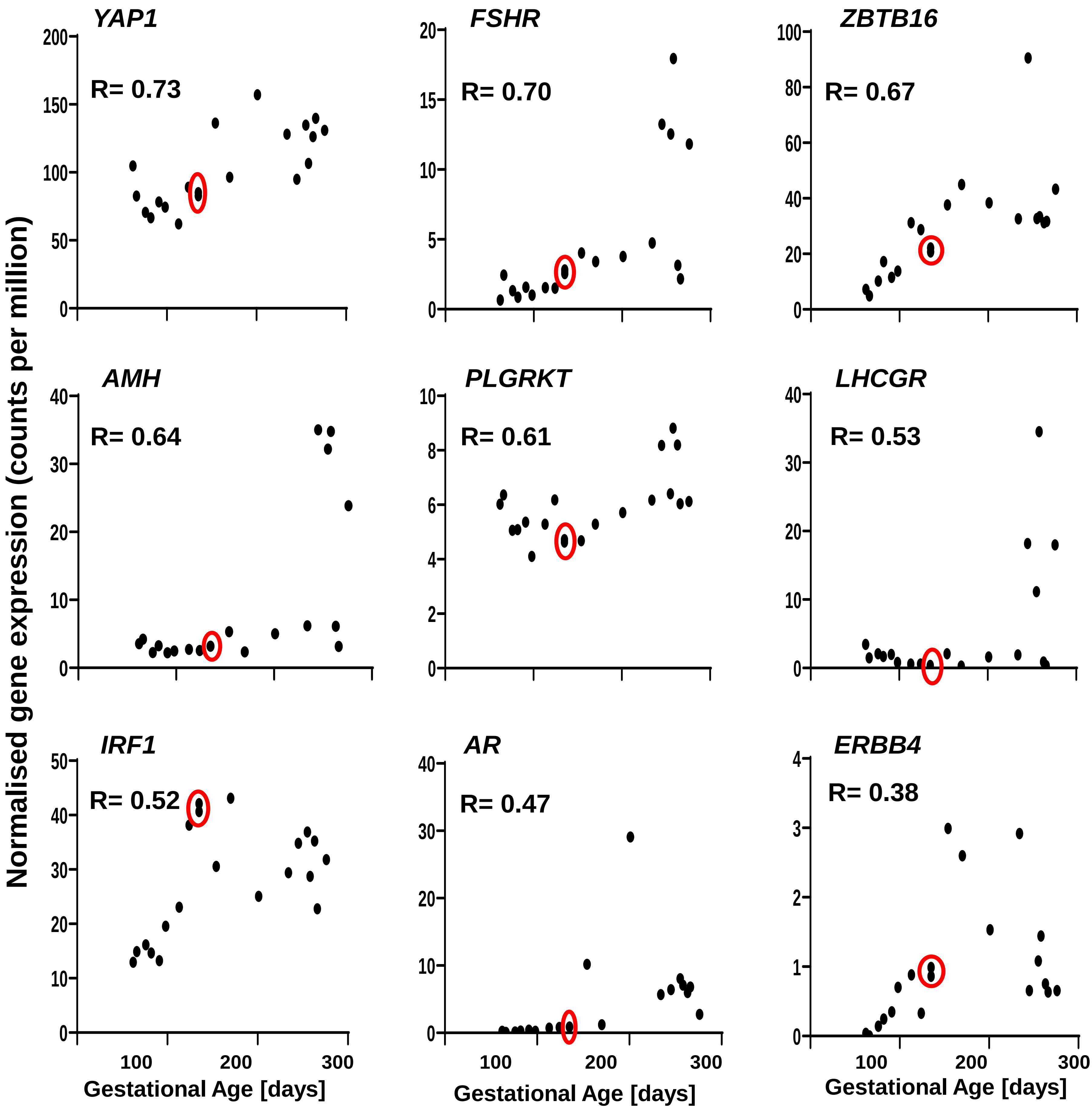
Normalised gene expression (counts per million)
YAP1
R= 0.73
FSHR
R= 0.70
ZBTB16
R= 0.67
AMH
R= 0.64
PLGRKT
R= 0.61
LHCGR
R= 0.53
IRF1
R= 0.52
AR
R= 0.47
ERBB4
R= 0.38
Gestational Age [days]
Gestational Age [days]
Gestational Age [days]

S2 Fig. Selected networks from cluster 1 and their respective functions and diseases. Red represents downregulation during the second half of gestation. The strength of regulation is shown by the intensity of each color.

Network 11 'DNA Replication, Recombination, and Repair, Nucleic Acid Metabolism, Small Molecule Biochemistry'

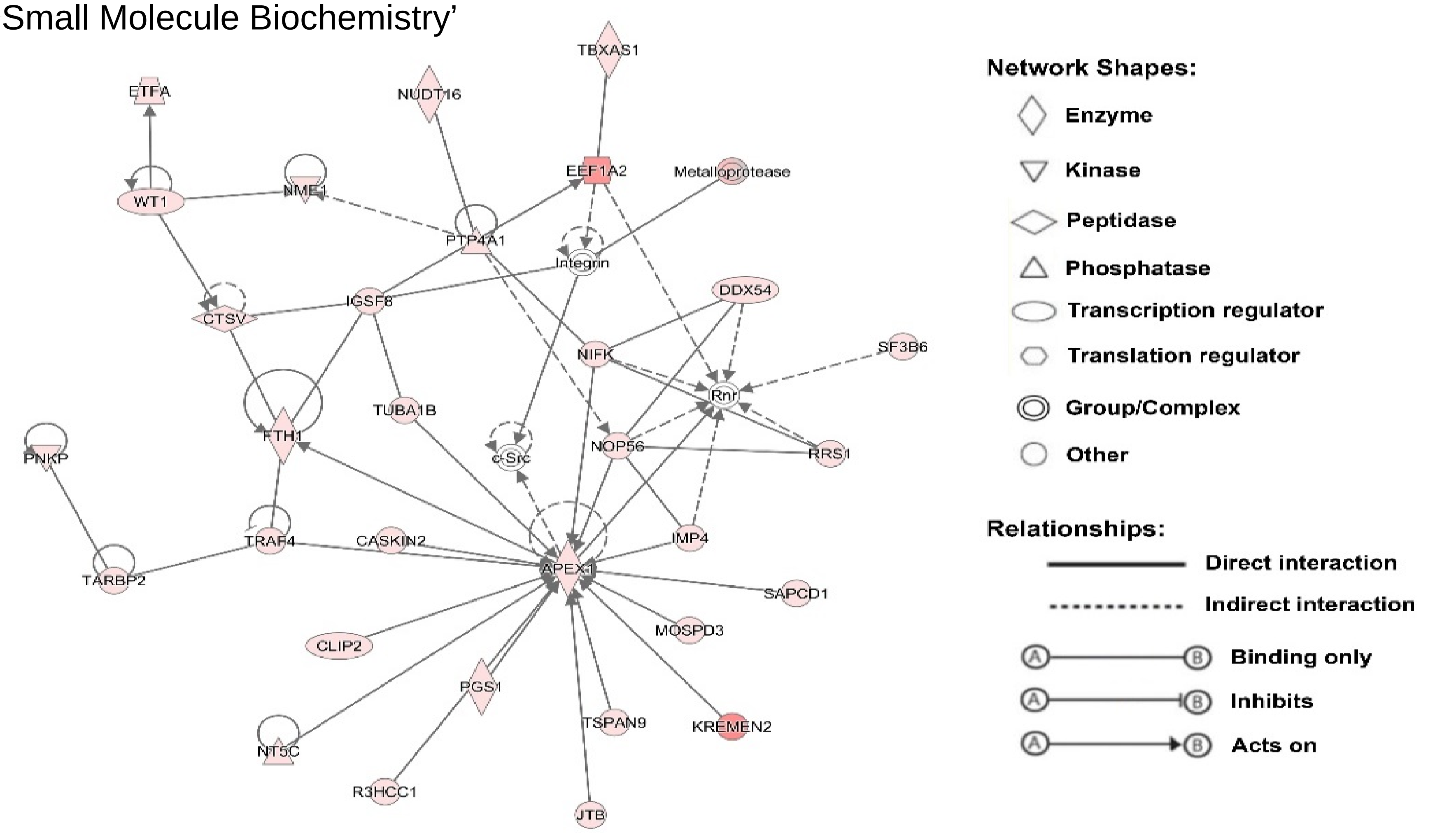

Network 14 'Cardiovascular Disease, Cellular Assembly and Organization, Cellular Function and Maintenance'

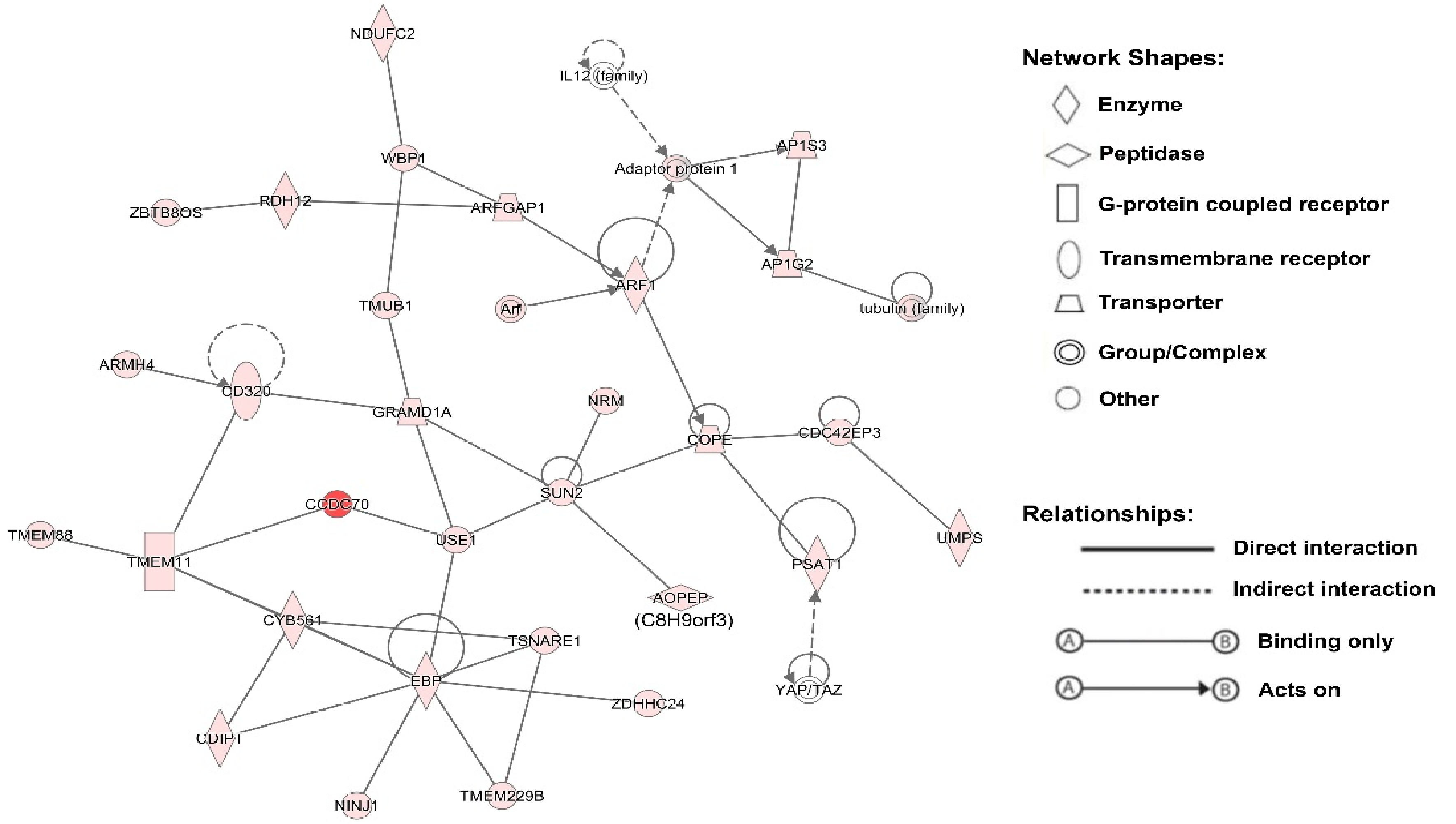

Network 18 ‘Endocrine System Disorders, Gene Expression, Hereditary Disorder’

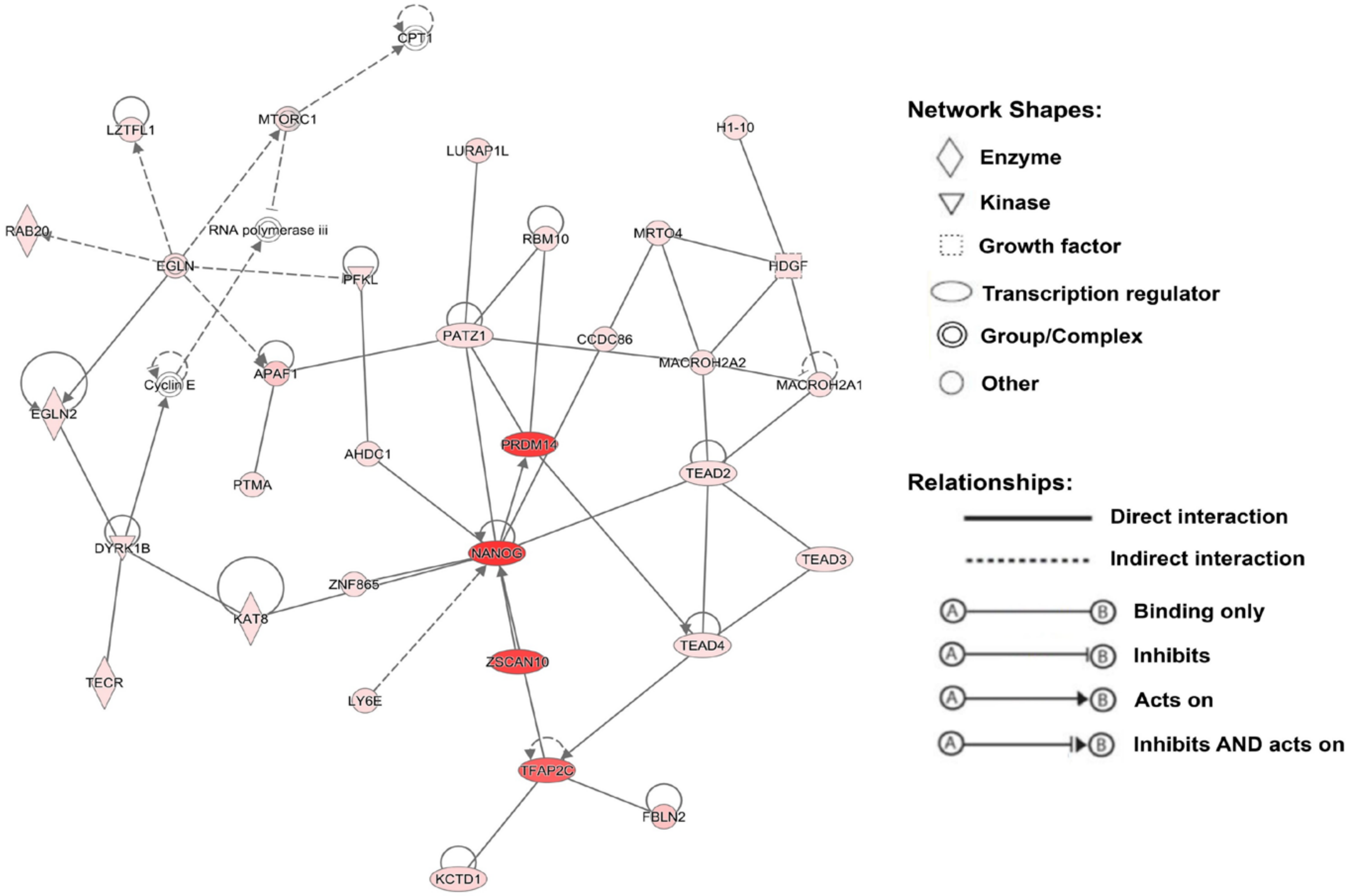

S3 Fig: Selected networks from cluster 2 and their respective functions and diseases. Red represents downregulation and blue represents upregulation during the second half of gestation. The strength of regulation is shown by the intensity of each color.

Network 1 ‘Cancer, Gastrointestinal Disease, Hepatic System Disease’

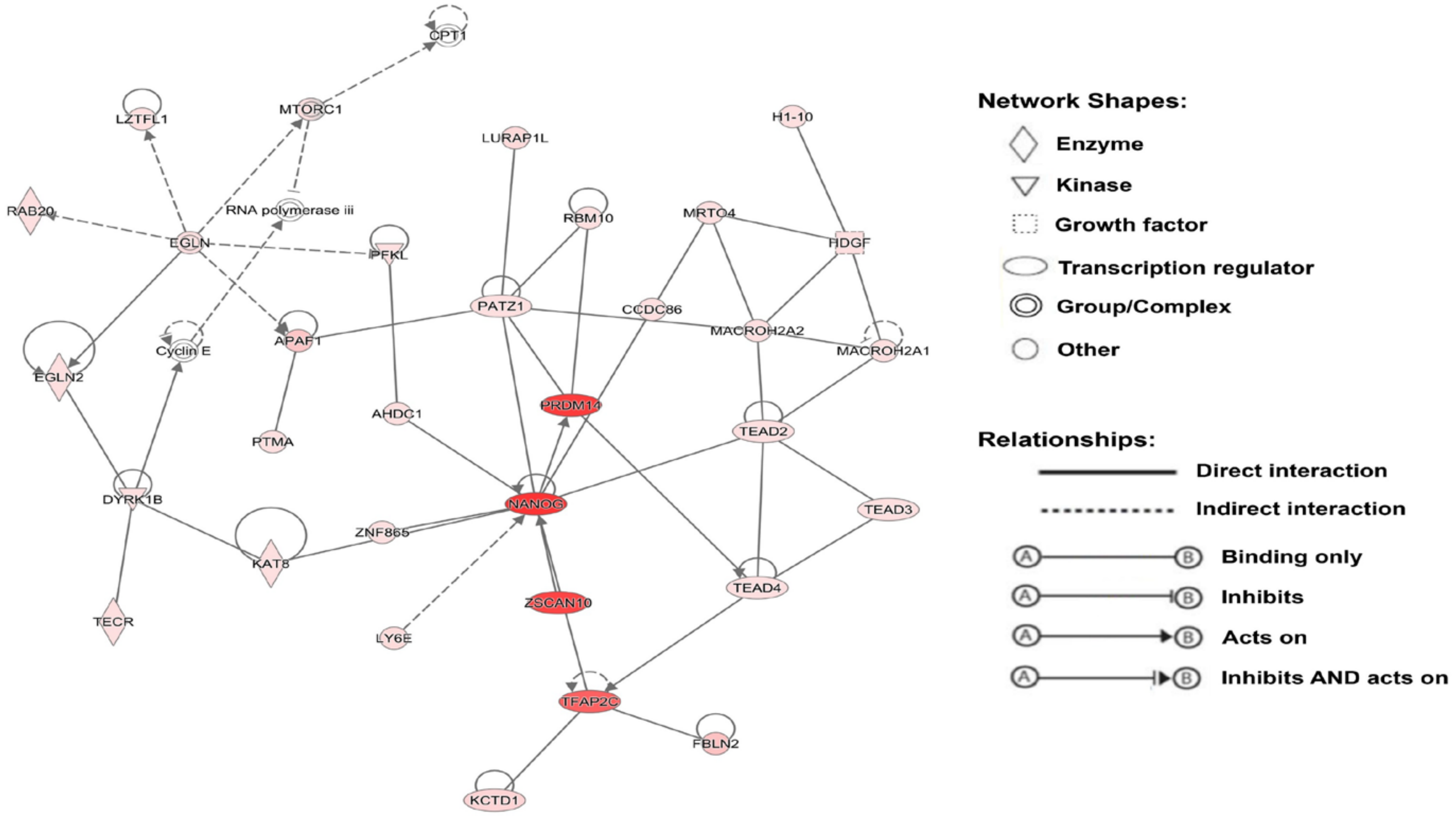

Network 4 ‘Carbohydrate Metabolism, Nucleic Acid Metabolism, Small Molecule Biochemistry’

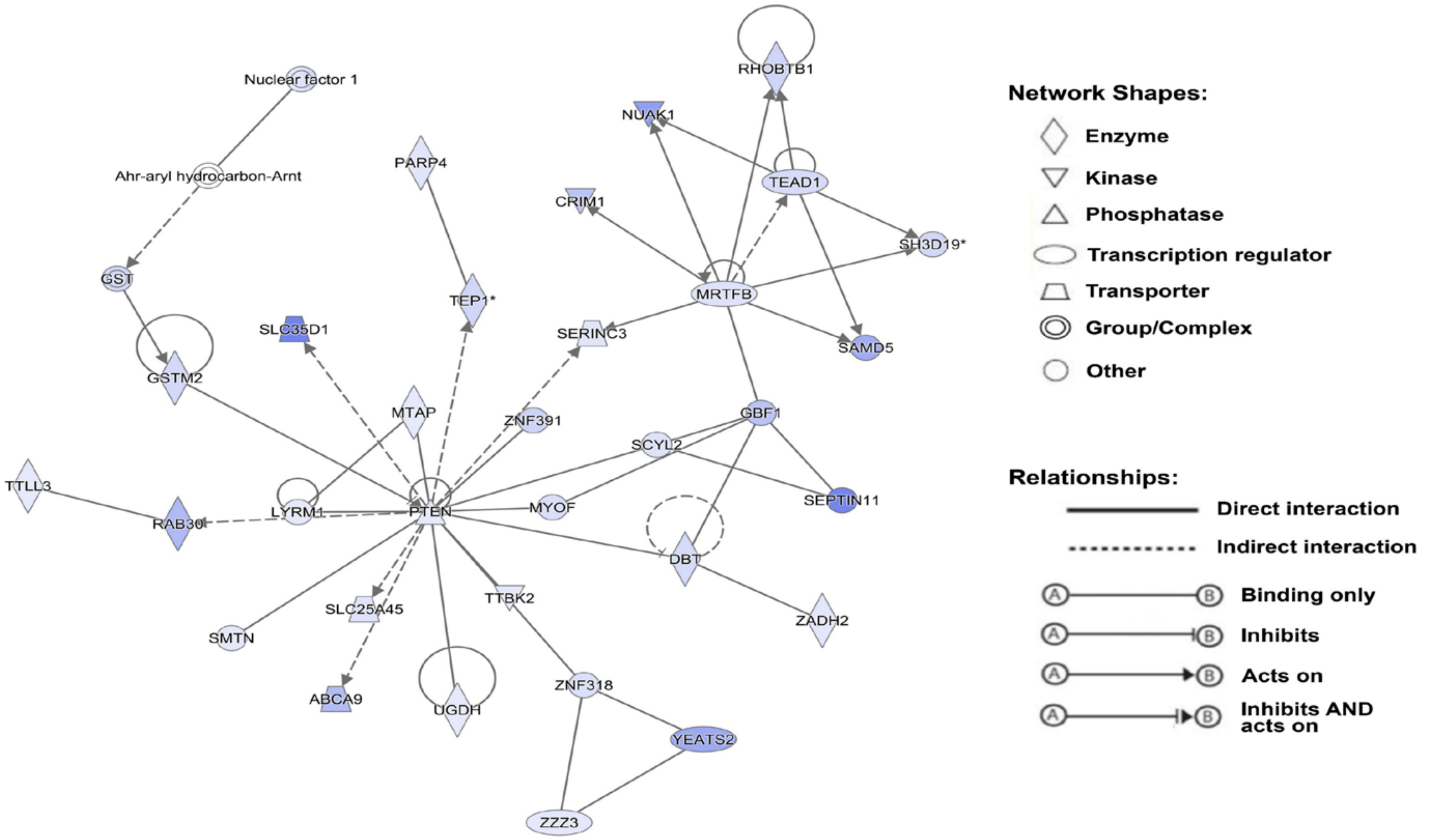

Network 8 ‘Embryonic Development, Nervous System Development and Function, Organ Development’

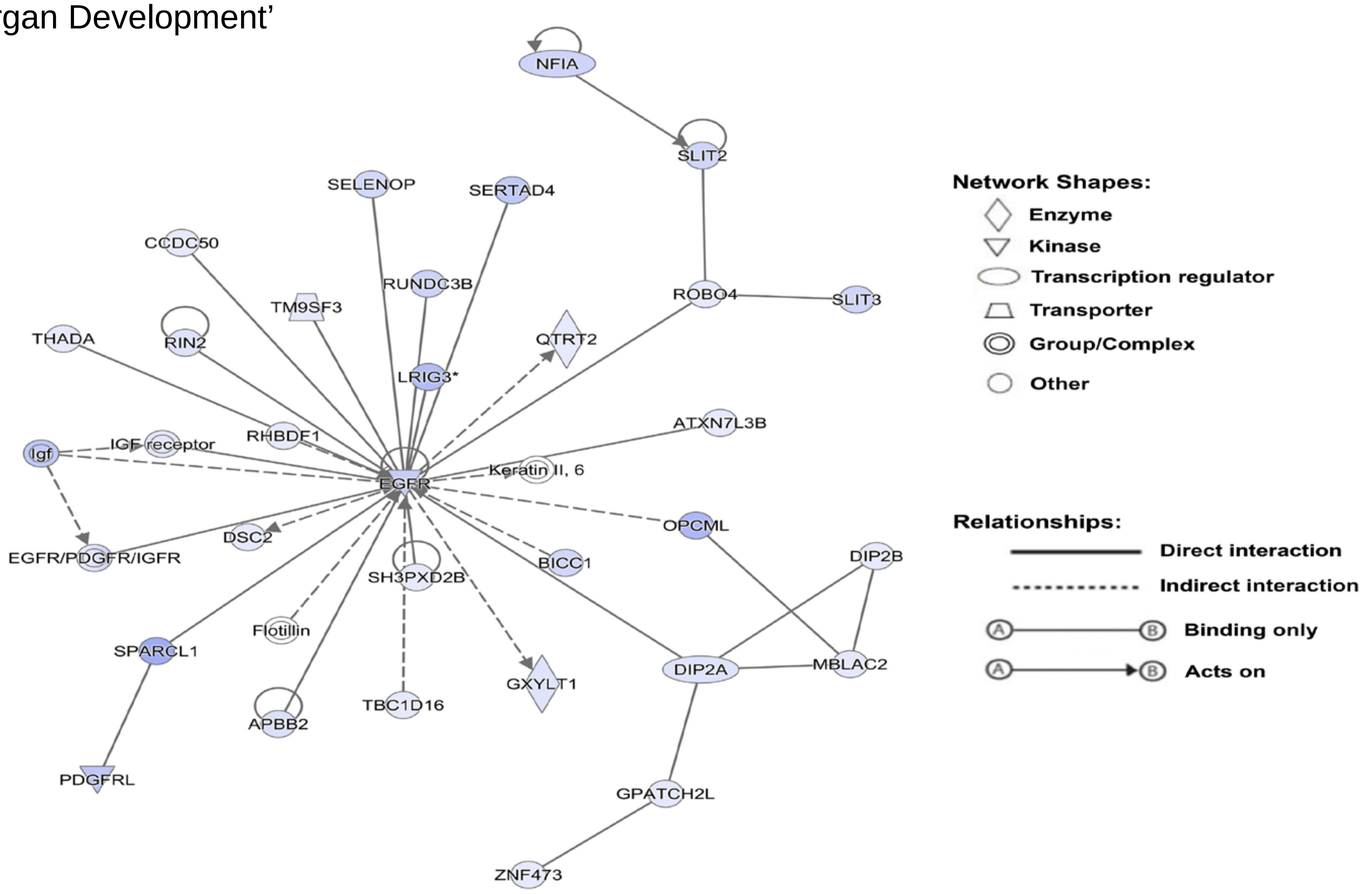

Network 10 ‘Cellular Development, Gene Expression, Tissue Development’

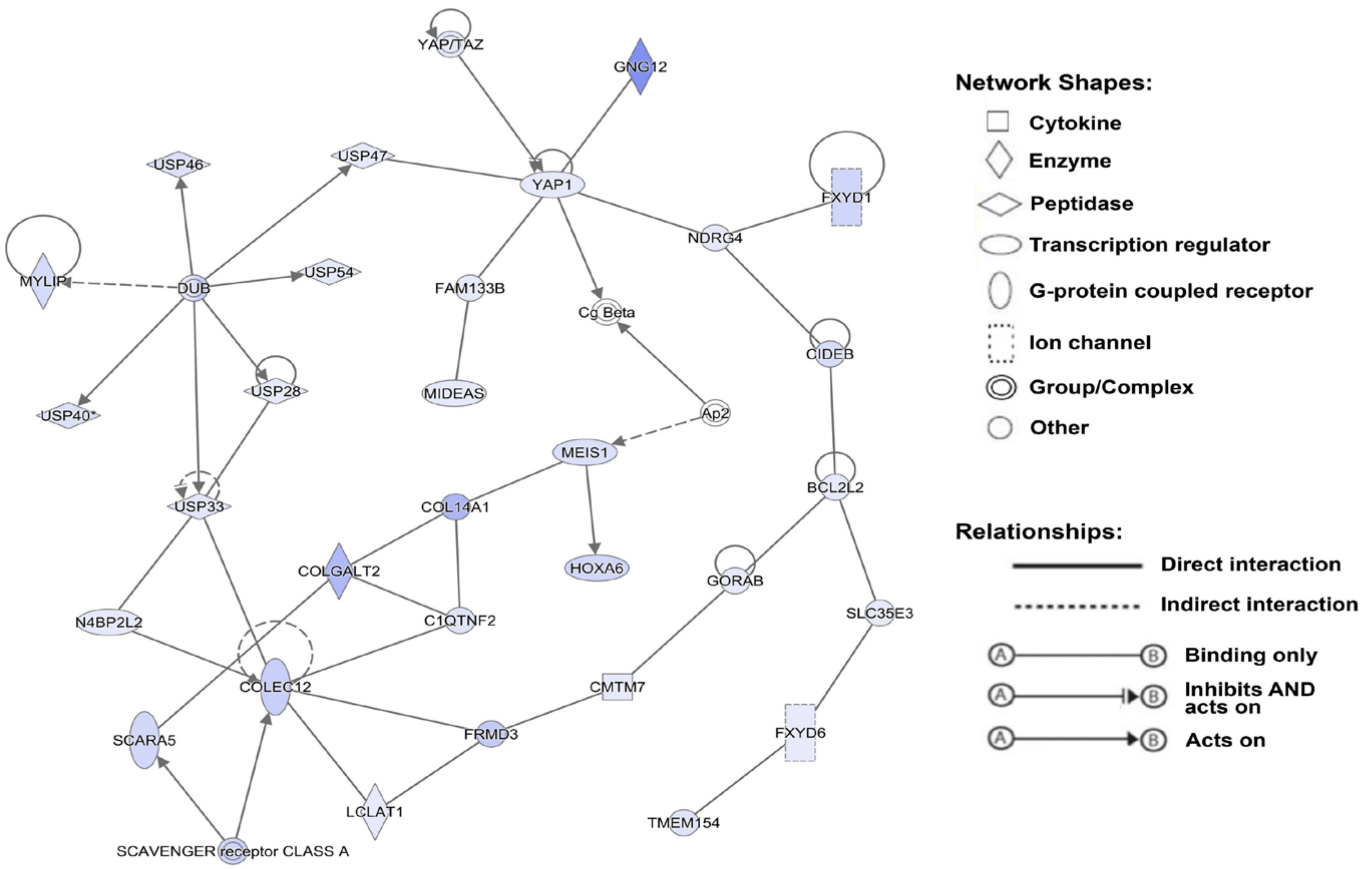

Network 14 ‘Cell Signalling, Cellular Assembly and Organization, Cellular Function and Maintenance’

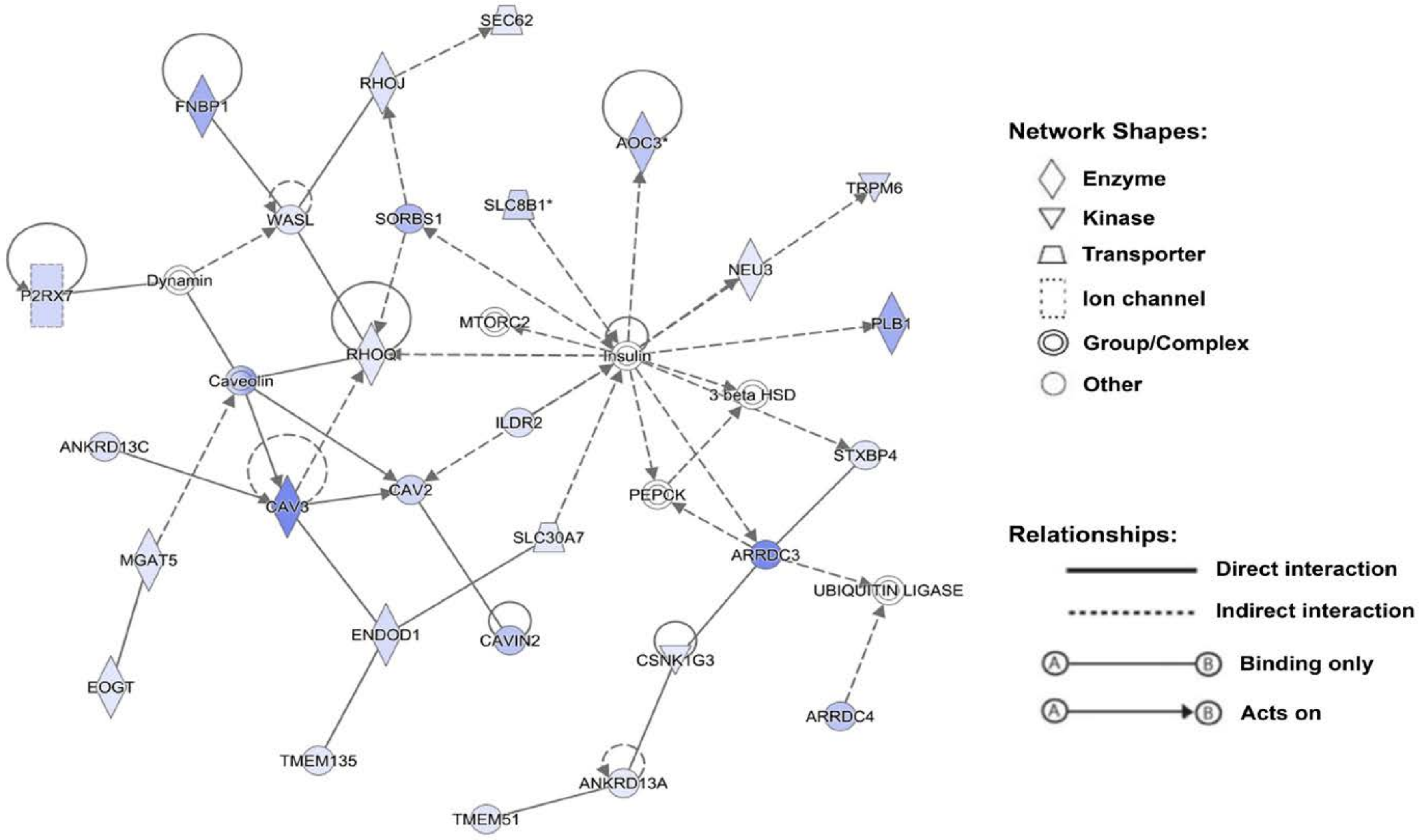

S4 Fig 4. Selected networks from cluster 3 and their respective functions and diseases. Red represents downregulation and blue represents upregulation during the second half of gestation. The strength of regulation is shown by the intensity of each color.

Network 3 ‘Cell-To-Cell Signaling and Interaction, Cellular Assembly and Organization, Cellular Function and Maintenance’

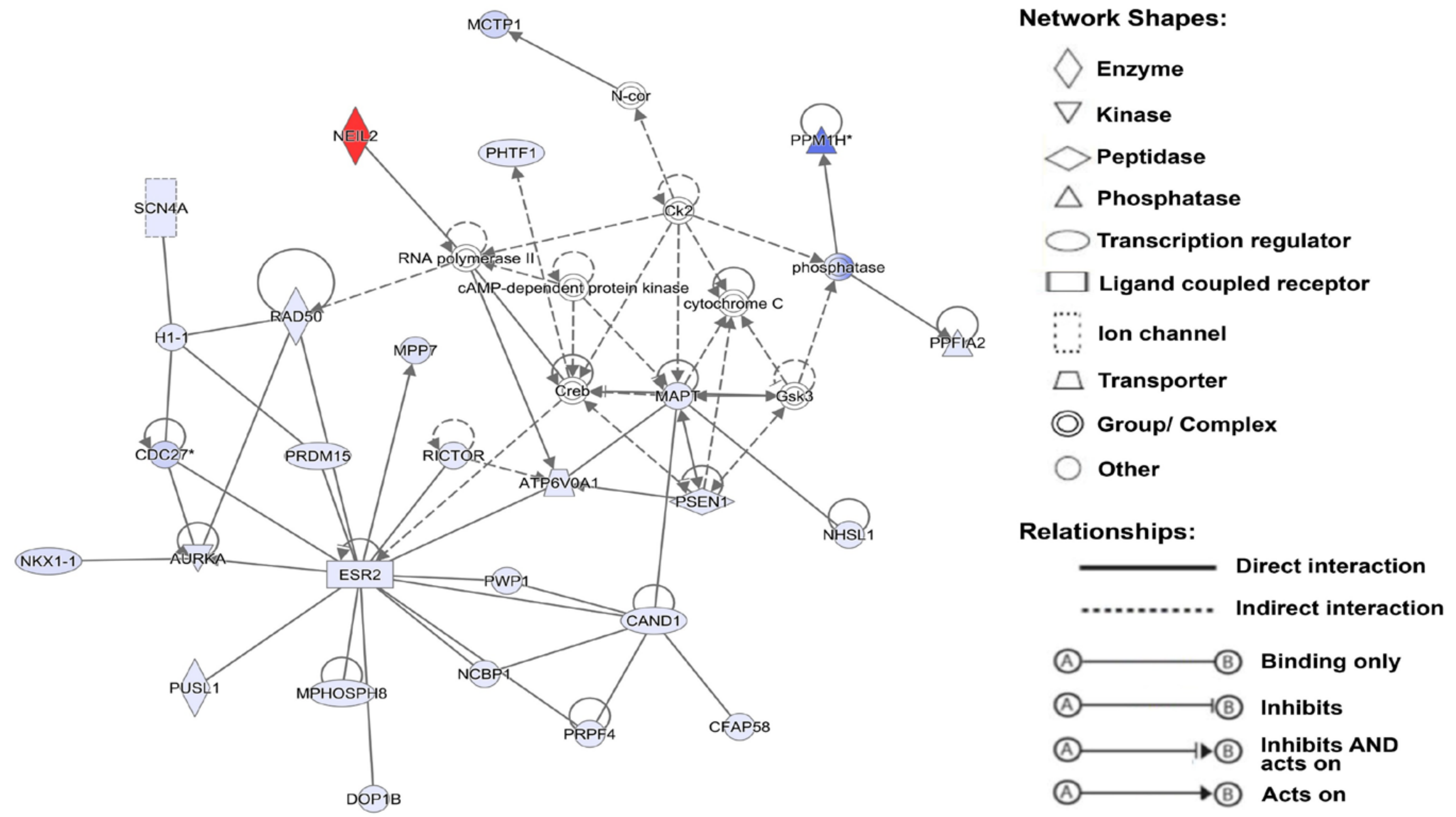

Network 5 ‘Lipid Metabolism, Molecular Transport, Small Molecule Biochemistry’

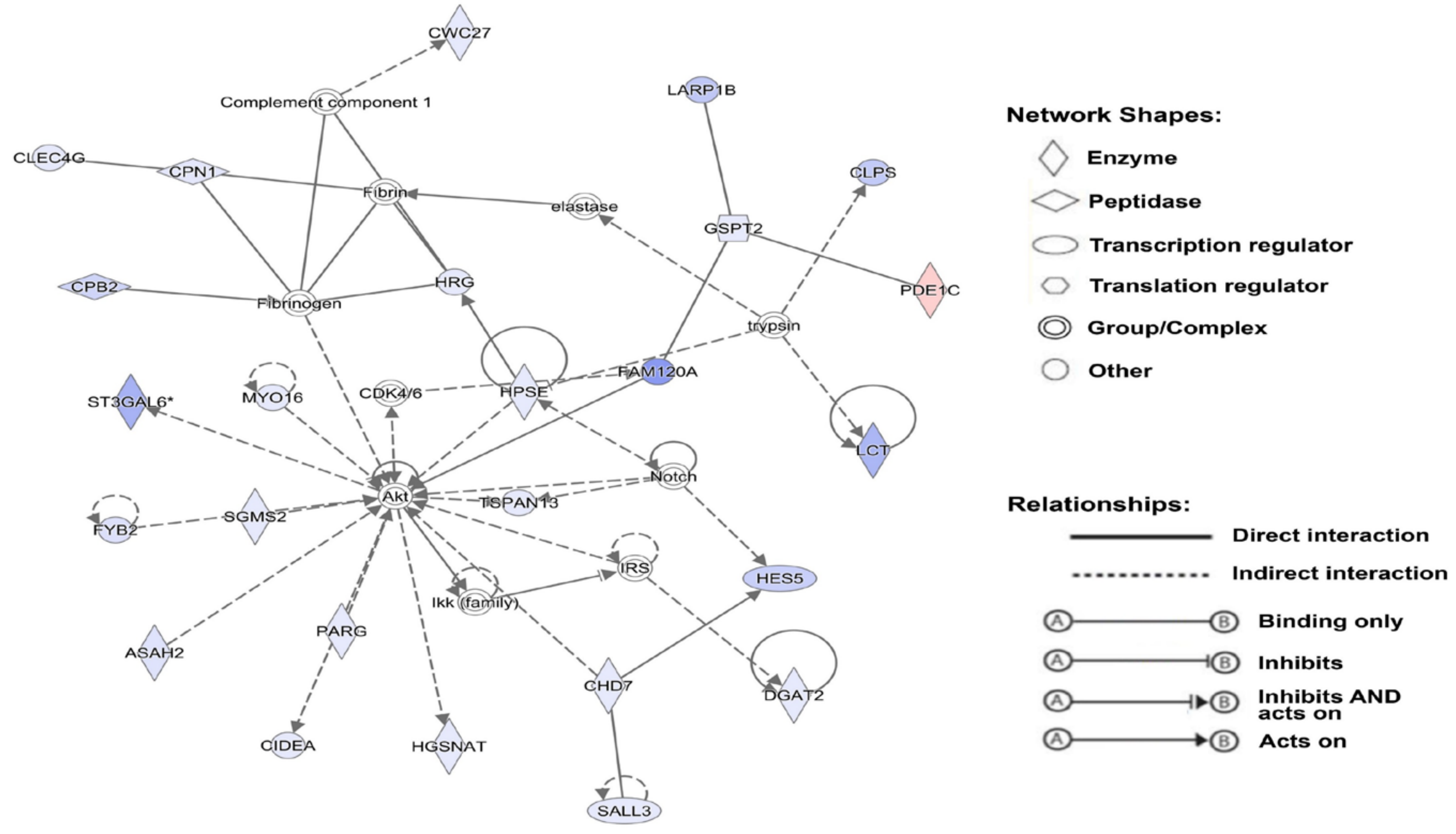

Network 6 ‘Cancer, Gastrointestinal Disease, Post-Translational Modification’

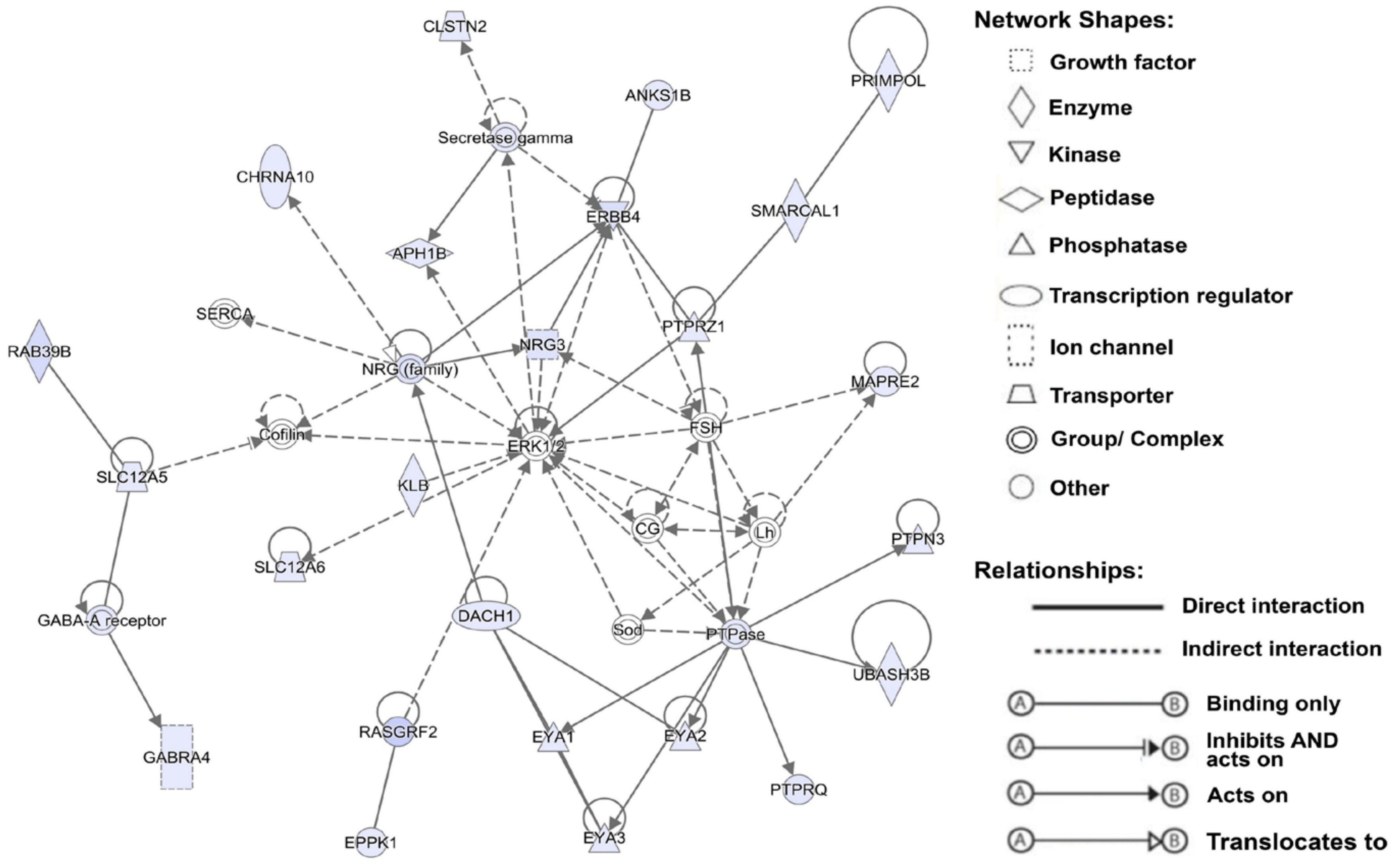

Network 20 ‘Nervous System Development and Function, Neurological Disease, Organ Morphology’

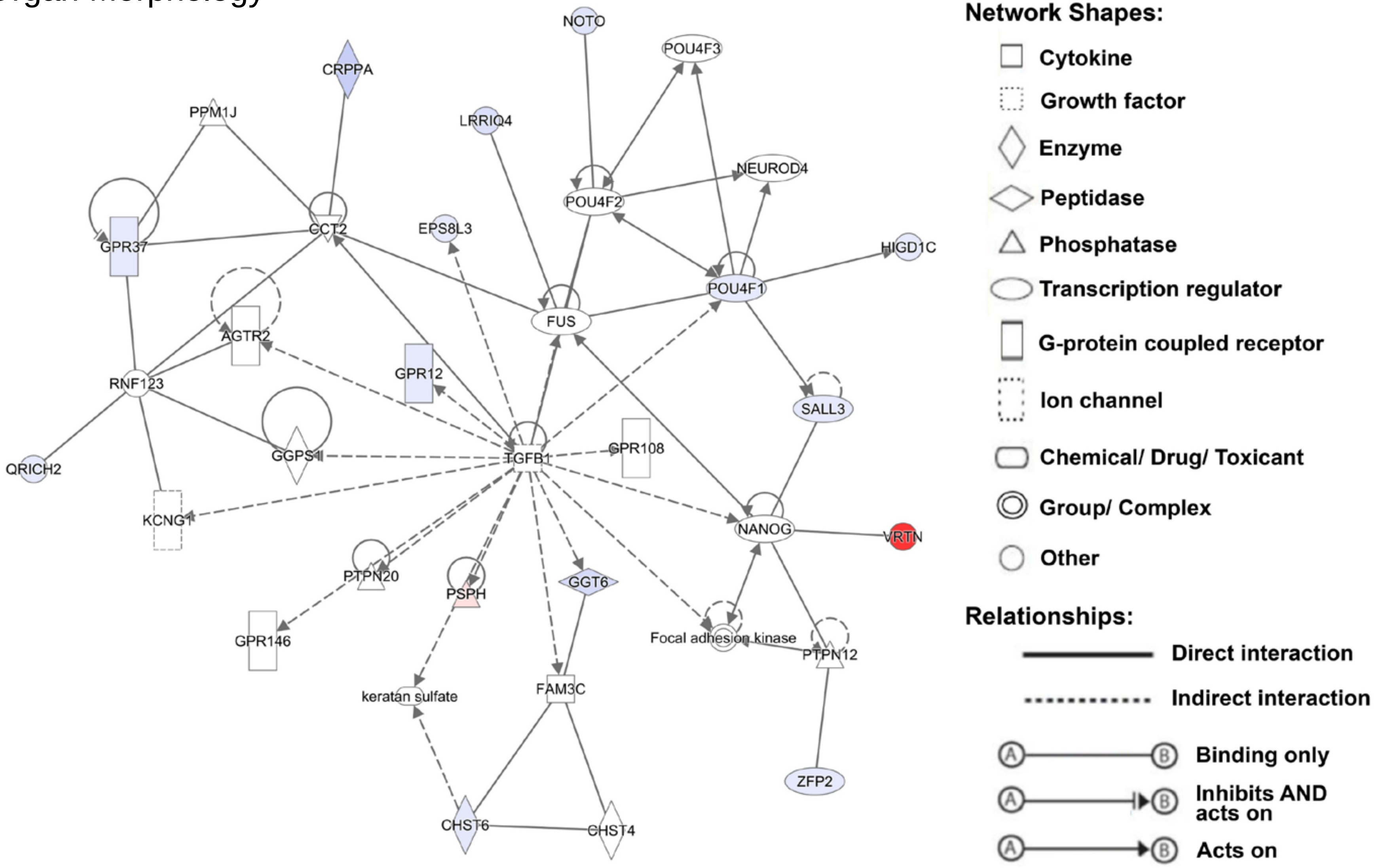

Network 21 ‘Cell Signaling, Molecular Transport, Vitamin and Mineral Metabolism’

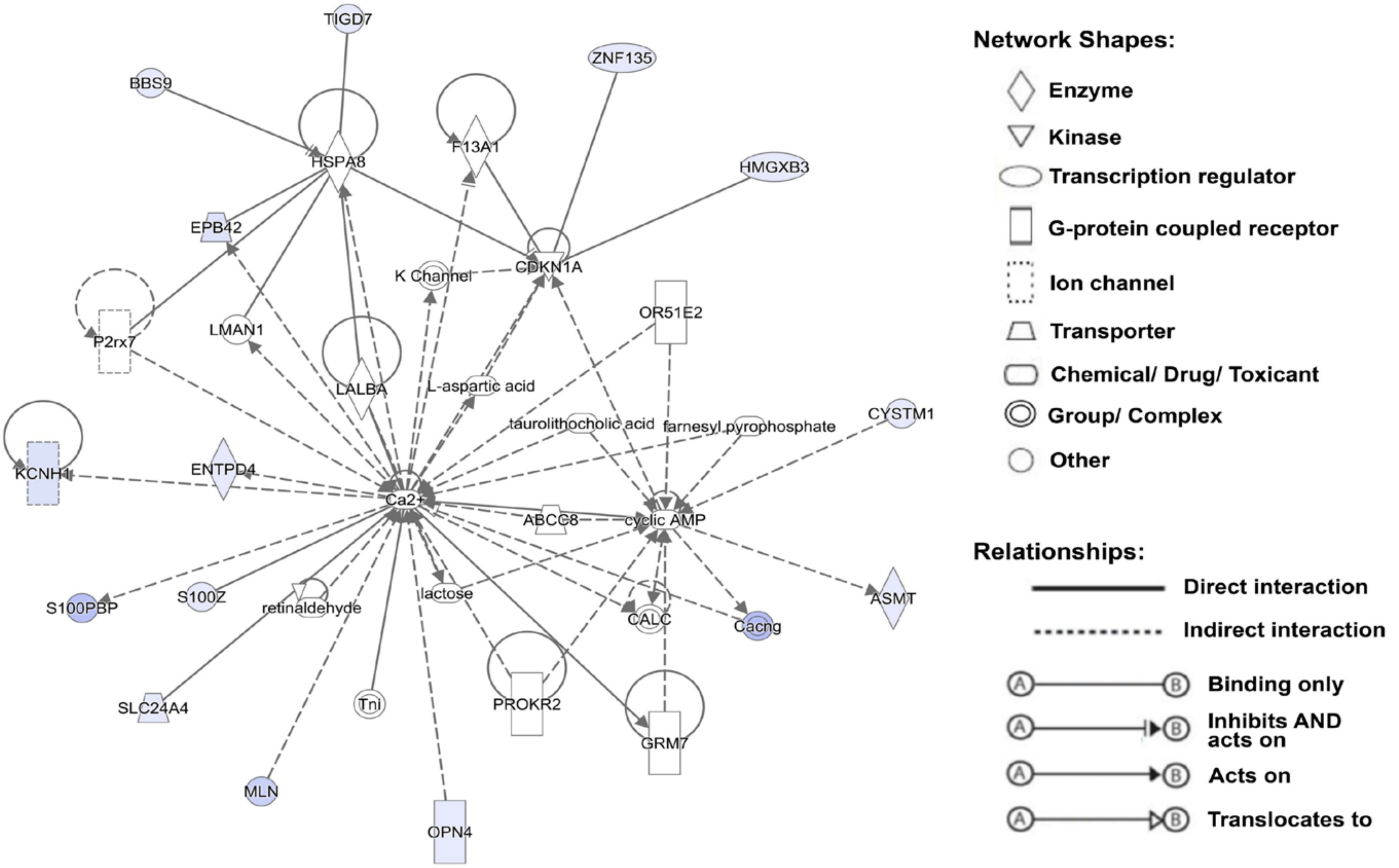

Network 22 ‘Amino Acid Metabolism, Molecular Transport, Small Molecule Biochemistry’

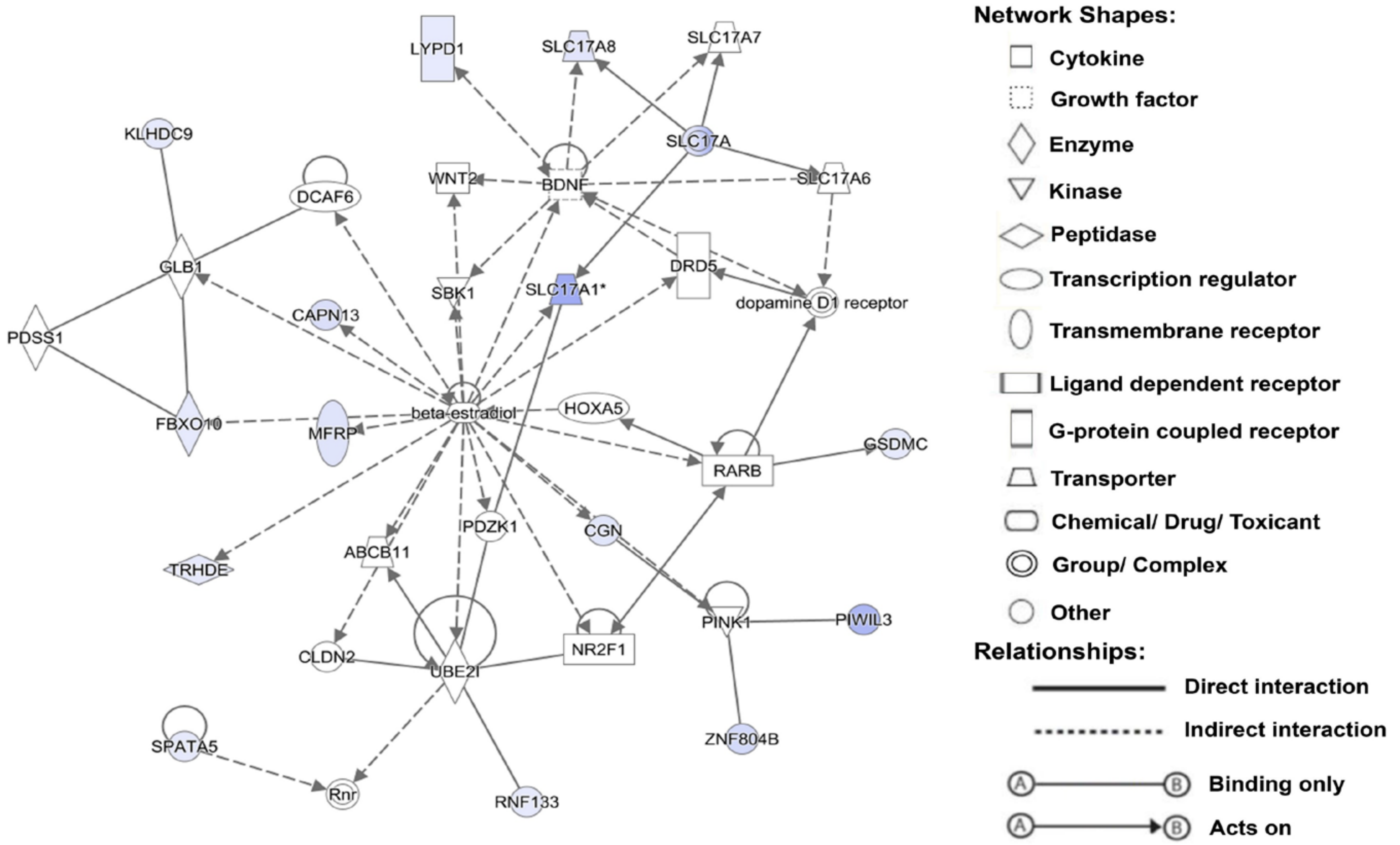

S5 Fig. Selected networks from cluster 4 and their respective functions and diseases. Blue represents upregulation during the second half of gestation. The strength of regulation is shown by the intensity of each color.

Network 4 'Lipid Metabolism, Small Molecule Biochemistry, Vitamin and Mineral Metabolism'

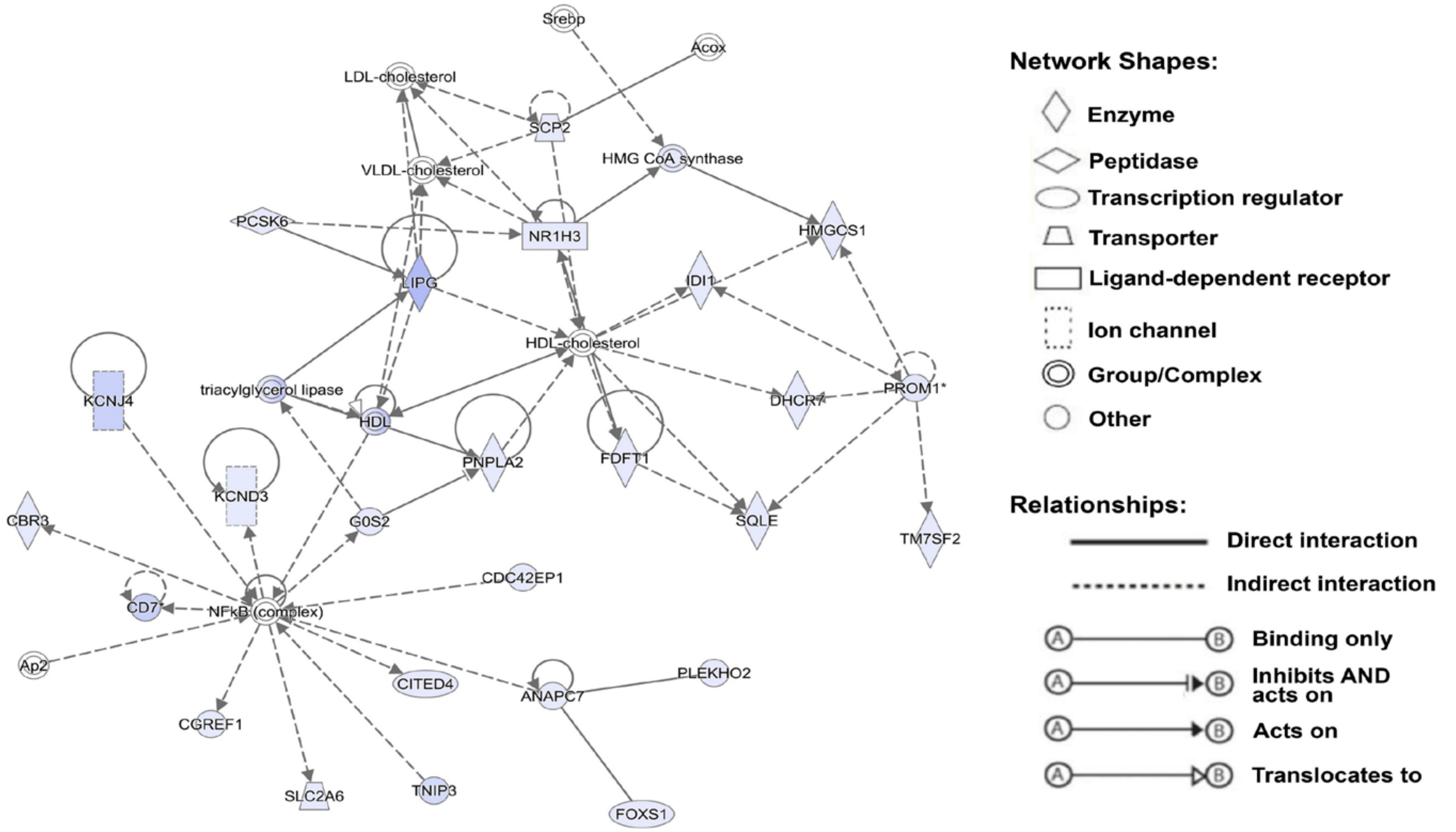

Network 9 'Carbohydrate Metabolism, Cell Cycle, Gene Expression'

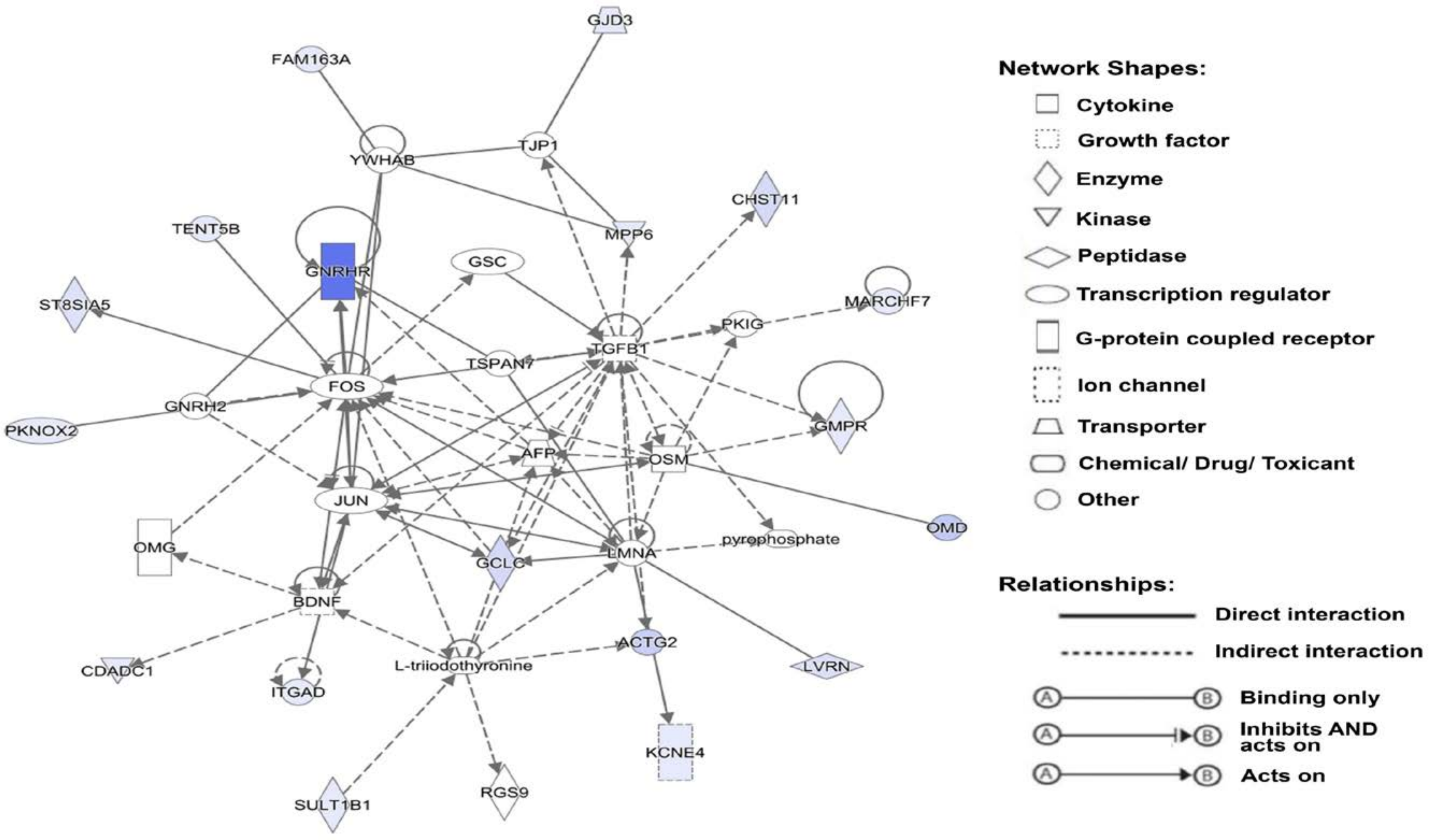

Network 12 ‘Cell Signaling, Connective Tissue Development and Function, Post-Translational Modification’

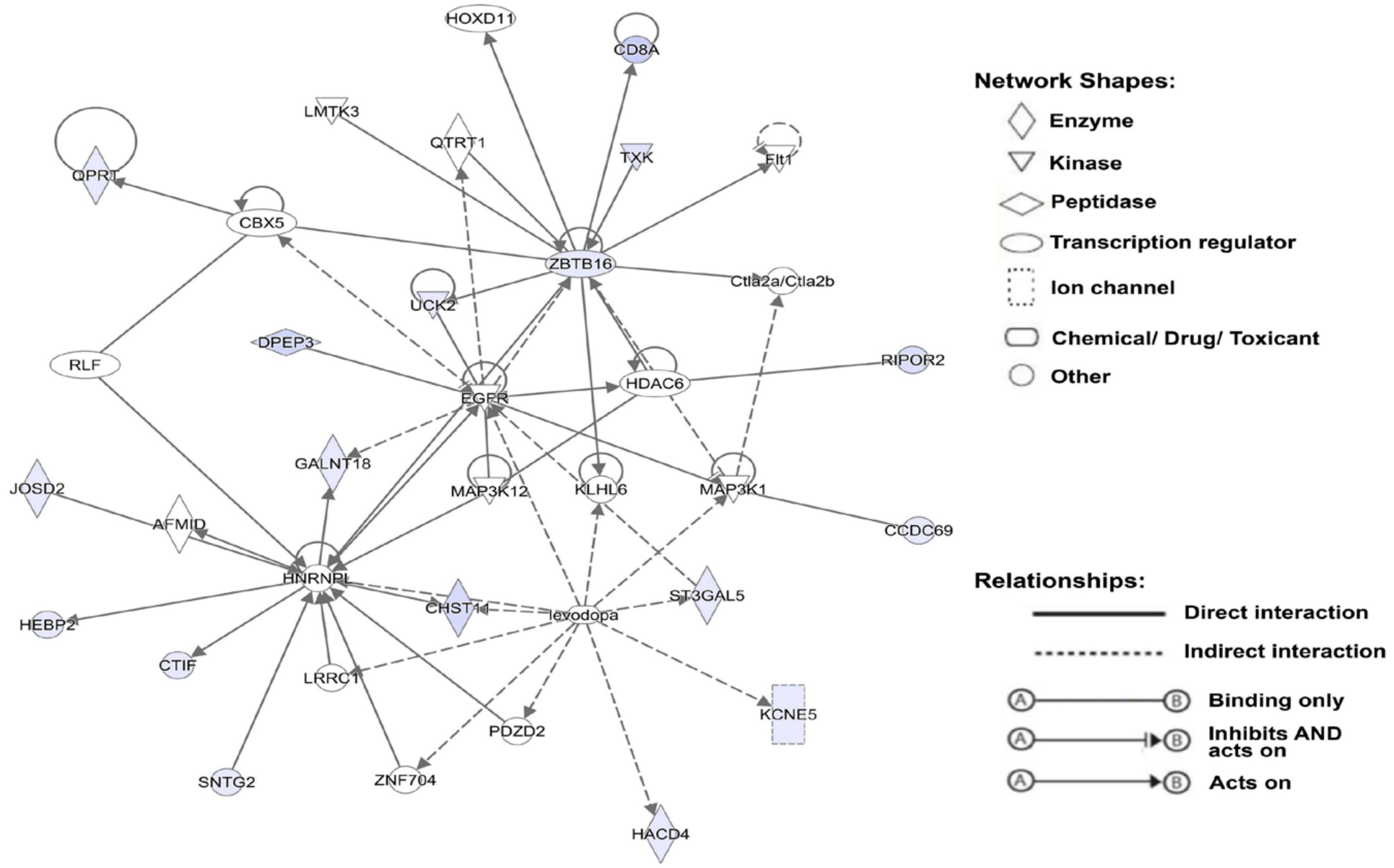

Network 14 ‘Lipid Metabolism, Molecular Transport, Small Molecule Biochemistry’

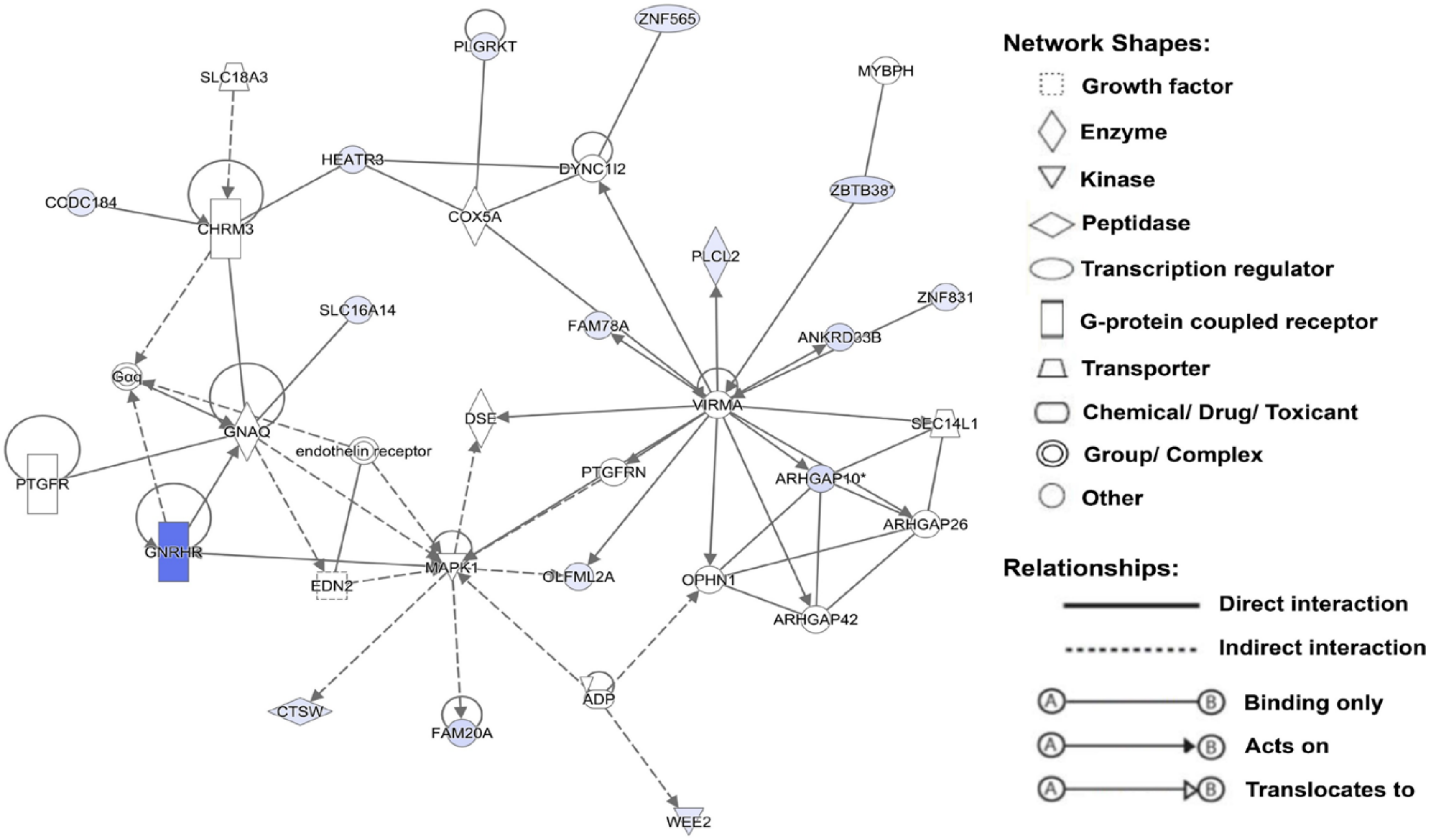

Network 16 ‘Cancer, Organismal Injury and Abnormalities, Tumor Morphology’

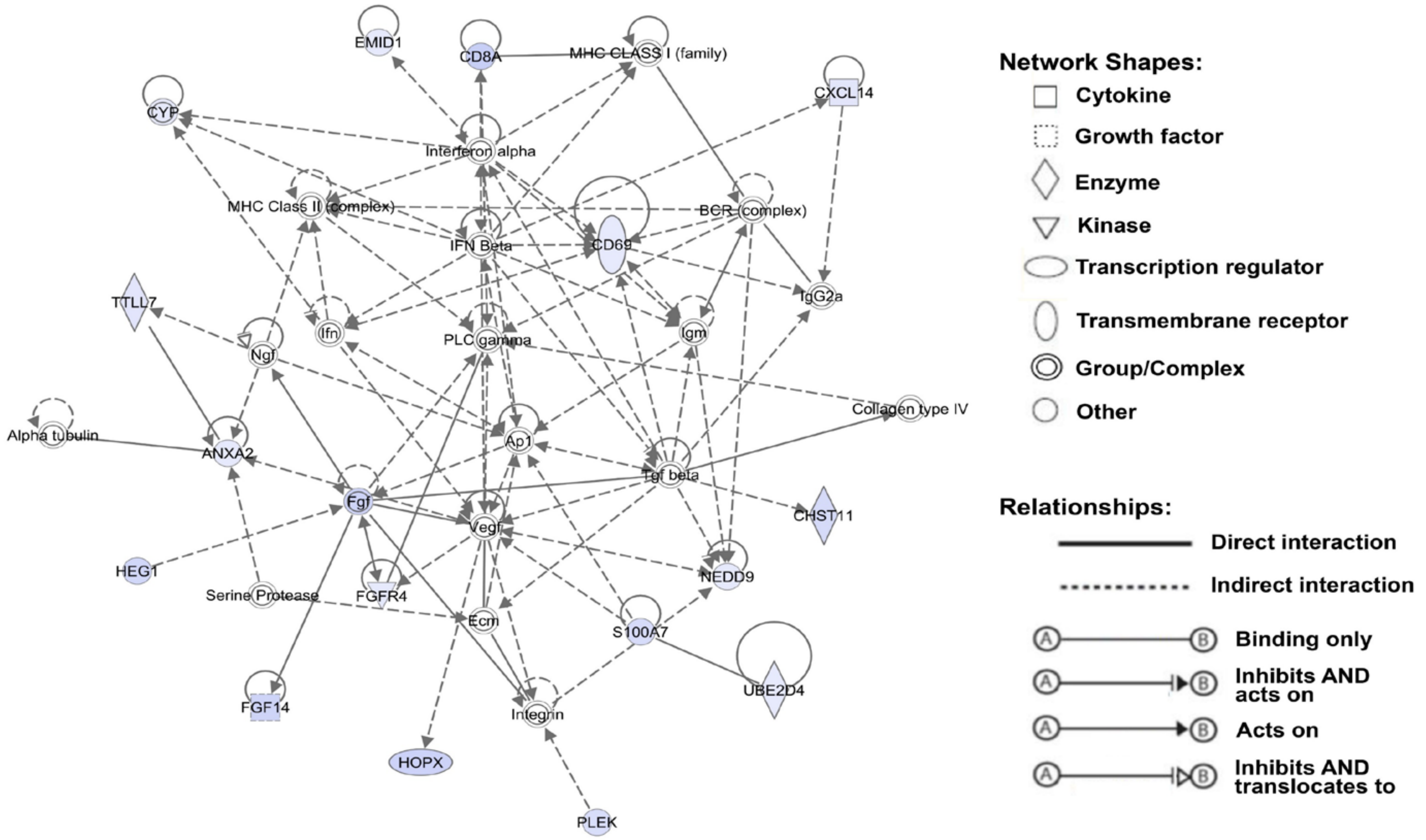

Network 20 ‘Embryonic Development, Nervous System Development and Function, Organ Development’

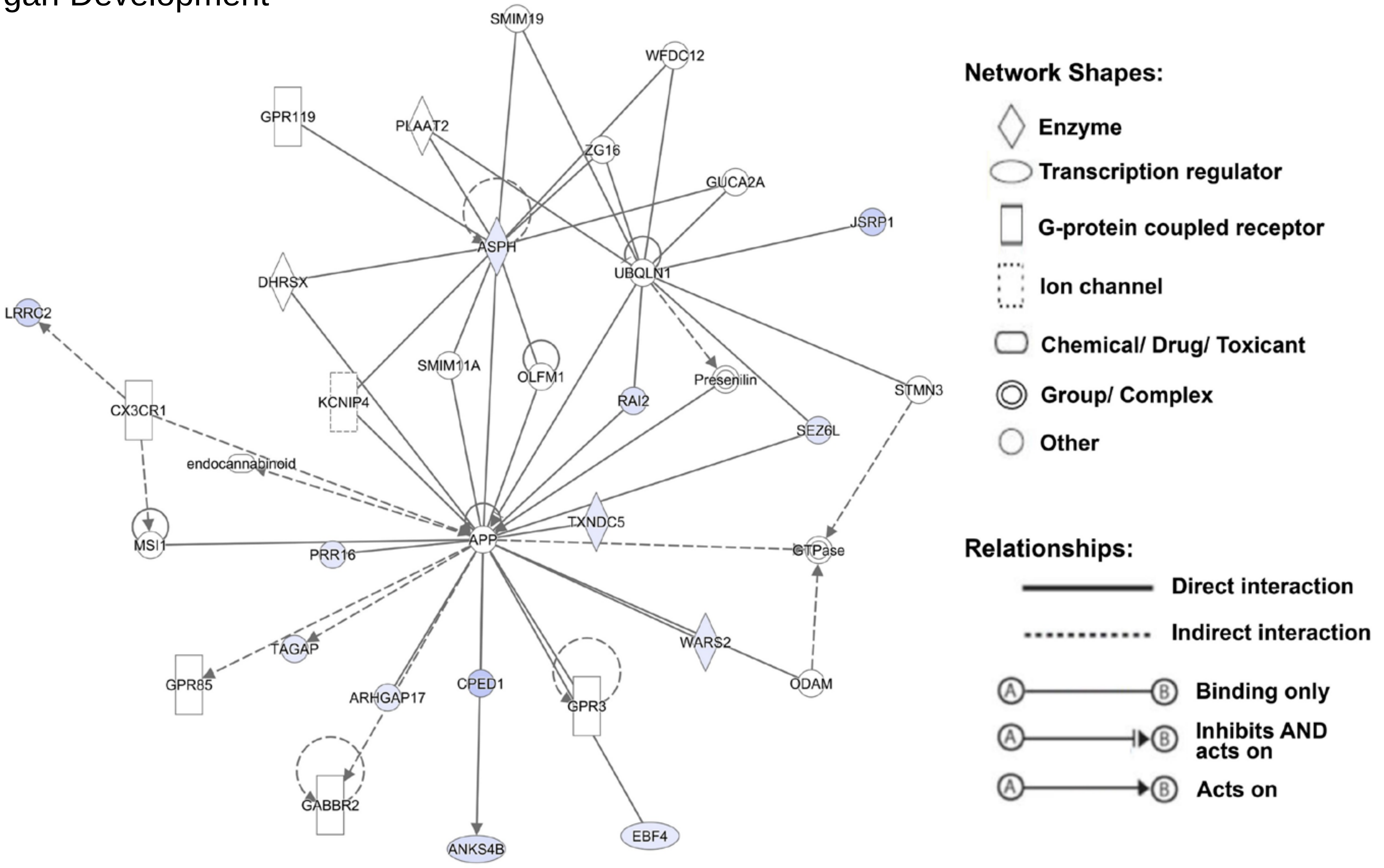

Network 21 'Endocrine System Disorders, Organismal Injury and Abnormalities, Reproductive System Disease'

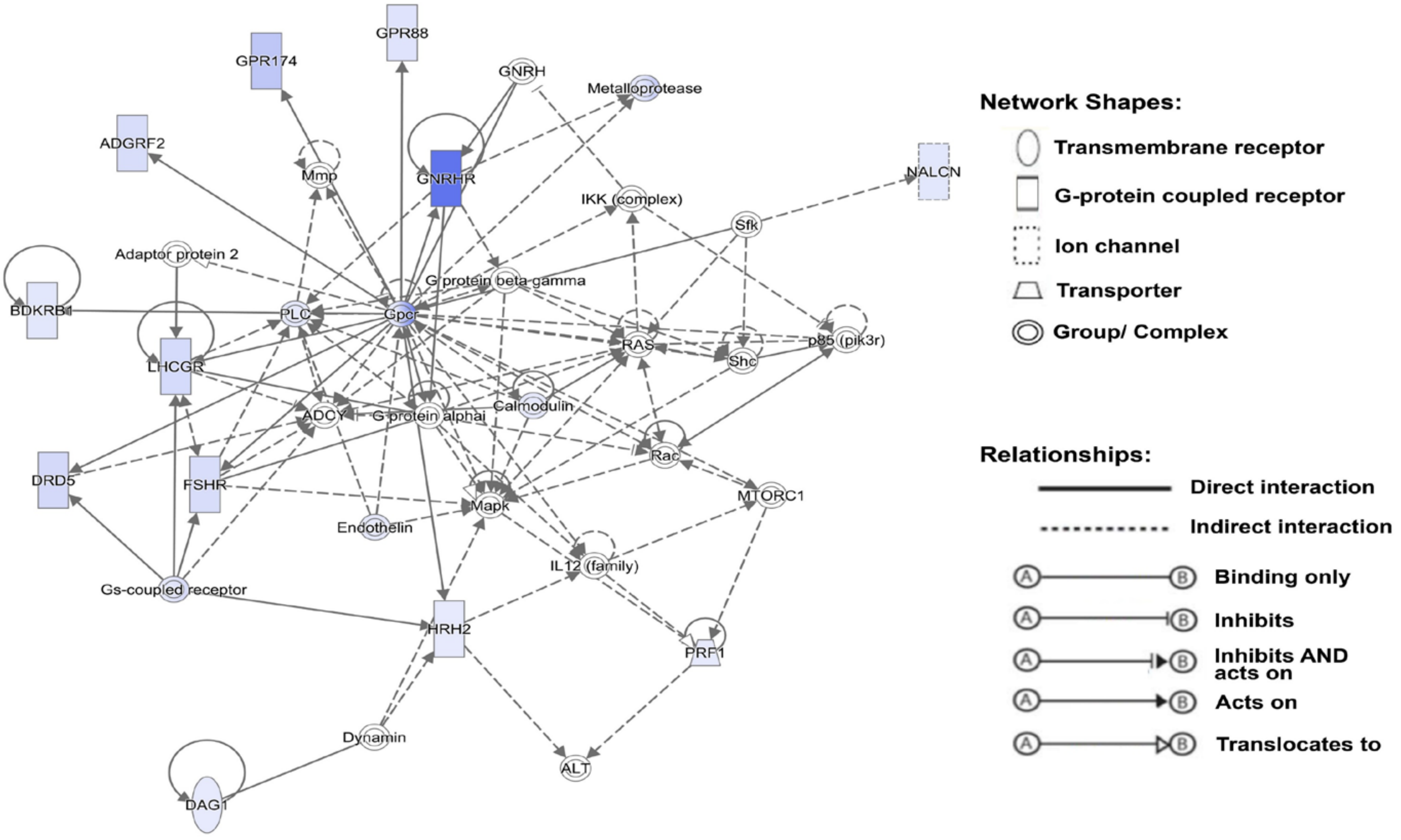

S6 Fig. Scatterplots showing the expression pattern of nuclear-encoded mitochondrial genes associated with the top canonical pathways 'oxidative phosphorylation' and 'mitochondrial dysfunction' identified in cluster 1.

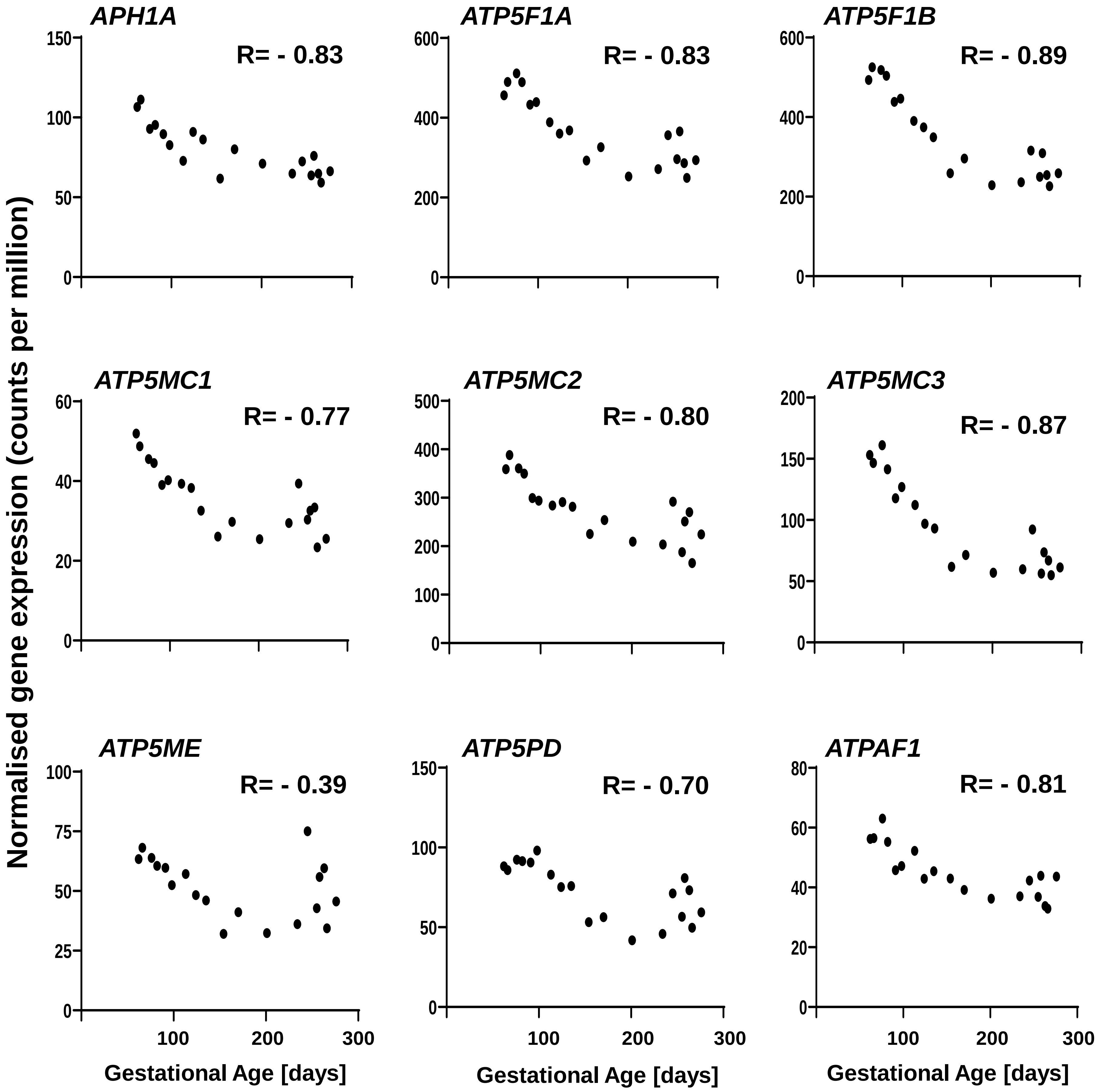

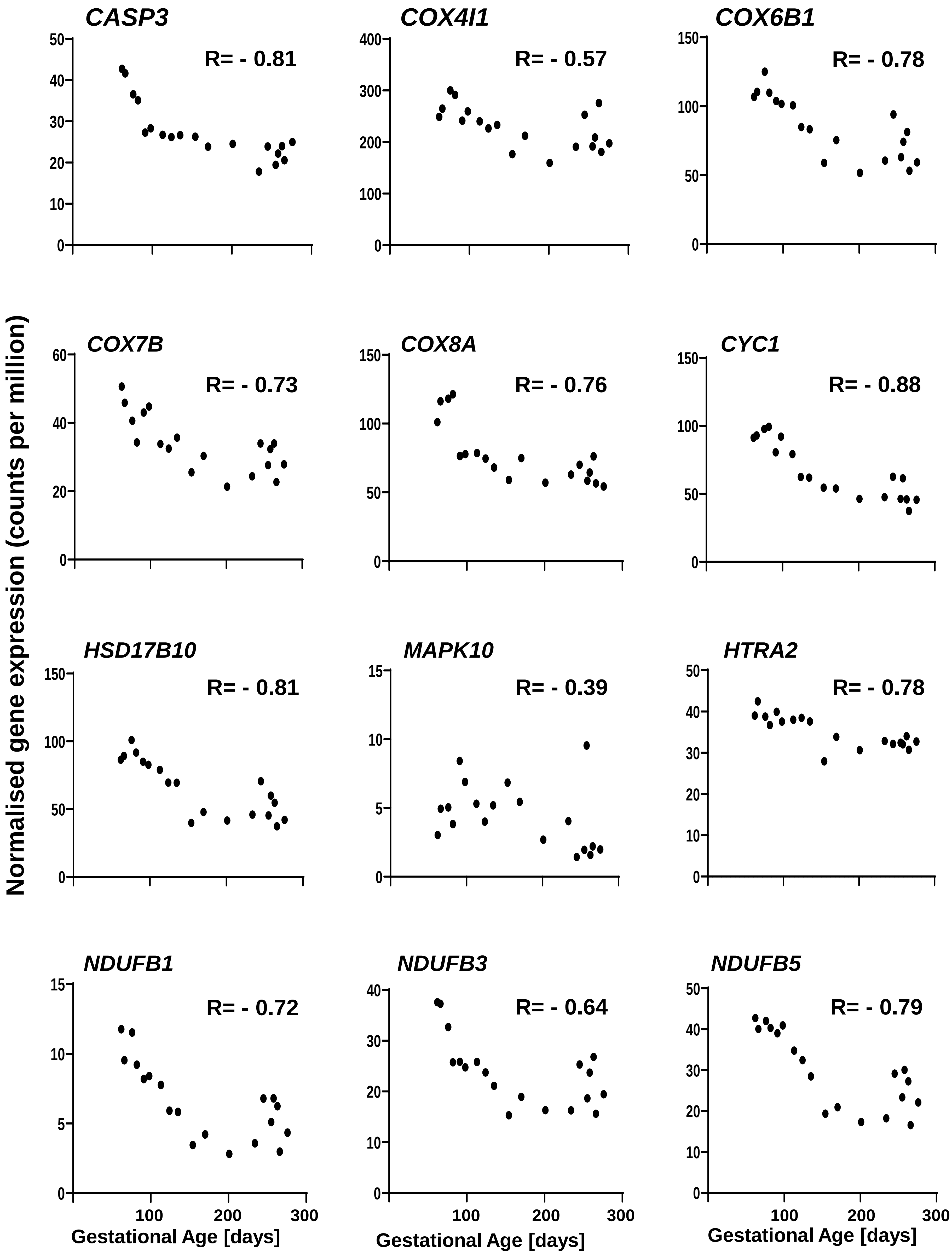
Normalised gene expression (counts per million)
CASP3
R= - 0.81
COX4I1
R= - 0.57
COX6B1
R= - 0.78
COX7B
R= - 0.73
COX8A
R= - 0.76
CYC1
R= - 0.88
HSD17B10
R= - 0.81
MAPK10
R= - 0.39
HTRA2
R= - 0.78
NDUFB1
R= - 0.72
NDUFB3
R= - 0.64
NDUFB5
R= - 0.79
Gestational Age [days]
Gestational Age [days]
Gestational Age [days]

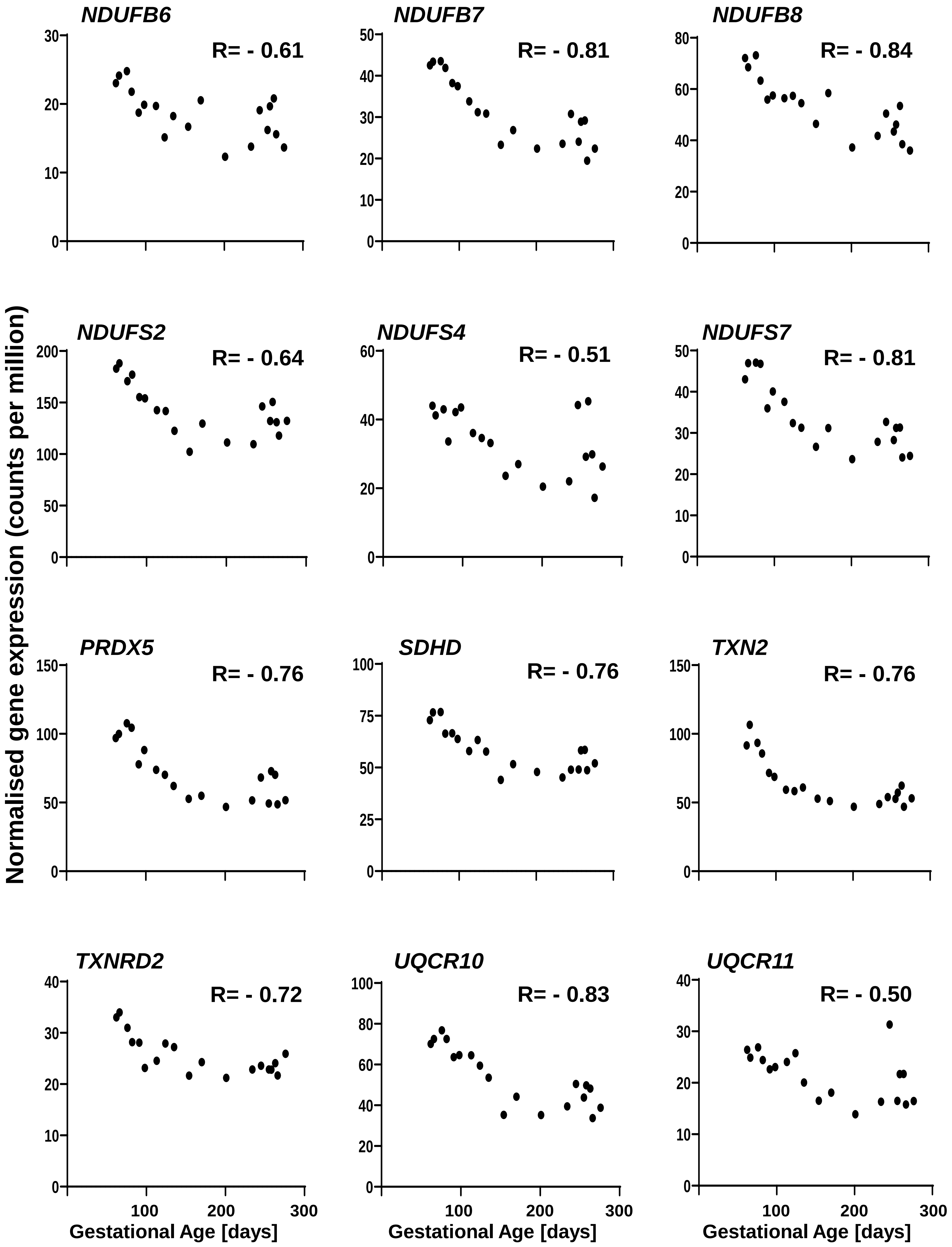

NDUFB6
R= - 0.61
NDUFB7
R= - 0.81
NDUFB8
R= - 0.84
NDUFS2
R= - 0.64
NDUFS4
R= - 0.51
NDUFS7
R= - 0.81
PRDX5
R= - 0.76
SDHD
R= - 0.76
TXN2
R= - 0.76
TXNRD2
R= - 0.72
UQCR10
R= - 0.83
UQCR11
R= - 0.50
Normalised gene expression (counts per million)
Gestational Age [days]

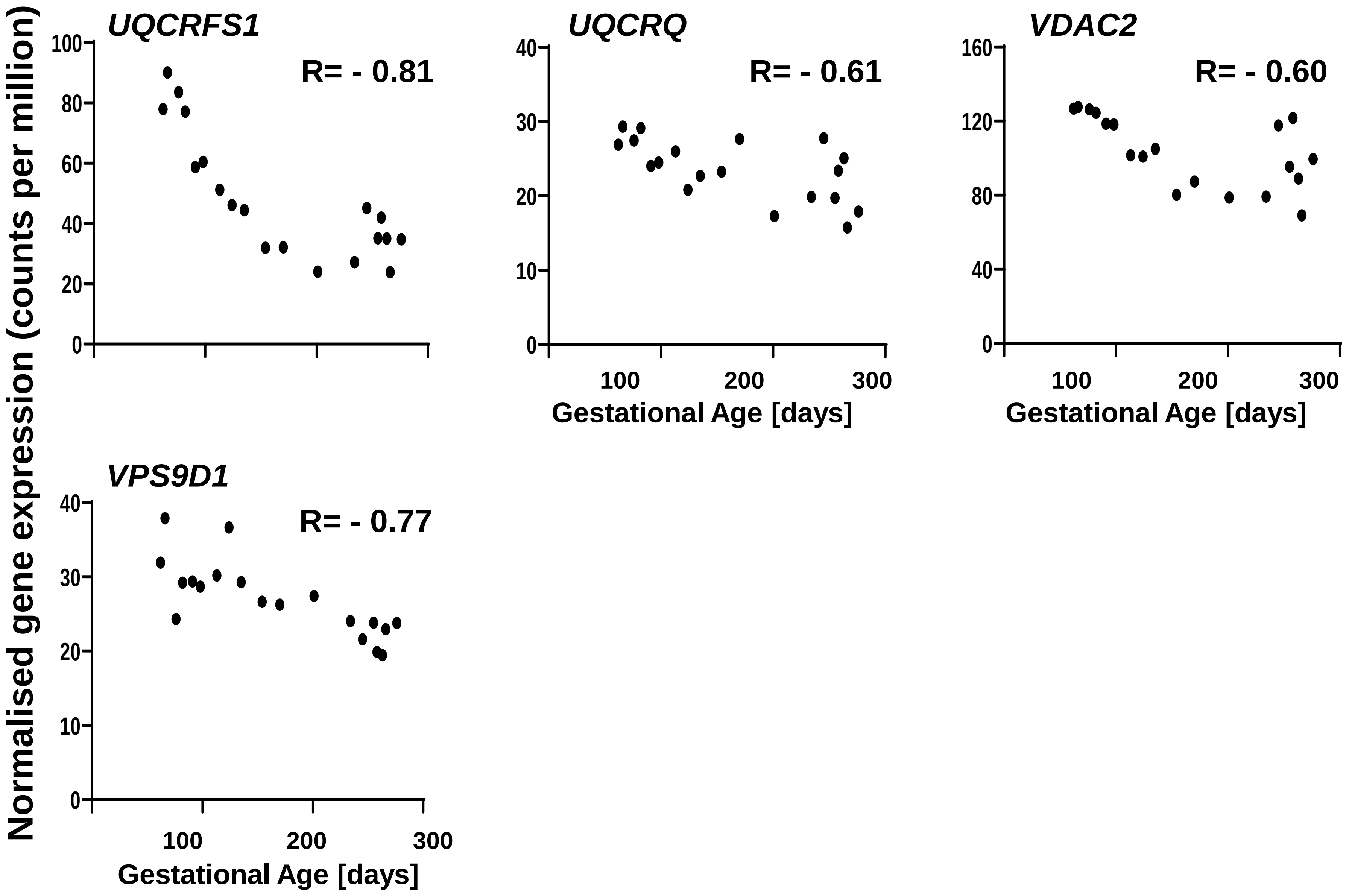
Normalised gene expression (counts per million)
UQCRFS1
R= - 0.81
UQCRQ
R= - 0.61
VDAC2
R= - 0.60
VPS9D1
R= - 0.77
Gestational Age [days]
Gestational Age [days]
Gestational Age [days]

S7 Fig. Scatterplots showing the expression pattern of mitochondrial DNA-encoded genes across gestation in bovine fetal ovaries

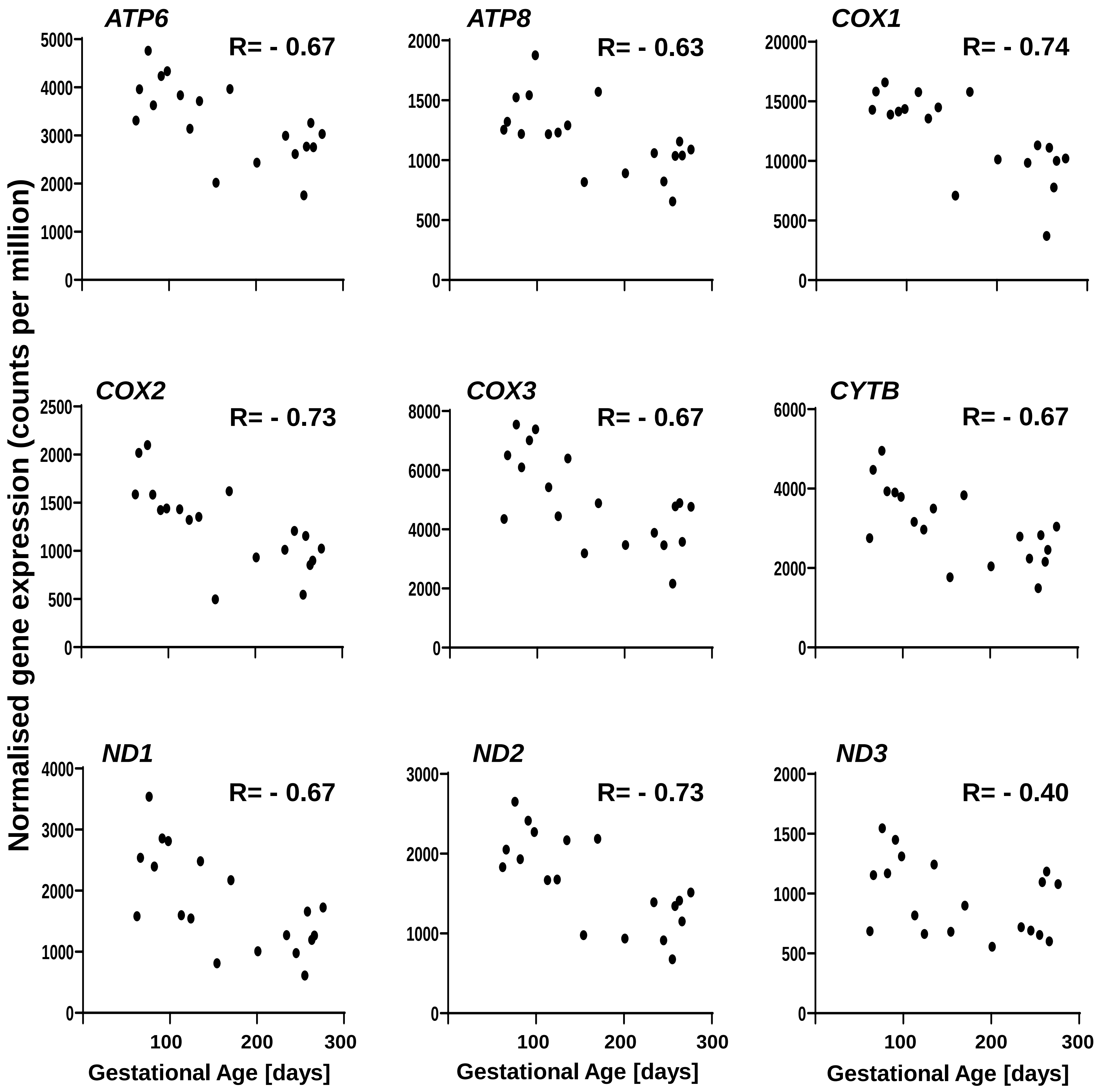

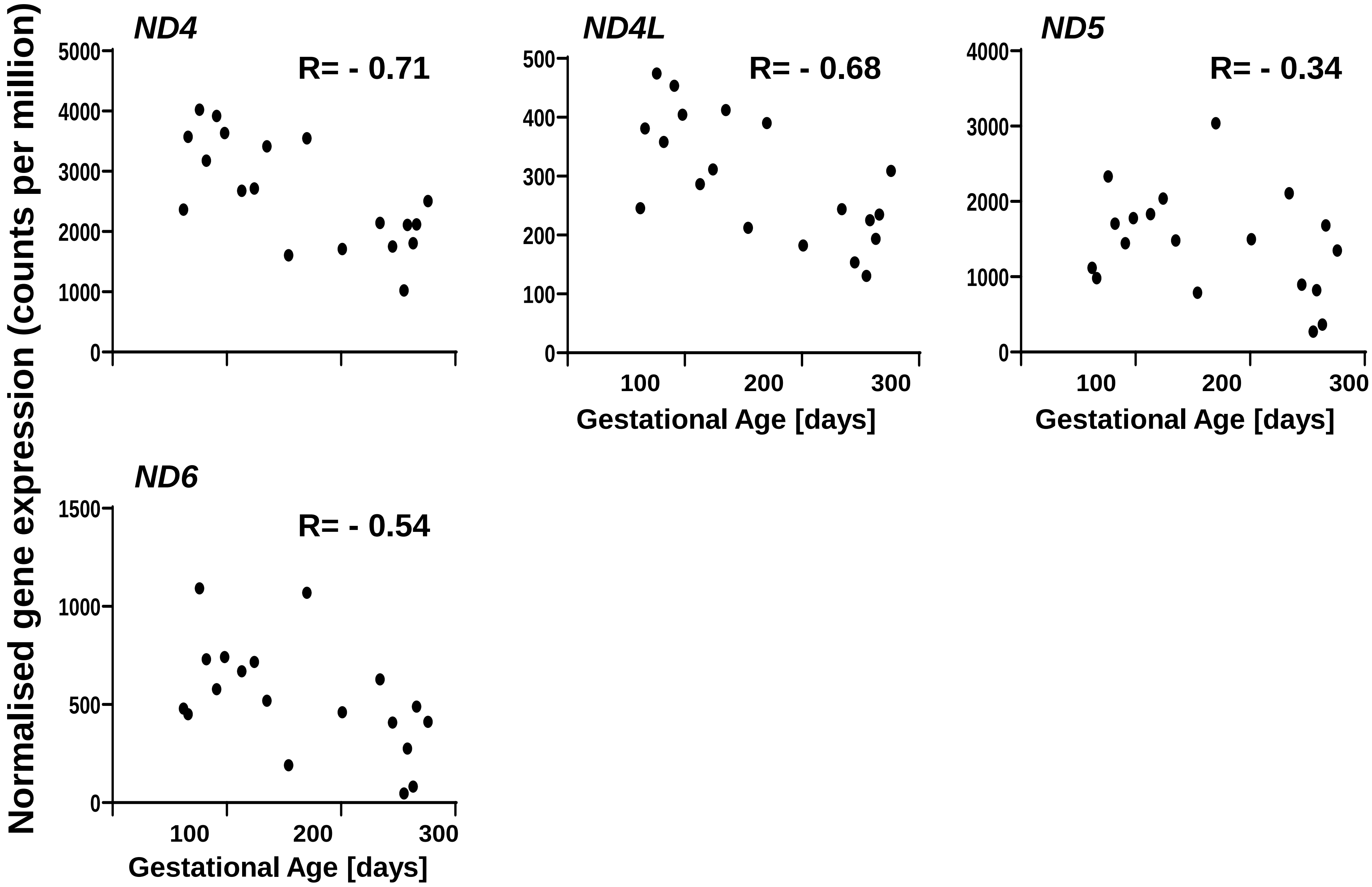
ND4
R= - 0.71
ND4L
R= - 0.68
ND5
R= - 0.34
ND6
R= - 0.54
Normalised gene expression (counts per million)
Gestational Age [days]
0
1000
2000
3000
4000
5000
100
200
300
400
500
1500
